# Supplementary figures and images for: Zika virus causes placental pyroptosis and associated adverse fetal outcomes by activating GSDME (part 1 of 4)
Source: eLife. 2022 Aug 16;11:e73792. doi: 10.7554/eLife.73792 (PMC9381041; doi:10.7554/eLife.73792)

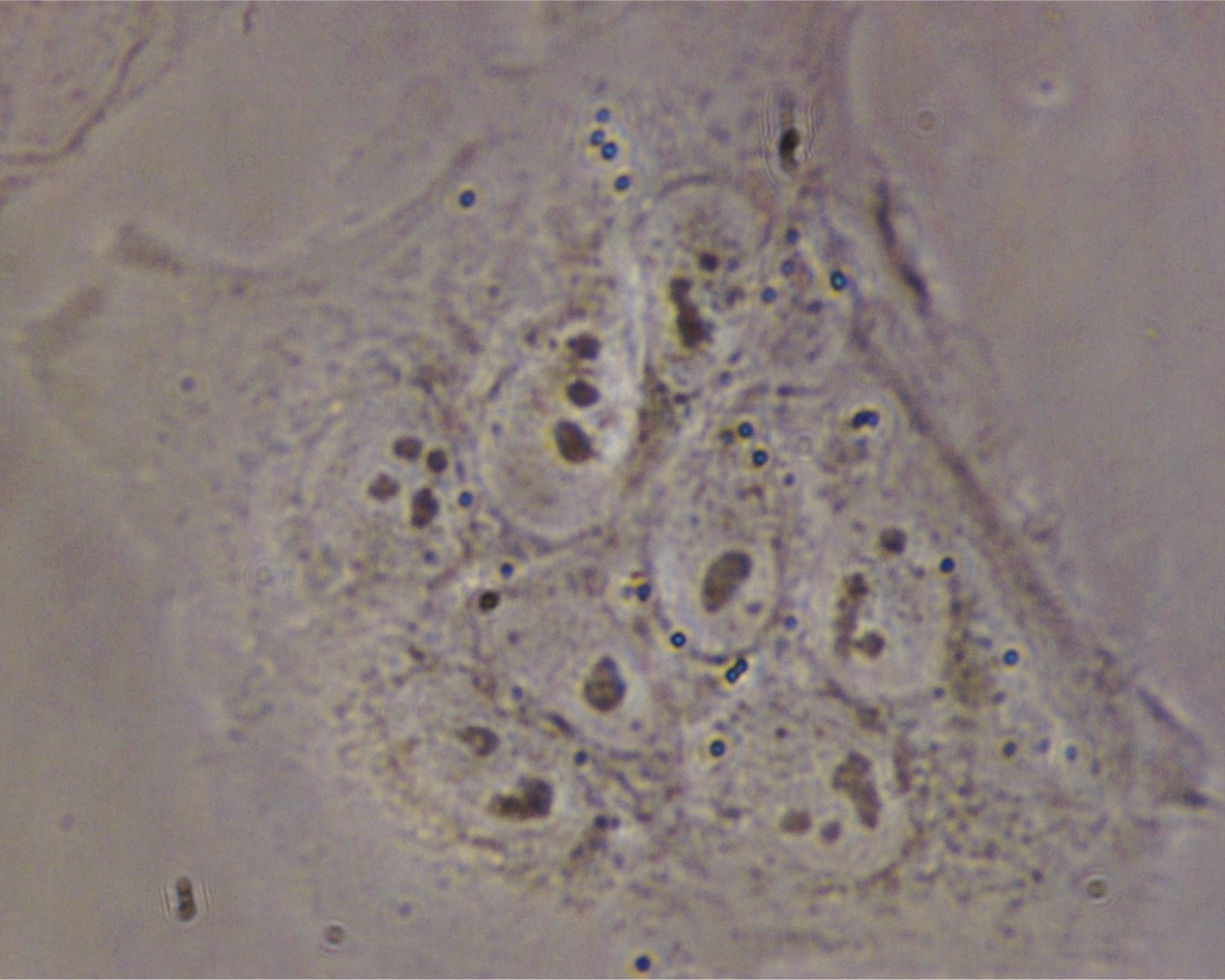

Supplement: Figure 1—source data 1. [file elife-73792-fig1-data1.zip › Figure 1-source data 1/Fig 1A/Fig 1A mock.tif]

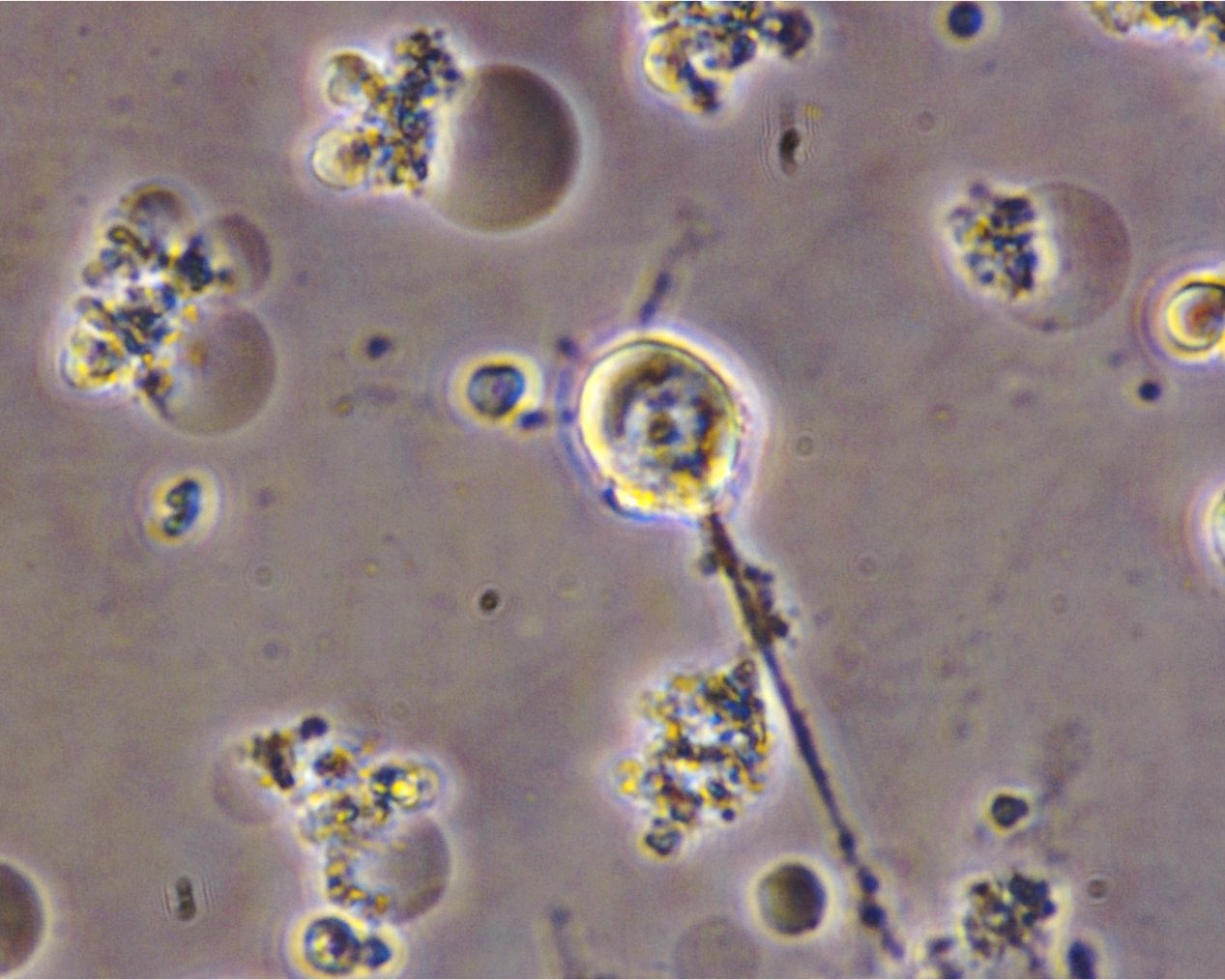

Supplement: Figure 1—source data 1. [file elife-73792-fig1-data1.zip › Figure 1-source data 1/Fig 1A/Fig 1A zikv.tif]

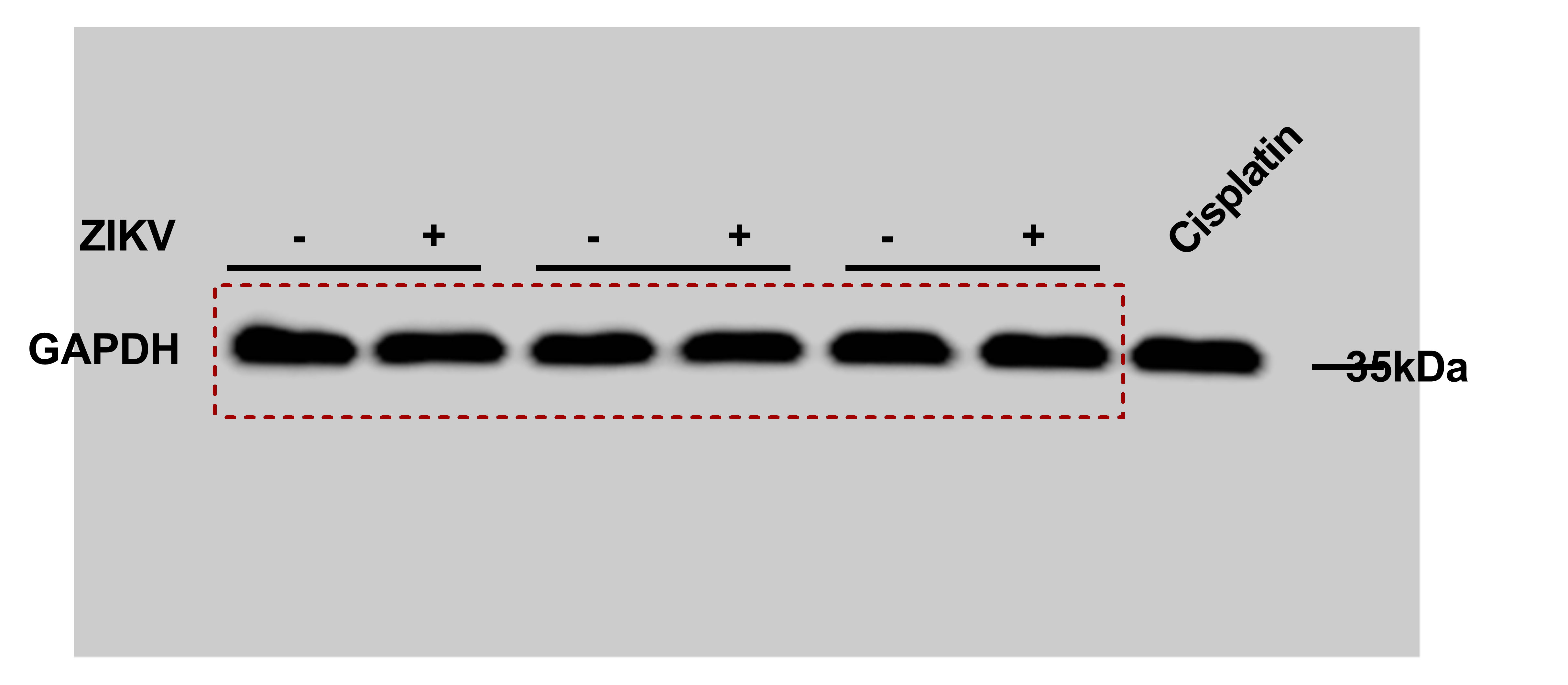

Supplement: Figure 1—source data 1. [file elife-73792-fig1-data1.zip › Figure 1-source data 1/Fig 1C/Figure 1C GAPDH-labeled.tif]

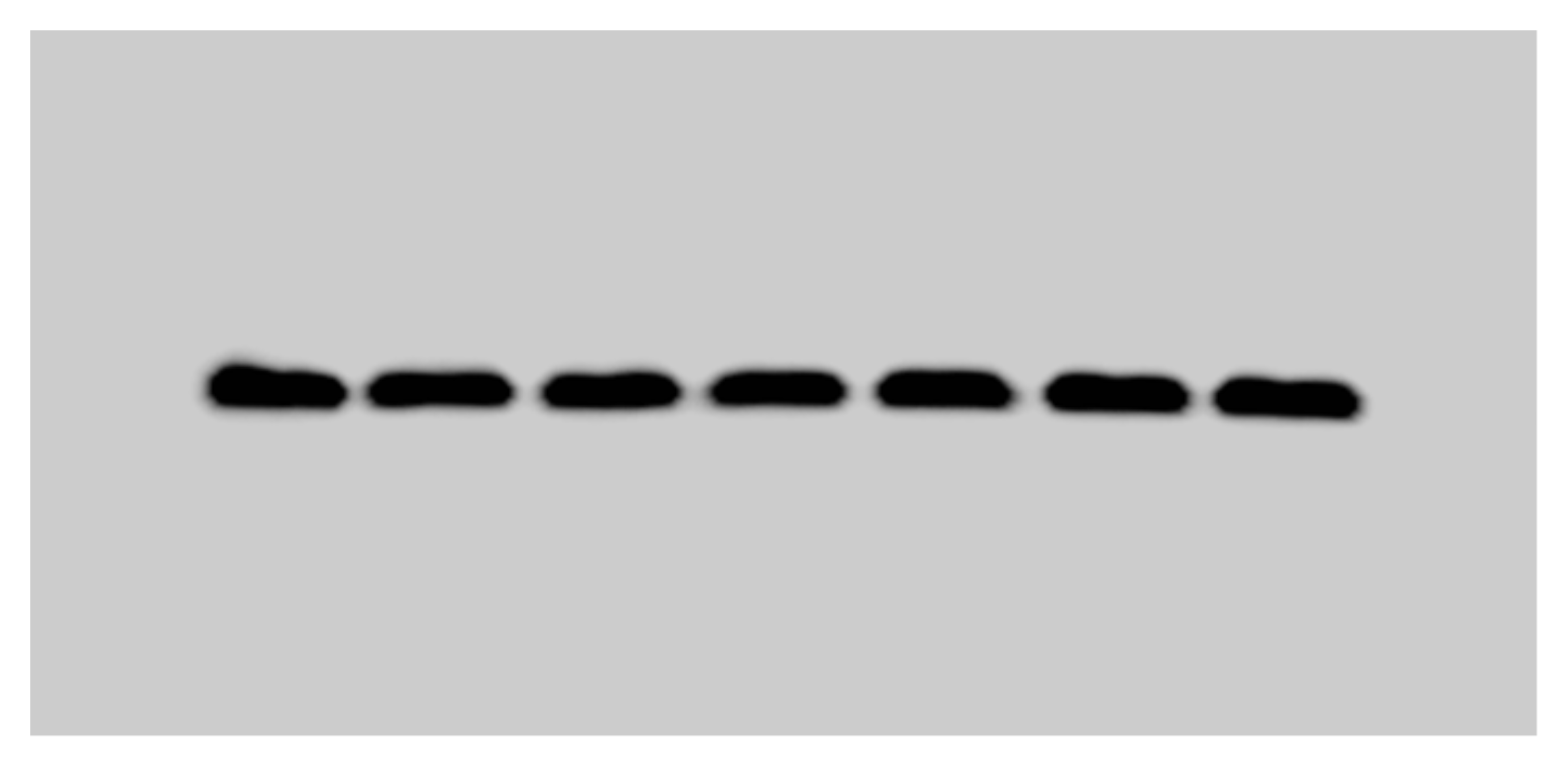

Supplement: Figure 1—source data 1. [file elife-73792-fig1-data1.zip › Figure 1-source data 1/Fig 1C/Figure 1C GAPDH-raw.tif]

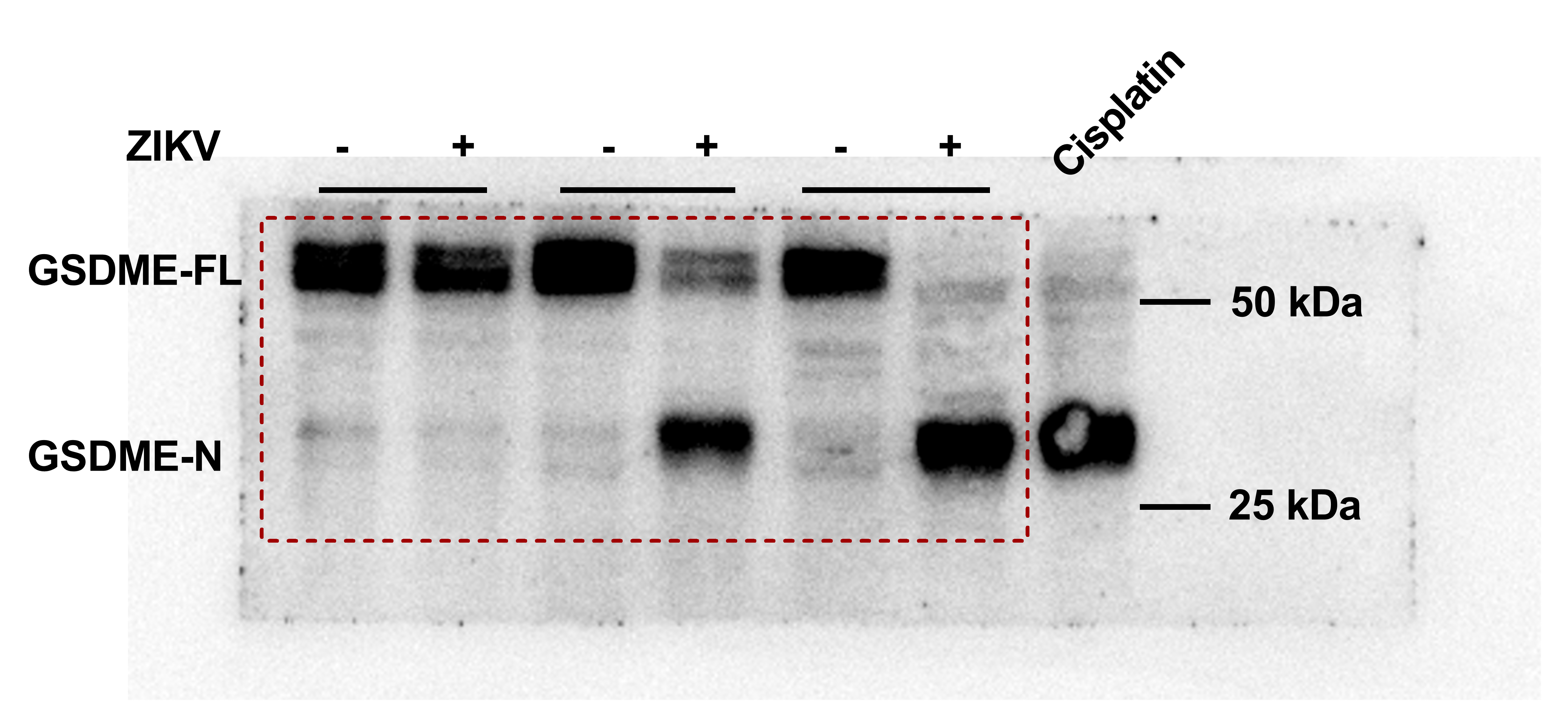

Supplement: Figure 1—source data 1. [file elife-73792-fig1-data1.zip › Figure 1-source data 1/Fig 1C/Figure 1C GSDME-labeled.tif]

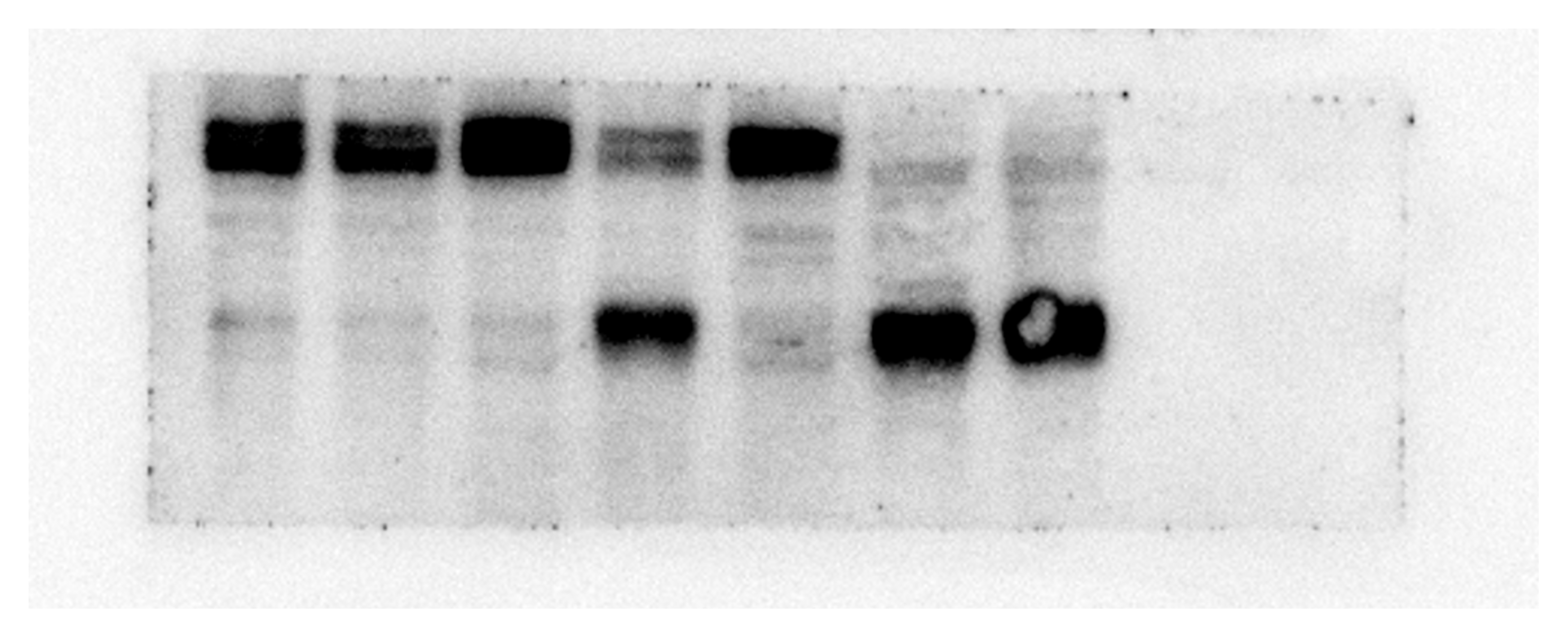

Supplement: Figure 1—source data 1. [file elife-73792-fig1-data1.zip › Figure 1-source data 1/Fig 1C/Figure 1C GSDME-RAW.tif]

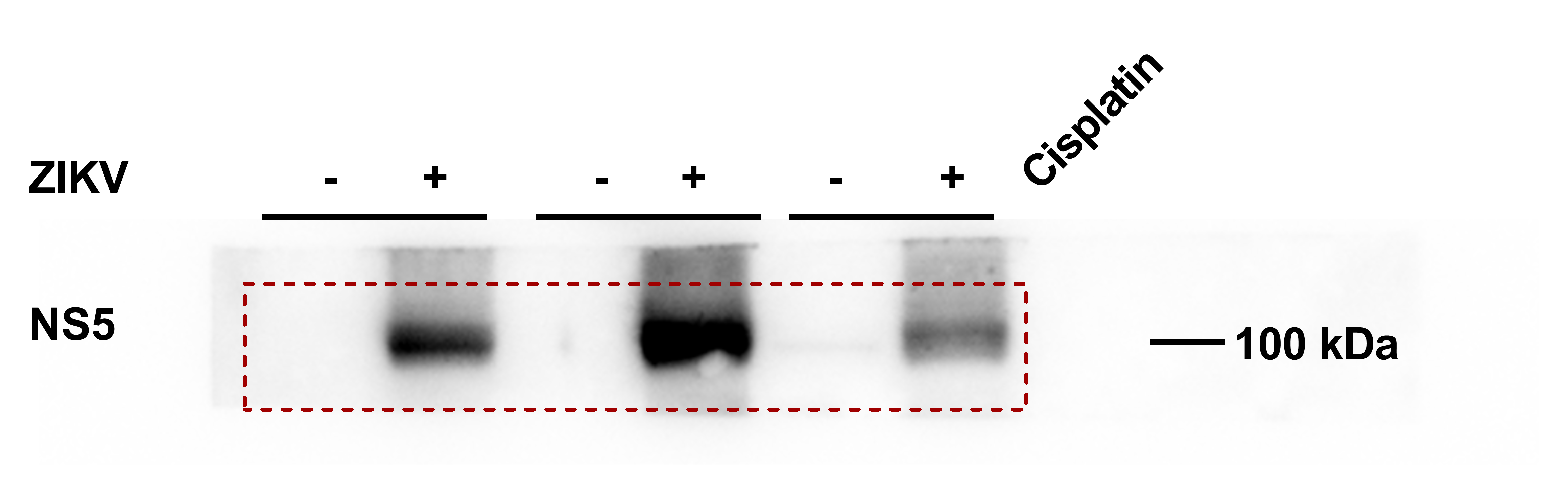

Supplement: Figure 1—source data 1. [file elife-73792-fig1-data1.zip › Figure 1-source data 1/Fig 1C/Figure 1C NS5-labeled.tif]

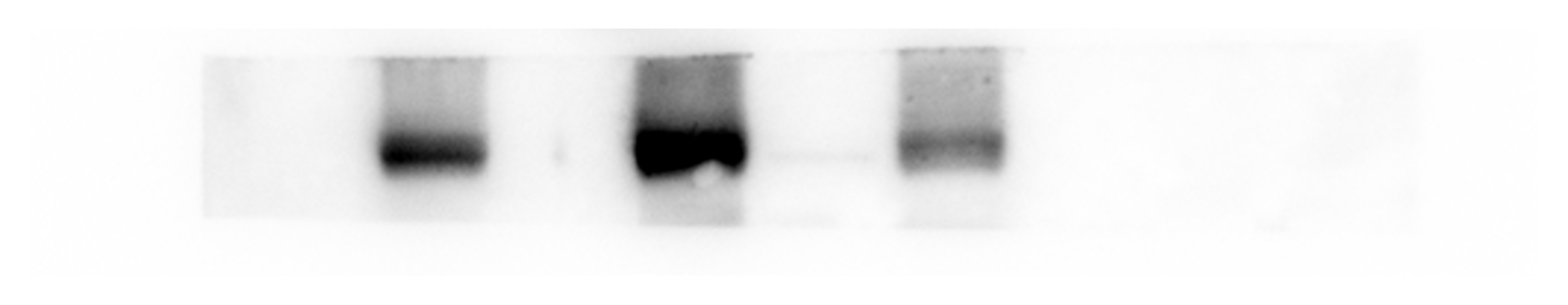

Supplement: Figure 1—source data 1. [file elife-73792-fig1-data1.zip › Figure 1-source data 1/Fig 1C/Figure 1C NS5-raw.tif]

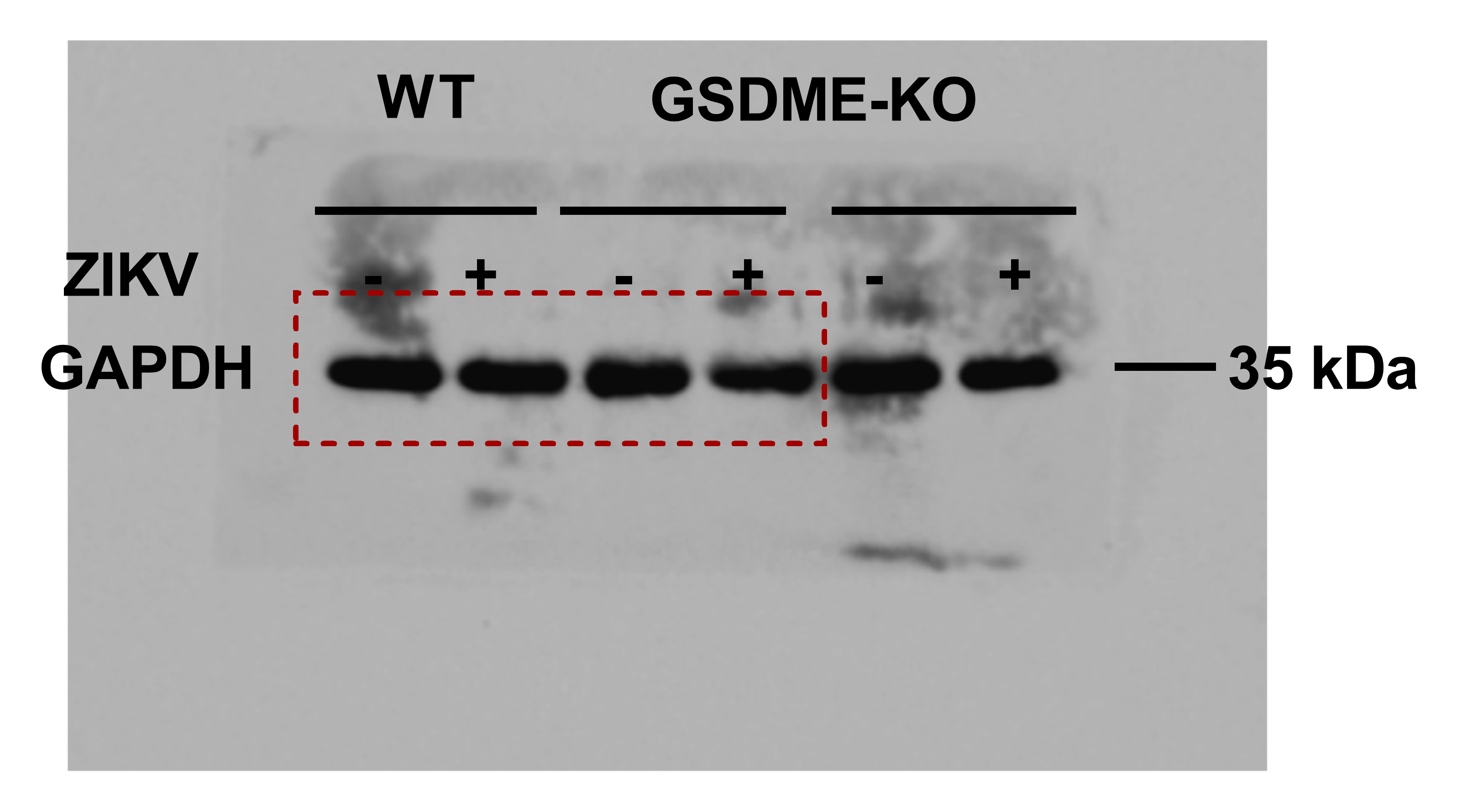

Supplement: Figure 1—source data 1. [file elife-73792-fig1-data1.zip › Figure 1-source data 1/Fig 1D/Figure 1D GAPDH-labeled.tif]

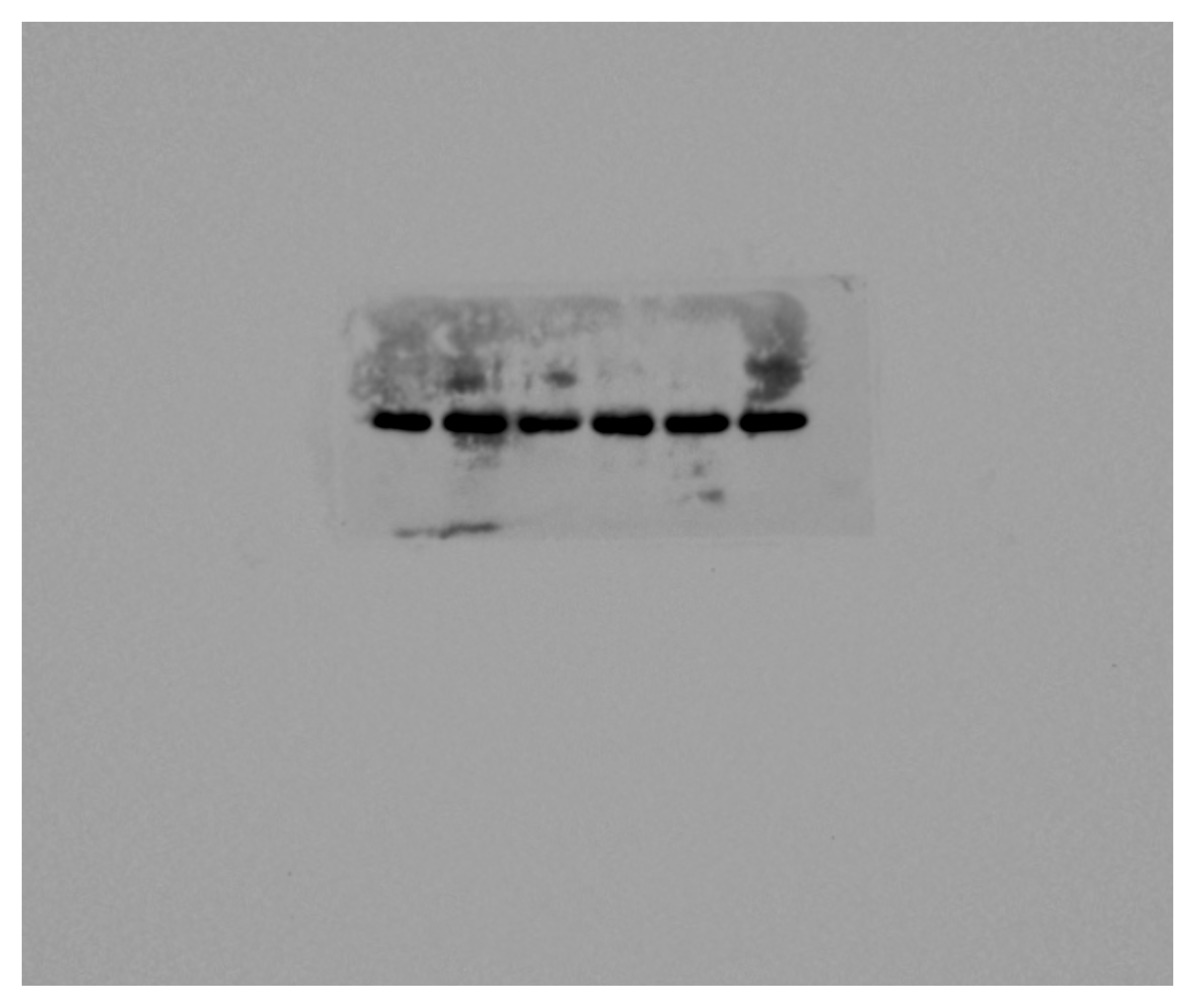

Supplement: Figure 1—source data 1. [file elife-73792-fig1-data1.zip › Figure 1-source data 1/Fig 1D/Figure 1D GAPDH-raw.tif]

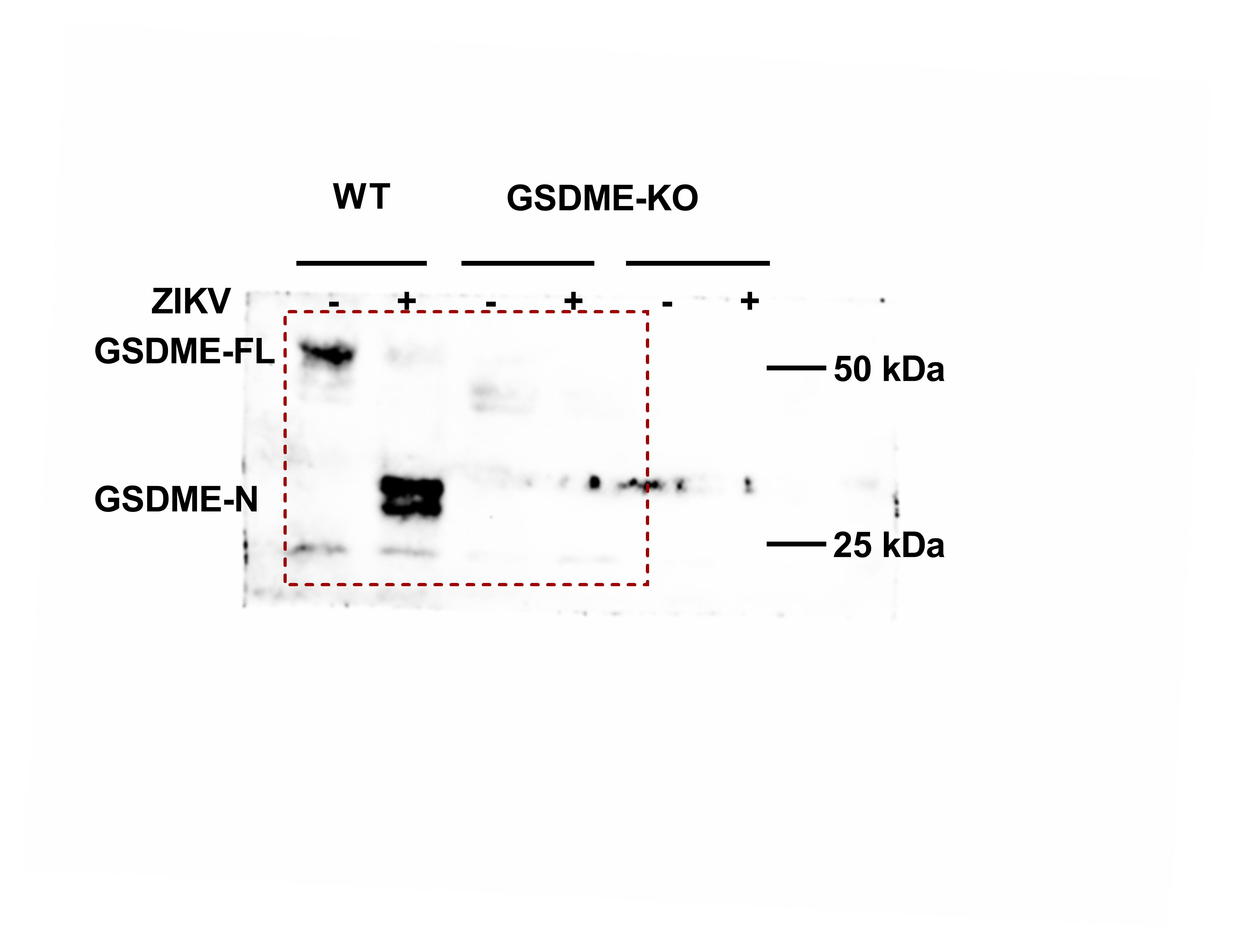

Supplement: Figure 1—source data 1. [file elife-73792-fig1-data1.zip › Figure 1-source data 1/Fig 1D/Figure 1D GSDME-labeled.tif]

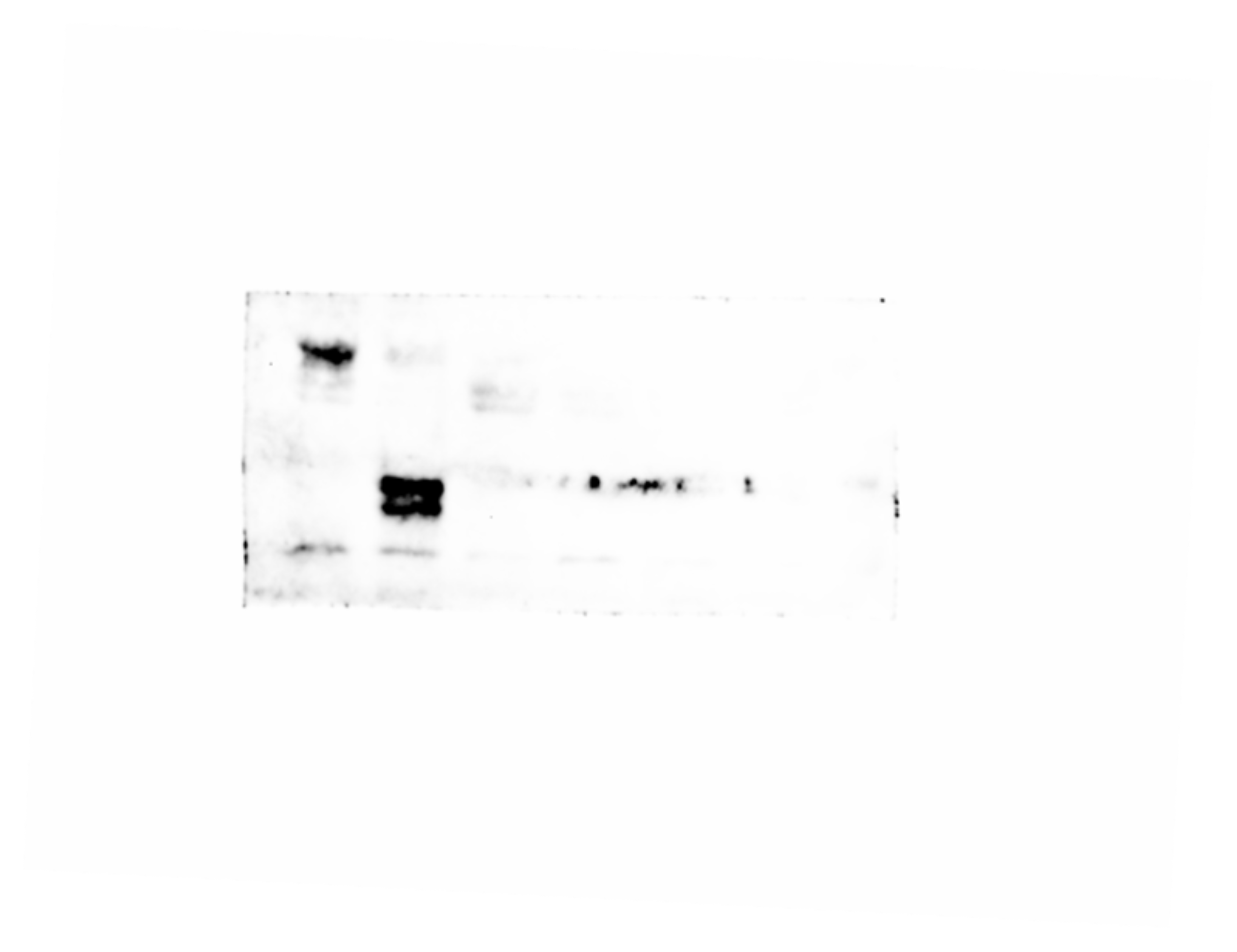

Supplement: Figure 1—source data 1. [file elife-73792-fig1-data1.zip › Figure 1-source data 1/Fig 1D/Figure 1D GSDME-raw.tif]

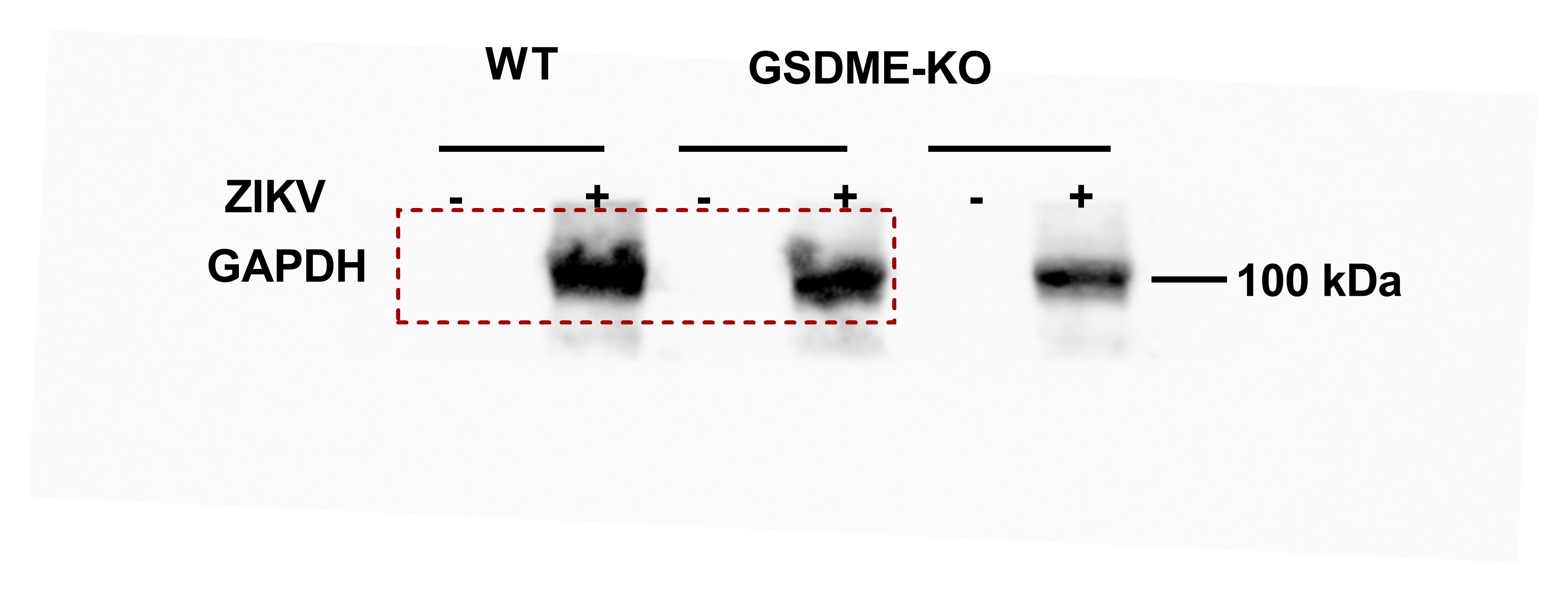

Supplement: Figure 1—source data 1. [file elife-73792-fig1-data1.zip › Figure 1-source data 1/Fig 1D/Figure 1D ns5-labeled.tif]

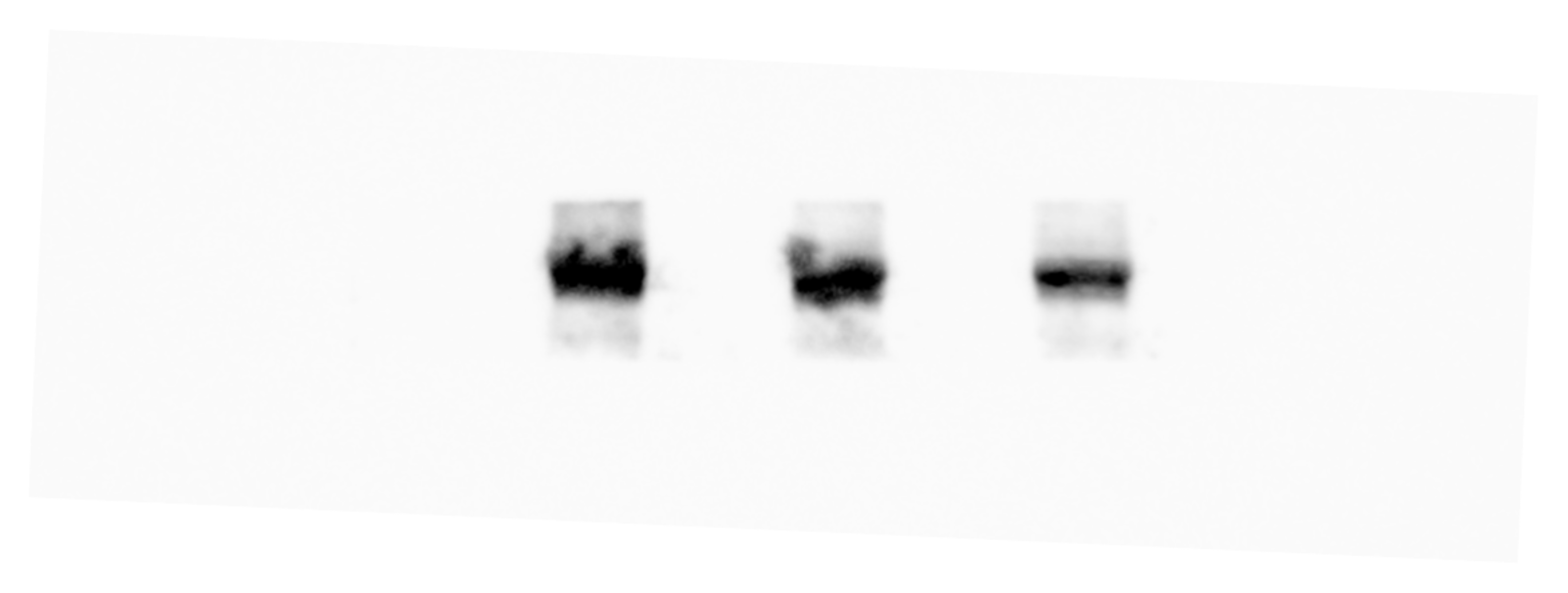

Supplement: Figure 1—source data 1. [file elife-73792-fig1-data1.zip › Figure 1-source data 1/Fig 1D/Figure 1D ns5-raw.tif]

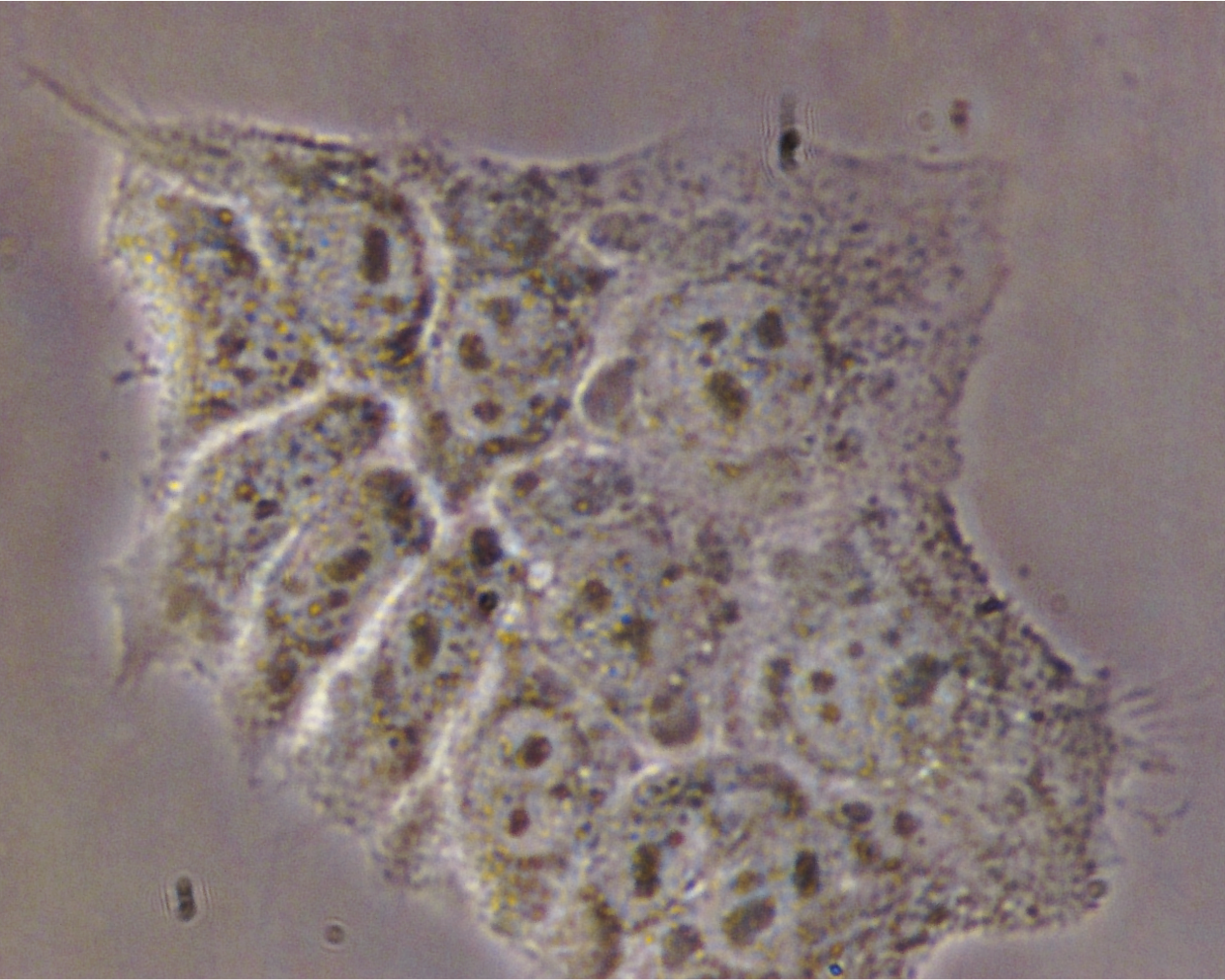

Supplement: Figure 1—source data 1. [file elife-73792-fig1-data1.zip › Figure 1-source data 1/Fig 1E/fig 1e gsdme-ko mock.tif]

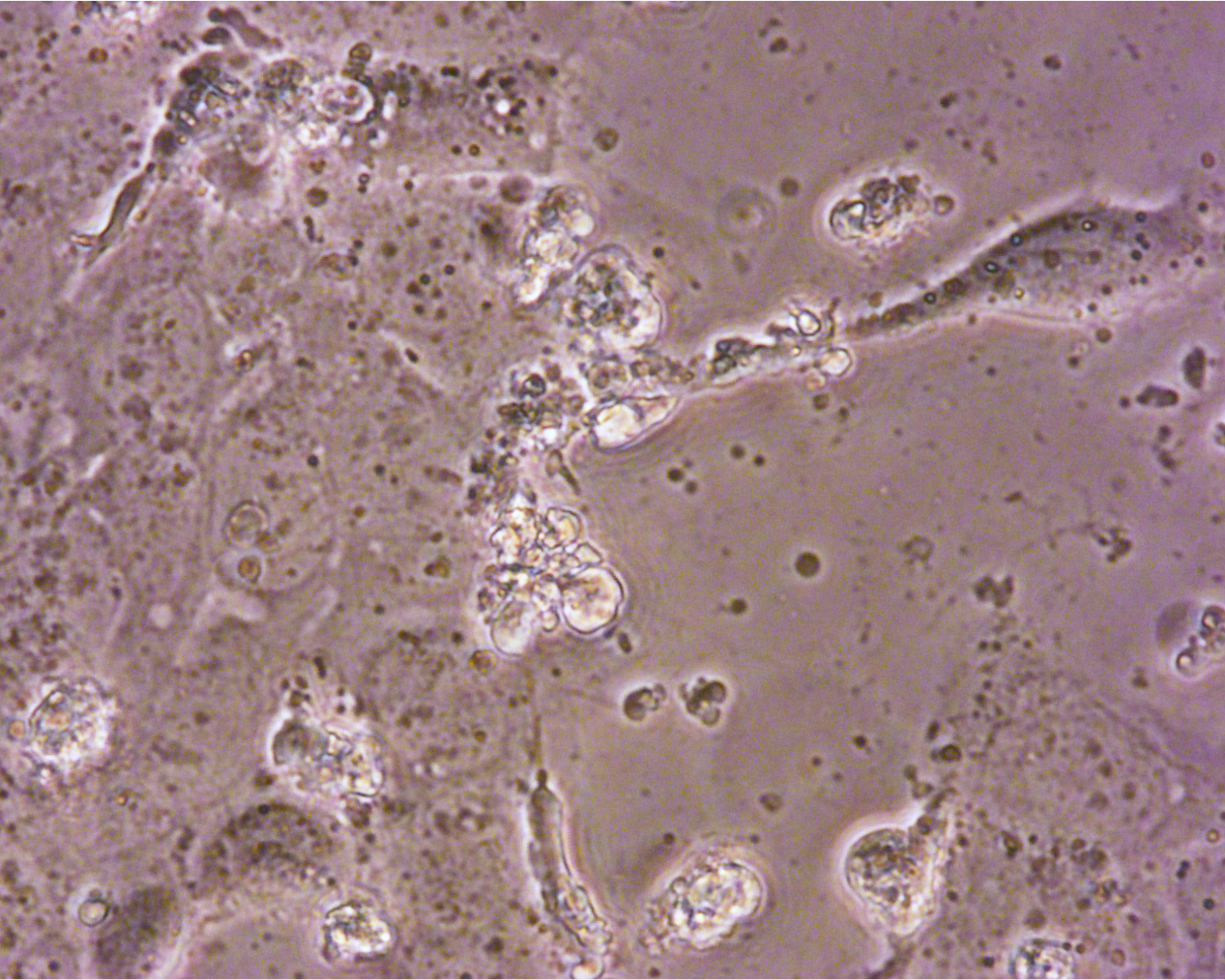

Supplement: Figure 1—source data 1. [file elife-73792-fig1-data1.zip › Figure 1-source data 1/Fig 1E/fig 1e gsdme-ko zikv.tif]

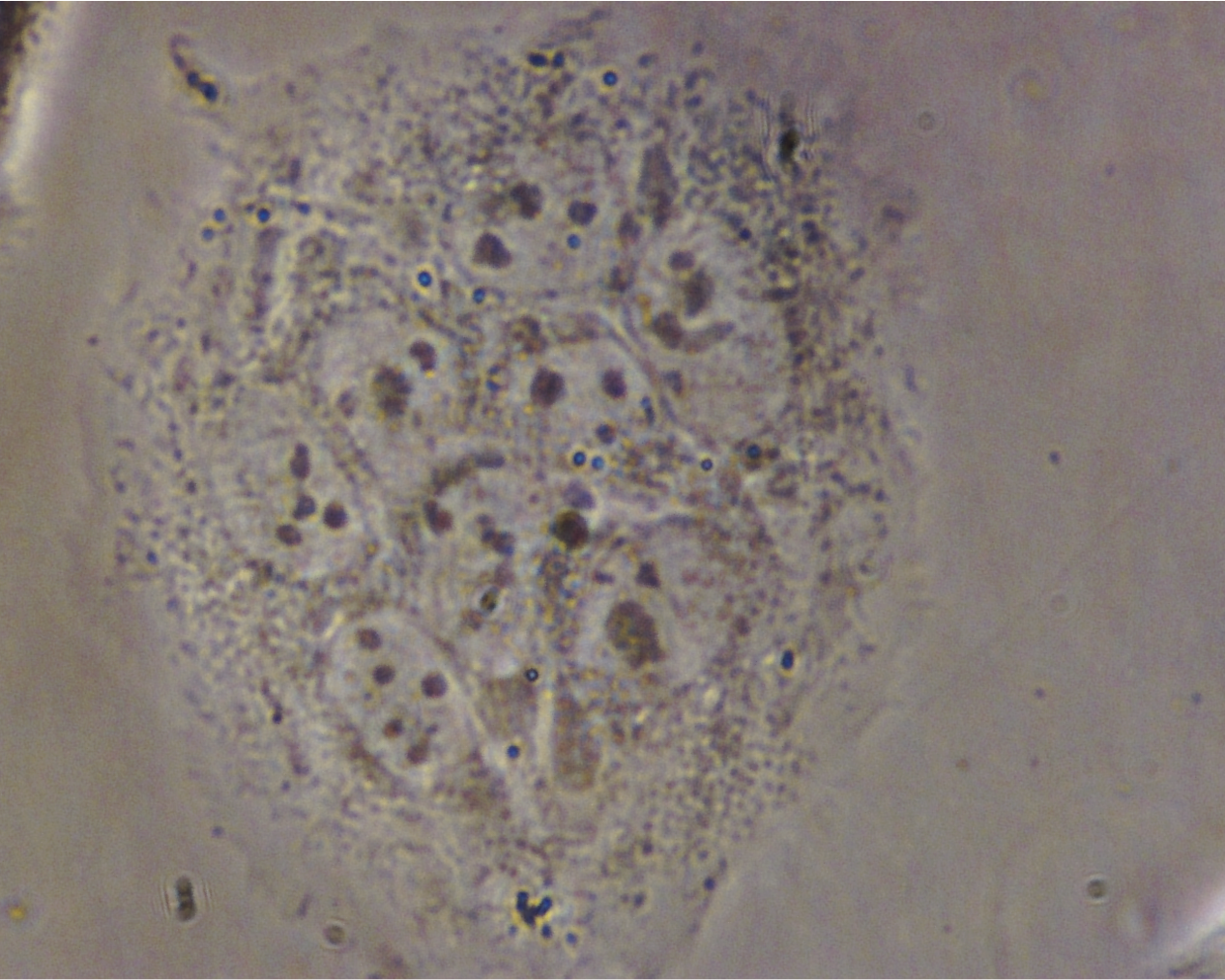

Supplement: Figure 1—source data 1. [file elife-73792-fig1-data1.zip › Figure 1-source data 1/Fig 1E/fig 1e jeg3 mock.tif]

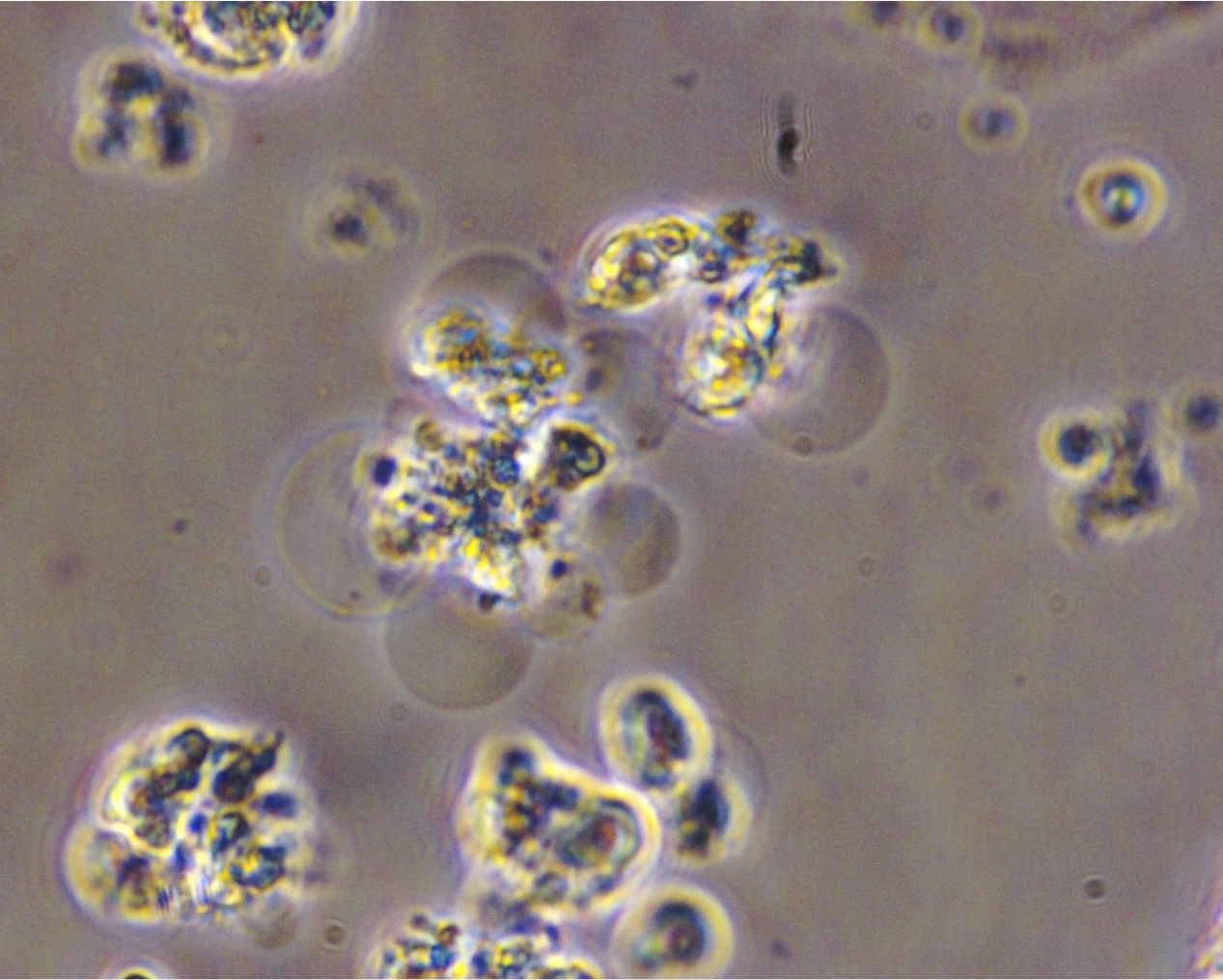

Supplement: Figure 1—source data 1. [file elife-73792-fig1-data1.zip › Figure 1-source data 1/Fig 1E/fig 1e jeg3 zikv.tif]

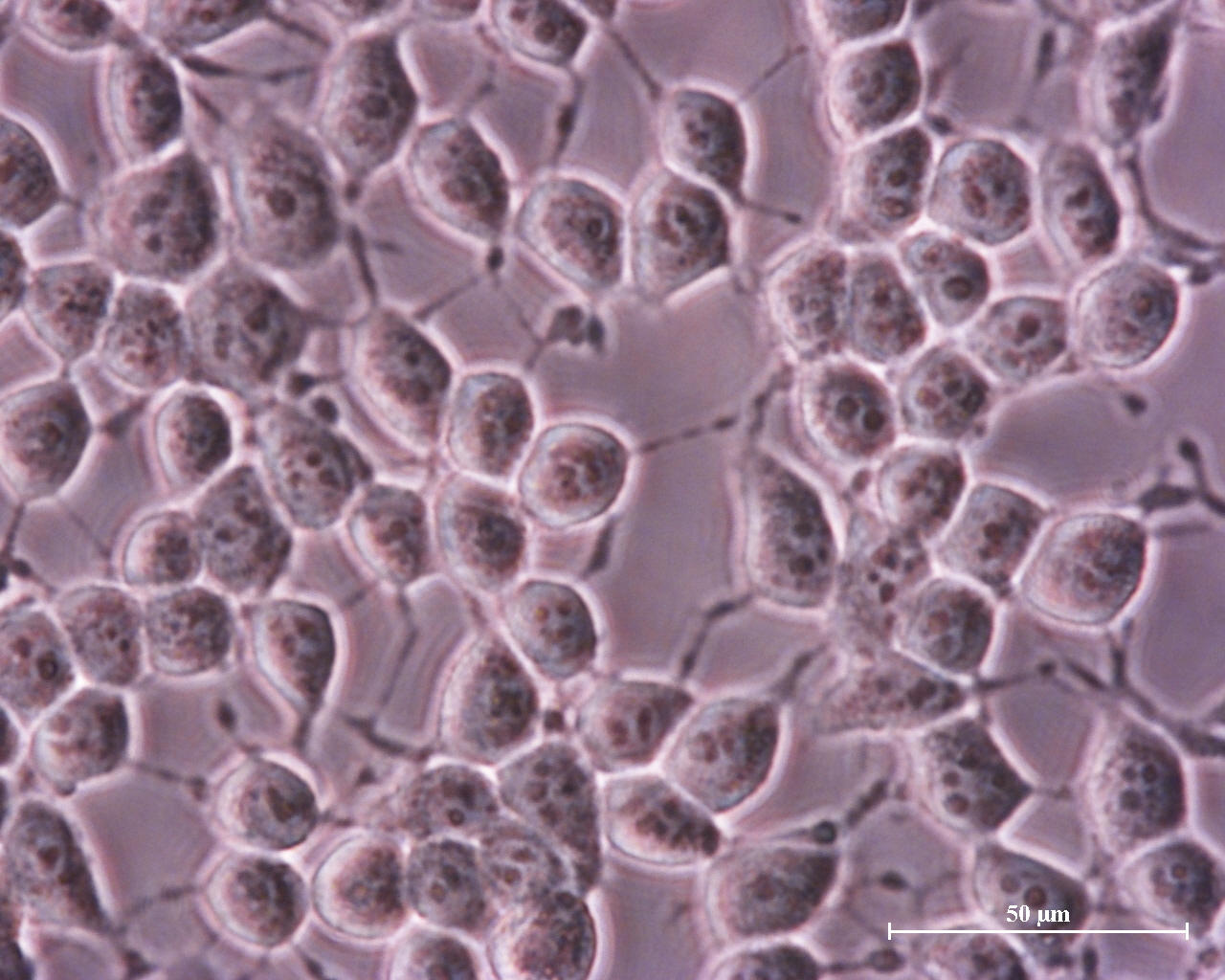

Supplement: Figure 2—source data 1. [file elife-73792-fig2-data1.zip › Figure 2-source data 1/Fig 2A/293 mock.tif]

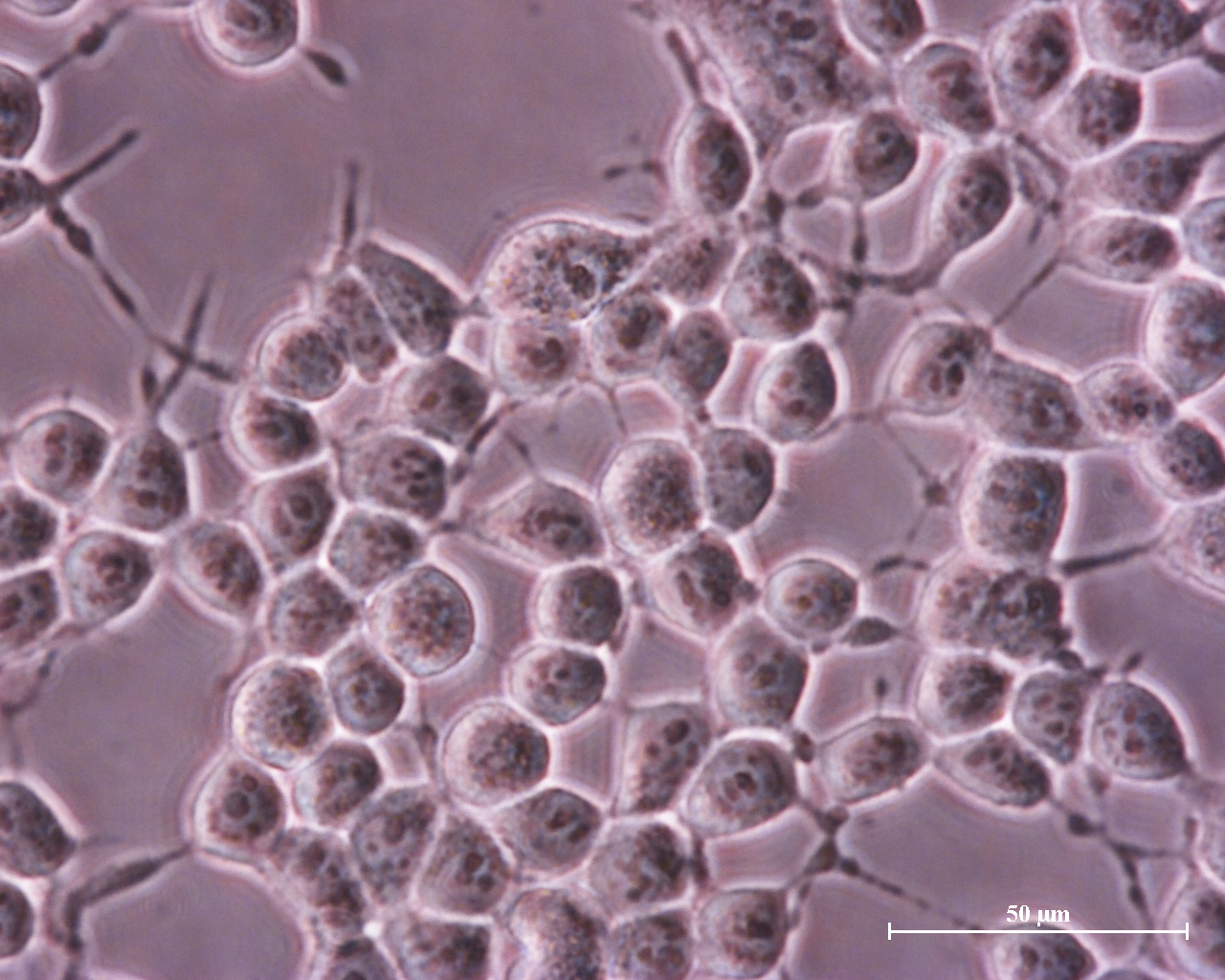

Supplement: Figure 2—source data 1. [file elife-73792-fig2-data1.zip › Figure 2-source data 1/Fig 2A/293 zikv.tif]

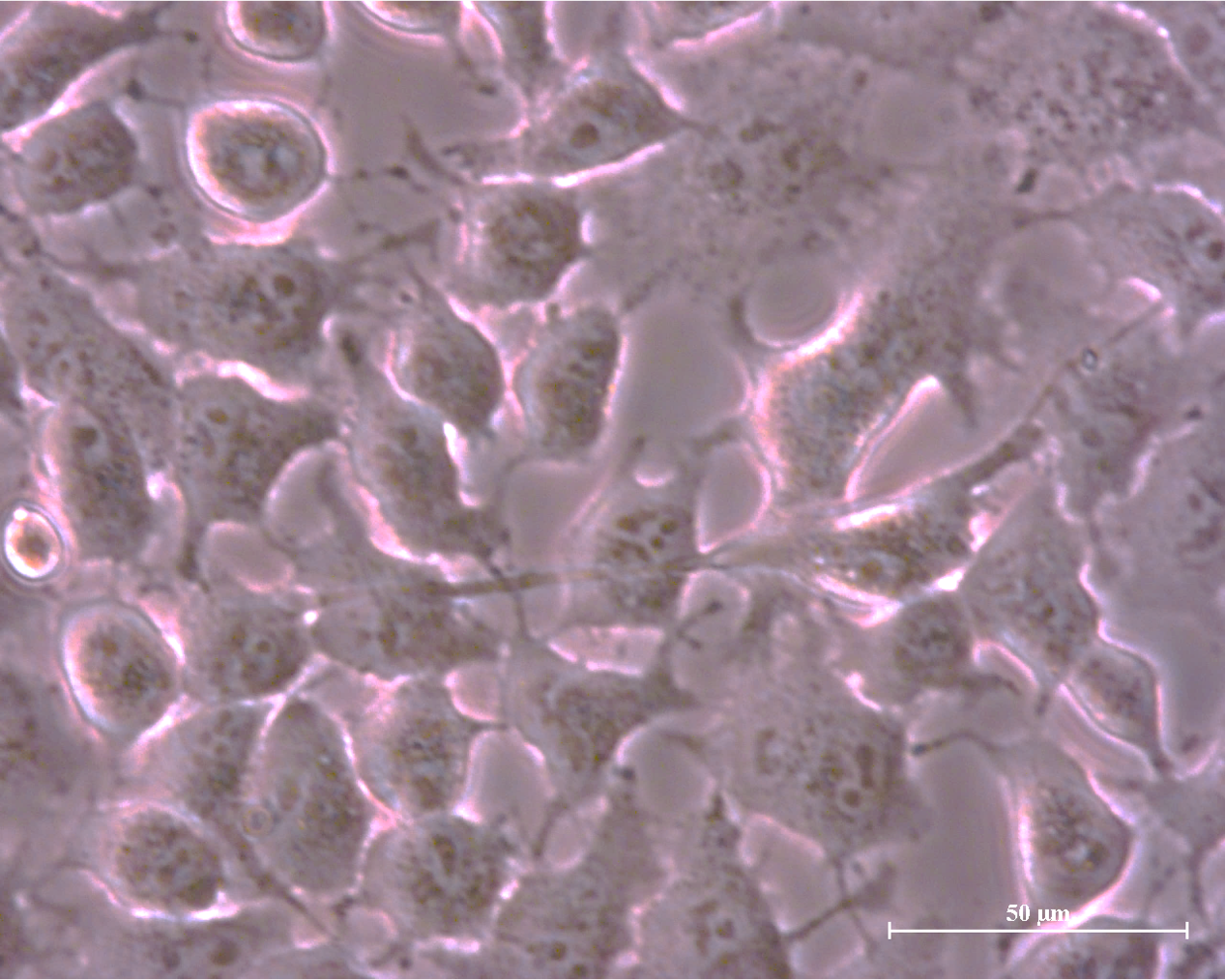

Supplement: Figure 2—source data 1. [file elife-73792-fig2-data1.zip › Figure 2-source data 1/Fig 2A/a549 mock.tif]

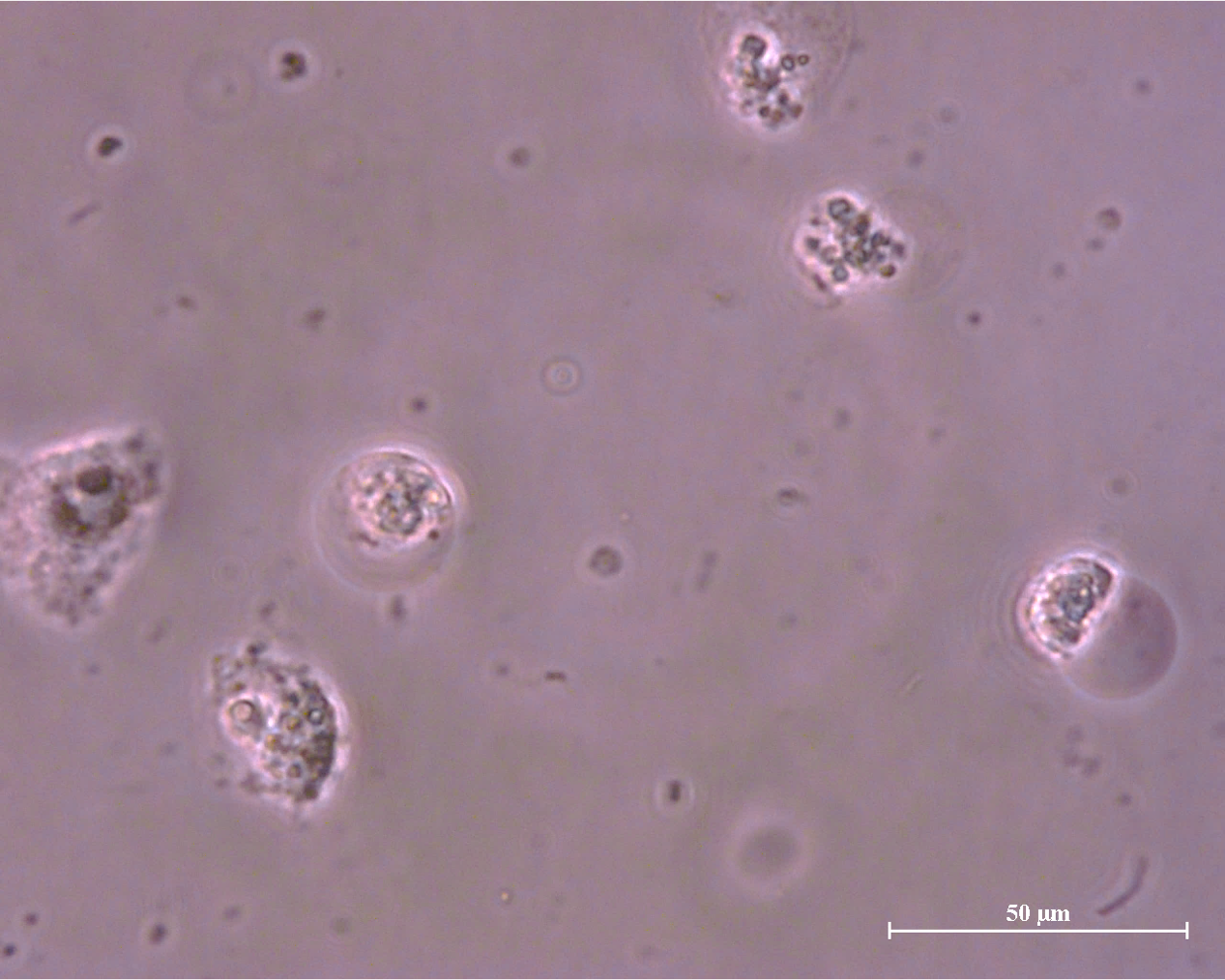

Supplement: Figure 2—source data 1. [file elife-73792-fig2-data1.zip › Figure 2-source data 1/Fig 2A/a549 zikv.tif]

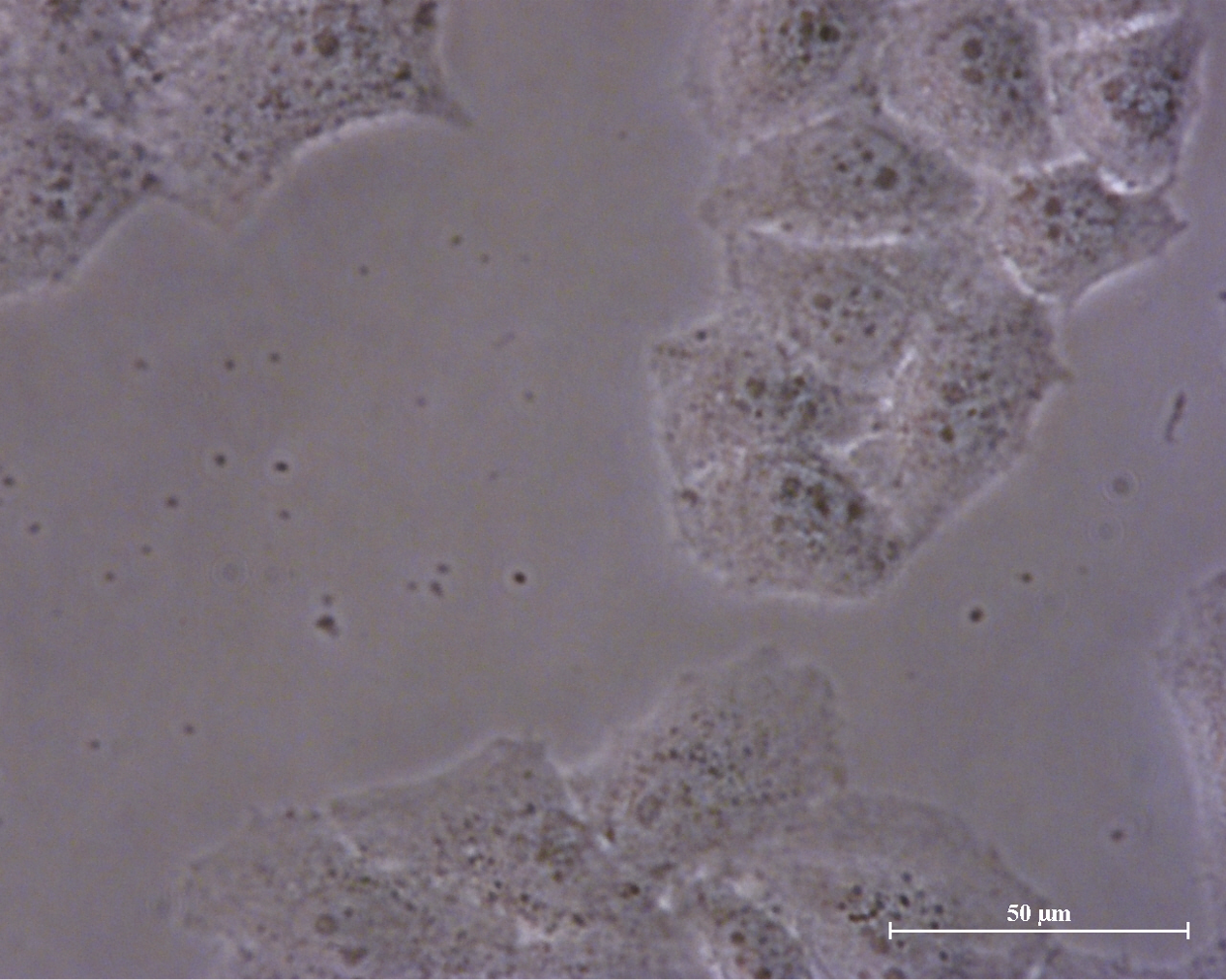

Supplement: Figure 2—source data 1. [file elife-73792-fig2-data1.zip › Figure 2-source data 1/Fig 2A/hela mock.tif]

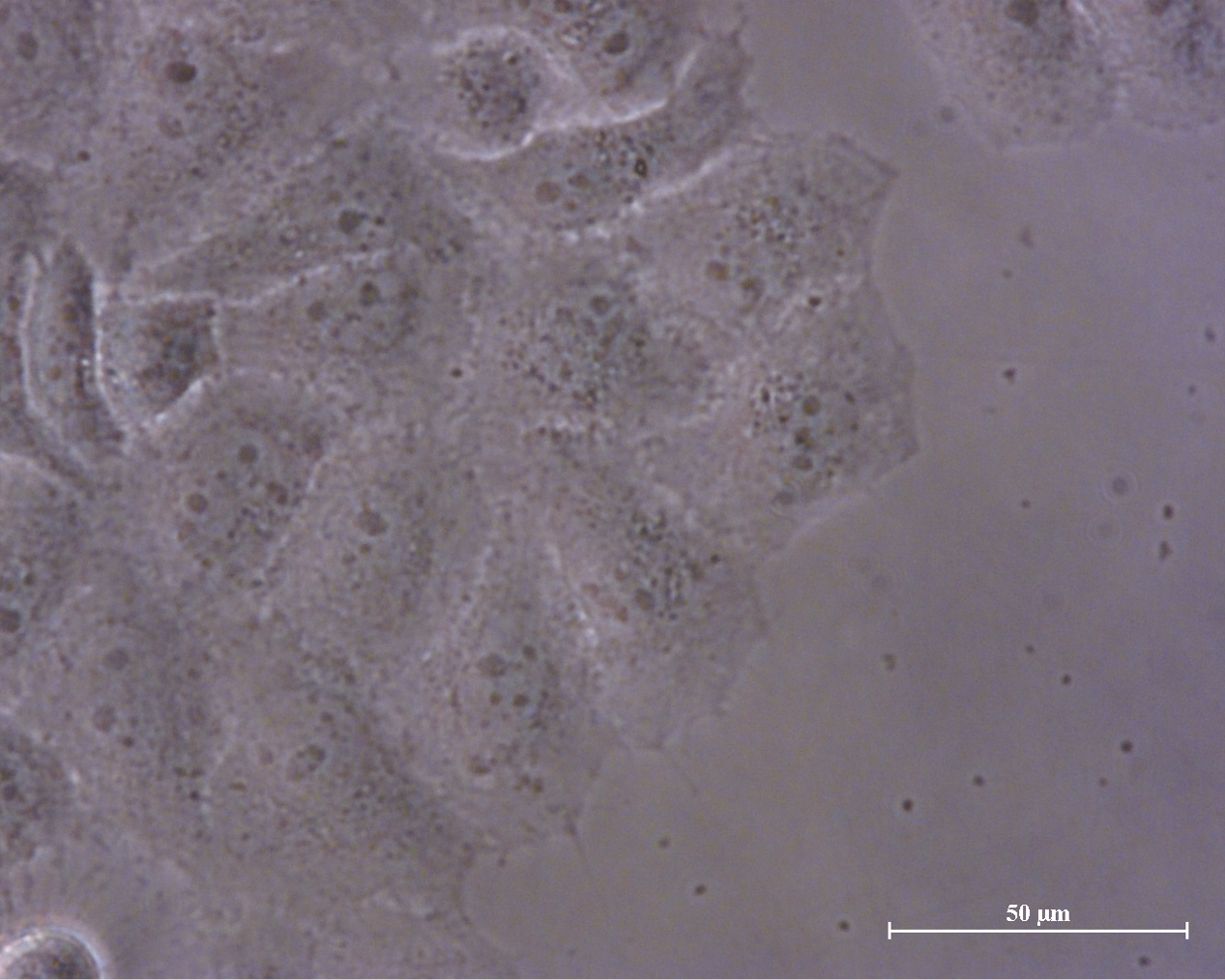

Supplement: Figure 2—source data 1. [file elife-73792-fig2-data1.zip › Figure 2-source data 1/Fig 2A/hela zikv.tif]

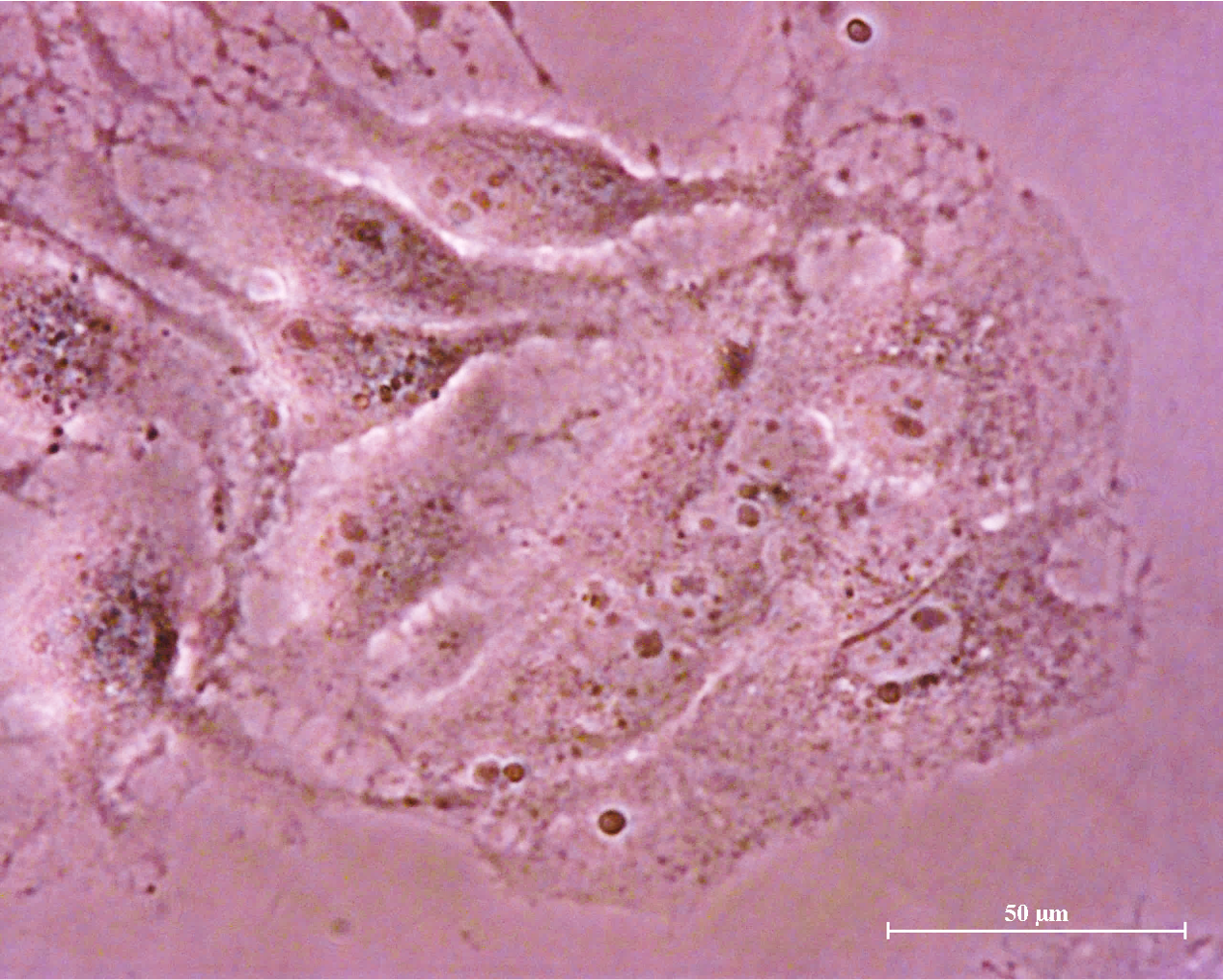

Supplement: Figure 2—source data 1. [file elife-73792-fig2-data1.zip › Figure 2-source data 1/Fig 2A/huh7 mock.tif]

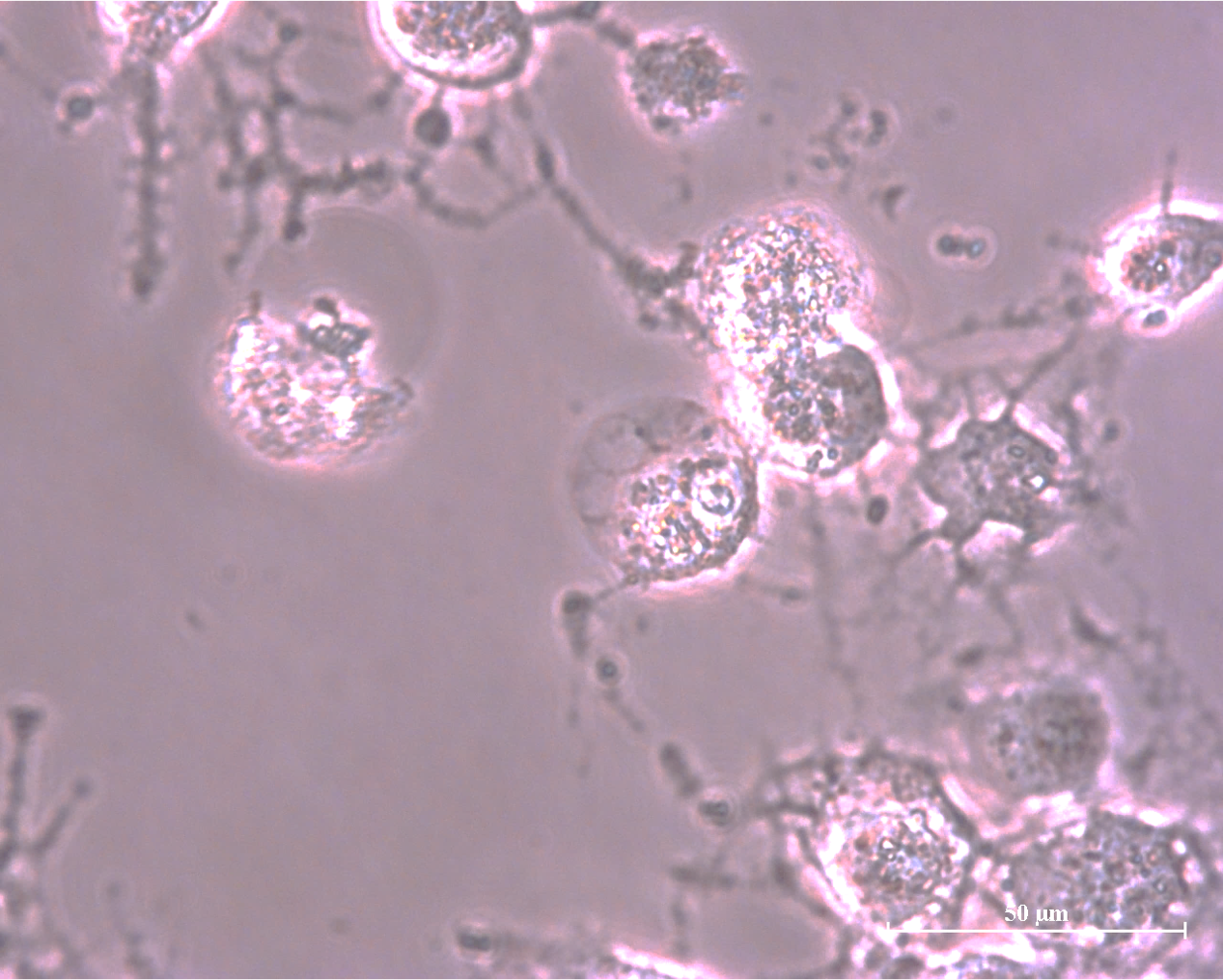

Supplement: Figure 2—source data 1. [file elife-73792-fig2-data1.zip › Figure 2-source data 1/Fig 2A/huh7 zikv.tif]

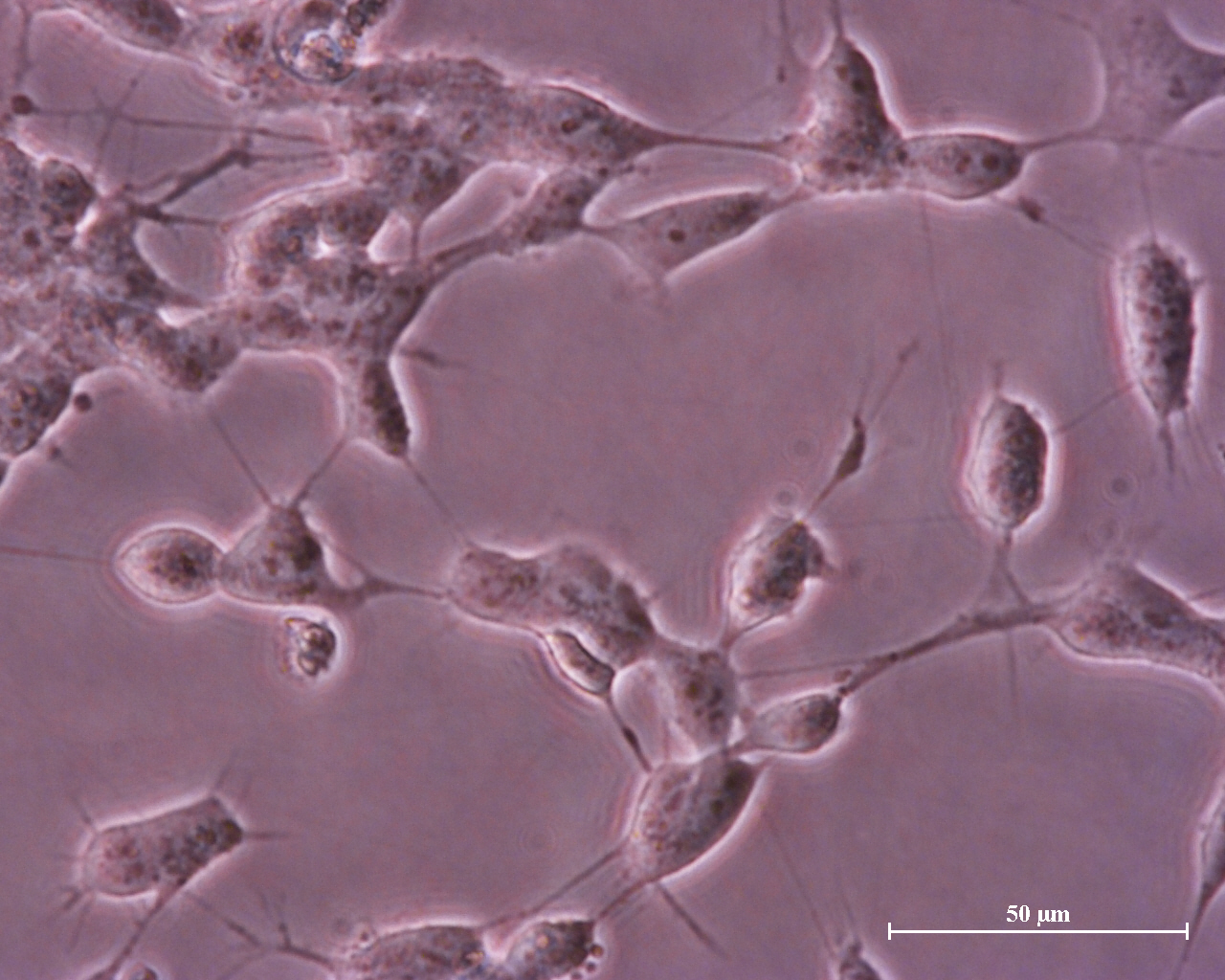

Supplement: Figure 2—source data 1. [file elife-73792-fig2-data1.zip › Figure 2-source data 1/Fig 2A/shsy5y mock.tif]

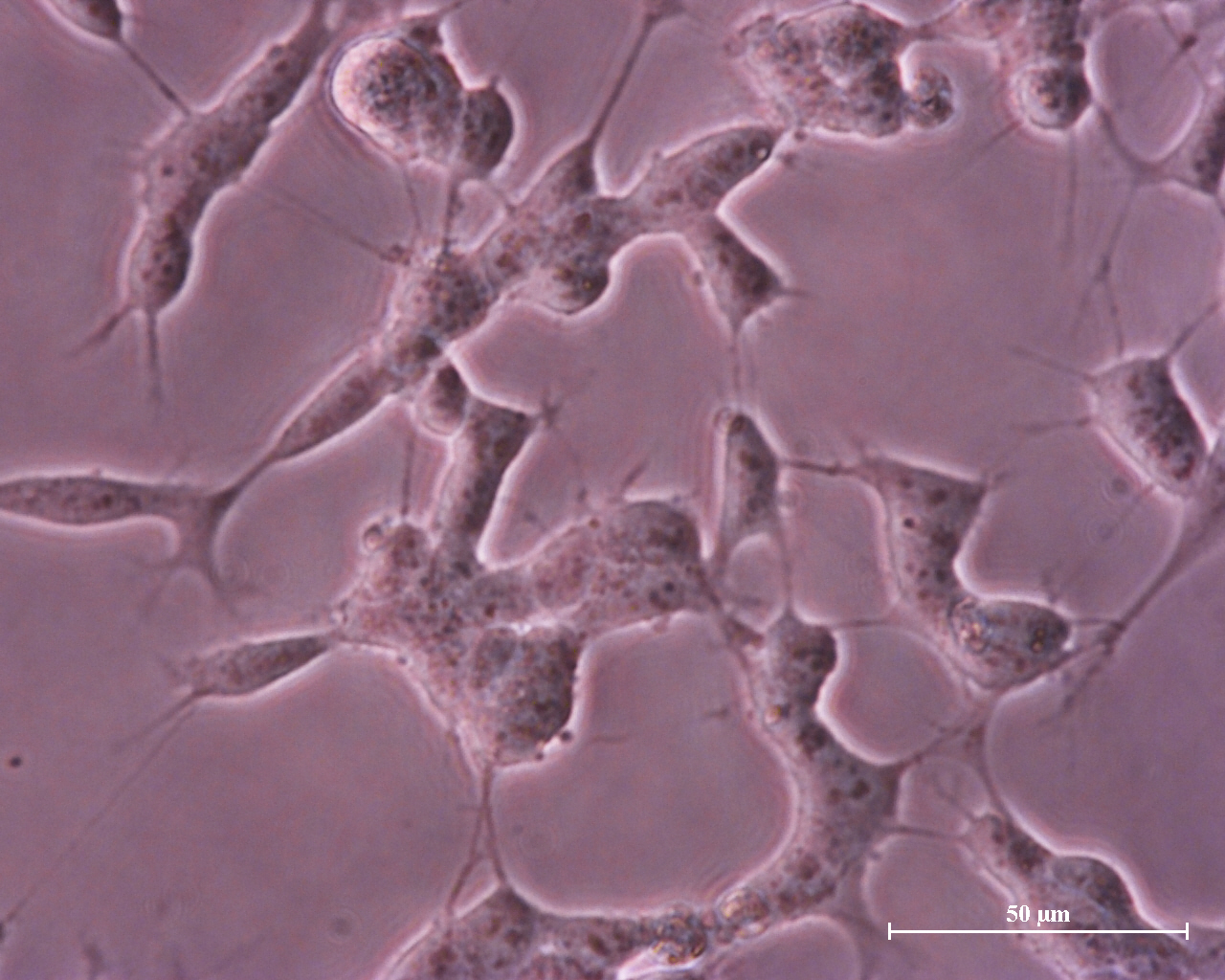

Supplement: Figure 2—source data 1. [file elife-73792-fig2-data1.zip › Figure 2-source data 1/Fig 2A/shsy5y zikv.tif]

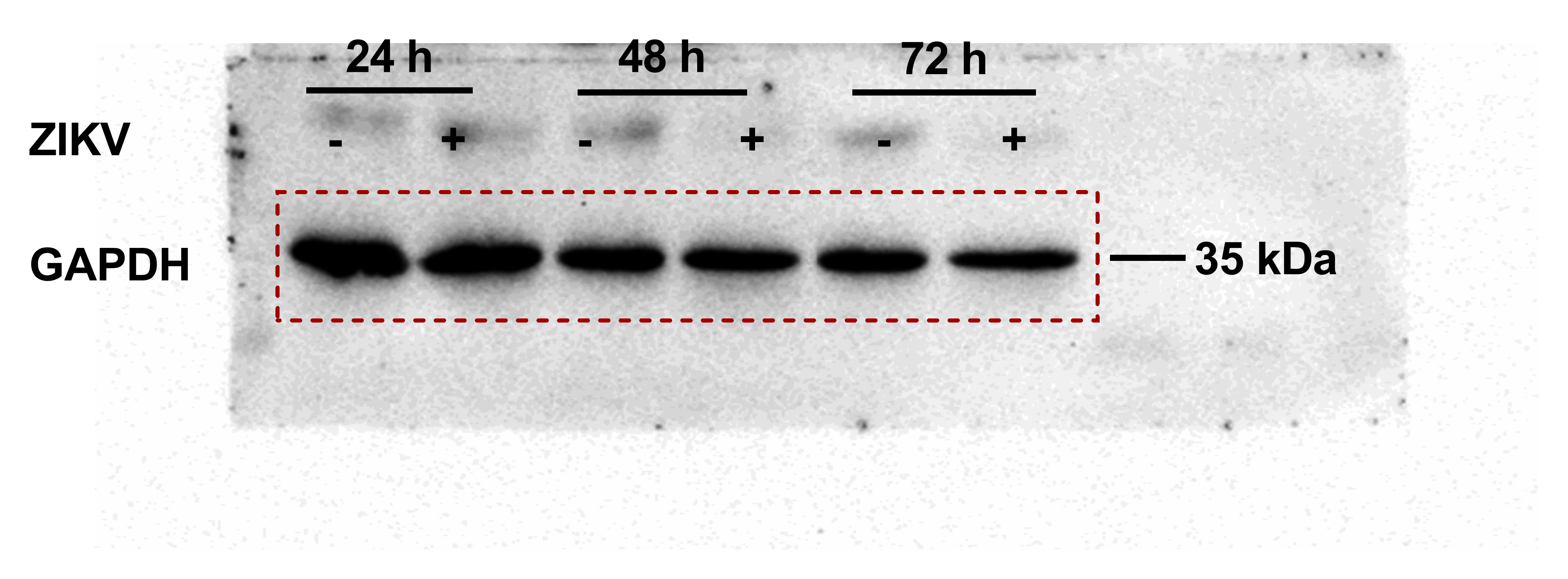

Supplement: Figure 2—source data 1. [file elife-73792-fig2-data1.zip › Figure 2-source data 1/Fig 2C/Figure 2C A549 GAPDH-labeled.tif]

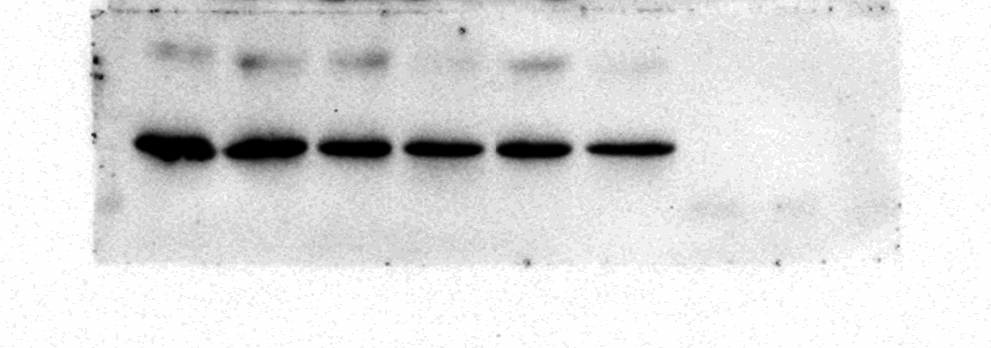

Supplement: Figure 2—source data 1. [file elife-73792-fig2-data1.zip › Figure 2-source data 1/Fig 2C/Figure 2C A549 GAPDH-raw.tif]

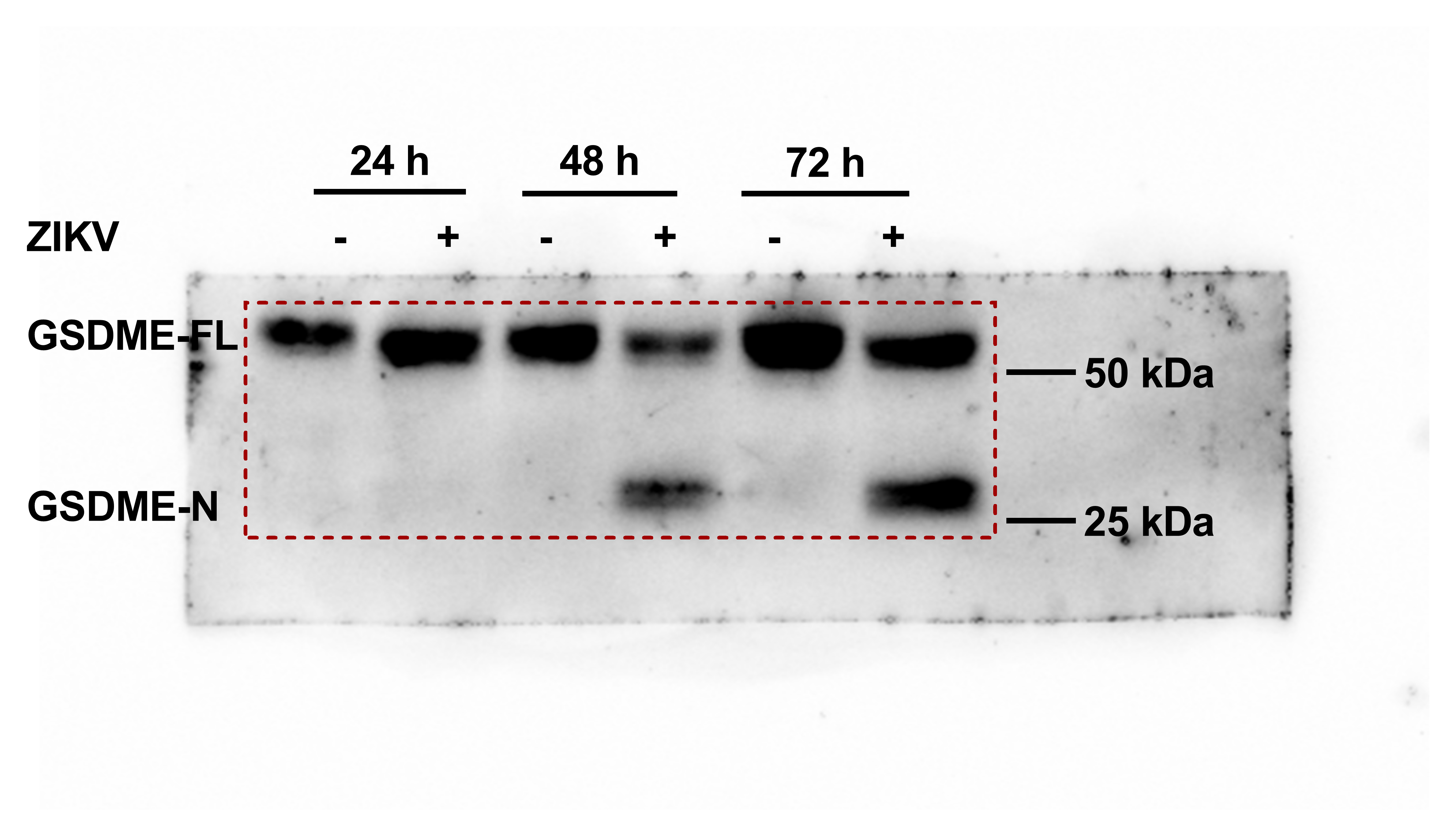

Supplement: Figure 2—source data 1. [file elife-73792-fig2-data1.zip › Figure 2-source data 1/Fig 2C/Figure 2C A549 GSDME-labeled.tif]

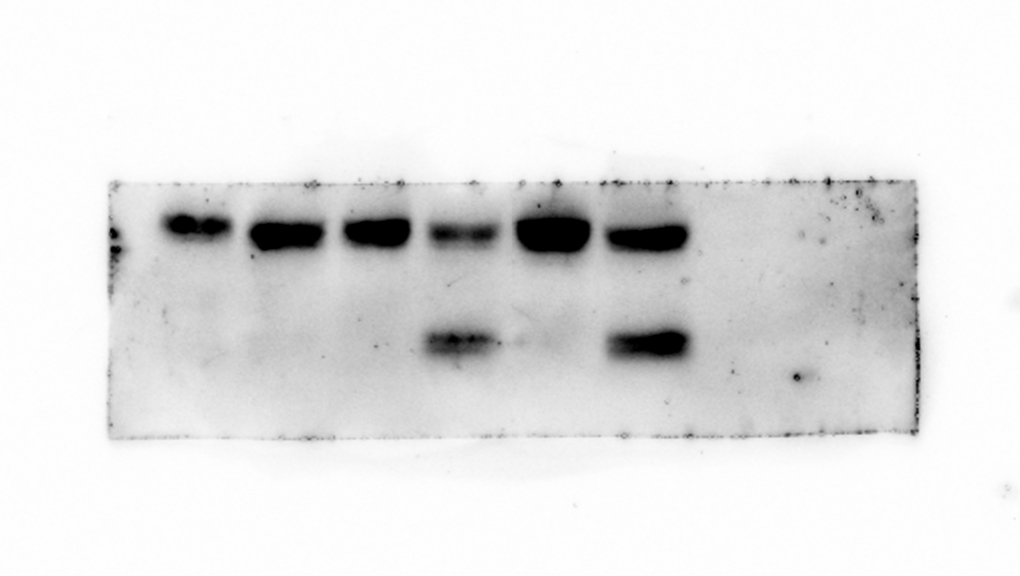

Supplement: Figure 2—source data 1. [file elife-73792-fig2-data1.zip › Figure 2-source data 1/Fig 2C/Figure 2C A549 GSDME-raw.tif]

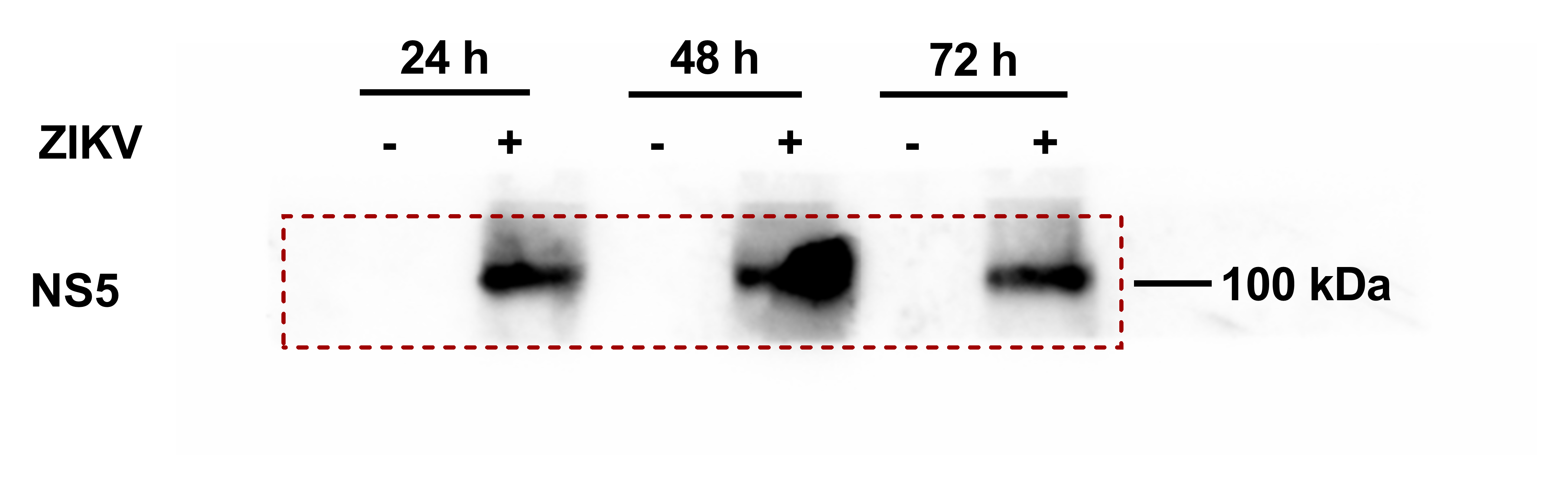

Supplement: Figure 2—source data 1. [file elife-73792-fig2-data1.zip › Figure 2-source data 1/Fig 2C/Figure 2C A549 NS5-labeled.tif]

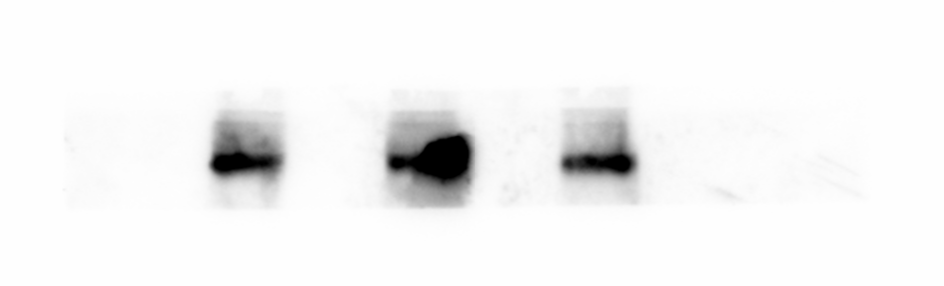

Supplement: Figure 2—source data 1. [file elife-73792-fig2-data1.zip › Figure 2-source data 1/Fig 2C/Figure 2C A549 NS5-raw.tif]

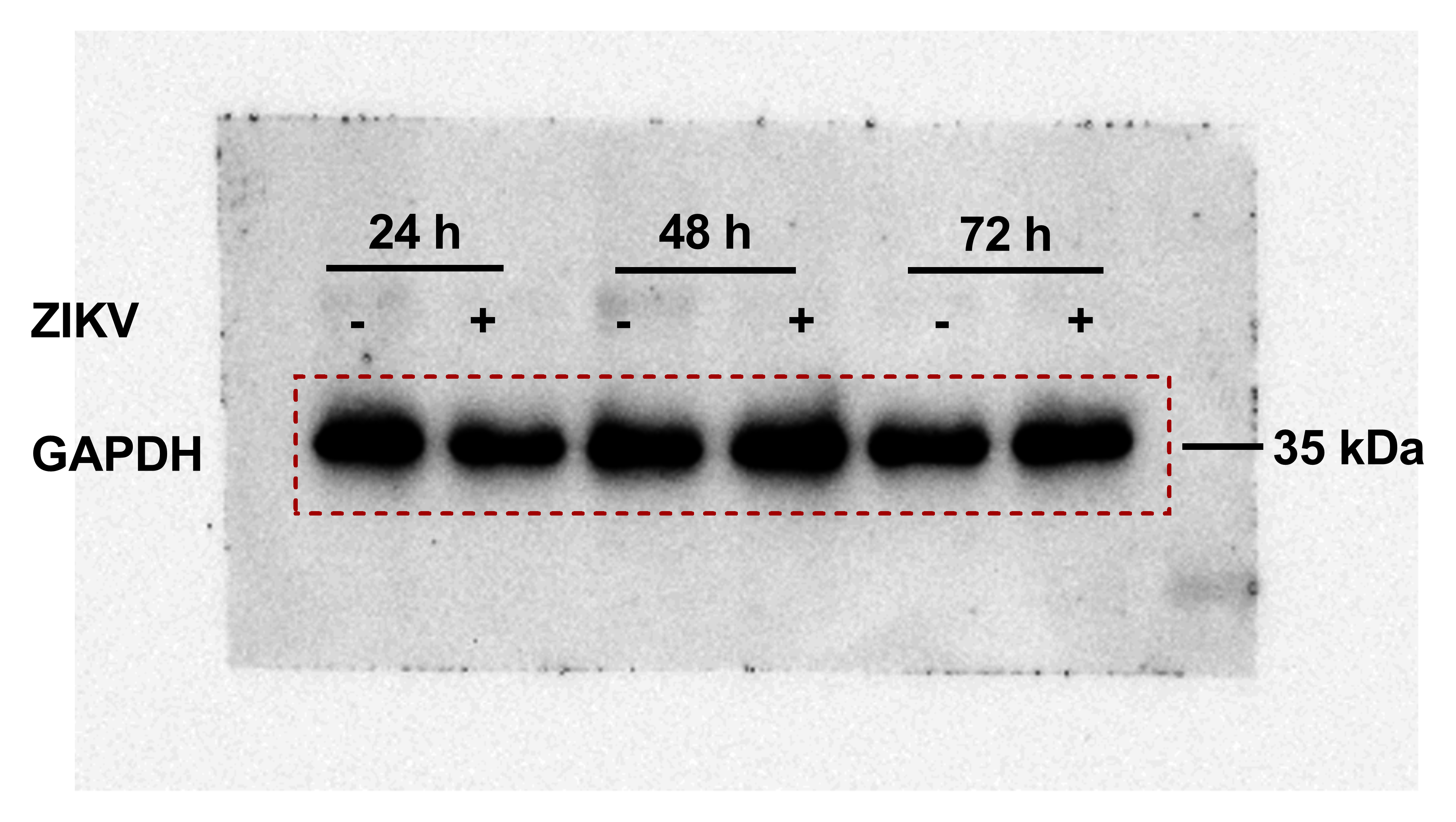

Supplement: Figure 2—source data 1. [file elife-73792-fig2-data1.zip › Figure 2-source data 1/Fig 2C/Figure 2C HEK-293T GAPDH-labeled.tif]

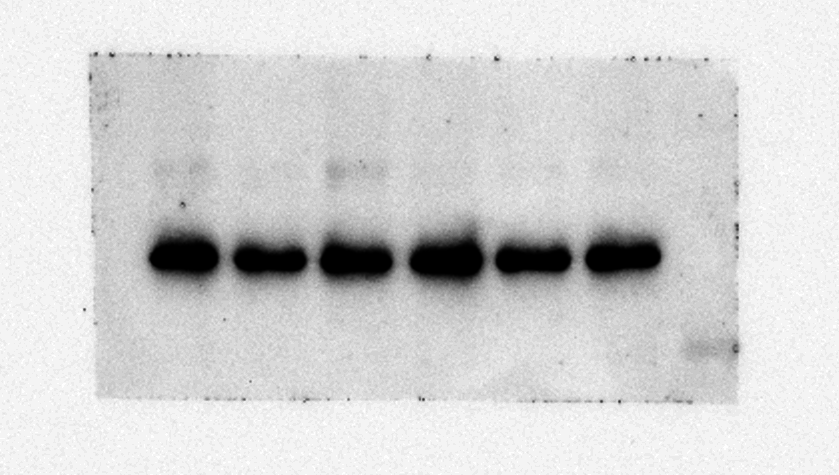

Supplement: Figure 2—source data 1. [file elife-73792-fig2-data1.zip › Figure 2-source data 1/Fig 2C/Figure 2C HEK-293T GAPDH-raw.tif]

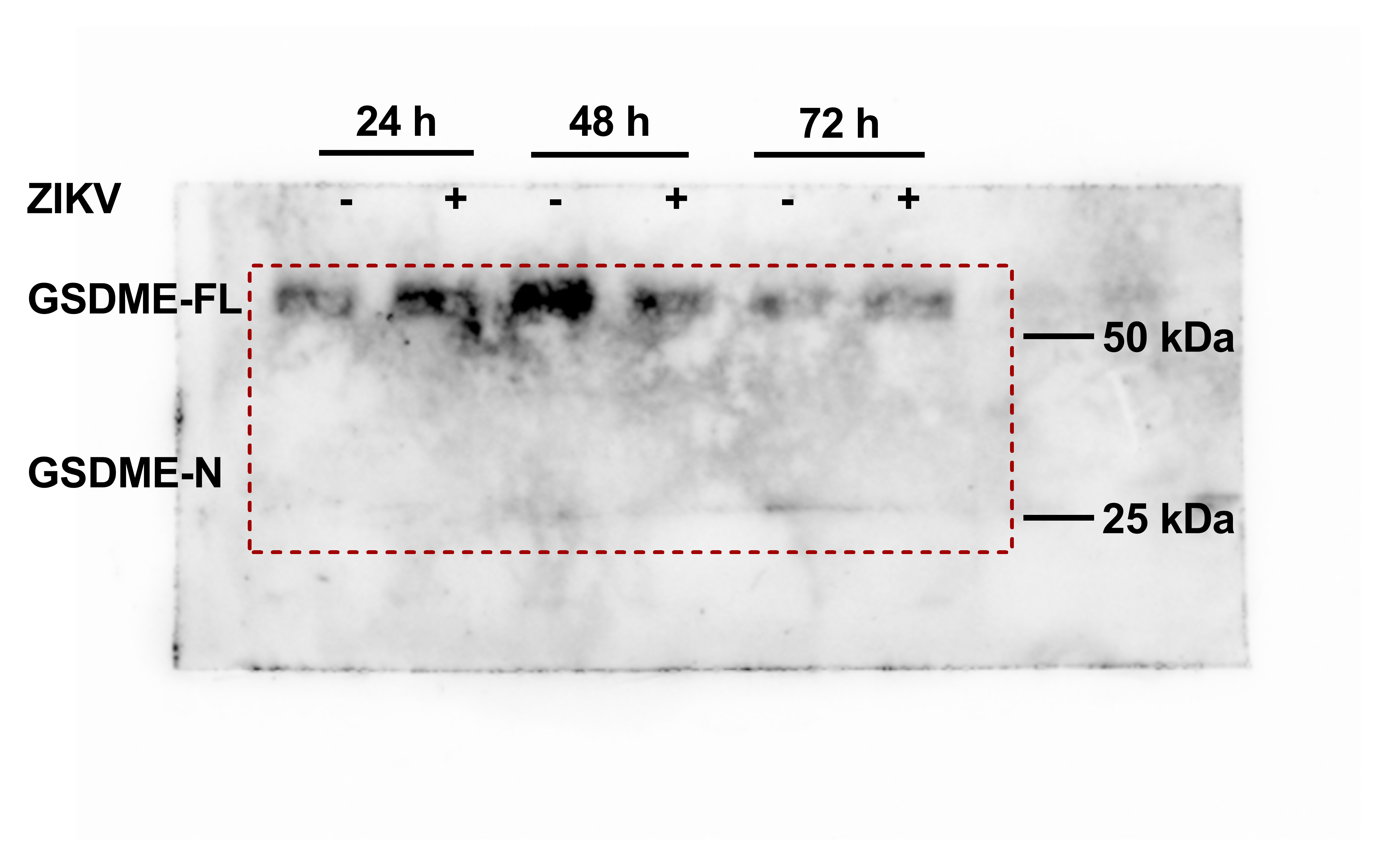

Supplement: Figure 2—source data 1. [file elife-73792-fig2-data1.zip › Figure 2-source data 1/Fig 2C/Figure 2C HEK-293T GSDME-labeled.tif]

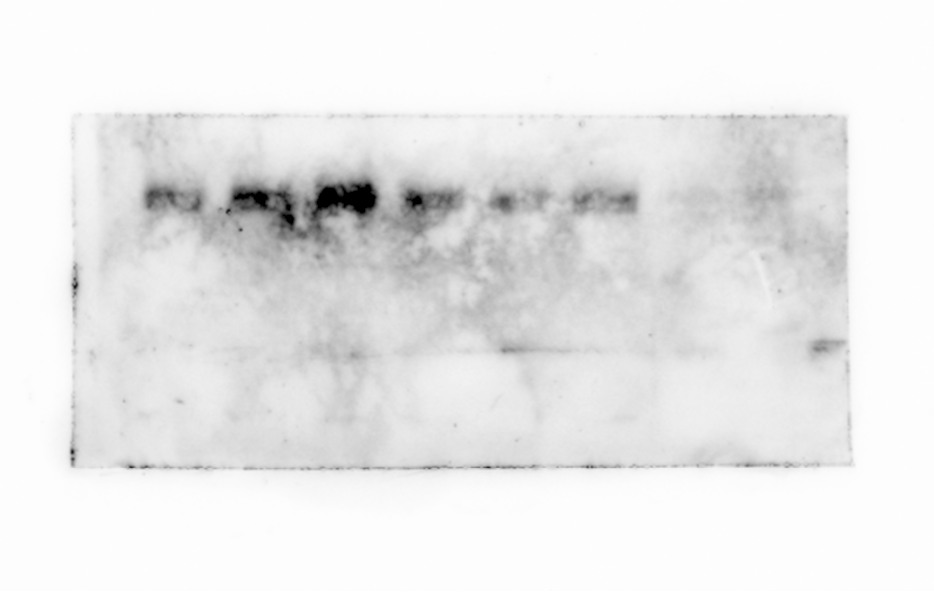

Supplement: Figure 2—source data 1. [file elife-73792-fig2-data1.zip › Figure 2-source data 1/Fig 2C/Figure 2C HEK-293T GSDME-raw.tif]

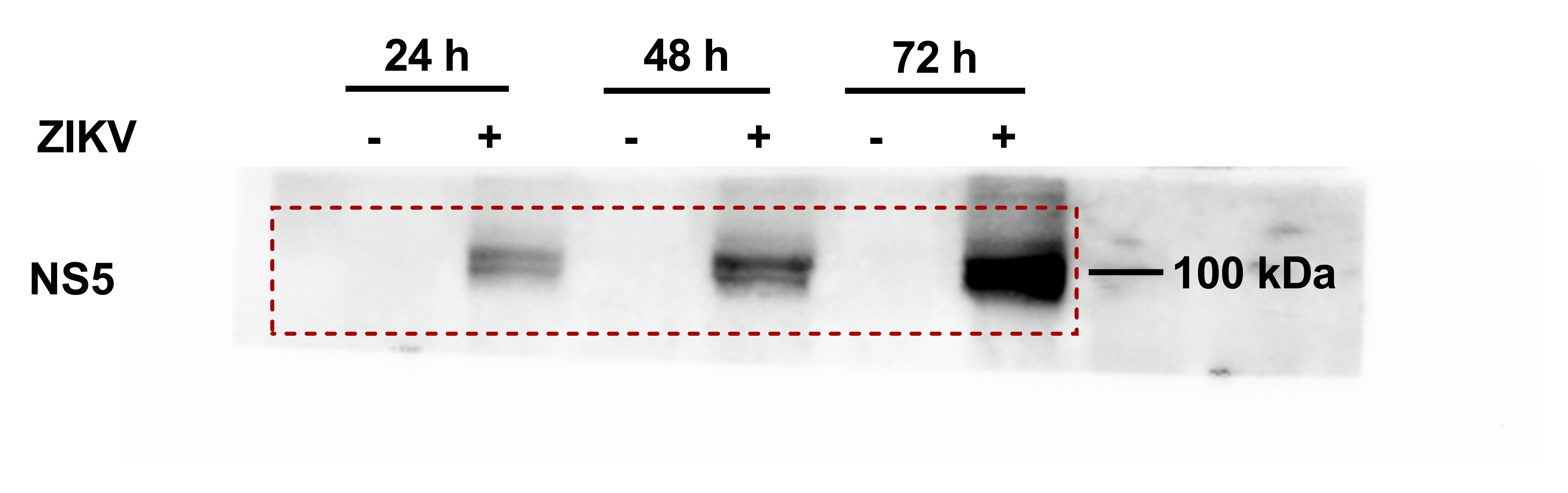

Supplement: Figure 2—source data 1. [file elife-73792-fig2-data1.zip › Figure 2-source data 1/Fig 2C/Figure 2C HEK-293T NS5-labeled.tif]

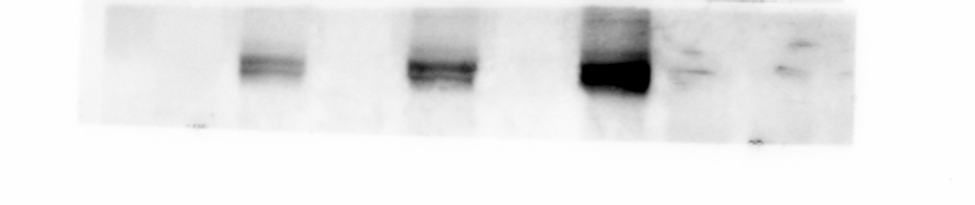

Supplement: Figure 2—source data 1. [file elife-73792-fig2-data1.zip › Figure 2-source data 1/Fig 2C/Figure 2C HEK-293T NS5-raw.tif]

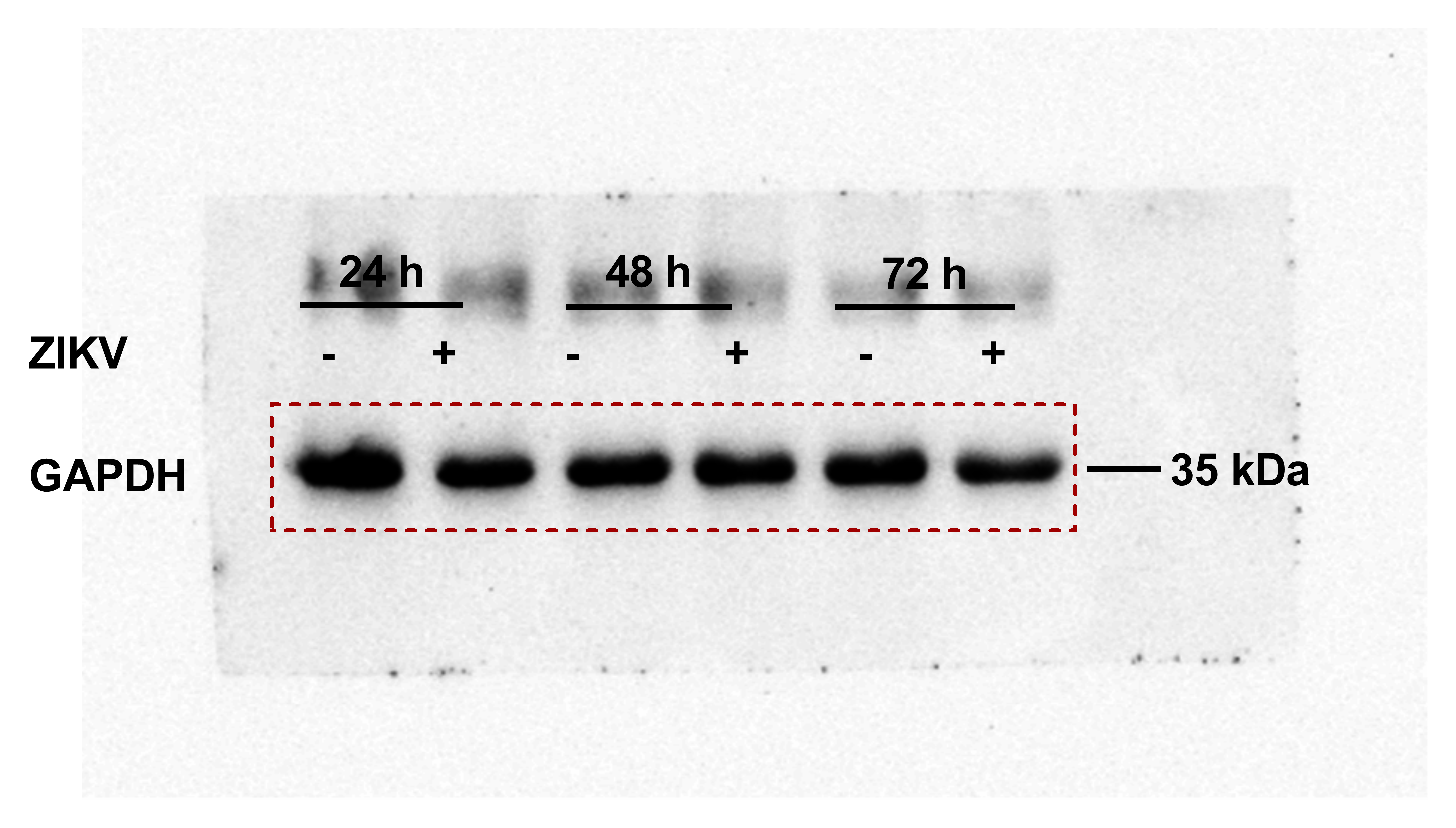

Supplement: Figure 2—source data 1. [file elife-73792-fig2-data1.zip › Figure 2-source data 1/Fig 2C/Figure 2C HeLa GAPDH-labeled.tif]

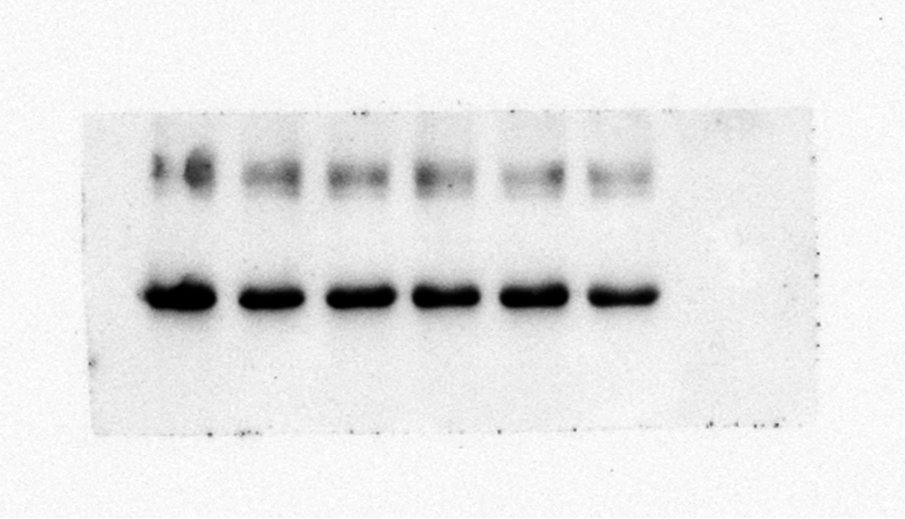

Supplement: Figure 2—source data 1. [file elife-73792-fig2-data1.zip › Figure 2-source data 1/Fig 2C/Figure 2C HeLa GAPDH-raw.tif]

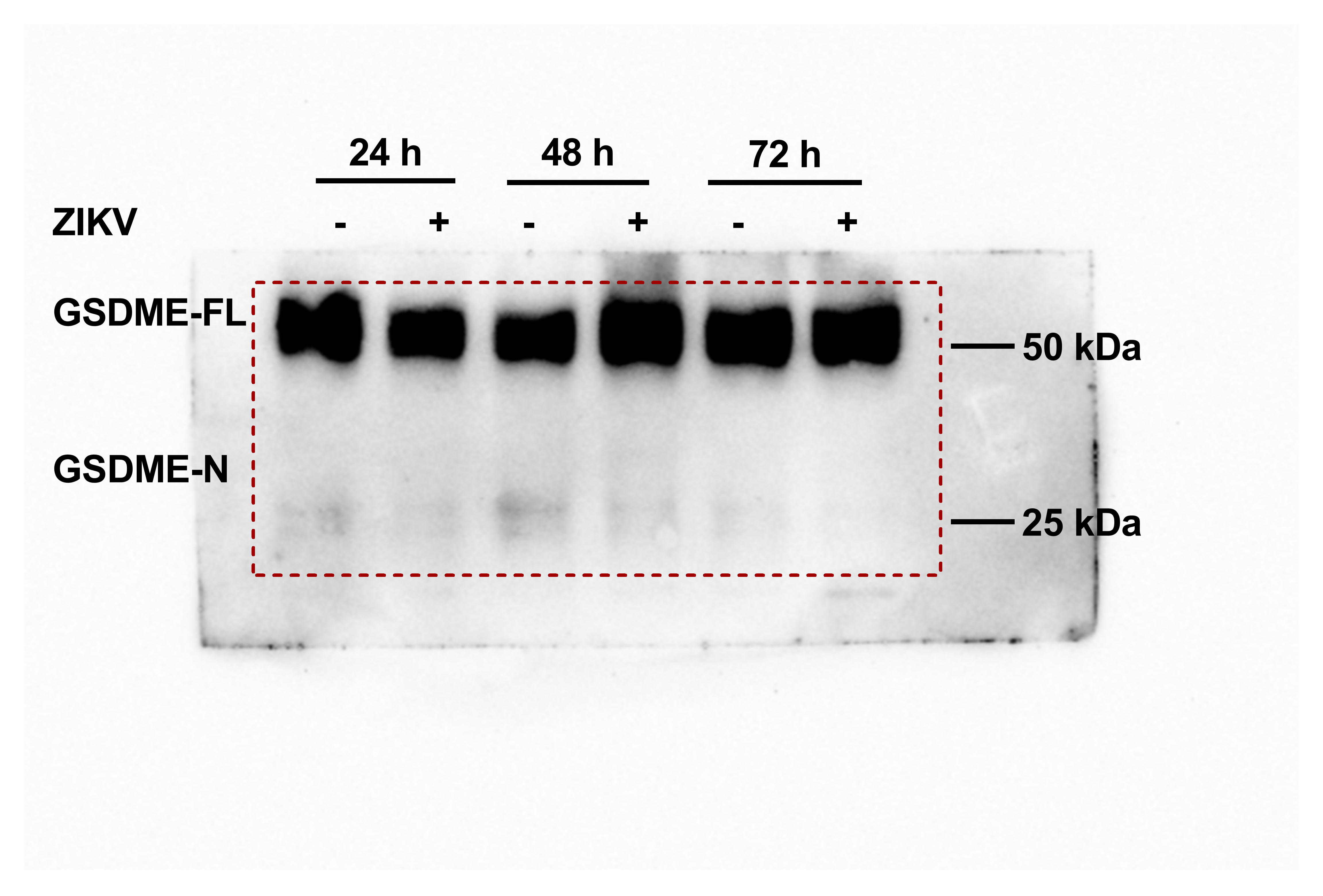

Supplement: Figure 2—source data 1. [file elife-73792-fig2-data1.zip › Figure 2-source data 1/Fig 2C/Figure 2C HeLa GSDME-labeled.tif]

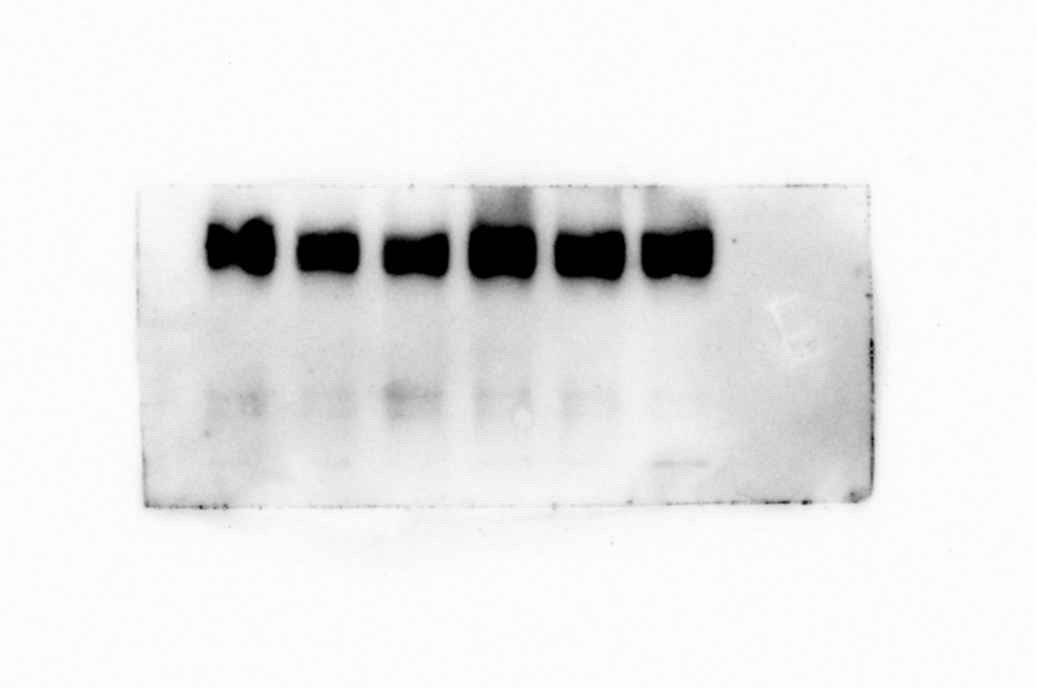

Supplement: Figure 2—source data 1. [file elife-73792-fig2-data1.zip › Figure 2-source data 1/Fig 2C/Figure 2C HeLa GSDME-raw.tif]

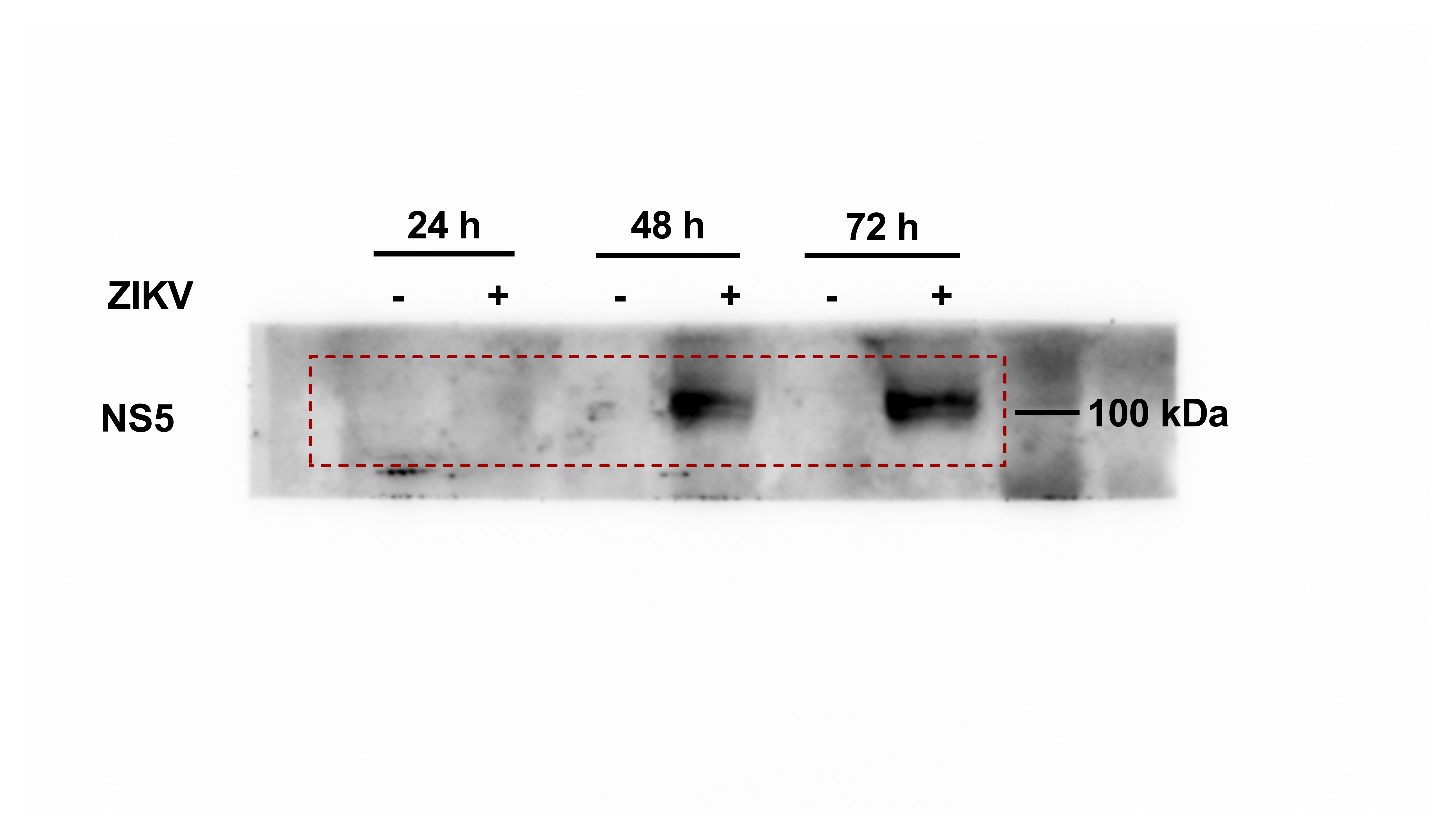

Supplement: Figure 2—source data 1. [file elife-73792-fig2-data1.zip › Figure 2-source data 1/Fig 2C/Figure 2C HeLa NS5-labeled.tif]

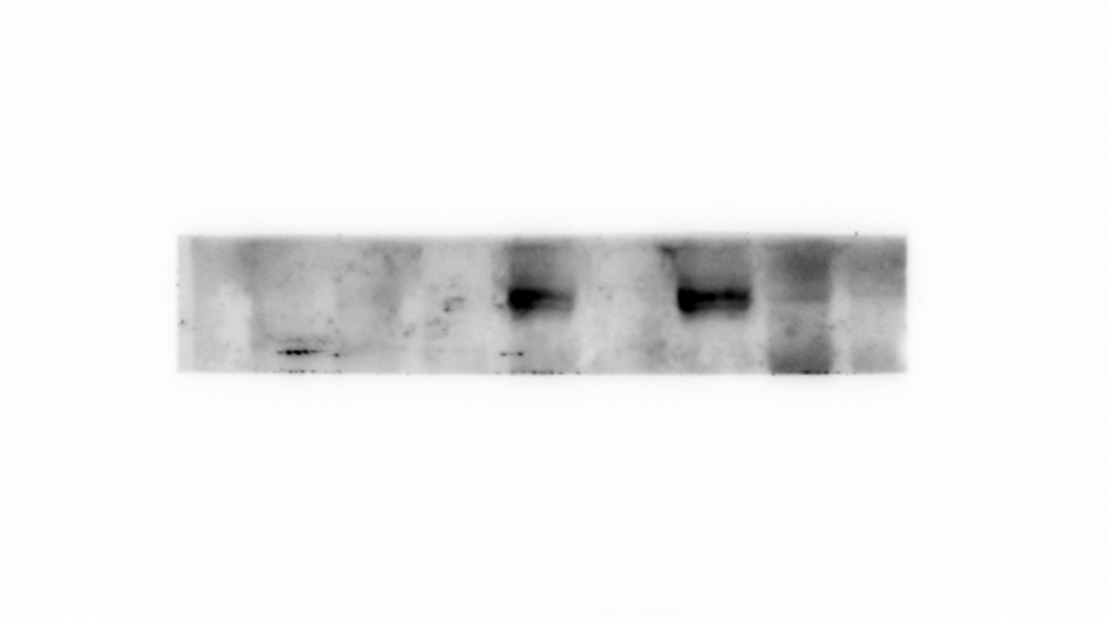

Supplement: Figure 2—source data 1. [file elife-73792-fig2-data1.zip › Figure 2-source data 1/Fig 2C/Figure 2C HeLa NS5-raw.tif]

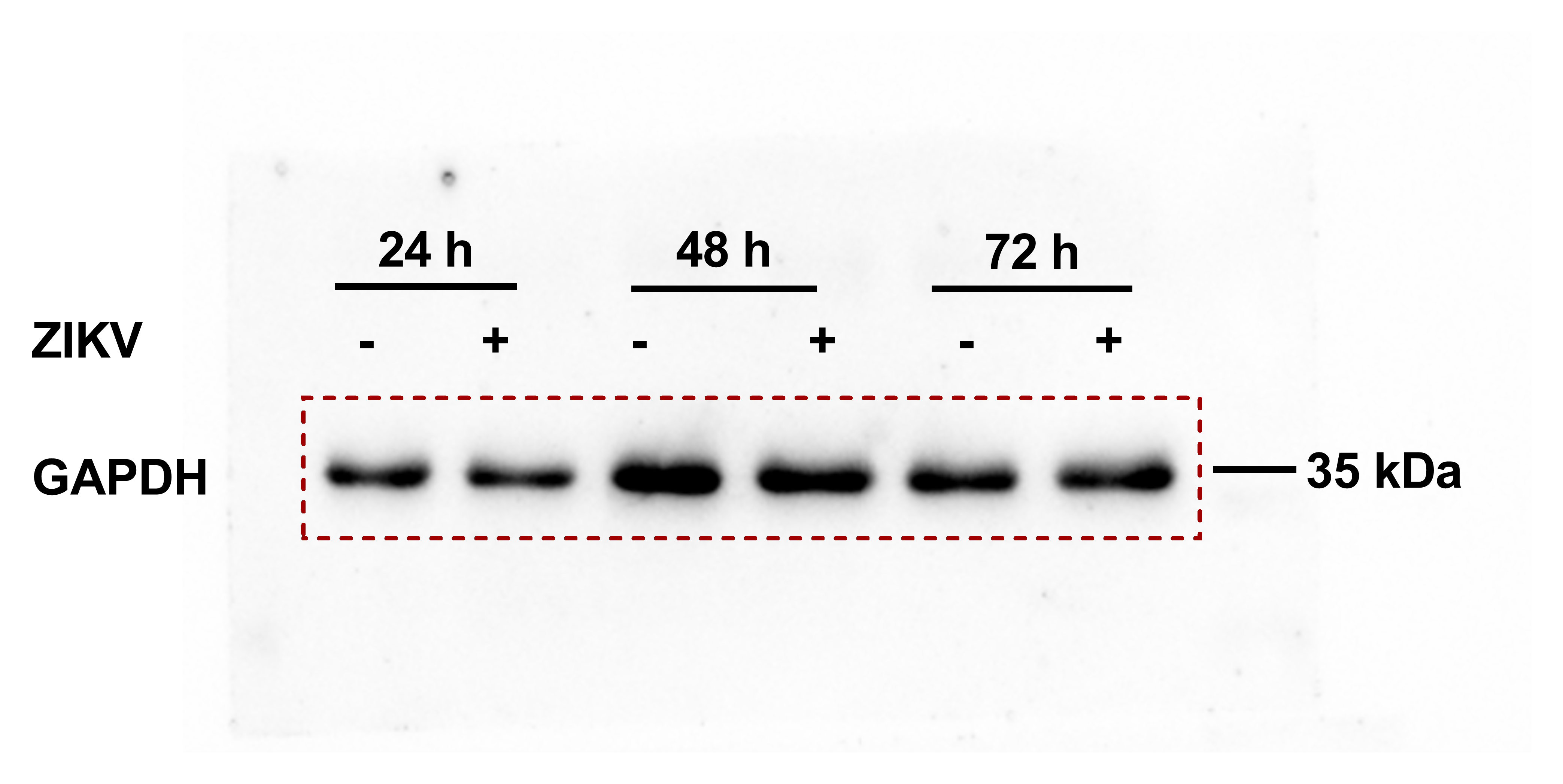

Supplement: Figure 2—source data 1. [file elife-73792-fig2-data1.zip › Figure 2-source data 1/Fig 2C/Figure 2C Huh-7 GAPDH-labeled.tif]

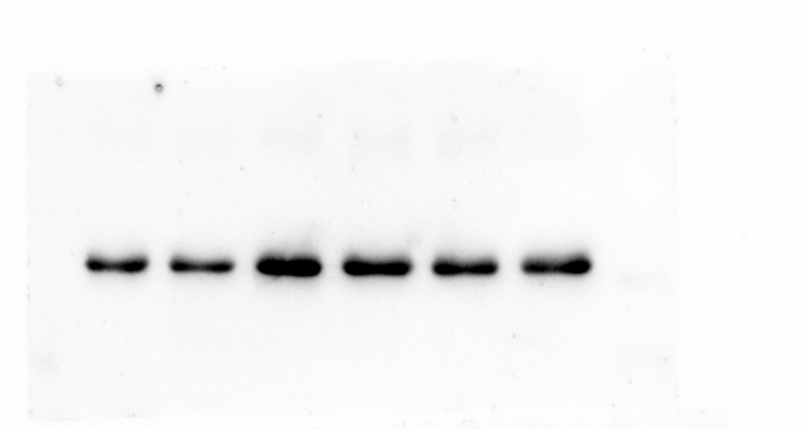

Supplement: Figure 2—source data 1. [file elife-73792-fig2-data1.zip › Figure 2-source data 1/Fig 2C/Figure 2C Huh-7 GAPDH-raw.tif]

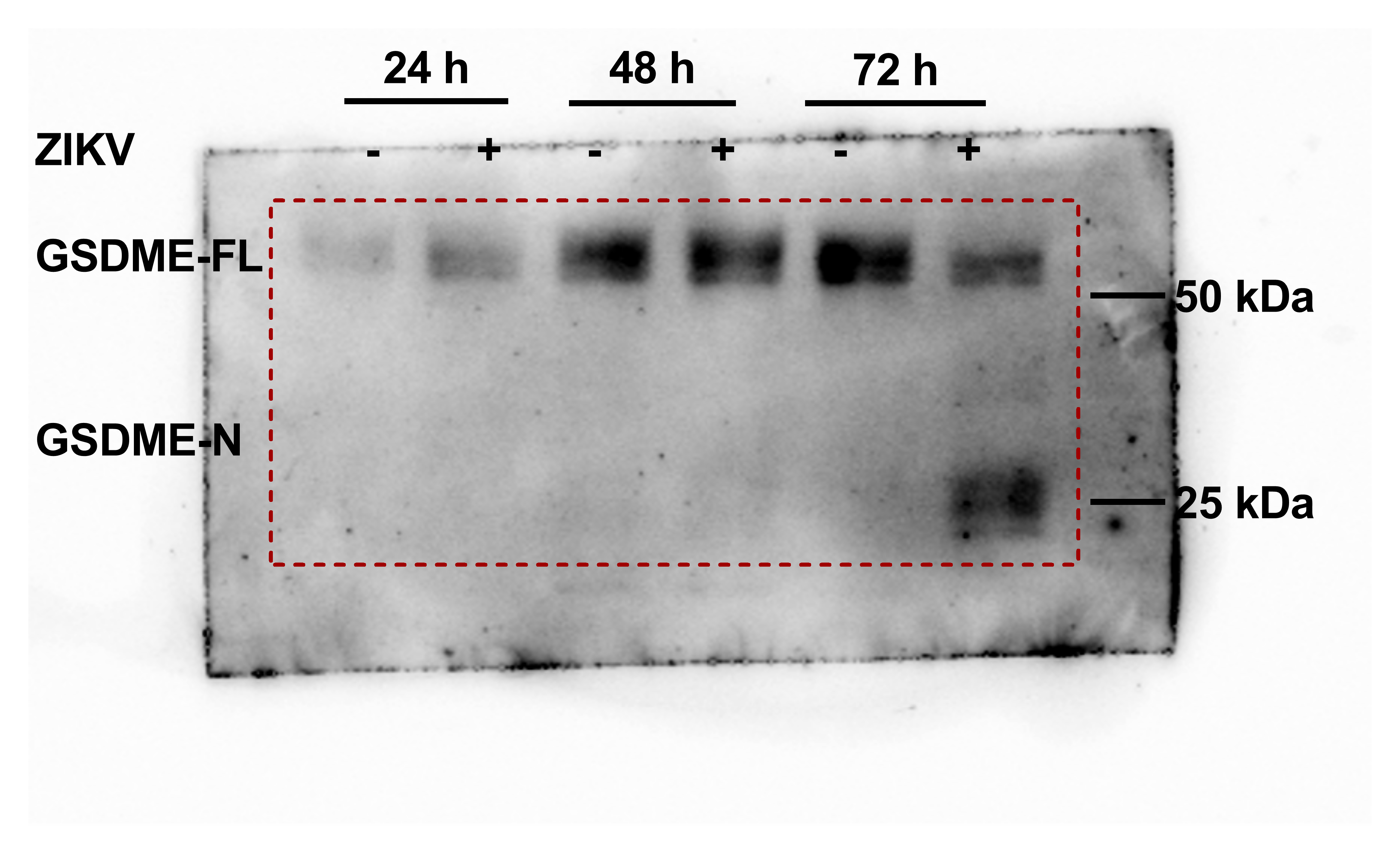

Supplement: Figure 2—source data 1. [file elife-73792-fig2-data1.zip › Figure 2-source data 1/Fig 2C/Figure 2C hUH-7 GSDME-labeled.tif]

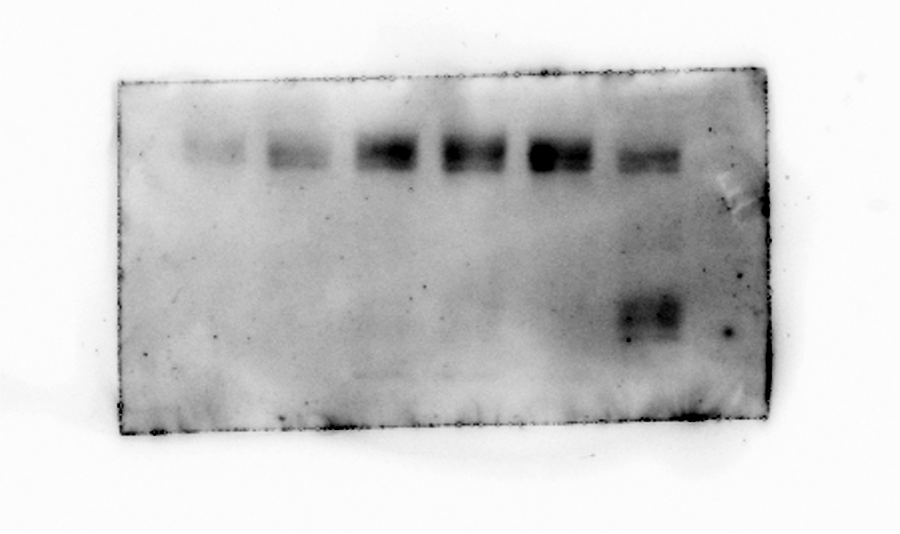

Supplement: Figure 2—source data 1. [file elife-73792-fig2-data1.zip › Figure 2-source data 1/Fig 2C/Figure 2C hUH-7 GSDME-raw.tif]

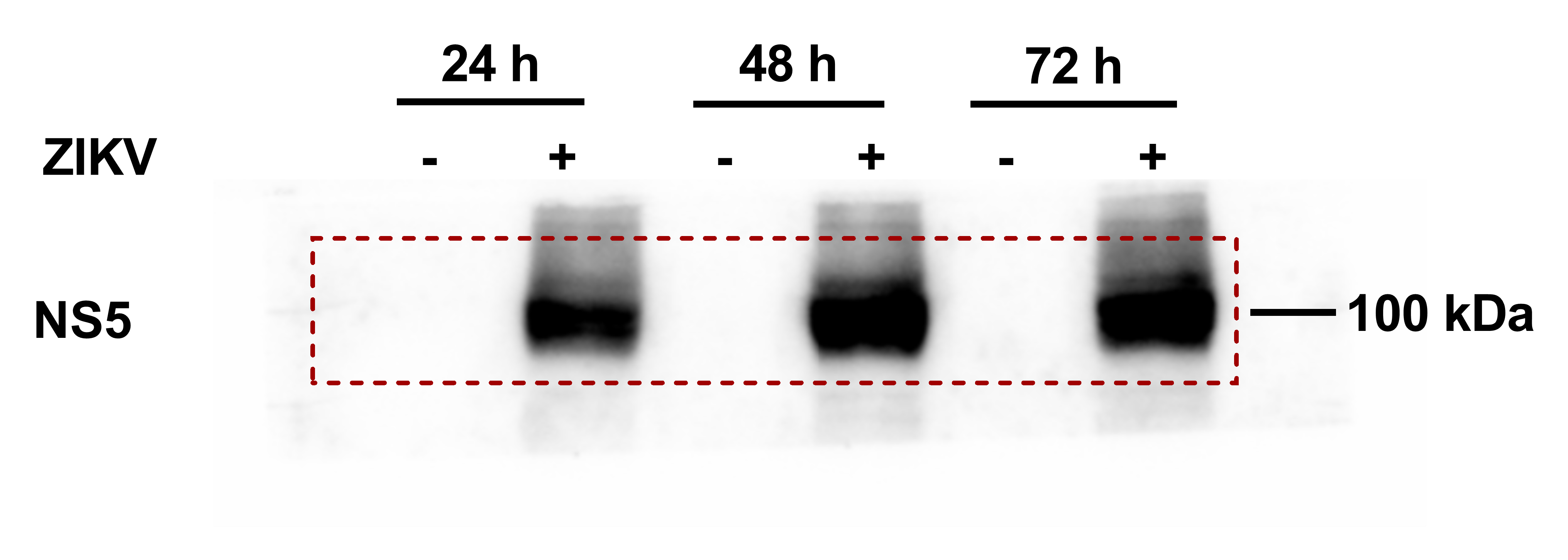

Supplement: Figure 2—source data 1. [file elife-73792-fig2-data1.zip › Figure 2-source data 1/Fig 2C/Figure 2C Huh-7 NS5-labeled.tif]

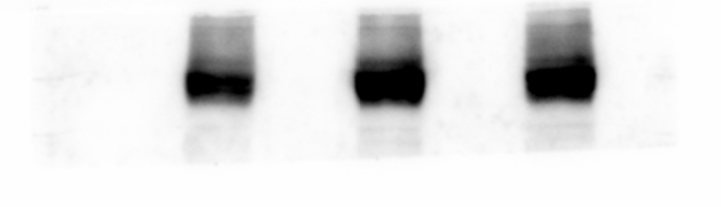

Supplement: Figure 2—source data 1. [file elife-73792-fig2-data1.zip › Figure 2-source data 1/Fig 2C/Figure 2C Huh-7 NS5-raw.tif]

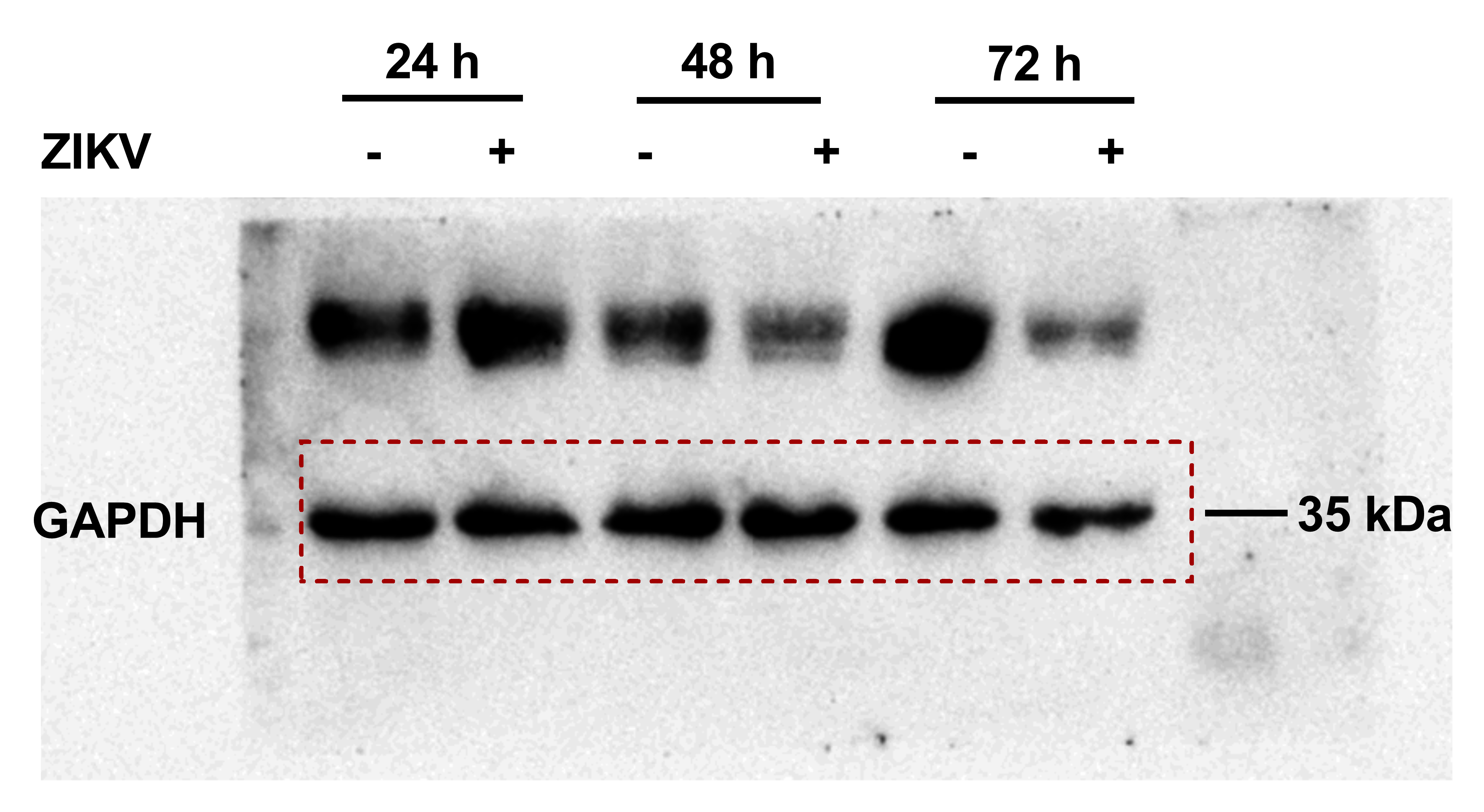

Supplement: Figure 2—source data 1. [file elife-73792-fig2-data1.zip › Figure 2-source data 1/Fig 2C/Figure 2C SH-SY5Y GAPDH-labeled.tif]

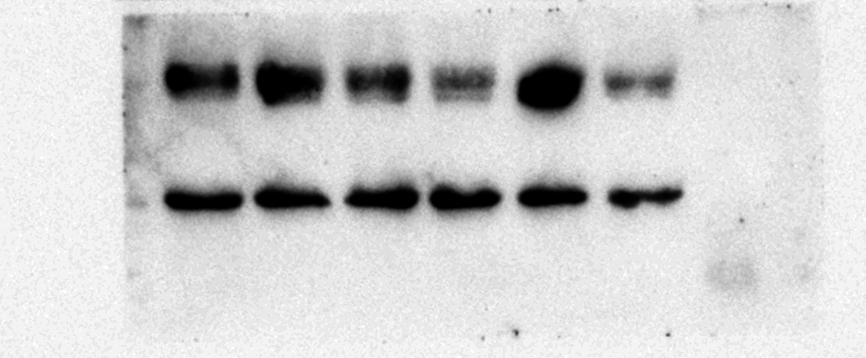

Supplement: Figure 2—source data 1. [file elife-73792-fig2-data1.zip › Figure 2-source data 1/Fig 2C/Figure 2C SH-SY5Y GAPDH-raw.tif]

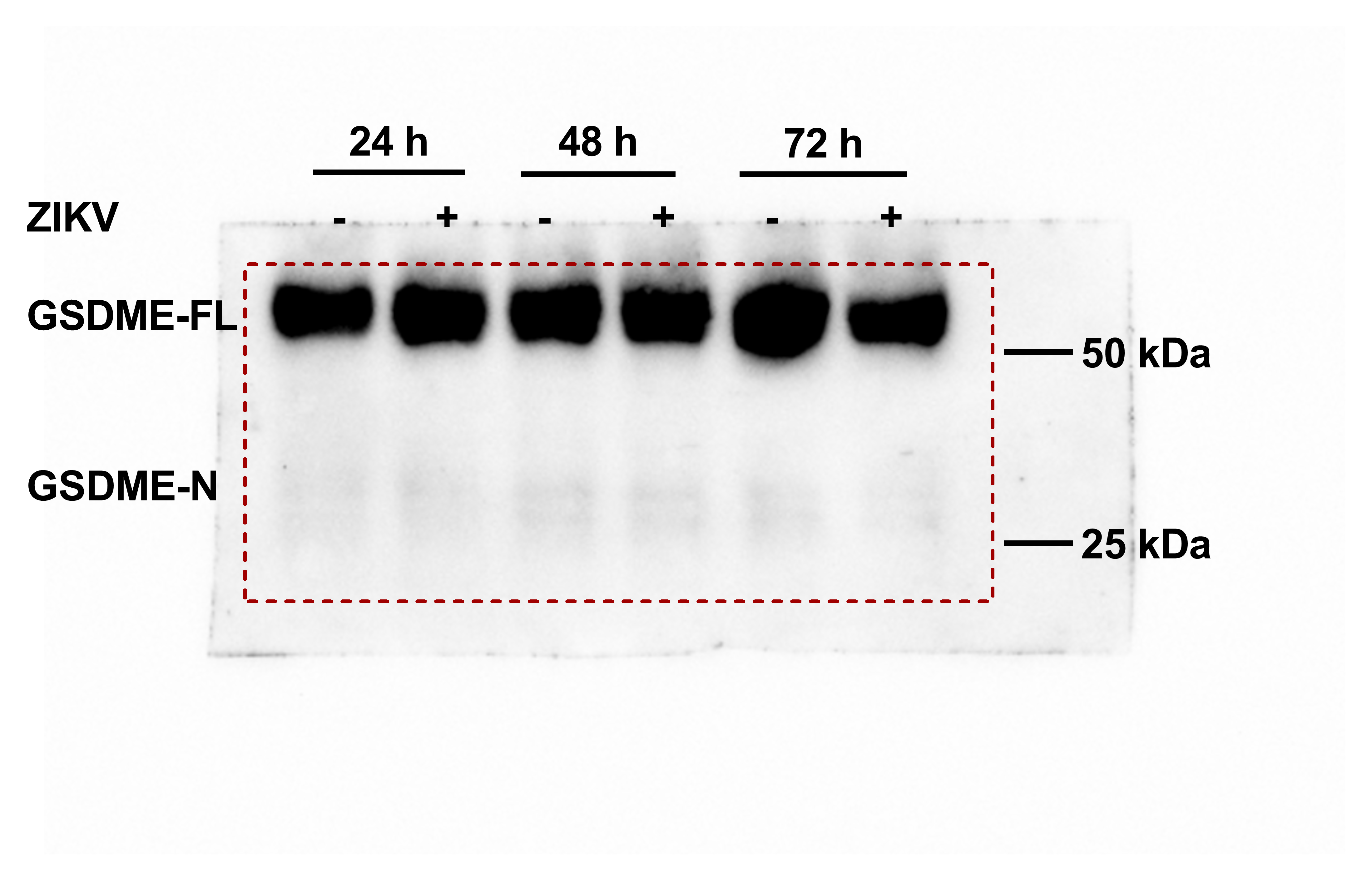

Supplement: Figure 2—source data 1. [file elife-73792-fig2-data1.zip › Figure 2-source data 1/Fig 2C/Figure 2C SH-SY5Y GSDME-labeled.tif]

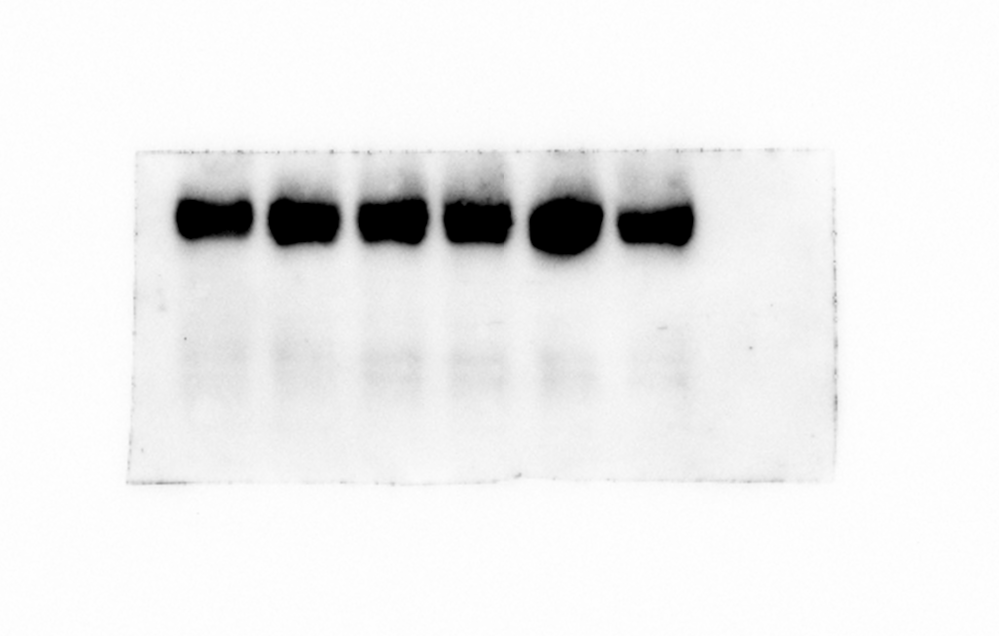

Supplement: Figure 2—source data 1. [file elife-73792-fig2-data1.zip › Figure 2-source data 1/Fig 2C/Figure 2C SH-SY5Y GSDME-raw.tif]

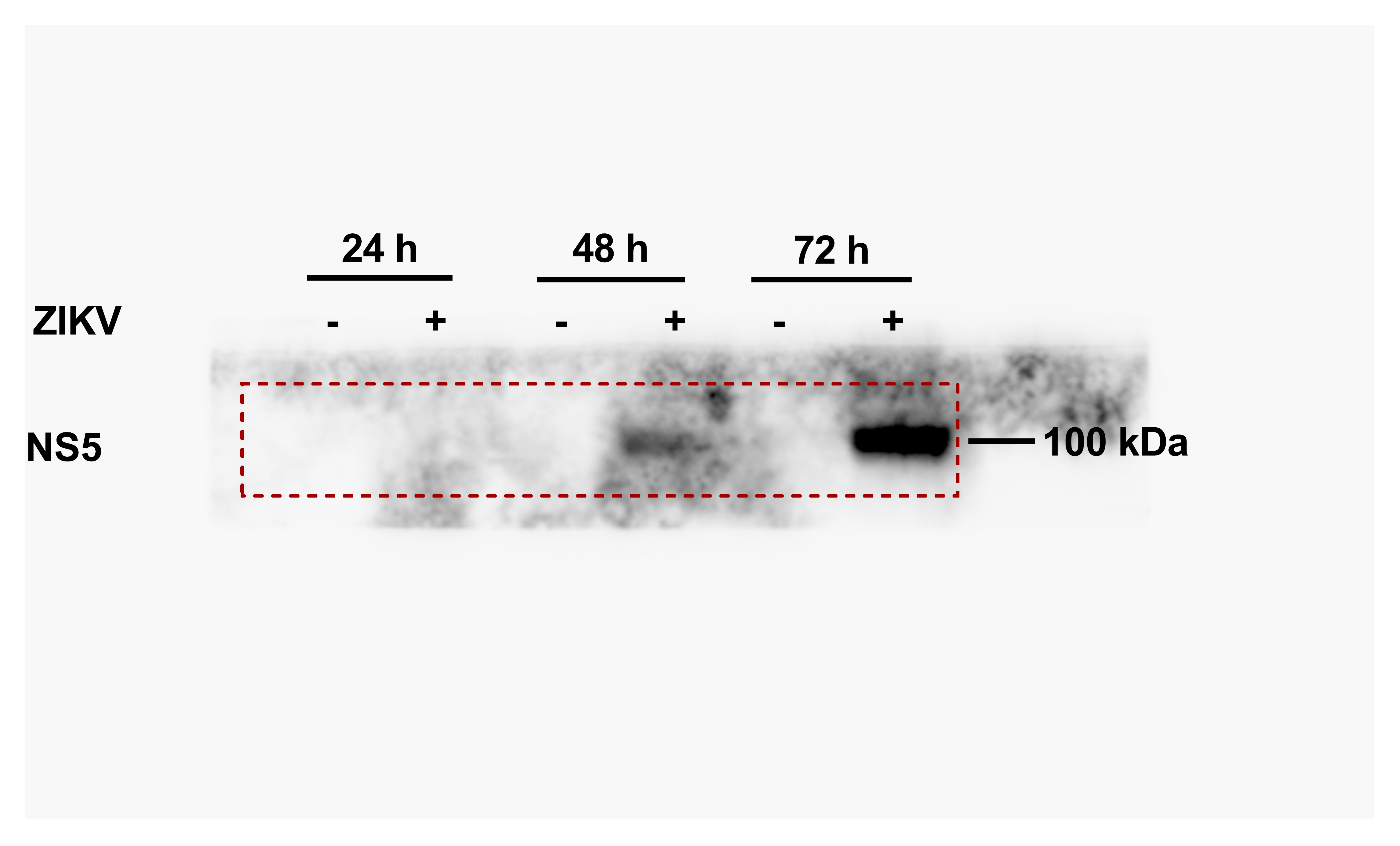

Supplement: Figure 2—source data 1. [file elife-73792-fig2-data1.zip › Figure 2-source data 1/Fig 2C/Figure 2C SH-SY5Y NS5-labeled.tif]

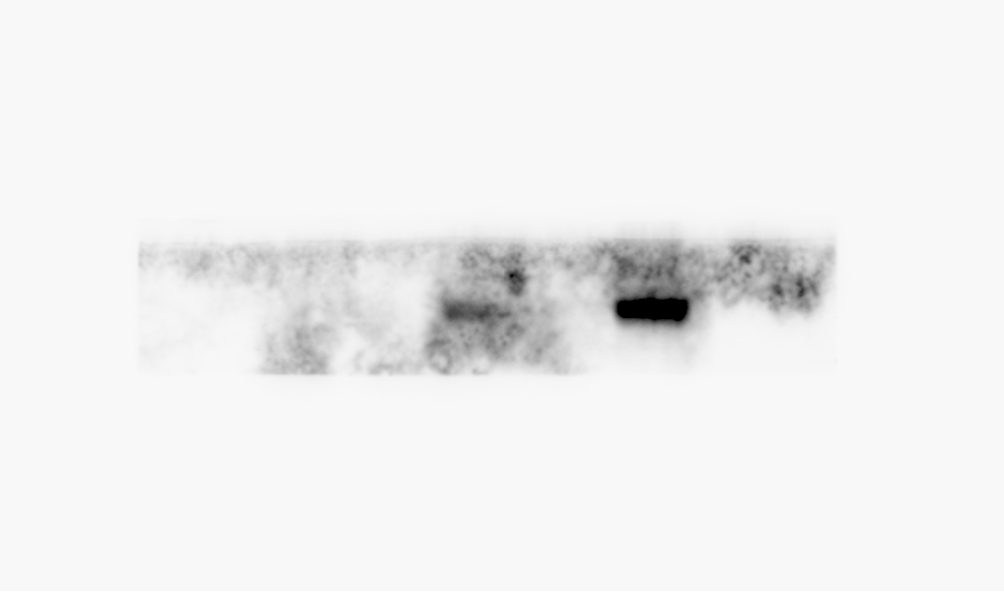

Supplement: Figure 2—source data 1. [file elife-73792-fig2-data1.zip › Figure 2-source data 1/Fig 2C/Figure 2C SH-SY5Y NS5-raw.tif]

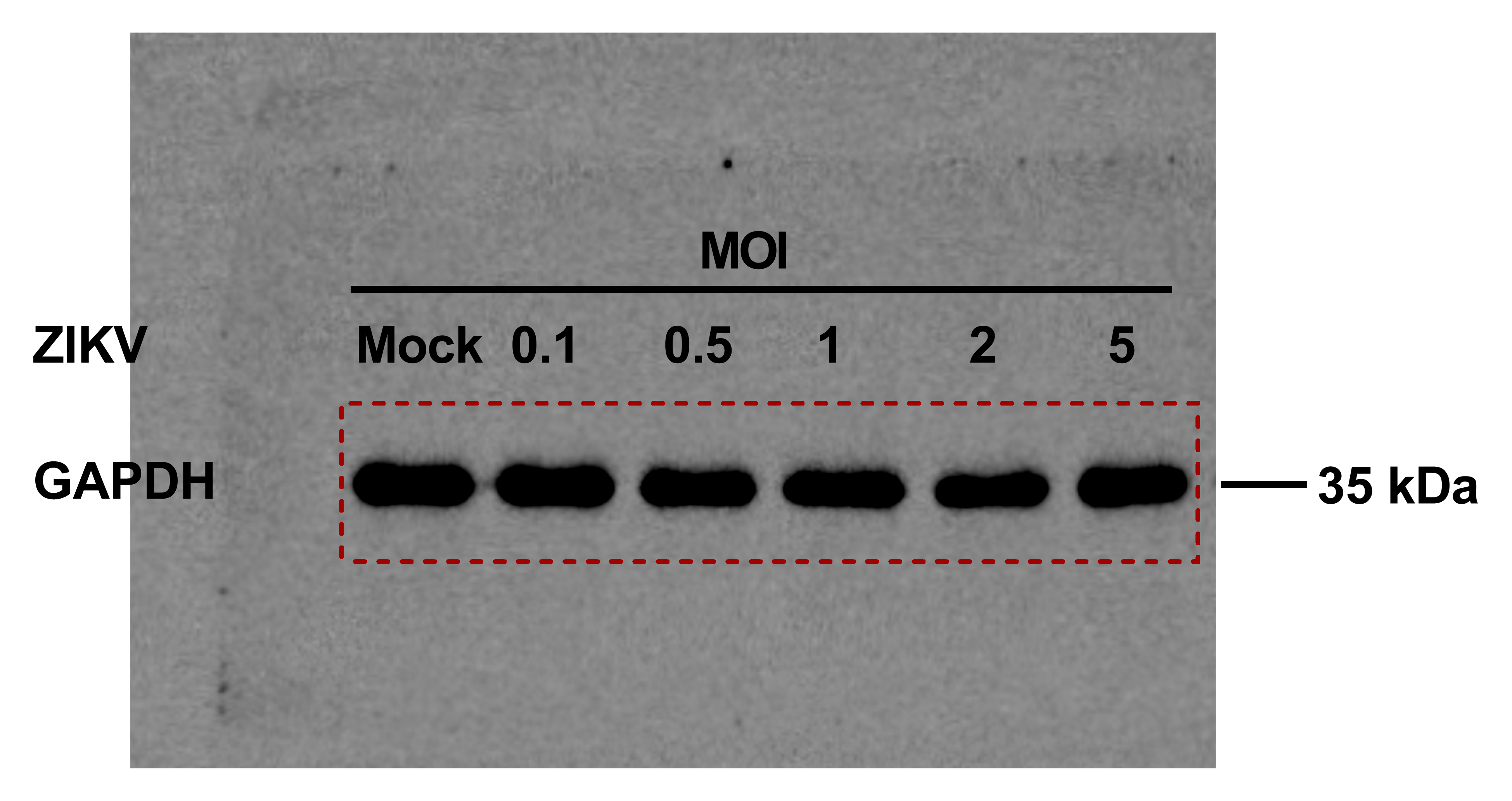

Supplement: Figure 2—source data 1. [file elife-73792-fig2-data1.zip › Figure 2-source data 1/Fig 2F/Figure 2F GAPDH-labeled.tif]

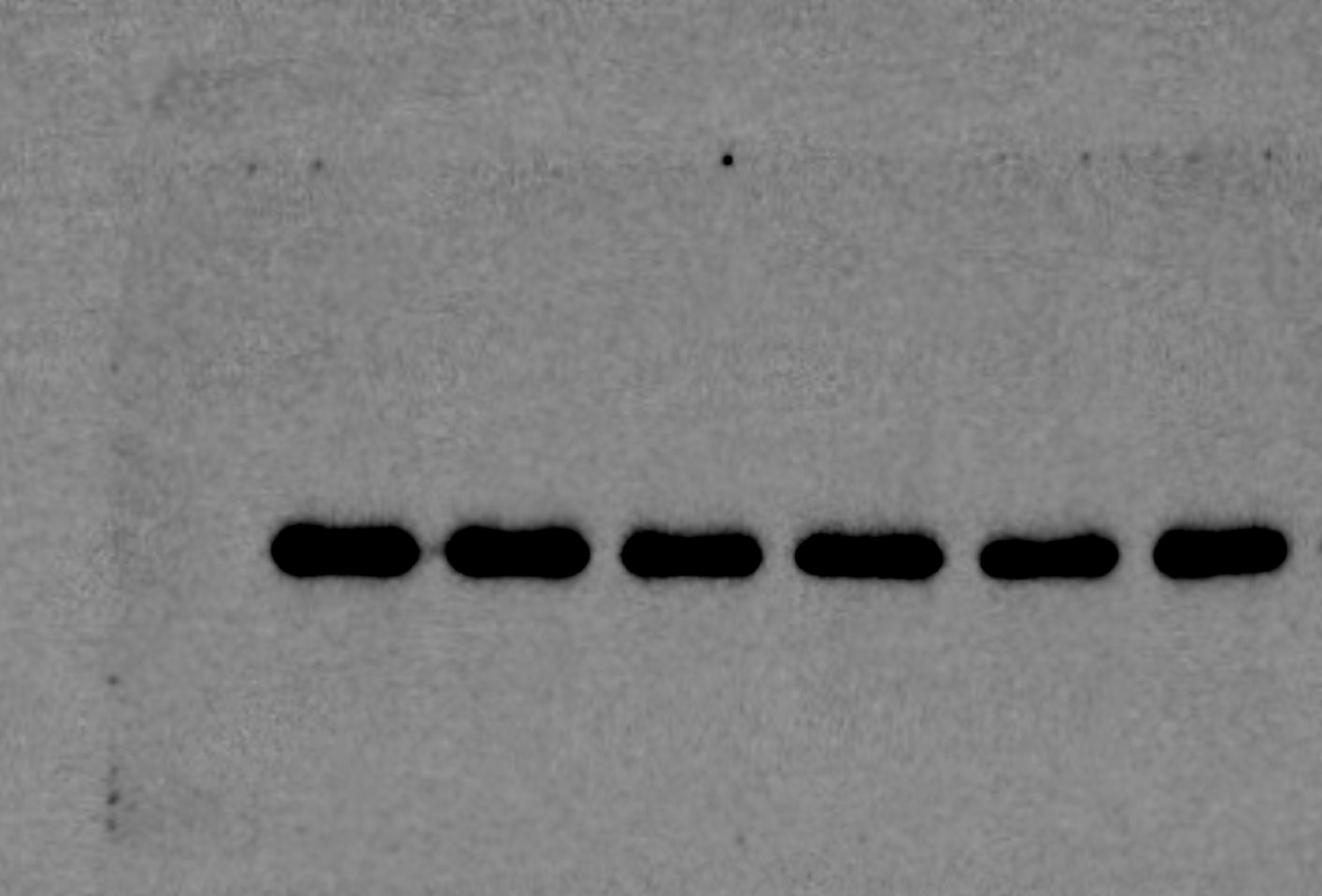

Supplement: Figure 2—source data 1. [file elife-73792-fig2-data1.zip › Figure 2-source data 1/Fig 2F/Figure 2F GAPDH-raw.tif]

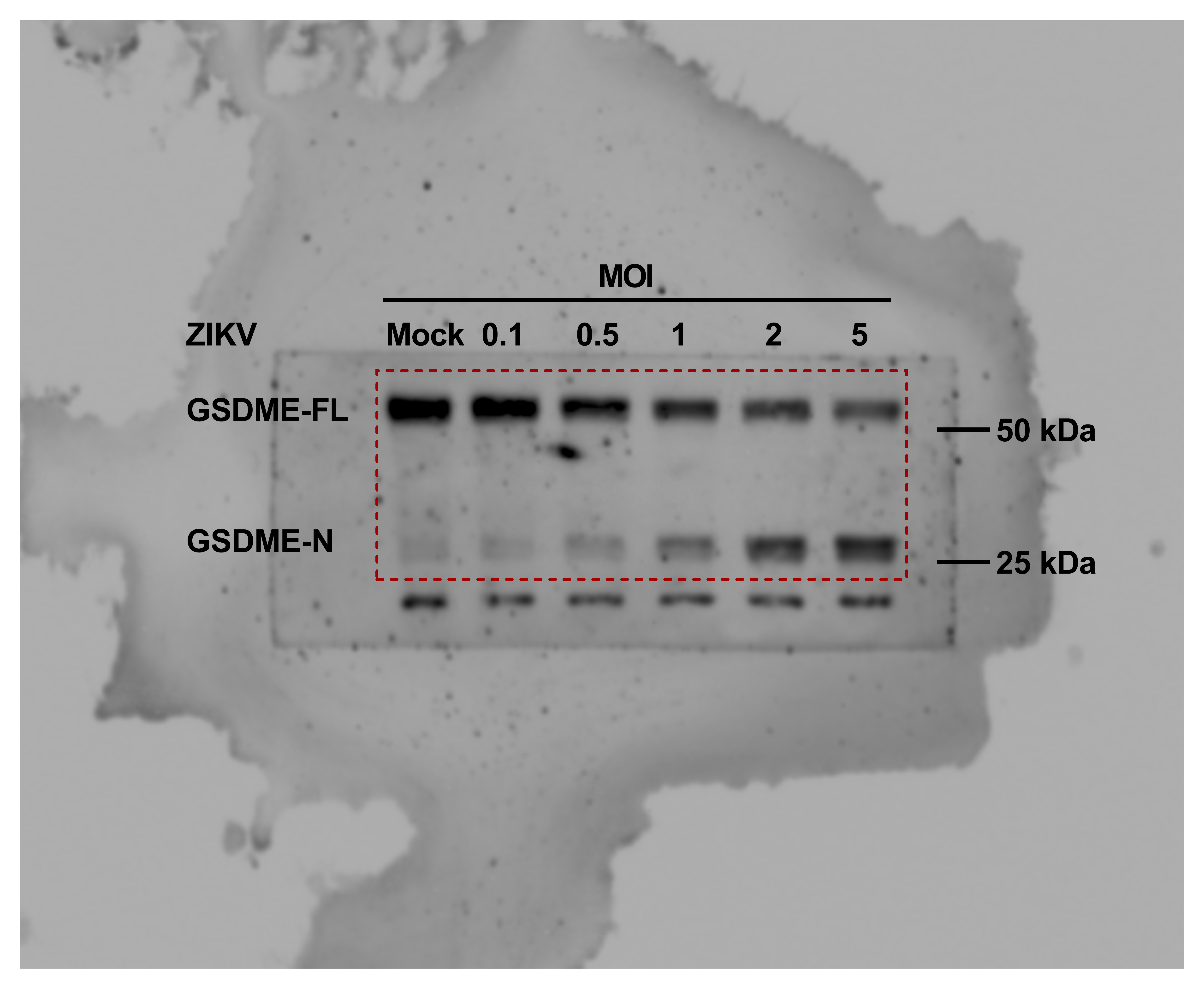

Supplement: Figure 2—source data 1. [file elife-73792-fig2-data1.zip › Figure 2-source data 1/Fig 2F/Figure 2F GSDME-labeled.tif]

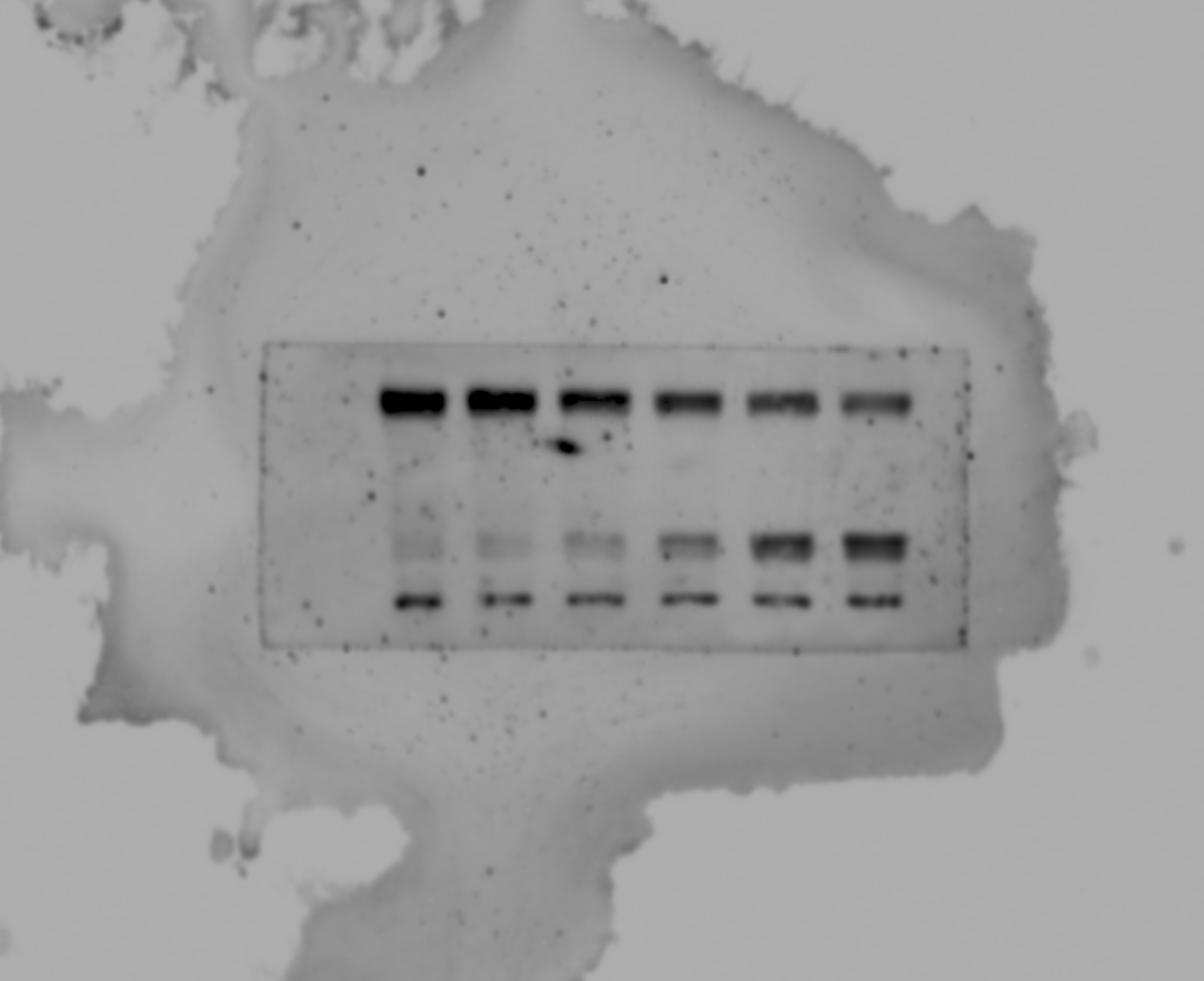

Supplement: Figure 2—source data 1. [file elife-73792-fig2-data1.zip › Figure 2-source data 1/Fig 2F/Figure 2F GSDME-raw.tif]

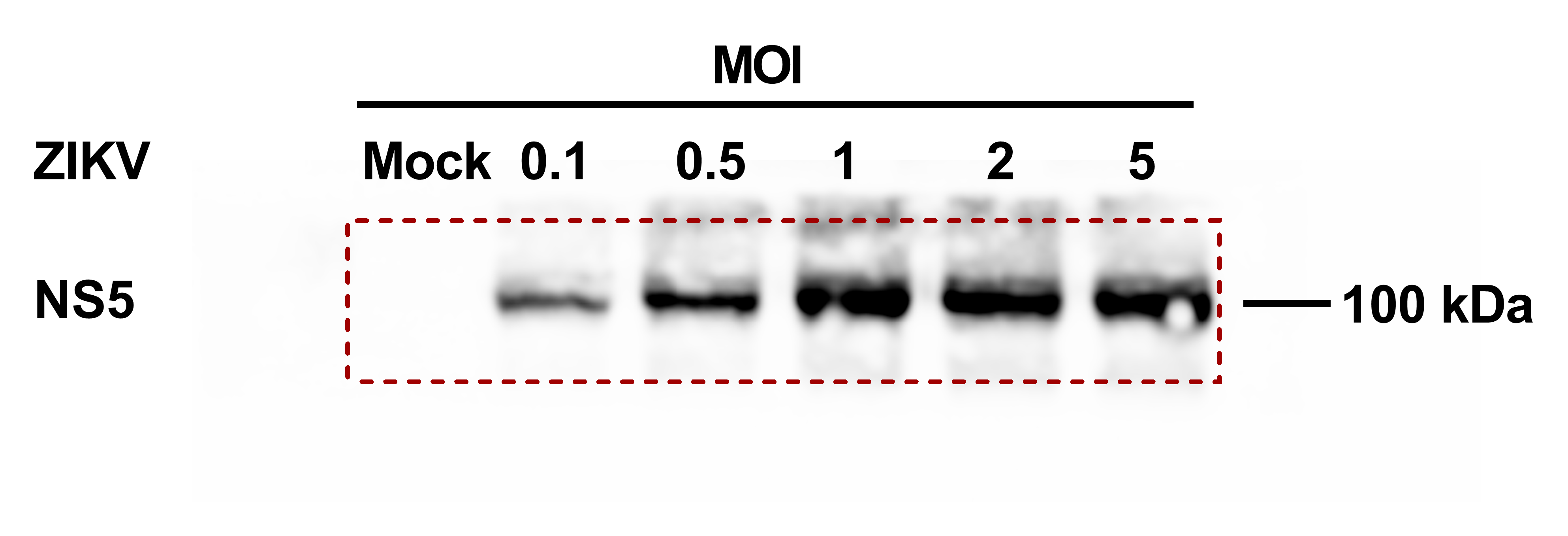

Supplement: Figure 2—source data 1. [file elife-73792-fig2-data1.zip › Figure 2-source data 1/Fig 2F/Figure 2F NS5-labeled.tif]

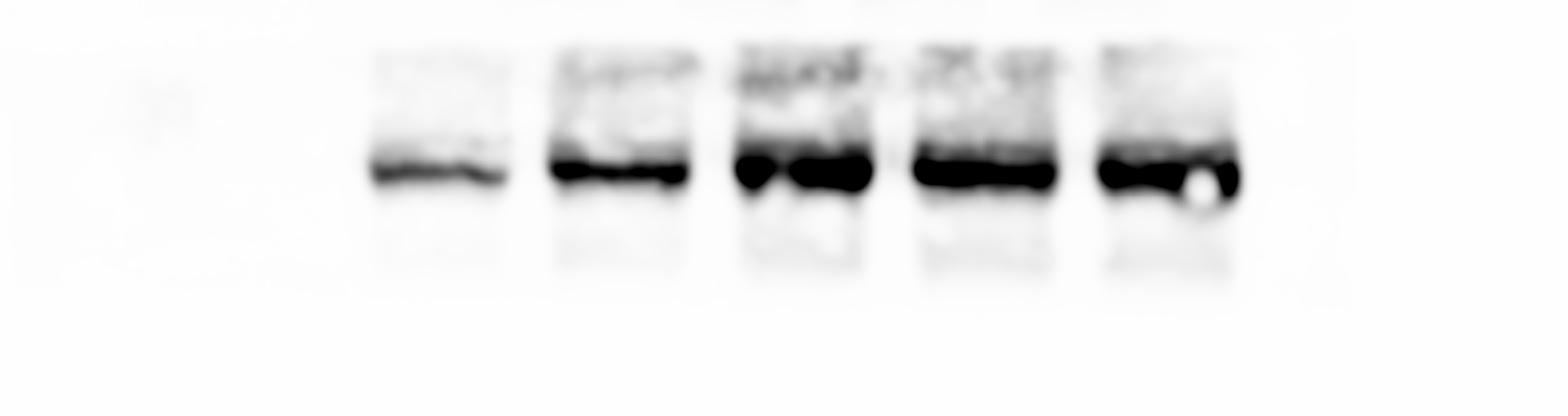

Supplement: Figure 2—source data 1. [file elife-73792-fig2-data1.zip › Figure 2-source data 1/Fig 2F/Figure 2F NS5-raw.tif]

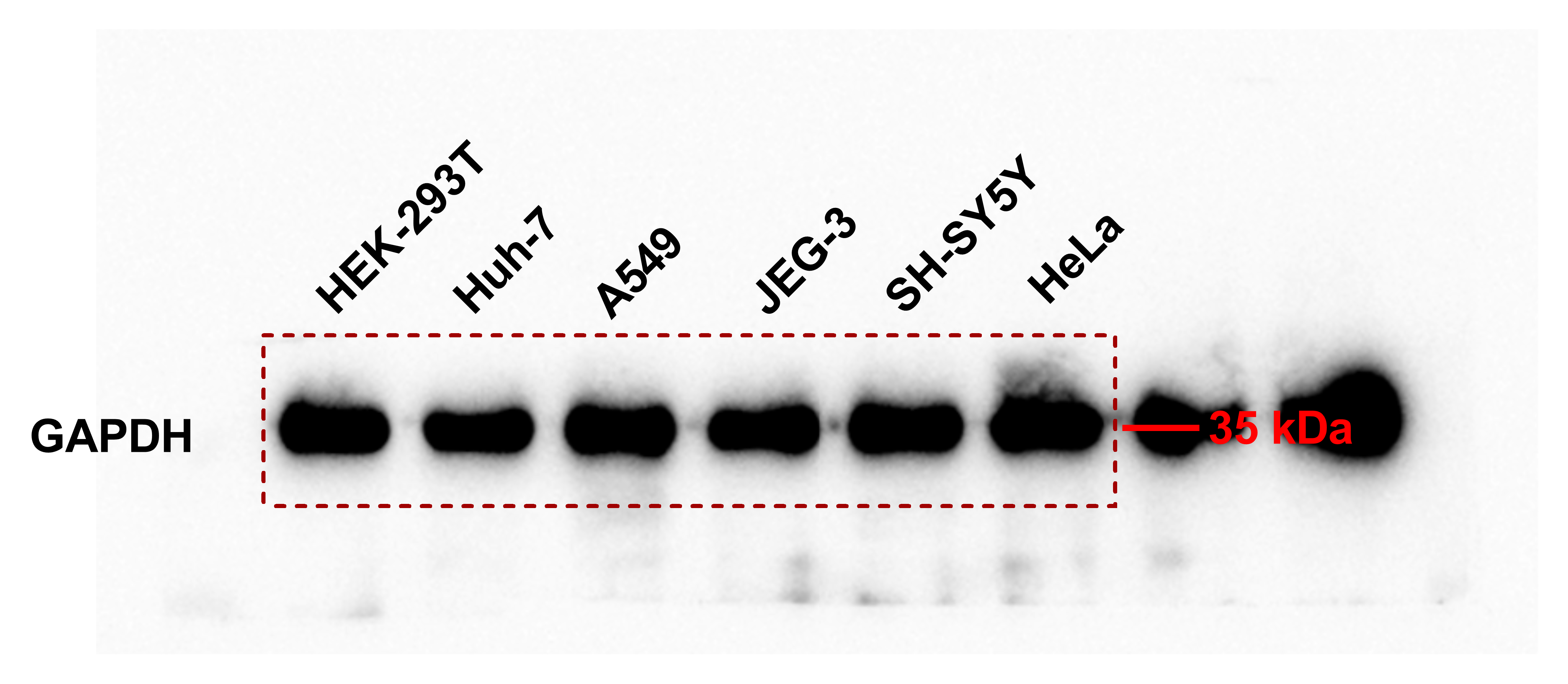

Supplement: Figure 2—figure supplement 1—source data 1. [file elife-73792-fig2-figsupp1-data1.zip › Figure 2-figure supplement 1-source data/1a/Figure 2-figure supplement 1 GAPDH-labeled.tif]

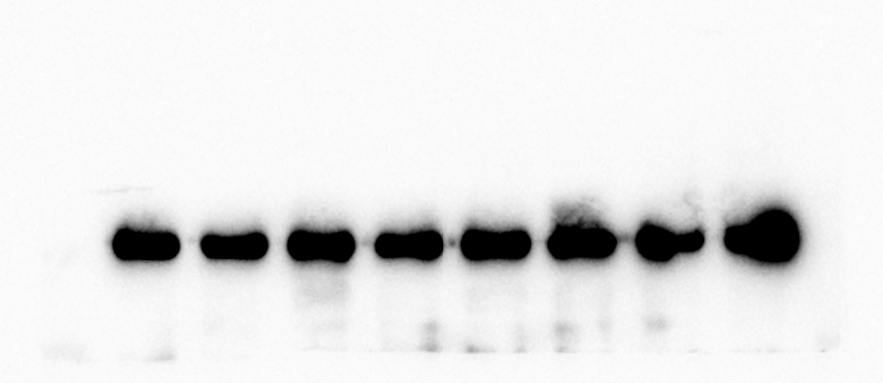

Supplement: Figure 2—figure supplement 1—source data 1. [file elife-73792-fig2-figsupp1-data1.zip › Figure 2-figure supplement 1-source data/1a/Figure 2-figure supplement 1 GAPDH-raw.tif]

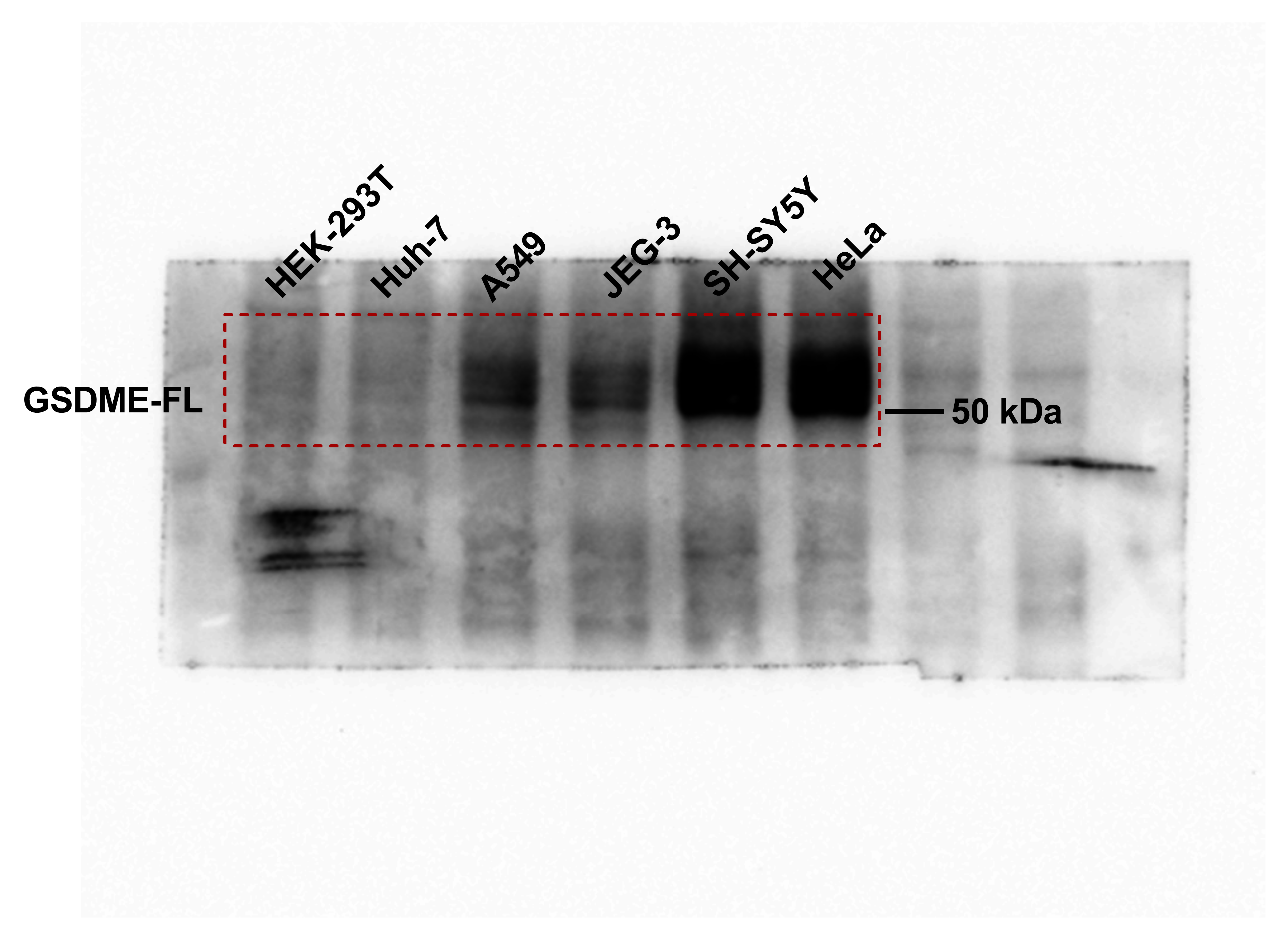

Supplement: Figure 2—figure supplement 1—source data 1. [file elife-73792-fig2-figsupp1-data1.zip › Figure 2-figure supplement 1-source data/1a/Figure 2-figure supplement 1 GSDME-labeled.tif]

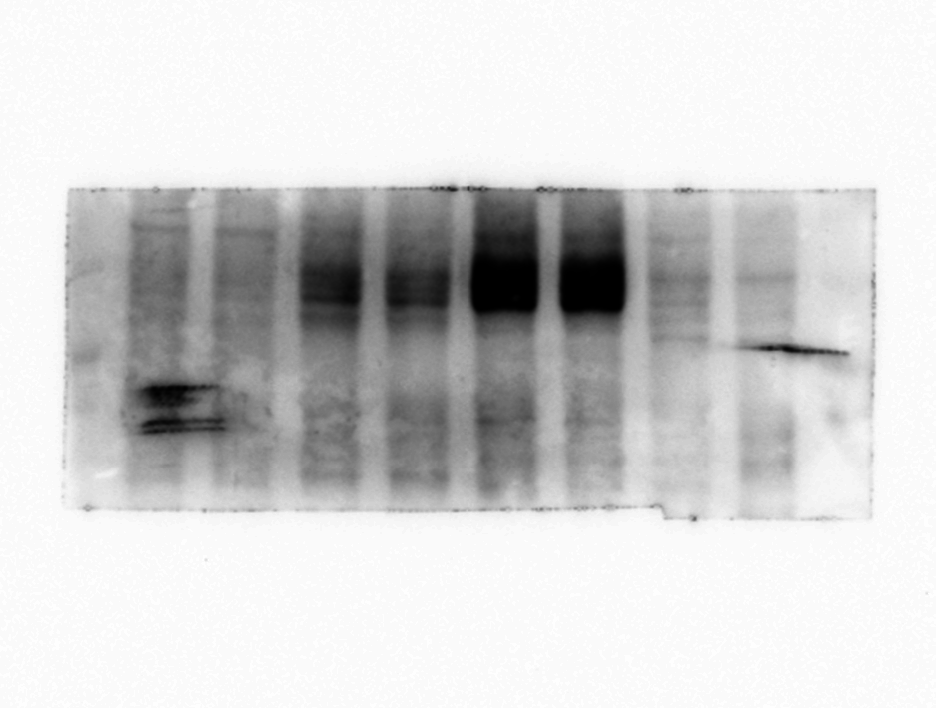

Supplement: Figure 2—figure supplement 1—source data 1. [file elife-73792-fig2-figsupp1-data1.zip › Figure 2-figure supplement 1-source data/1a/Figure 2-figure supplement 1 GSDME-raw.tif.tif]

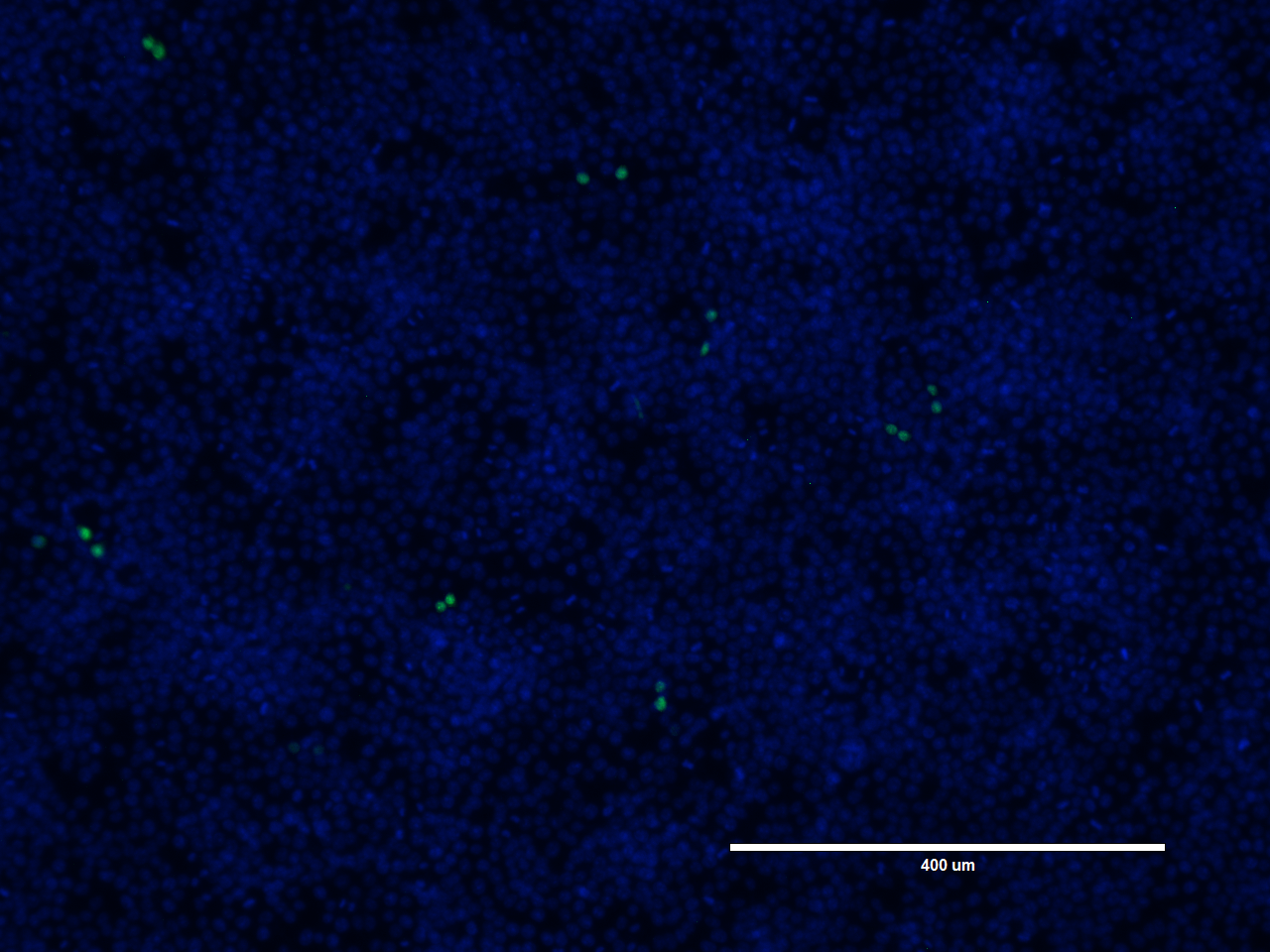

Supplement: Figure 2—figure supplement 1—source data 1. [file elife-73792-fig2-figsupp1-data1.zip › Figure 2-figure supplement 1-source data/1b/293/293 24.tif]

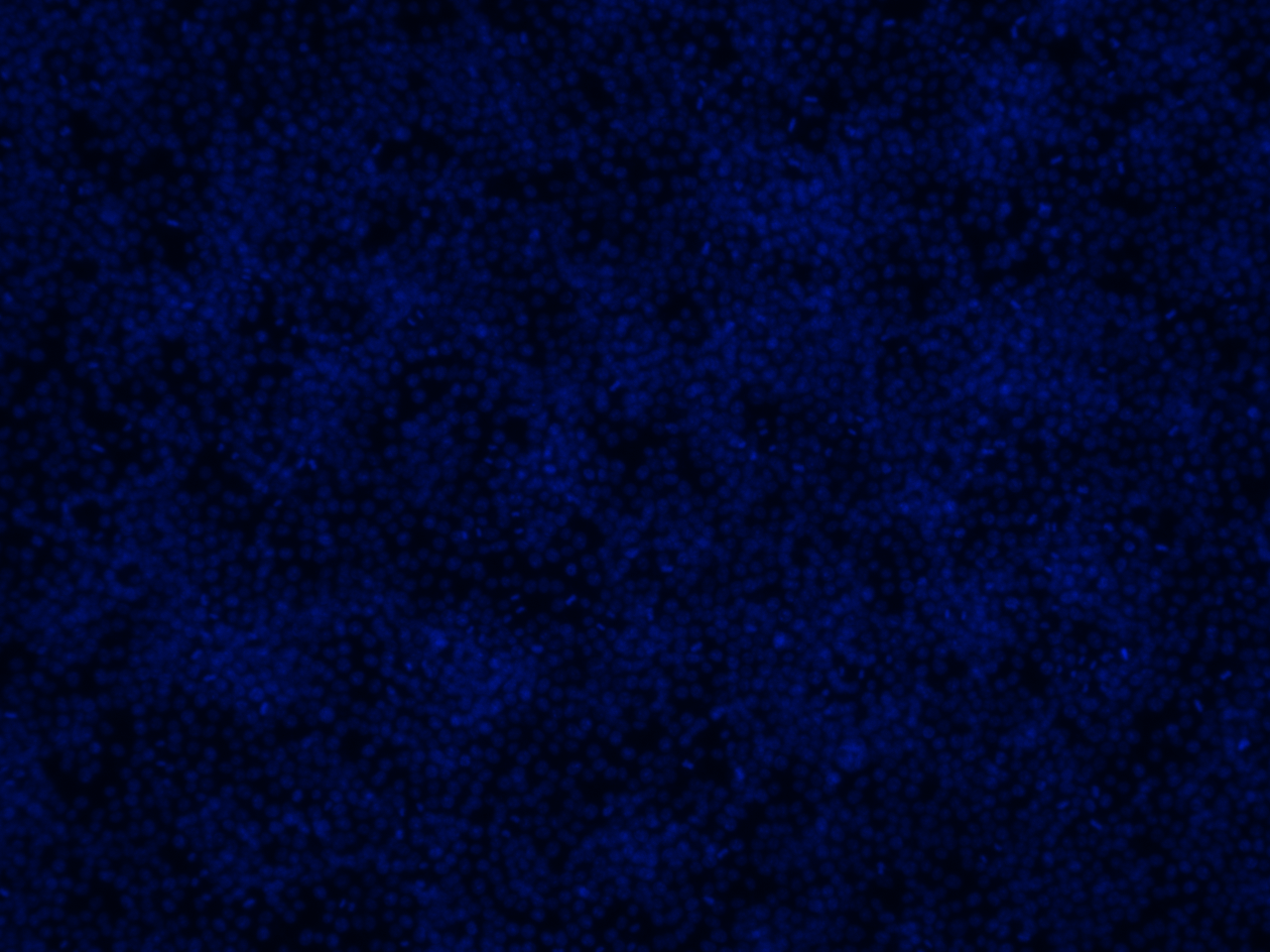

Supplement: Figure 2—figure supplement 1—source data 1. [file elife-73792-fig2-figsupp1-data1.zip › Figure 2-figure supplement 1-source data/1b/293/293 24_DAPI.tif]

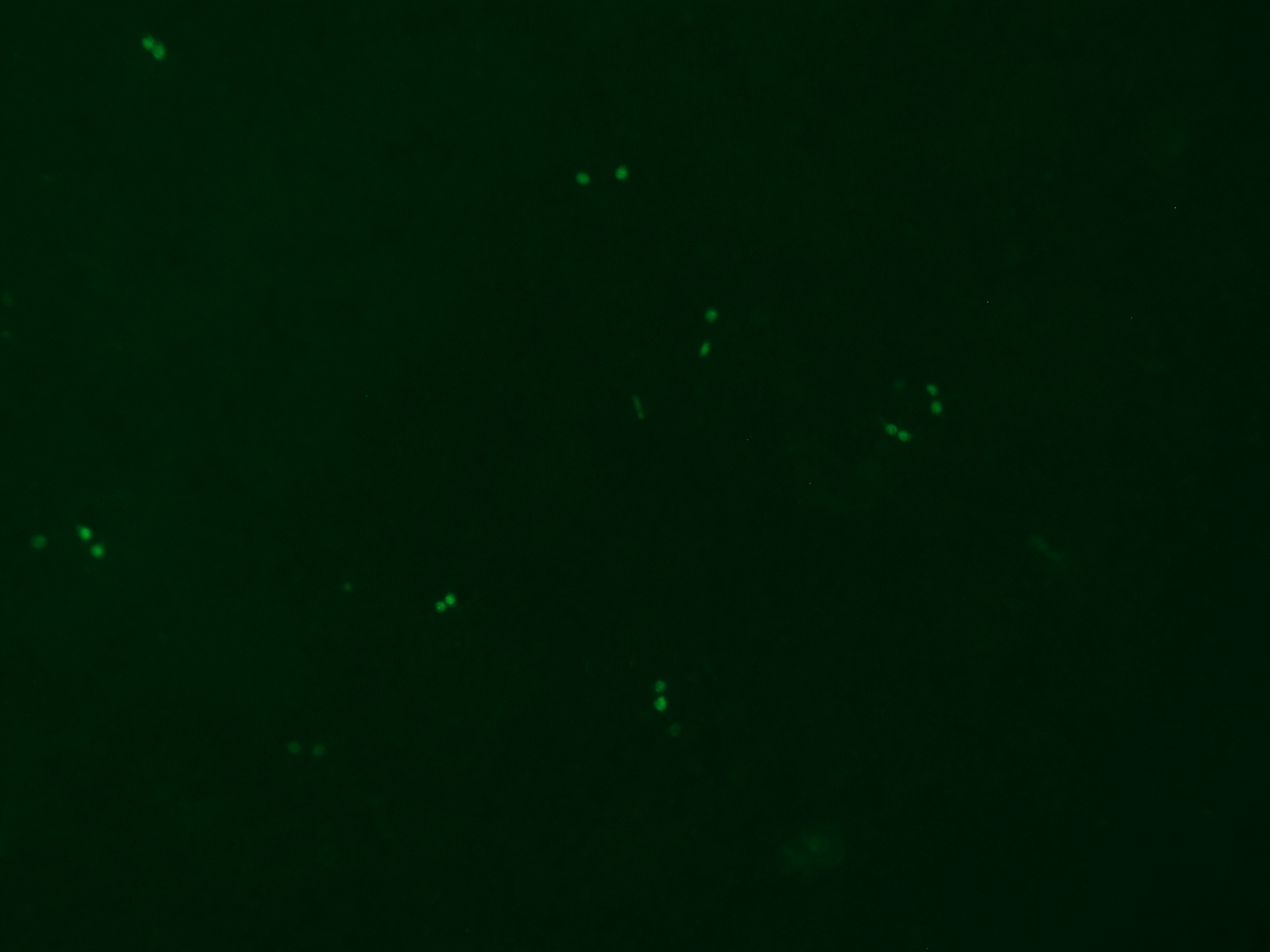

Supplement: Figure 2—figure supplement 1—source data 1. [file elife-73792-fig2-figsupp1-data1.zip › Figure 2-figure supplement 1-source data/1b/293/293 24_GFP.tif]

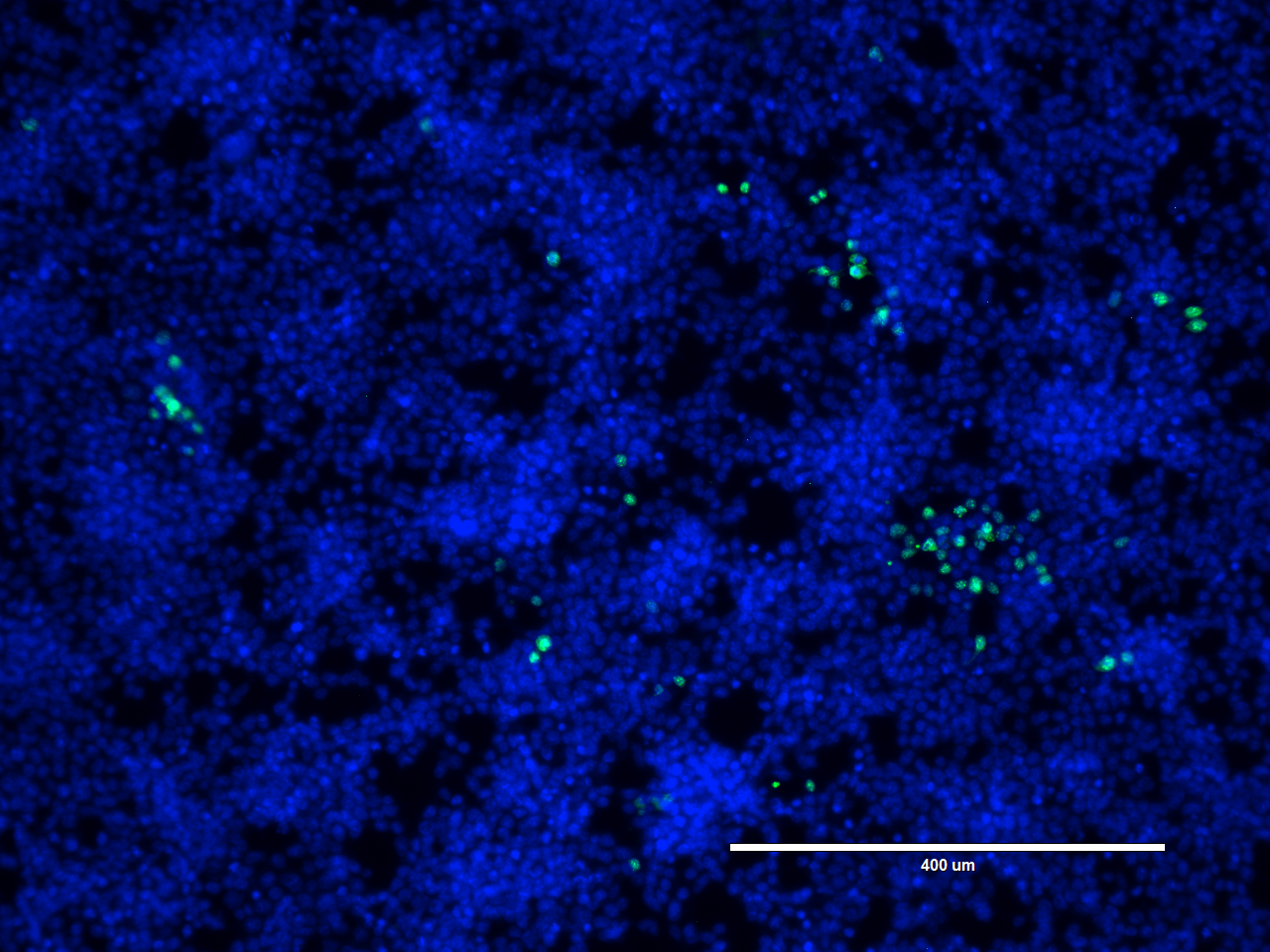

Supplement: Figure 2—figure supplement 1—source data 1. [file elife-73792-fig2-figsupp1-data1.zip › Figure 2-figure supplement 1-source data/1b/293/293 48.tif]

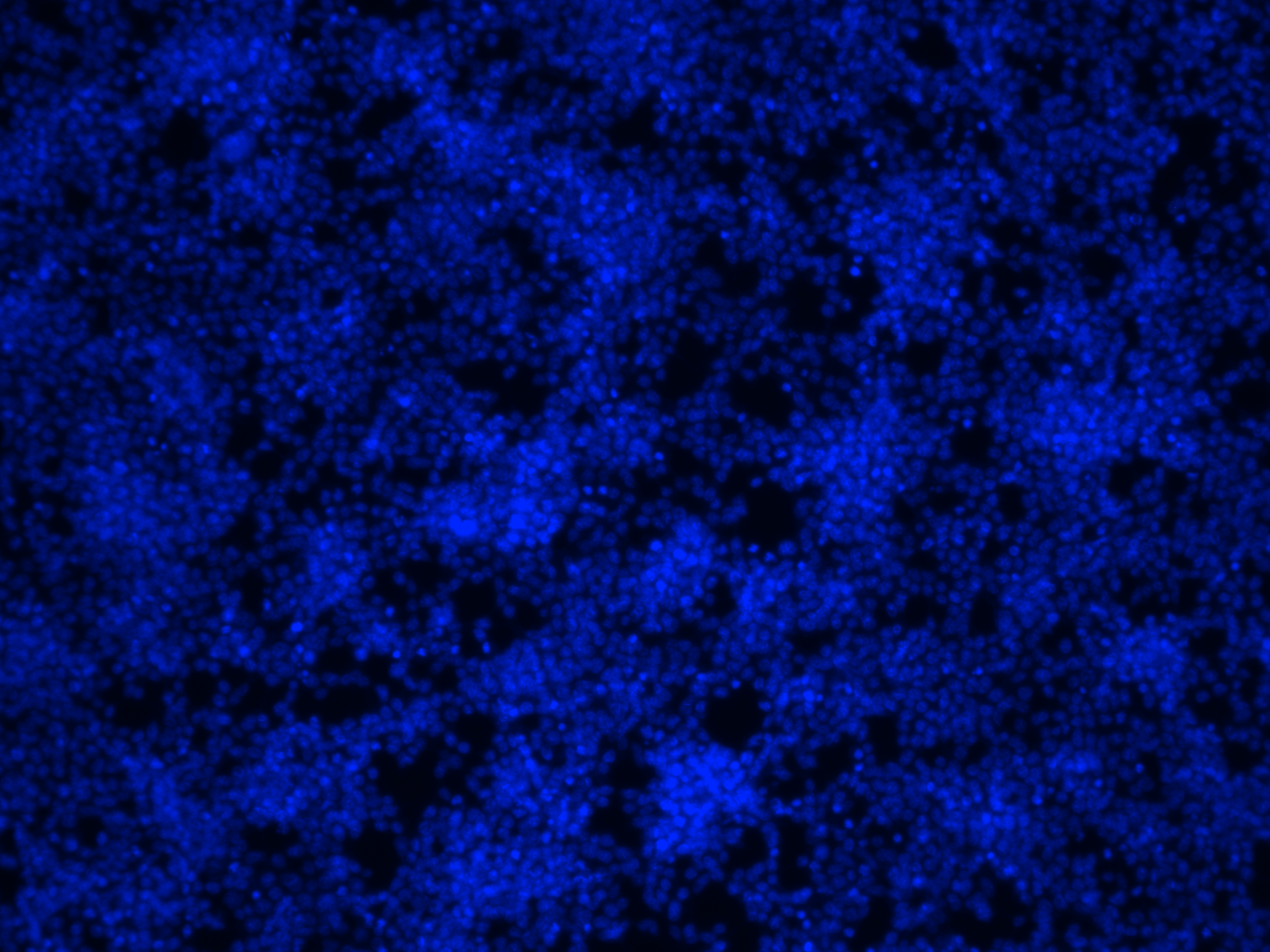

Supplement: Figure 2—figure supplement 1—source data 1. [file elife-73792-fig2-figsupp1-data1.zip › Figure 2-figure supplement 1-source data/1b/293/293 48_DAPI.tif]

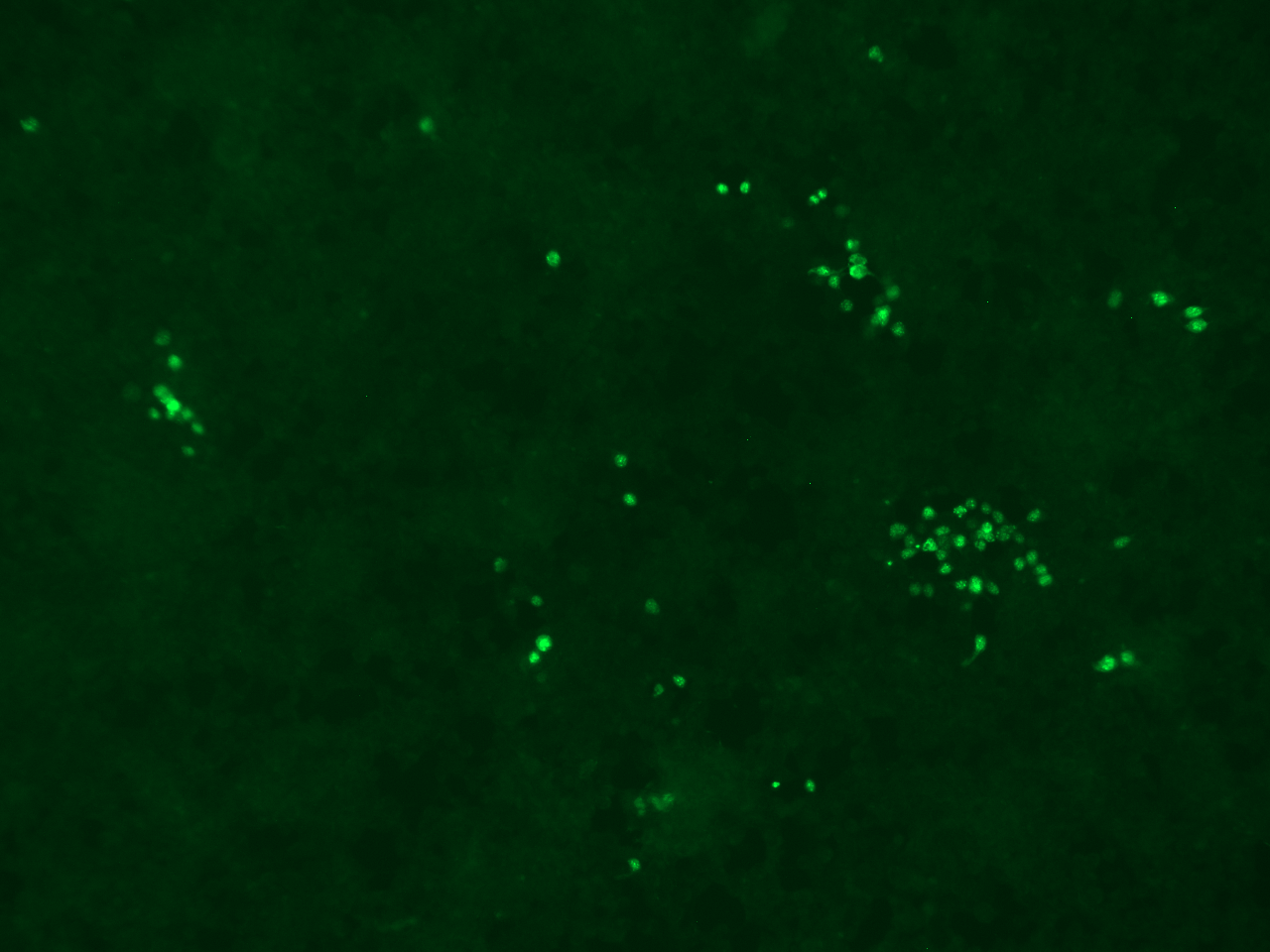

Supplement: Figure 2—figure supplement 1—source data 1. [file elife-73792-fig2-figsupp1-data1.zip › Figure 2-figure supplement 1-source data/1b/293/293 48_GFP.tif]

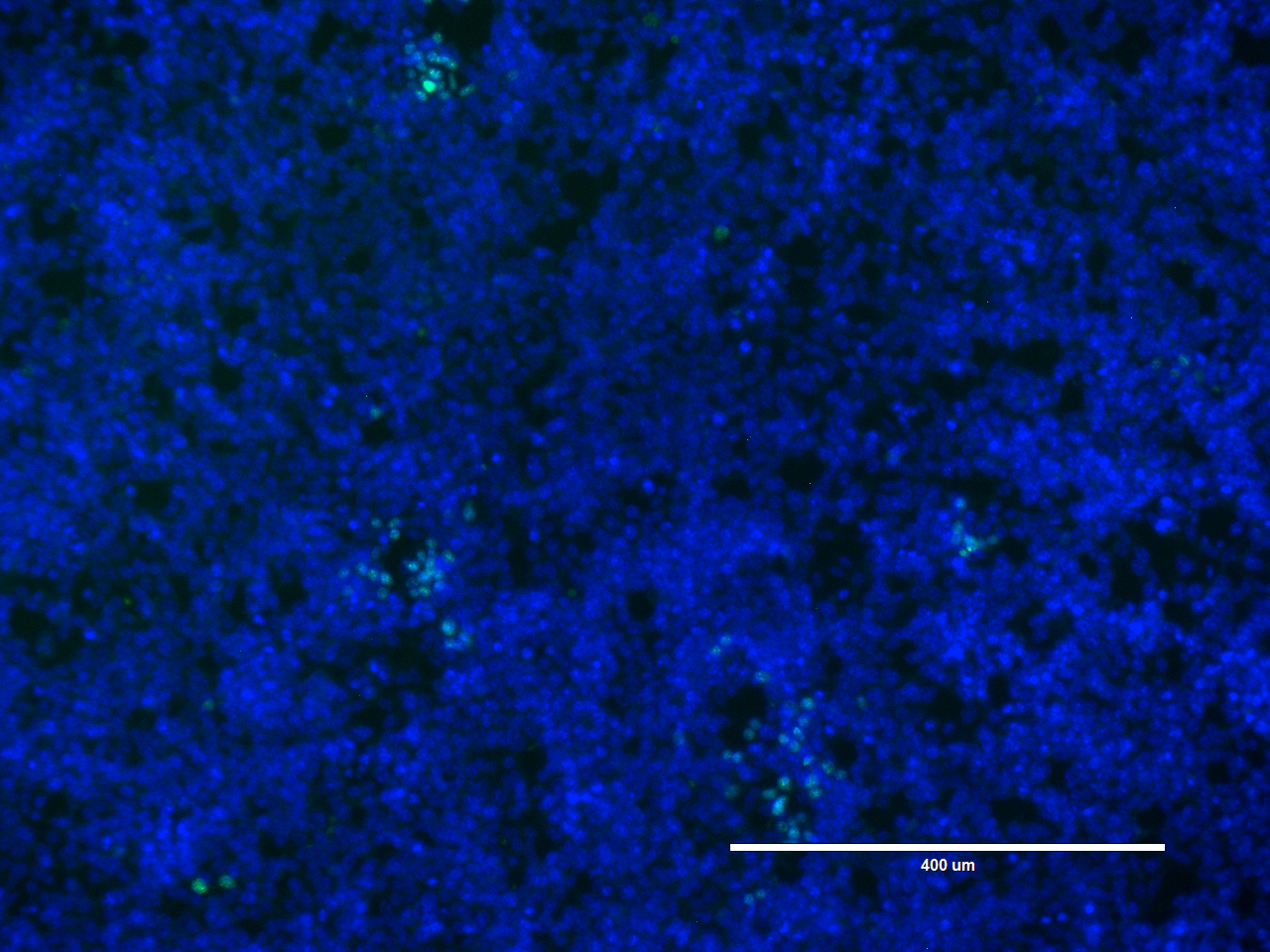

Supplement: Figure 2—figure supplement 1—source data 1. [file elife-73792-fig2-figsupp1-data1.zip › Figure 2-figure supplement 1-source data/1b/293/293 72.tif]

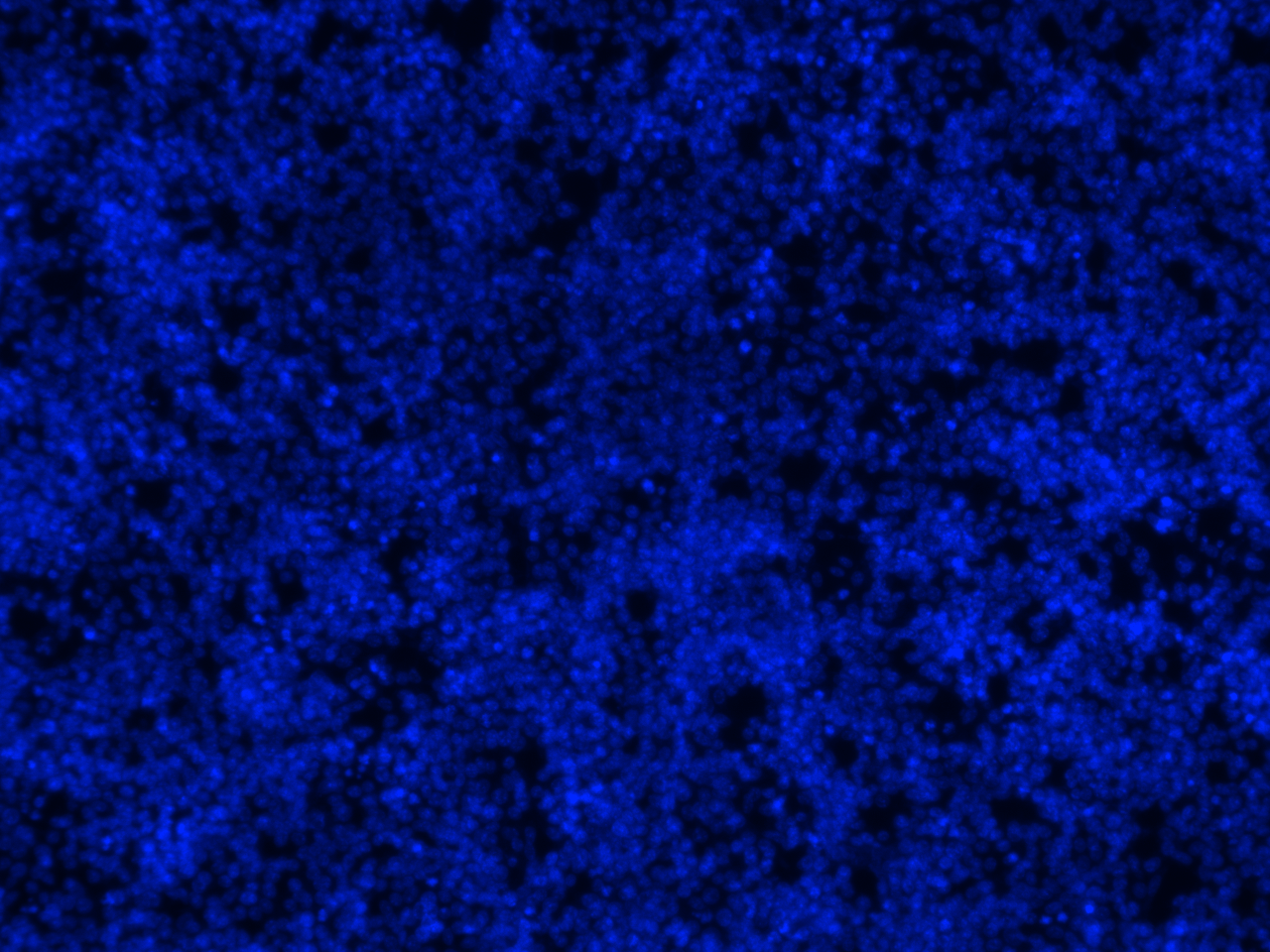

Supplement: Figure 2—figure supplement 1—source data 1. [file elife-73792-fig2-figsupp1-data1.zip › Figure 2-figure supplement 1-source data/1b/293/293 72_DAPI.tif]

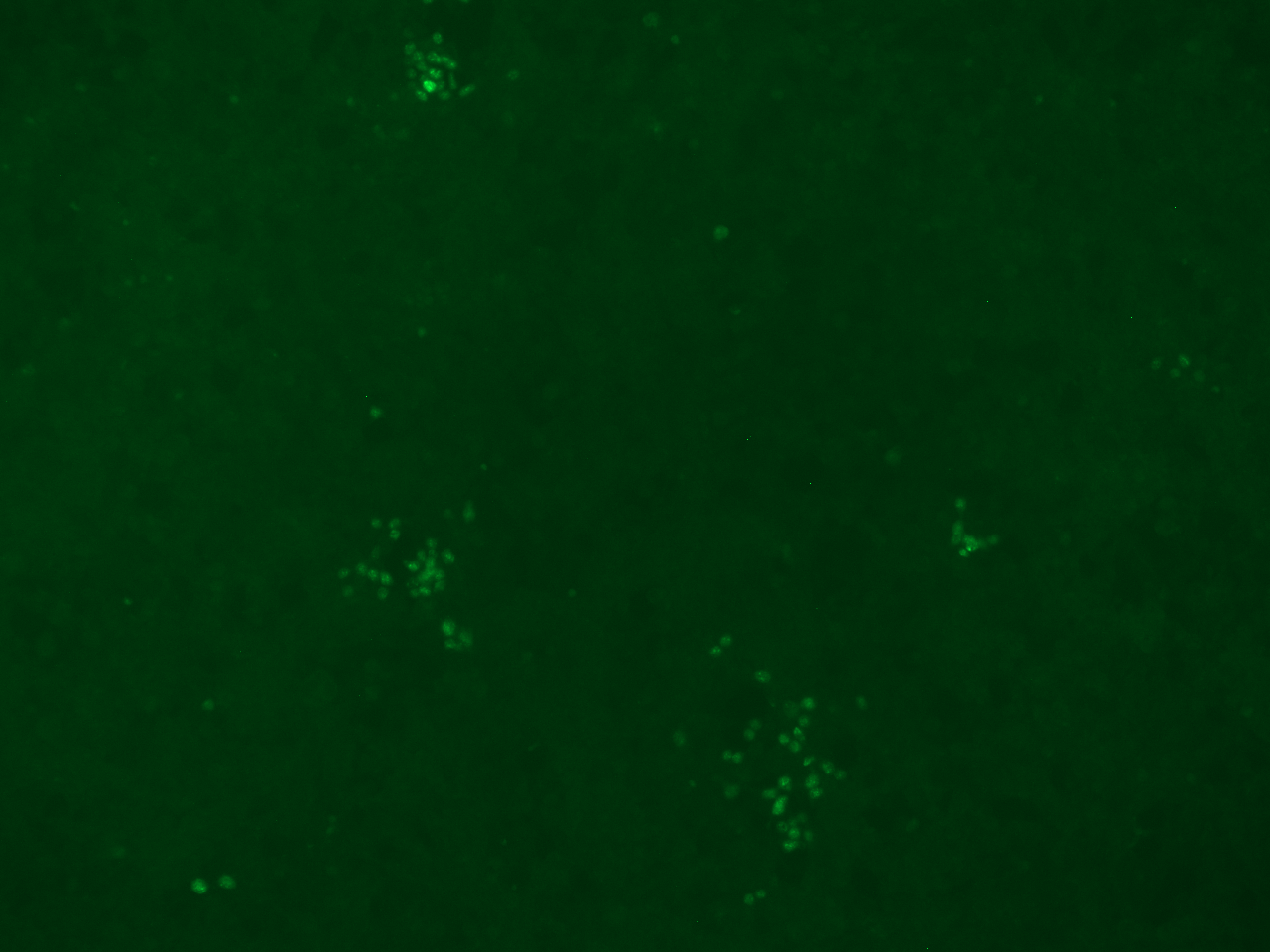

Supplement: Figure 2—figure supplement 1—source data 1. [file elife-73792-fig2-figsupp1-data1.zip › Figure 2-figure supplement 1-source data/1b/293/293 72_GFP.tif]

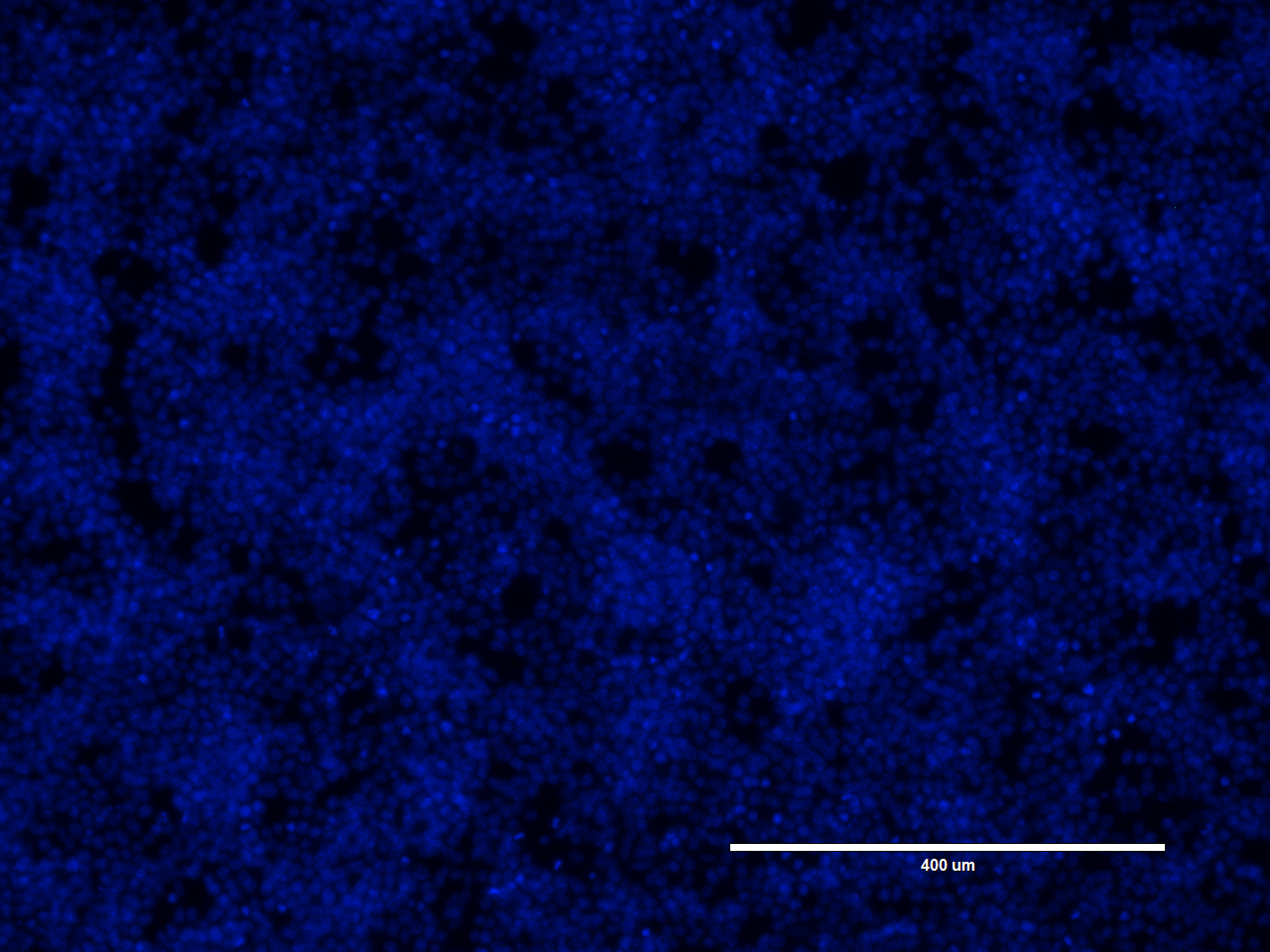

Supplement: Figure 2—figure supplement 1—source data 1. [file elife-73792-fig2-figsupp1-data1.zip › Figure 2-figure supplement 1-source data/1b/293/293 con.tif]

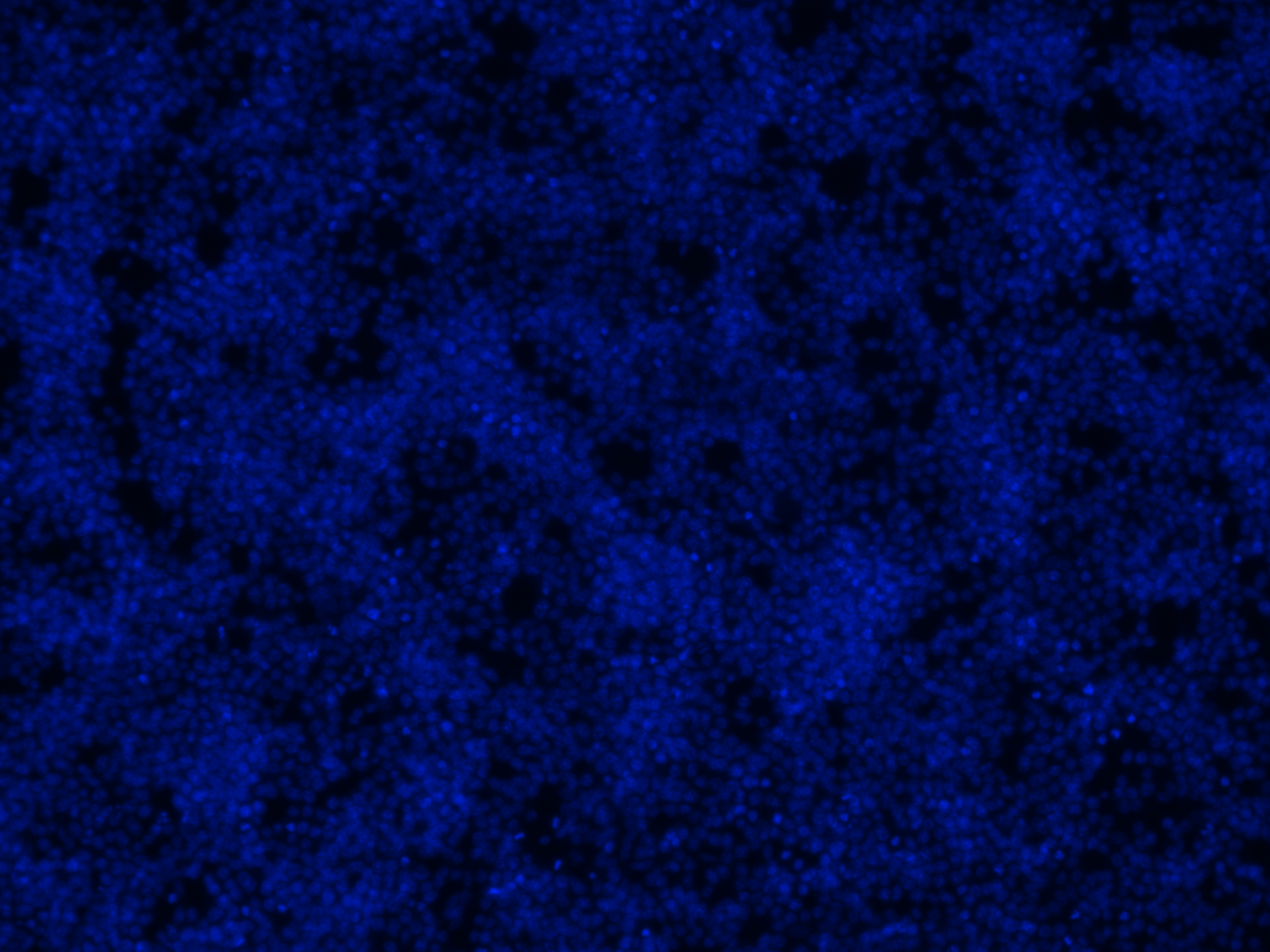

Supplement: Figure 2—figure supplement 1—source data 1. [file elife-73792-fig2-figsupp1-data1.zip › Figure 2-figure supplement 1-source data/1b/293/293 con_DAPI.tif]

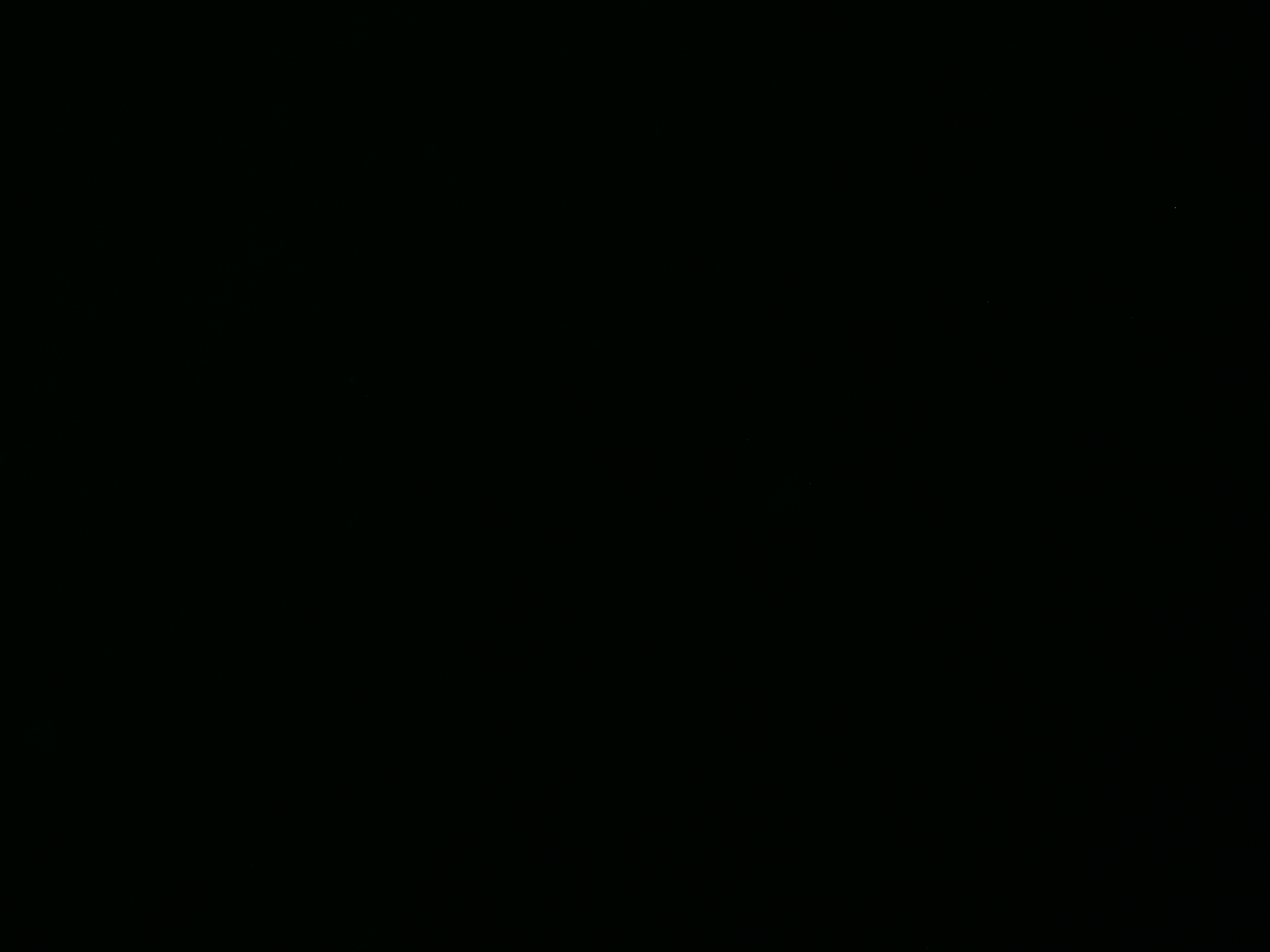

Supplement: Figure 2—figure supplement 1—source data 1. [file elife-73792-fig2-figsupp1-data1.zip › Figure 2-figure supplement 1-source data/1b/293/293 con_GFP.tif]

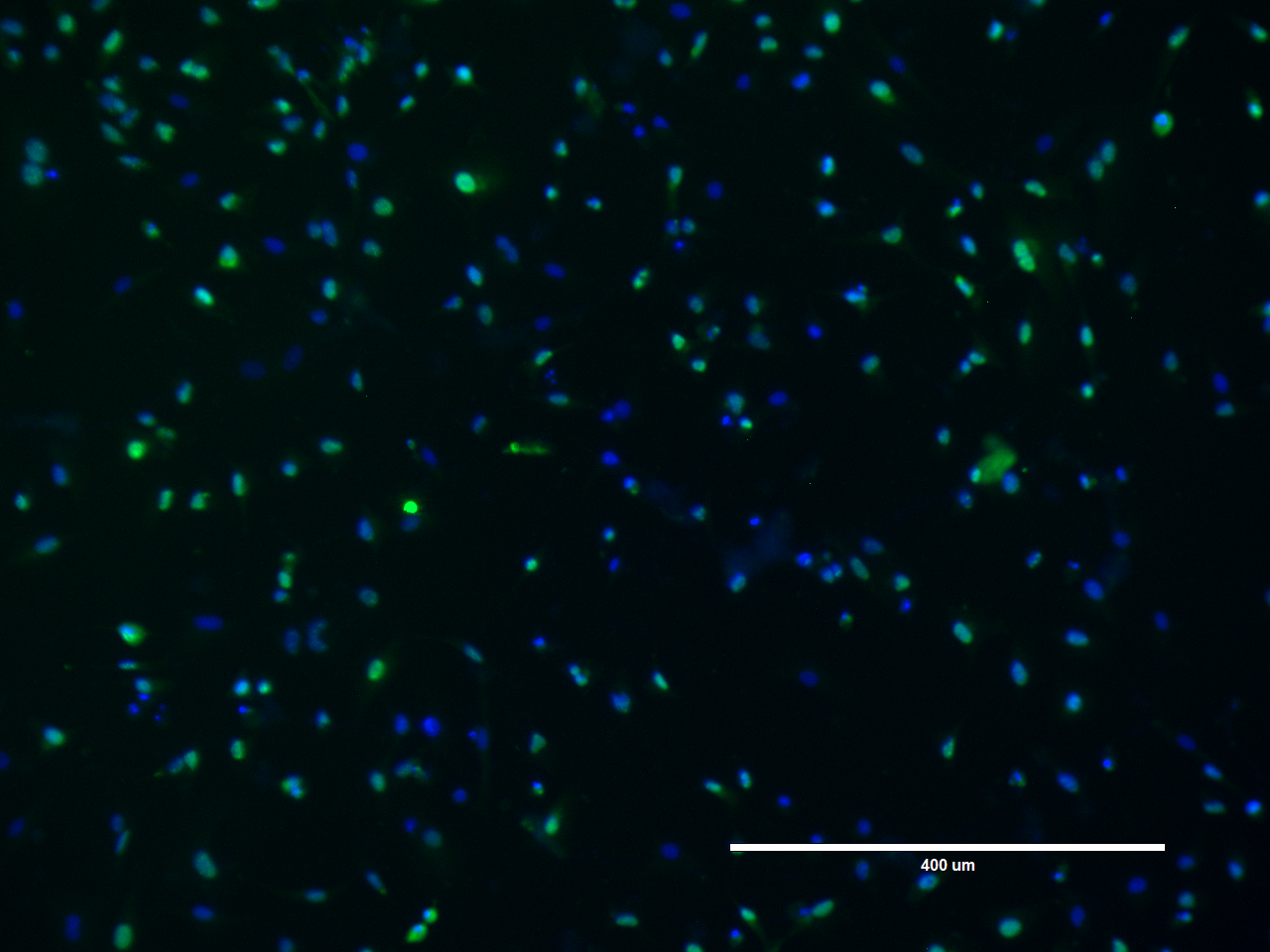

Supplement: Figure 2—figure supplement 1—source data 1. [file elife-73792-fig2-figsupp1-data1.zip › Figure 2-figure supplement 1-source data/1b/a549/a549 24h.tif]

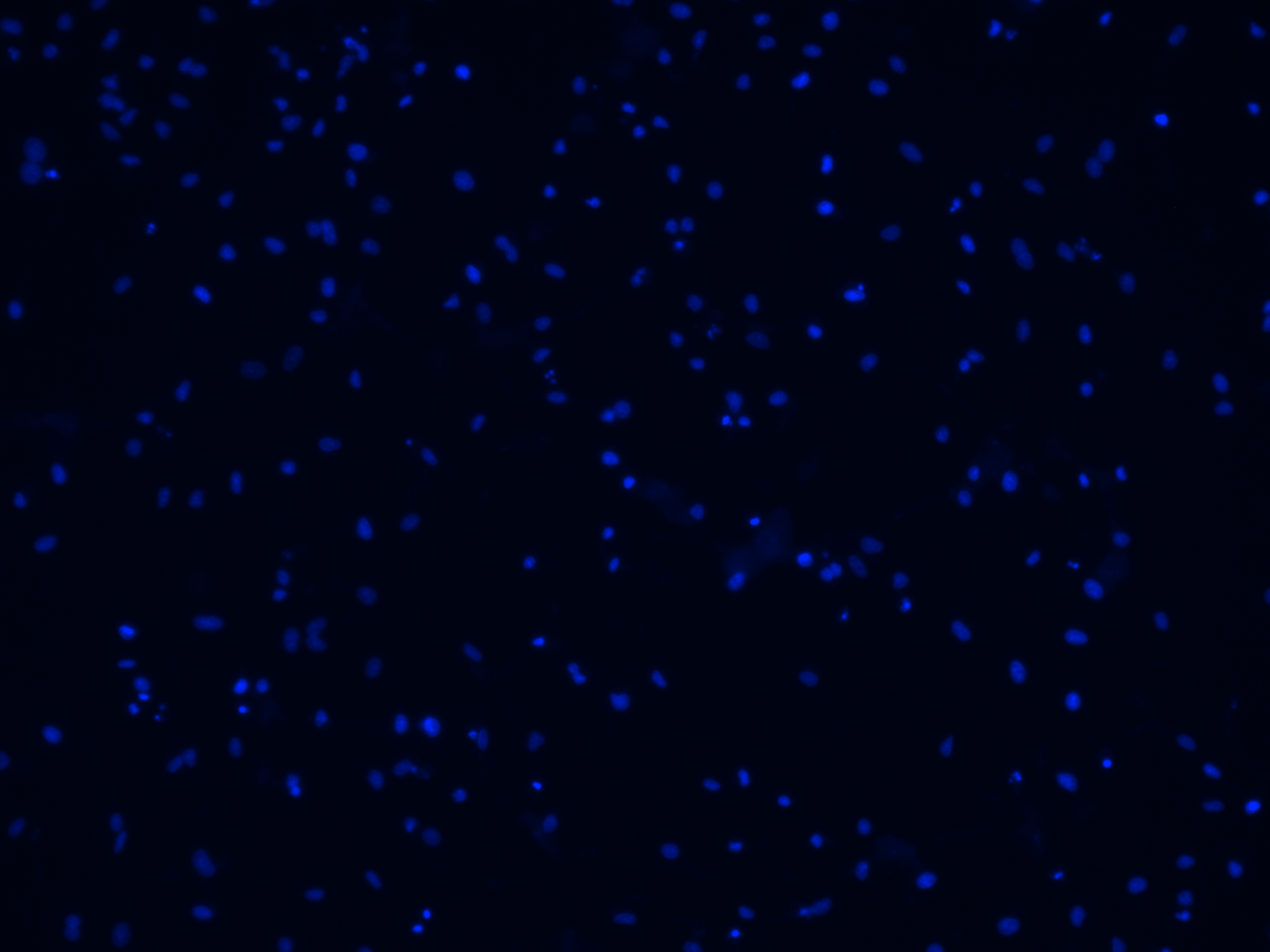

Supplement: Figure 2—figure supplement 1—source data 1. [file elife-73792-fig2-figsupp1-data1.zip › Figure 2-figure supplement 1-source data/1b/a549/a549 24h_DAPI.tif]

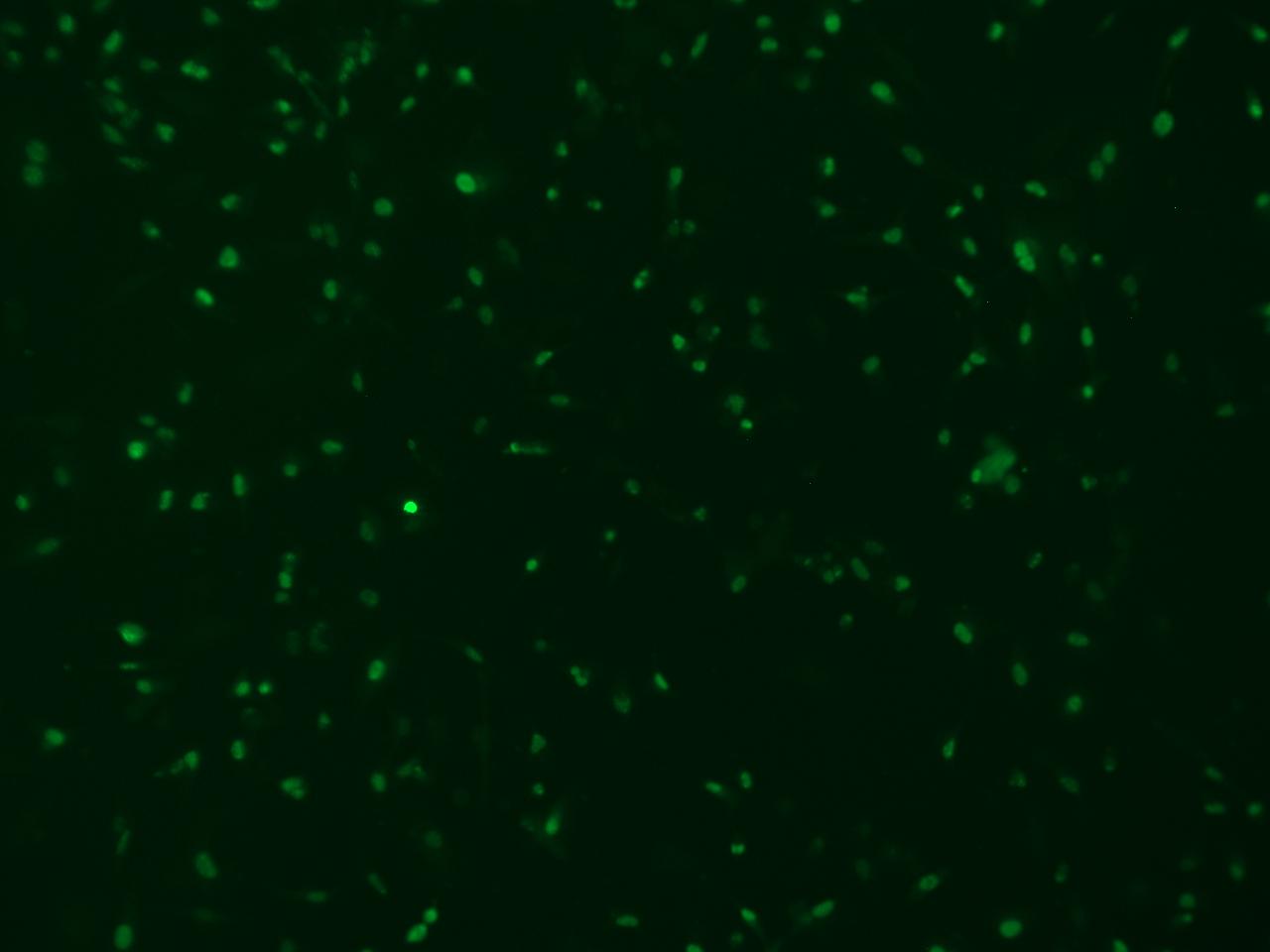

Supplement: Figure 2—figure supplement 1—source data 1. [file elife-73792-fig2-figsupp1-data1.zip › Figure 2-figure supplement 1-source data/1b/a549/a549 24h_GFP.tif]

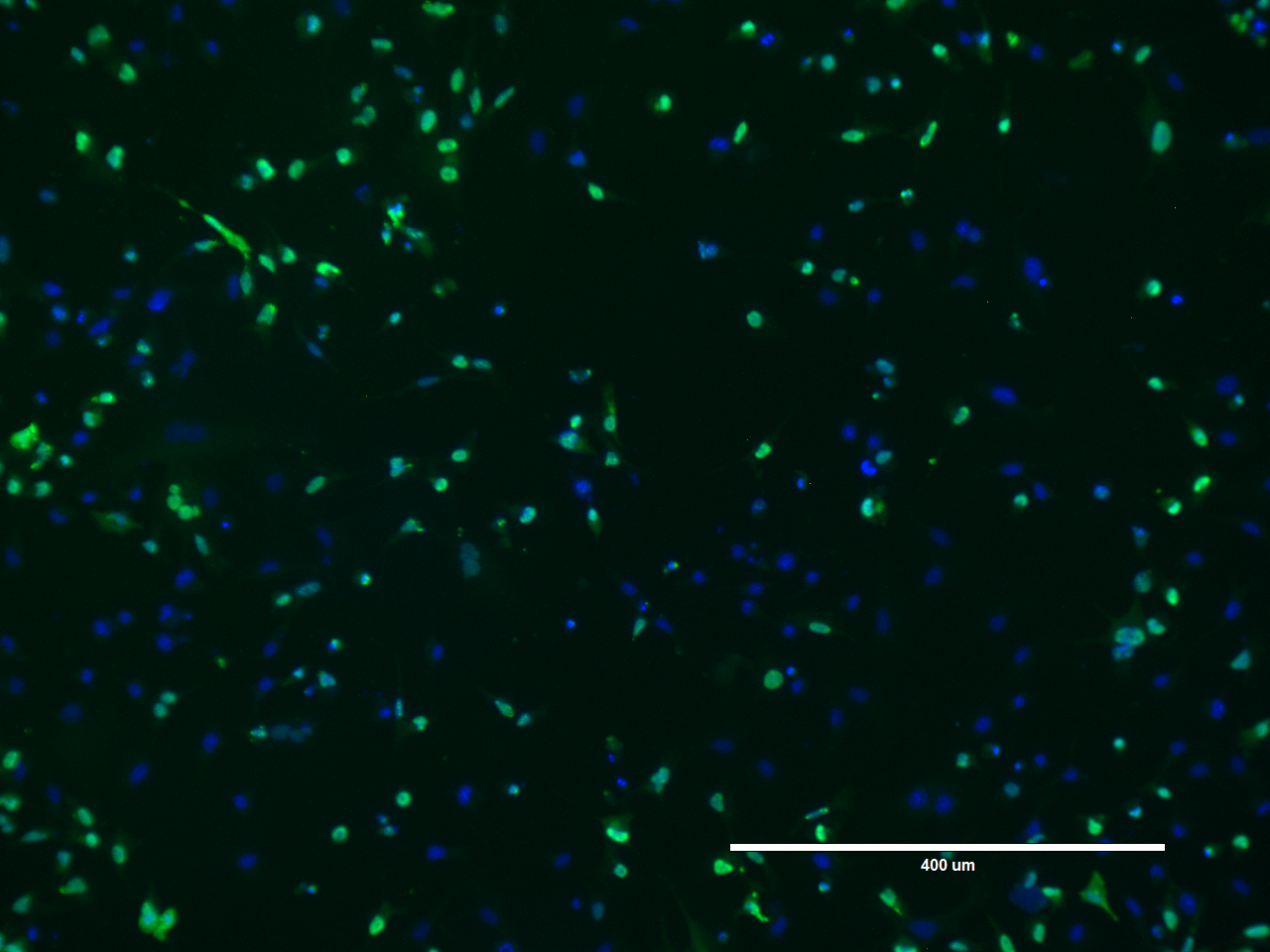

Supplement: Figure 2—figure supplement 1—source data 1. [file elife-73792-fig2-figsupp1-data1.zip › Figure 2-figure supplement 1-source data/1b/a549/a549 72.tif]

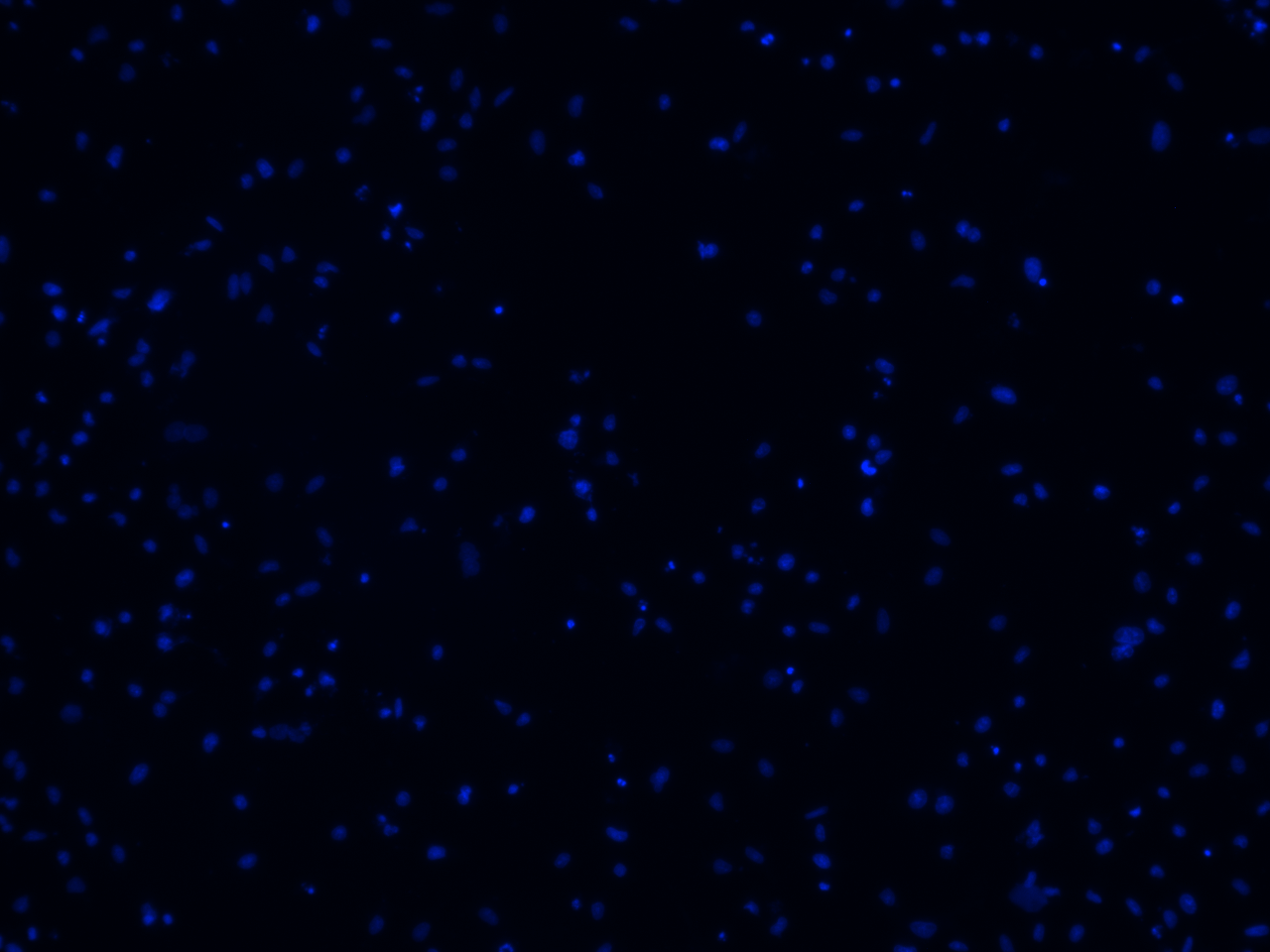

Supplement: Figure 2—figure supplement 1—source data 1. [file elife-73792-fig2-figsupp1-data1.zip › Figure 2-figure supplement 1-source data/1b/a549/a549 72_DAPI.tif]

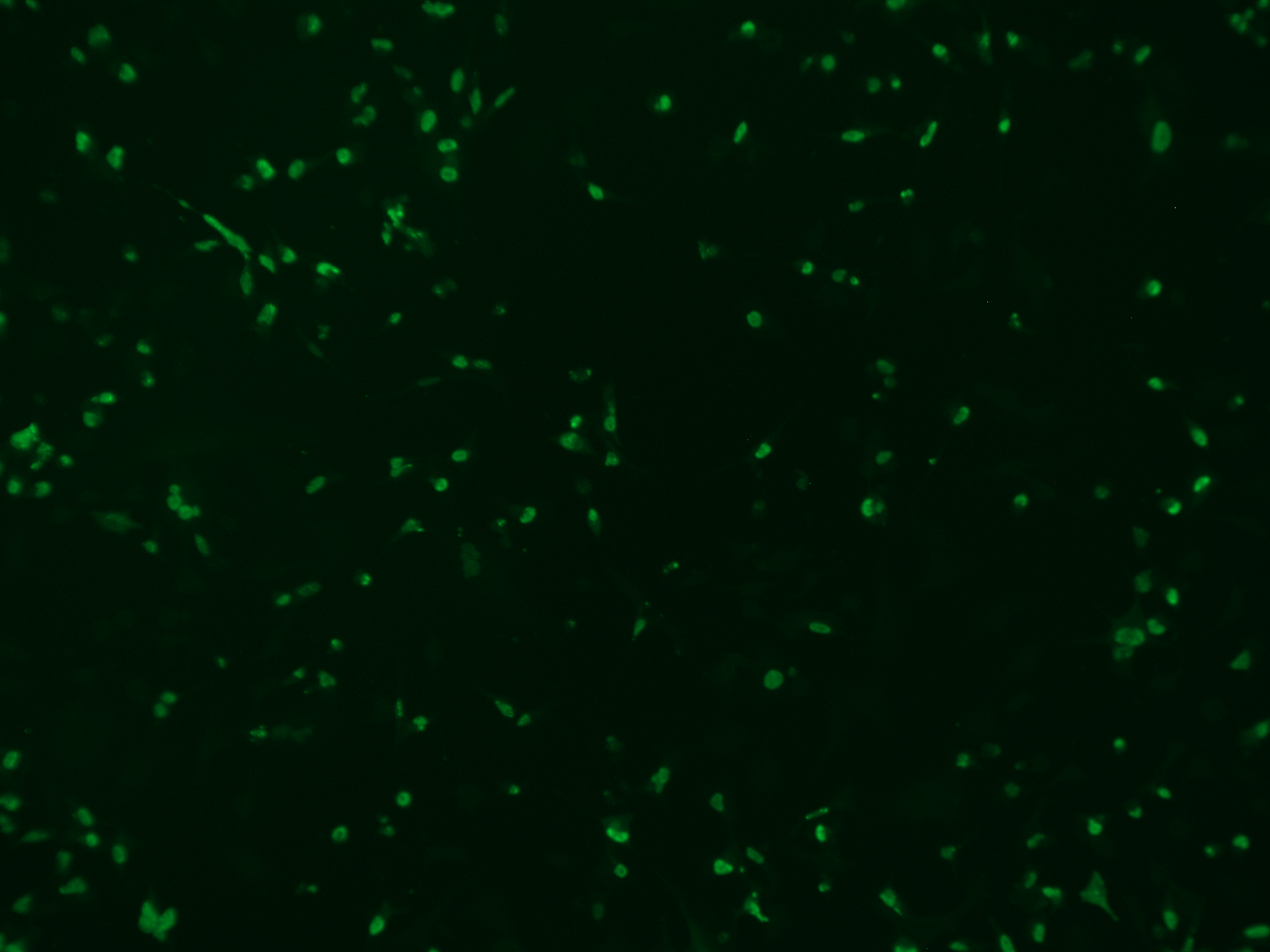

Supplement: Figure 2—figure supplement 1—source data 1. [file elife-73792-fig2-figsupp1-data1.zip › Figure 2-figure supplement 1-source data/1b/a549/a549 72_GFP.tif]

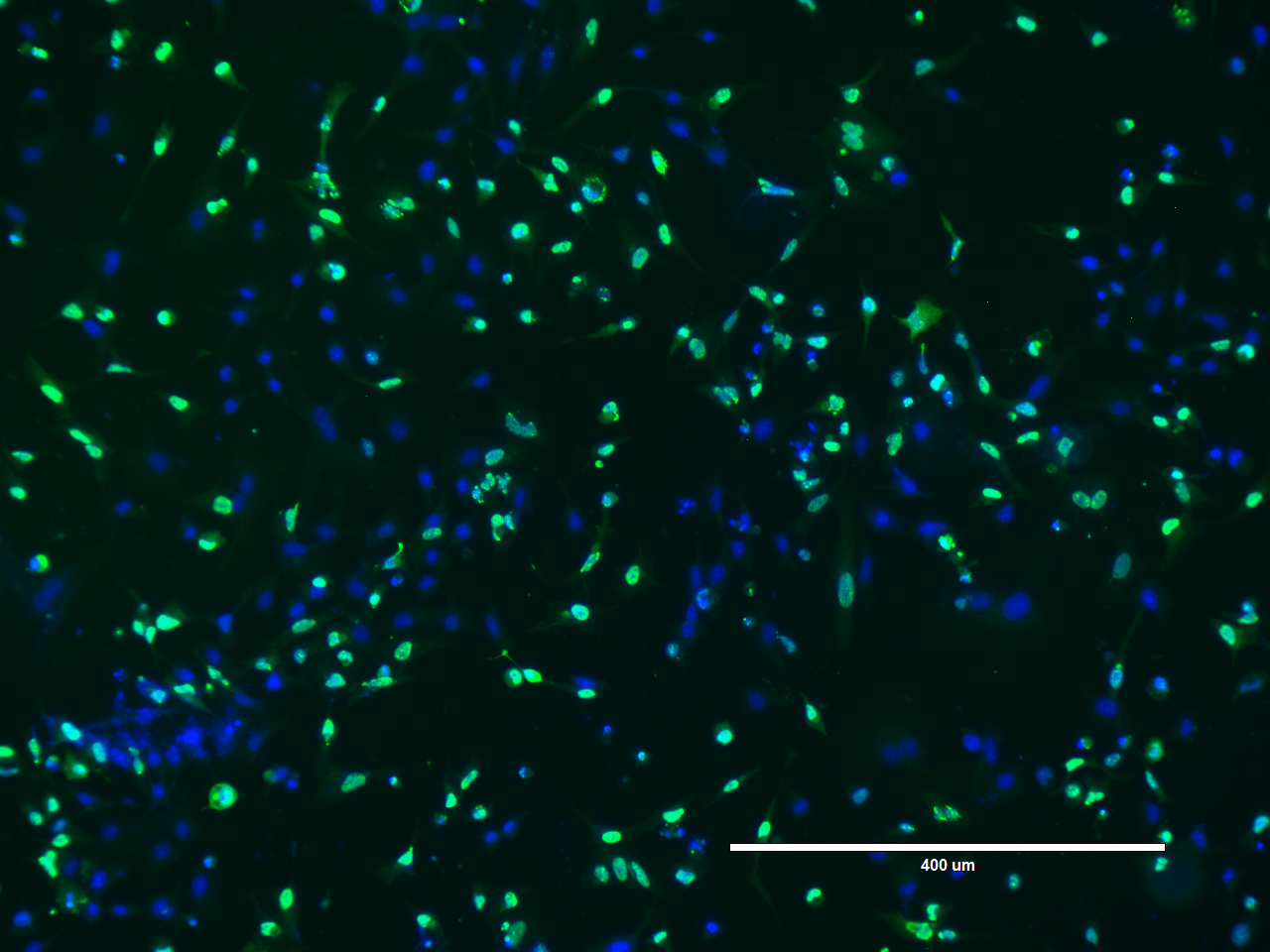

Supplement: Figure 2—figure supplement 1—source data 1. [file elife-73792-fig2-figsupp1-data1.zip › Figure 2-figure supplement 1-source data/1b/a549/e549 48.tif]

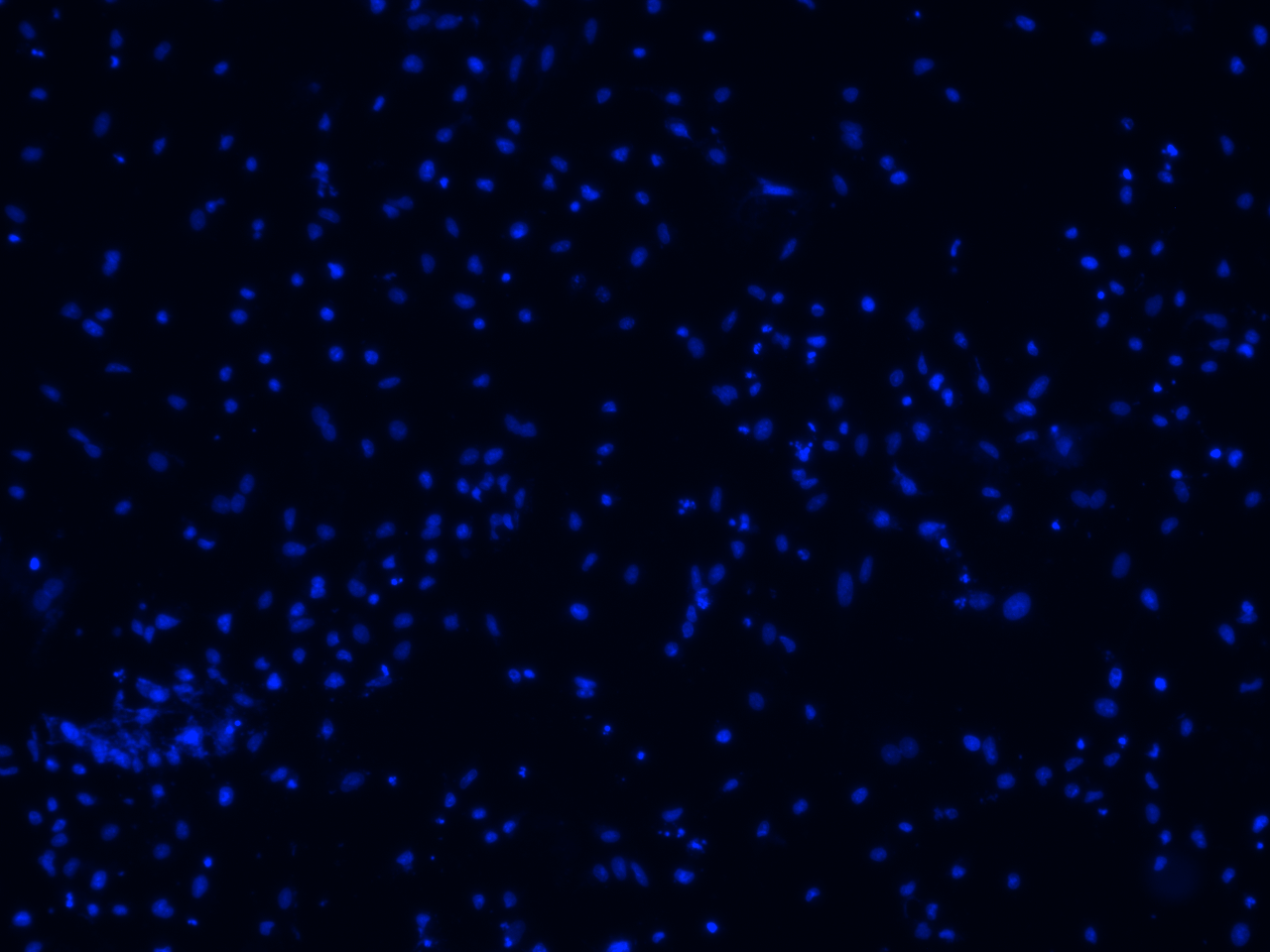

Supplement: Figure 2—figure supplement 1—source data 1. [file elife-73792-fig2-figsupp1-data1.zip › Figure 2-figure supplement 1-source data/1b/a549/e549 48_DAPI.tif]

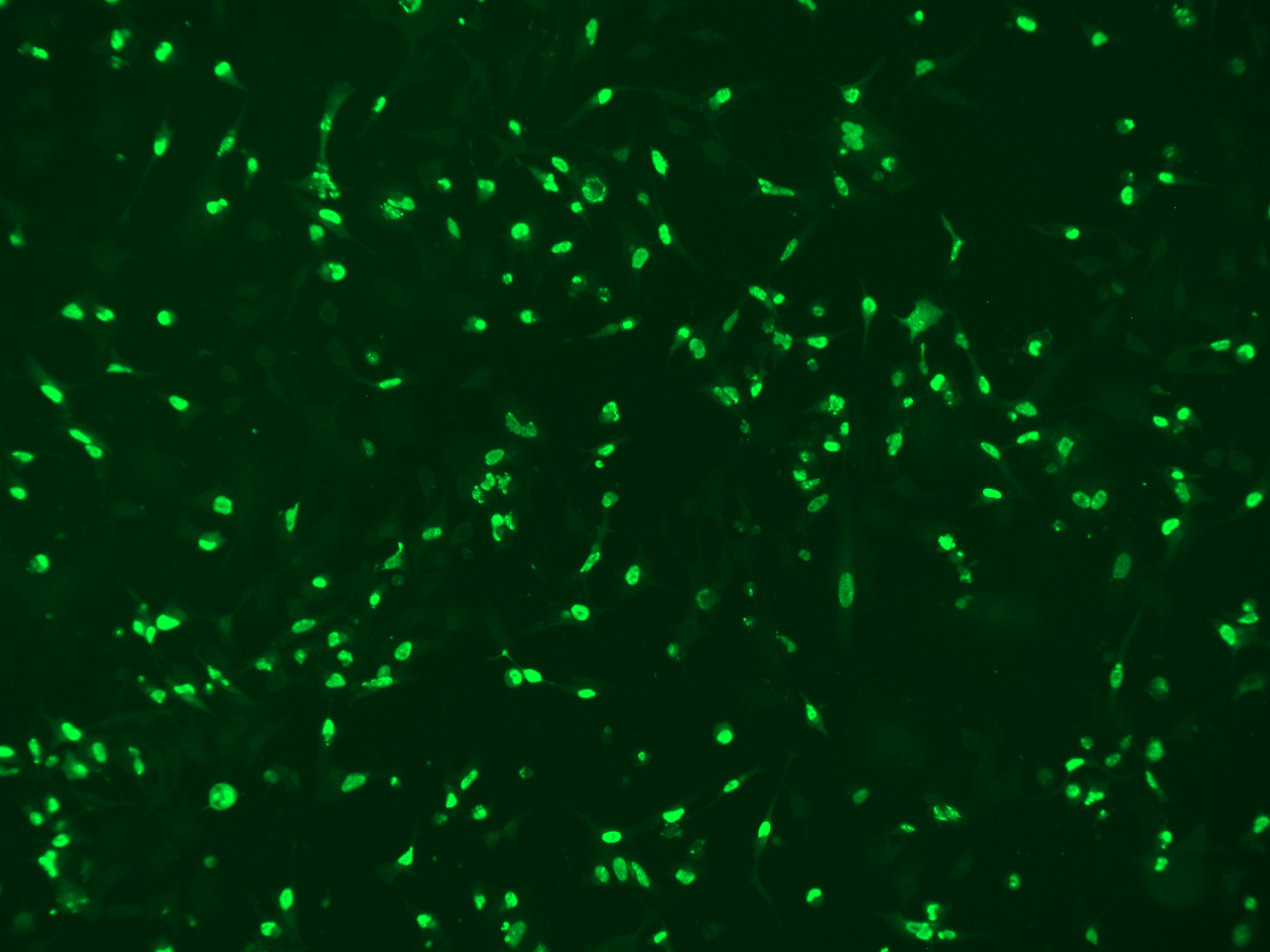

Supplement: Figure 2—figure supplement 1—source data 1. [file elife-73792-fig2-figsupp1-data1.zip › Figure 2-figure supplement 1-source data/1b/a549/e549 48_GFP.tif]

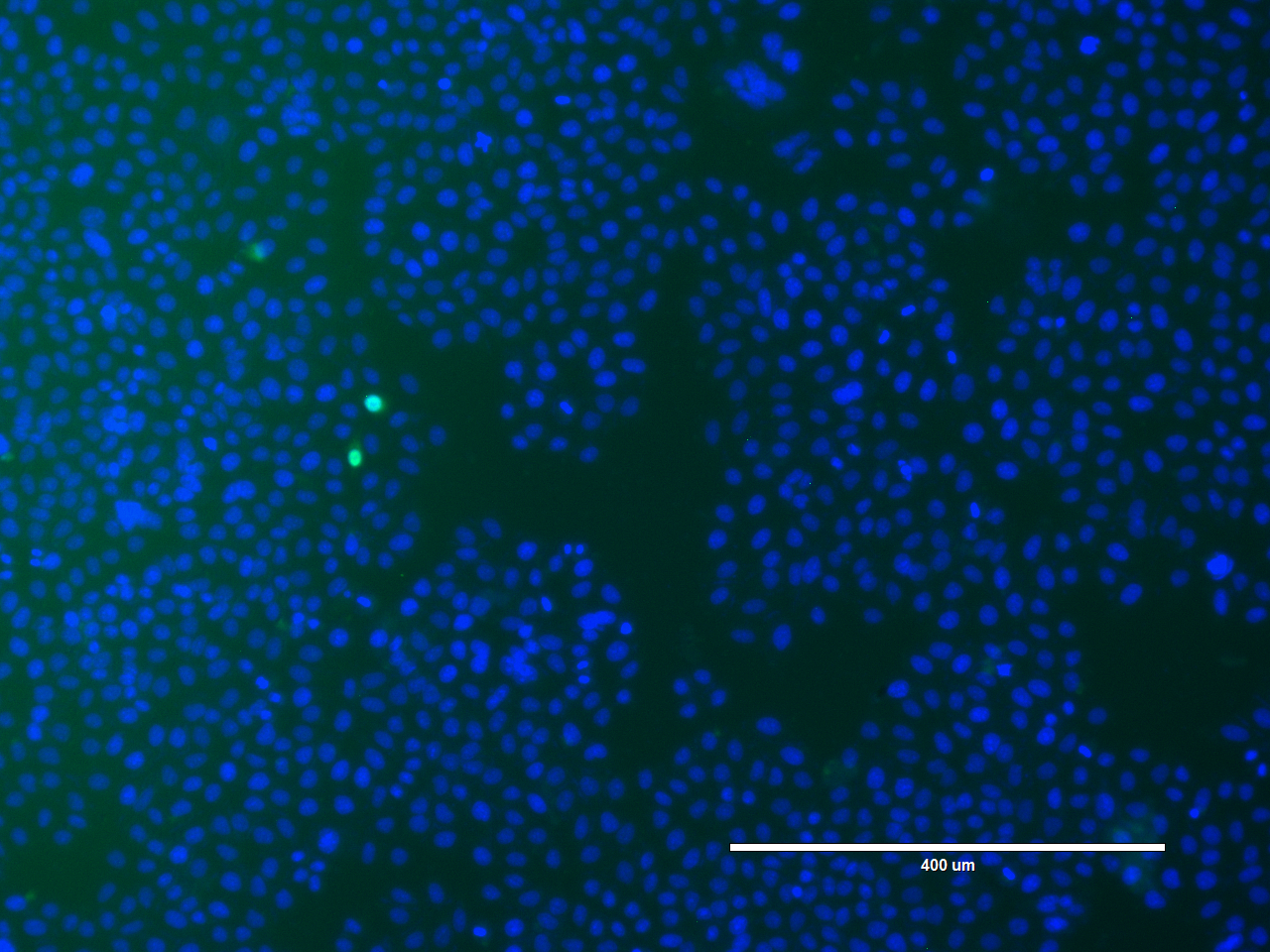

Supplement: Figure 2—figure supplement 1—source data 1. [file elife-73792-fig2-figsupp1-data1.zip › Figure 2-figure supplement 1-source data/1b/hela/hela 24h.tif]

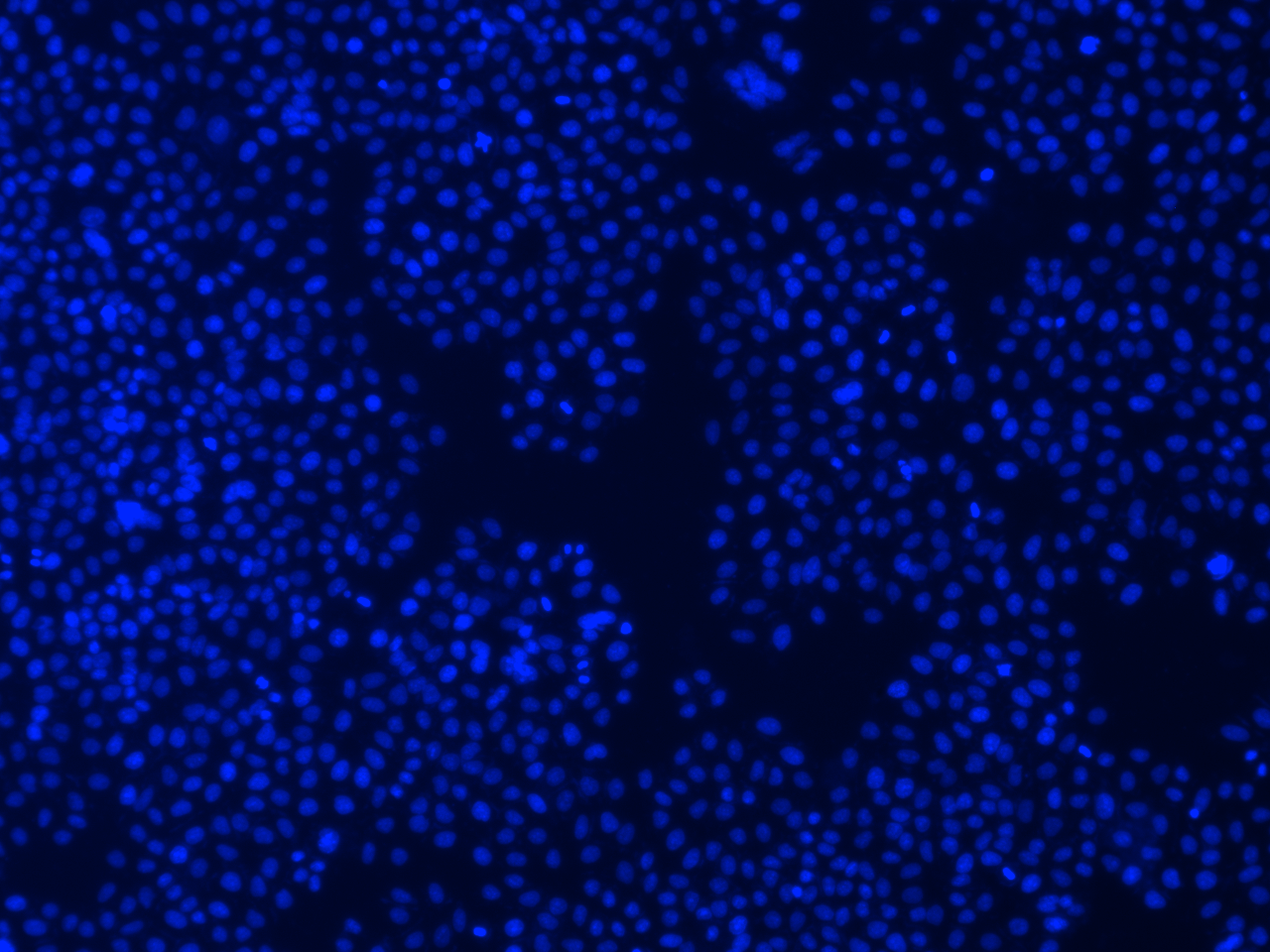

Supplement: Figure 2—figure supplement 1—source data 1. [file elife-73792-fig2-figsupp1-data1.zip › Figure 2-figure supplement 1-source data/1b/hela/hela 24h_DAPI.tif]

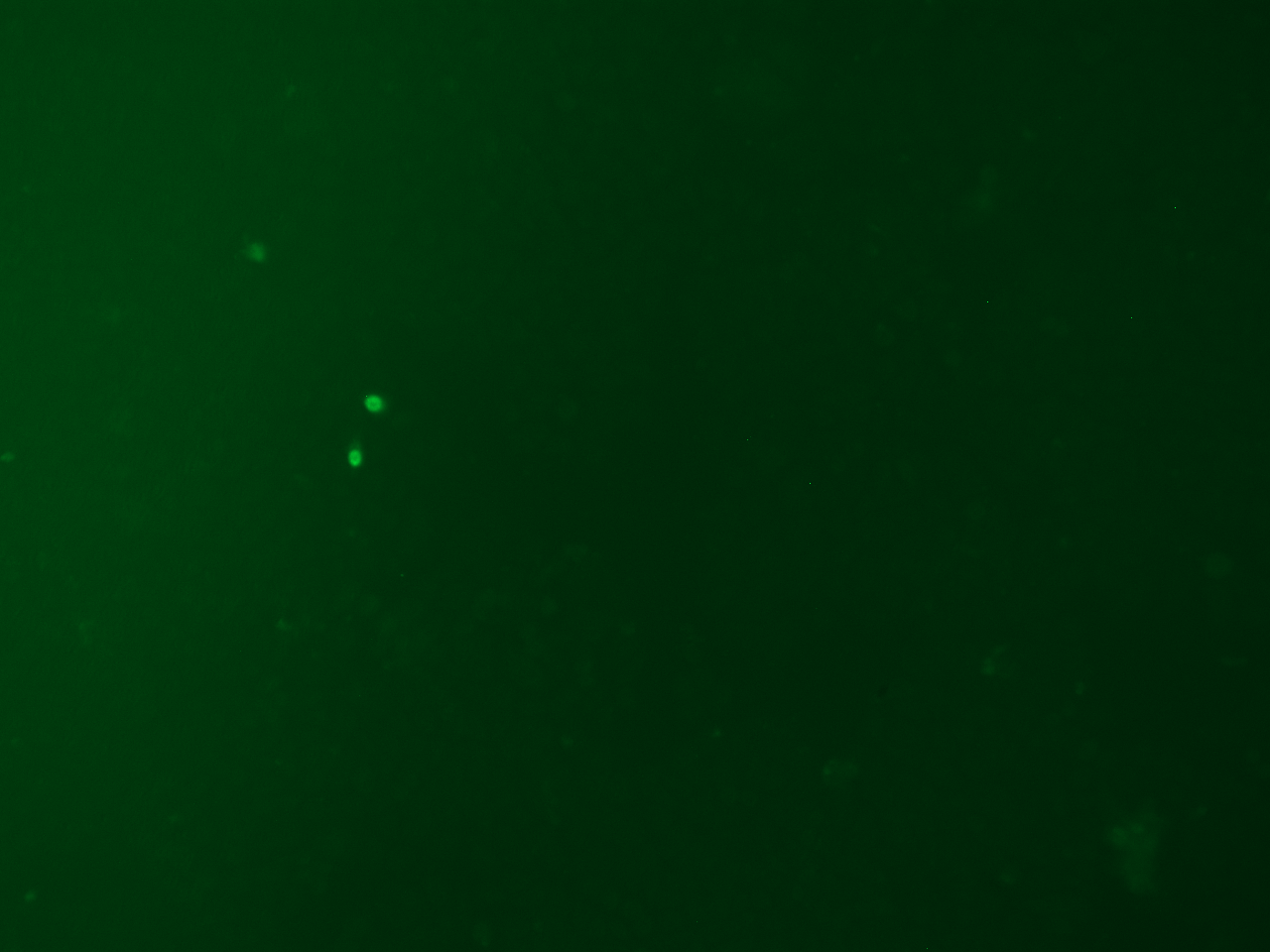

Supplement: Figure 2—figure supplement 1—source data 1. [file elife-73792-fig2-figsupp1-data1.zip › Figure 2-figure supplement 1-source data/1b/hela/hela 24h_GFP.tif]

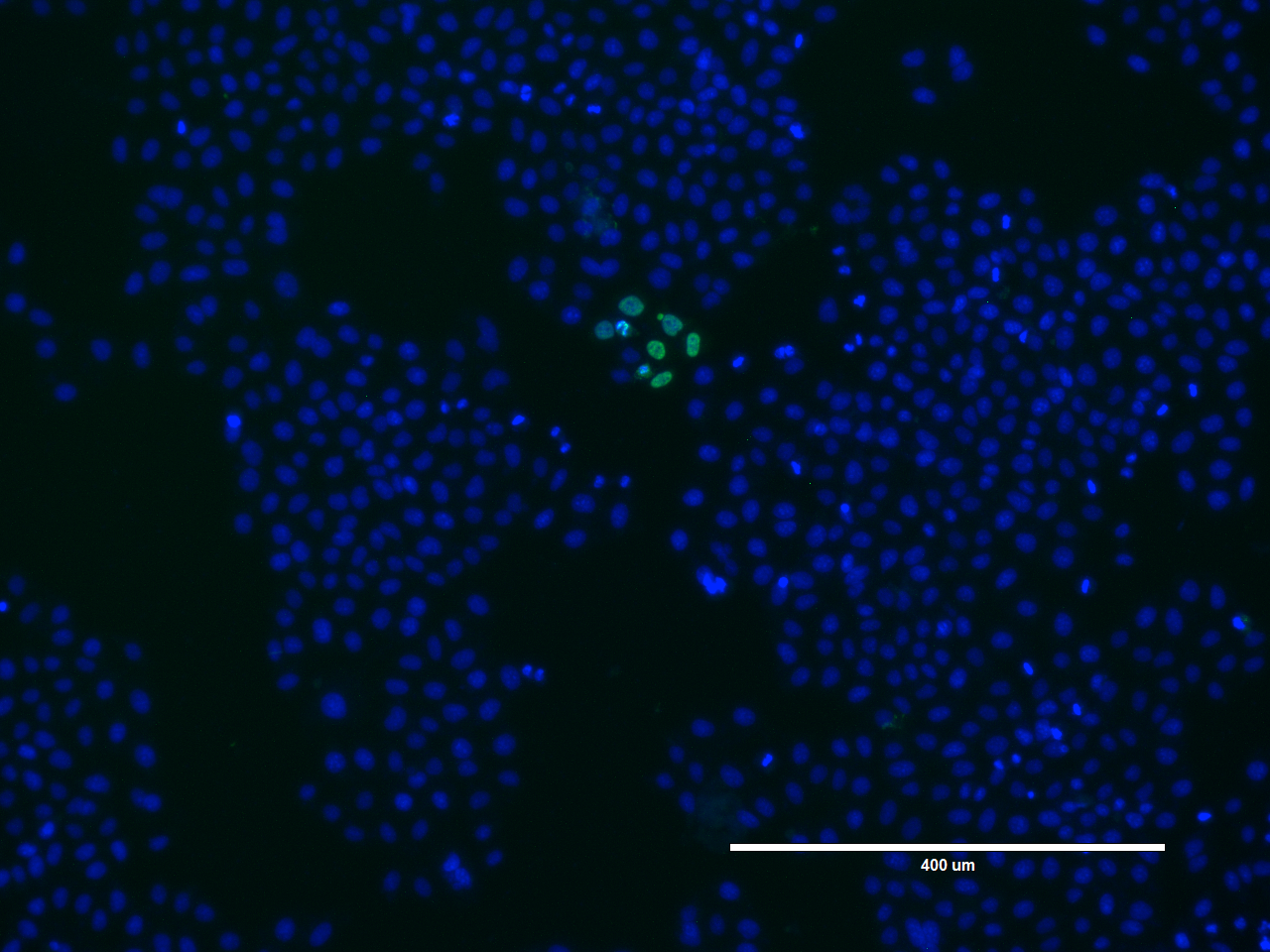

Supplement: Figure 2—figure supplement 1—source data 1. [file elife-73792-fig2-figsupp1-data1.zip › Figure 2-figure supplement 1-source data/1b/hela/hela 48.tif]

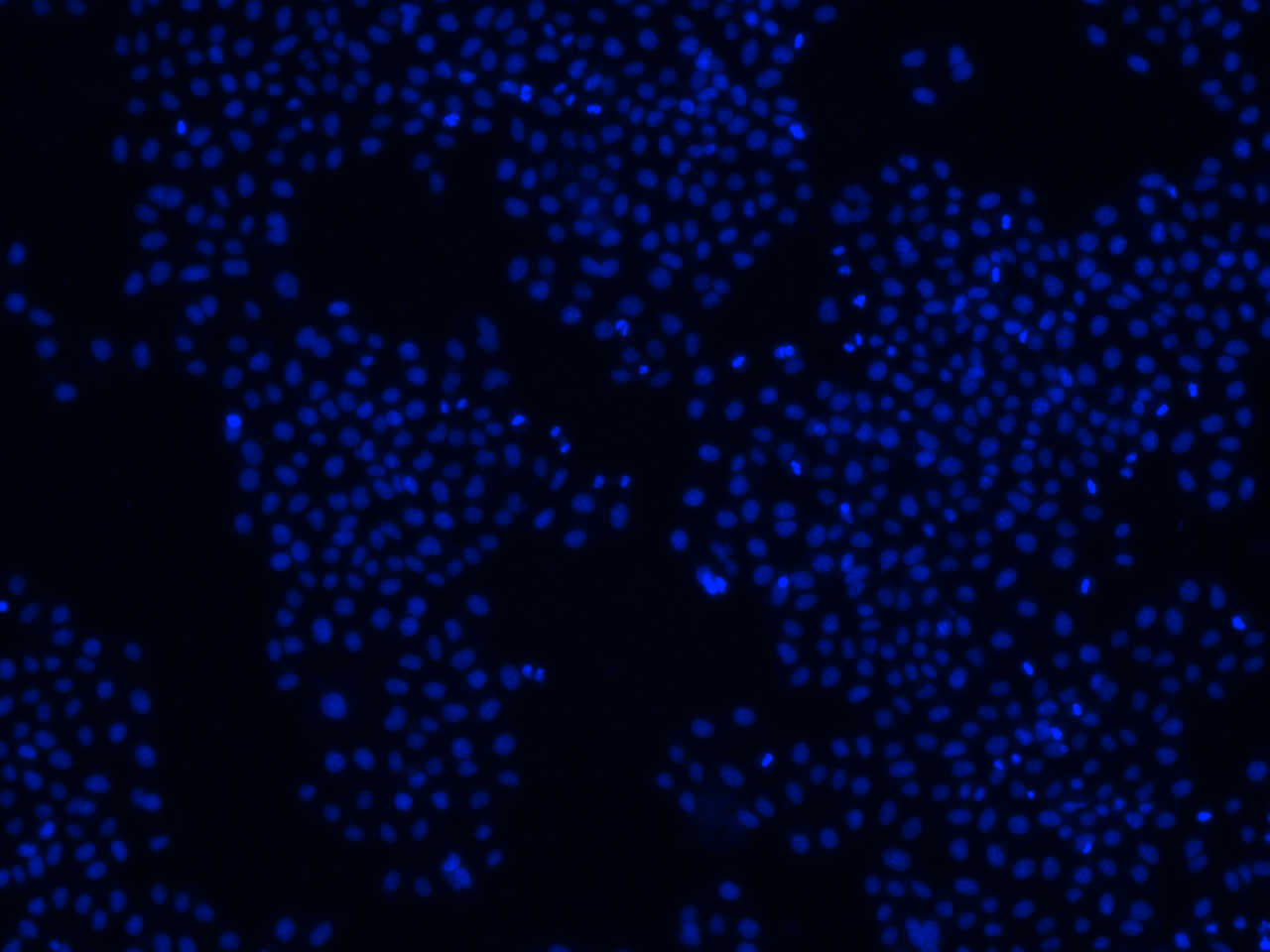

Supplement: Figure 2—figure supplement 1—source data 1. [file elife-73792-fig2-figsupp1-data1.zip › Figure 2-figure supplement 1-source data/1b/hela/hela 48_DAPI.tif]

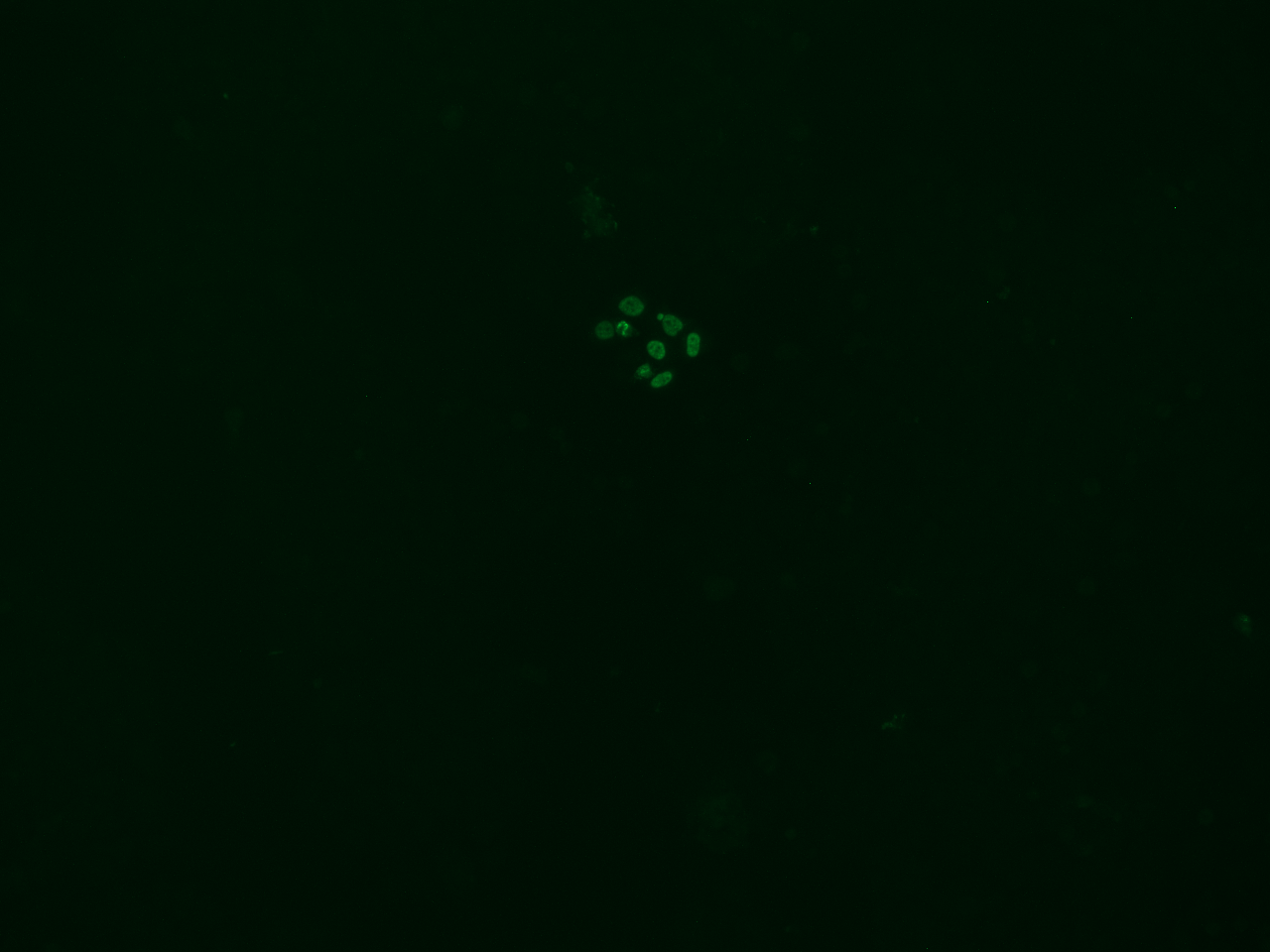

Supplement: Figure 2—figure supplement 1—source data 1. [file elife-73792-fig2-figsupp1-data1.zip › Figure 2-figure supplement 1-source data/1b/hela/hela 48_GFP.tif]

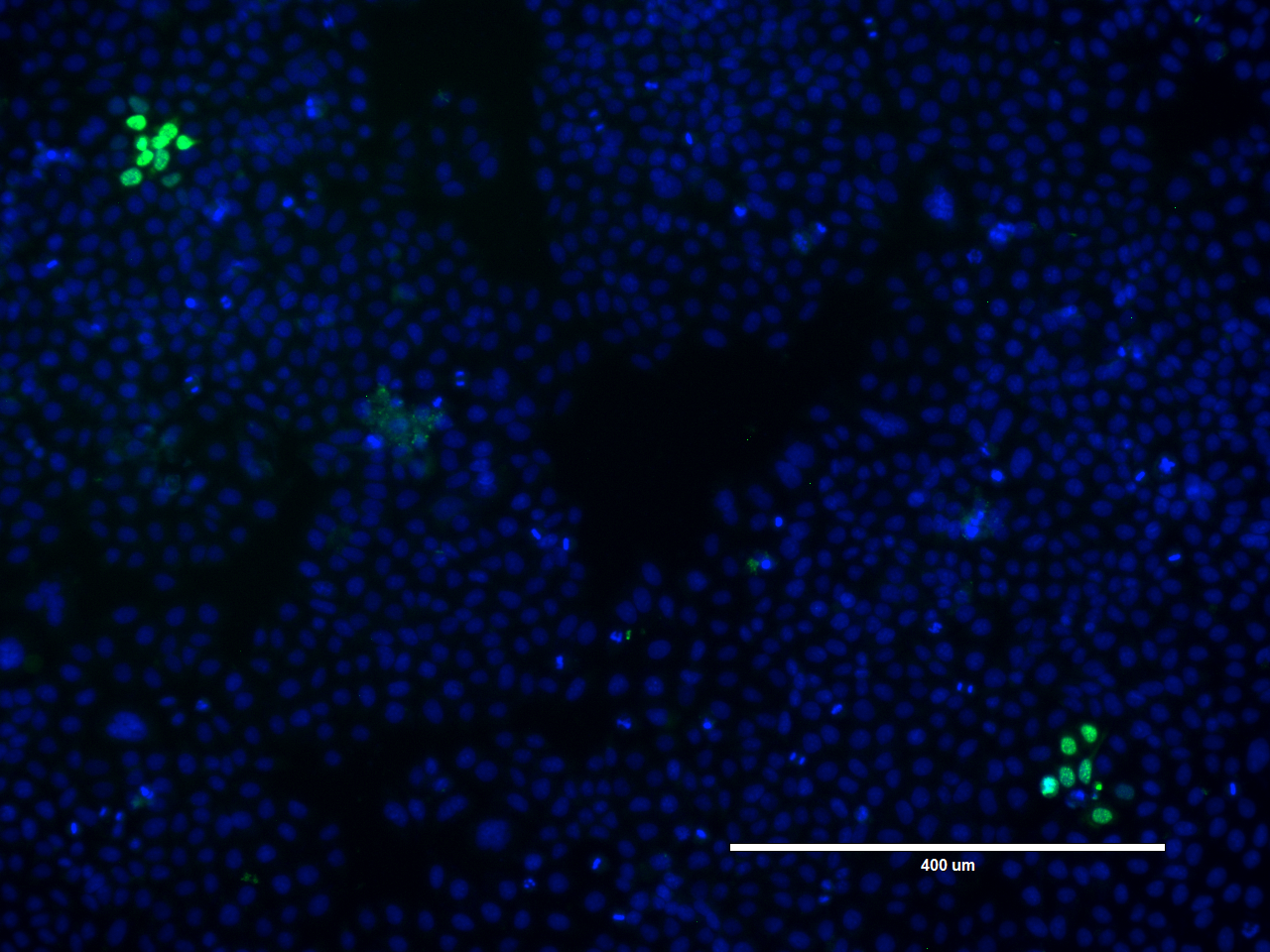

Supplement: Figure 2—figure supplement 1—source data 1. [file elife-73792-fig2-figsupp1-data1.zip › Figure 2-figure supplement 1-source data/1b/hela/hela 72.tif]

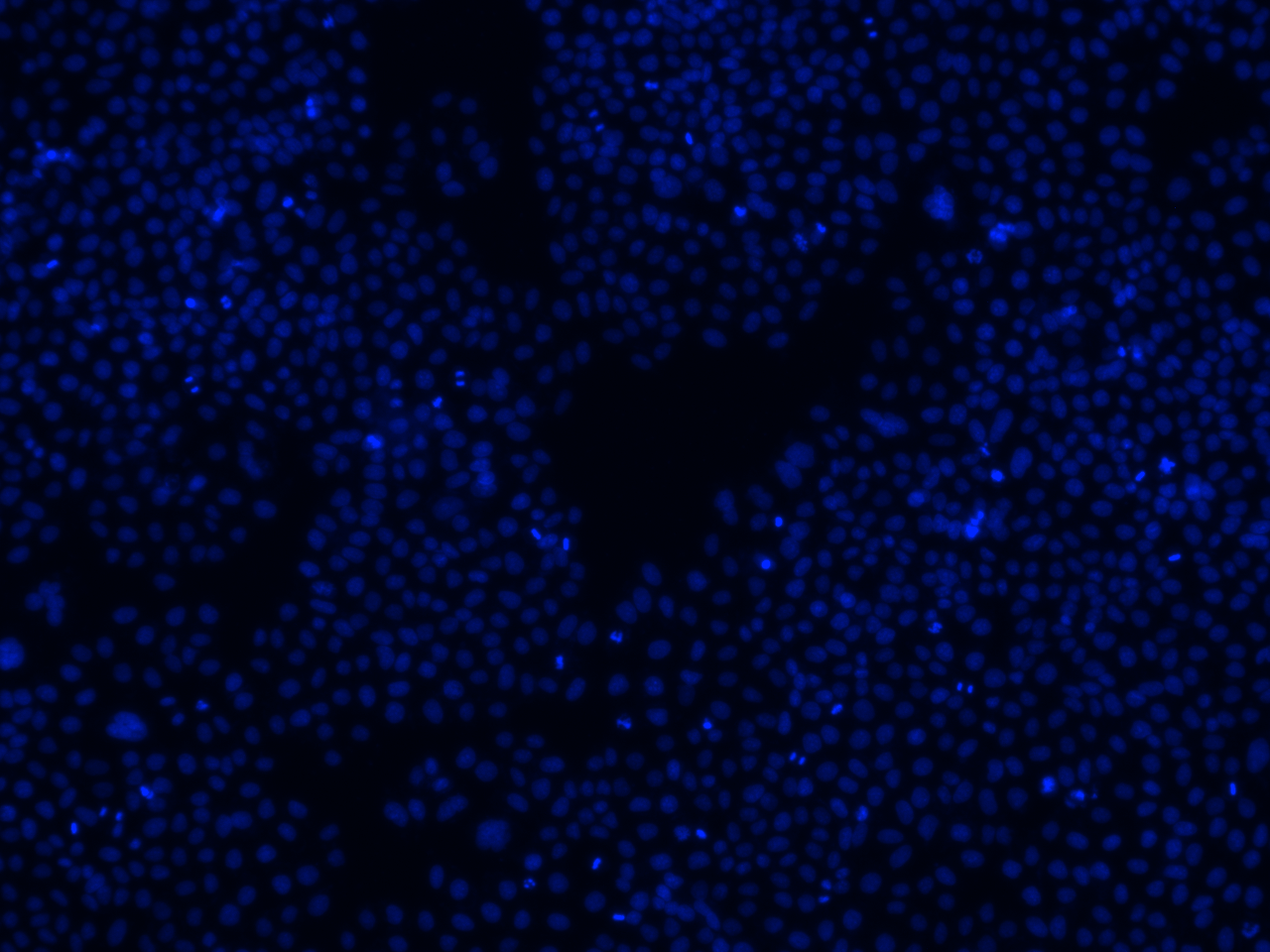

Supplement: Figure 2—figure supplement 1—source data 1. [file elife-73792-fig2-figsupp1-data1.zip › Figure 2-figure supplement 1-source data/1b/hela/hela 72_DAPI.tif]

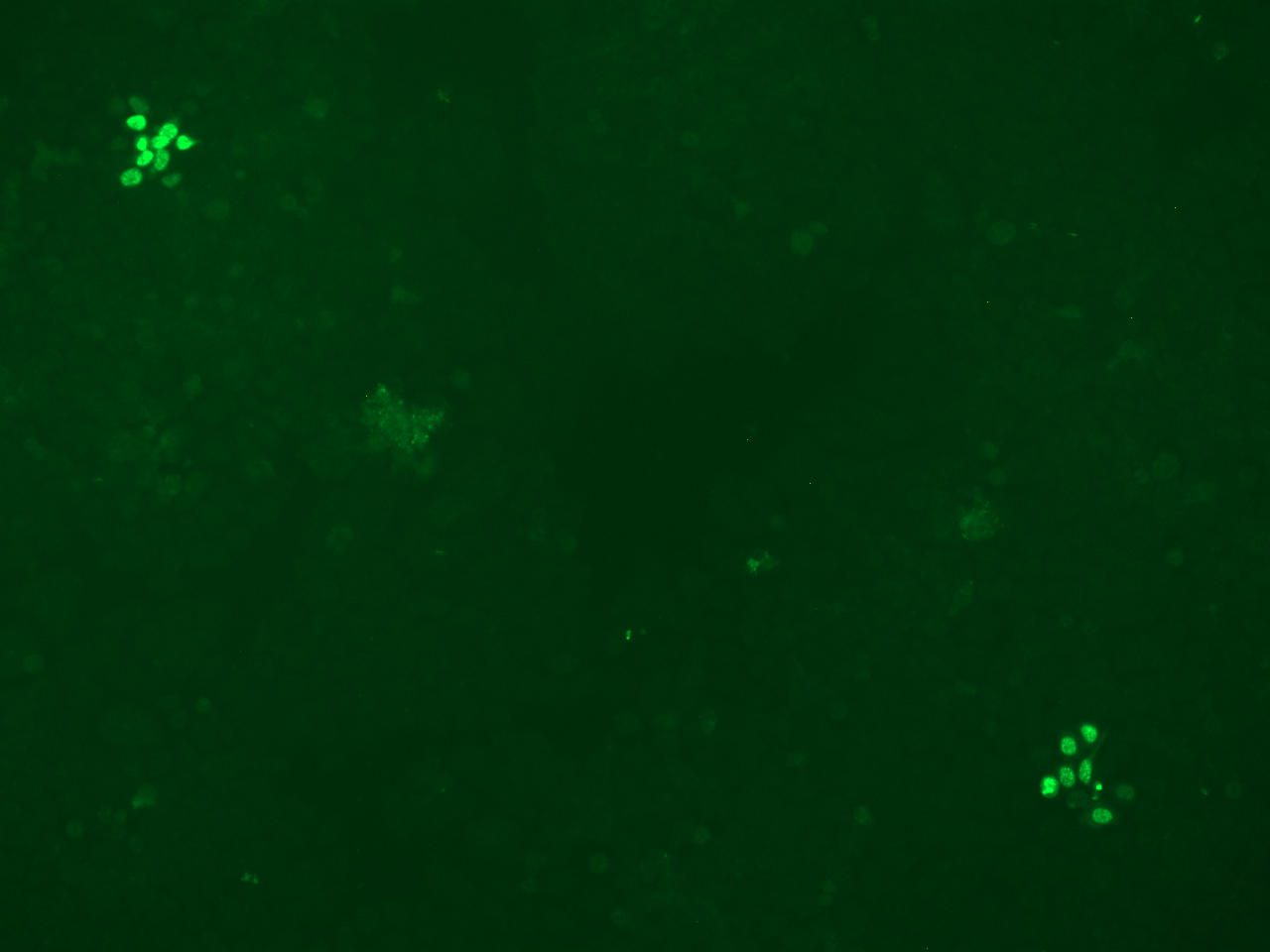

Supplement: Figure 2—figure supplement 1—source data 1. [file elife-73792-fig2-figsupp1-data1.zip › Figure 2-figure supplement 1-source data/1b/hela/hela 72_GFP.tif]

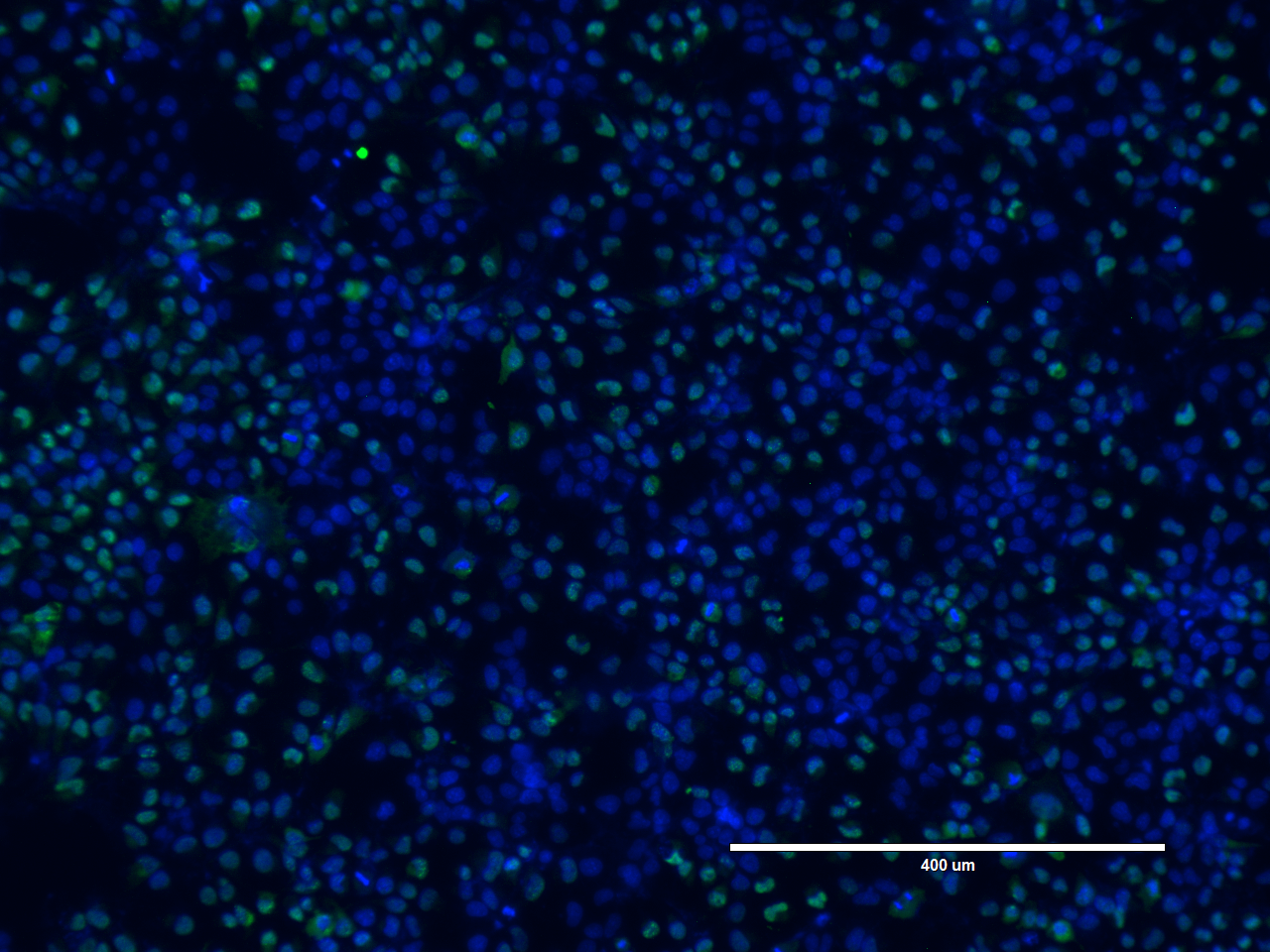

Supplement: Figure 2—figure supplement 1—source data 1. [file elife-73792-fig2-figsupp1-data1.zip › Figure 2-figure supplement 1-source data/1b/huh7/huh 24.tif]

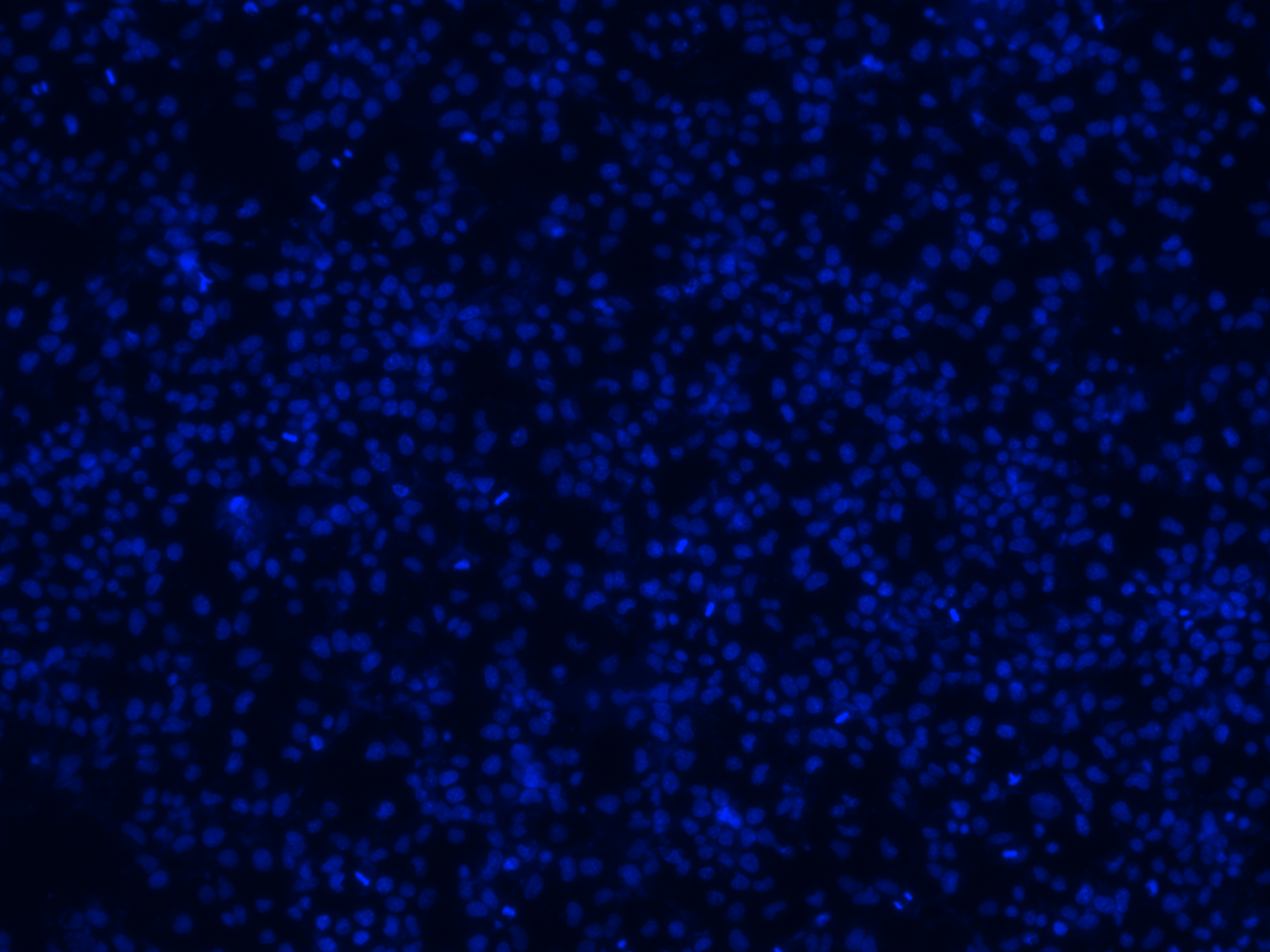

Supplement: Figure 2—figure supplement 1—source data 1. [file elife-73792-fig2-figsupp1-data1.zip › Figure 2-figure supplement 1-source data/1b/huh7/huh 24_DAPI.tif]
