# Supplementary figures and images for: Zika virus causes placental pyroptosis and associated adverse fetal outcomes by activating GSDME (part 2 of 4)
Source: eLife. 2022 Aug 16;11:e73792. doi: 10.7554/eLife.73792 (PMC9381041; doi:10.7554/eLife.73792)

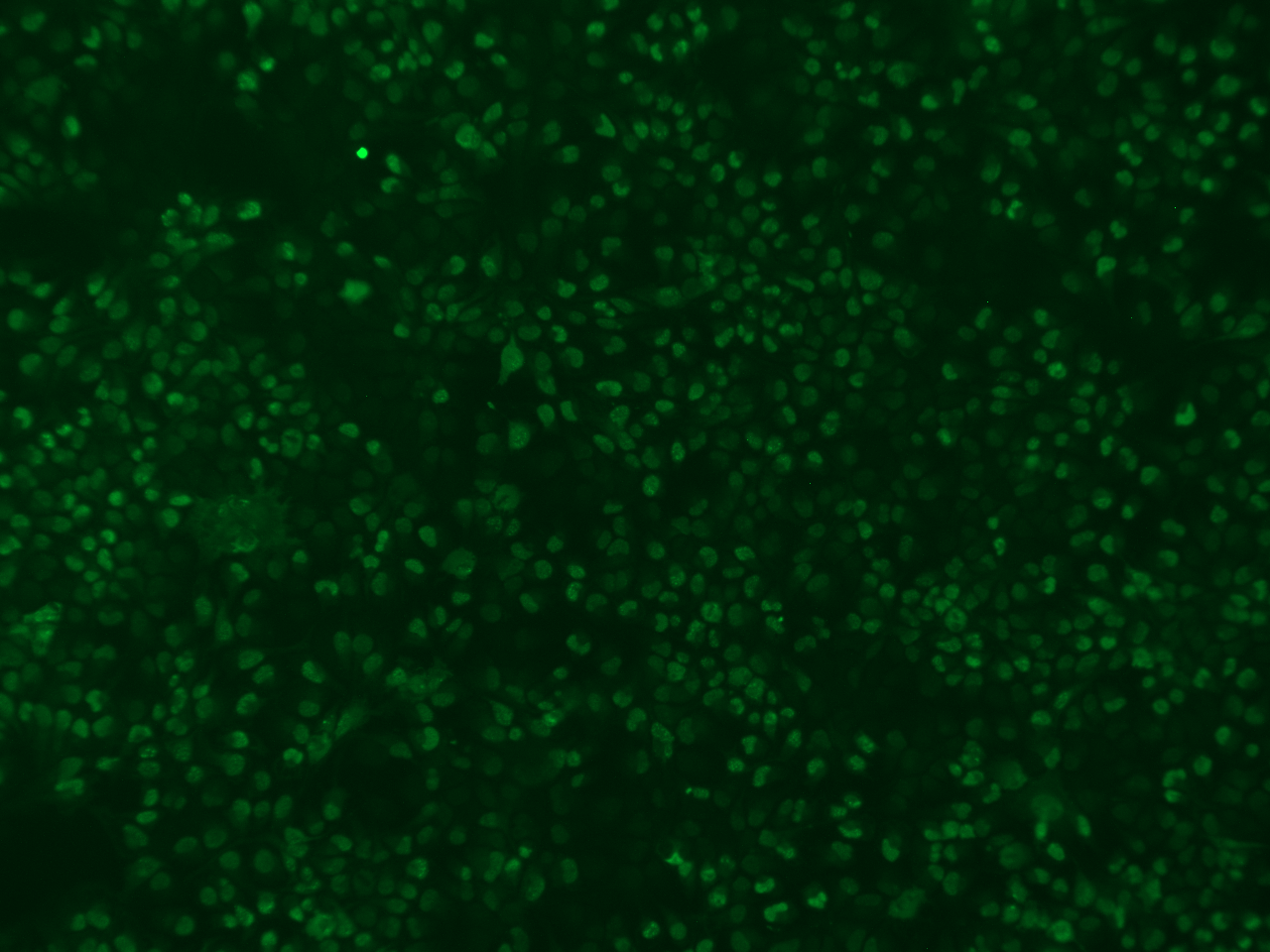

Supplement: Figure 2—figure supplement 1—source data 1. [file elife-73792-fig2-figsupp1-data1.zip › Figure 2-figure supplement 1-source data/1b/huh7/huh 24_GFP.tif]

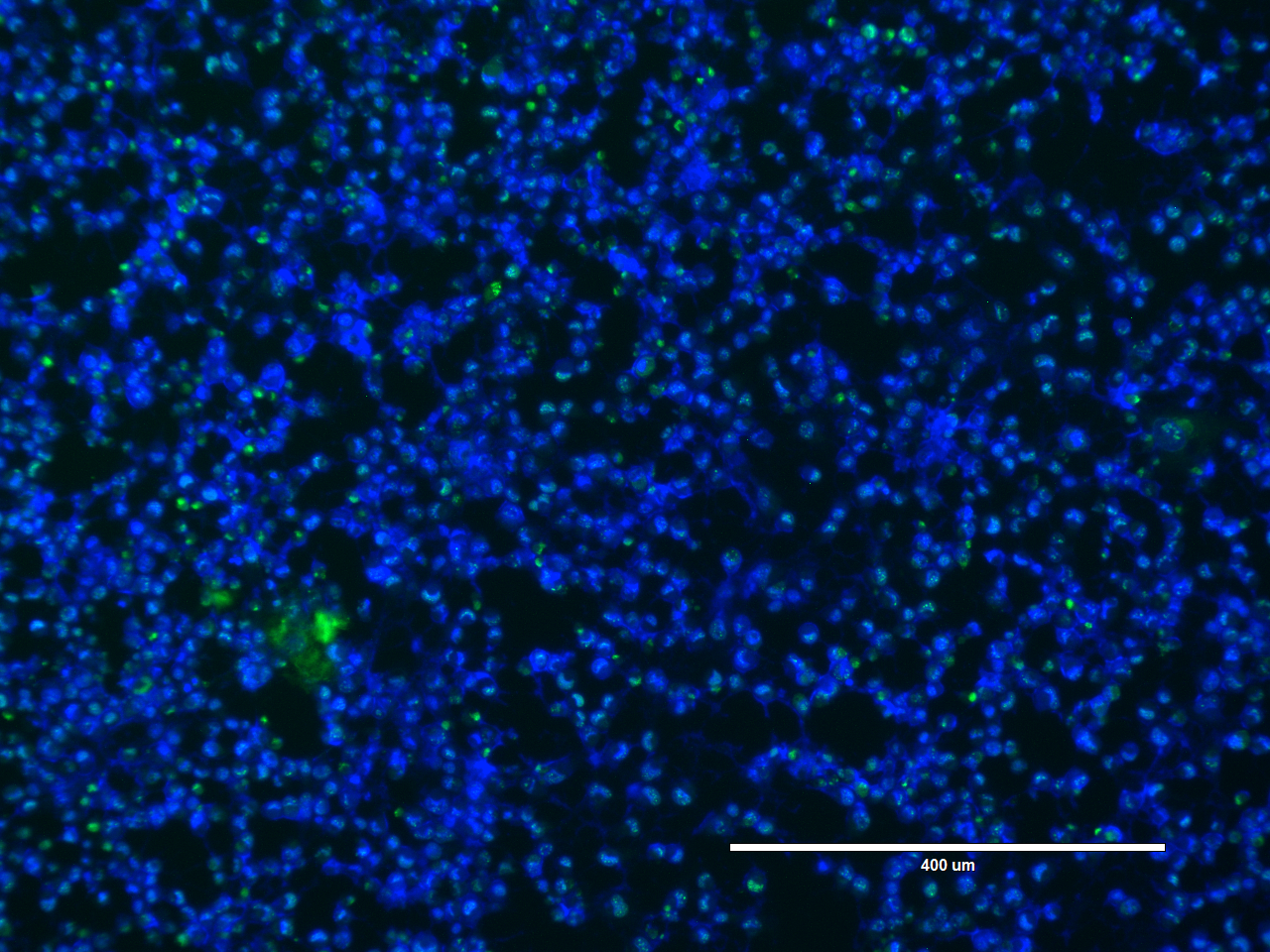

Supplement: Figure 2—figure supplement 1—source data 1. [file elife-73792-fig2-figsupp1-data1.zip › Figure 2-figure supplement 1-source data/1b/huh7/huh 72.tif]

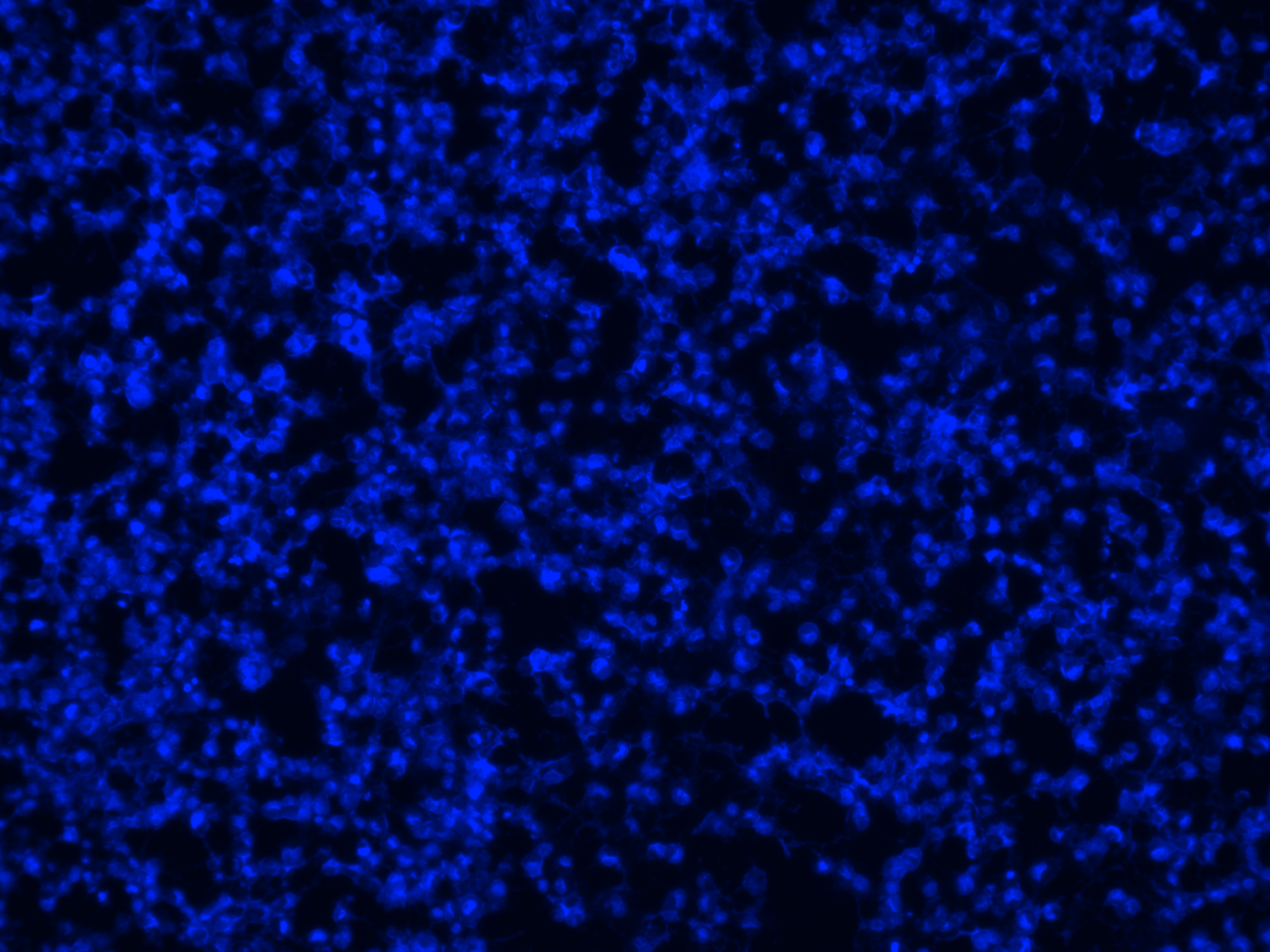

Supplement: Figure 2—figure supplement 1—source data 1. [file elife-73792-fig2-figsupp1-data1.zip › Figure 2-figure supplement 1-source data/1b/huh7/huh 72_DAPI.tif]

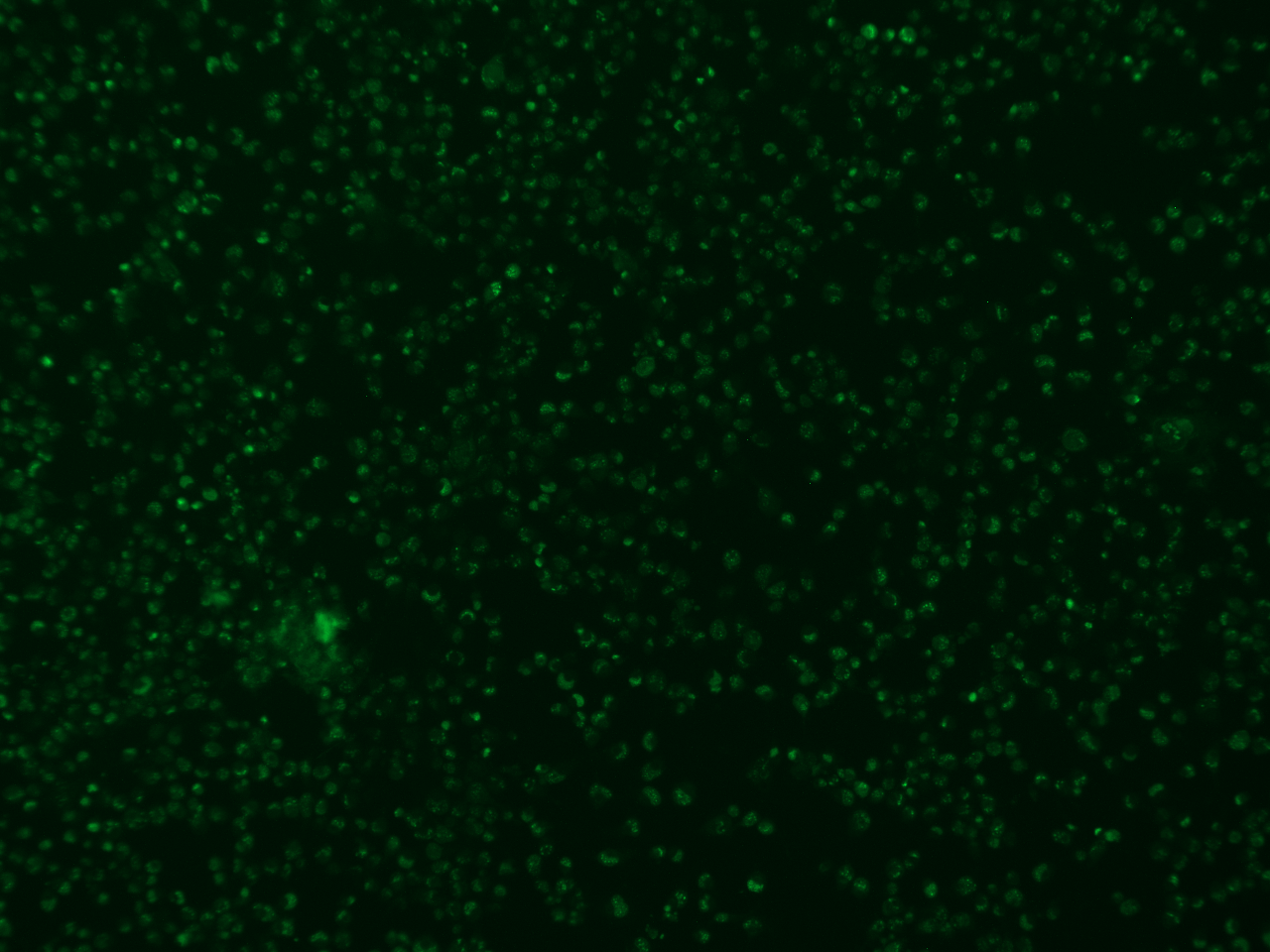

Supplement: Figure 2—figure supplement 1—source data 1. [file elife-73792-fig2-figsupp1-data1.zip › Figure 2-figure supplement 1-source data/1b/huh7/huh 72_GFP.tif]

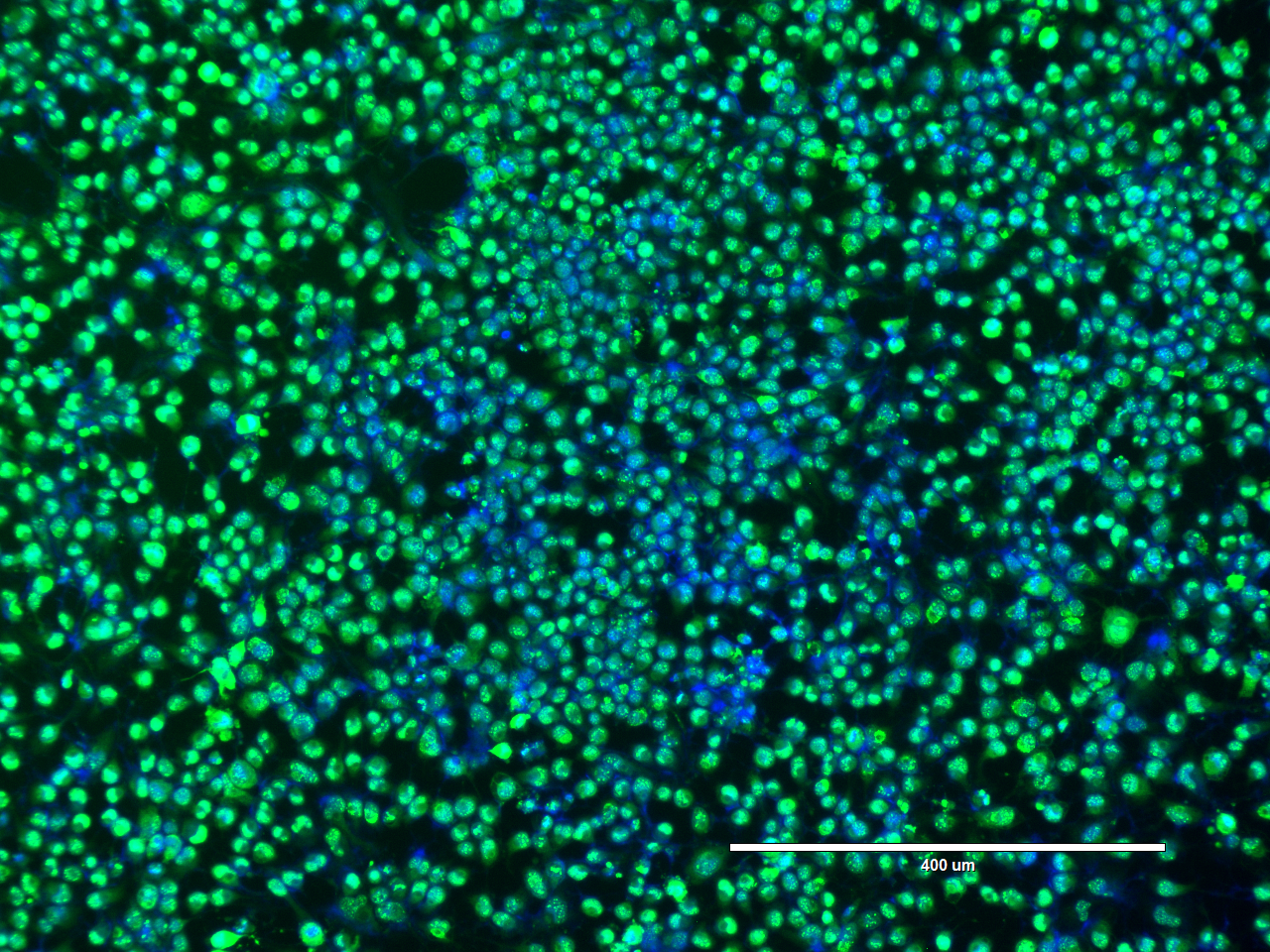

Supplement: Figure 2—figure supplement 1—source data 1. [file elife-73792-fig2-figsupp1-data1.zip › Figure 2-figure supplement 1-source data/1b/huh7/huh7 48.tif]

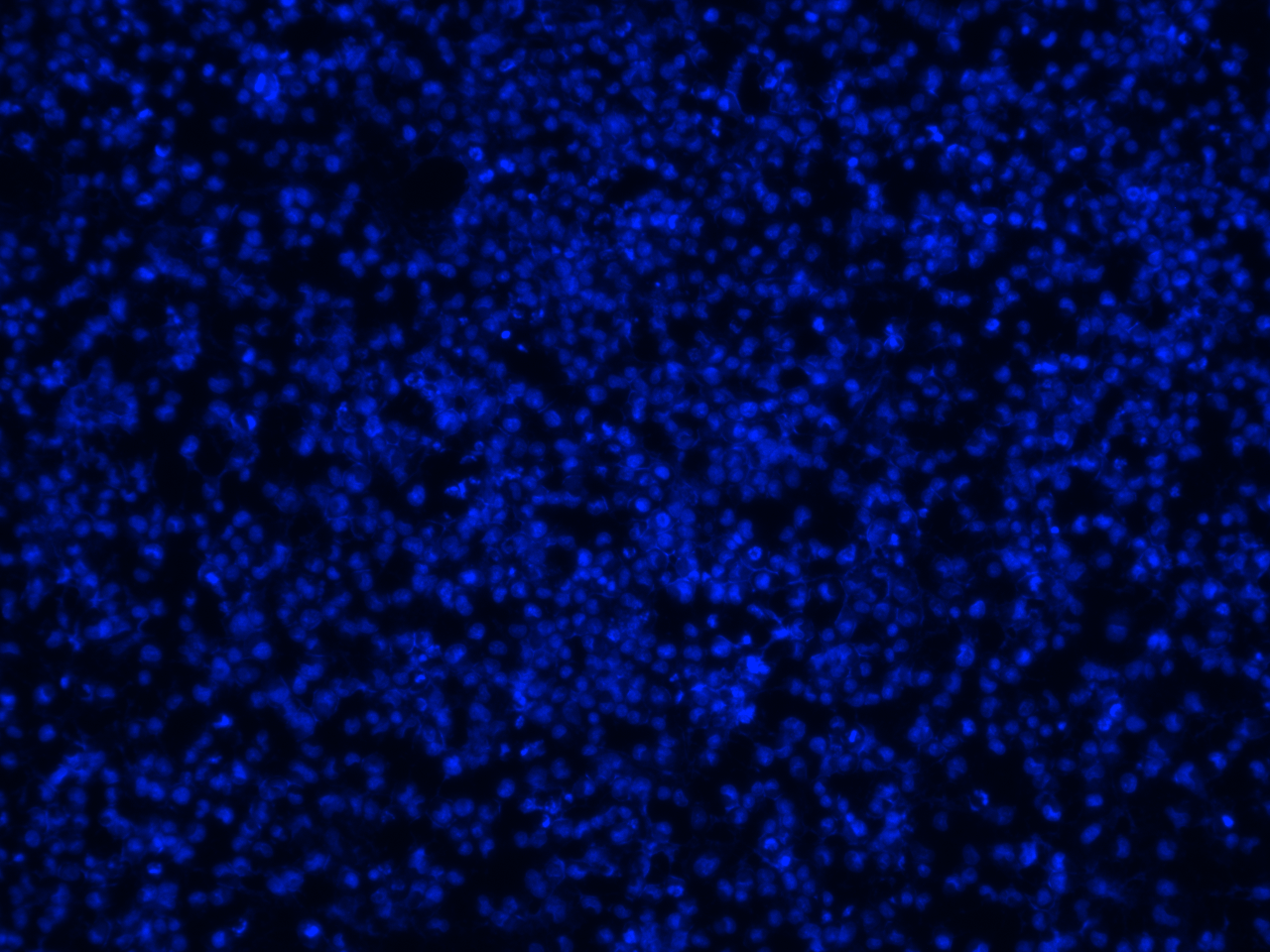

Supplement: Figure 2—figure supplement 1—source data 1. [file elife-73792-fig2-figsupp1-data1.zip › Figure 2-figure supplement 1-source data/1b/huh7/huh7 48_DAPI.tif]

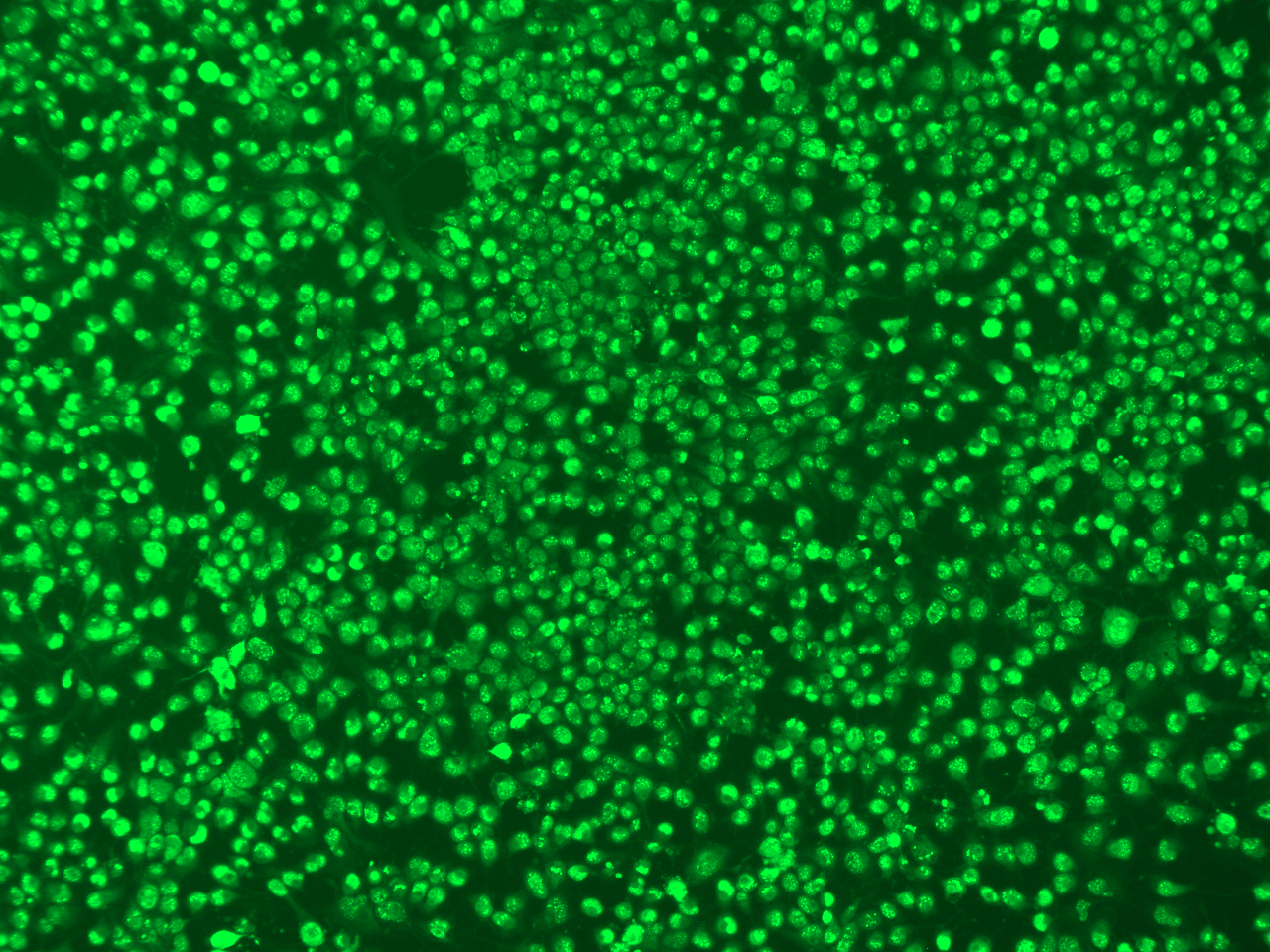

Supplement: Figure 2—figure supplement 1—source data 1. [file elife-73792-fig2-figsupp1-data1.zip › Figure 2-figure supplement 1-source data/1b/huh7/huh7 48_GFP.tif]

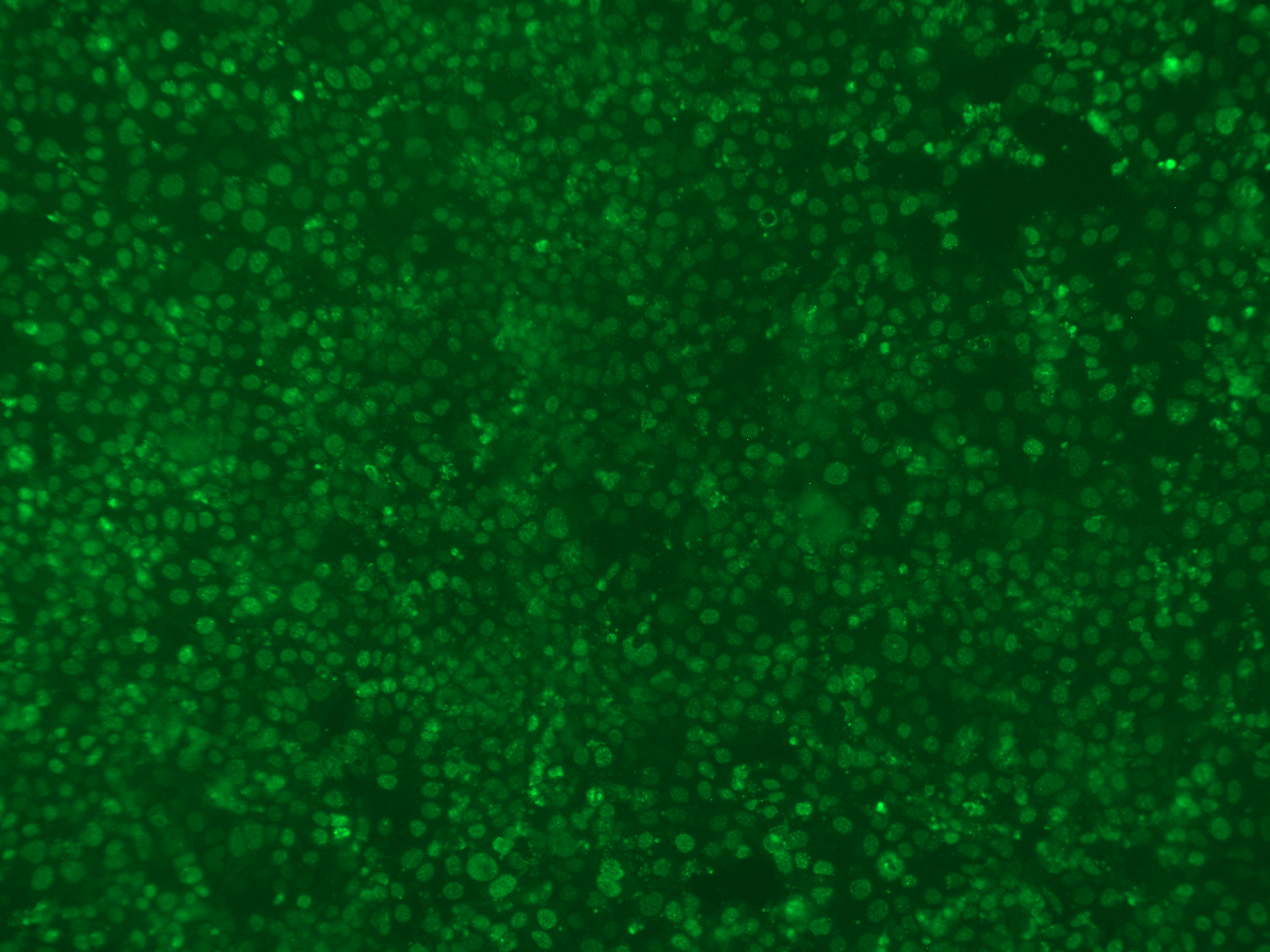

Supplement: Figure 2—figure supplement 1—source data 1. [file elife-73792-fig2-figsupp1-data1.zip › Figure 2-figure supplement 1-source data/1b/jeg3/jeg3 24 GFP.tif]

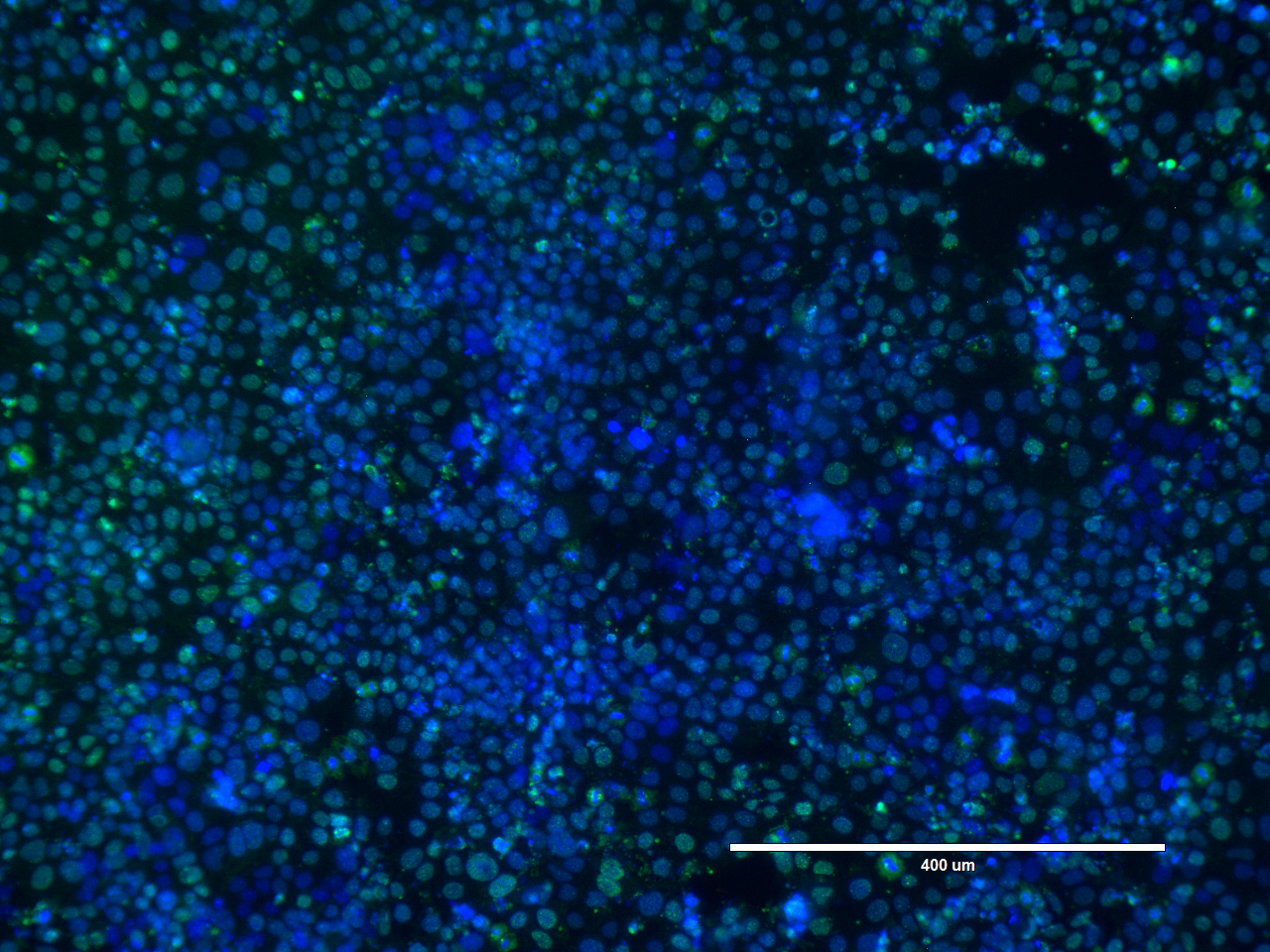

Supplement: Figure 2—figure supplement 1—source data 1. [file elife-73792-fig2-figsupp1-data1.zip › Figure 2-figure supplement 1-source data/1b/jeg3/jeg3 24.tif]

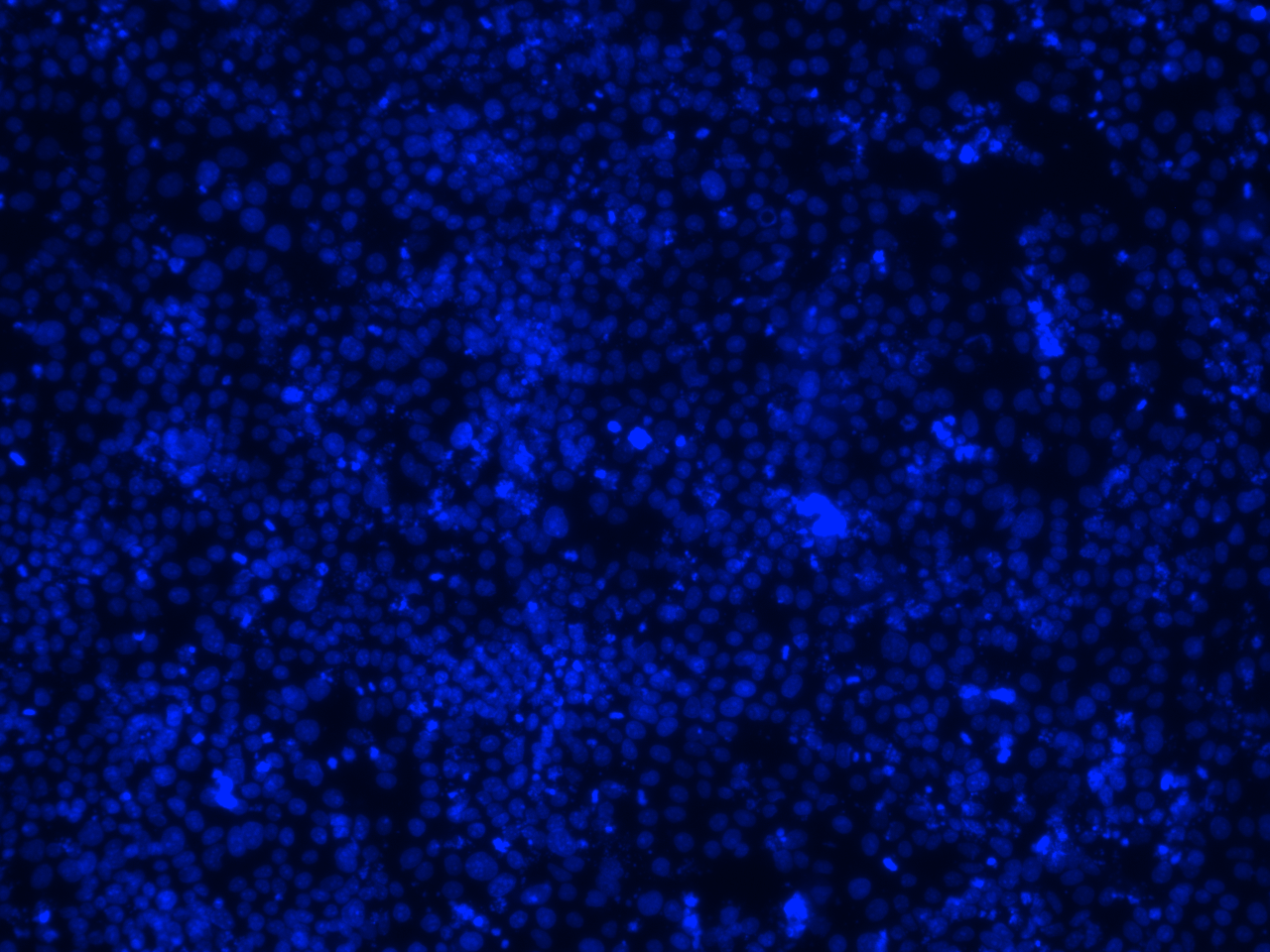

Supplement: Figure 2—figure supplement 1—source data 1. [file elife-73792-fig2-figsupp1-data1.zip › Figure 2-figure supplement 1-source data/1b/jeg3/jeg3 24_DAPI.tif]

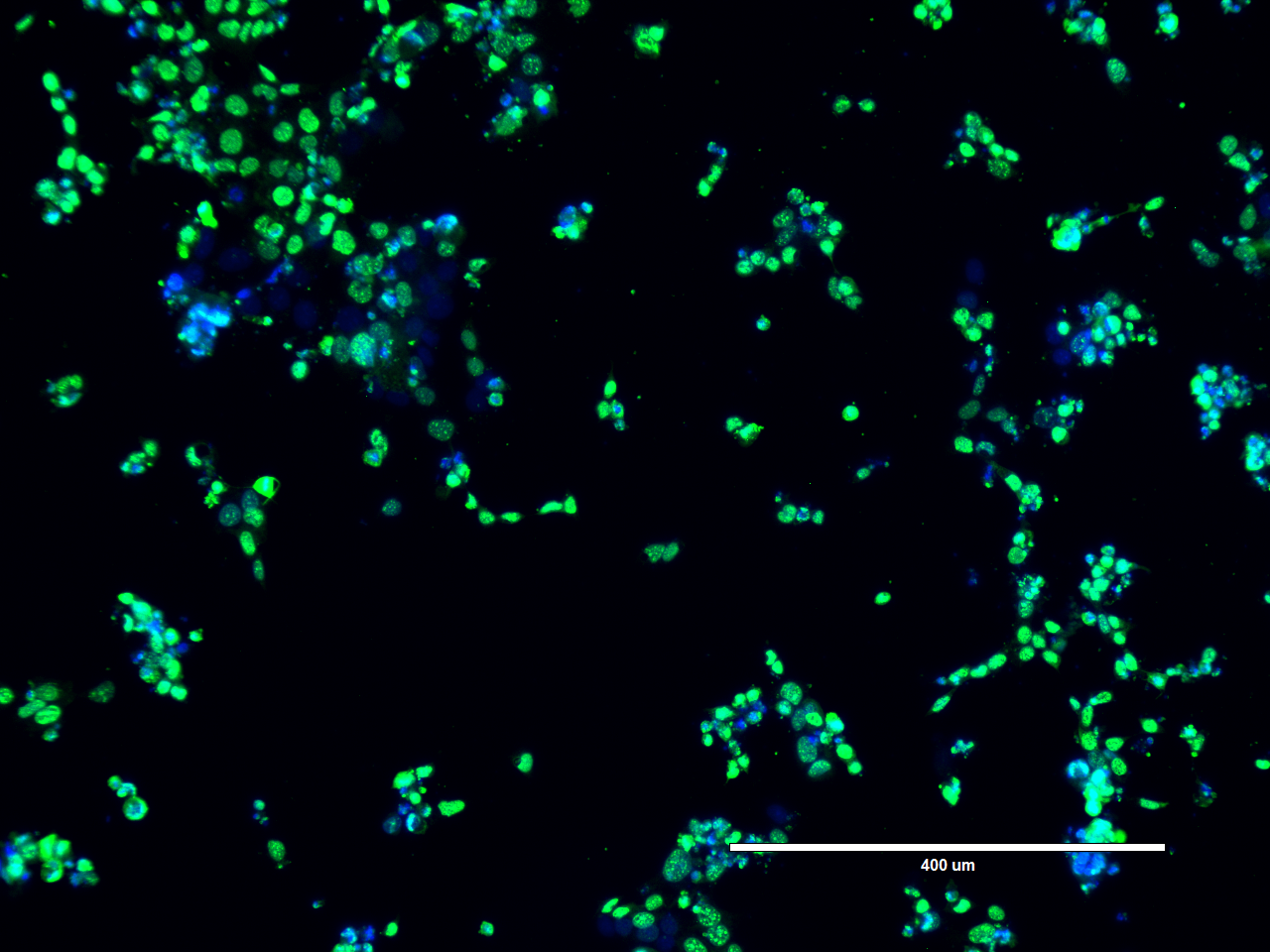

Supplement: Figure 2—figure supplement 1—source data 1. [file elife-73792-fig2-figsupp1-data1.zip › Figure 2-figure supplement 1-source data/1b/jeg3/jeg3 48.tif]

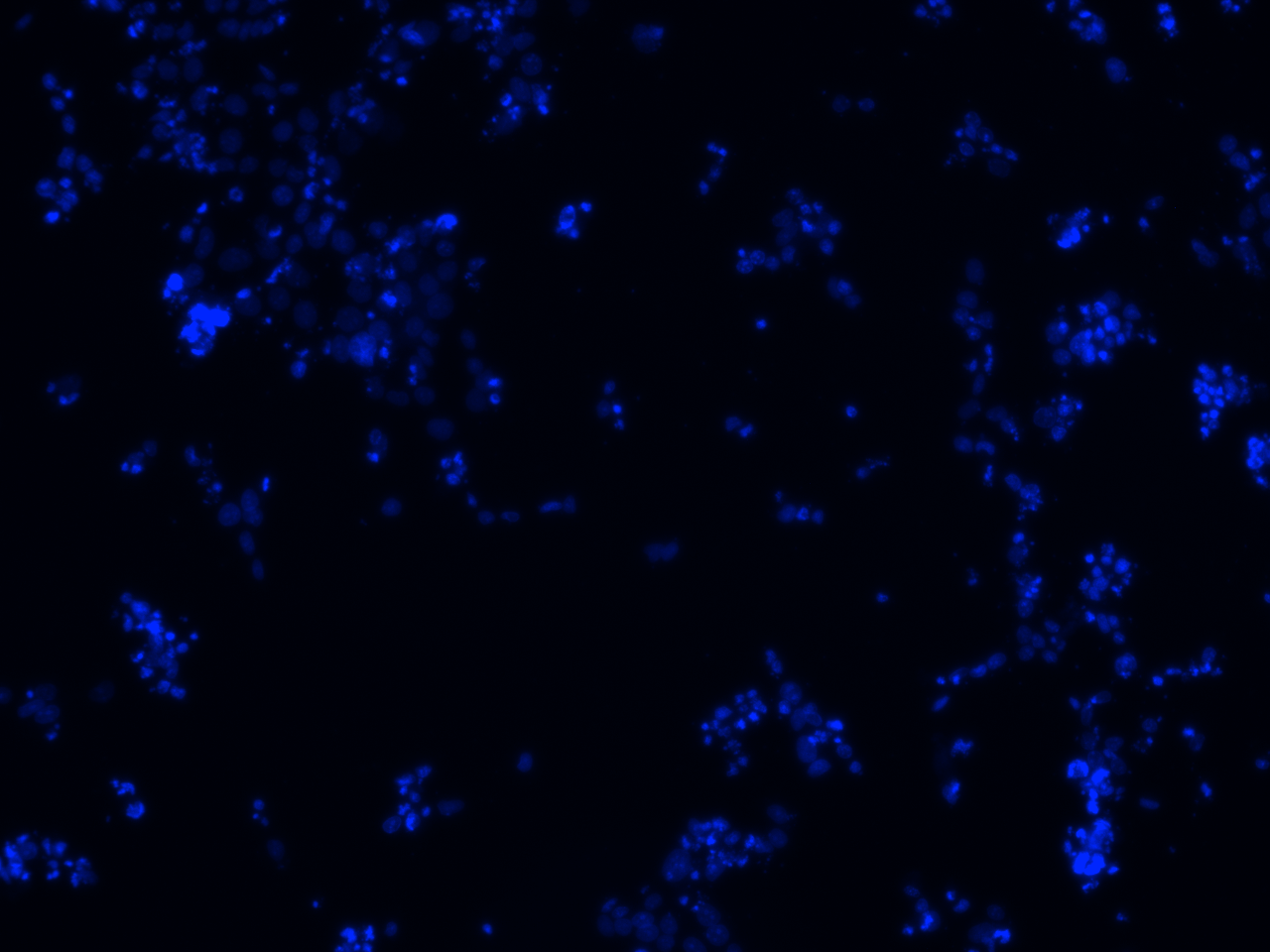

Supplement: Figure 2—figure supplement 1—source data 1. [file elife-73792-fig2-figsupp1-data1.zip › Figure 2-figure supplement 1-source data/1b/jeg3/jeg3 48_DAPI.tif]

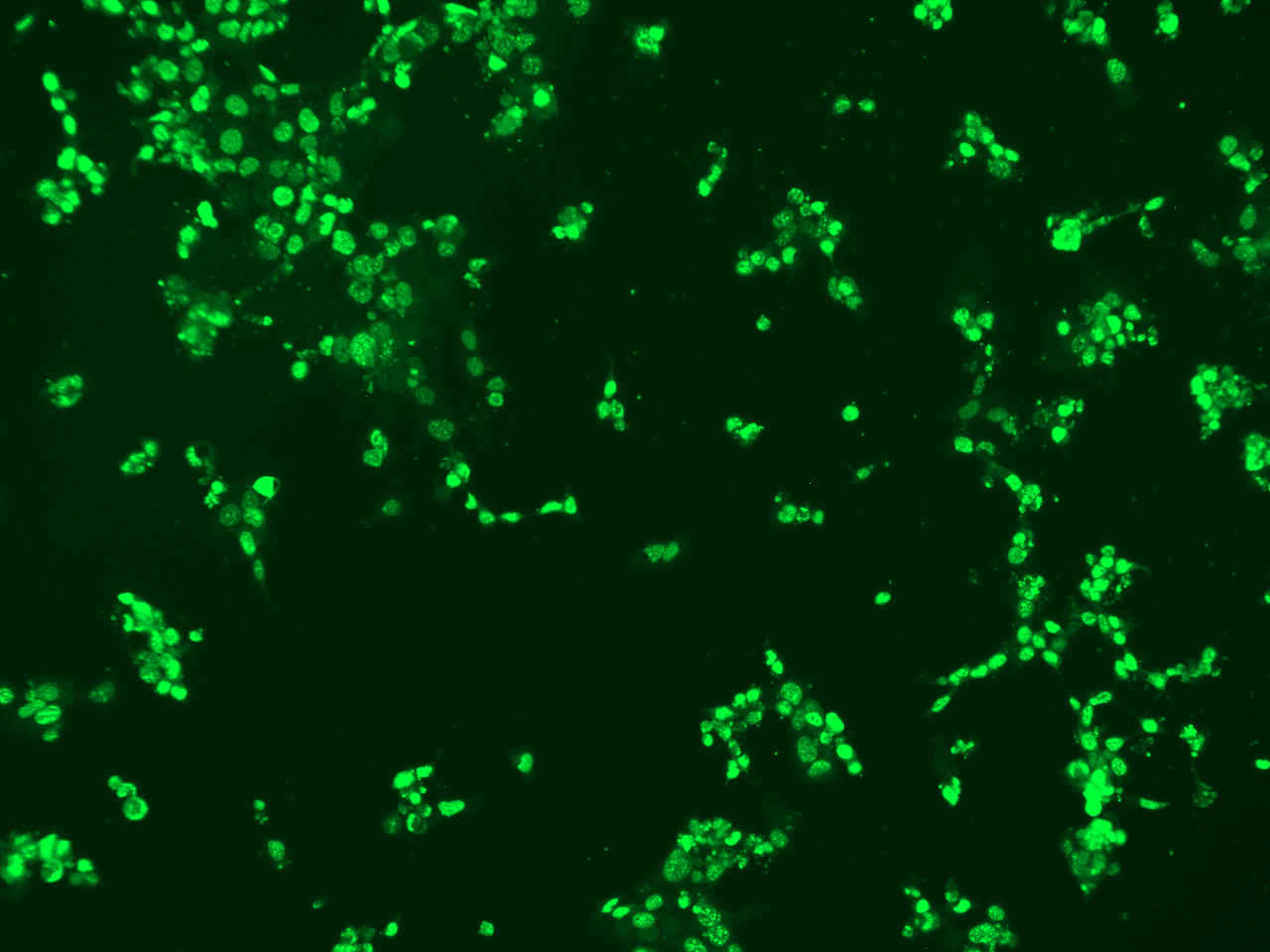

Supplement: Figure 2—figure supplement 1—source data 1. [file elife-73792-fig2-figsupp1-data1.zip › Figure 2-figure supplement 1-source data/1b/jeg3/jeg3 48_GFP.tif]

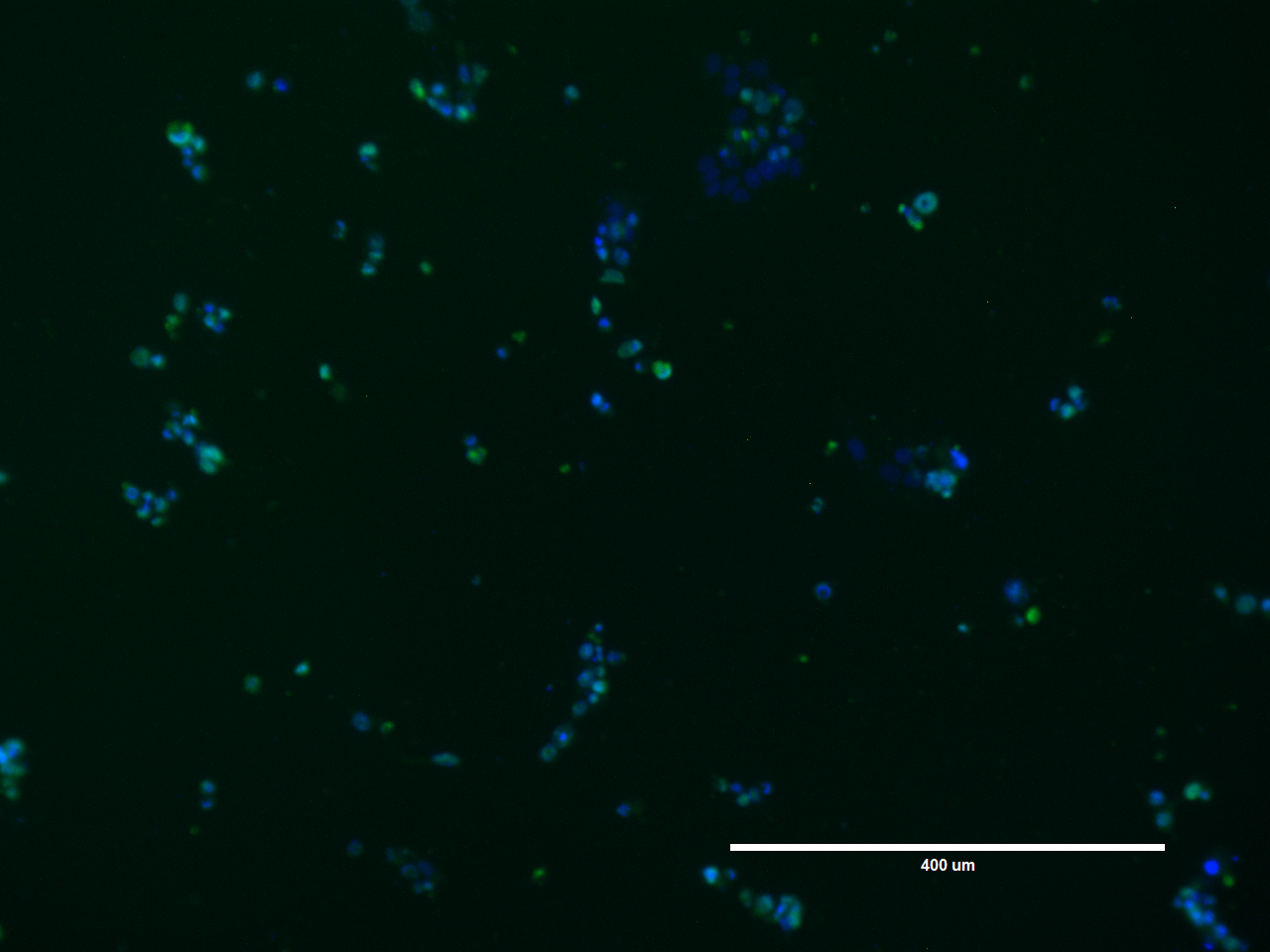

Supplement: Figure 2—figure supplement 1—source data 1. [file elife-73792-fig2-figsupp1-data1.zip › Figure 2-figure supplement 1-source data/1b/jeg3/jeg372.tif]

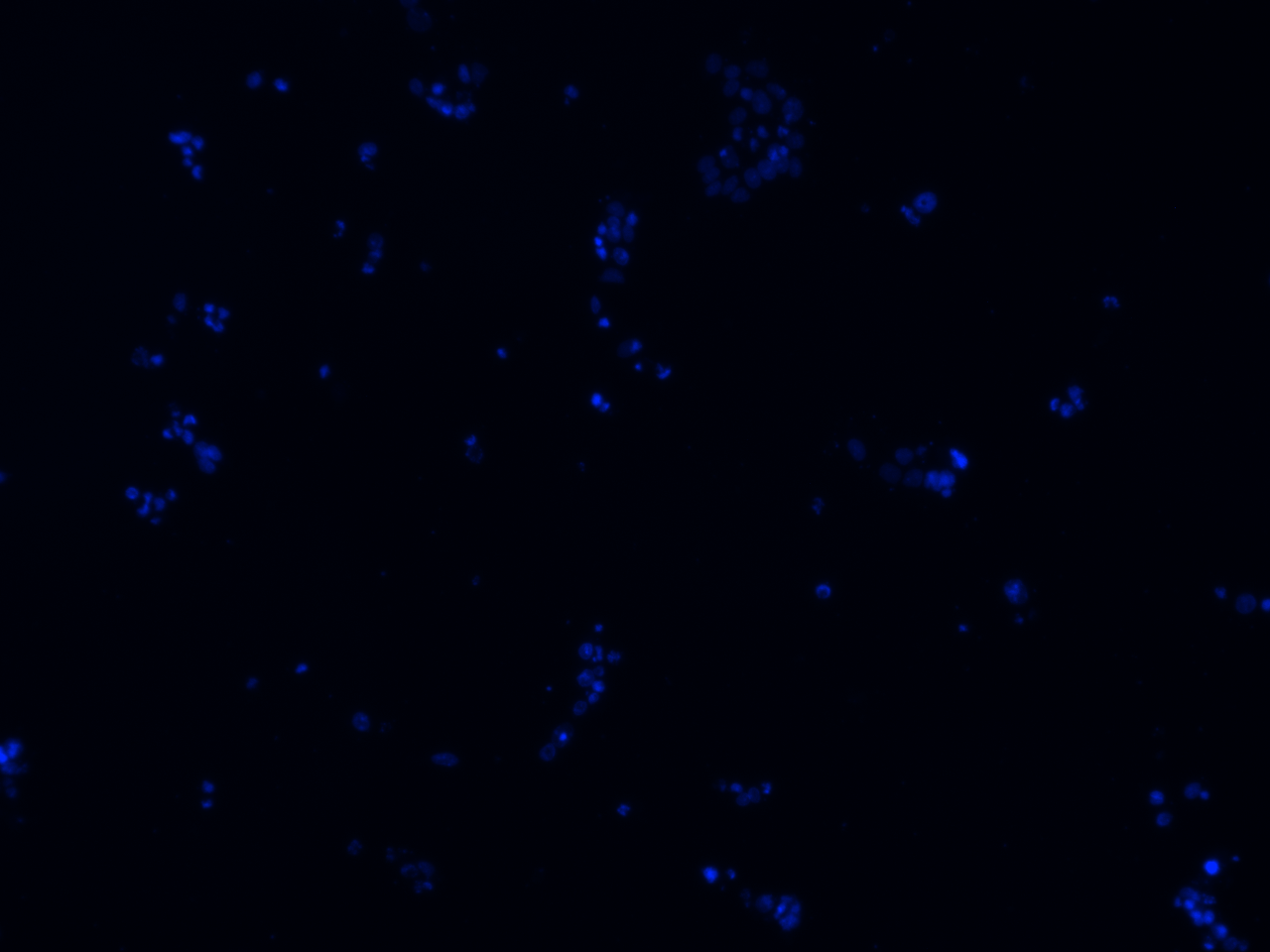

Supplement: Figure 2—figure supplement 1—source data 1. [file elife-73792-fig2-figsupp1-data1.zip › Figure 2-figure supplement 1-source data/1b/jeg3/jeg372_DAPI.tif]

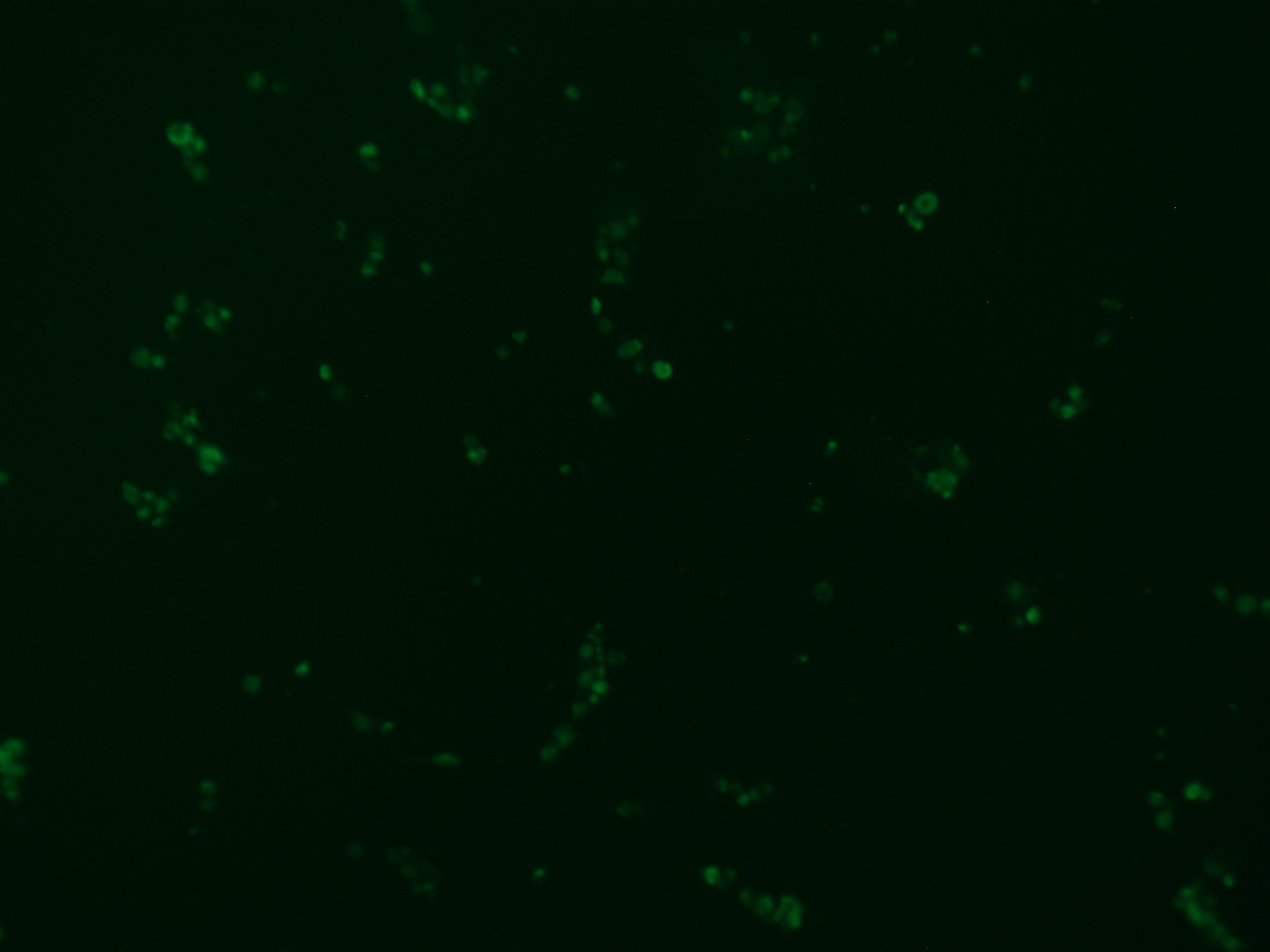

Supplement: Figure 2—figure supplement 1—source data 1. [file elife-73792-fig2-figsupp1-data1.zip › Figure 2-figure supplement 1-source data/1b/jeg3/jeg372_GFP.tif]

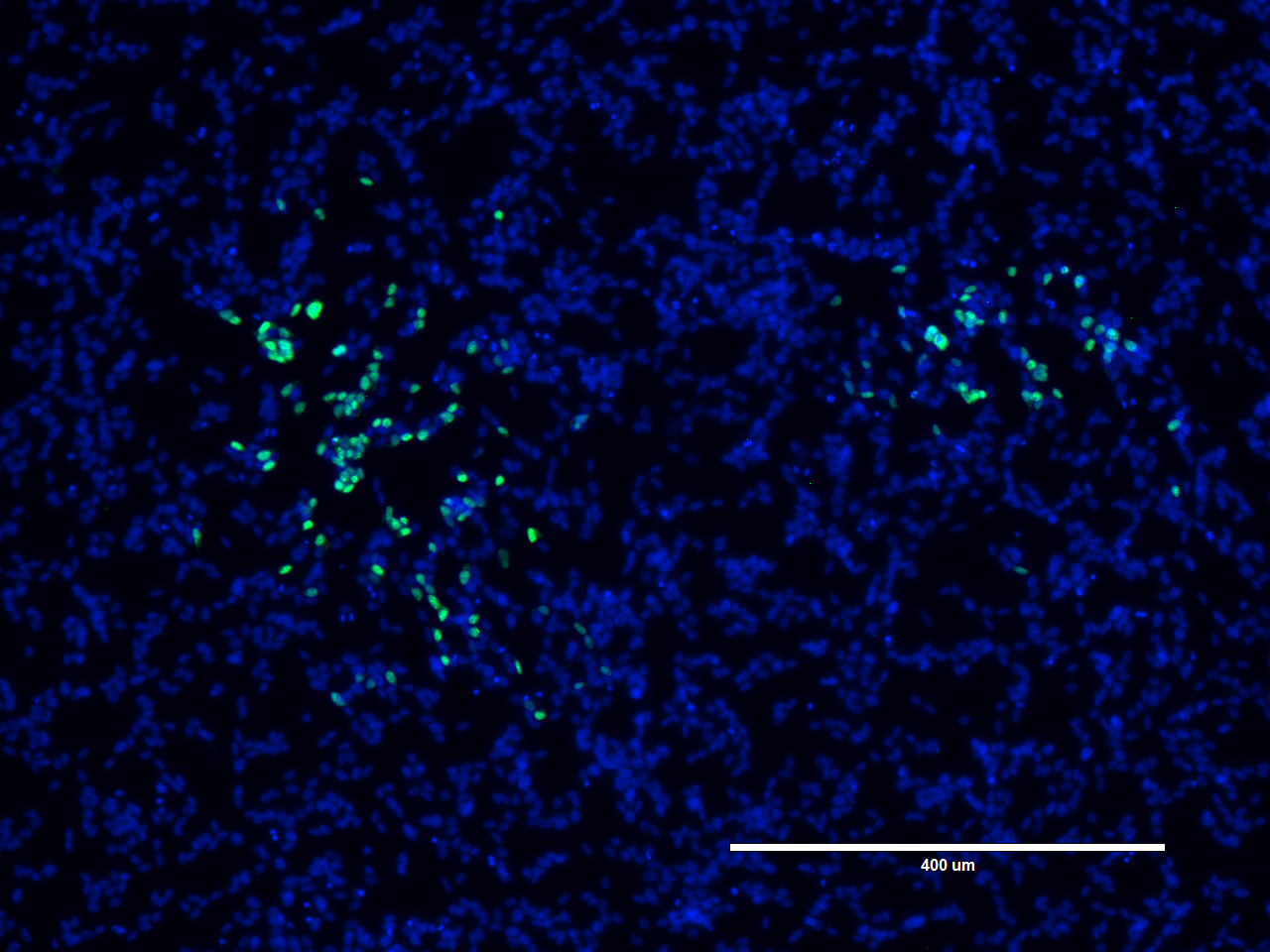

Supplement: Figure 2—figure supplement 1—source data 1. [file elife-73792-fig2-figsupp1-data1.zip › Figure 2-figure supplement 1-source data/1b/shsy5y/shsy5y 48.tif]

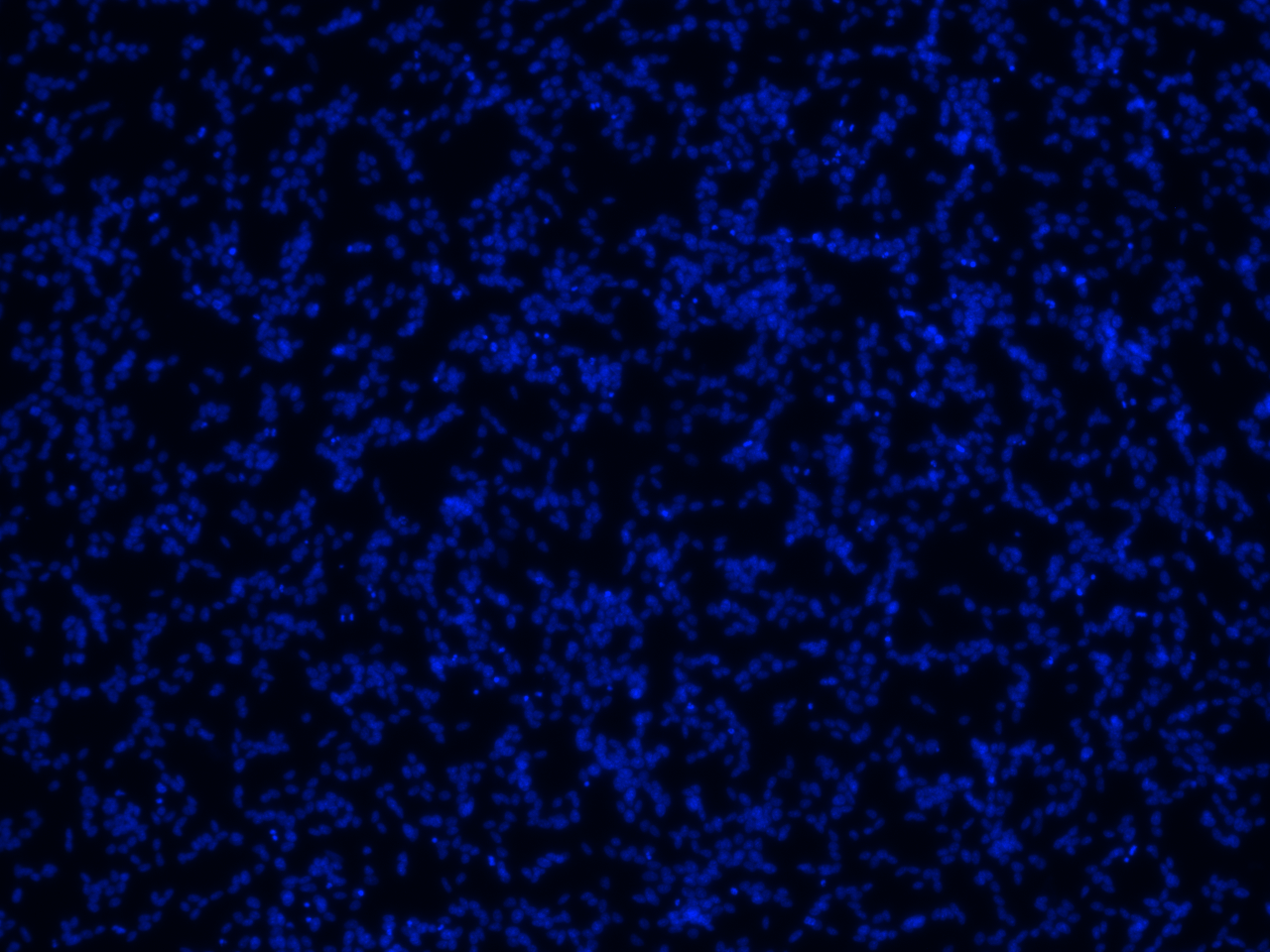

Supplement: Figure 2—figure supplement 1—source data 1. [file elife-73792-fig2-figsupp1-data1.zip › Figure 2-figure supplement 1-source data/1b/shsy5y/shsy5y 48_DAPI.tif]

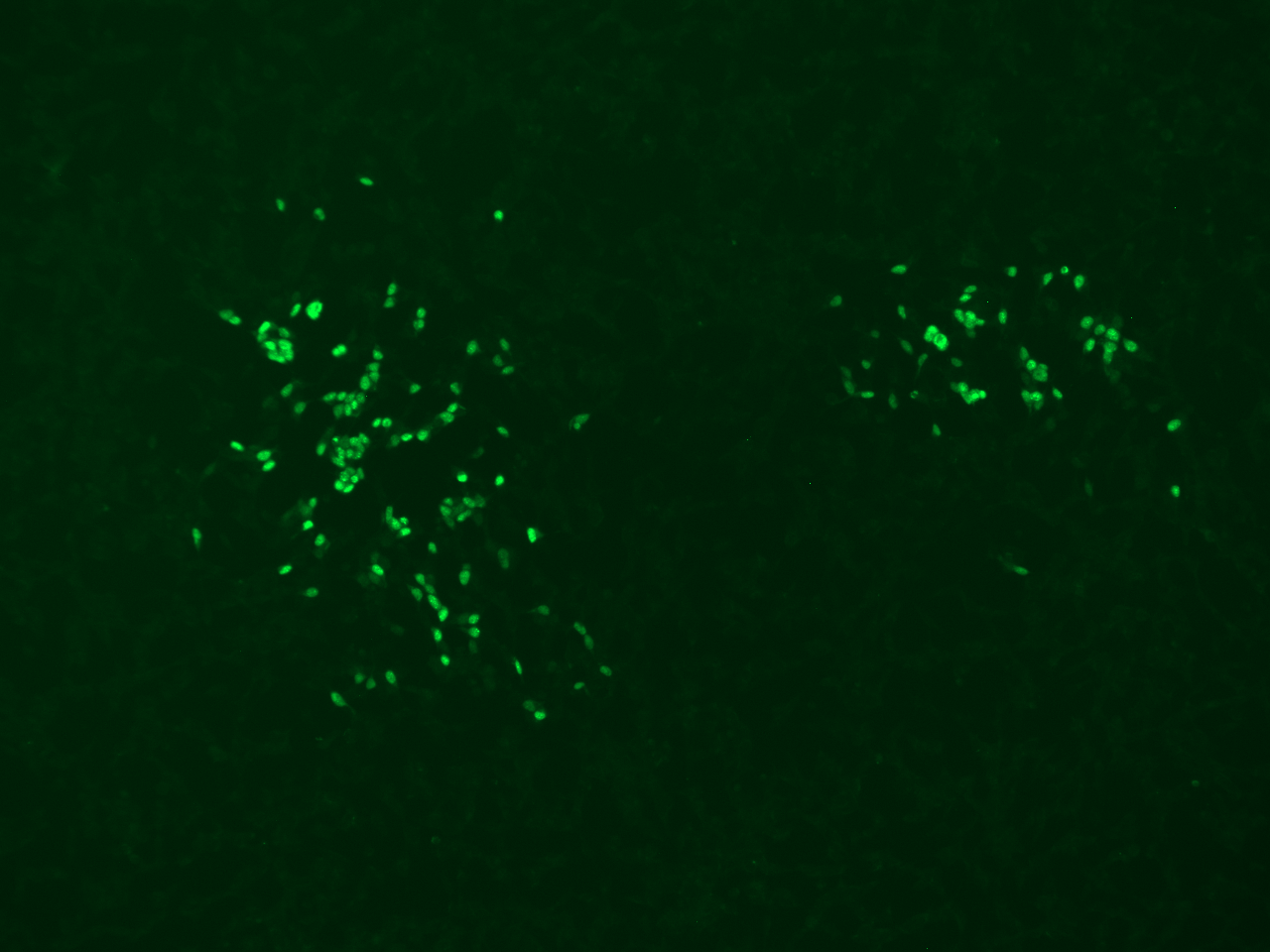

Supplement: Figure 2—figure supplement 1—source data 1. [file elife-73792-fig2-figsupp1-data1.zip › Figure 2-figure supplement 1-source data/1b/shsy5y/shsy5y 48_GFP.tif]

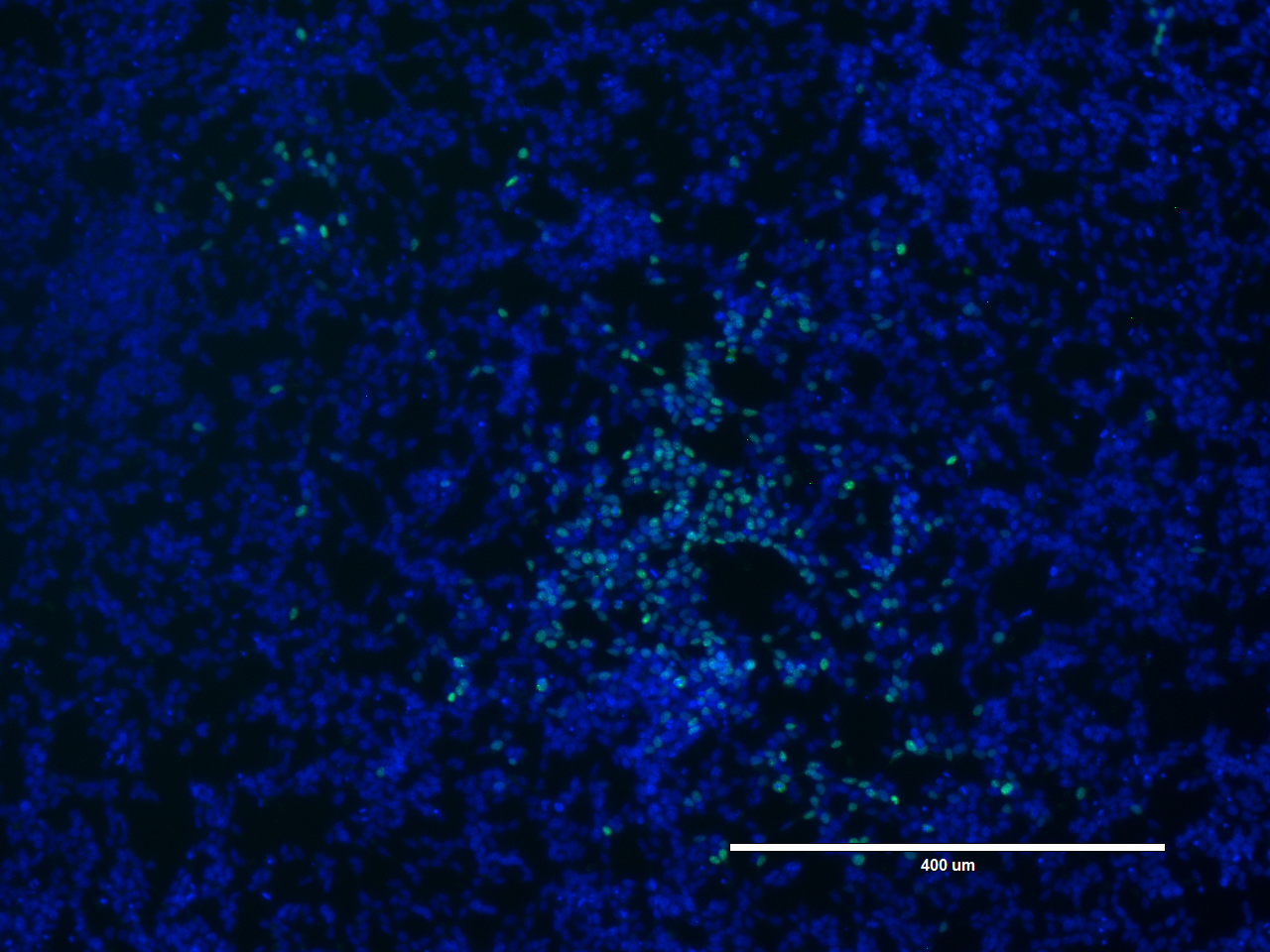

Supplement: Figure 2—figure supplement 1—source data 1. [file elife-73792-fig2-figsupp1-data1.zip › Figure 2-figure supplement 1-source data/1b/shsy5y/shsy5y 72.tif]

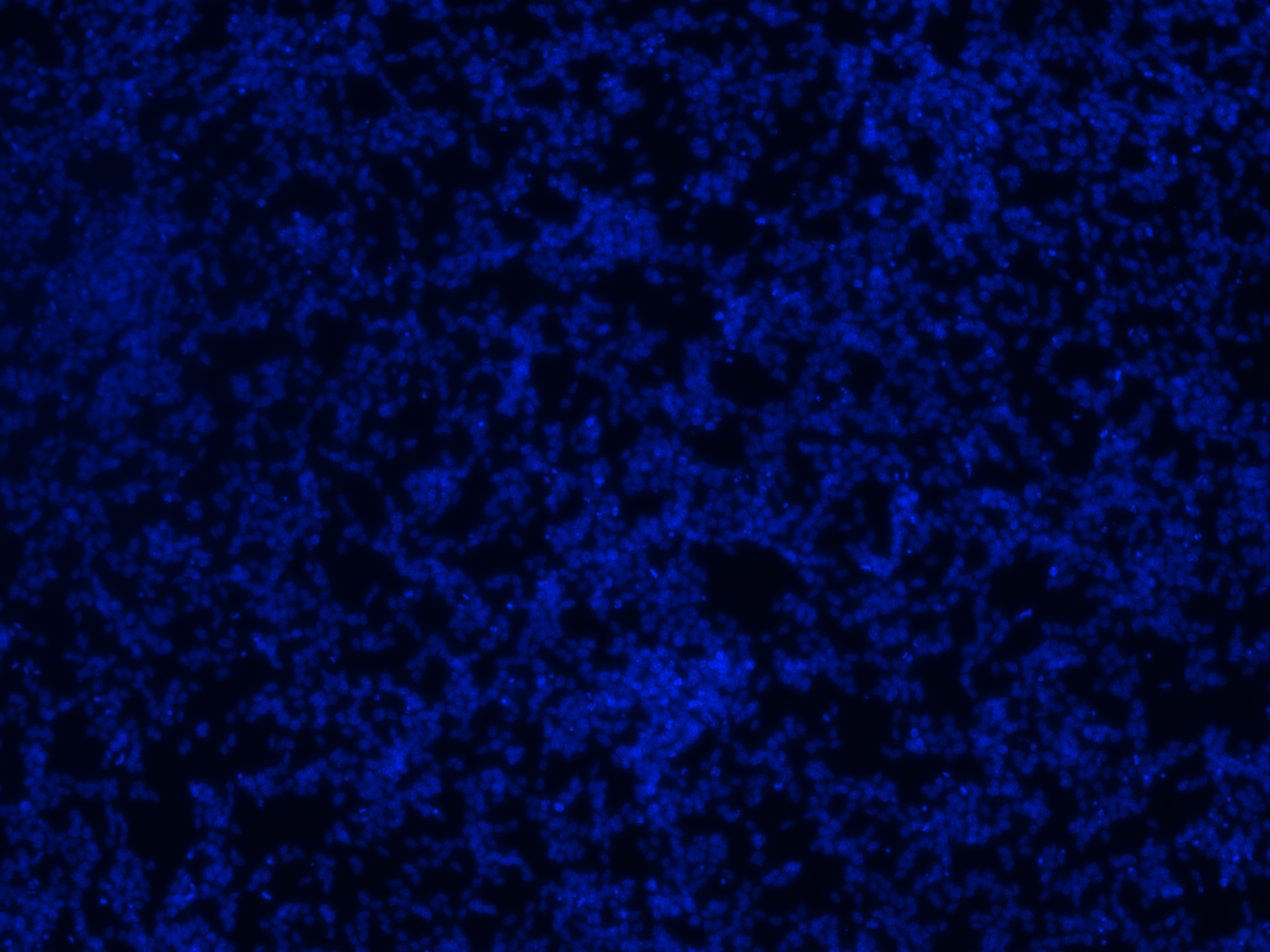

Supplement: Figure 2—figure supplement 1—source data 1. [file elife-73792-fig2-figsupp1-data1.zip › Figure 2-figure supplement 1-source data/1b/shsy5y/shsy5y 72_DAPI.tif]

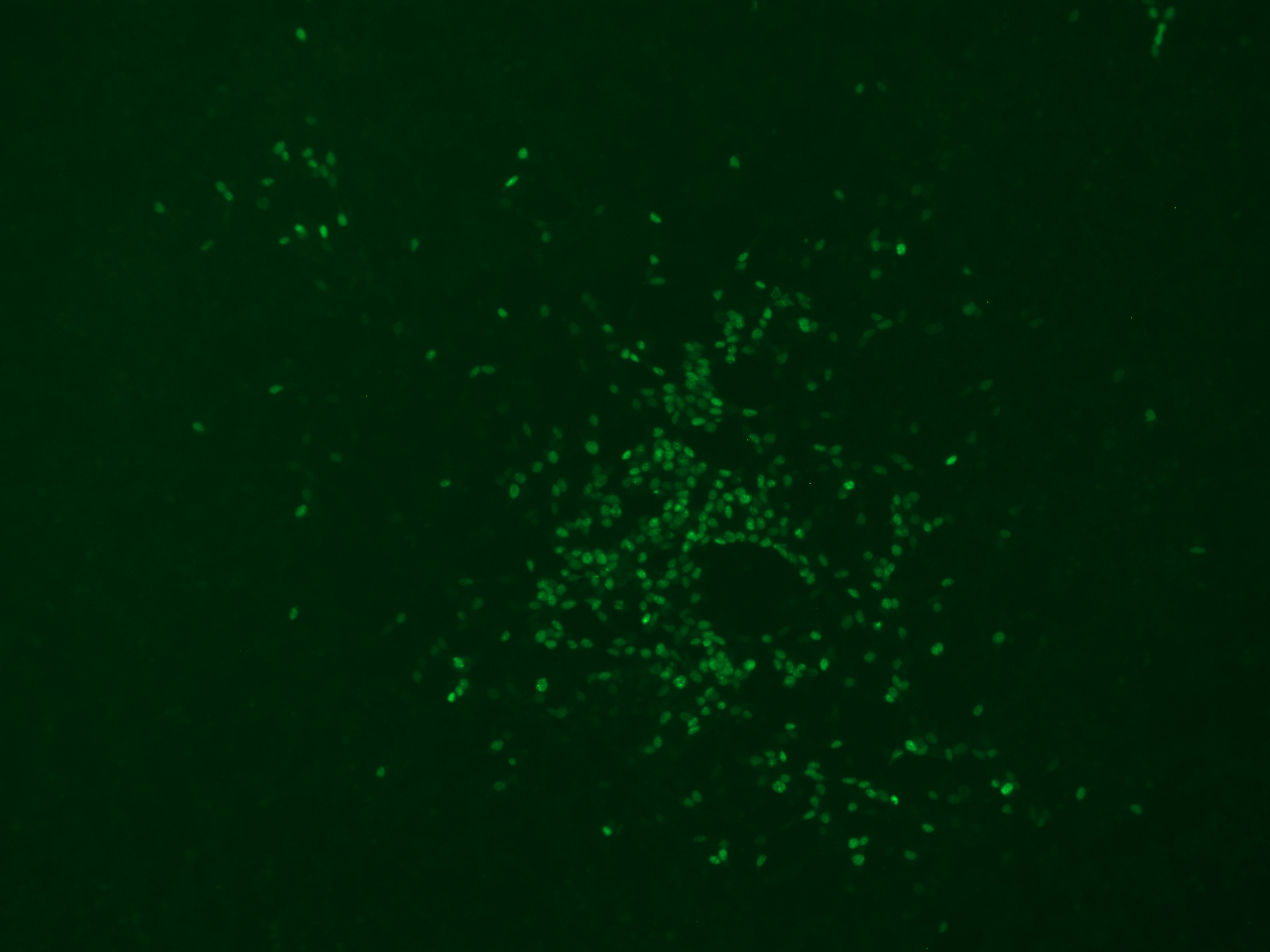

Supplement: Figure 2—figure supplement 1—source data 1. [file elife-73792-fig2-figsupp1-data1.zip › Figure 2-figure supplement 1-source data/1b/shsy5y/shsy5y 72_GFP.tif]

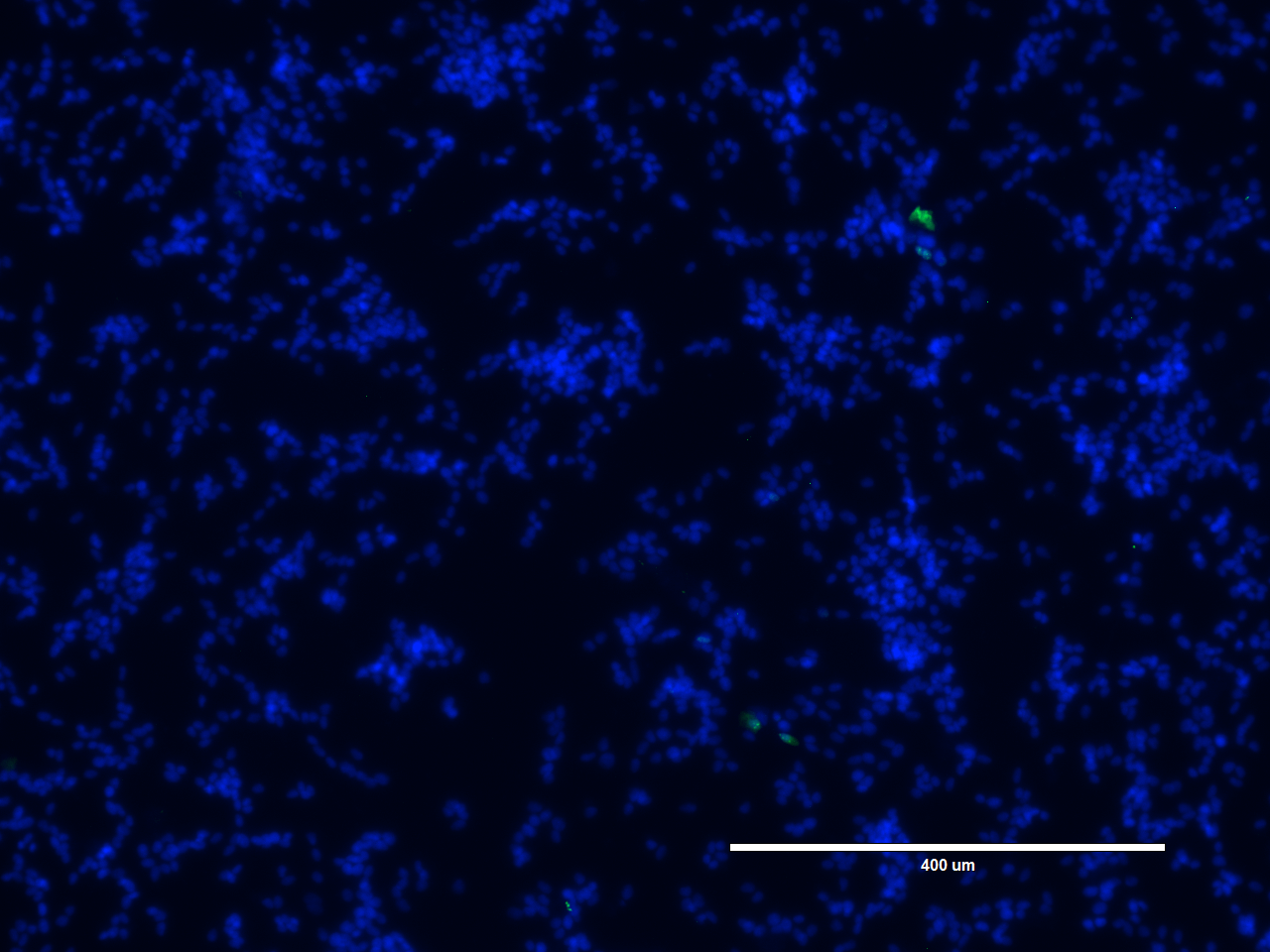

Supplement: Figure 2—figure supplement 1—source data 1. [file elife-73792-fig2-figsupp1-data1.zip › Figure 2-figure supplement 1-source data/1b/shsy5y/shsy5y24.tif]

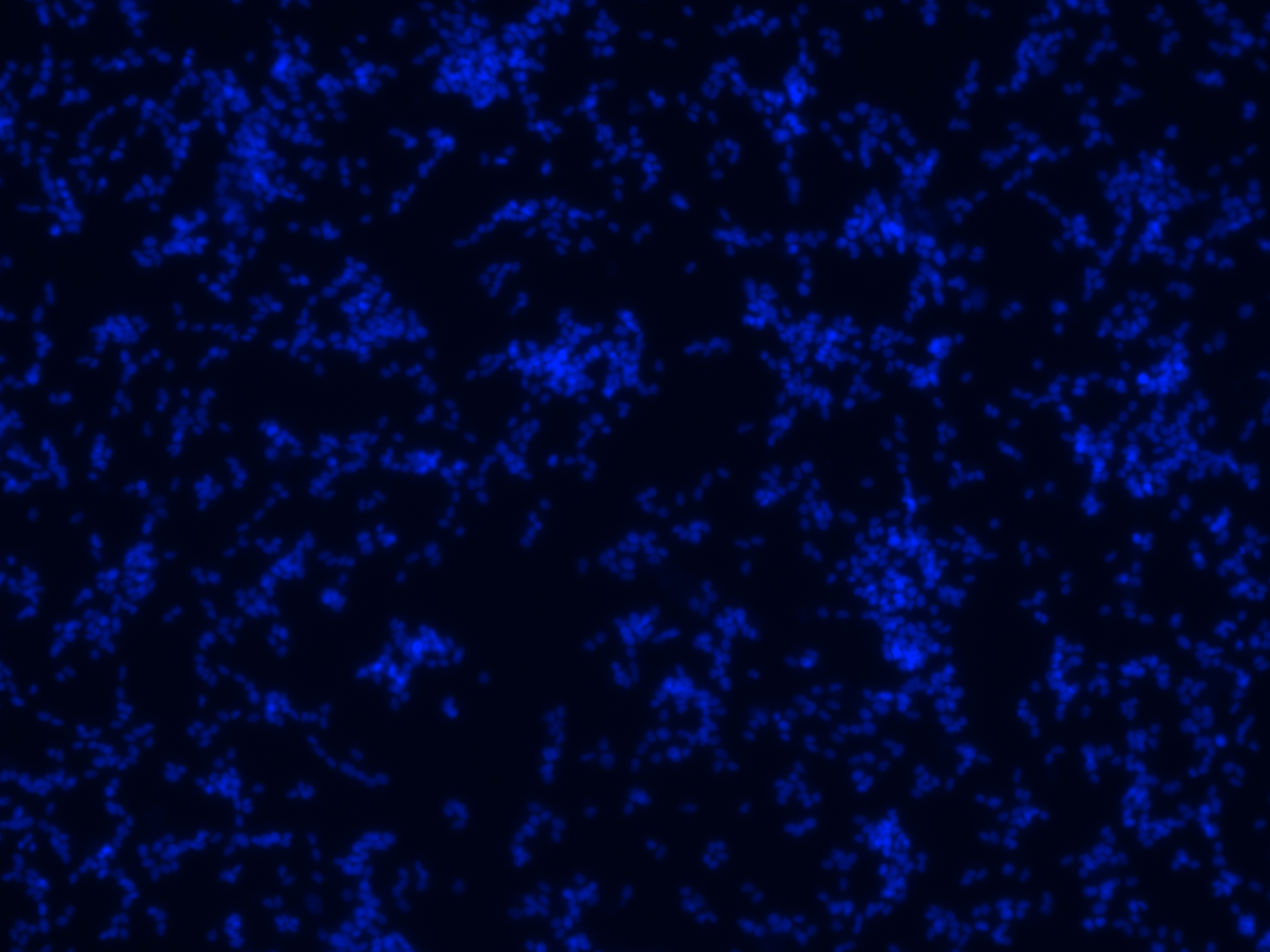

Supplement: Figure 2—figure supplement 1—source data 1. [file elife-73792-fig2-figsupp1-data1.zip › Figure 2-figure supplement 1-source data/1b/shsy5y/shsy5y24_DAPI.tif]

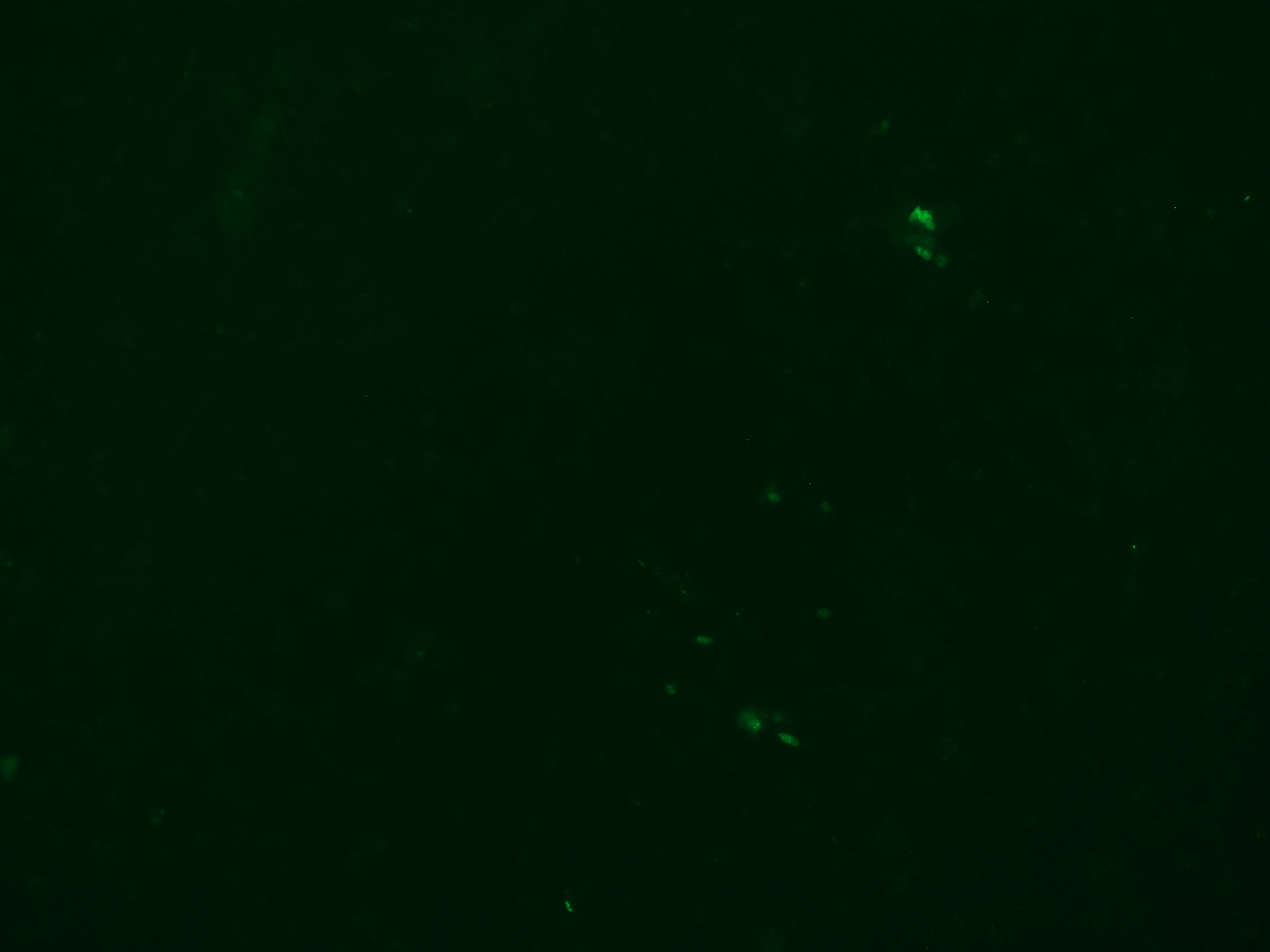

Supplement: Figure 2—figure supplement 1—source data 1. [file elife-73792-fig2-figsupp1-data1.zip › Figure 2-figure supplement 1-source data/1b/shsy5y/shsy5y24_GFP.tif]

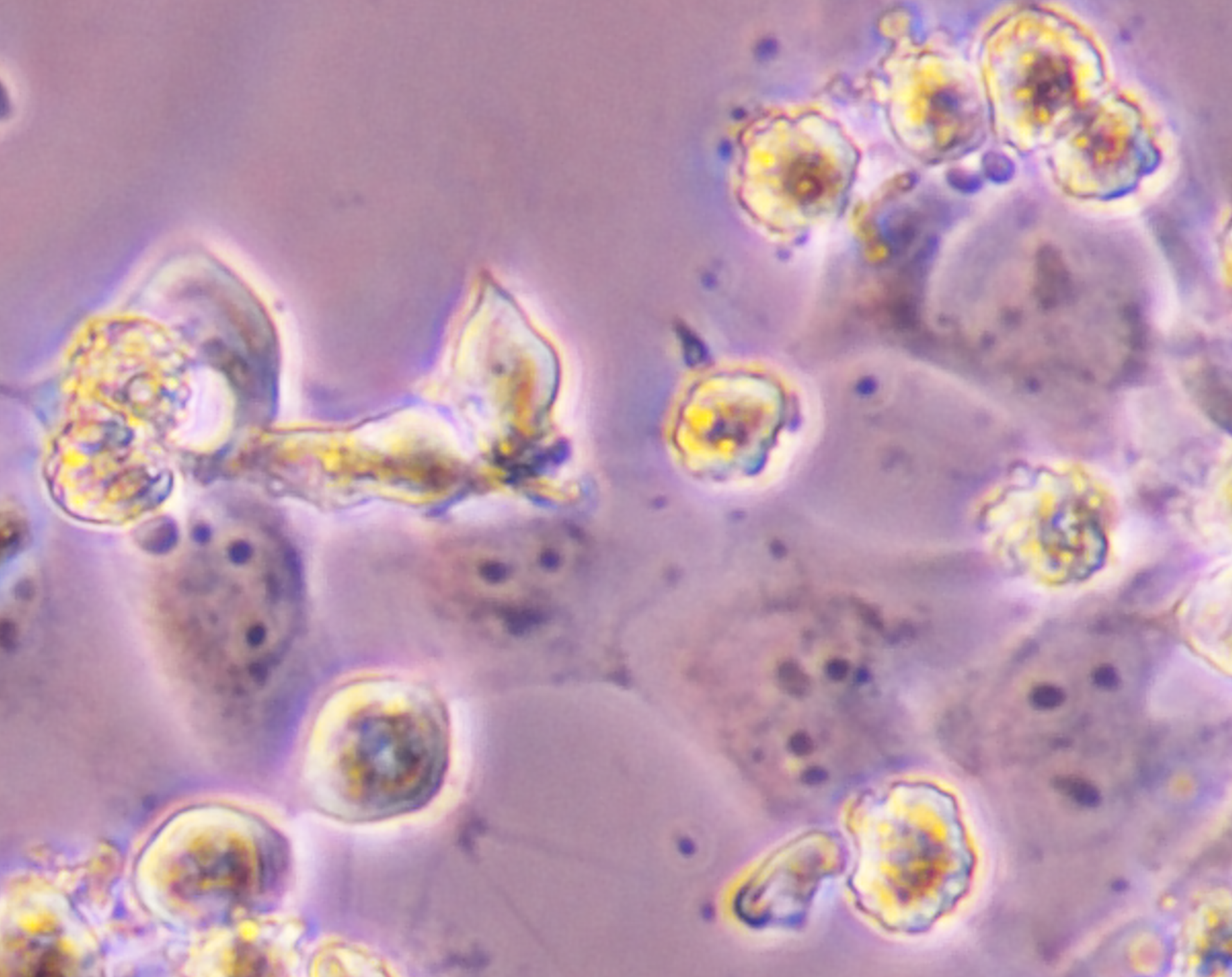

Supplement: Figure 3—source data 1. [file elife-73792-fig3-data1.zip › Figure 3-source data 1/Fig 3A/devd zikv.tif]

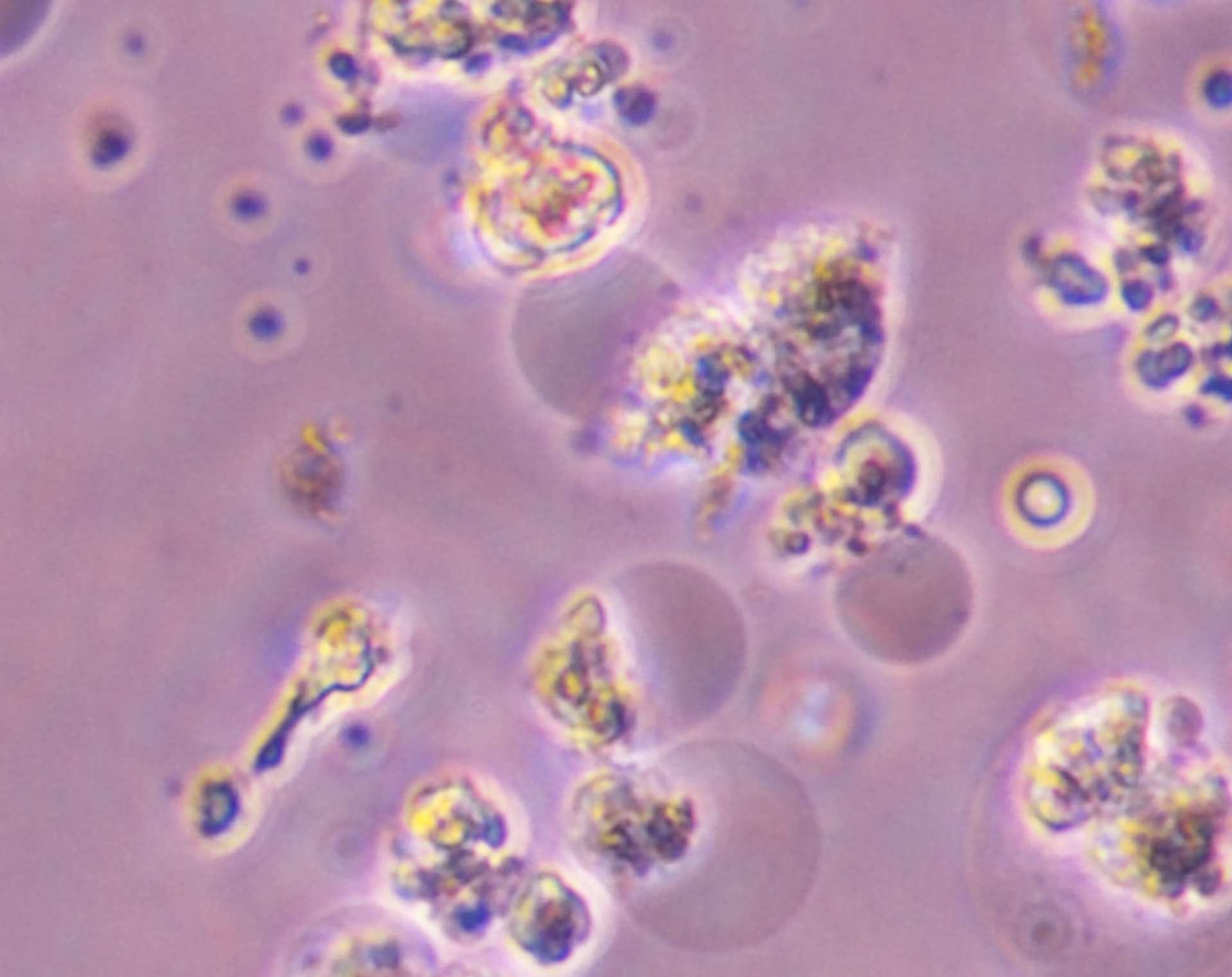

Supplement: Figure 3—source data 1. [file elife-73792-fig3-data1.zip › Figure 3-source data 1/Fig 3A/dmso zikv.tif]

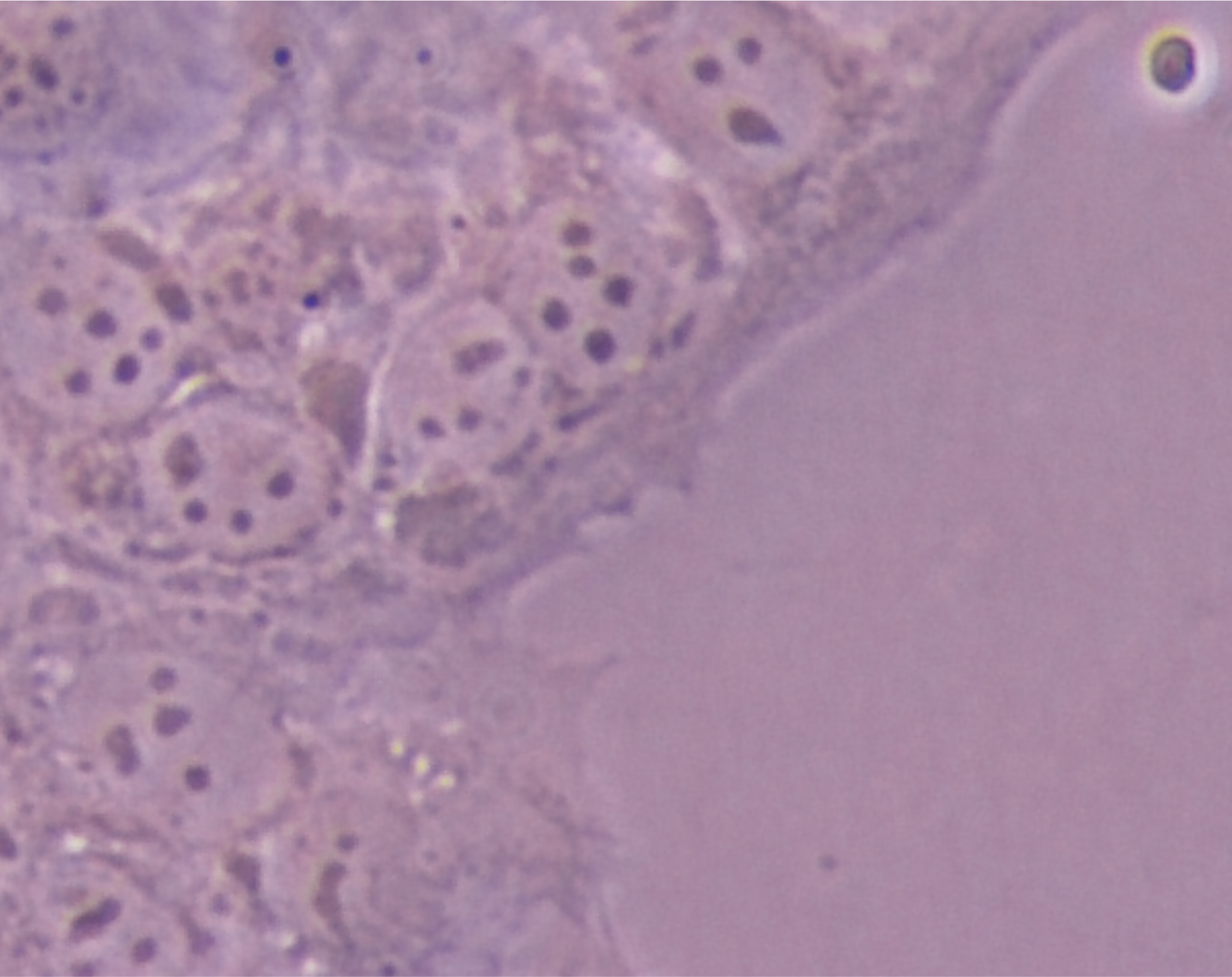

Supplement: Figure 3—source data 1. [file elife-73792-fig3-data1.zip › Figure 3-source data 1/Fig 3A/dmso.tif]

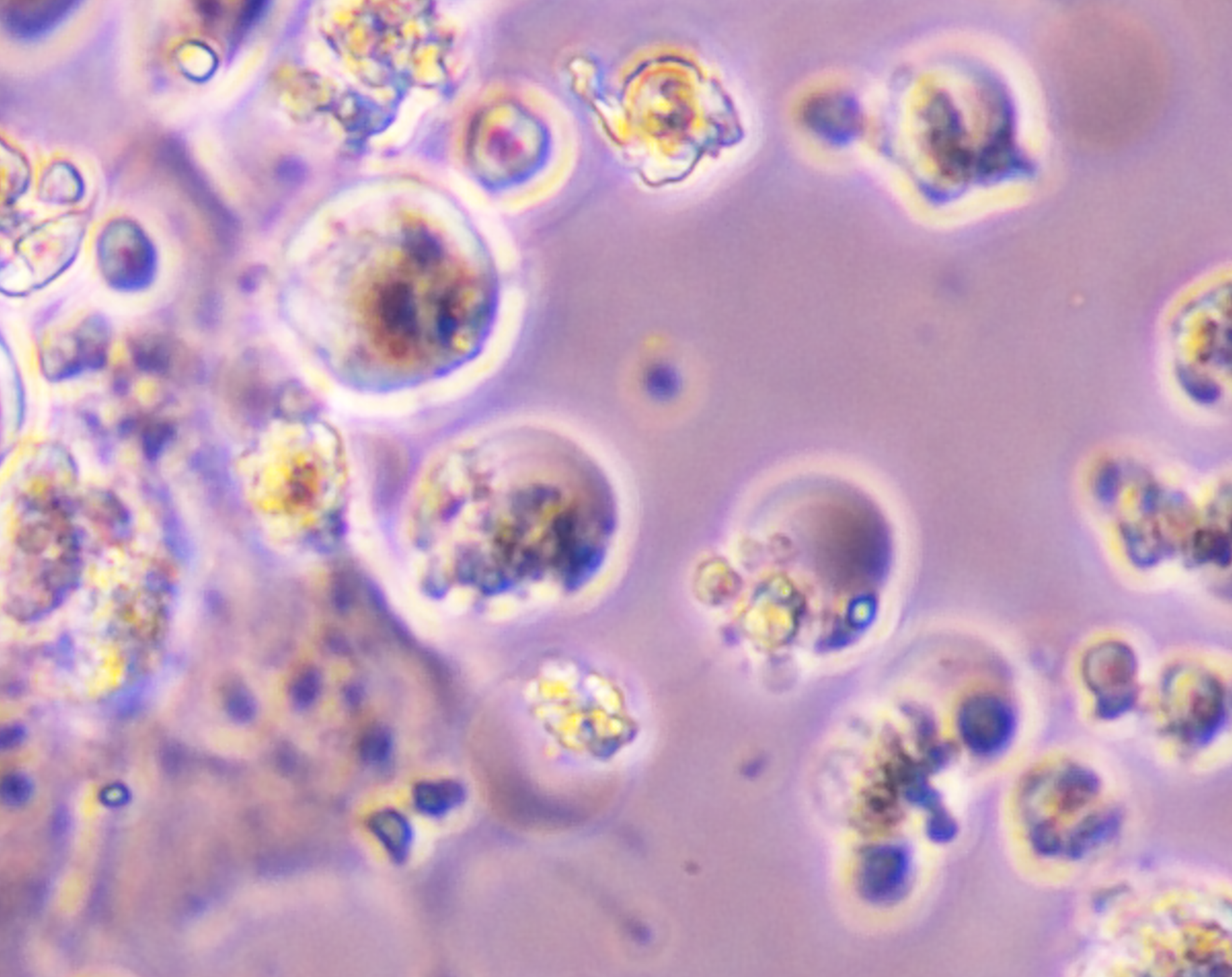

Supplement: Figure 3—source data 1. [file elife-73792-fig3-data1.zip › Figure 3-source data 1/Fig 3A/gsk872 zikv.tif]

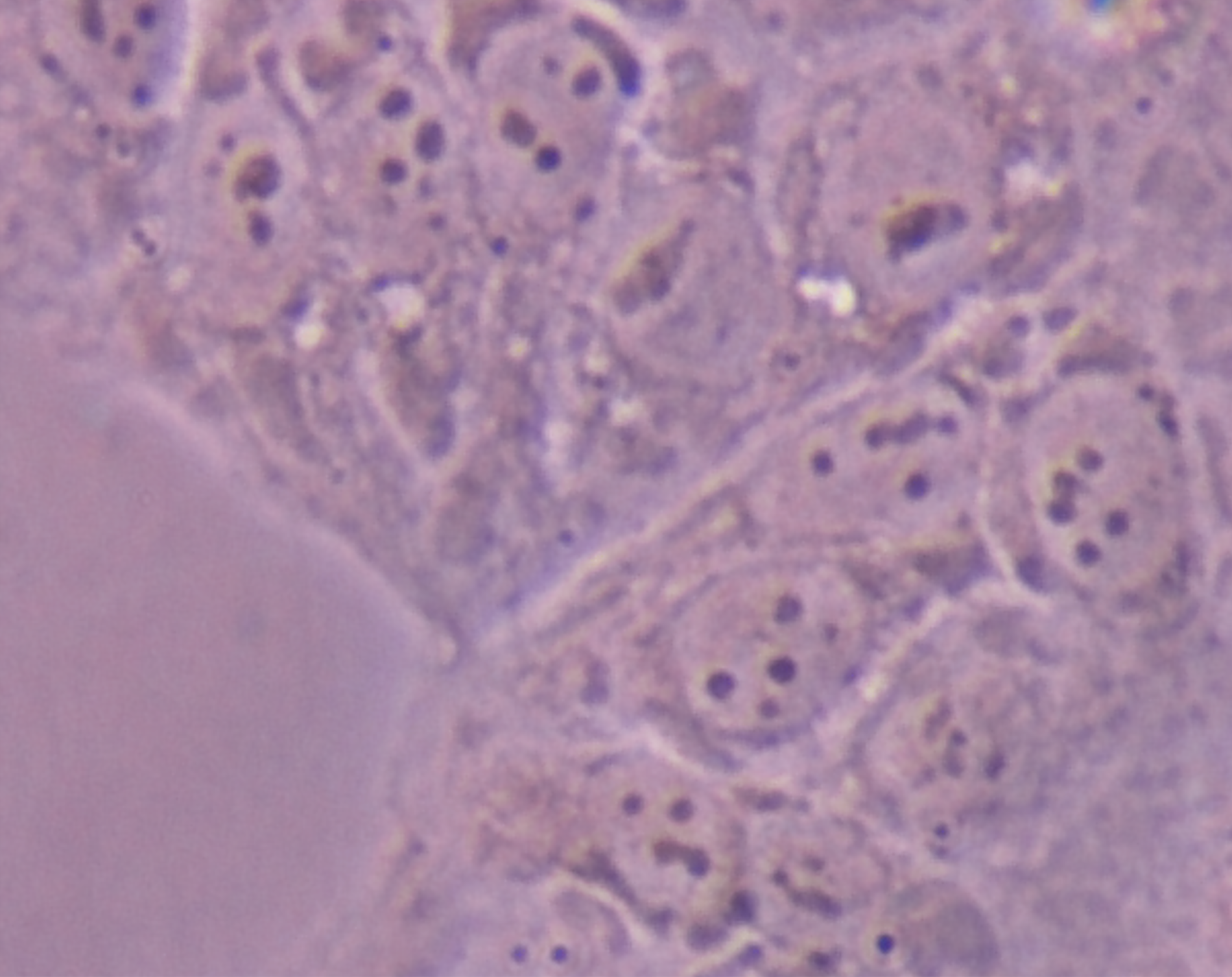

Supplement: Figure 3—source data 1. [file elife-73792-fig3-data1.zip › Figure 3-source data 1/Fig 3A/mock.tif]

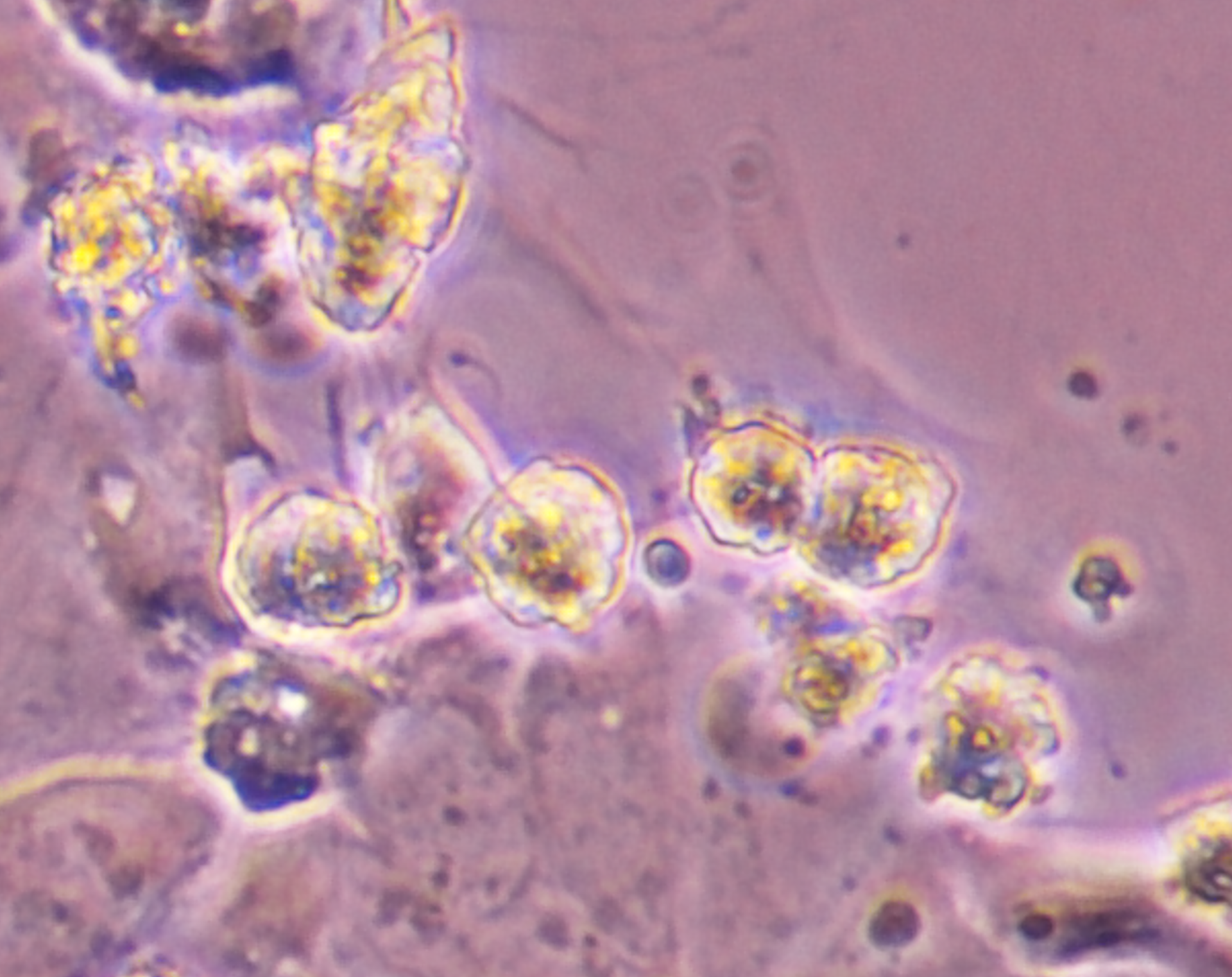

Supplement: Figure 3—source data 1. [file elife-73792-fig3-data1.zip › Figure 3-source data 1/Fig 3A/vad zikv.tif]

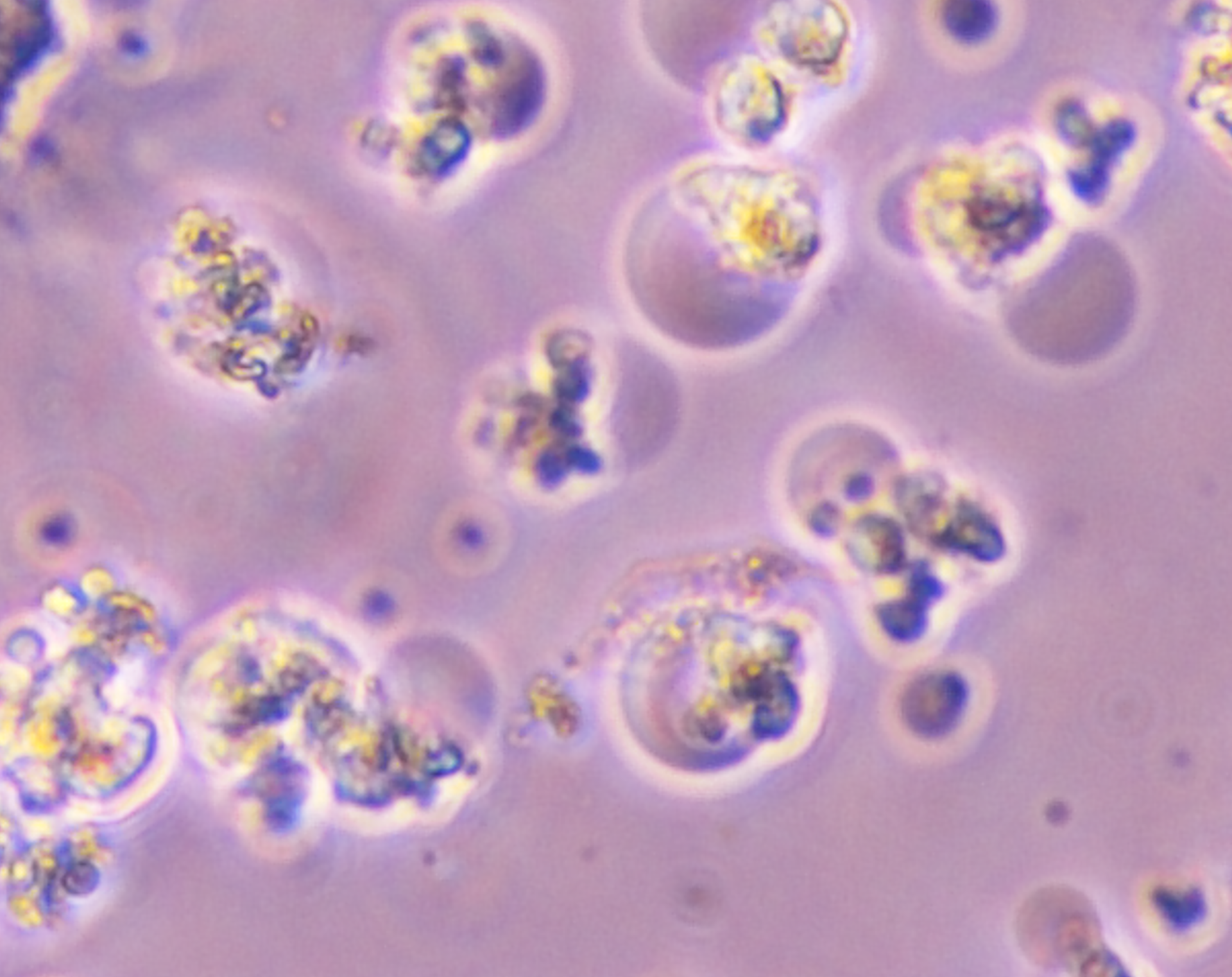

Supplement: Figure 3—source data 1. [file elife-73792-fig3-data1.zip › Figure 3-source data 1/Fig 3A/vx765 zikv.tif]

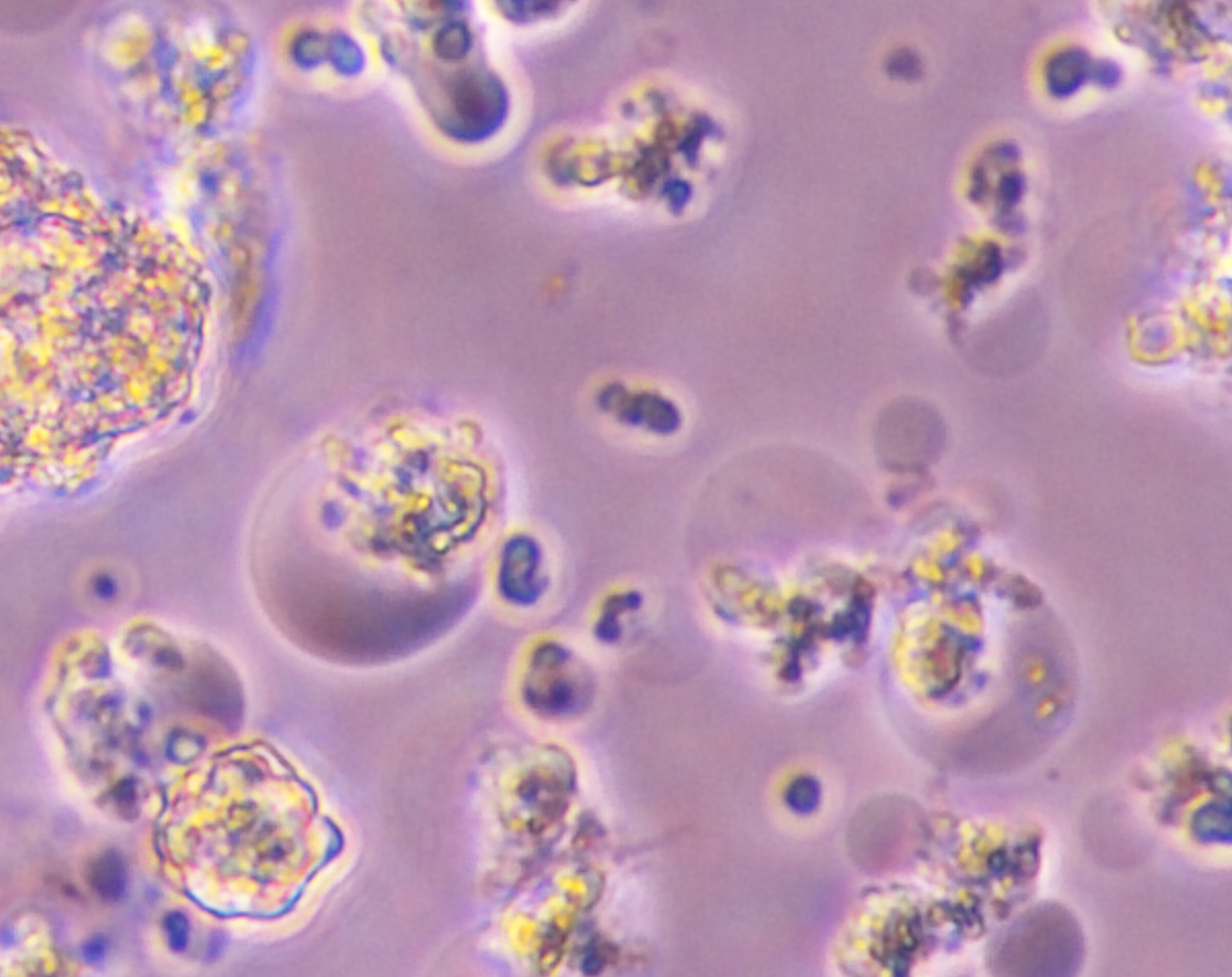

Supplement: Figure 3—source data 1. [file elife-73792-fig3-data1.zip › Figure 3-source data 1/Fig 3A/zikv.tif]

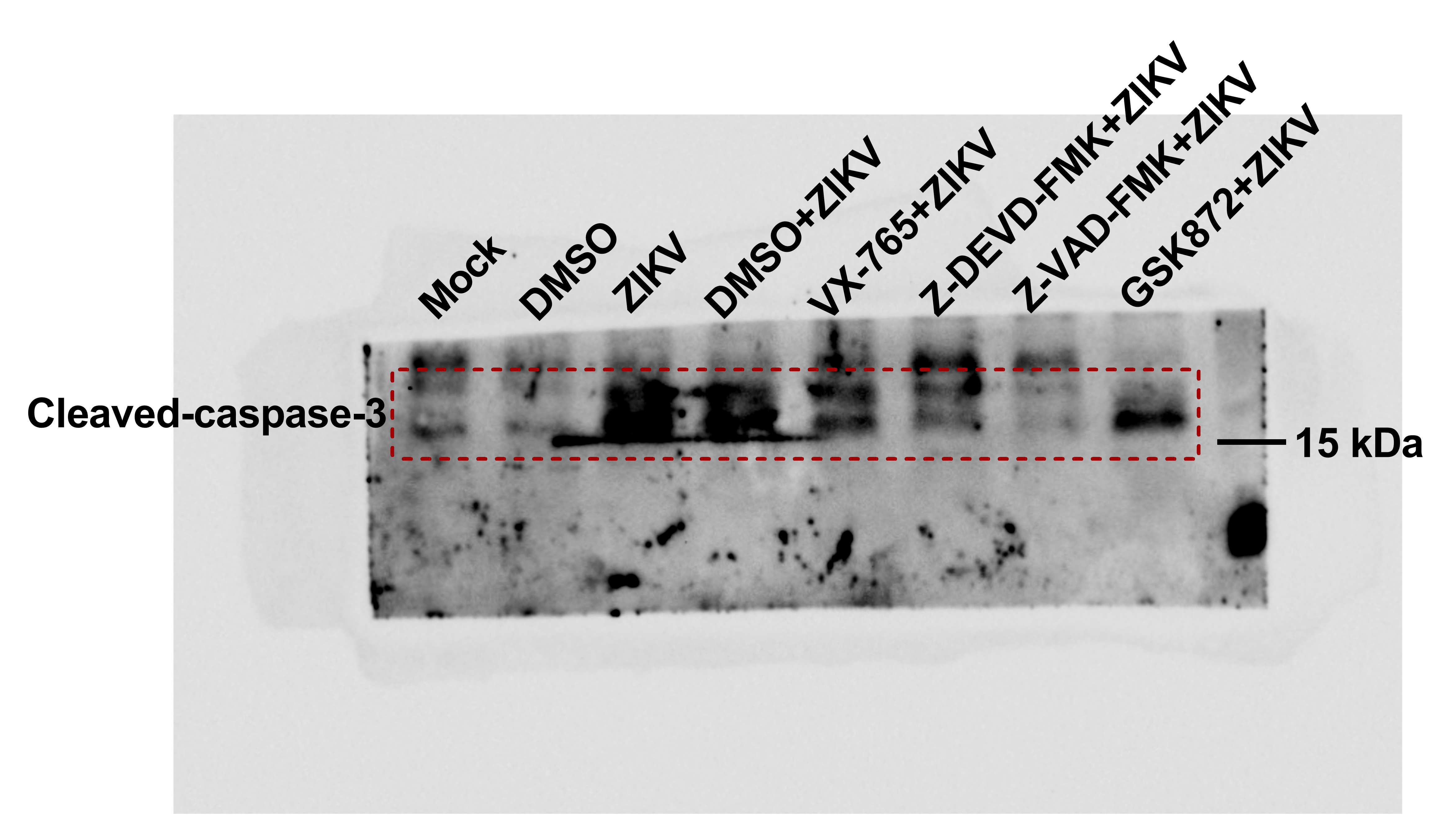

Supplement: Figure 3—source data 1. [file elife-73792-fig3-data1.zip › Figure 3-source data 1/Fig 3C/Figure 3C Cleaved-caspase-3-labeled.tif]

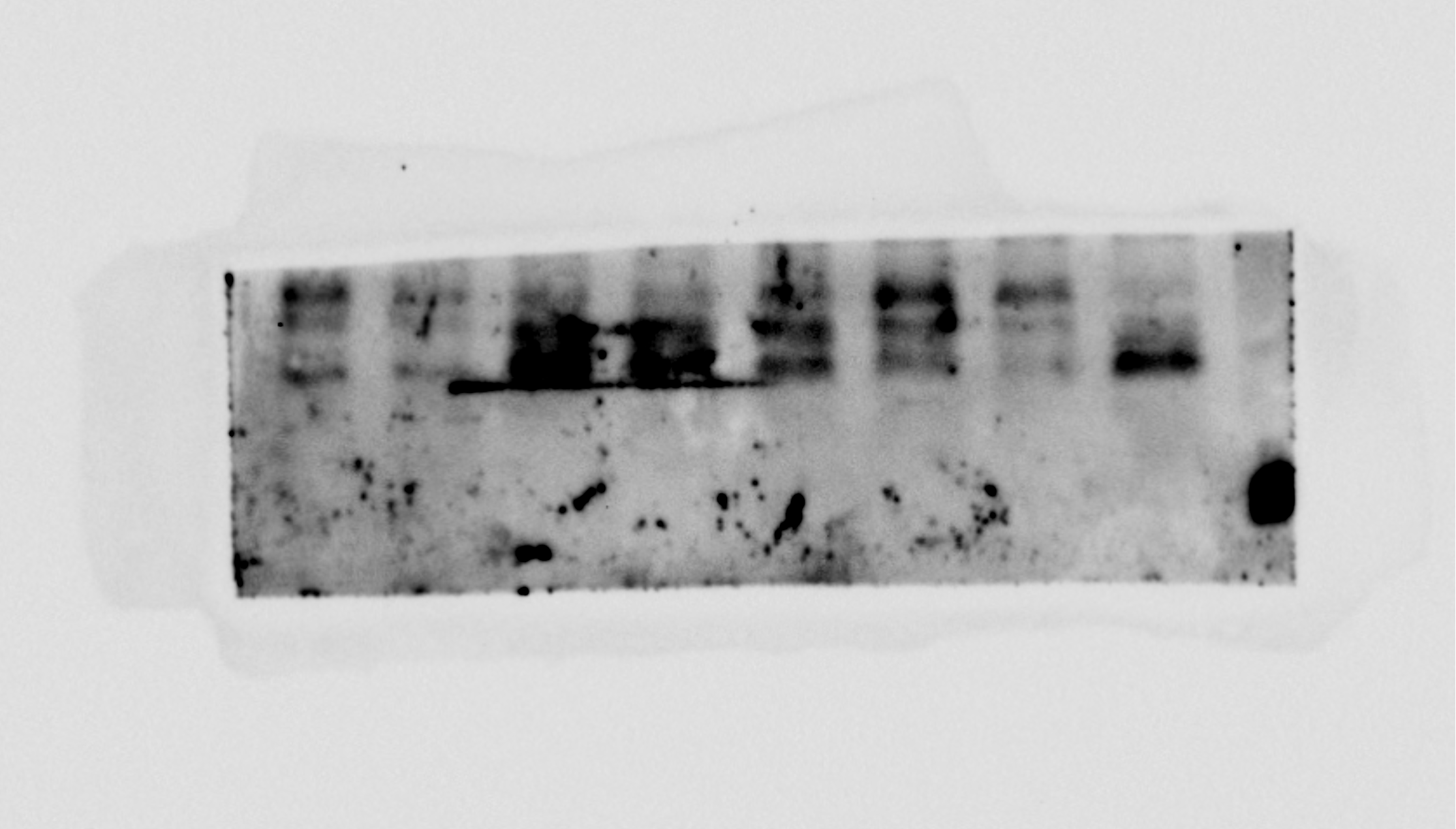

Supplement: Figure 3—source data 1. [file elife-73792-fig3-data1.zip › Figure 3-source data 1/Fig 3C/Figure 3C Cleaved-caspase-3-raw.tif]

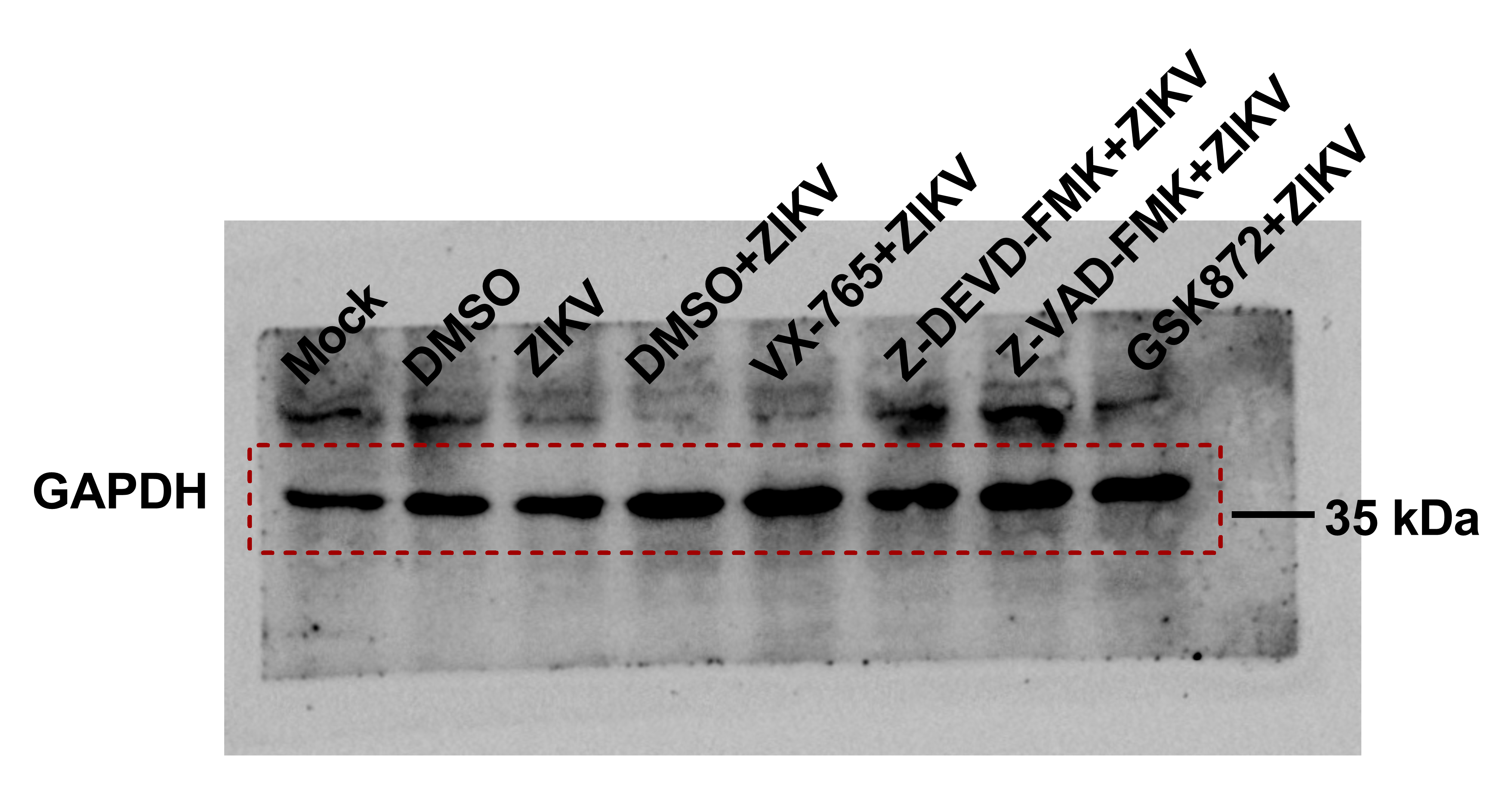

Supplement: Figure 3—source data 1. [file elife-73792-fig3-data1.zip › Figure 3-source data 1/Fig 3C/Figure 3C GAPDH-labeled.tif]

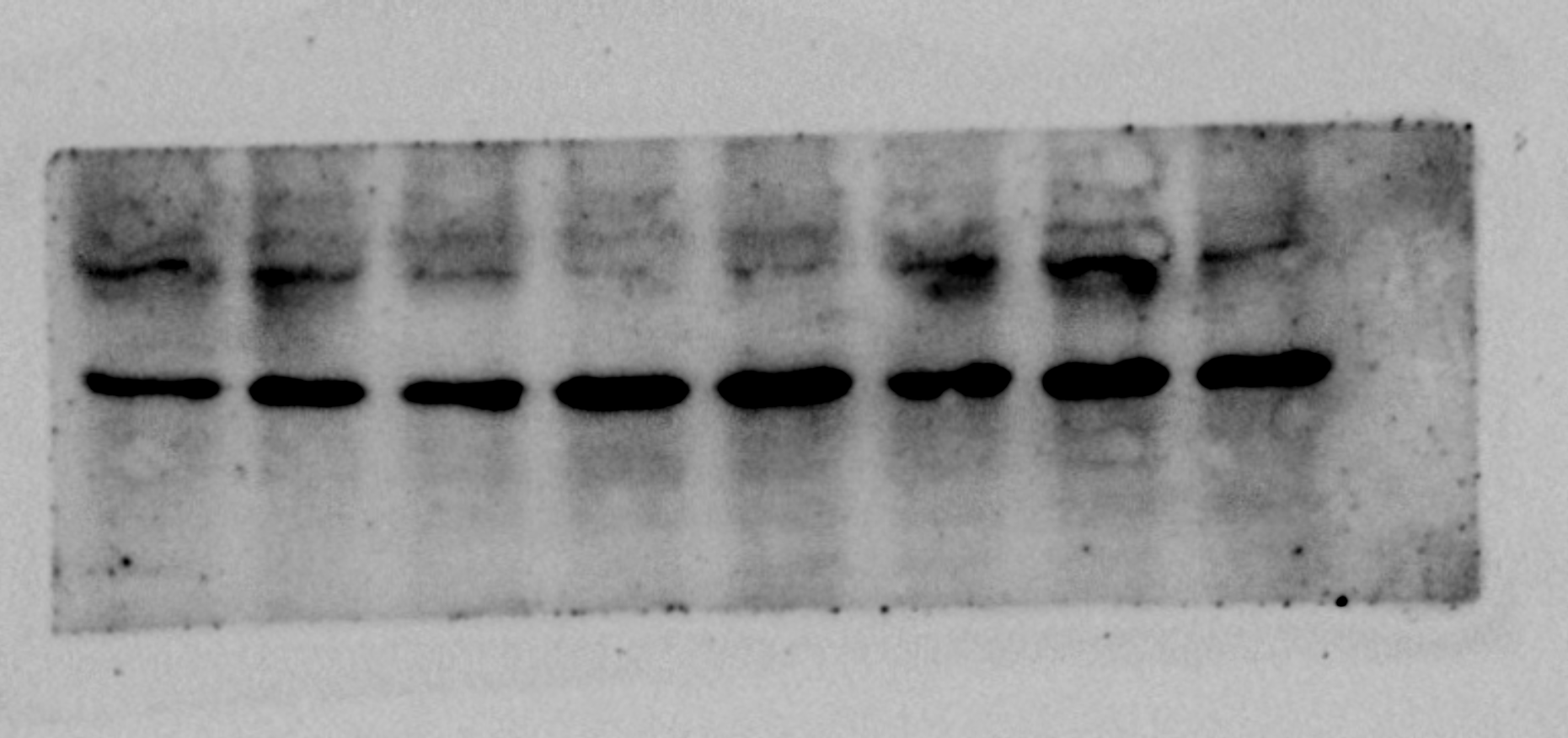

Supplement: Figure 3—source data 1. [file elife-73792-fig3-data1.zip › Figure 3-source data 1/Fig 3C/Figure 3C GAPDH-raw.tif]

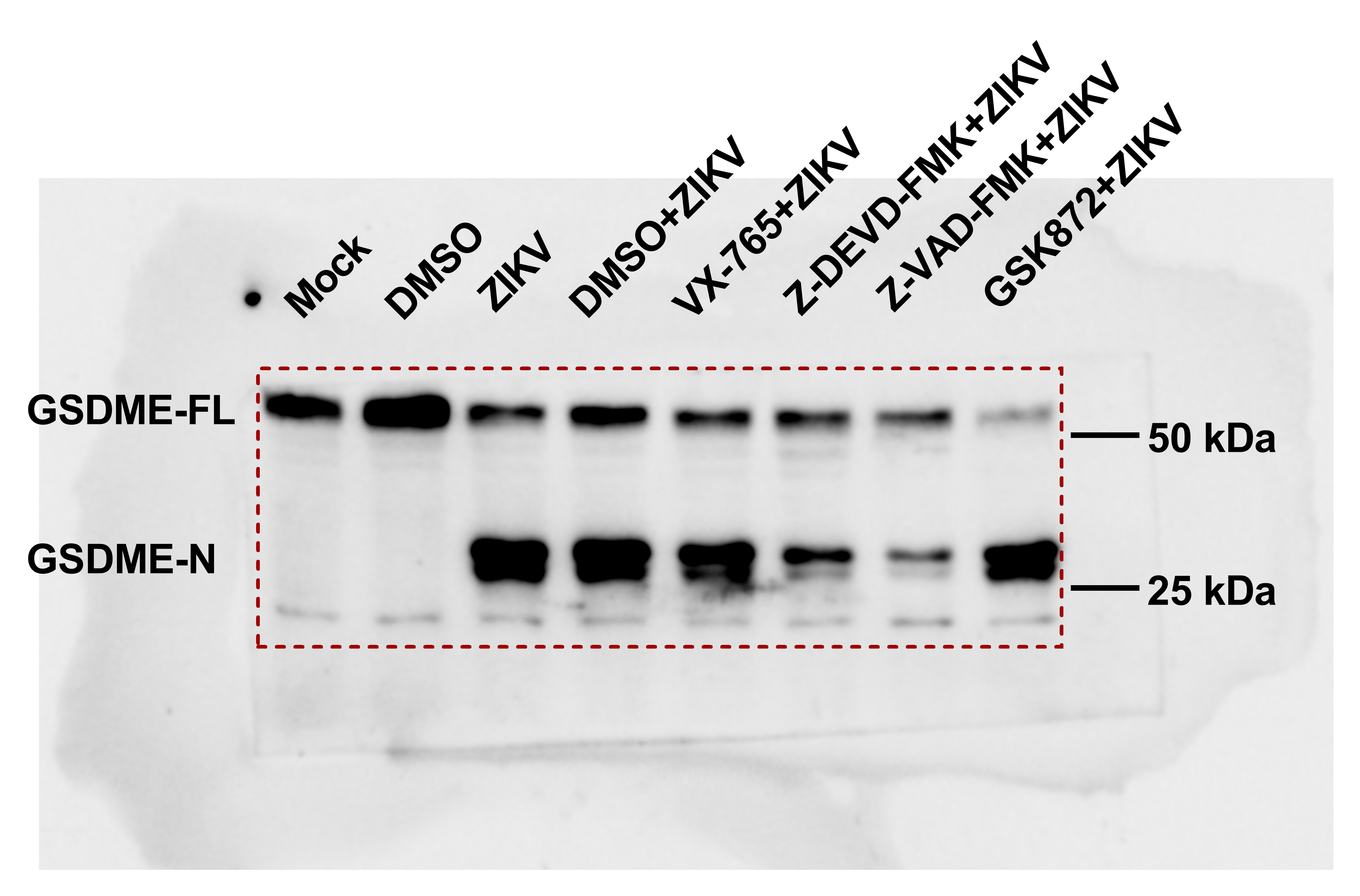

Supplement: Figure 3—source data 1. [file elife-73792-fig3-data1.zip › Figure 3-source data 1/Fig 3C/Figure 3C GSDME-labeled.tif]

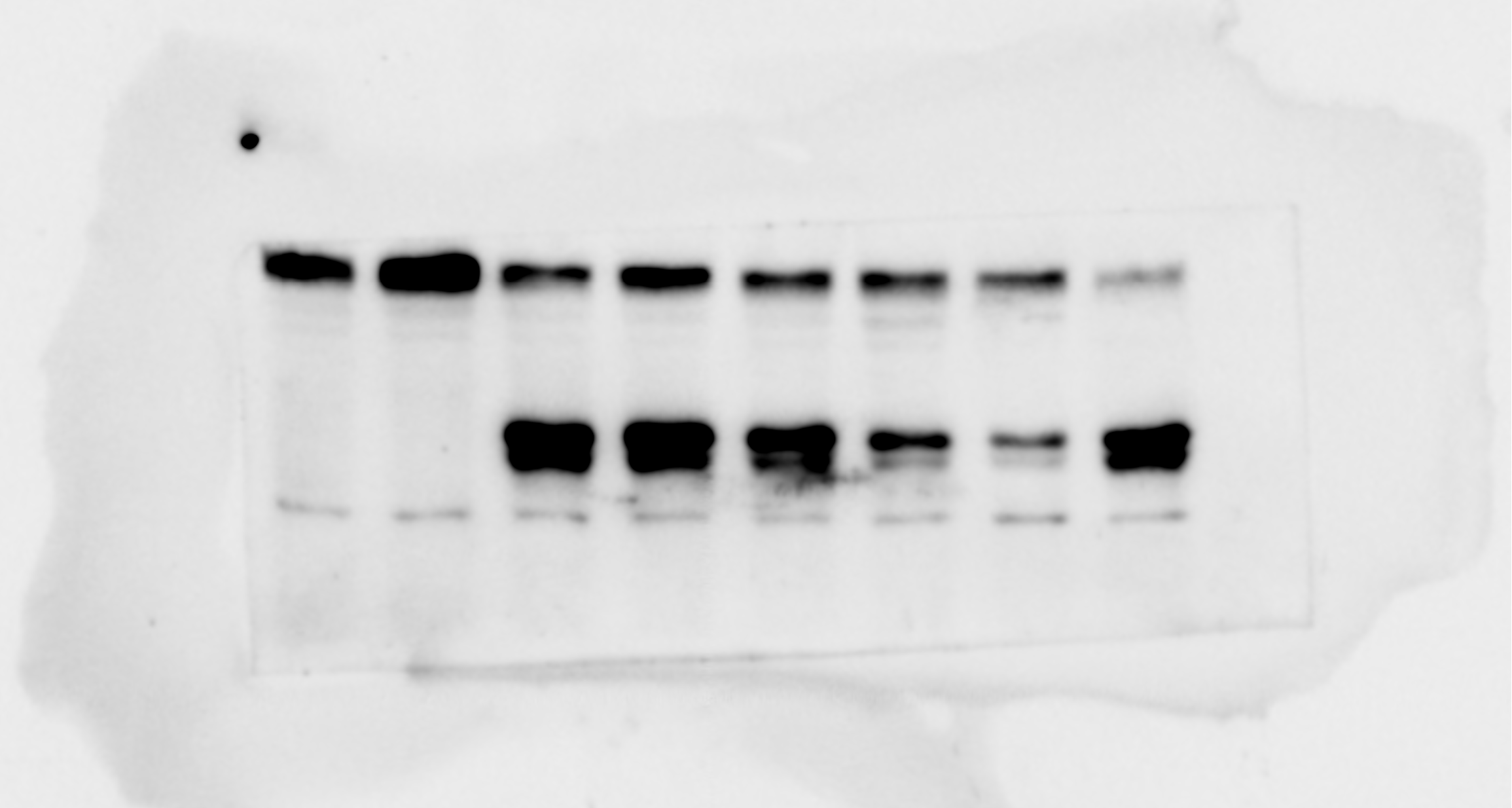

Supplement: Figure 3—source data 1. [file elife-73792-fig3-data1.zip › Figure 3-source data 1/Fig 3C/Figure 3C GSDME-raw.tif]

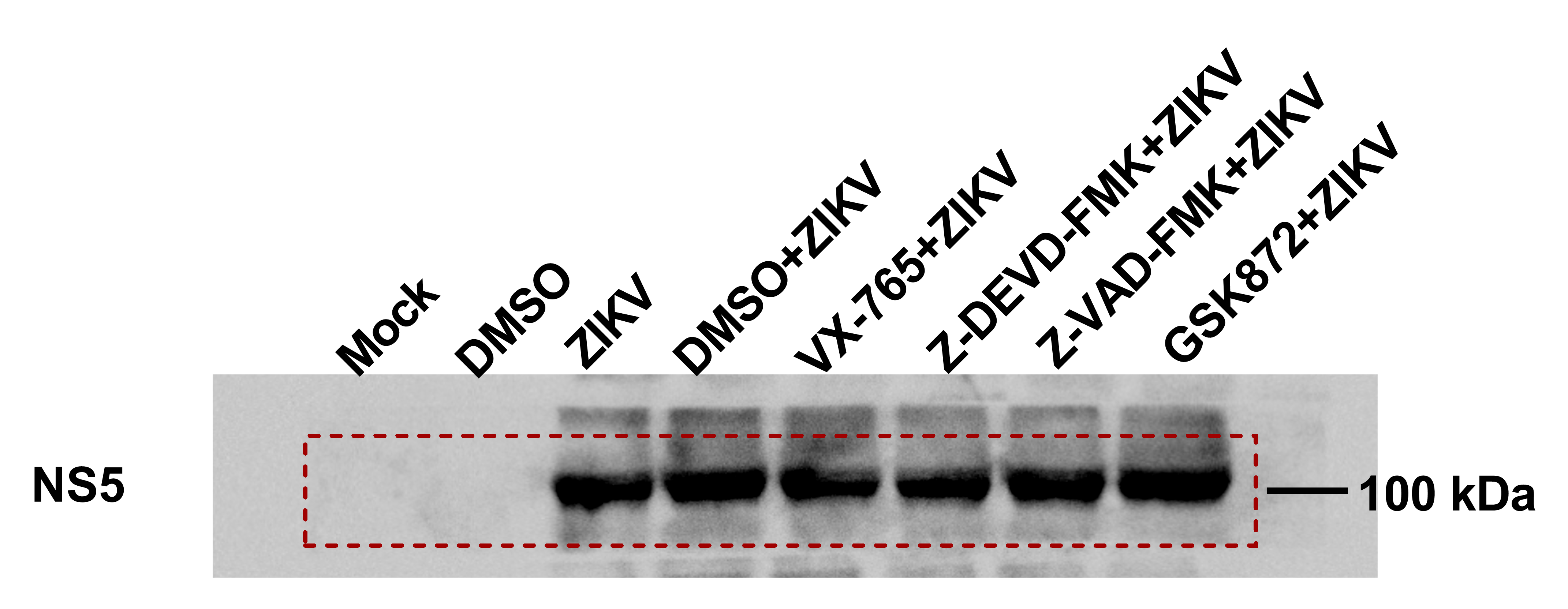

Supplement: Figure 3—source data 1. [file elife-73792-fig3-data1.zip › Figure 3-source data 1/Fig 3C/Figure 3C NS5-labeled.tif]

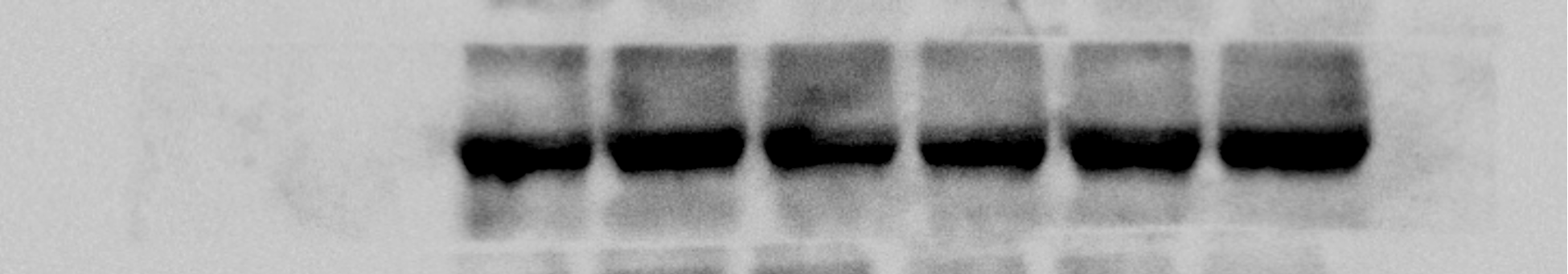

Supplement: Figure 3—source data 1. [file elife-73792-fig3-data1.zip › Figure 3-source data 1/Fig 3C/Figure 3C NS5-raw.tif]

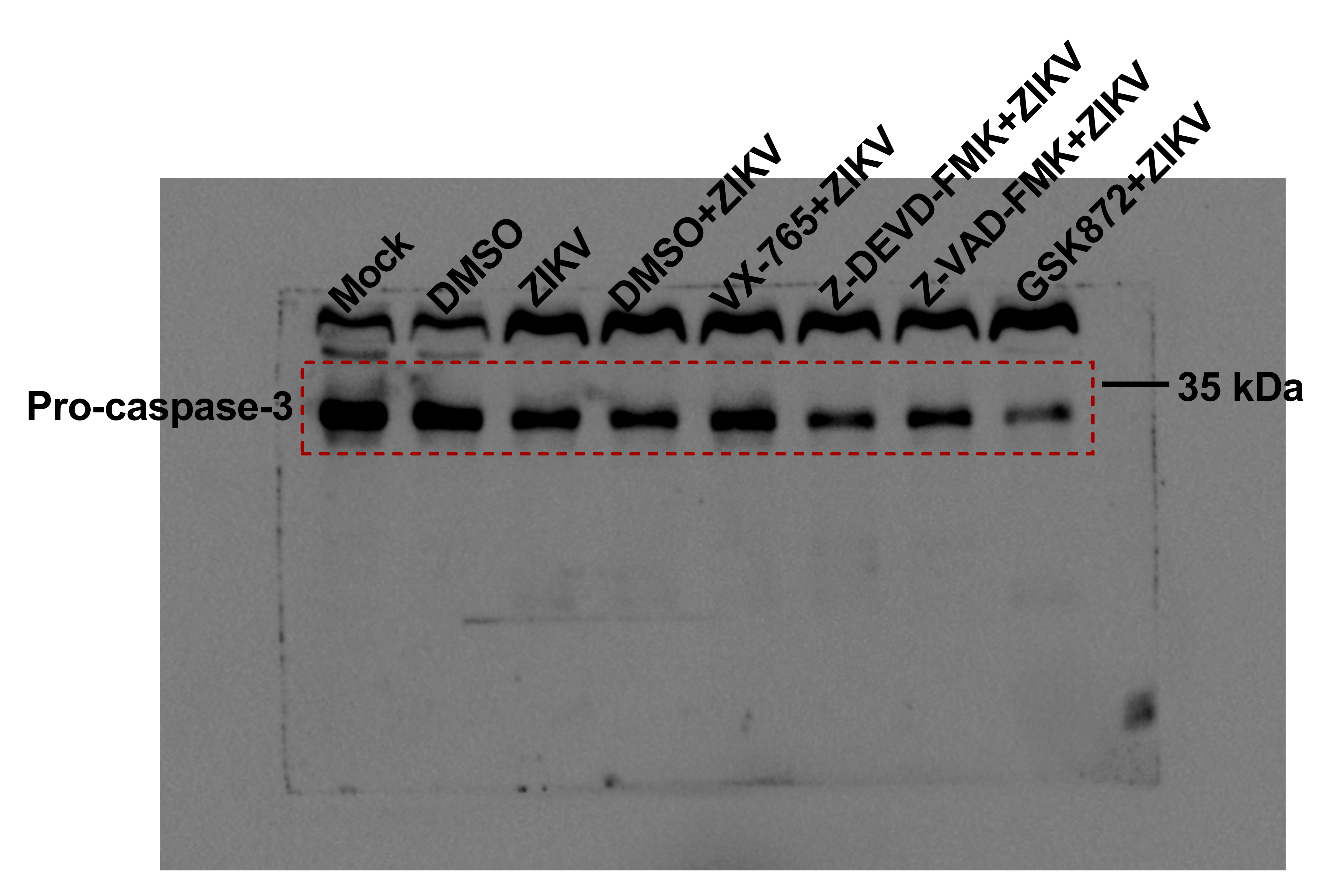

Supplement: Figure 3—source data 1. [file elife-73792-fig3-data1.zip › Figure 3-source data 1/Fig 3C/Figure 3C Pro-caspase-3-labeled.tif]

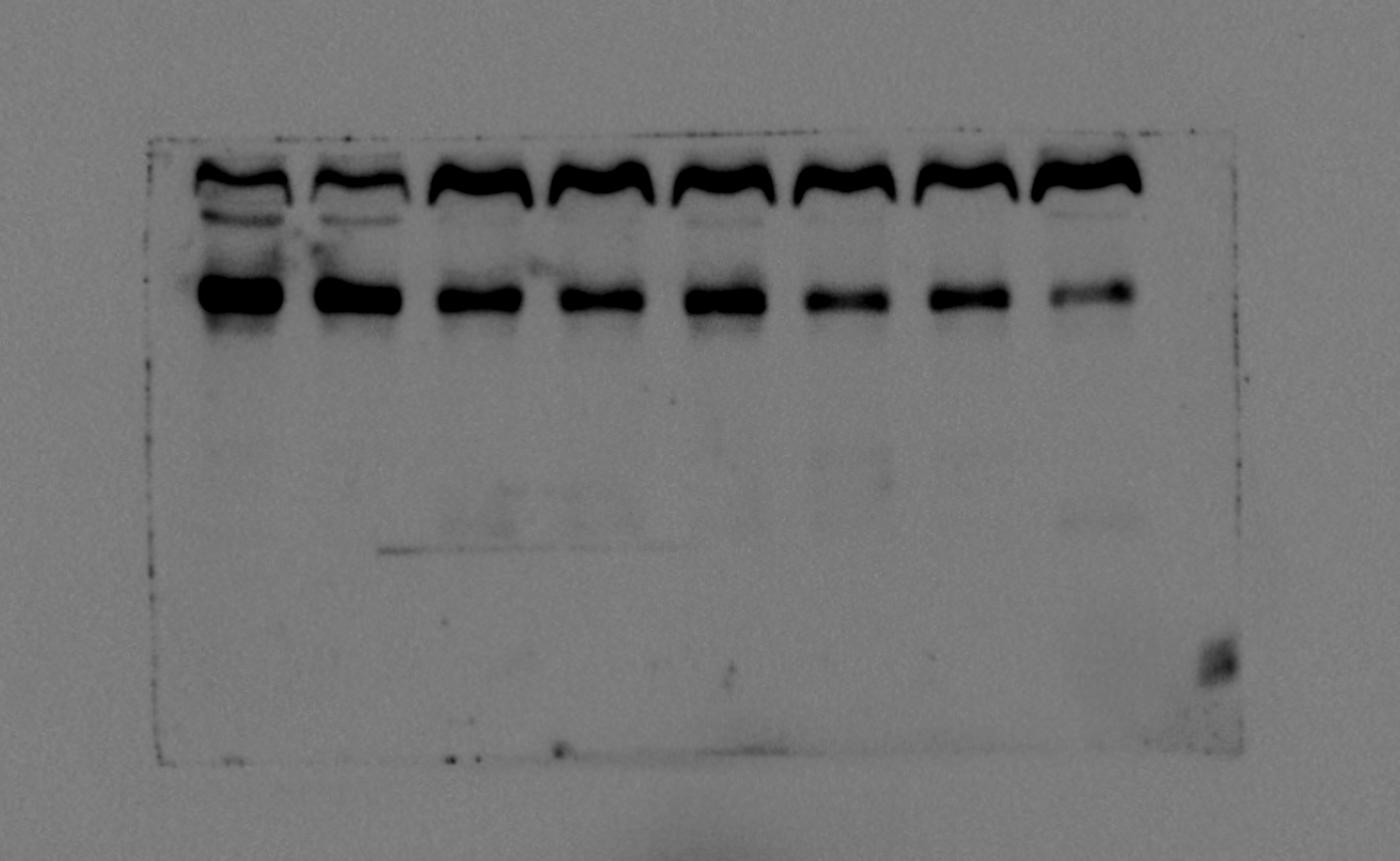

Supplement: Figure 3—source data 1. [file elife-73792-fig3-data1.zip › Figure 3-source data 1/Fig 3C/Figure 3C Pro-caspase-3-raw.tif]

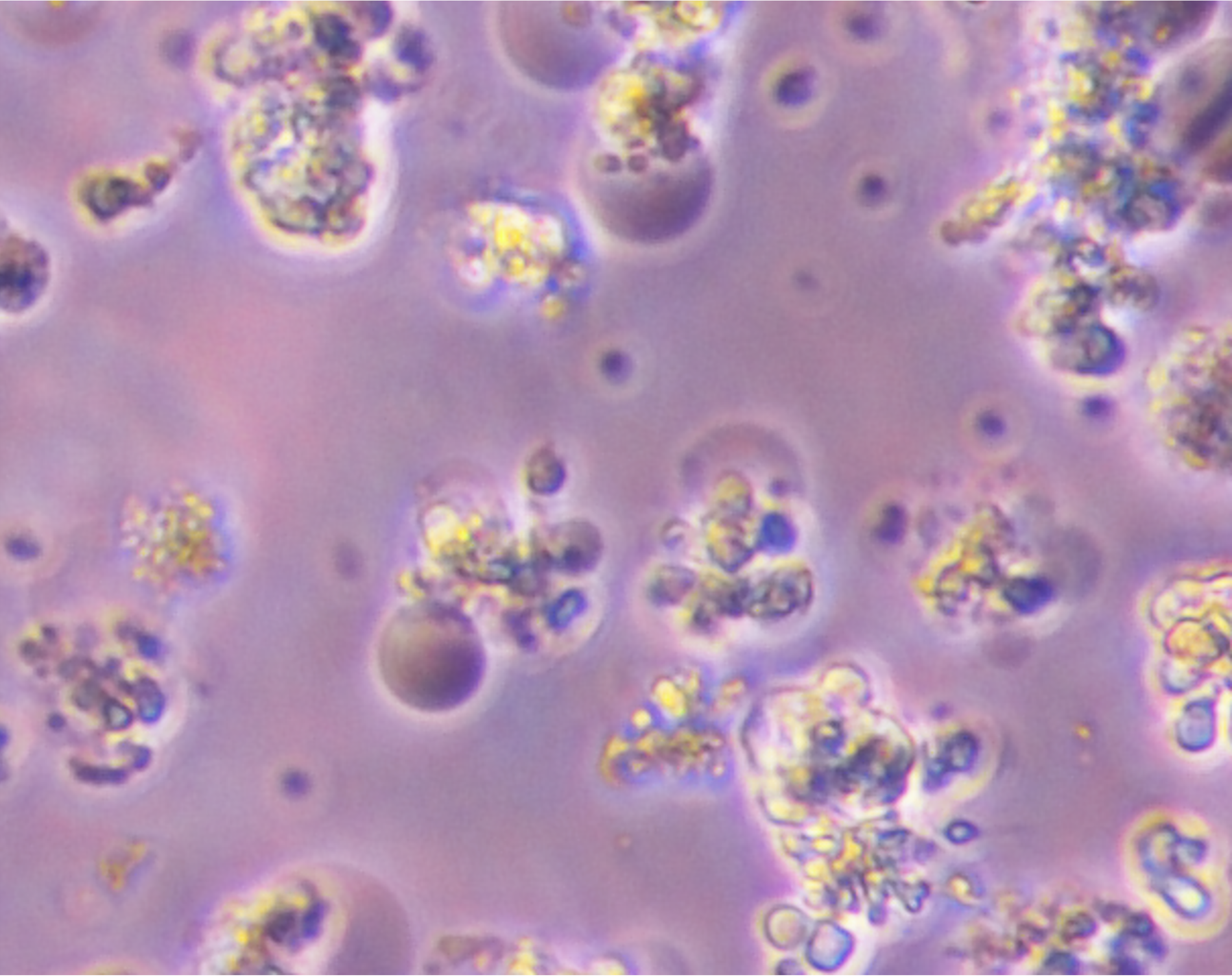

Supplement: Figure 3—source data 1. [file elife-73792-fig3-data1.zip › Figure 3-source data 1/fig 3D/dmso zikv.tif]

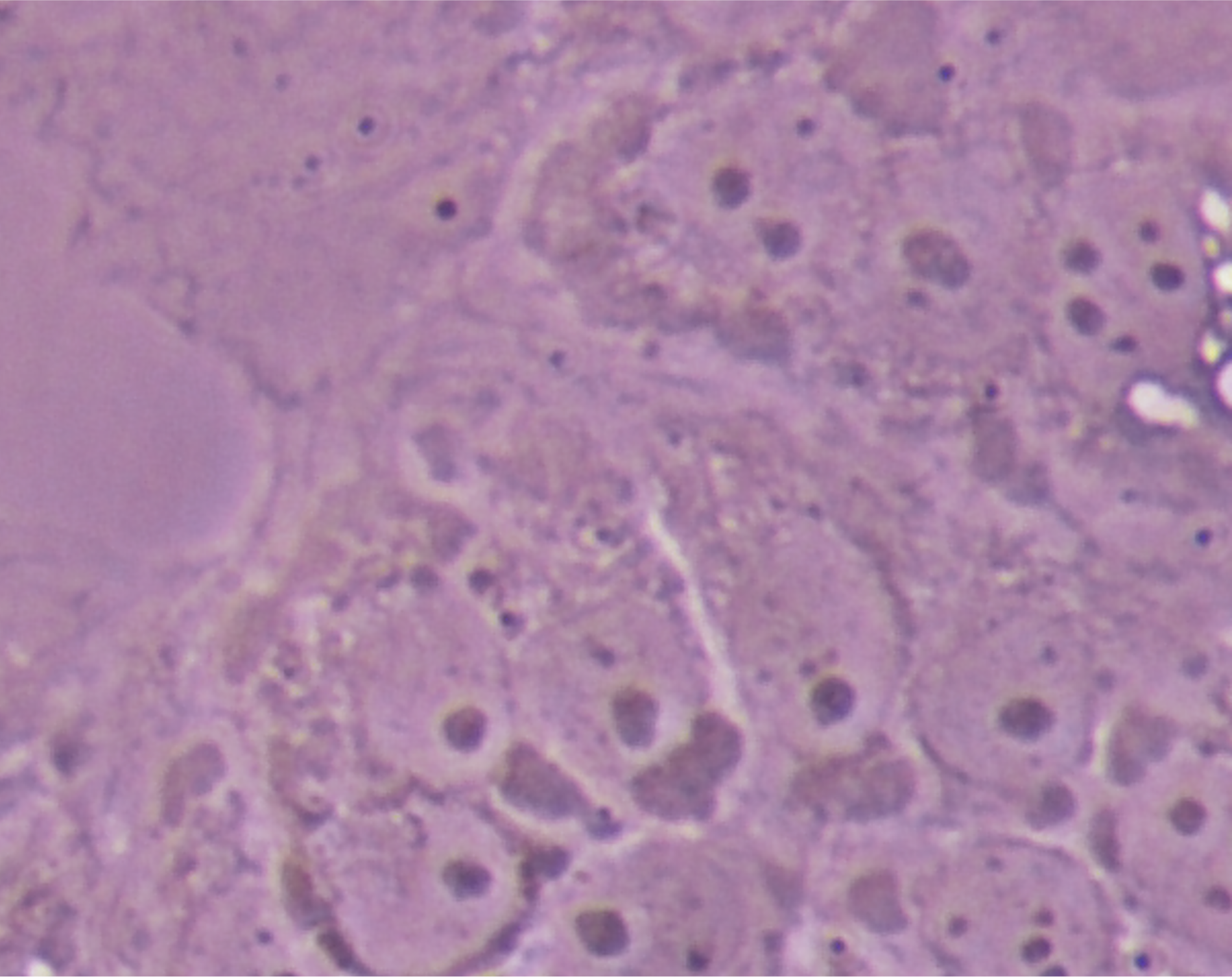

Supplement: Figure 3—source data 1. [file elife-73792-fig3-data1.zip › Figure 3-source data 1/fig 3D/dmso.tif]

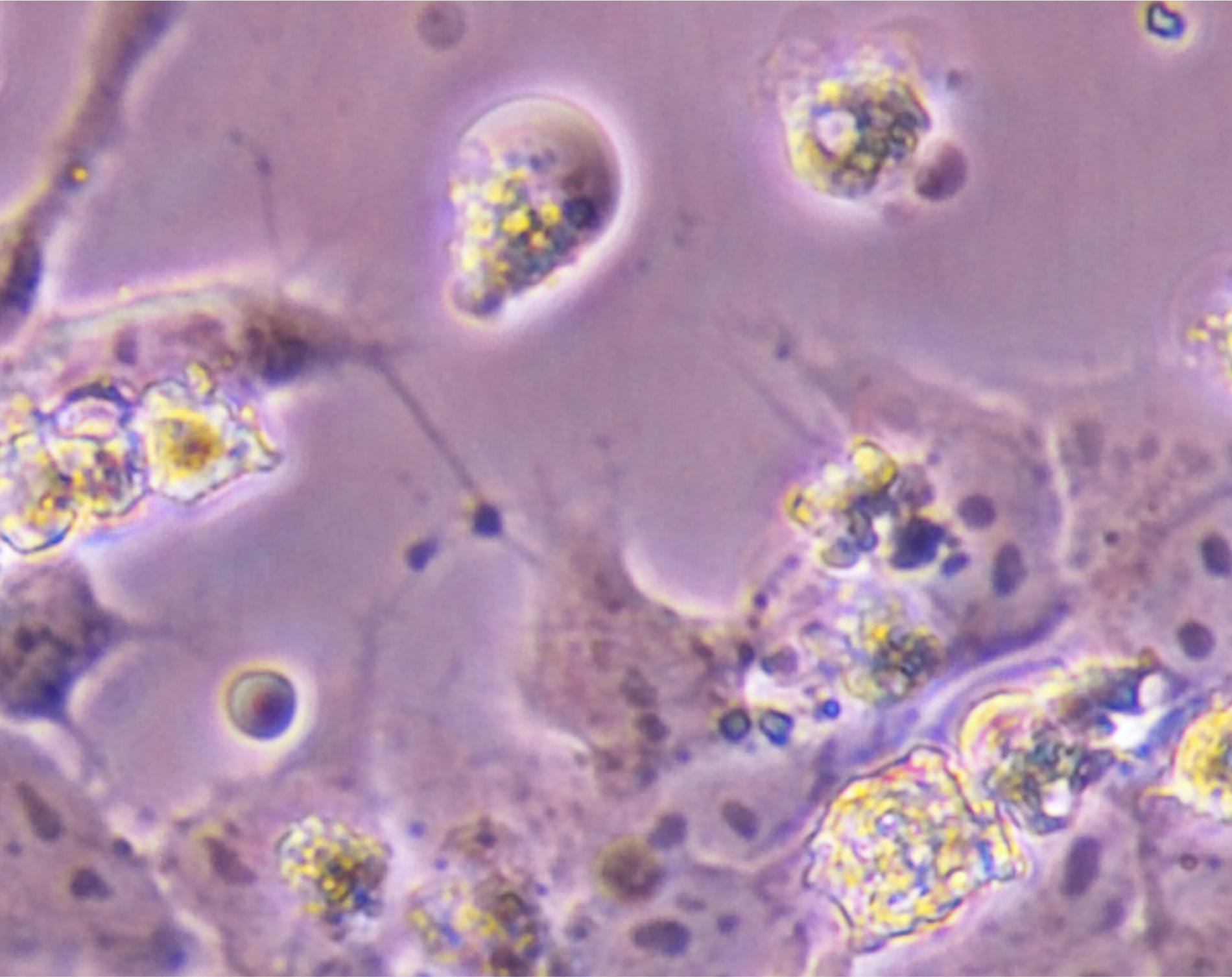

Supplement: Figure 3—source data 1. [file elife-73792-fig3-data1.zip › Figure 3-source data 1/fig 3D/ietd zikv.tif]

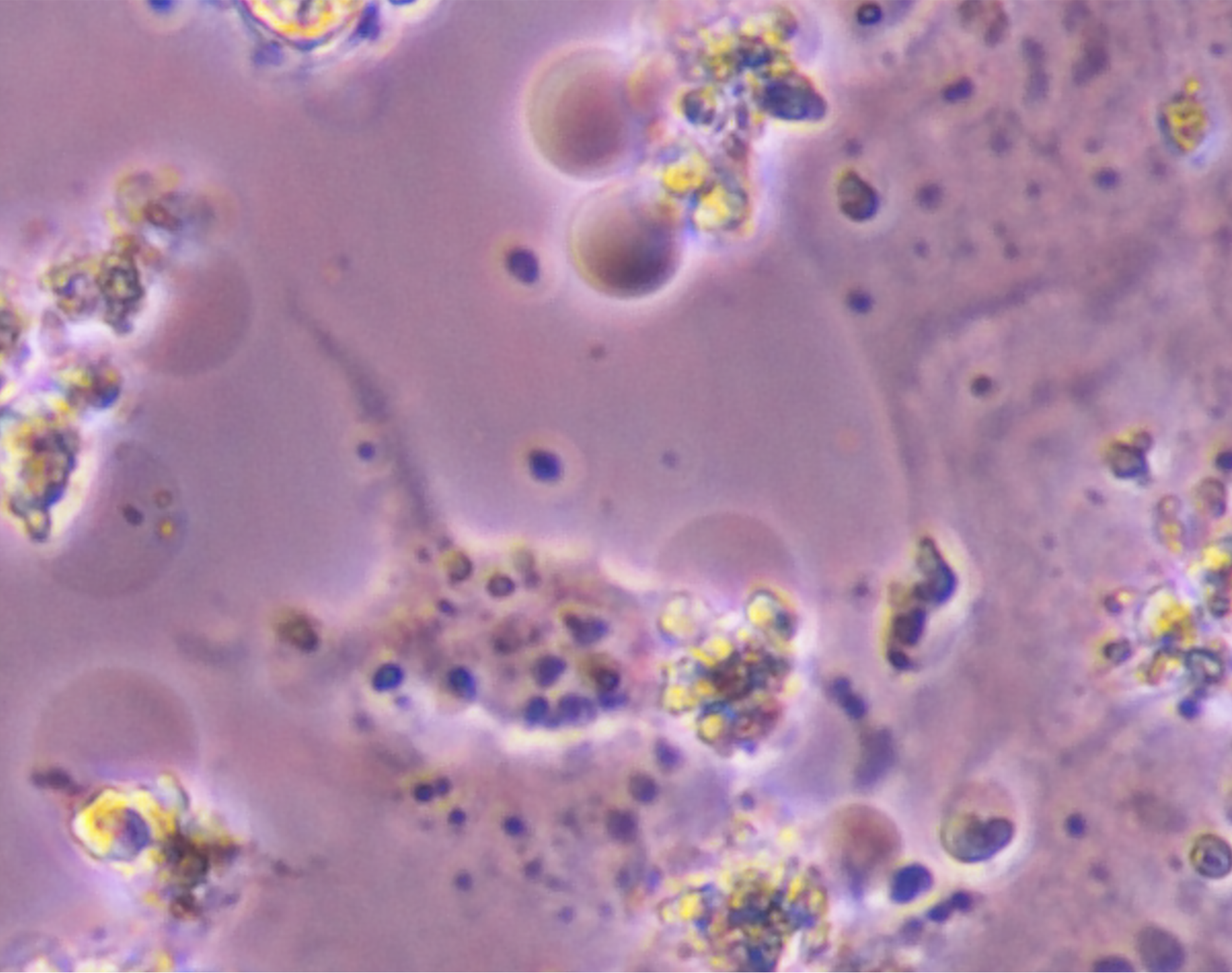

Supplement: Figure 3—source data 1. [file elife-73792-fig3-data1.zip › Figure 3-source data 1/fig 3D/lehd zikv.tif]

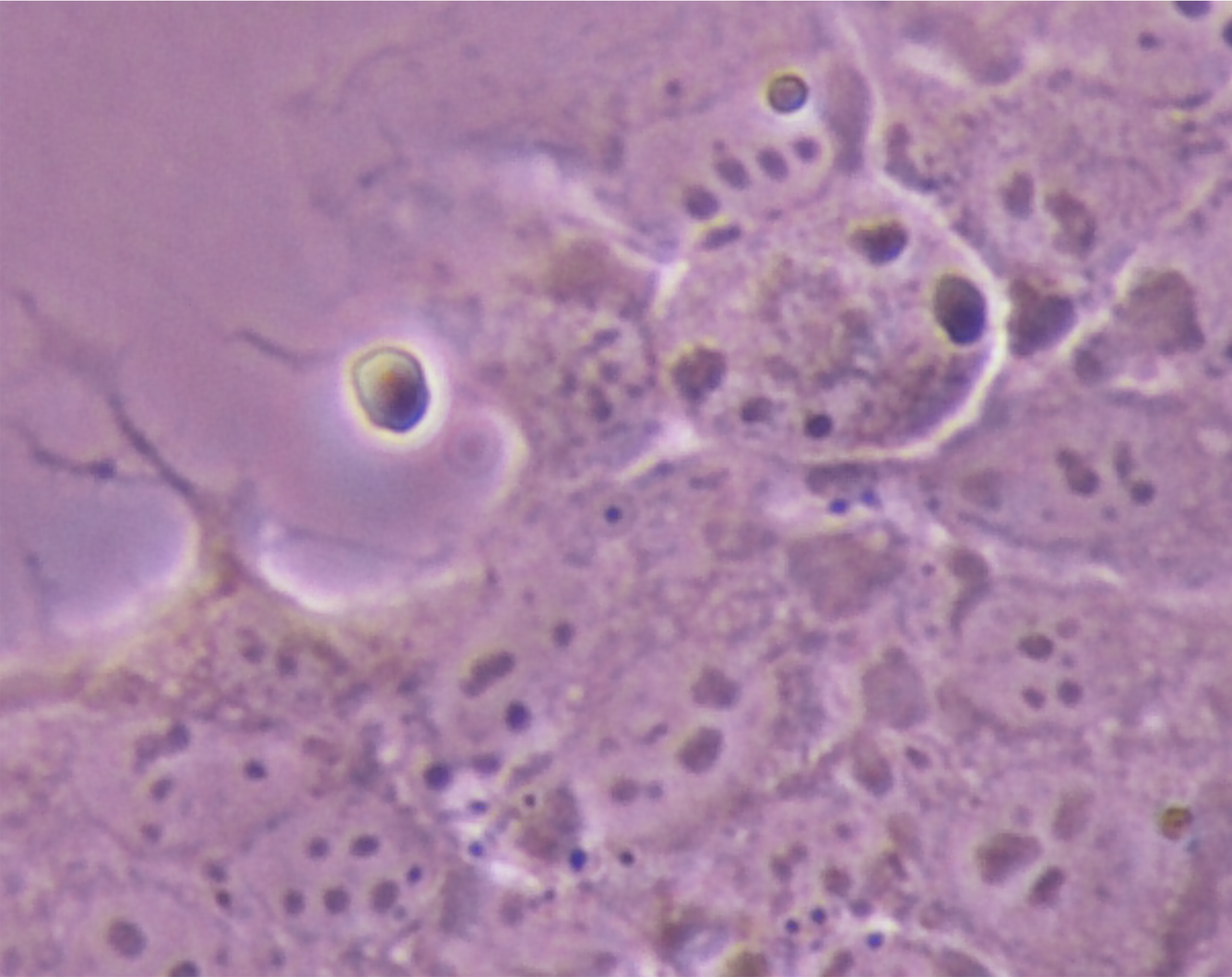

Supplement: Figure 3—source data 1. [file elife-73792-fig3-data1.zip › Figure 3-source data 1/fig 3D/mock.tif]

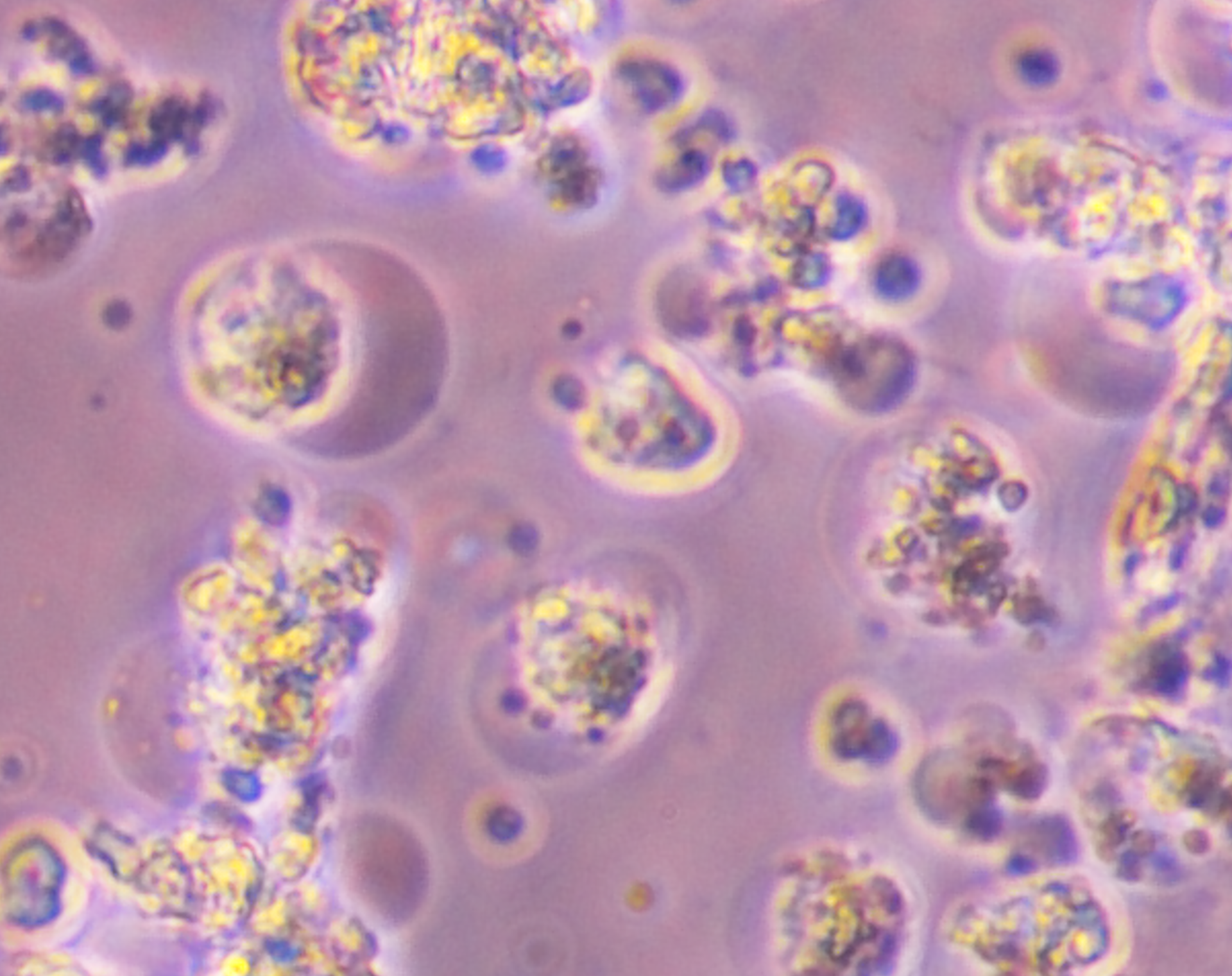

Supplement: Figure 3—source data 1. [file elife-73792-fig3-data1.zip › Figure 3-source data 1/fig 3D/zikv.tif]

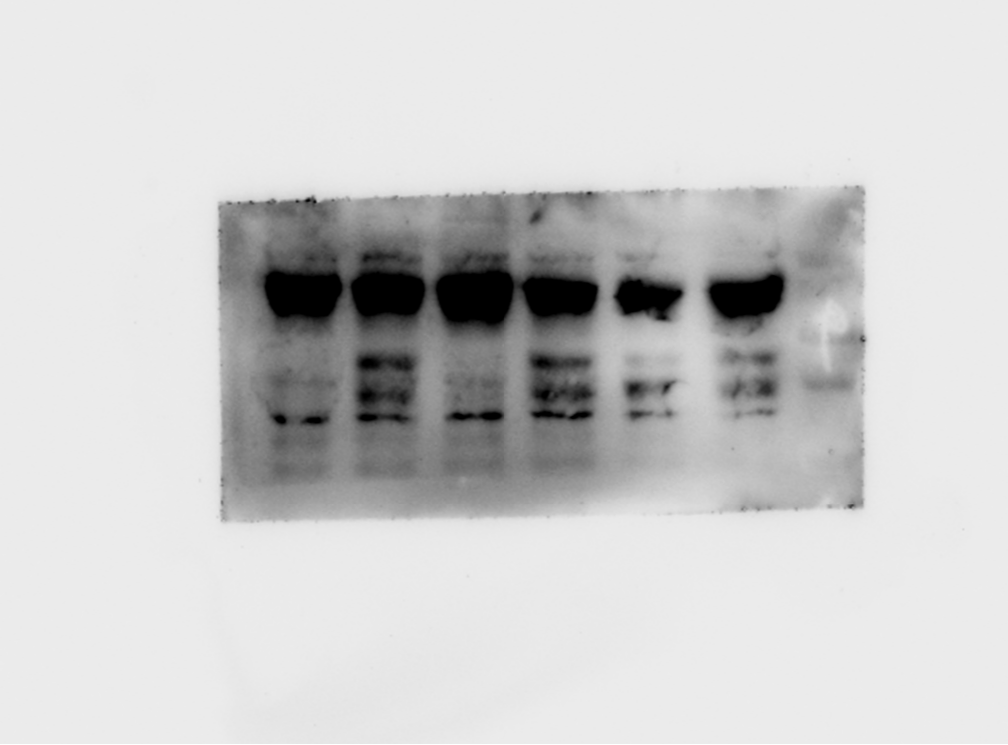

Supplement: Figure 3—source data 1. [file elife-73792-fig3-data1.zip › Figure 3-source data 1/Fig 3F/caspase9 cl.tif]

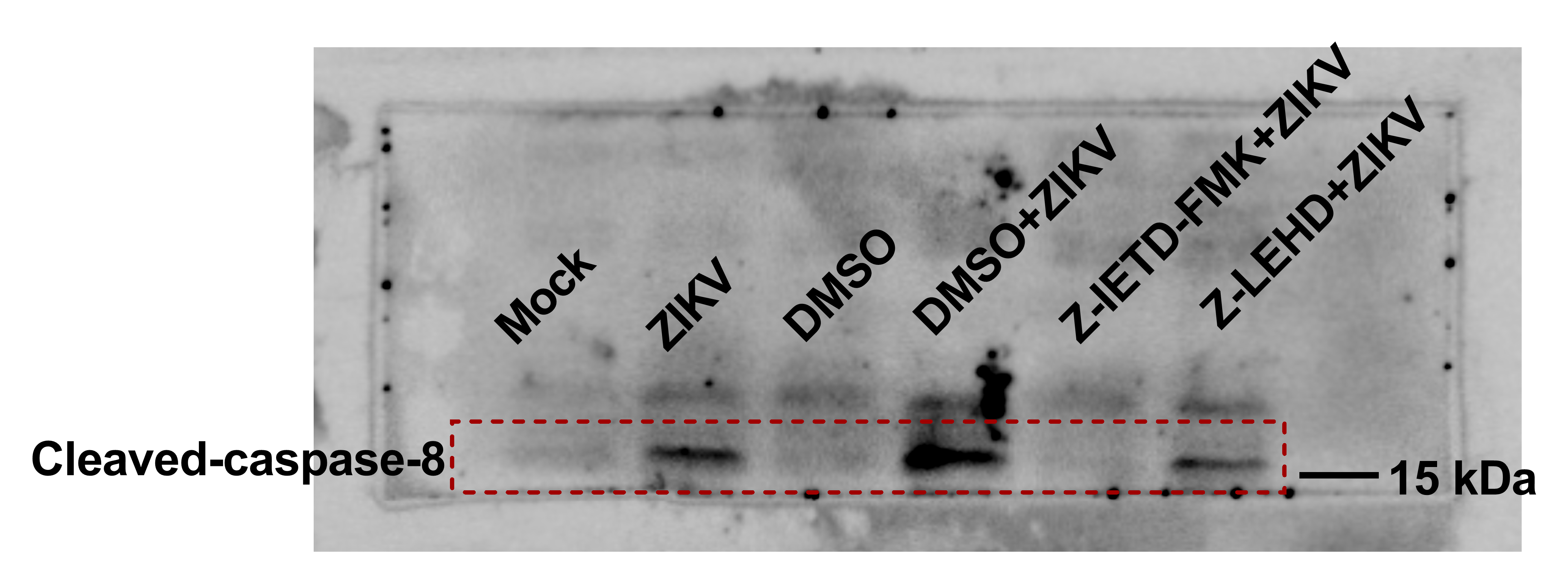

Supplement: Figure 3—source data 1. [file elife-73792-fig3-data1.zip › Figure 3-source data 1/Fig 3F/Figure 3F Cleaved-caspase-8-labeled.tif]

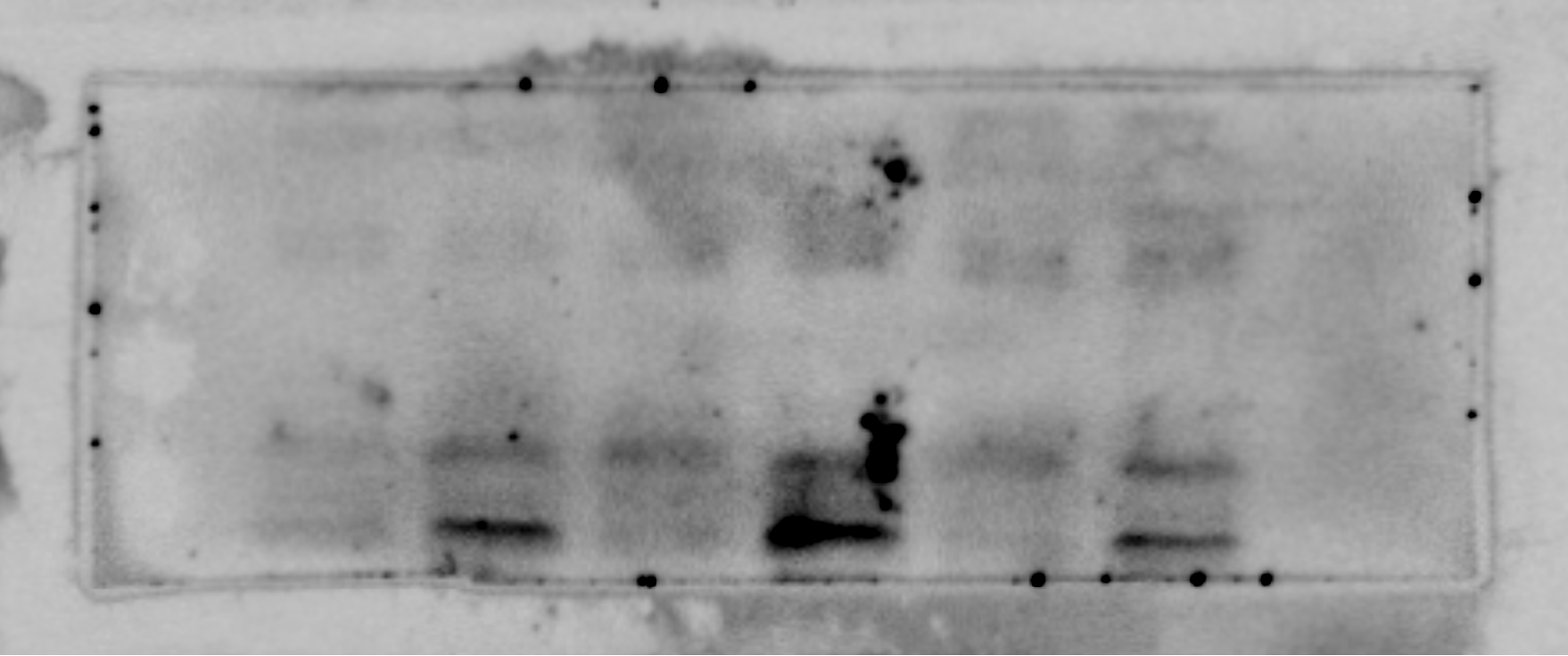

Supplement: Figure 3—source data 1. [file elife-73792-fig3-data1.zip › Figure 3-source data 1/Fig 3F/Figure 3F Cleaved-caspase-8-raw.tif]

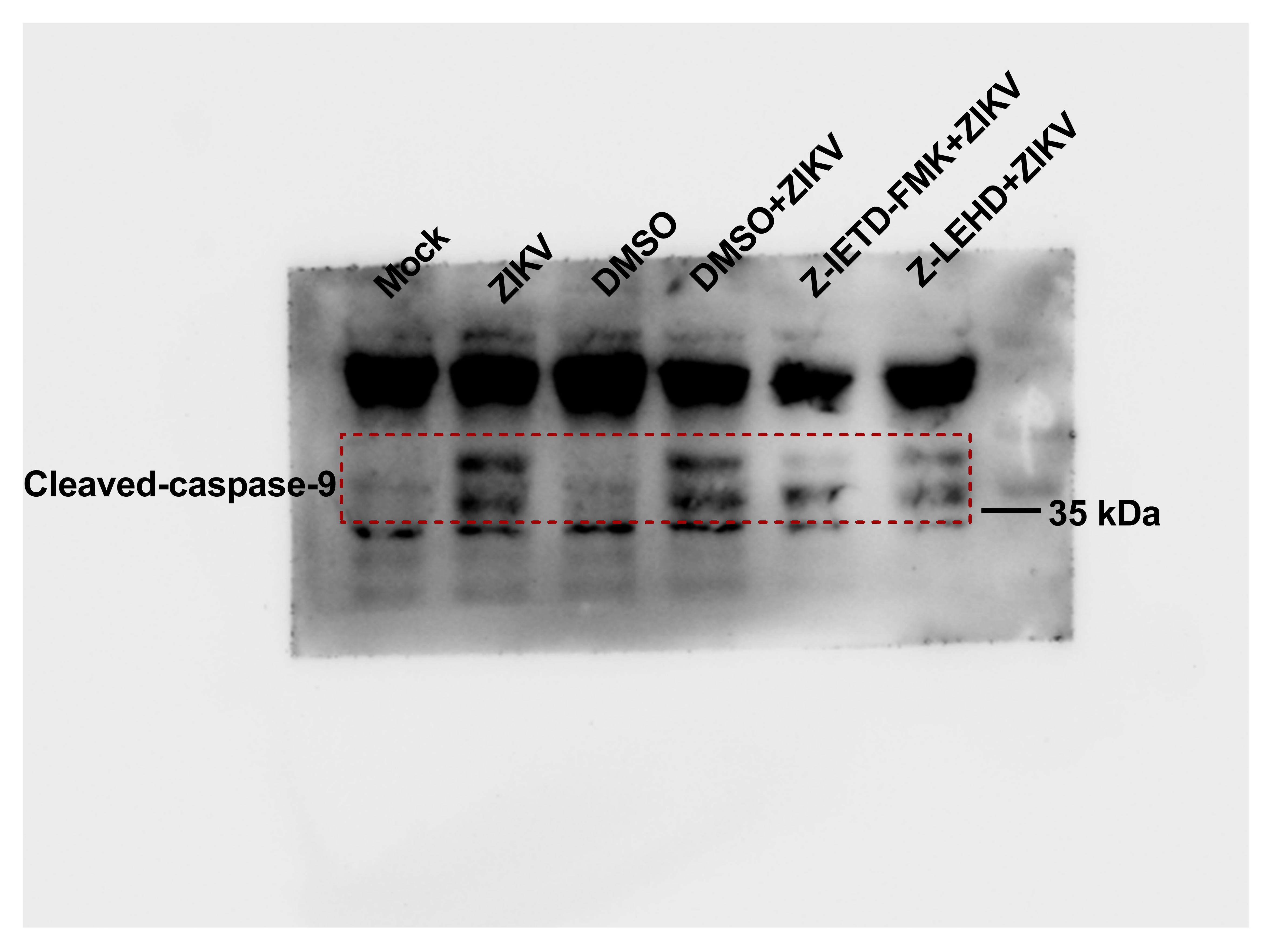

Supplement: Figure 3—source data 1. [file elife-73792-fig3-data1.zip › Figure 3-source data 1/Fig 3F/Figure 3F Cleaved-caspase-9-labeled.tif]

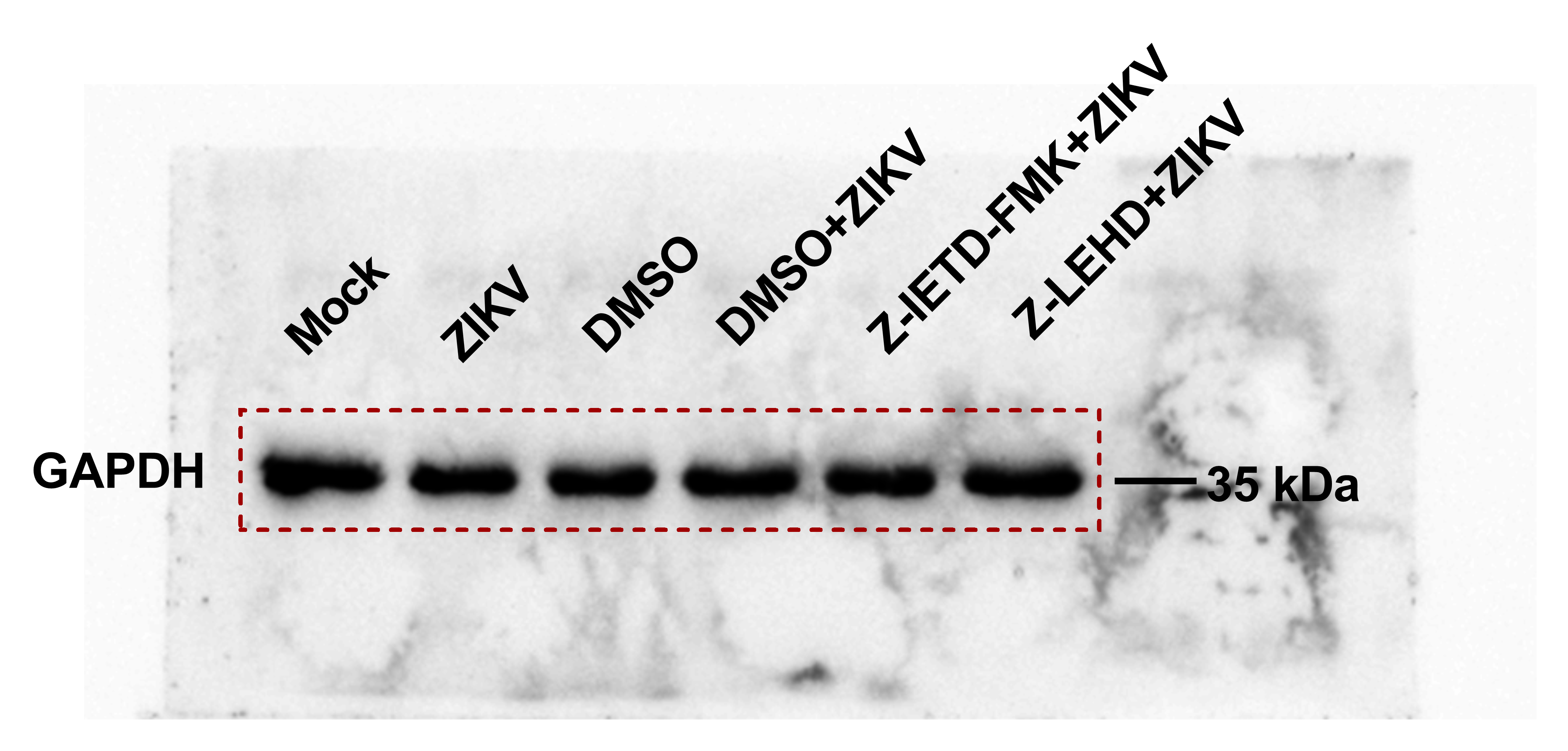

Supplement: Figure 3—source data 1. [file elife-73792-fig3-data1.zip › Figure 3-source data 1/Fig 3F/Figure 3F GAPDH-labeled.tif]

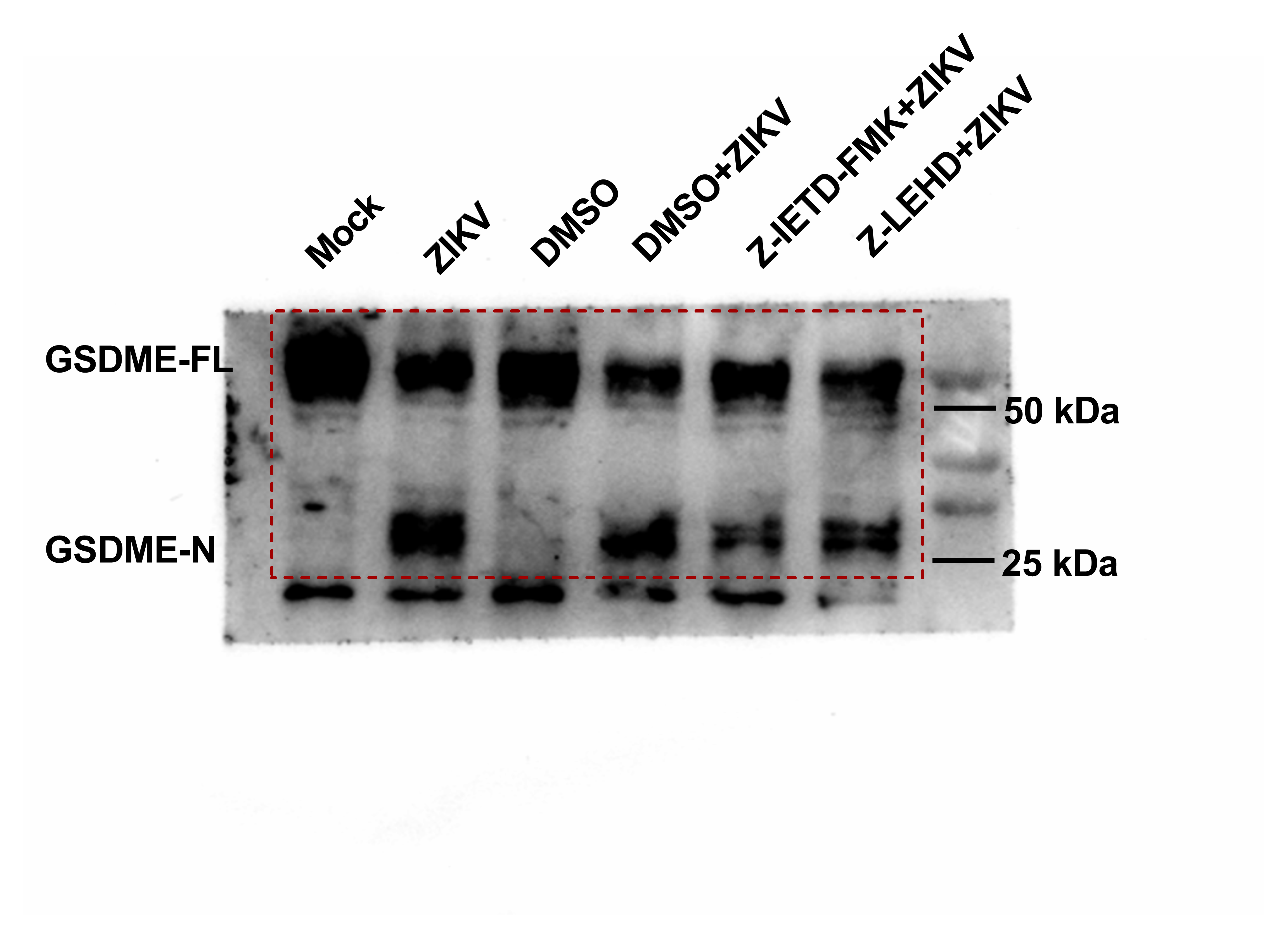

Supplement: Figure 3—source data 1. [file elife-73792-fig3-data1.zip › Figure 3-source data 1/Fig 3F/Figure 3F GSDME-labeled.tif]

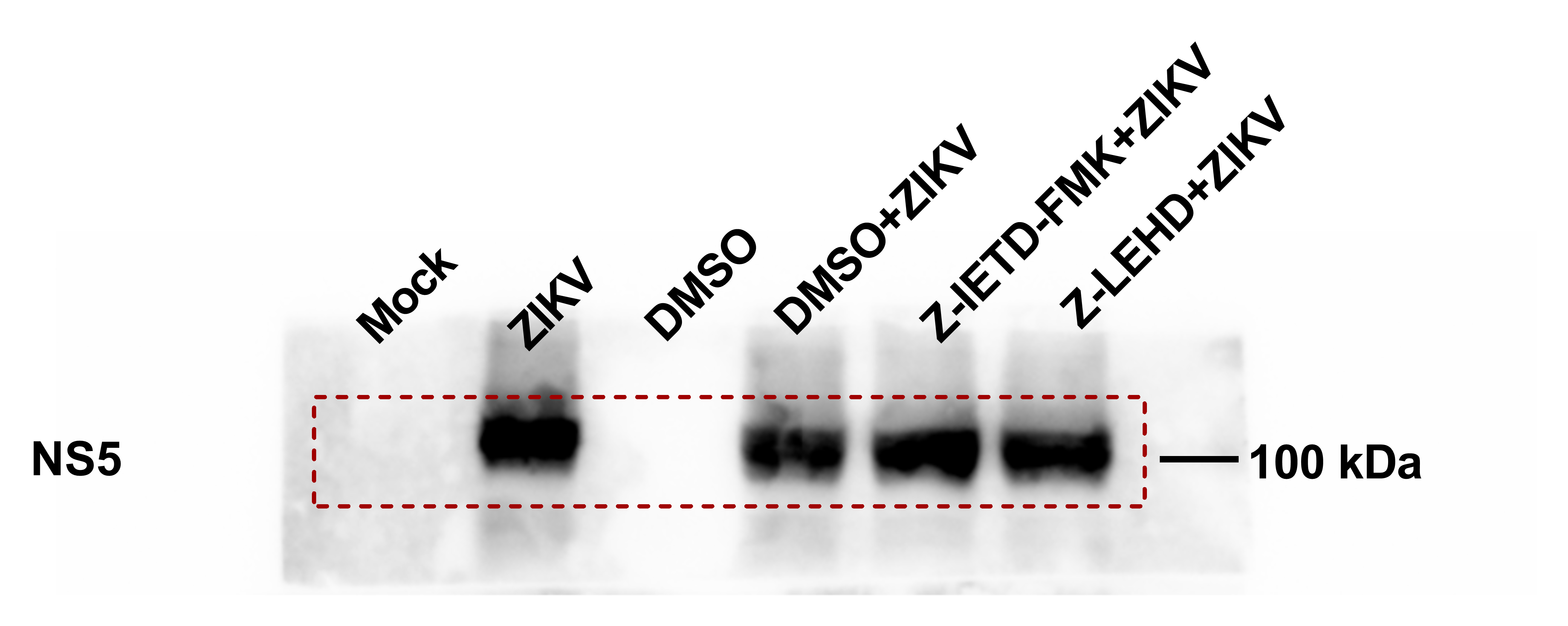

Supplement: Figure 3—source data 1. [file elife-73792-fig3-data1.zip › Figure 3-source data 1/Fig 3F/Figure 3F NS5-labeled.tif]

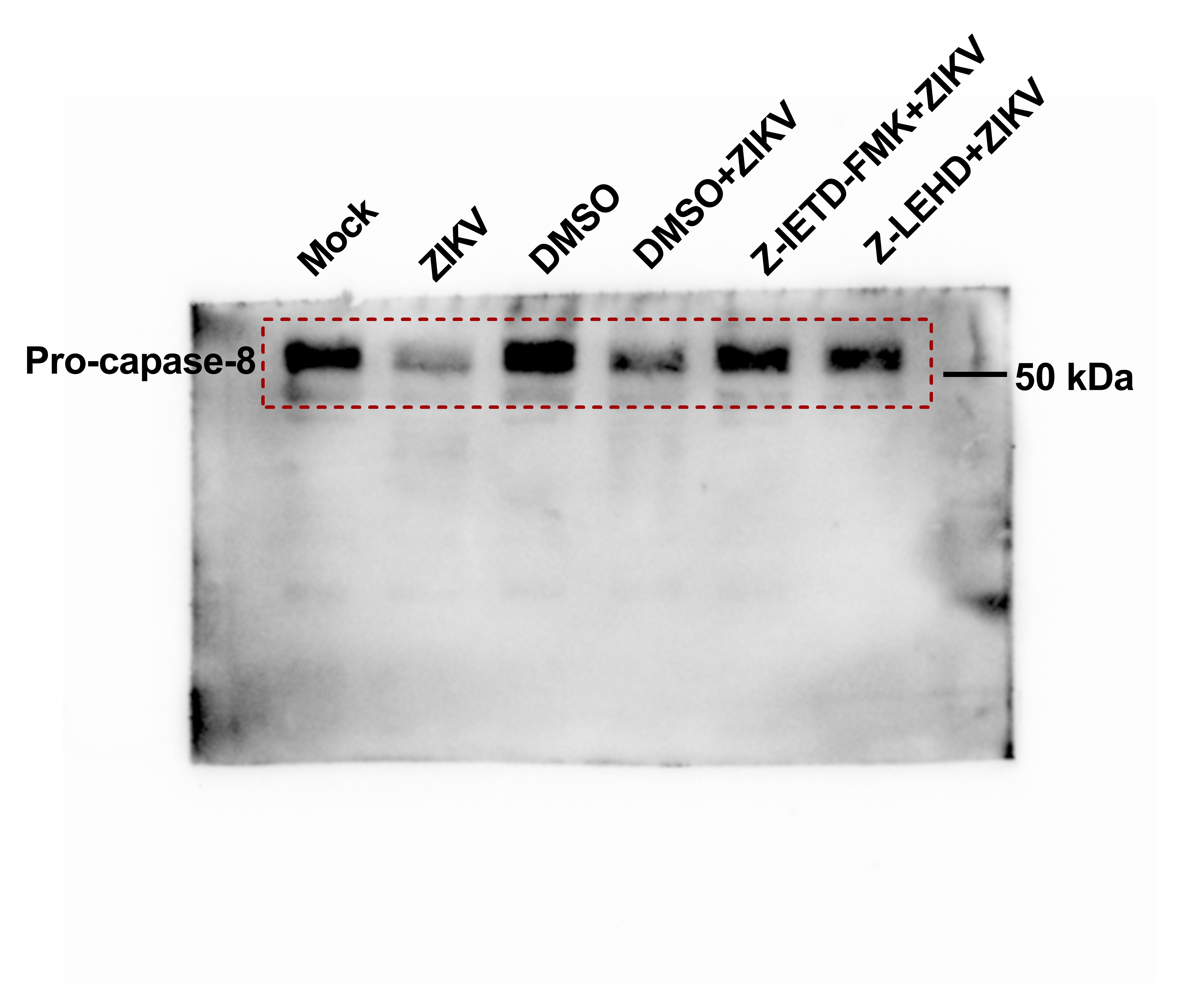

Supplement: Figure 3—source data 1. [file elife-73792-fig3-data1.zip › Figure 3-source data 1/Fig 3F/Figure 3F Pro-caspase-8-labeled.tif]

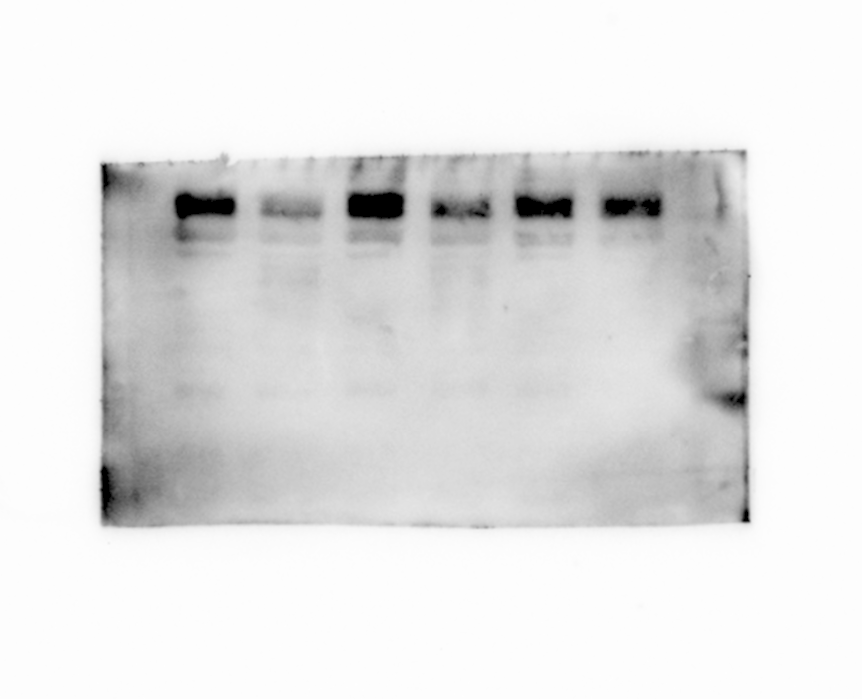

Supplement: Figure 3—source data 1. [file elife-73792-fig3-data1.zip › Figure 3-source data 1/Fig 3F/Figure 3F Pro-caspase-8-raw.tif]

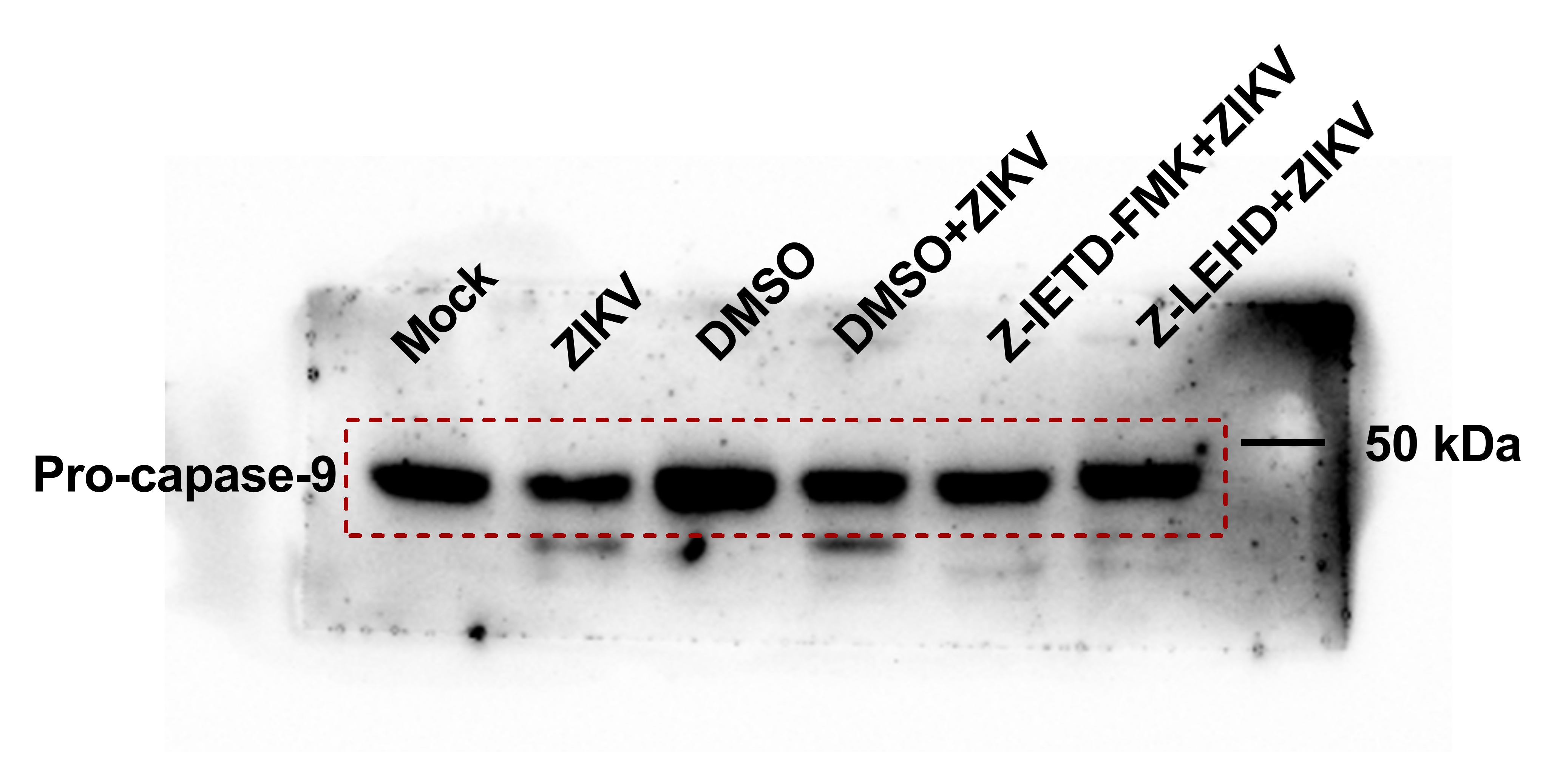

Supplement: Figure 3—source data 1. [file elife-73792-fig3-data1.zip › Figure 3-source data 1/Fig 3F/Figure 3F Pro-caspase-9-labeled.tif]

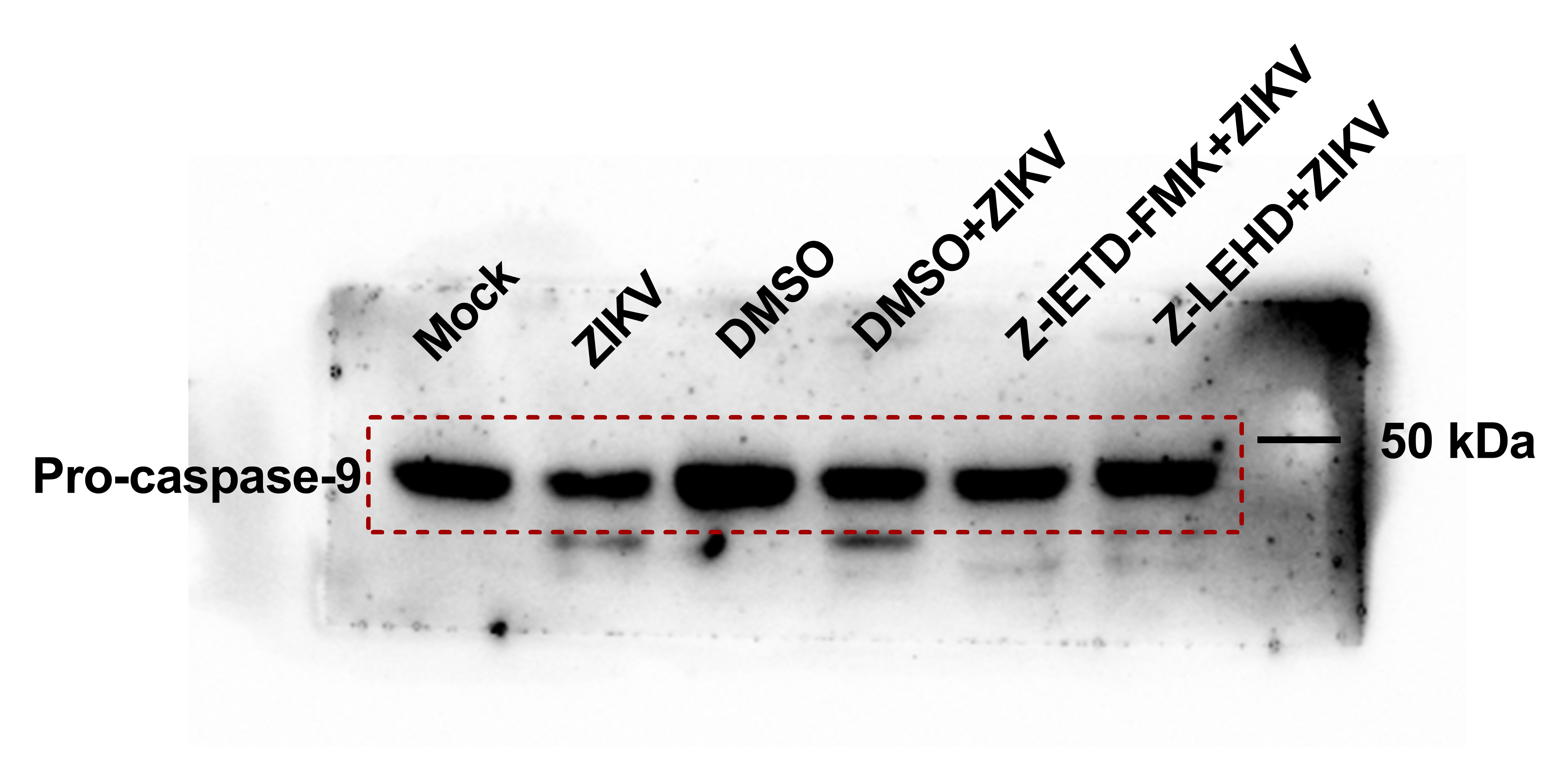

Supplement: Figure 3—source data 1. [file elife-73792-fig3-data1.zip › Figure 3-source data 1/Fig 3F/Figure 3F Pro-caspase-9-raw.tif]

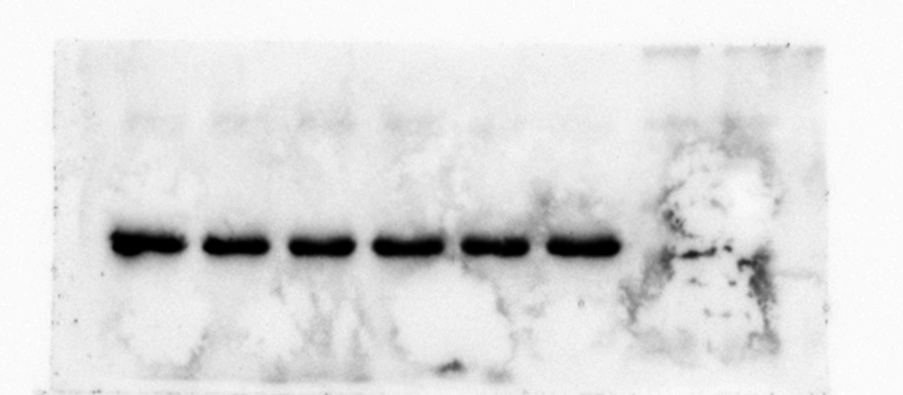

Supplement: Figure 3—source data 1. [file elife-73792-fig3-data1.zip › Figure 3-source data 1/Fig 3F/GAPDH.tif]

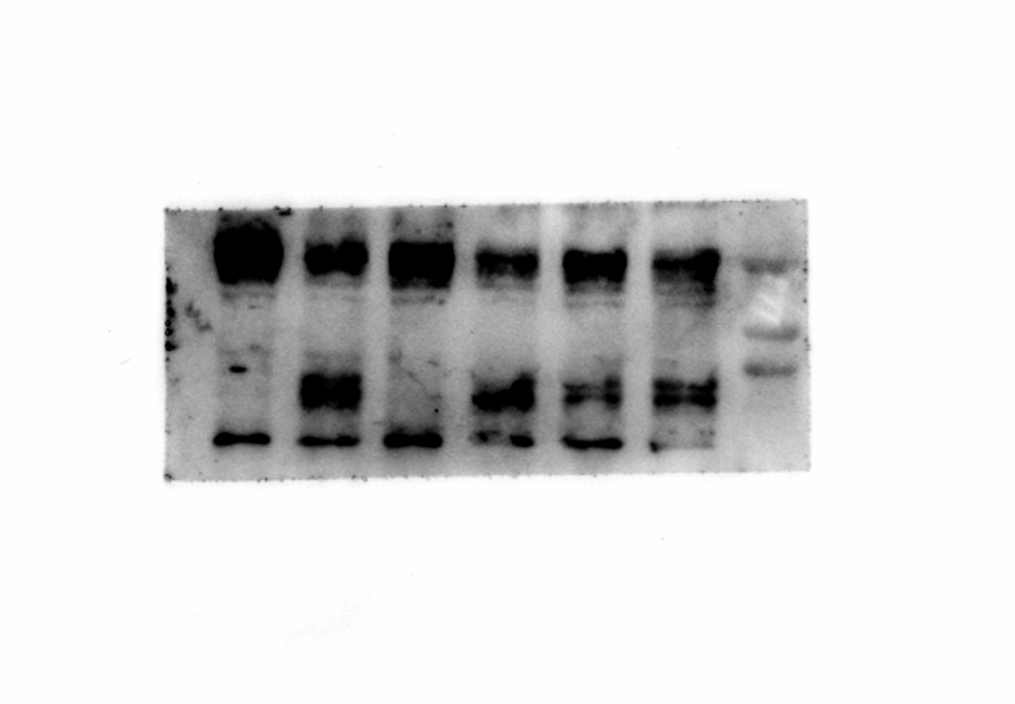

Supplement: Figure 3—source data 1. [file elife-73792-fig3-data1.zip › Figure 3-source data 1/Fig 3F/GSDME.tif]

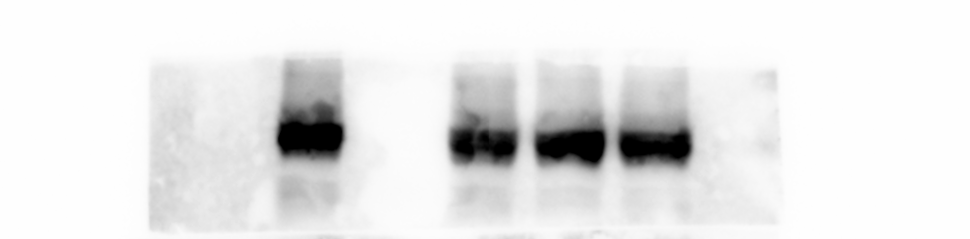

Supplement: Figure 3—source data 1. [file elife-73792-fig3-data1.zip › Figure 3-source data 1/Fig 3F/NS5.tif]

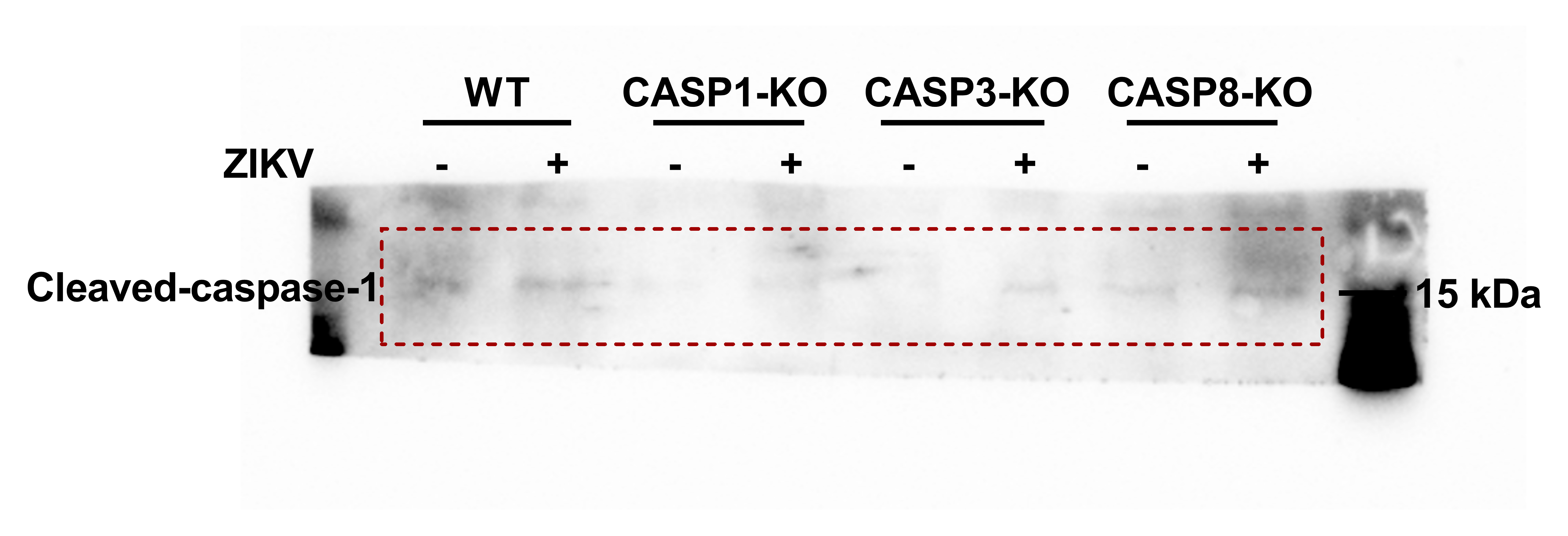

Supplement: Figure 3—source data 1. [file elife-73792-fig3-data1.zip › Figure 3-source data 1/Fig 3H/Figure 3H Cleaved-caspase-1-labeled.tif]

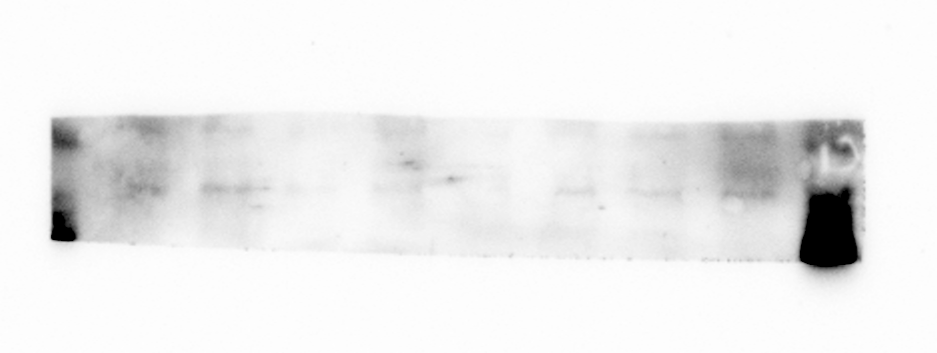

Supplement: Figure 3—source data 1. [file elife-73792-fig3-data1.zip › Figure 3-source data 1/Fig 3H/Figure 3H Cleaved-caspase-1-raw.tif]

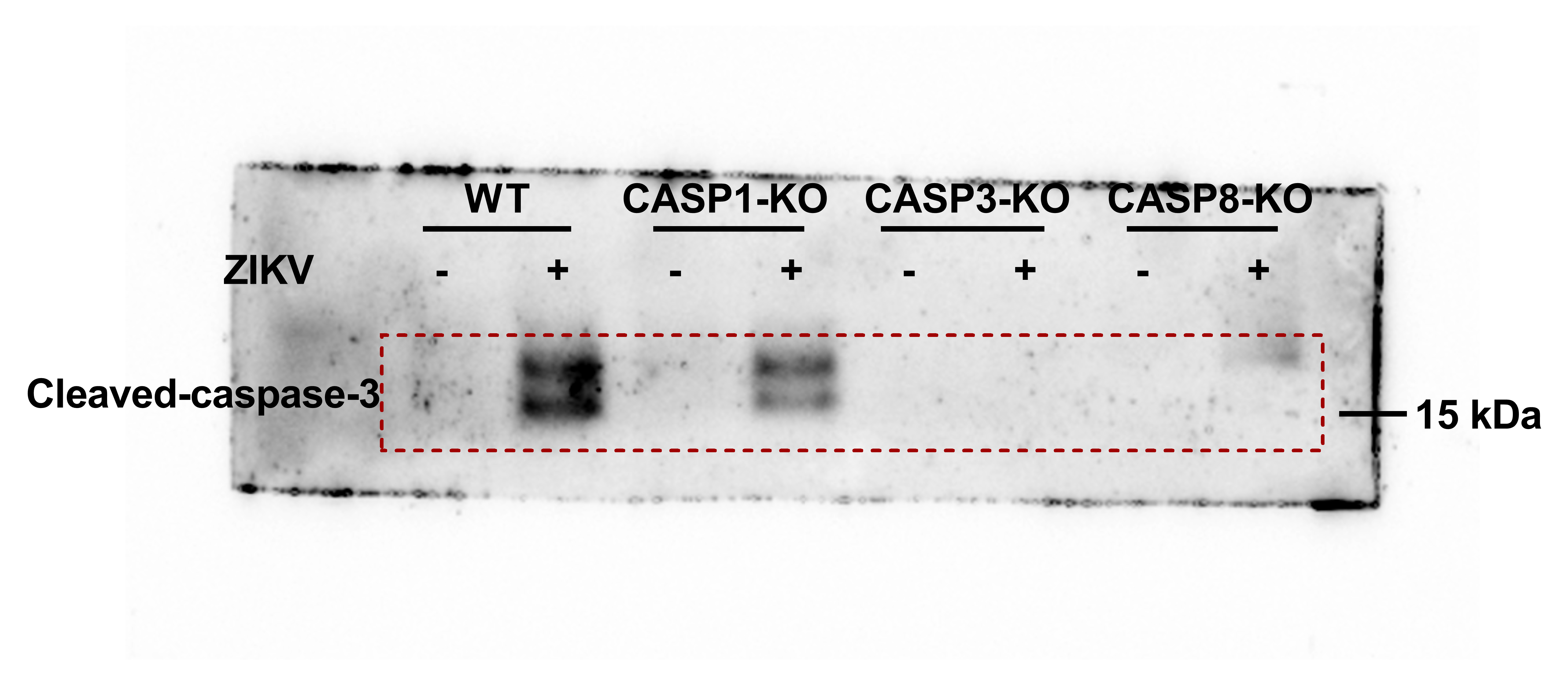

Supplement: Figure 3—source data 1. [file elife-73792-fig3-data1.zip › Figure 3-source data 1/Fig 3H/Figure 3H Cleaved-caspase-3-labeled.tif]

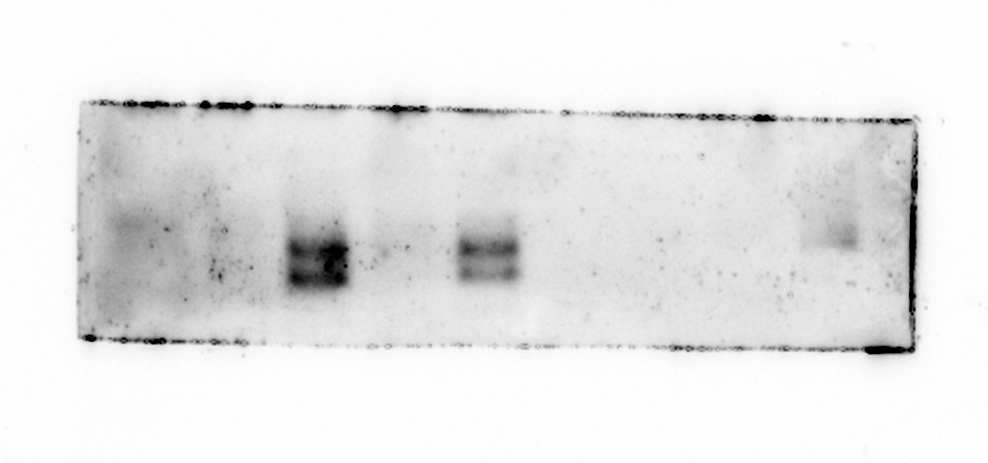

Supplement: Figure 3—source data 1. [file elife-73792-fig3-data1.zip › Figure 3-source data 1/Fig 3H/Figure 3H Cleaved-caspase-3-raw.tif]

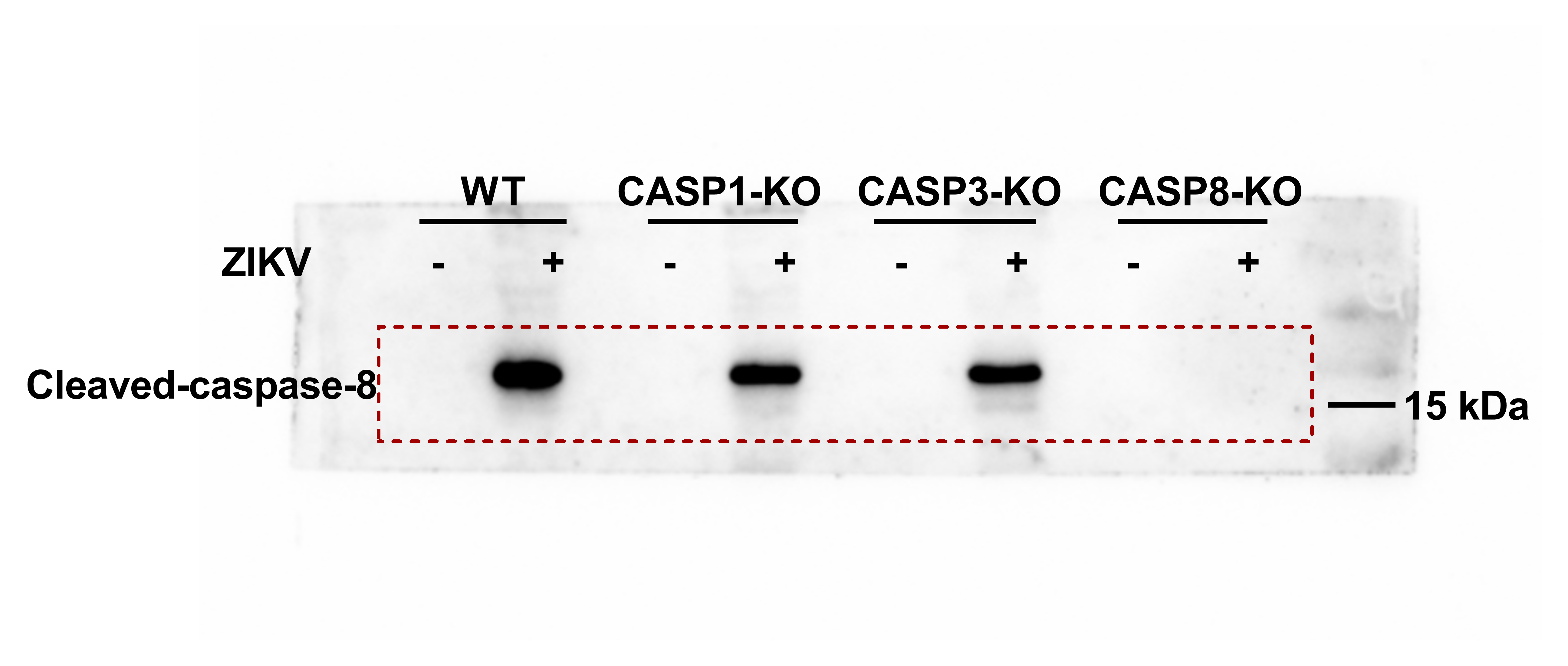

Supplement: Figure 3—source data 1. [file elife-73792-fig3-data1.zip › Figure 3-source data 1/Fig 3H/Figure 3H Cleaved-caspase-8-labeled.tif]

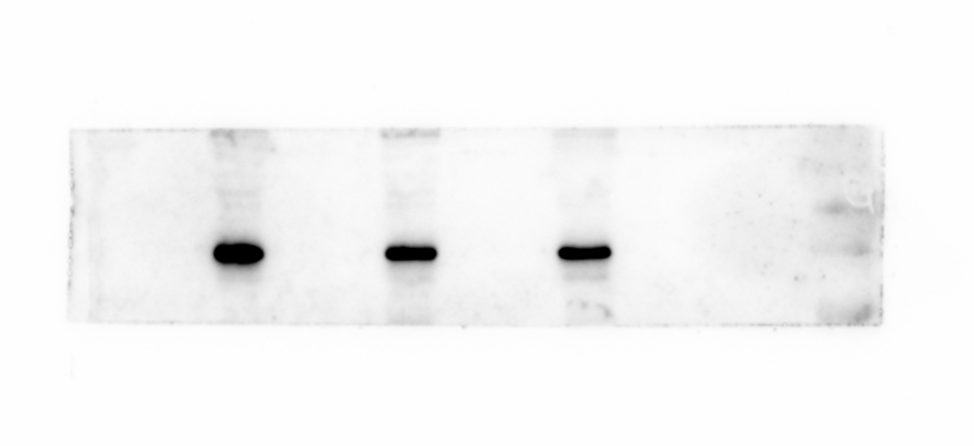

Supplement: Figure 3—source data 1. [file elife-73792-fig3-data1.zip › Figure 3-source data 1/Fig 3H/Figure 3H Cleaved-caspase-8-raw.Tif]

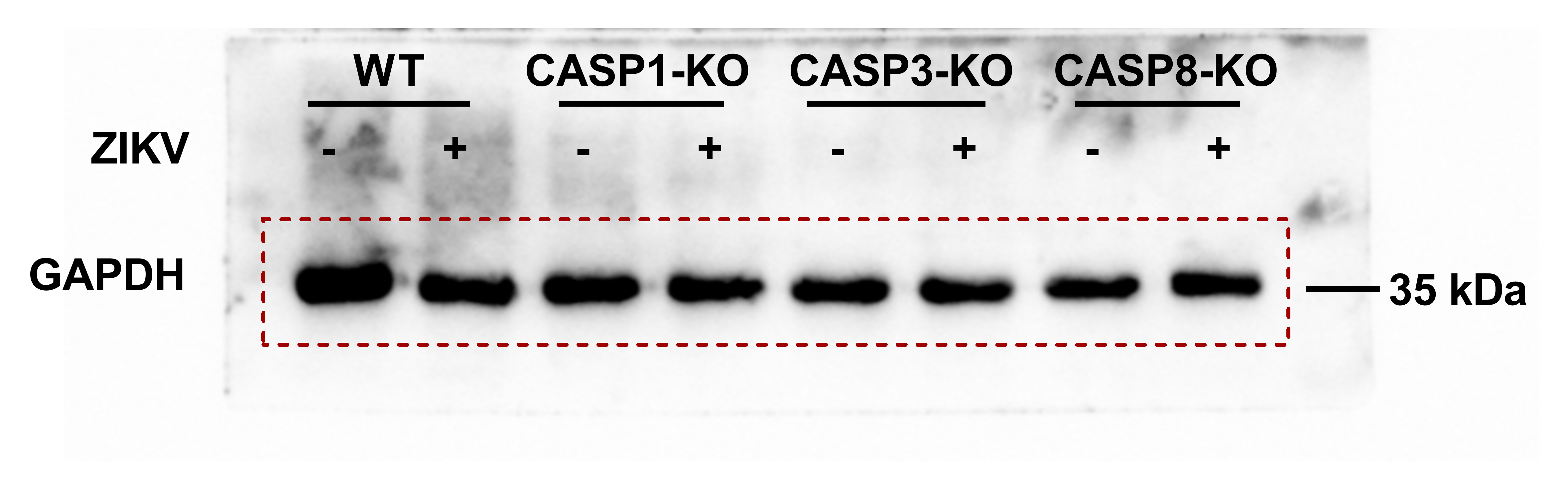

Supplement: Figure 3—source data 1. [file elife-73792-fig3-data1.zip › Figure 3-source data 1/Fig 3H/Figure 3H GAPDH-labeled.tif]

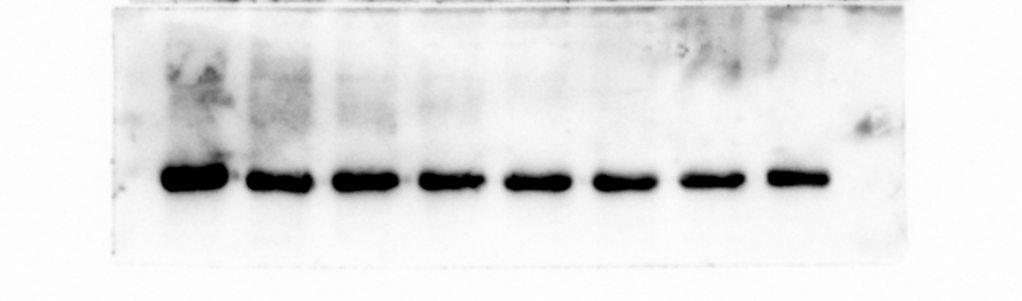

Supplement: Figure 3—source data 1. [file elife-73792-fig3-data1.zip › Figure 3-source data 1/Fig 3H/Figure 3H GAPDH-raw.tif]

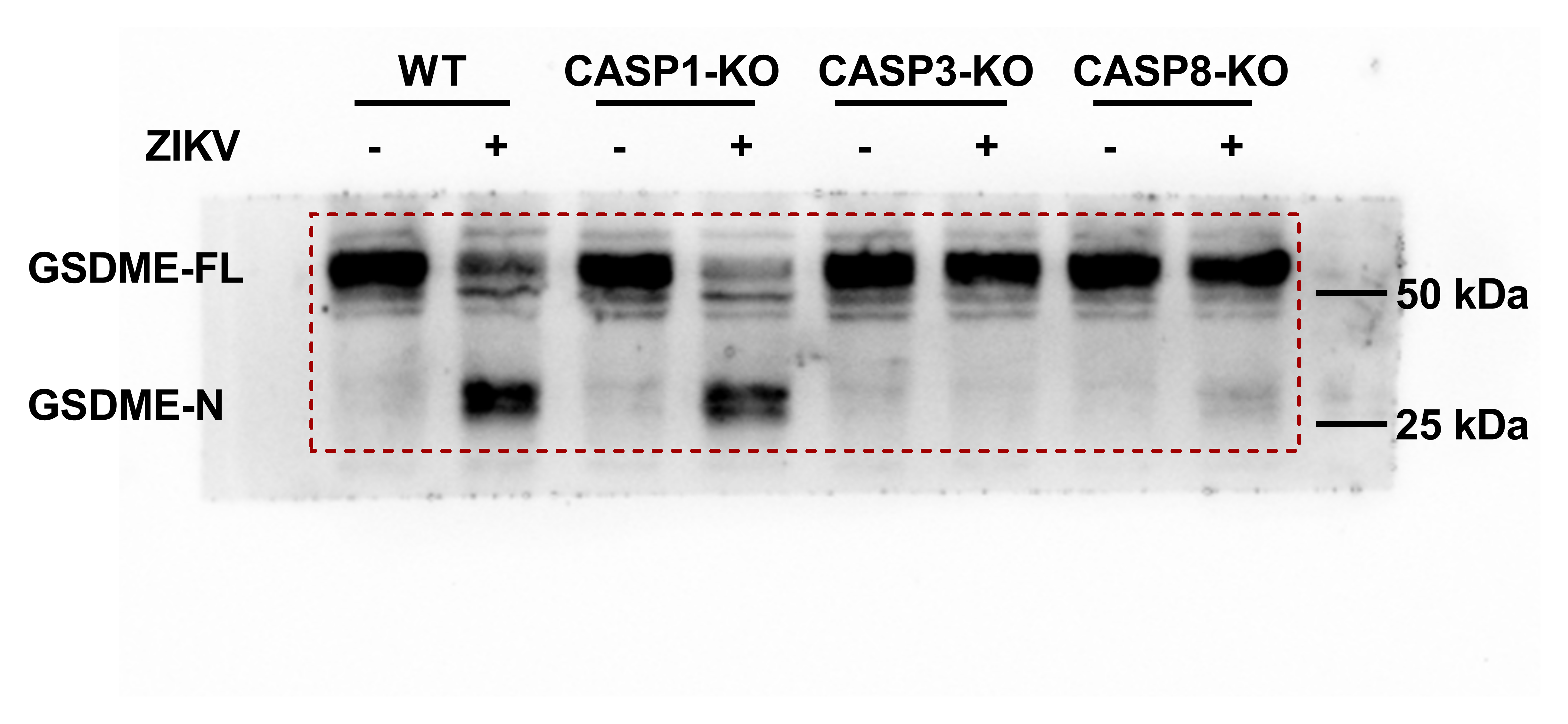

Supplement: Figure 3—source data 1. [file elife-73792-fig3-data1.zip › Figure 3-source data 1/Fig 3H/Figure 3H GSDME-labeled.tif]

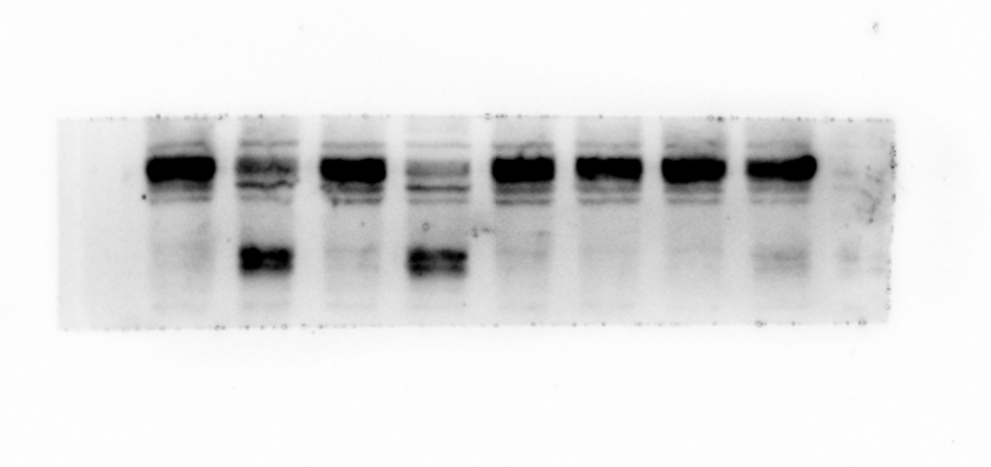

Supplement: Figure 3—source data 1. [file elife-73792-fig3-data1.zip › Figure 3-source data 1/Fig 3H/Figure 3H GSDME-raw.tif]

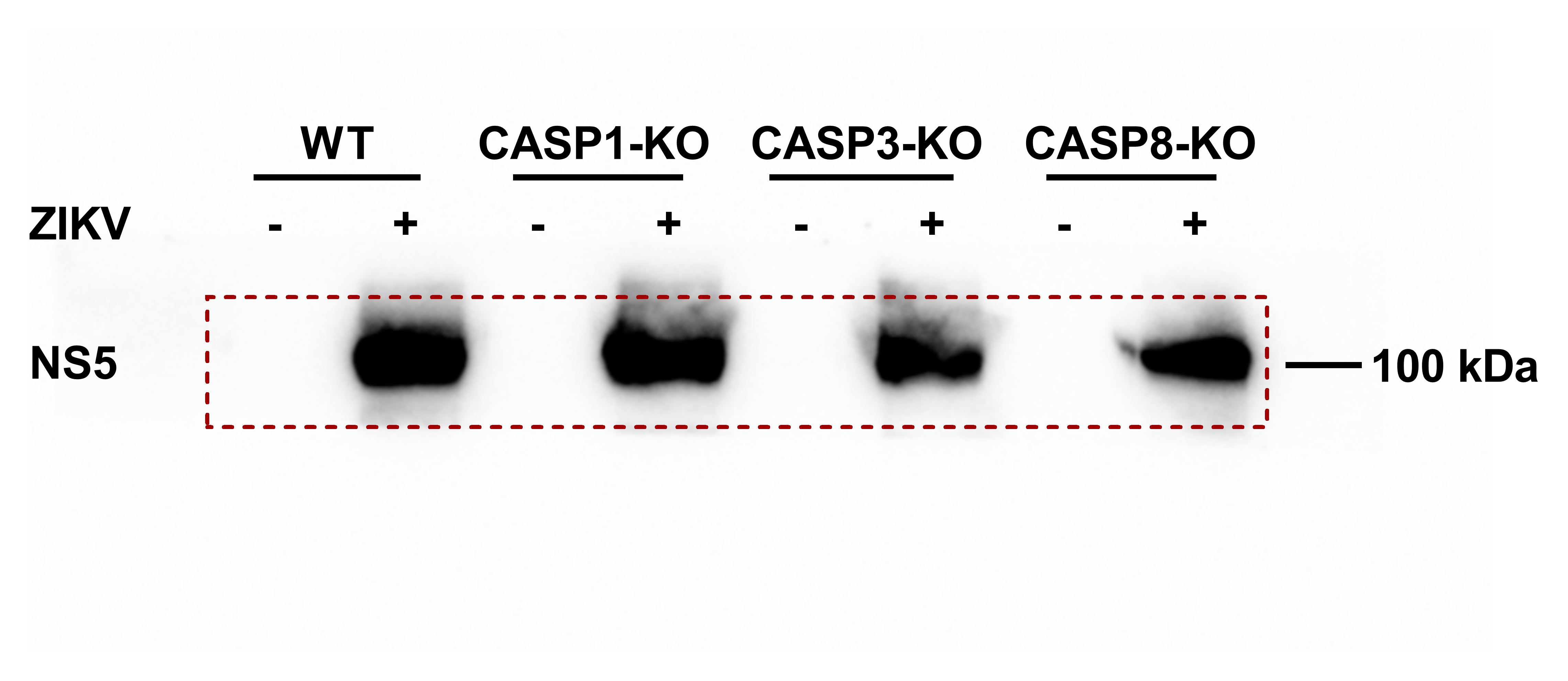

Supplement: Figure 3—source data 1. [file elife-73792-fig3-data1.zip › Figure 3-source data 1/Fig 3H/Figure 3H NS5-labeled.tif]

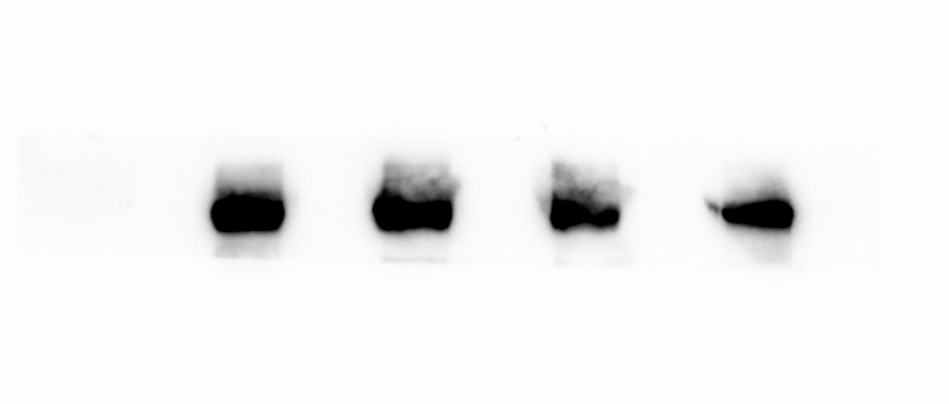

Supplement: Figure 3—source data 1. [file elife-73792-fig3-data1.zip › Figure 3-source data 1/Fig 3H/Figure 3H NS5-raw.Tif]

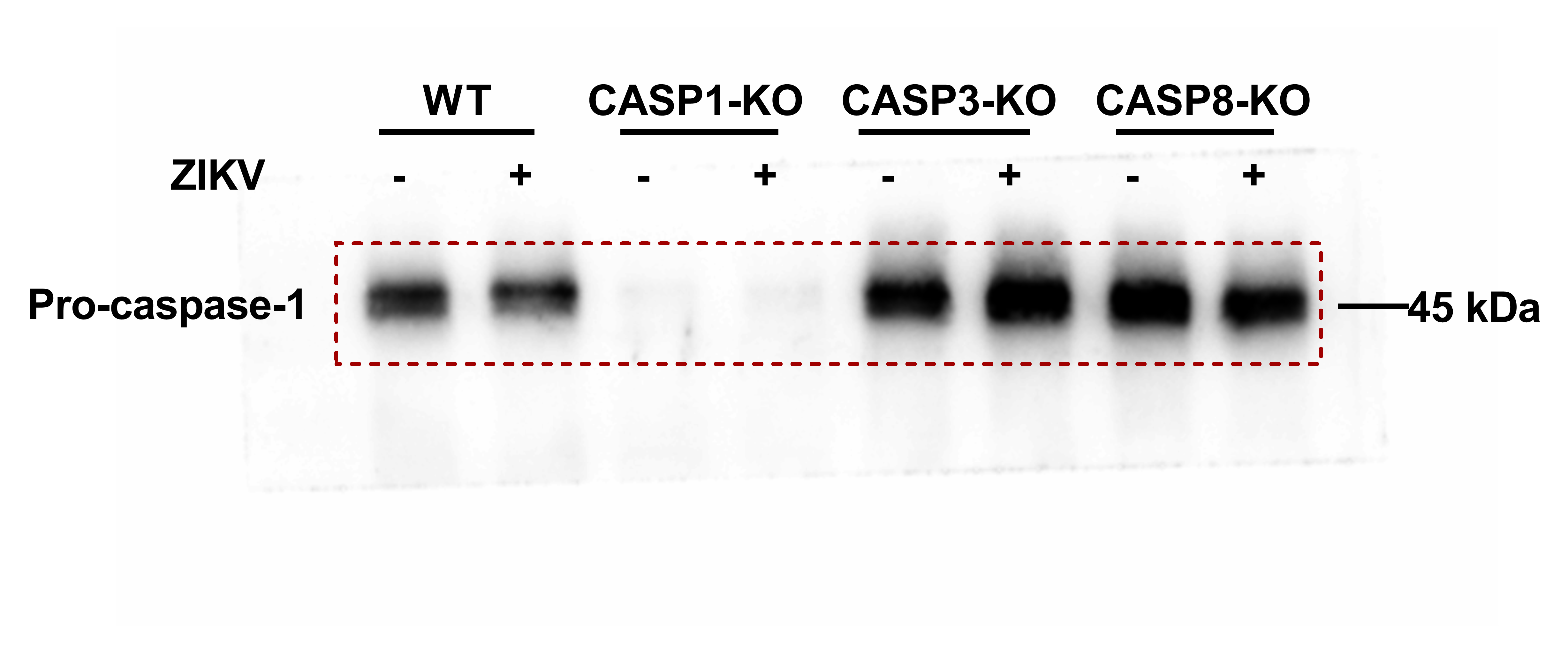

Supplement: Figure 3—source data 1. [file elife-73792-fig3-data1.zip › Figure 3-source data 1/Fig 3H/Figure 3H Pro-caspase-1-labeled.tif]

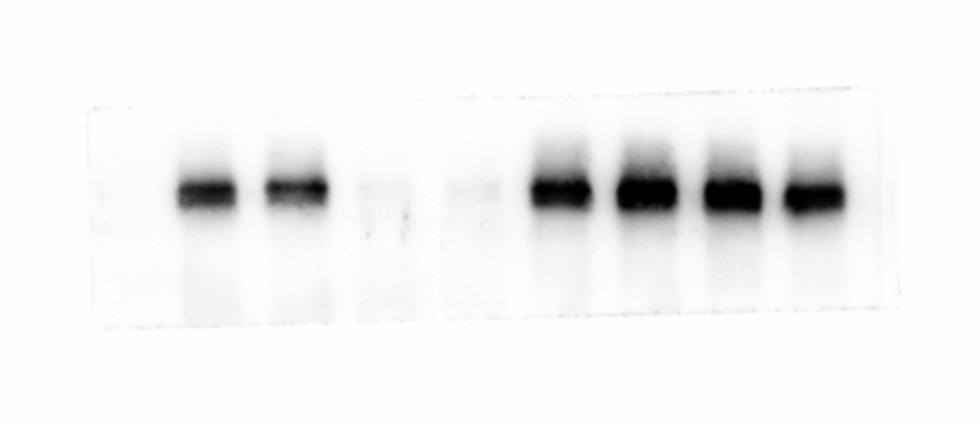

Supplement: Figure 3—source data 1. [file elife-73792-fig3-data1.zip › Figure 3-source data 1/Fig 3H/Figure 3H Pro-caspase-1-raw.tif]

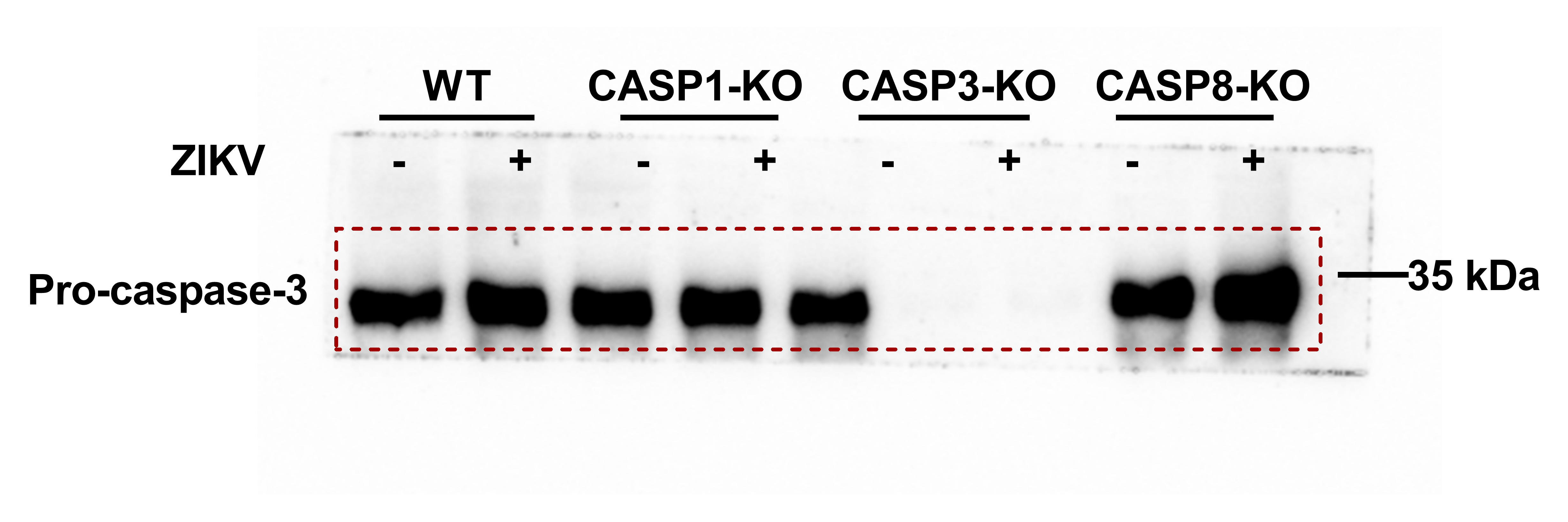

Supplement: Figure 3—source data 1. [file elife-73792-fig3-data1.zip › Figure 3-source data 1/Fig 3H/Figure 3H Pro-caspase-3-labeled.tif]

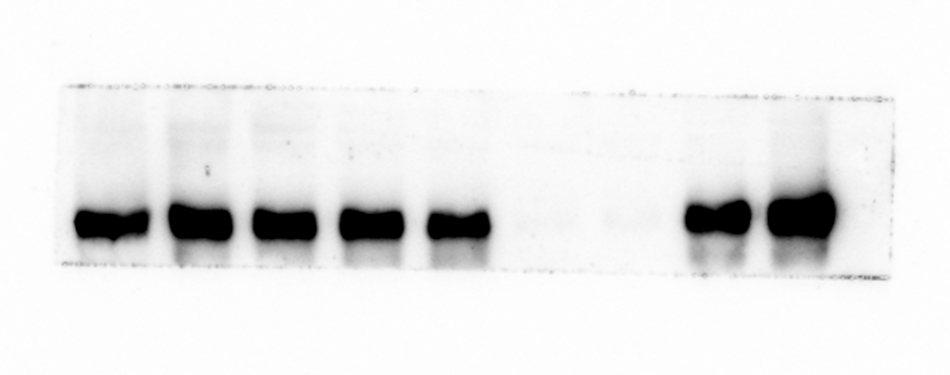

Supplement: Figure 3—source data 1. [file elife-73792-fig3-data1.zip › Figure 3-source data 1/Fig 3H/Figure 3H Pro-caspase-3-raw.tif]

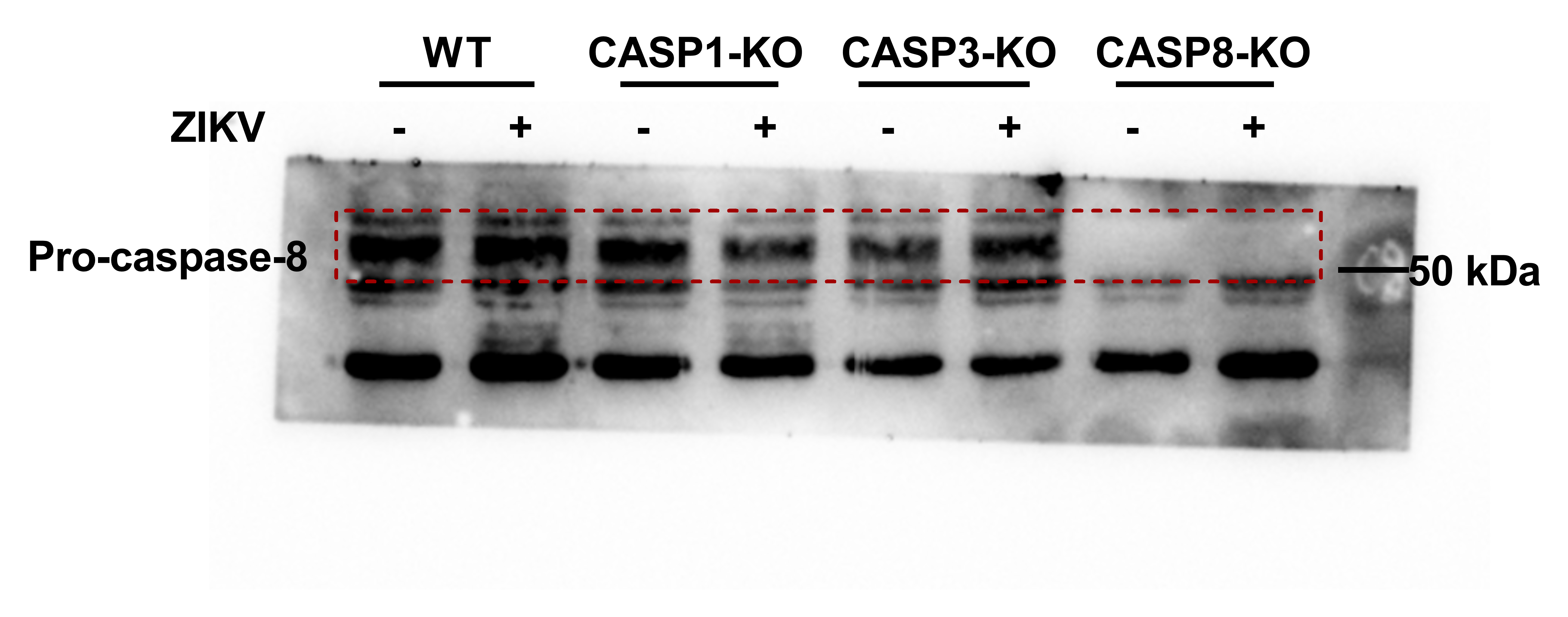

Supplement: Figure 3—source data 1. [file elife-73792-fig3-data1.zip › Figure 3-source data 1/Fig 3H/Figure 3H Pro-caspase-8-labeled.tif]

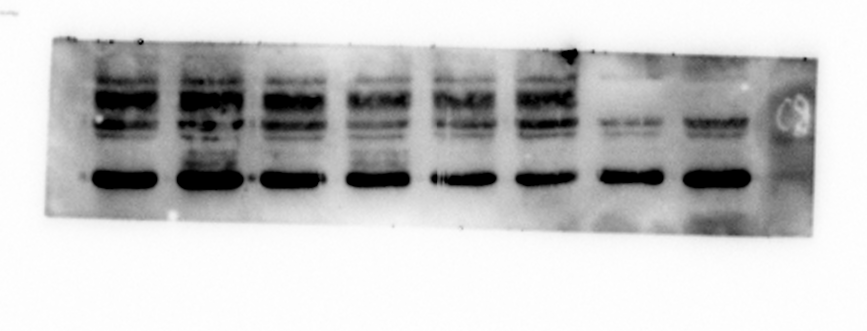

Supplement: Figure 3—source data 1. [file elife-73792-fig3-data1.zip › Figure 3-source data 1/Fig 3H/Figure 3H Pro-caspase-8-raw.Tif]

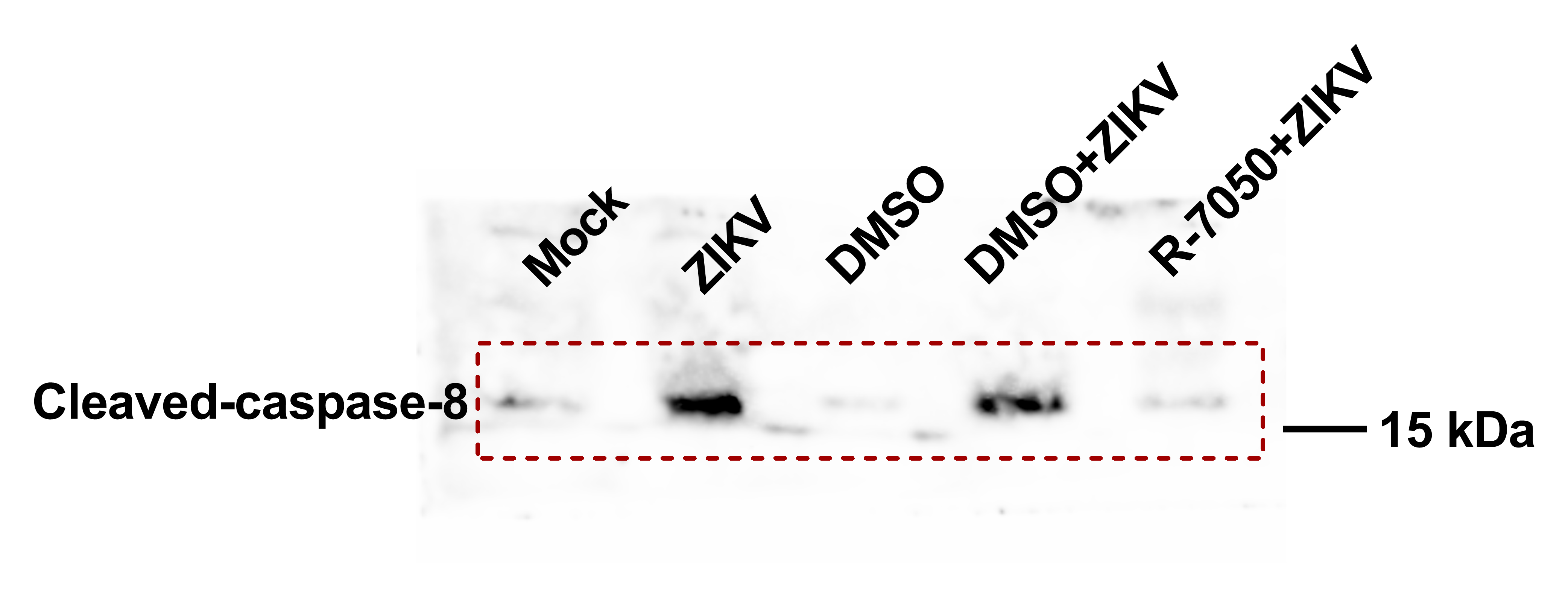

Supplement: Figure 3—source data 1. [file elife-73792-fig3-data1.zip › Figure 3-source data 1/Fig 3I/Figure 3L Cleaved-caspase-8-labeled.tif]

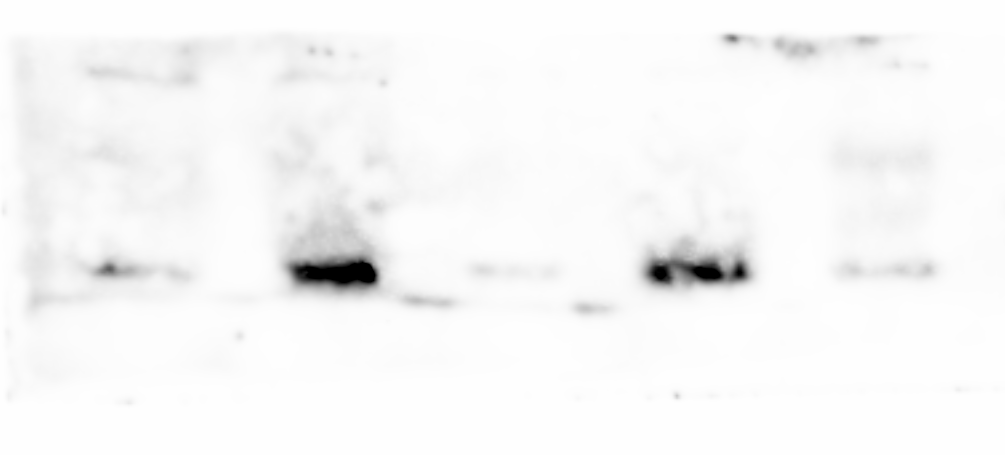

Supplement: Figure 3—source data 1. [file elife-73792-fig3-data1.zip › Figure 3-source data 1/Fig 3I/Figure 3L Cleaved-caspase-8-raw.tif]

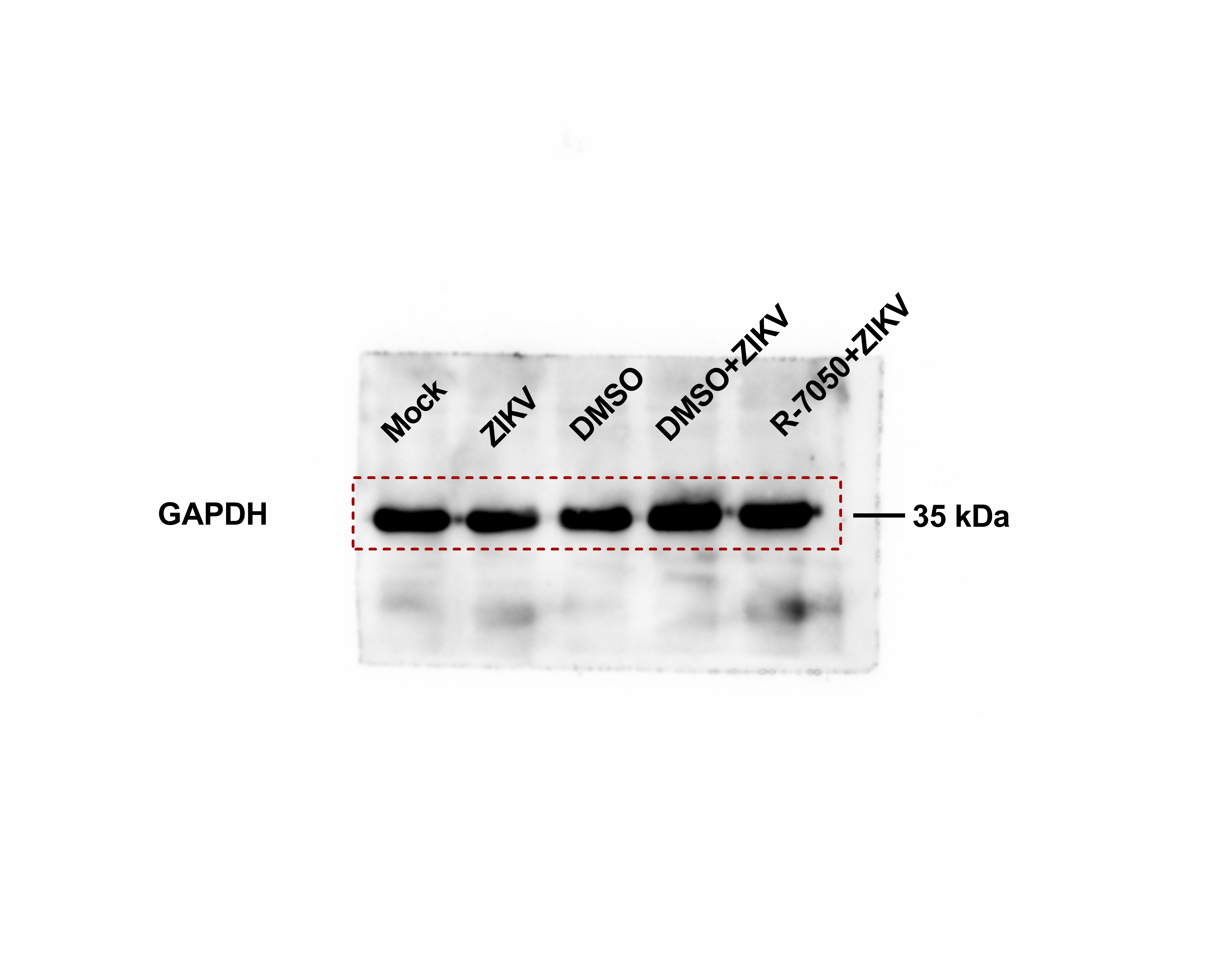

Supplement: Figure 3—source data 1. [file elife-73792-fig3-data1.zip › Figure 3-source data 1/Fig 3I/Figure 3L GAPDH-labeled.tif]

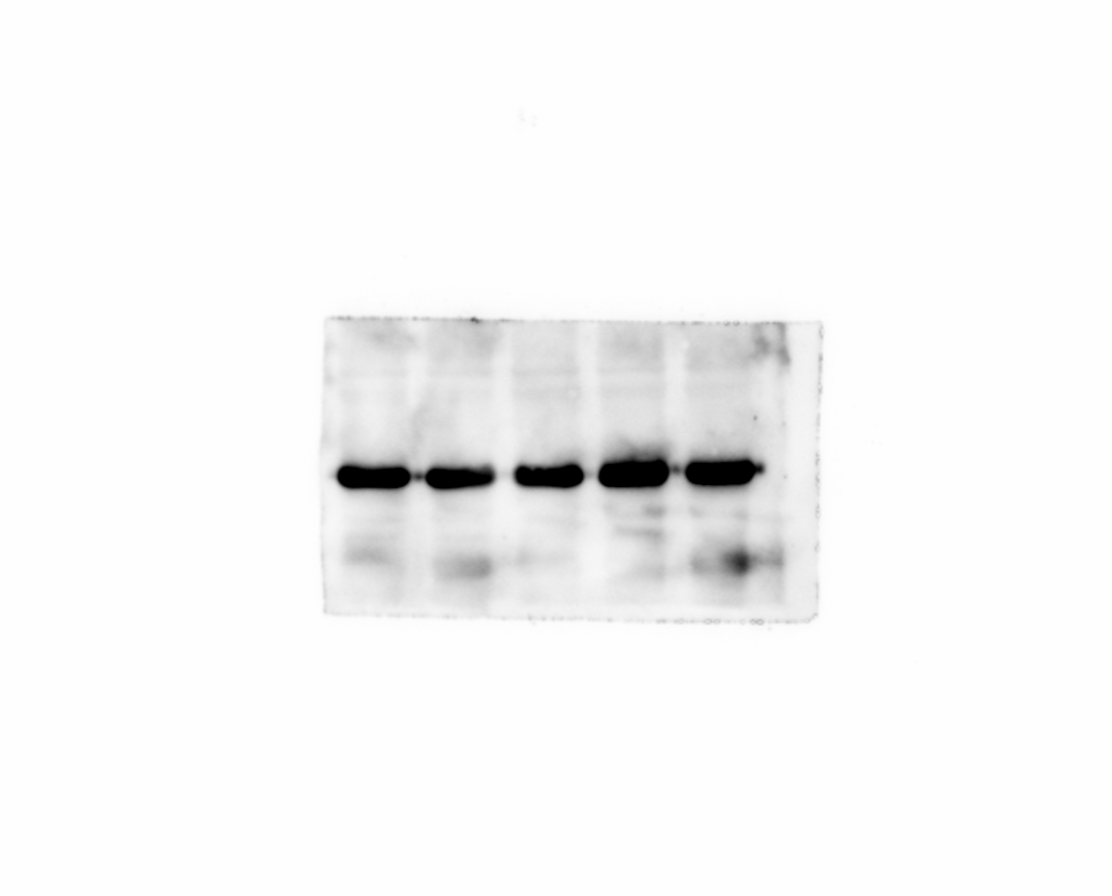

Supplement: Figure 3—source data 1. [file elife-73792-fig3-data1.zip › Figure 3-source data 1/Fig 3I/Figure 3L GAPDH-raw.tif]

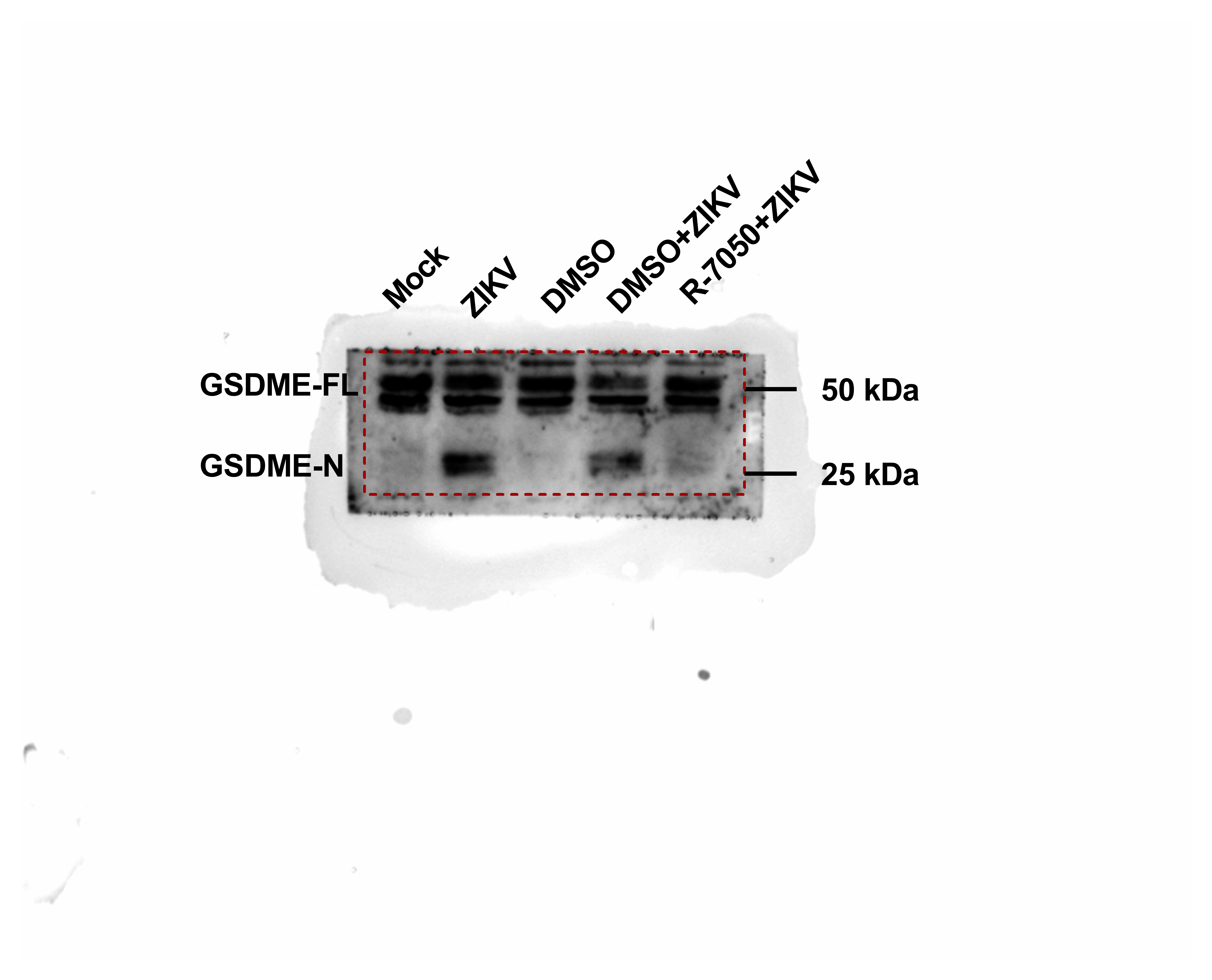

Supplement: Figure 3—source data 1. [file elife-73792-fig3-data1.zip › Figure 3-source data 1/Fig 3I/Figure 3L GSDME-labeled.tif]

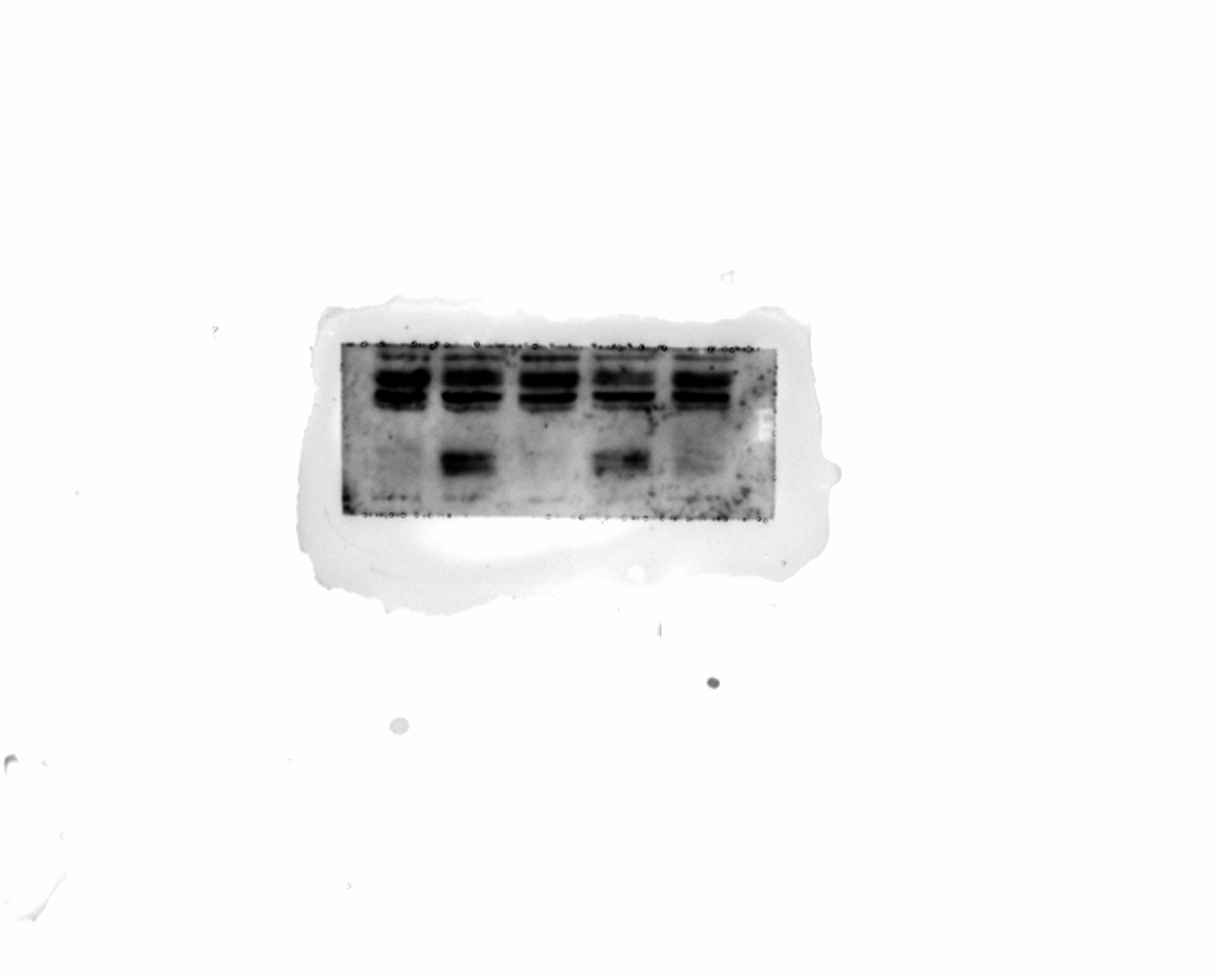

Supplement: Figure 3—source data 1. [file elife-73792-fig3-data1.zip › Figure 3-source data 1/Fig 3I/Figure 3L GSDME-raw.tif]

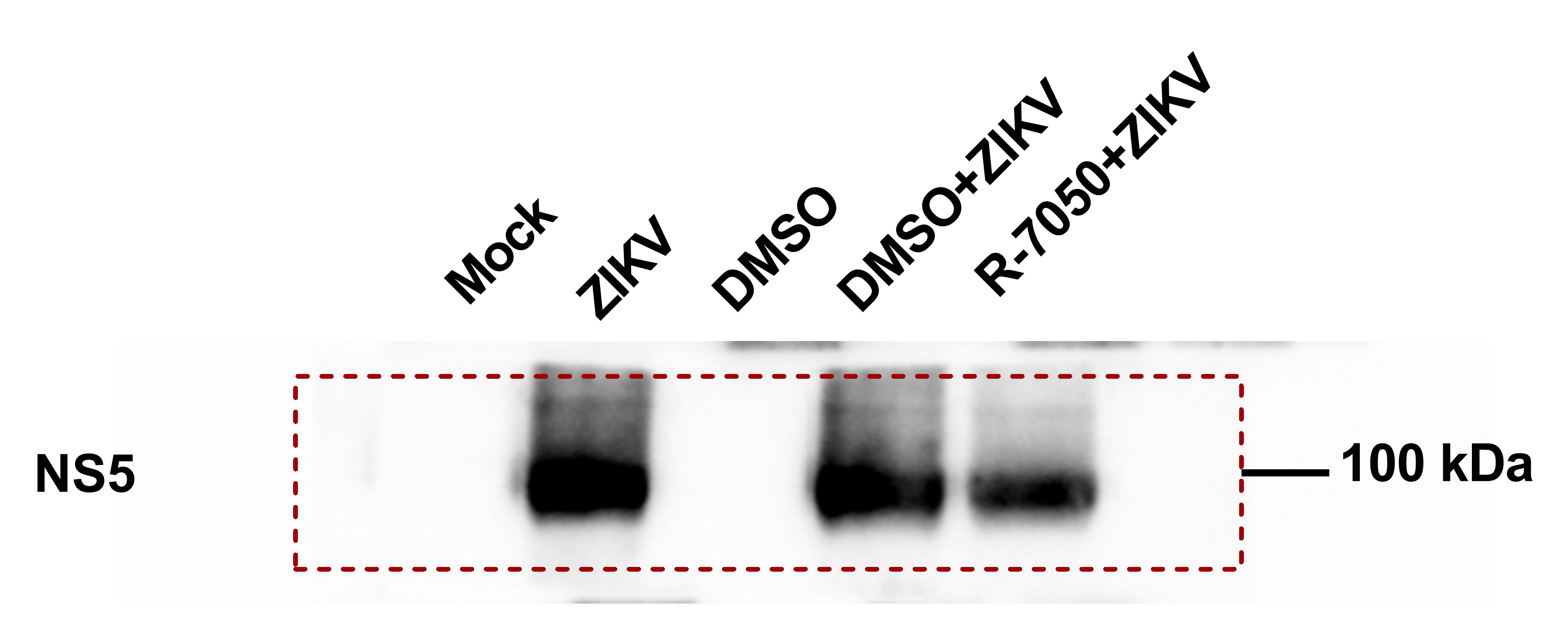

Supplement: Figure 3—source data 1. [file elife-73792-fig3-data1.zip › Figure 3-source data 1/Fig 3I/Figure 3L NS5-labeled.tif]

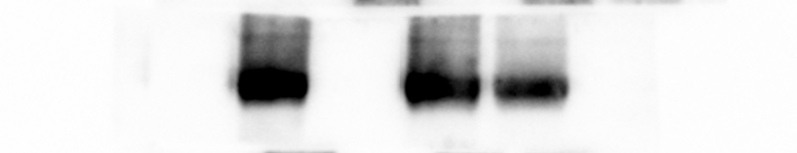

Supplement: Figure 3—source data 1. [file elife-73792-fig3-data1.zip › Figure 3-source data 1/Fig 3I/Figure 3L NS5-raw.tif]

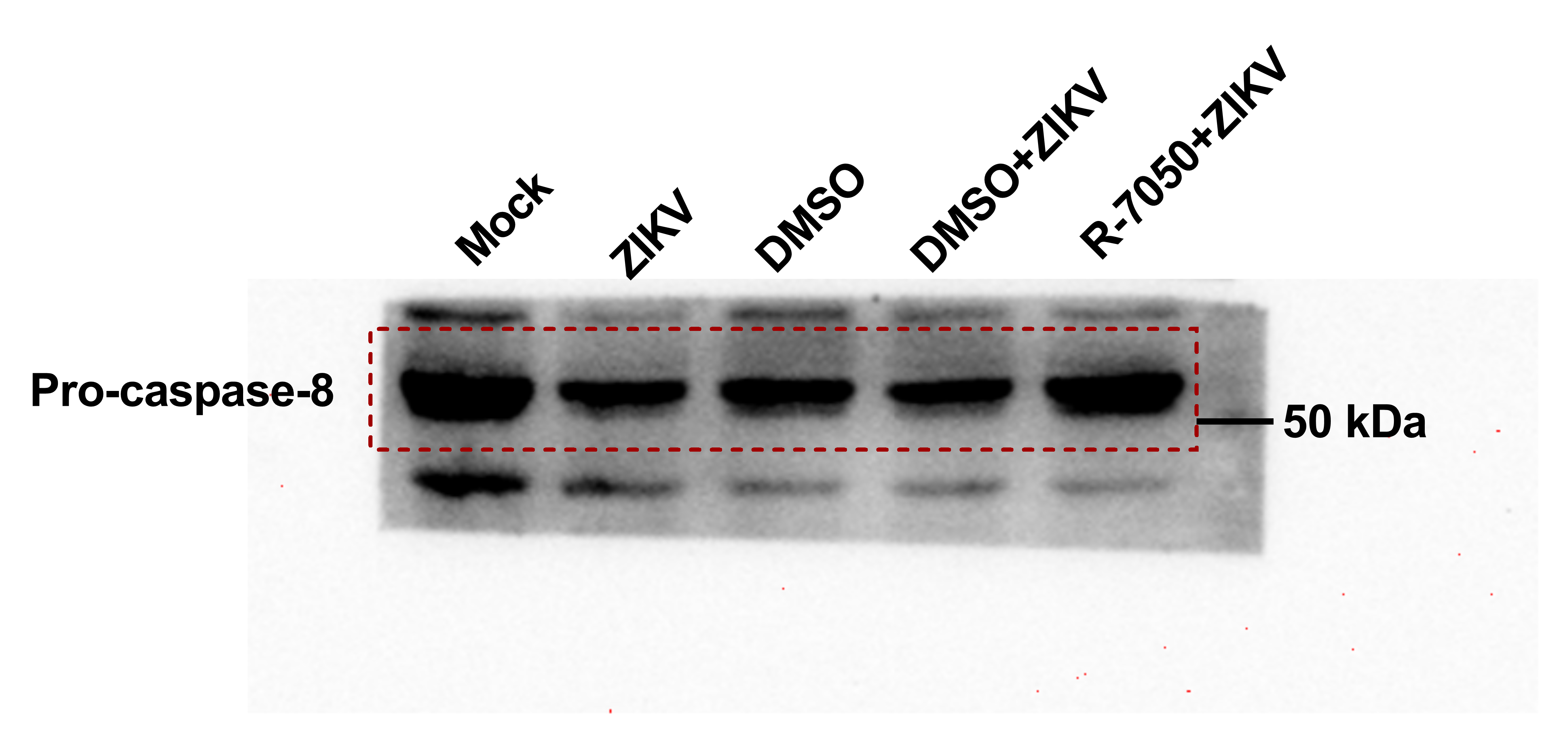

Supplement: Figure 3—source data 1. [file elife-73792-fig3-data1.zip › Figure 3-source data 1/Fig 3I/Figure 3L Pro-caspase-8-labeled.tif]

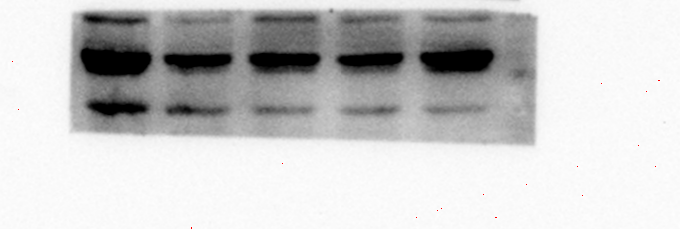

Supplement: Figure 3—source data 1. [file elife-73792-fig3-data1.zip › Figure 3-source data 1/Fig 3I/Figure 3L Pro-caspase-8-raw.tif]

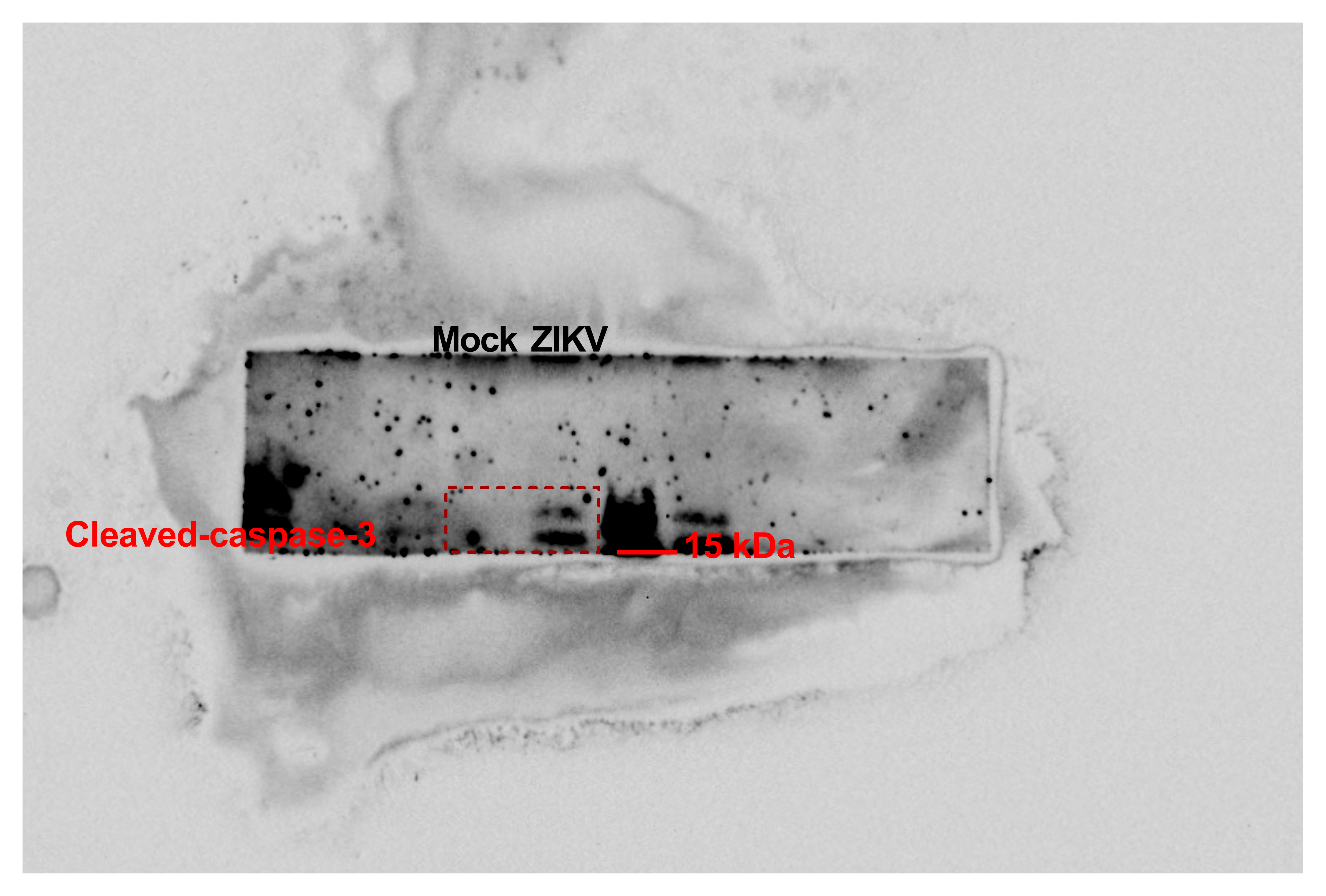

Supplement: Figure 3—figure supplement 1—source data 1. [file elife-73792-fig3-figsupp1-data1.zip › Figure 3-figure supplement 1-source data/1a/Figure 3-figure supplement 1 Cleaved caspase-3-labeled.tif]

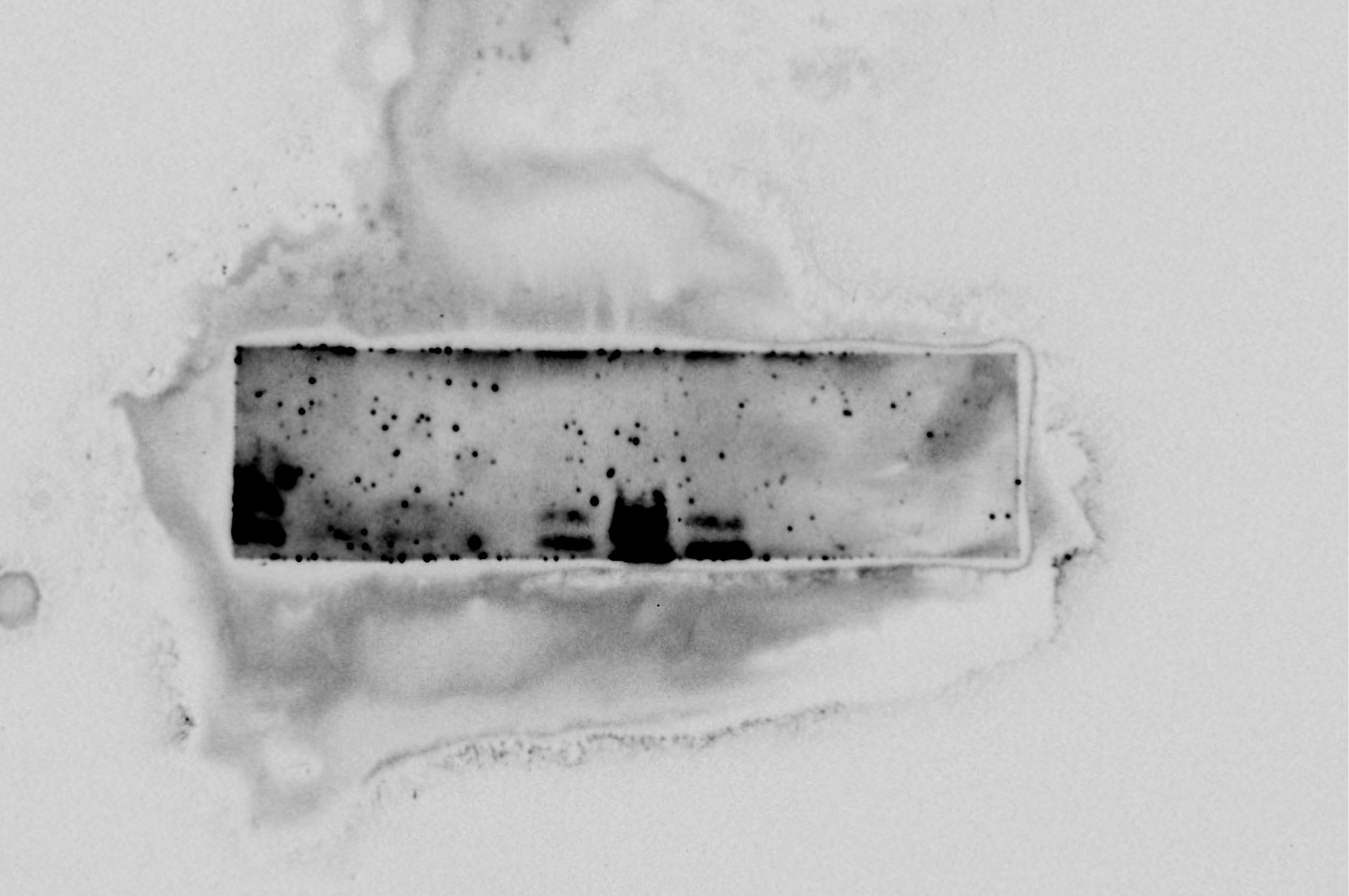

Supplement: Figure 3—figure supplement 1—source data 1. [file elife-73792-fig3-figsupp1-data1.zip › Figure 3-figure supplement 1-source data/1a/Figure 3-figure supplement 1 Cleaved caspase-3-raw.tif]

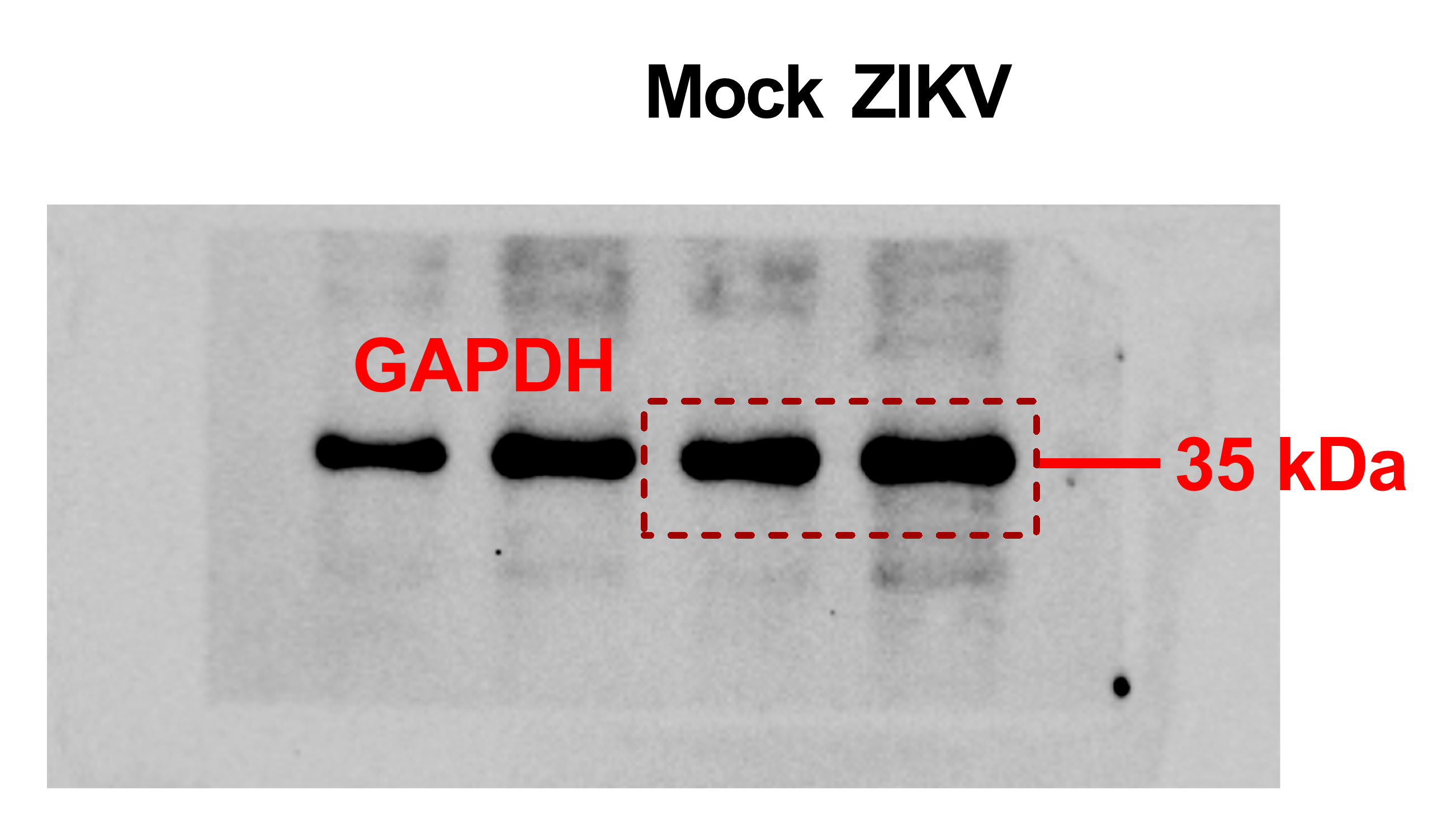

Supplement: Figure 3—figure supplement 1—source data 1. [file elife-73792-fig3-figsupp1-data1.zip › Figure 3-figure supplement 1-source data/1a/Figure 3-figure supplement 1 GAPDH-labeled.tif]

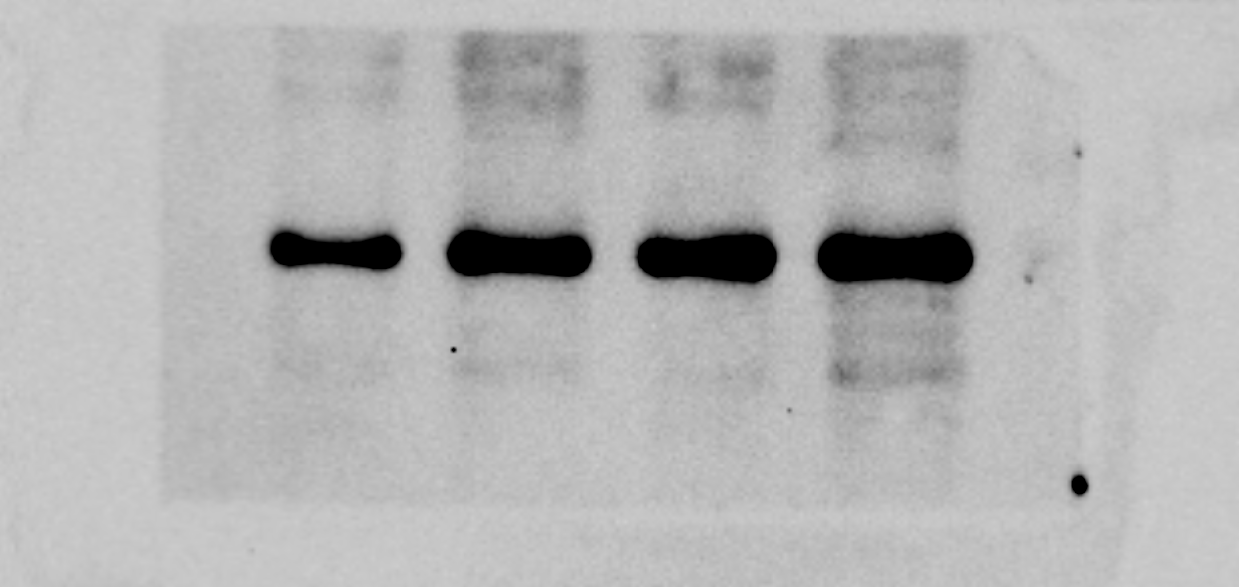

Supplement: Figure 3—figure supplement 1—source data 1. [file elife-73792-fig3-figsupp1-data1.zip › Figure 3-figure supplement 1-source data/1a/Figure 3-figure supplement 1 GAPDH-raw.tif.tif]

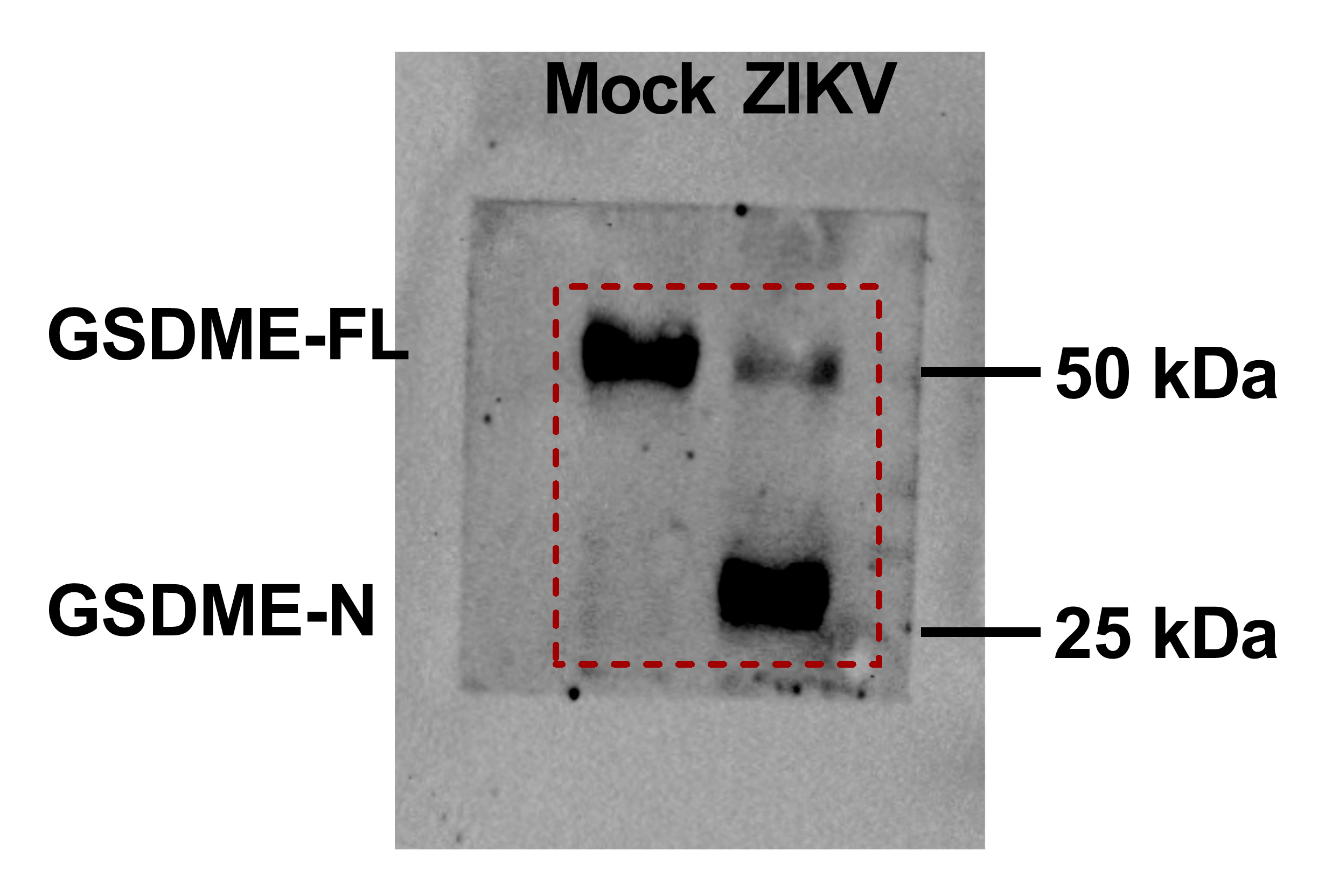

Supplement: Figure 3—figure supplement 1—source data 1. [file elife-73792-fig3-figsupp1-data1.zip › Figure 3-figure supplement 1-source data/1a/Figure 3-figure supplement 1 GSDME-labeled.tif]

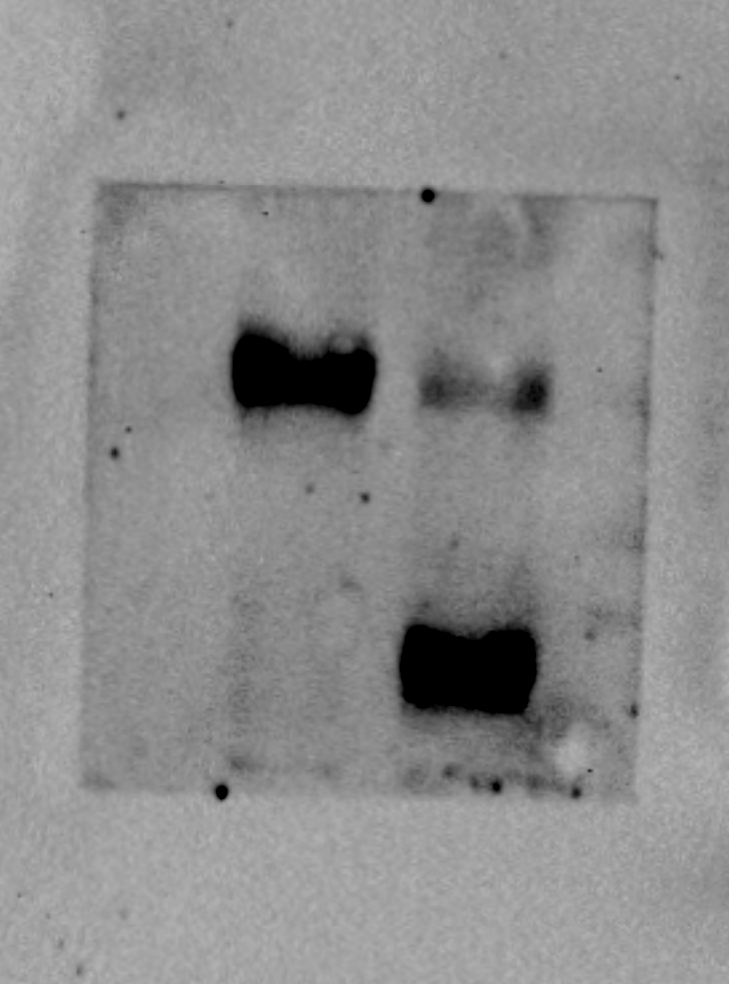

Supplement: Figure 3—figure supplement 1—source data 1. [file elife-73792-fig3-figsupp1-data1.zip › Figure 3-figure supplement 1-source data/1a/Figure 3-figure supplement 1 GSDME-raw.tif.tif]

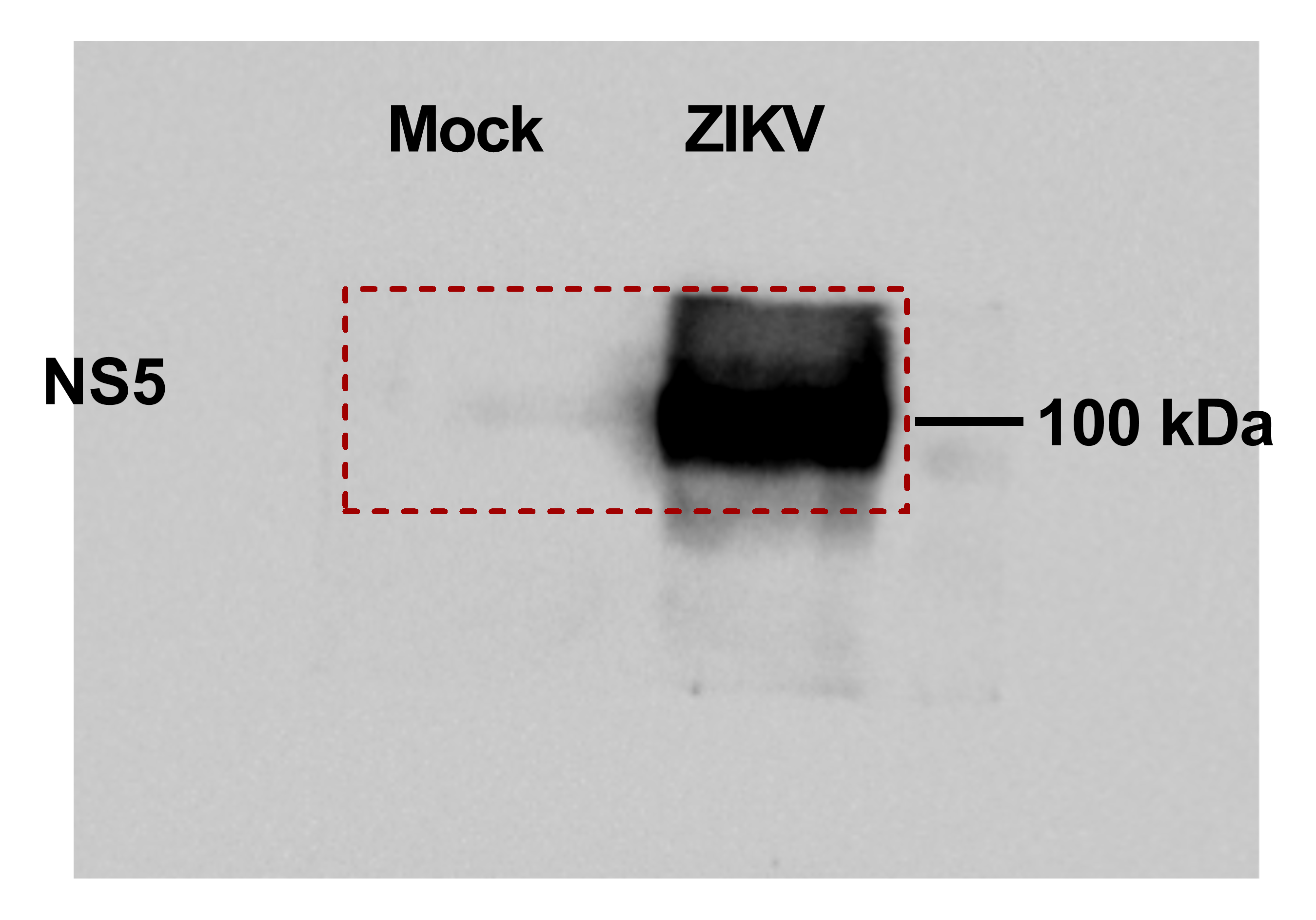

Supplement: Figure 3—figure supplement 1—source data 1. [file elife-73792-fig3-figsupp1-data1.zip › Figure 3-figure supplement 1-source data/1a/Figure 3-figure supplement 1 NS5-labeled.tif]

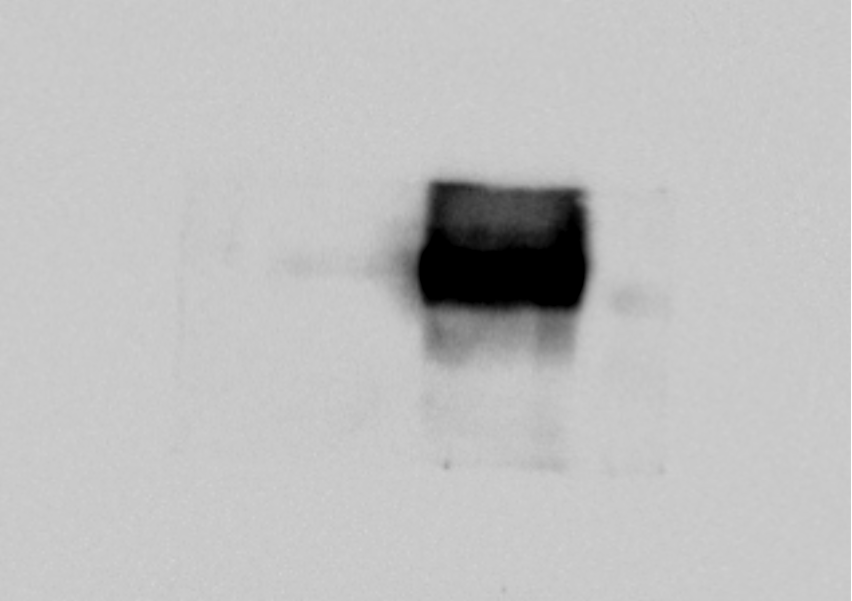

Supplement: Figure 3—figure supplement 1—source data 1. [file elife-73792-fig3-figsupp1-data1.zip › Figure 3-figure supplement 1-source data/1a/Figure 3-figure supplement 1 NS5-raw.tif]

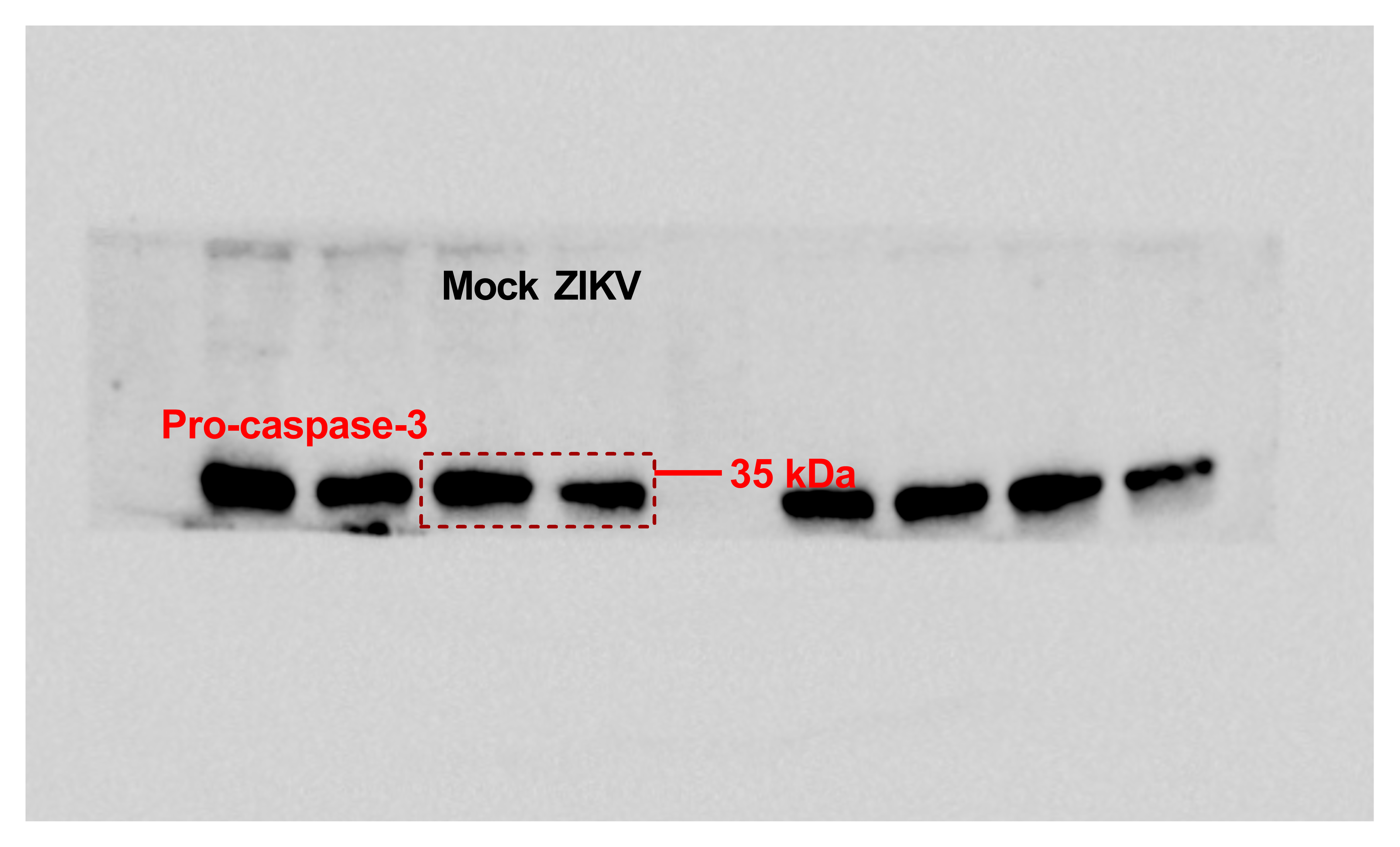

Supplement: Figure 3—figure supplement 1—source data 1. [file elife-73792-fig3-figsupp1-data1.zip › Figure 3-figure supplement 1-source data/1a/Figure 3-figure supplement 1 Pro-caspase-3-labeled.tif]
