# Supplementary figures and images for: Zika virus causes placental pyroptosis and associated adverse fetal outcomes by activating GSDME (part 3 of 4)
Source: eLife. 2022 Aug 16;11:e73792. doi: 10.7554/eLife.73792 (PMC9381041; doi:10.7554/eLife.73792)

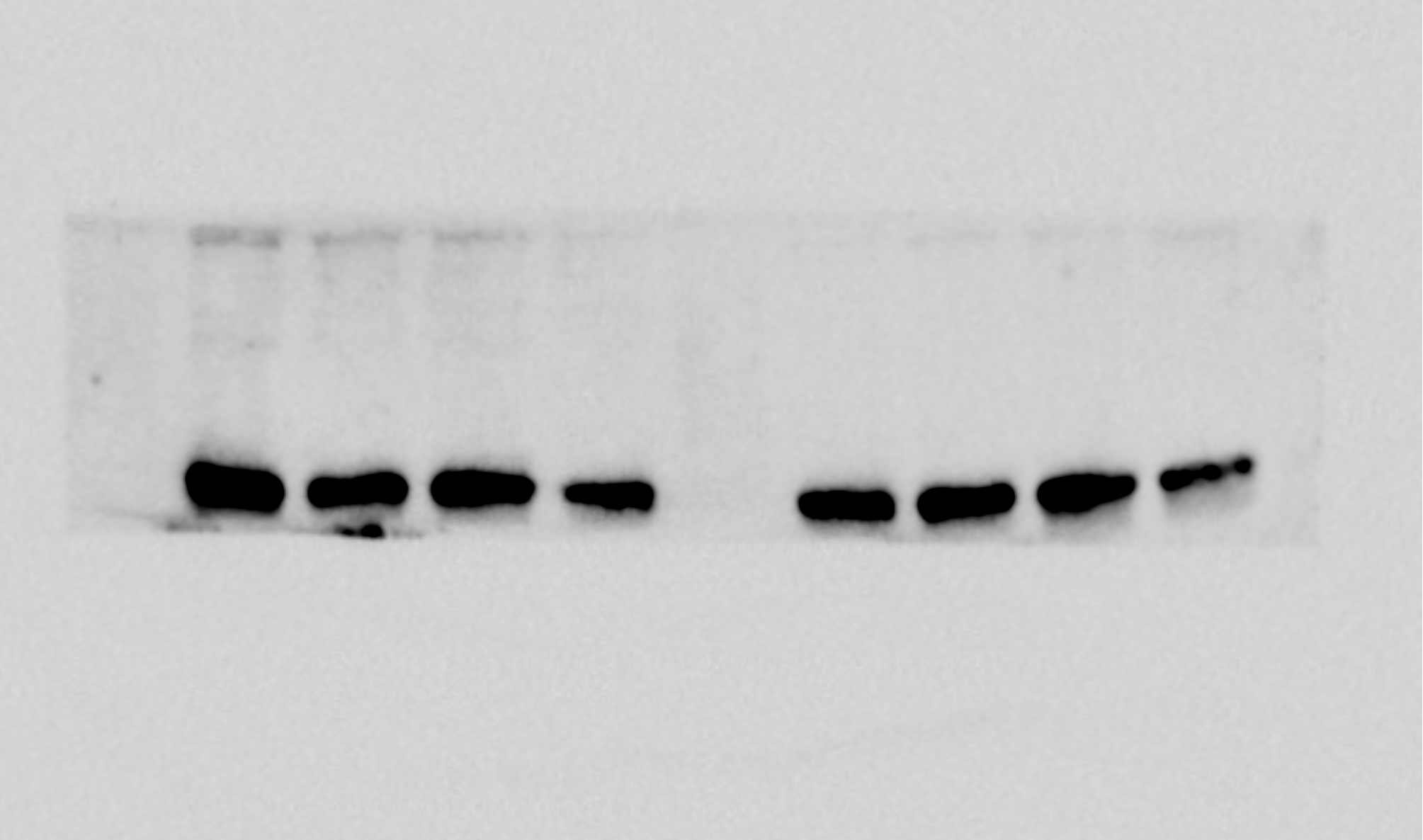

Supplement: Figure 3—figure supplement 1—source data 1. [file elife-73792-fig3-figsupp1-data1.zip › Figure 3-figure supplement 1-source data/1a/Figure 3-figure supplement 1 Pro-caspase-3-raw.tif]

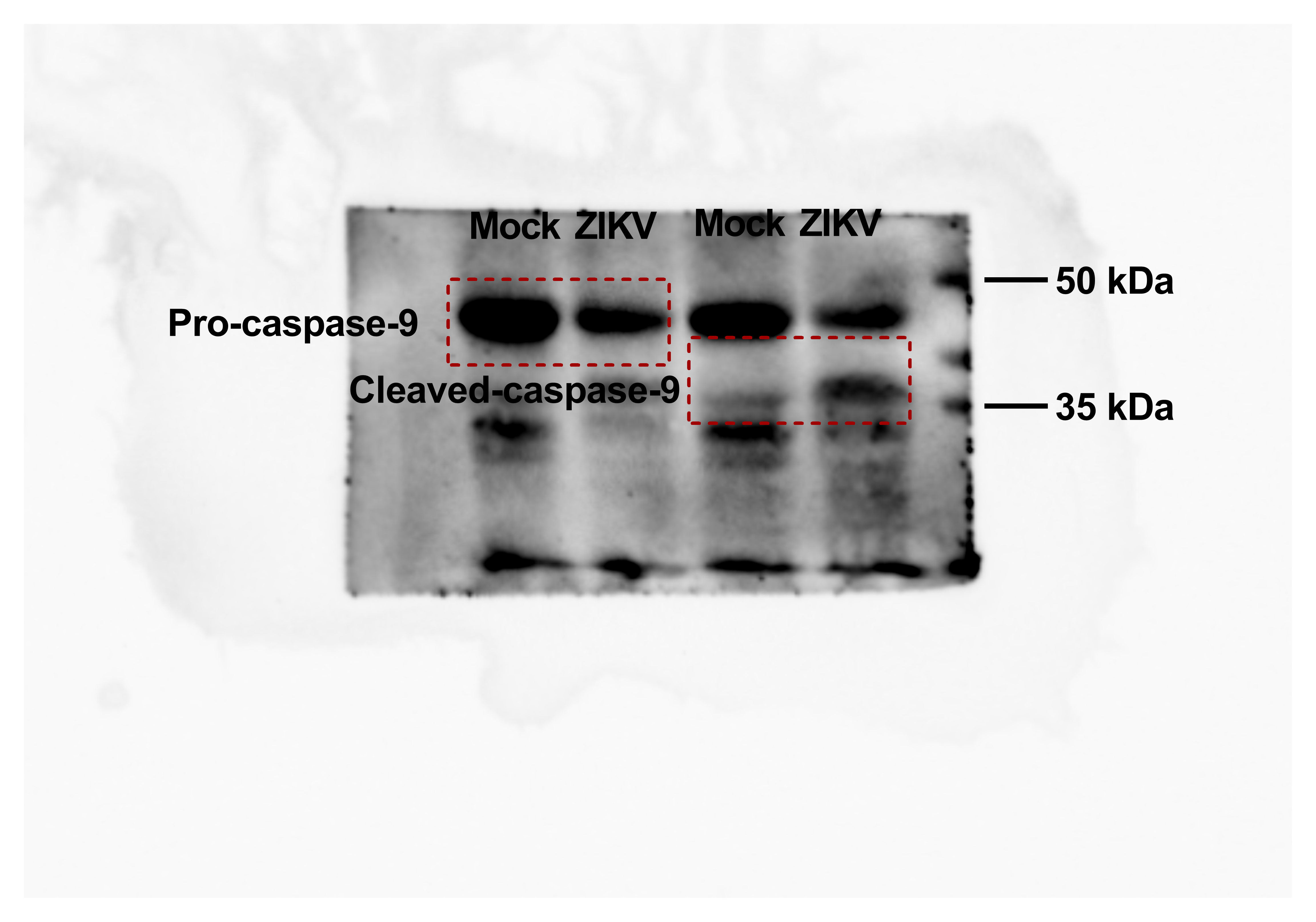

Supplement: Figure 3—figure supplement 1—source data 1. [file elife-73792-fig3-figsupp1-data1.zip › Figure 3-figure supplement 1-source data/1c/Figure 3-figure supplement 2 caspase-9-labeled.tif]

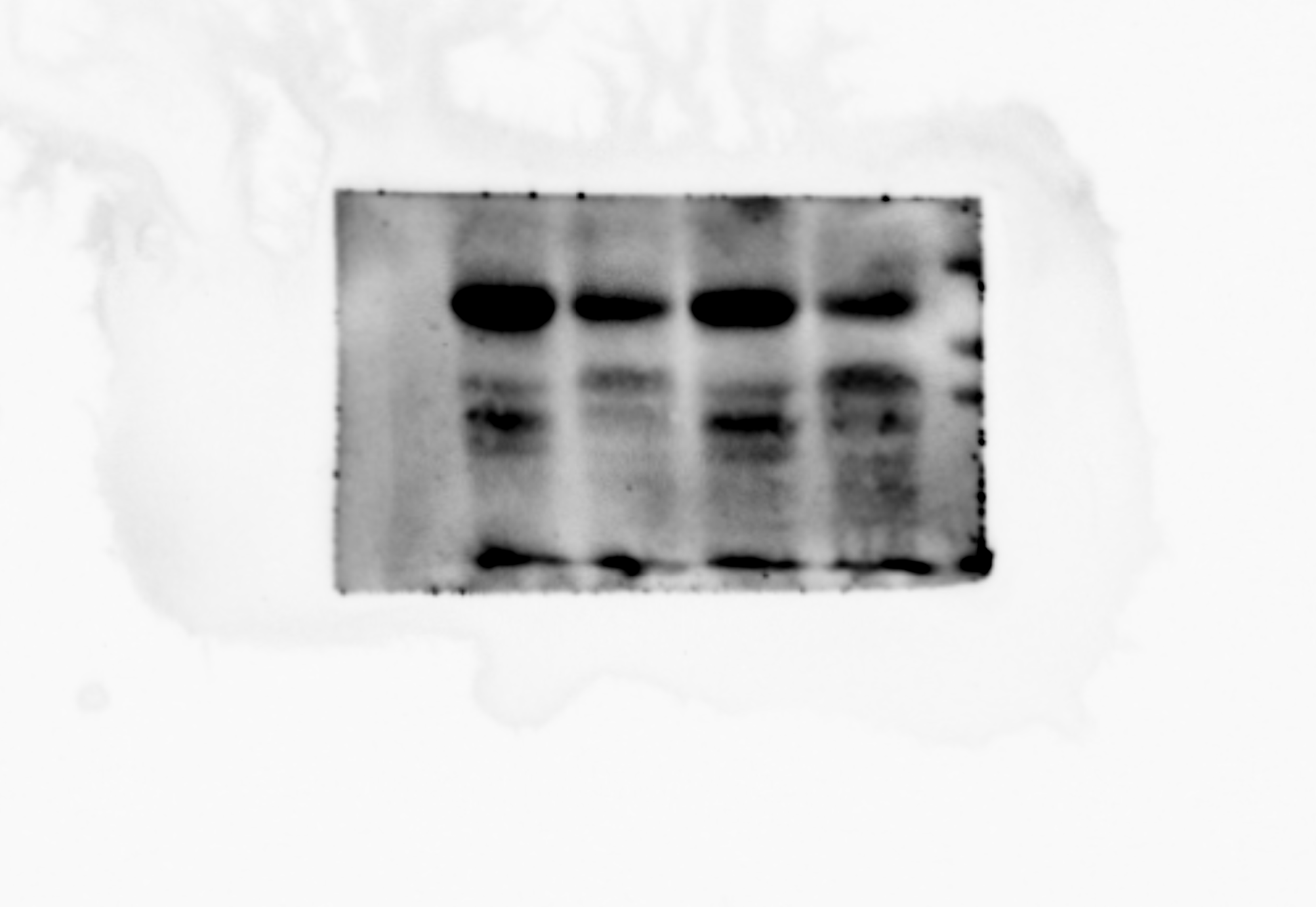

Supplement: Figure 3—figure supplement 1—source data 1. [file elife-73792-fig3-figsupp1-data1.zip › Figure 3-figure supplement 1-source data/1c/Figure 3-figure supplement 2 caspase-9-raw.tif]

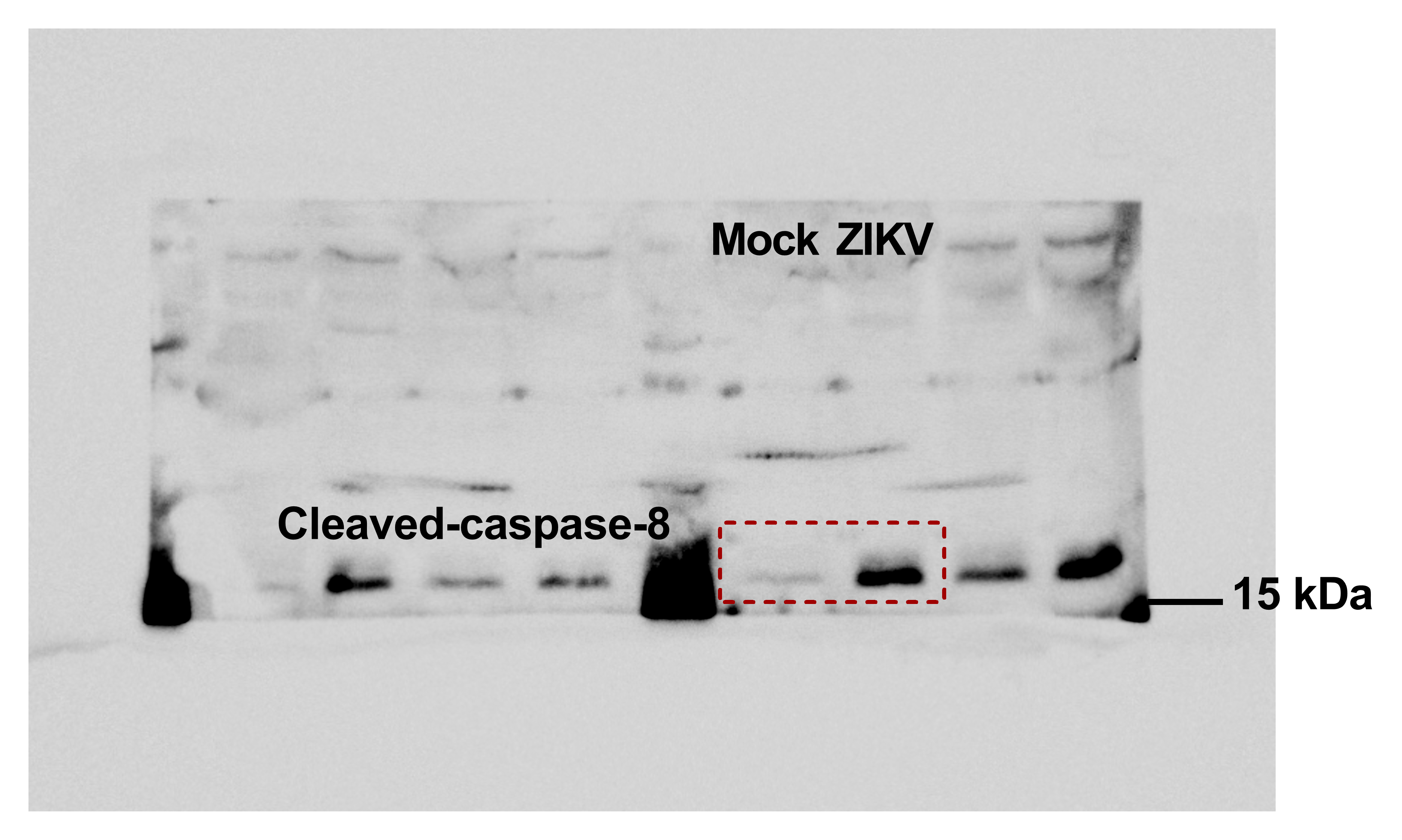

Supplement: Figure 3—figure supplement 1—source data 1. [file elife-73792-fig3-figsupp1-data1.zip › Figure 3-figure supplement 1-source data/1c/Figure 3-figure supplement 2 Cleaved caspase-8-labeled.tif]

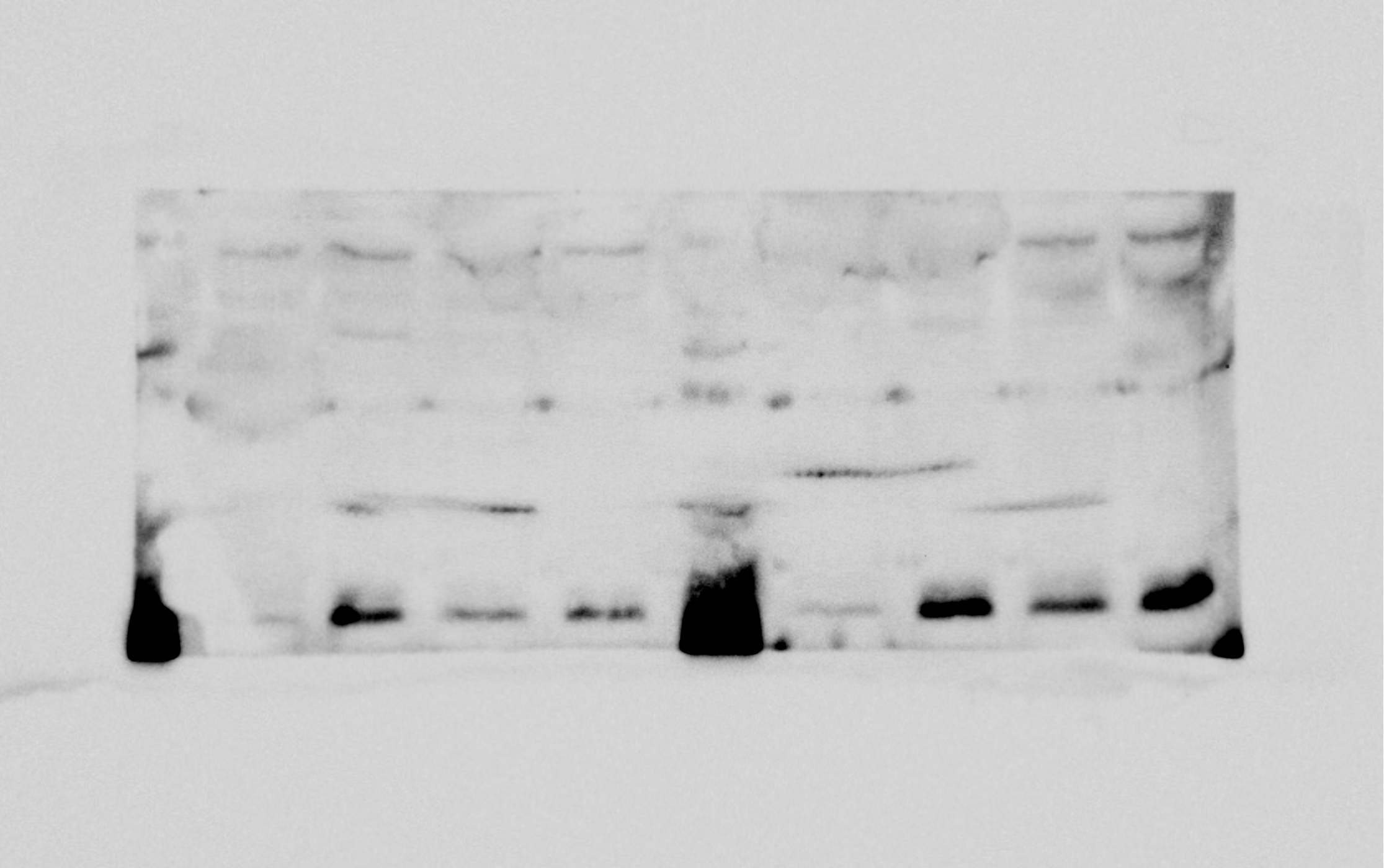

Supplement: Figure 3—figure supplement 1—source data 1. [file elife-73792-fig3-figsupp1-data1.zip › Figure 3-figure supplement 1-source data/1c/Figure 3-figure supplement 2 Cleaved caspase-8-raw.tif]

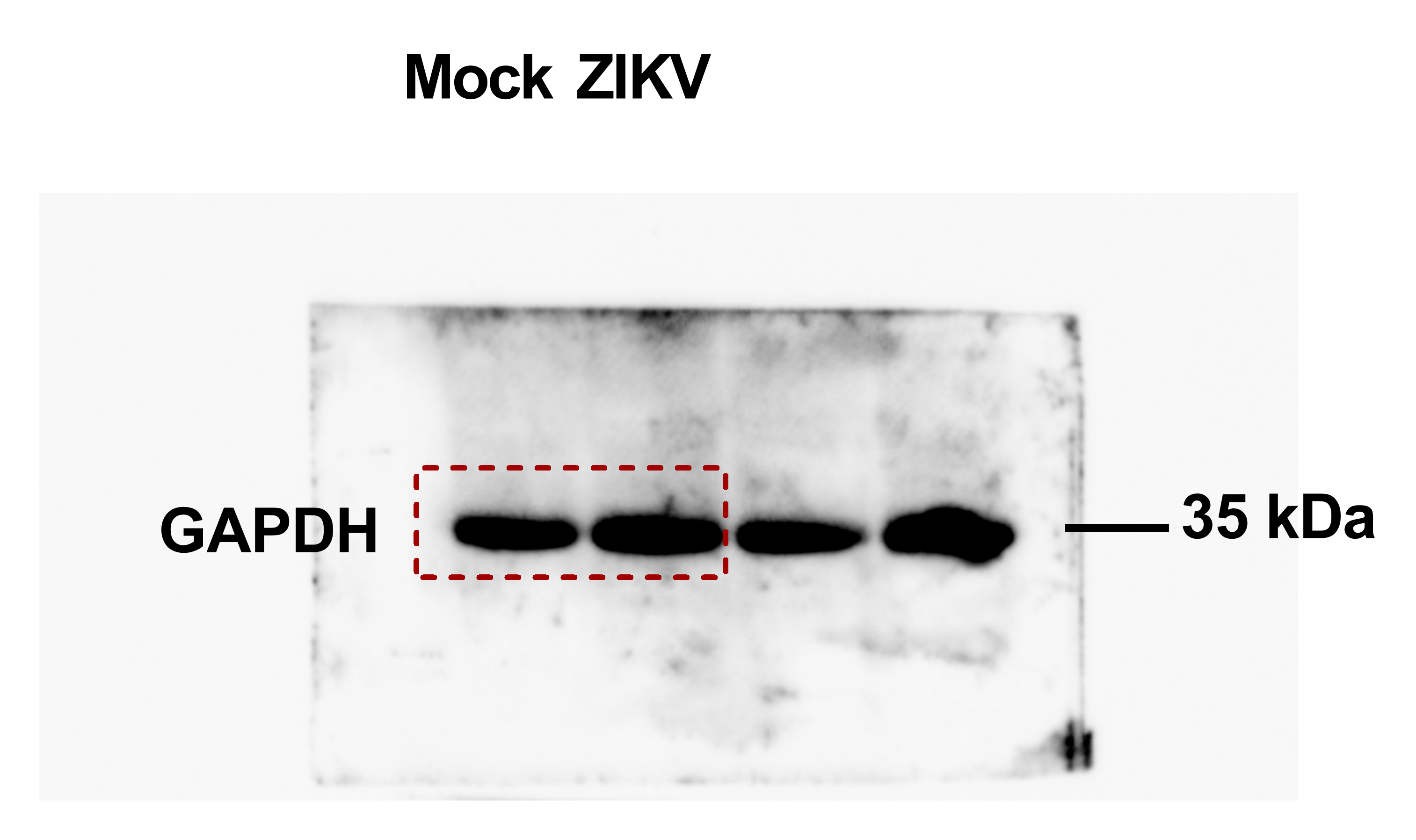

Supplement: Figure 3—figure supplement 1—source data 1. [file elife-73792-fig3-figsupp1-data1.zip › Figure 3-figure supplement 1-source data/1c/Figure 3-figure supplement 2 GAPDH-labeled.tif]

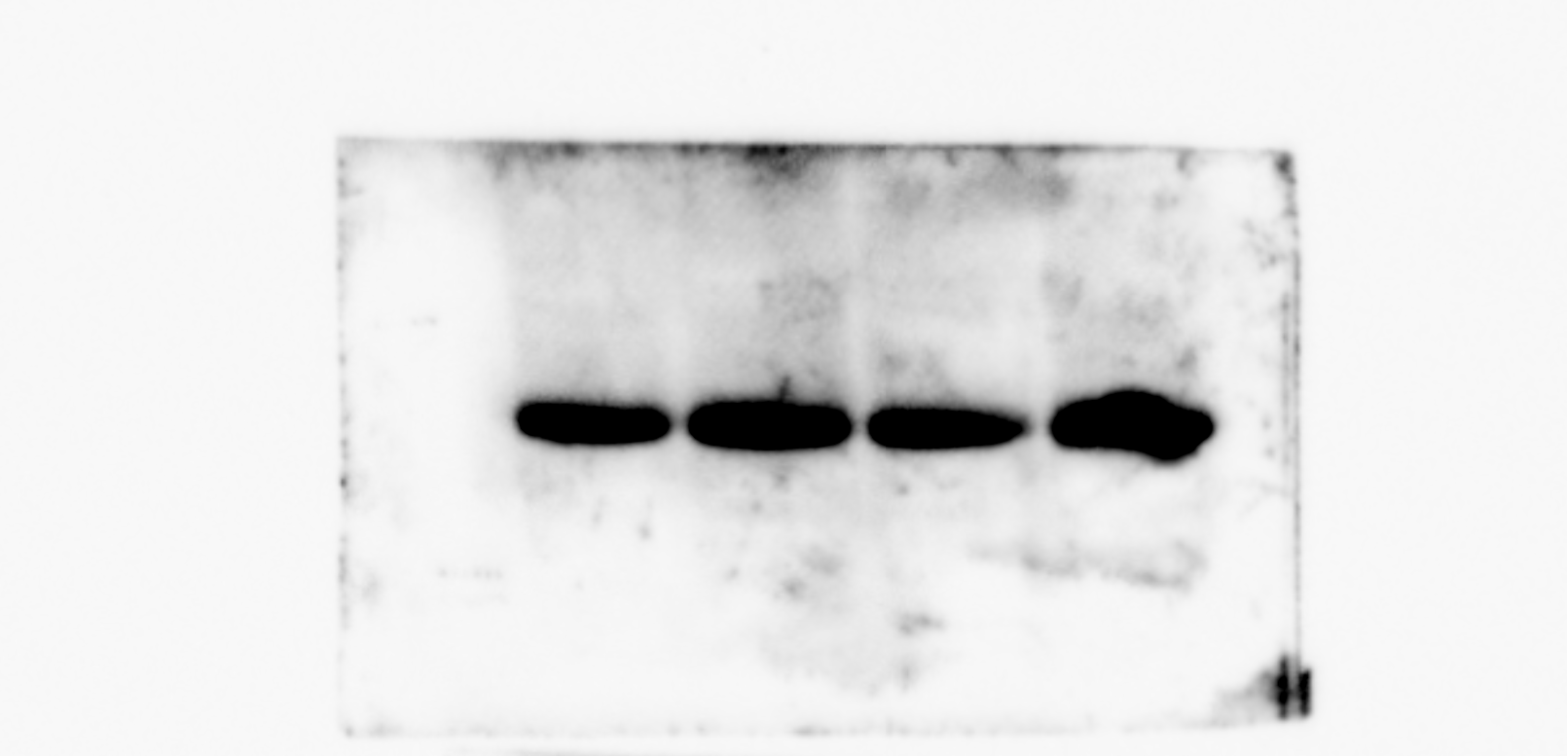

Supplement: Figure 3—figure supplement 1—source data 1. [file elife-73792-fig3-figsupp1-data1.zip › Figure 3-figure supplement 1-source data/1c/Figure 3-figure supplement 2 GAPDH-raw.tif]

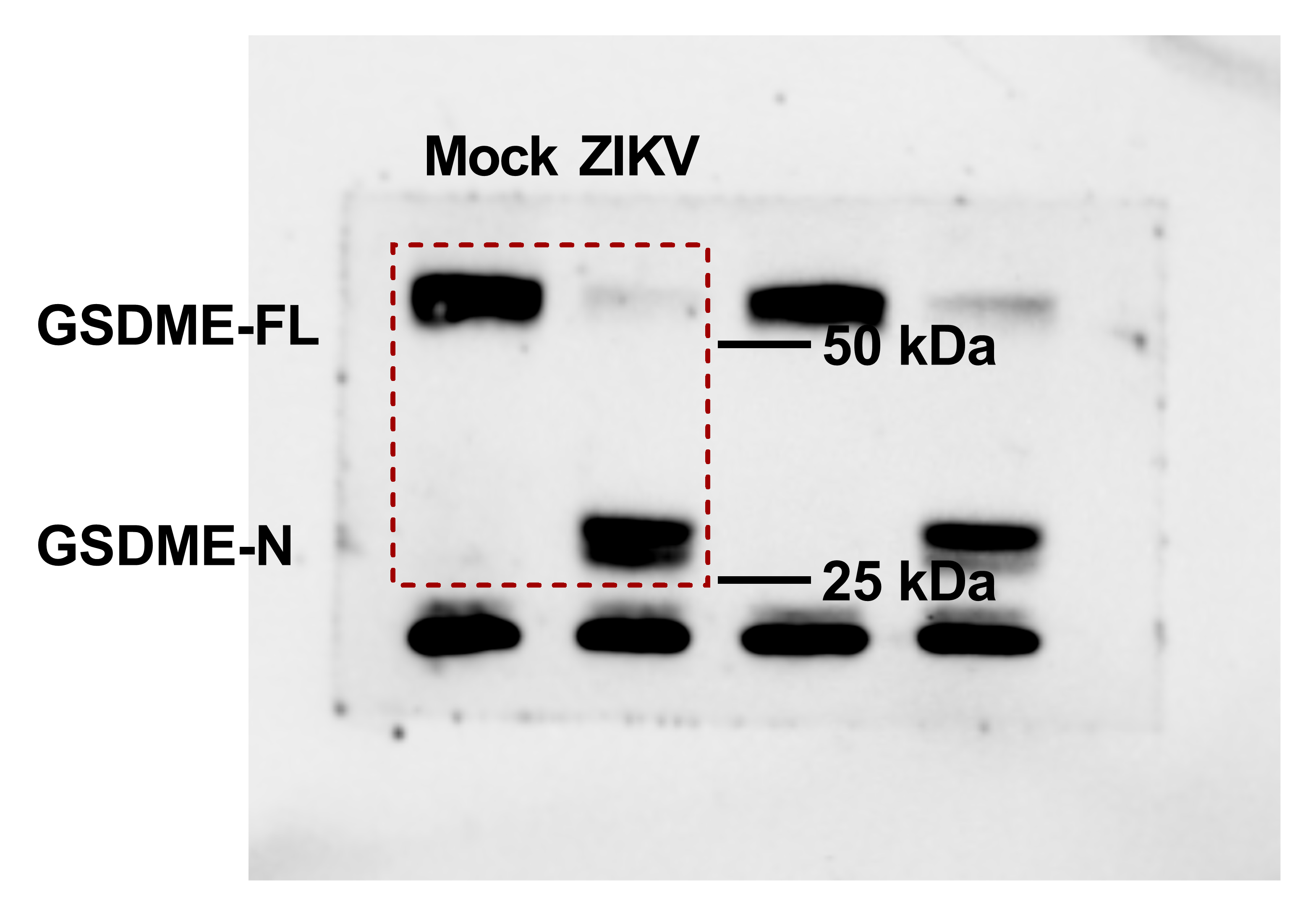

Supplement: Figure 3—figure supplement 1—source data 1. [file elife-73792-fig3-figsupp1-data1.zip › Figure 3-figure supplement 1-source data/1c/Figure 3-figure supplement 2 GSDME-labeled.tif]

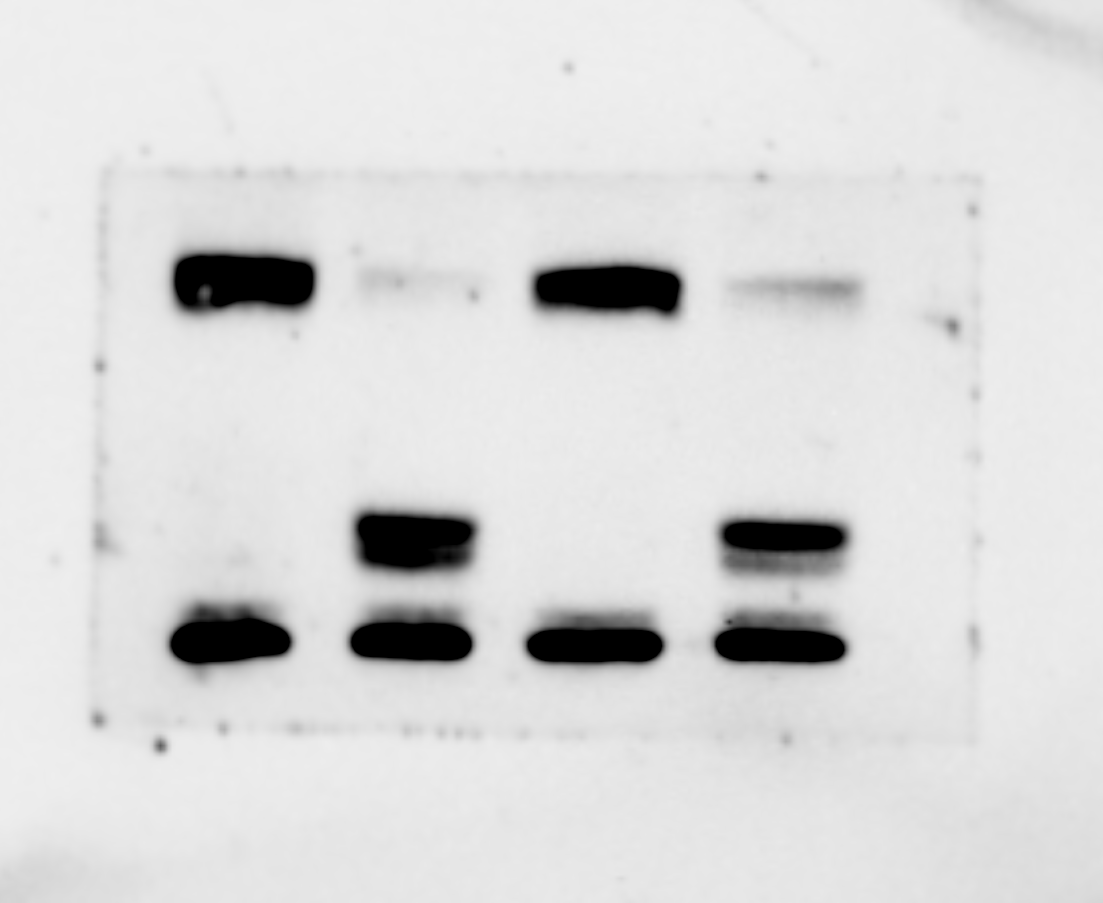

Supplement: Figure 3—figure supplement 1—source data 1. [file elife-73792-fig3-figsupp1-data1.zip › Figure 3-figure supplement 1-source data/1c/Figure 3-figure supplement 2 GSDME-raw.tif]

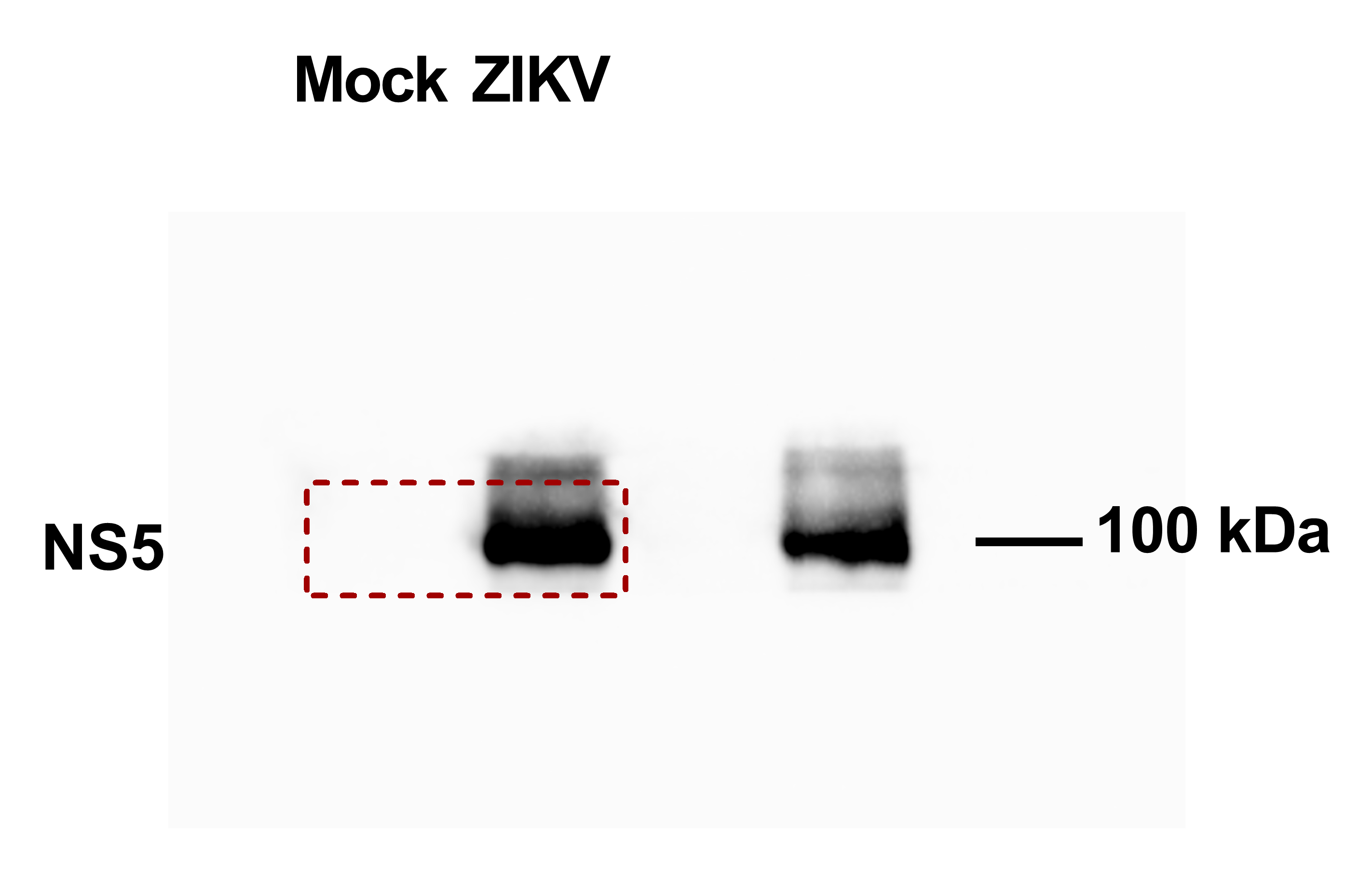

Supplement: Figure 3—figure supplement 1—source data 1. [file elife-73792-fig3-figsupp1-data1.zip › Figure 3-figure supplement 1-source data/1c/Figure 3-figure supplement 2 NS5-labeled.tif]

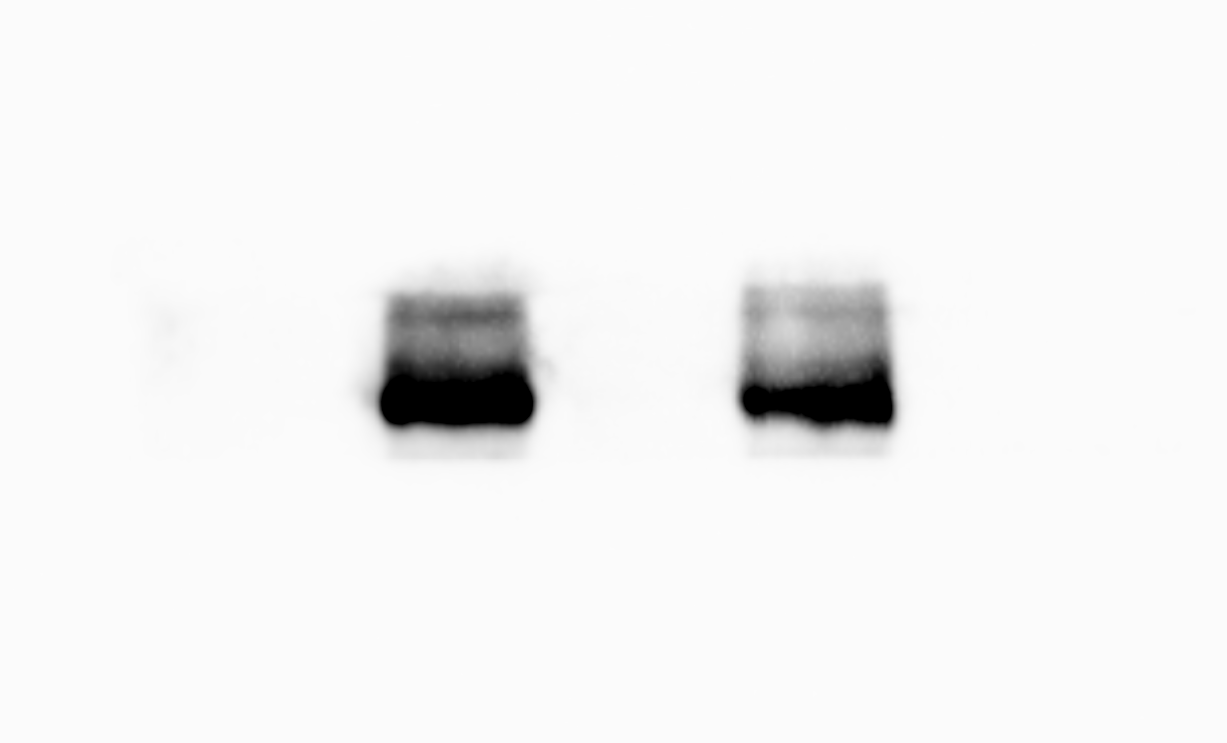

Supplement: Figure 3—figure supplement 1—source data 1. [file elife-73792-fig3-figsupp1-data1.zip › Figure 3-figure supplement 1-source data/1c/Figure 3-figure supplement 2 NS5-raw.tif]

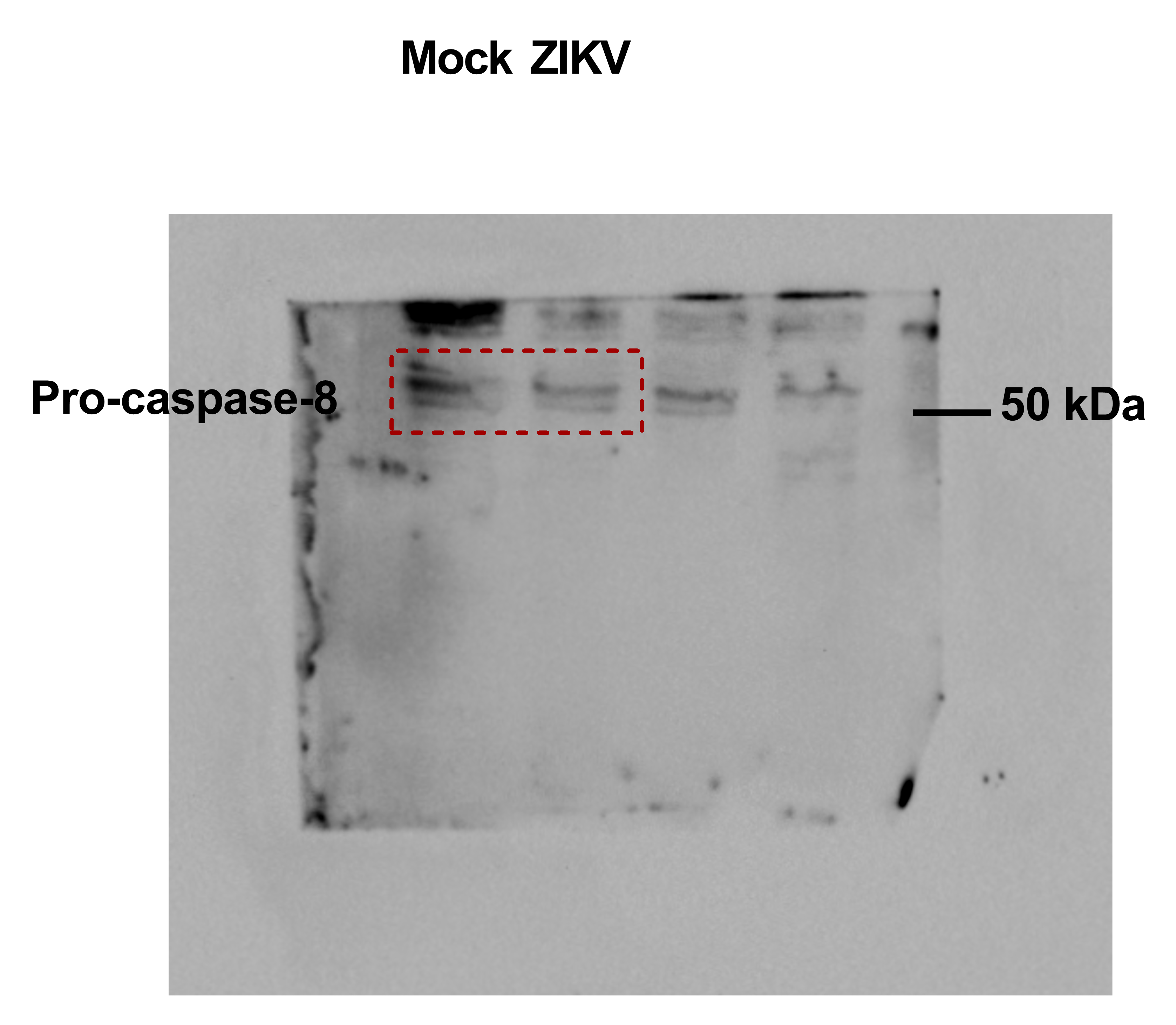

Supplement: Figure 3—figure supplement 1—source data 1. [file elife-73792-fig3-figsupp1-data1.zip › Figure 3-figure supplement 1-source data/1c/Figure 3-figure supplement 2 Pro caspase-8-labeled.tif]

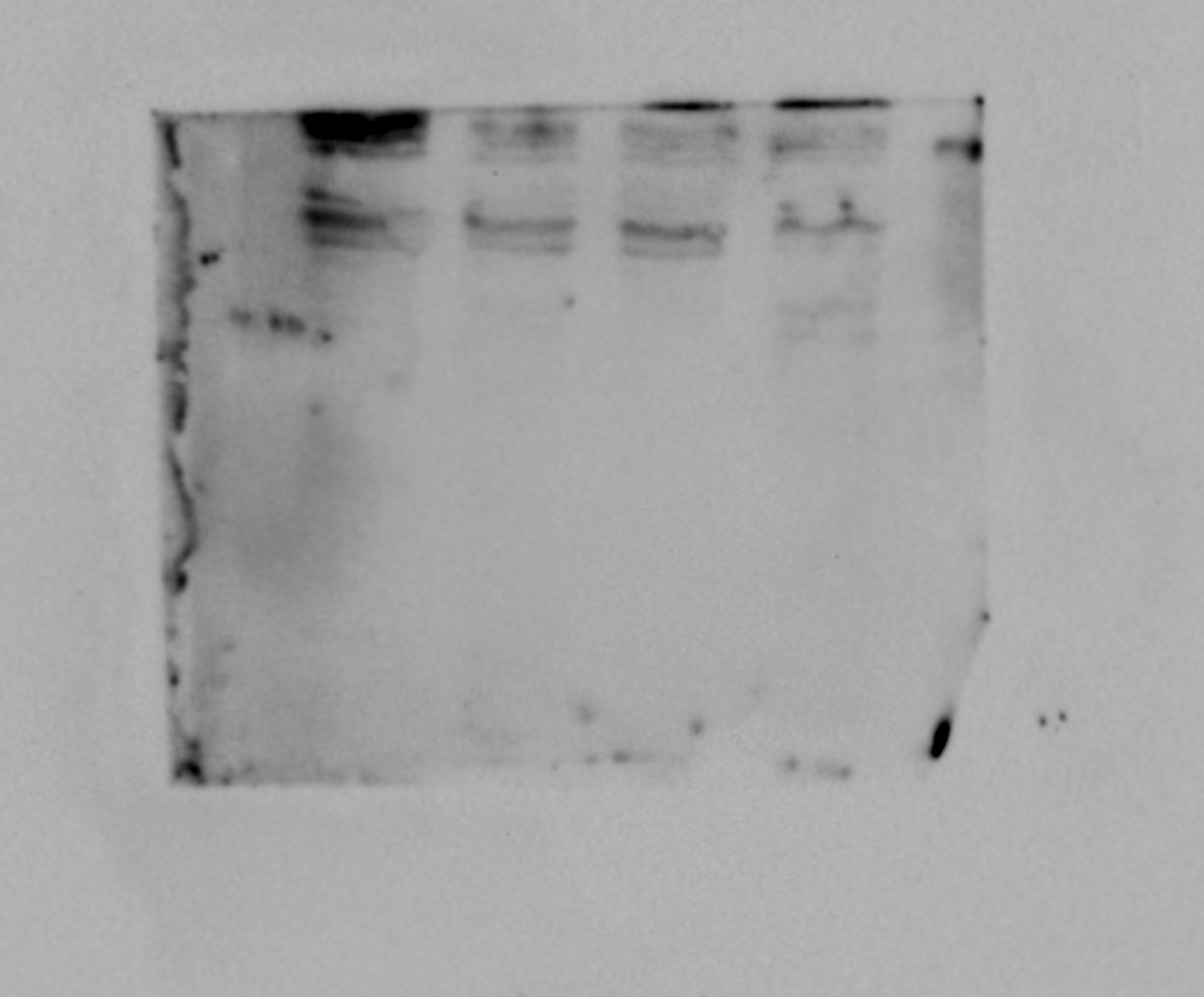

Supplement: Figure 3—figure supplement 1—source data 1. [file elife-73792-fig3-figsupp1-data1.zip › Figure 3-figure supplement 1-source data/1c/Figure 3-figure supplement 2 Pro caspase-8-raw.tif]

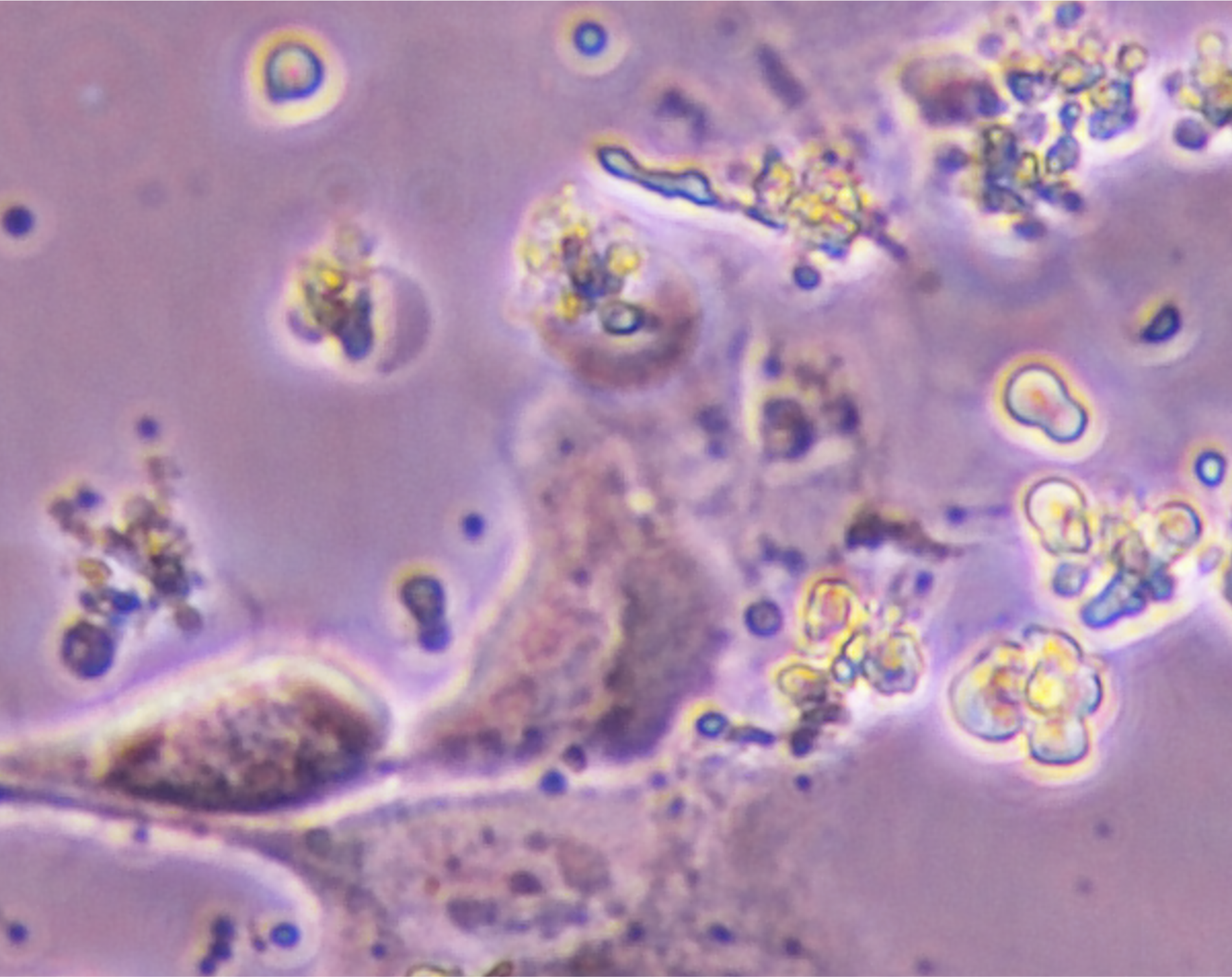

Supplement: Figure 4—source data 1. [file elife-73792-fig4-data1.zip › Figure 4-source data 1/Fig 4A/3 utr.tif]

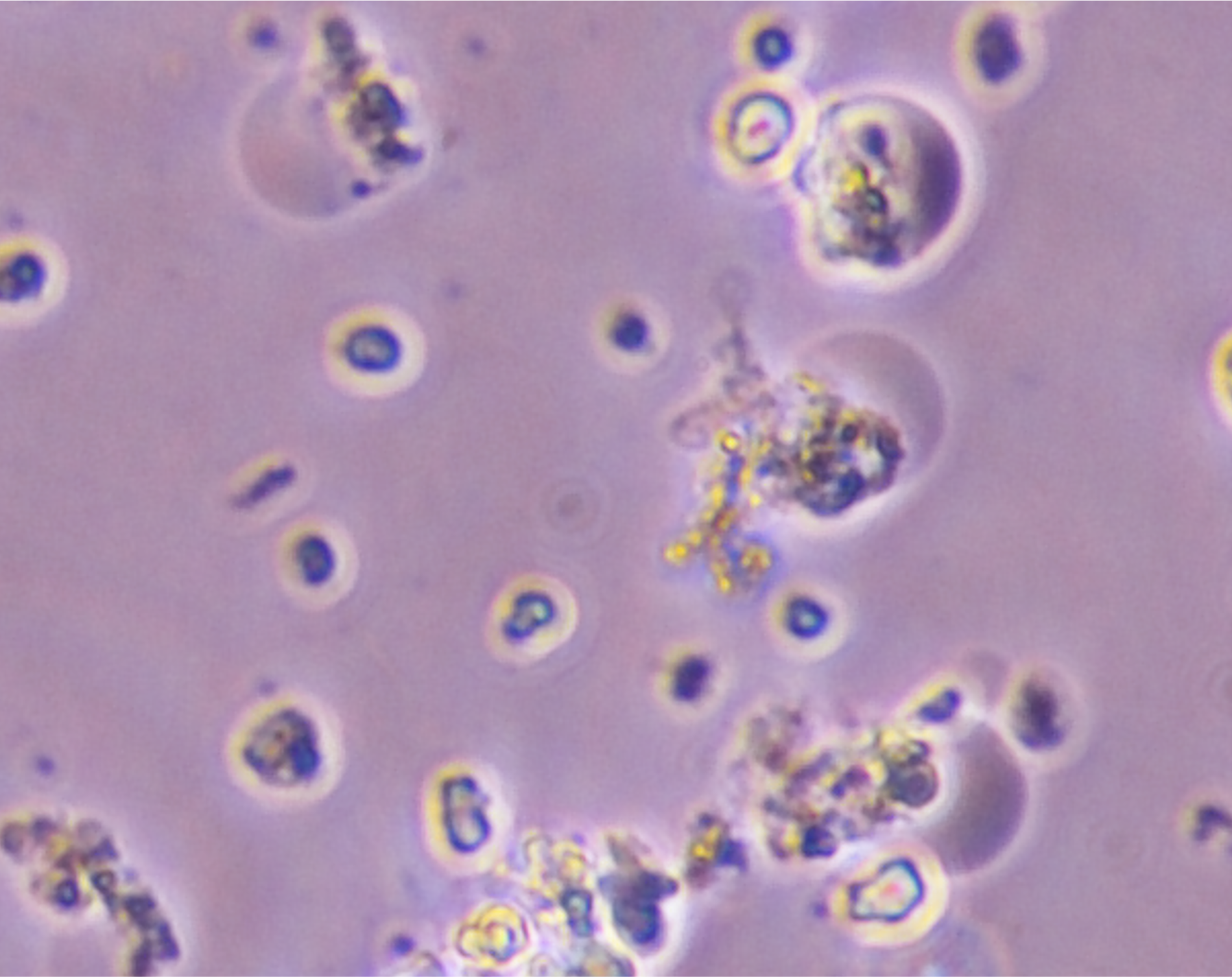

Supplement: Figure 4—source data 1. [file elife-73792-fig4-data1.zip › Figure 4-source data 1/Fig 4A/5 utr.tif]

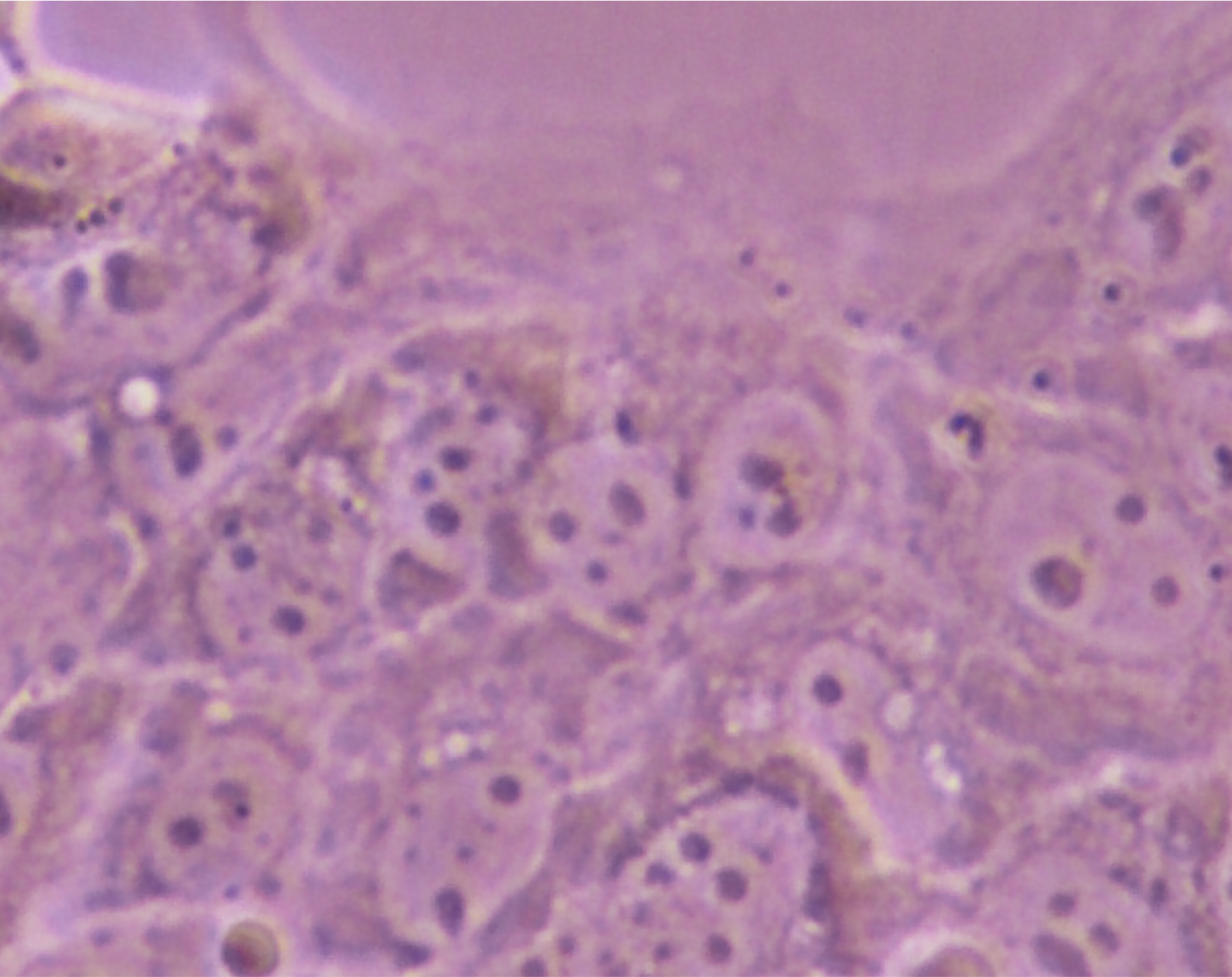

Supplement: Figure 4—source data 1. [file elife-73792-fig4-data1.zip › Figure 4-source data 1/Fig 4A/control.tif]

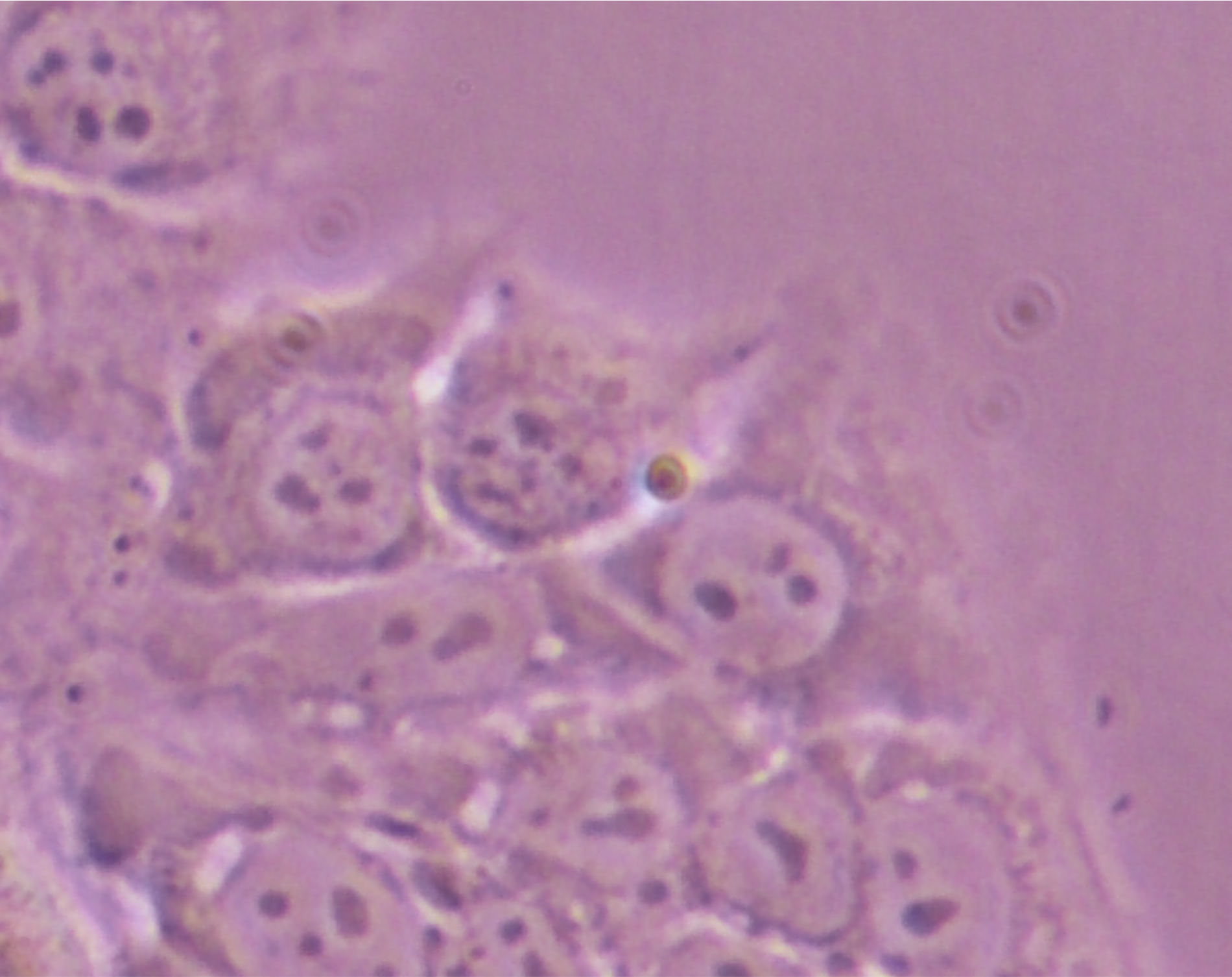

Supplement: Figure 4—source data 1. [file elife-73792-fig4-data1.zip › Figure 4-source data 1/Fig 4A/lipo2000.tif]

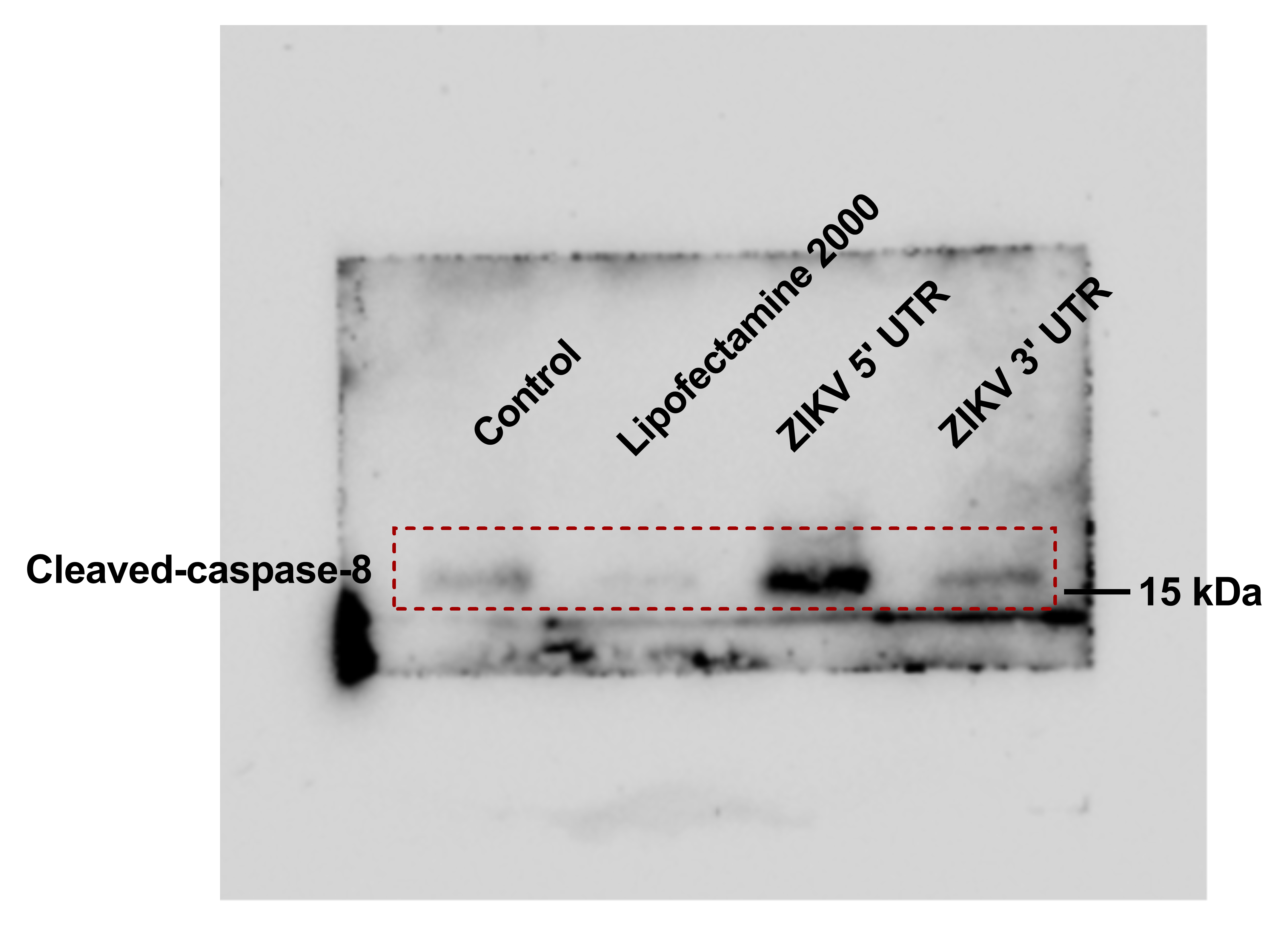

Supplement: Figure 4—source data 1. [file elife-73792-fig4-data1.zip › Figure 4-source data 1/Fig 4C/Figure 4C Cleased-caspase-8-labeled.tif]

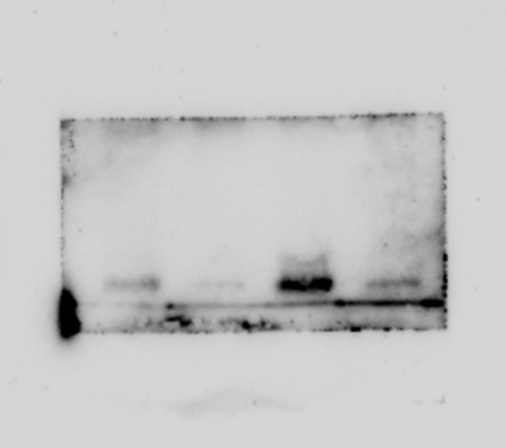

Supplement: Figure 4—source data 1. [file elife-73792-fig4-data1.zip › Figure 4-source data 1/Fig 4C/Figure 4C Cleased-caspase-8-raw.tif]

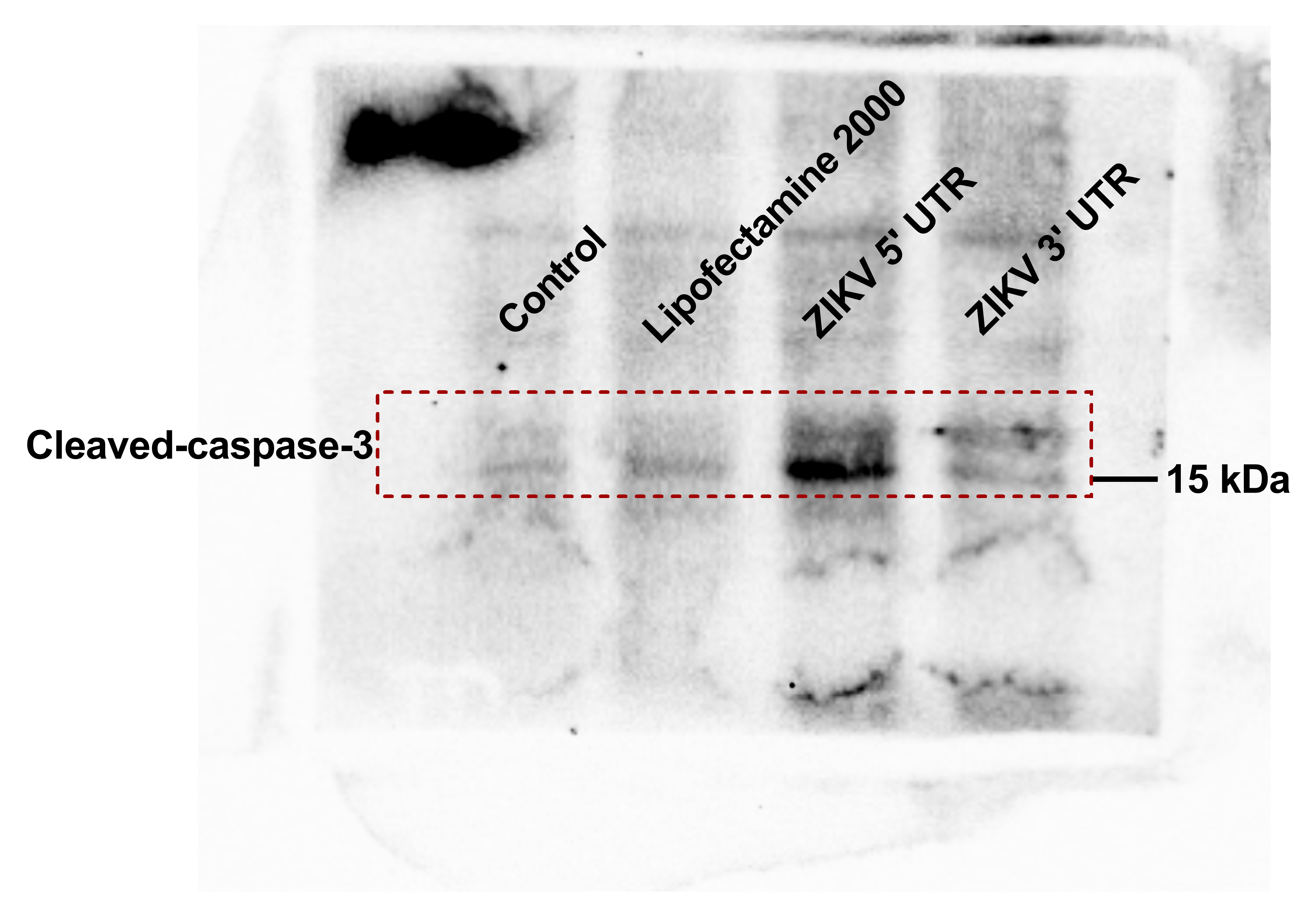

Supplement: Figure 4—source data 1. [file elife-73792-fig4-data1.zip › Figure 4-source data 1/Fig 4C/Figure 4C Cleaved-caspase-3-labeled.tif]

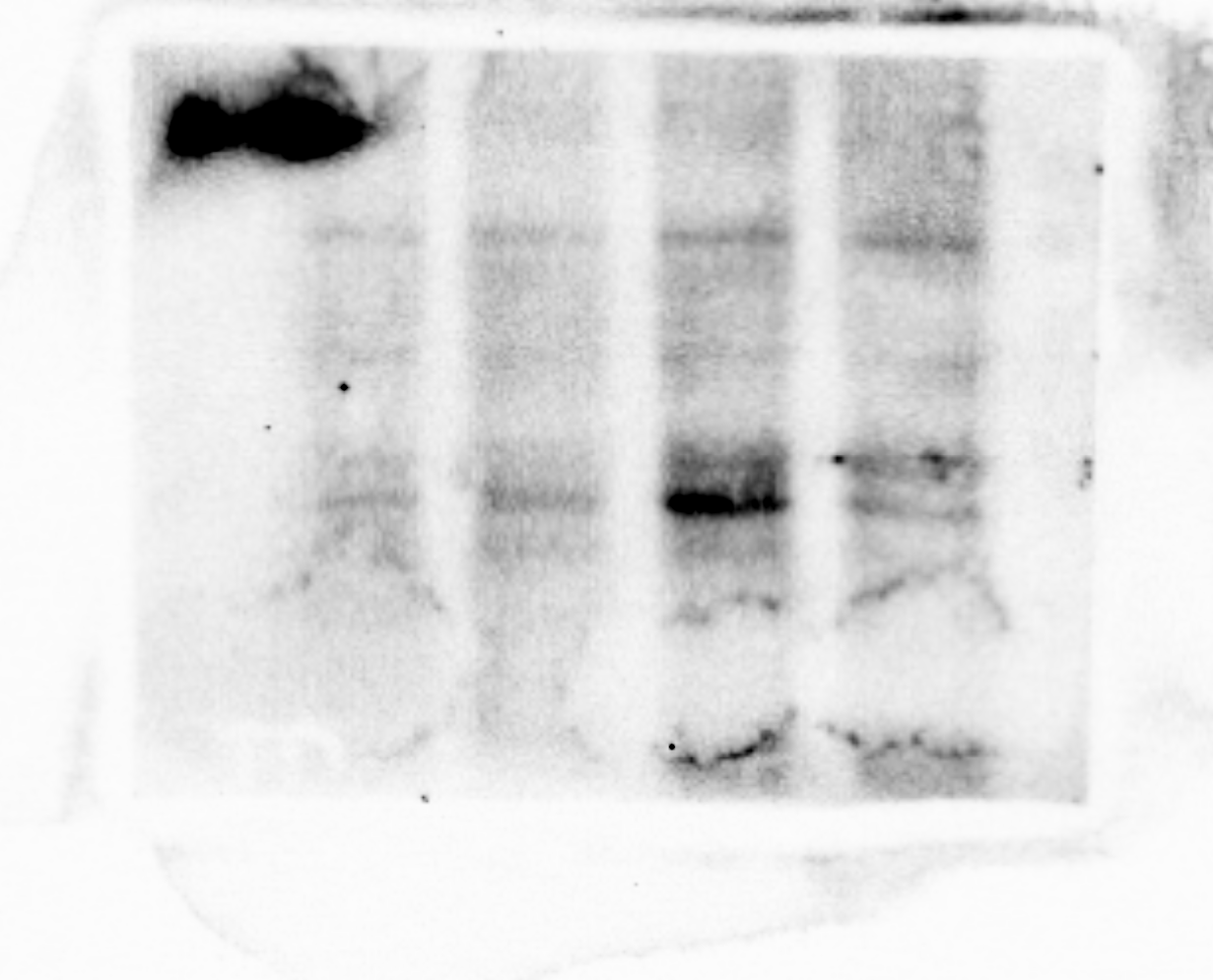

Supplement: Figure 4—source data 1. [file elife-73792-fig4-data1.zip › Figure 4-source data 1/Fig 4C/Figure 4C Cleaved-caspase-3-raw.tif]

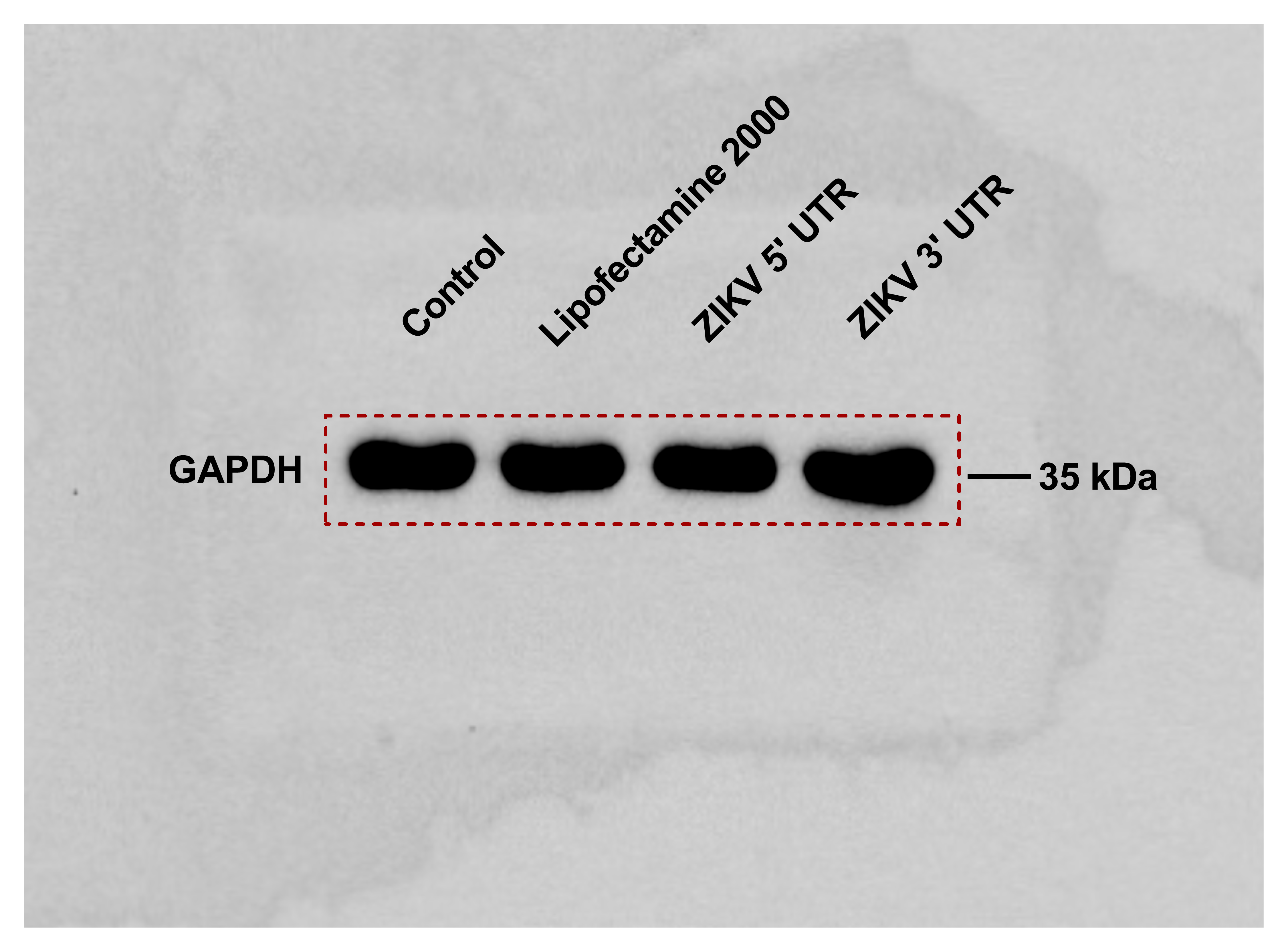

Supplement: Figure 4—source data 1. [file elife-73792-fig4-data1.zip › Figure 4-source data 1/Fig 4C/Figure 4C GAPDH-labeled.tif]

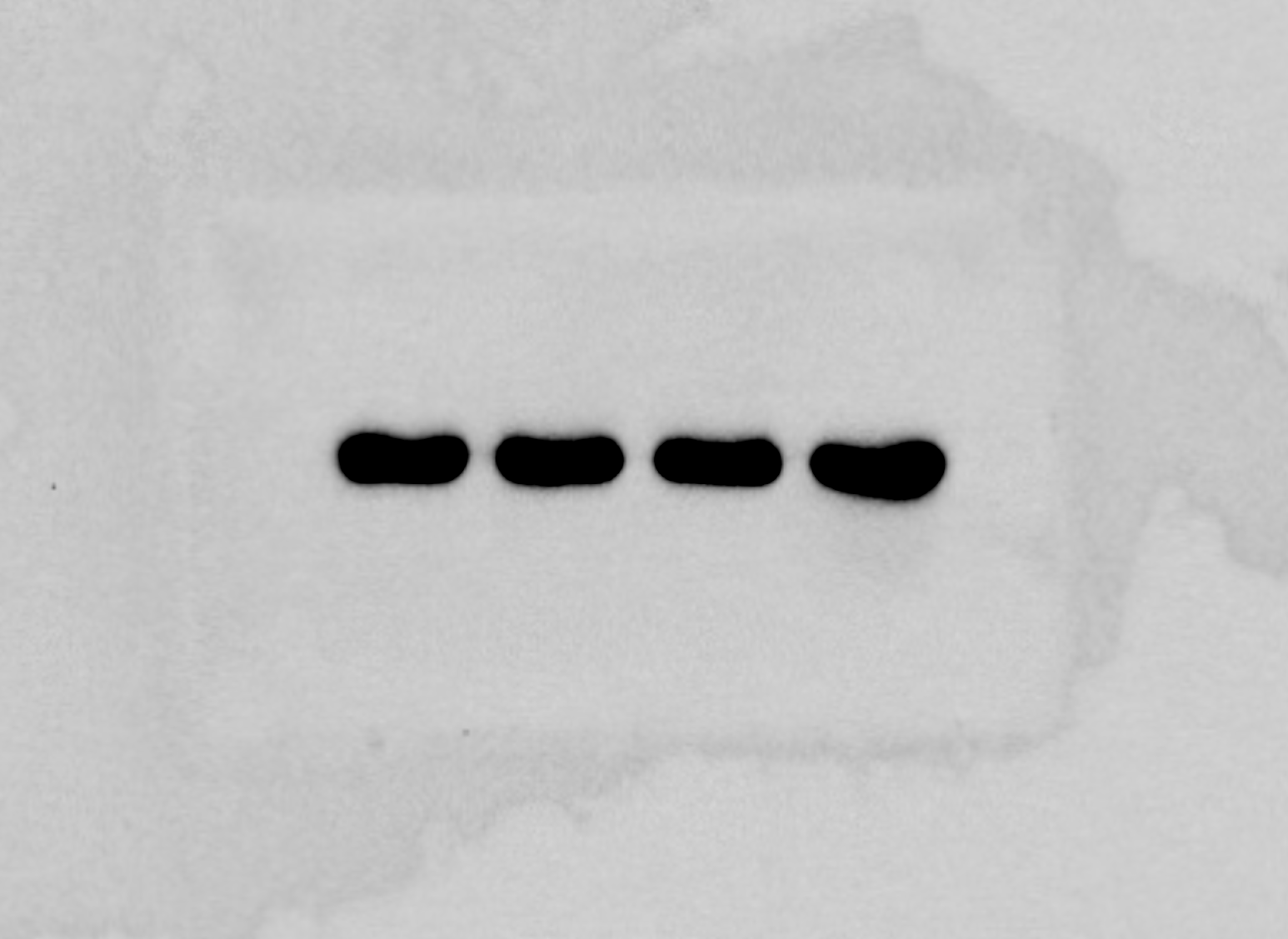

Supplement: Figure 4—source data 1. [file elife-73792-fig4-data1.zip › Figure 4-source data 1/Fig 4C/Figure 4C GAPDH-raw.tif]

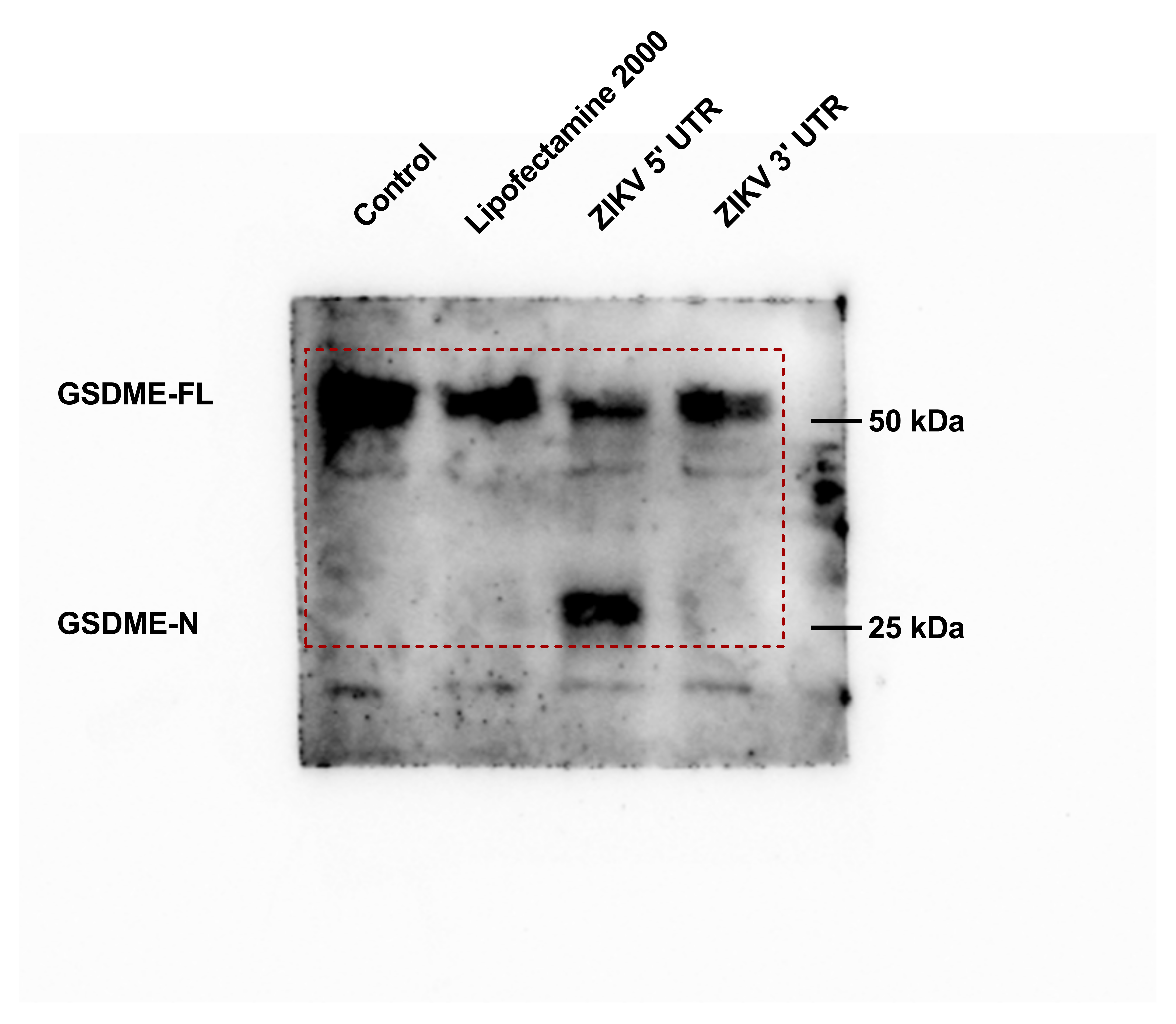

Supplement: Figure 4—source data 1. [file elife-73792-fig4-data1.zip › Figure 4-source data 1/Fig 4C/Figure 4C GSDME-labeled.tif]

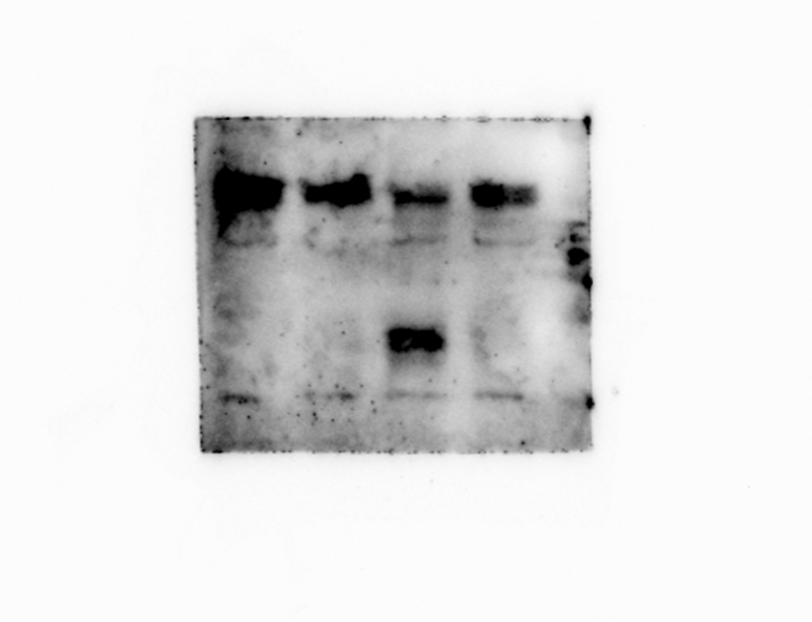

Supplement: Figure 4—source data 1. [file elife-73792-fig4-data1.zip › Figure 4-source data 1/Fig 4C/Figure 4C GSDME-raw.tif]

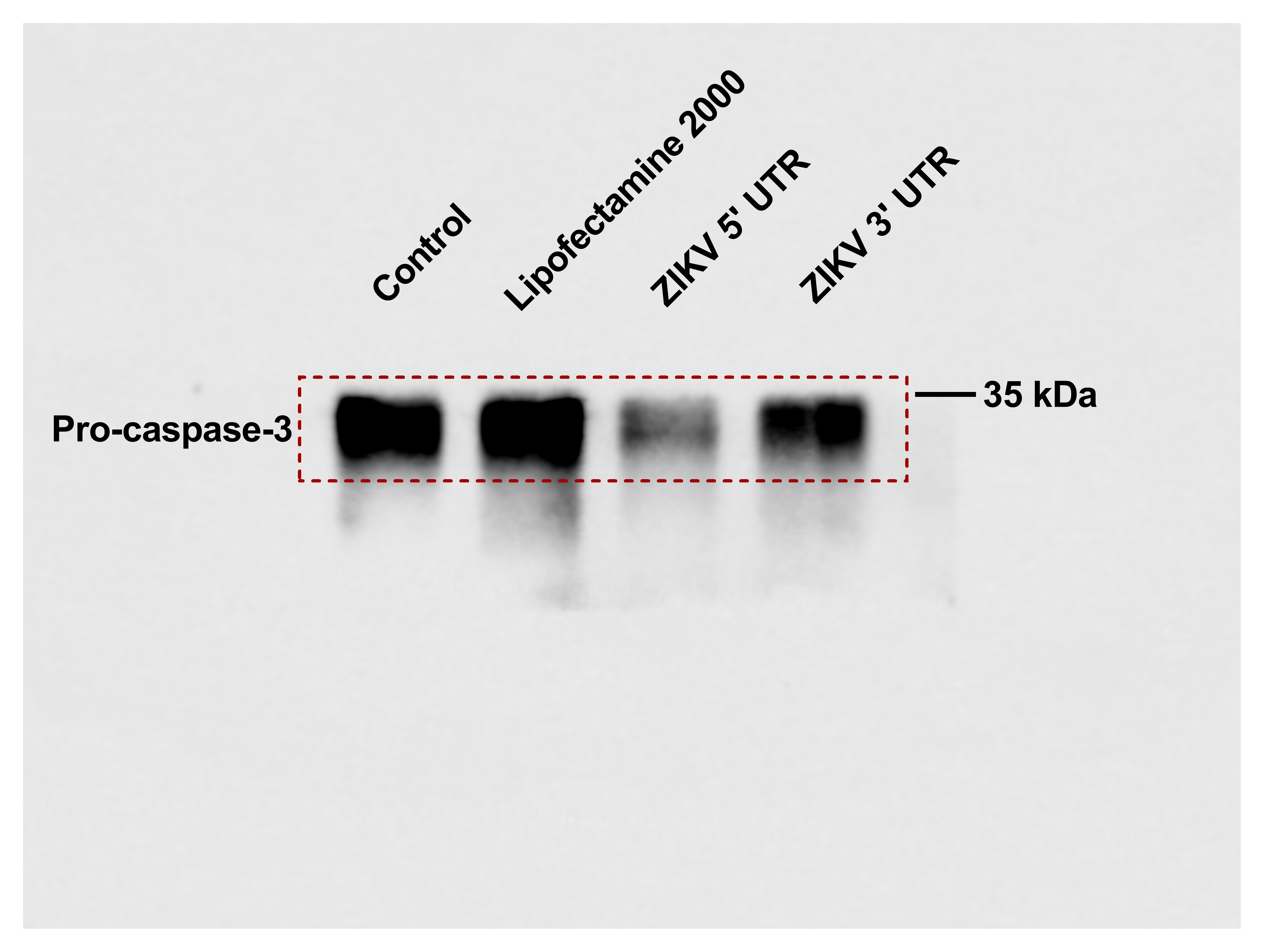

Supplement: Figure 4—source data 1. [file elife-73792-fig4-data1.zip › Figure 4-source data 1/Fig 4C/Figure 4C Pro-caspase-3-labeled.tif]

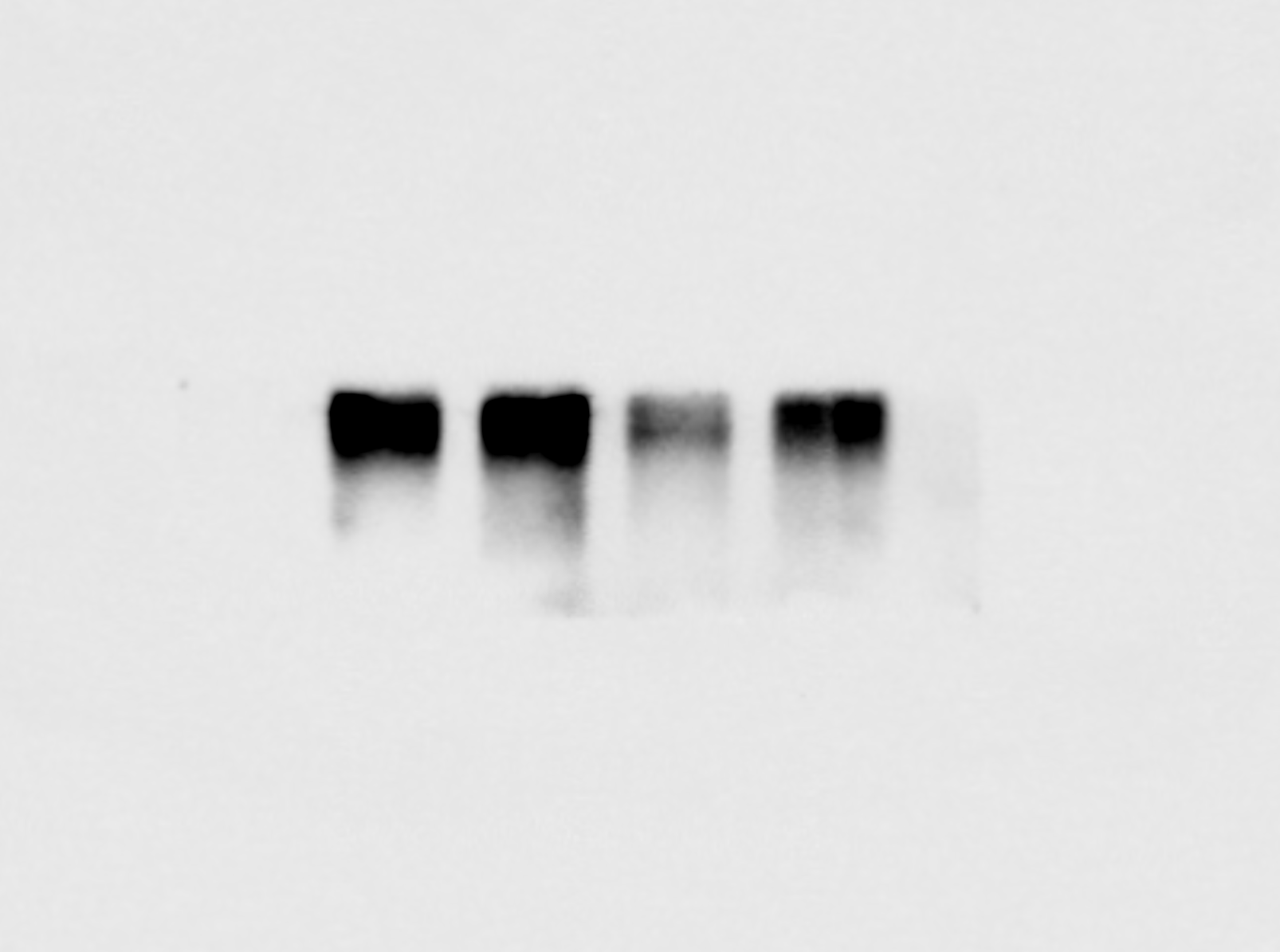

Supplement: Figure 4—source data 1. [file elife-73792-fig4-data1.zip › Figure 4-source data 1/Fig 4C/Figure 4C Pro-caspase-3-raw.tif]

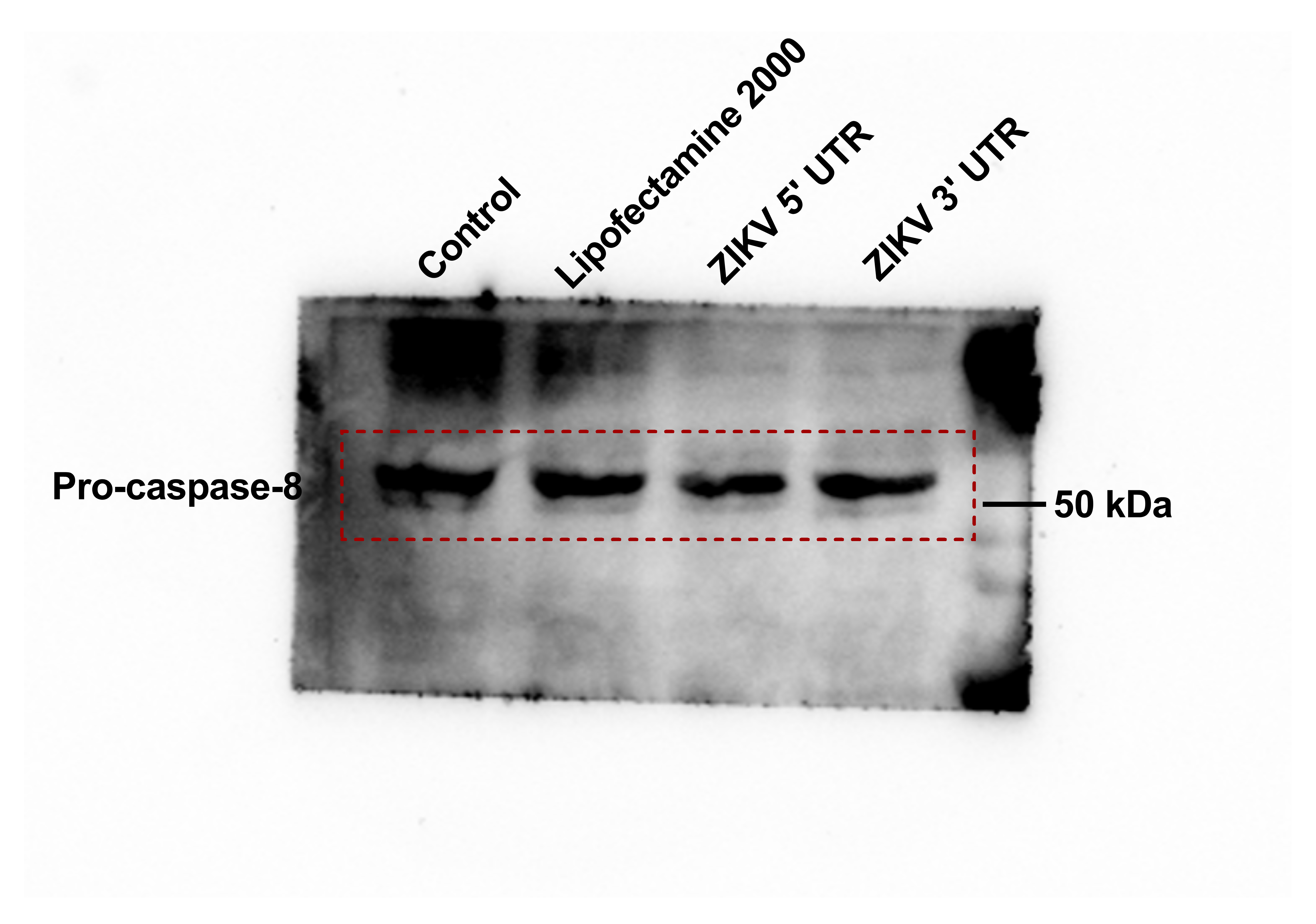

Supplement: Figure 4—source data 1. [file elife-73792-fig4-data1.zip › Figure 4-source data 1/Fig 4C/Figure 4C Pro-caspase-8-labeled.tif]

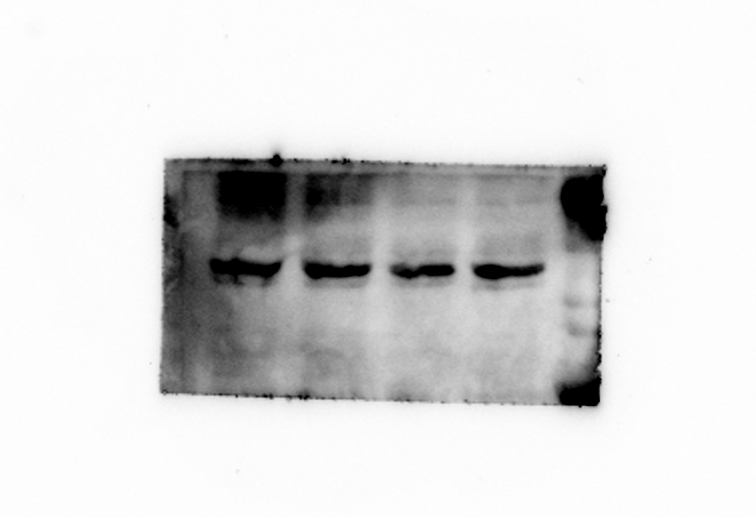

Supplement: Figure 4—source data 1. [file elife-73792-fig4-data1.zip › Figure 4-source data 1/Fig 4C/Figure 4C Pro-caspase-8-raw.tif]

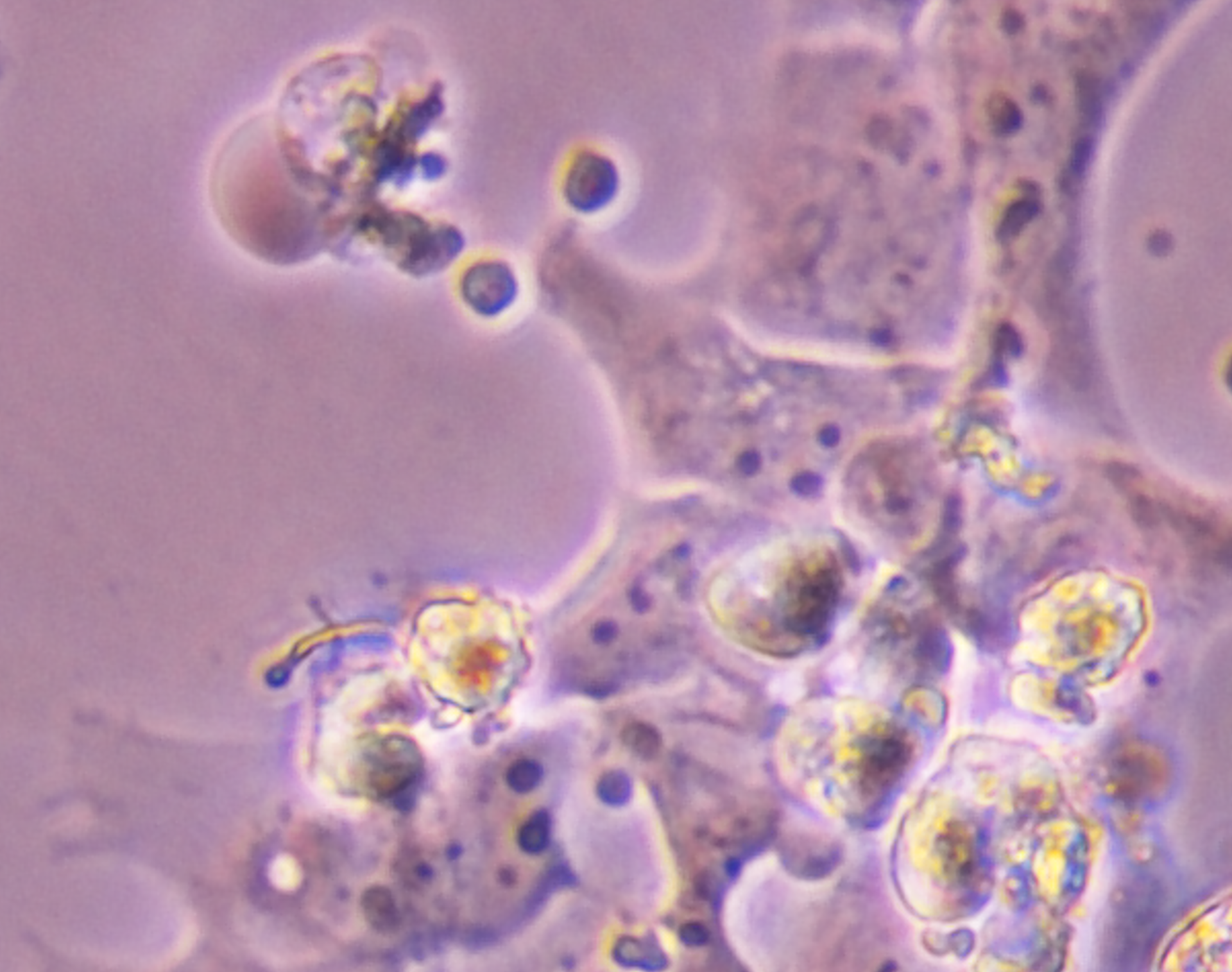

Supplement: Figure 4—source data 1. [file elife-73792-fig4-data1.zip › Figure 4-source data 1/Fig 4D/rig i kc zikv.tif]

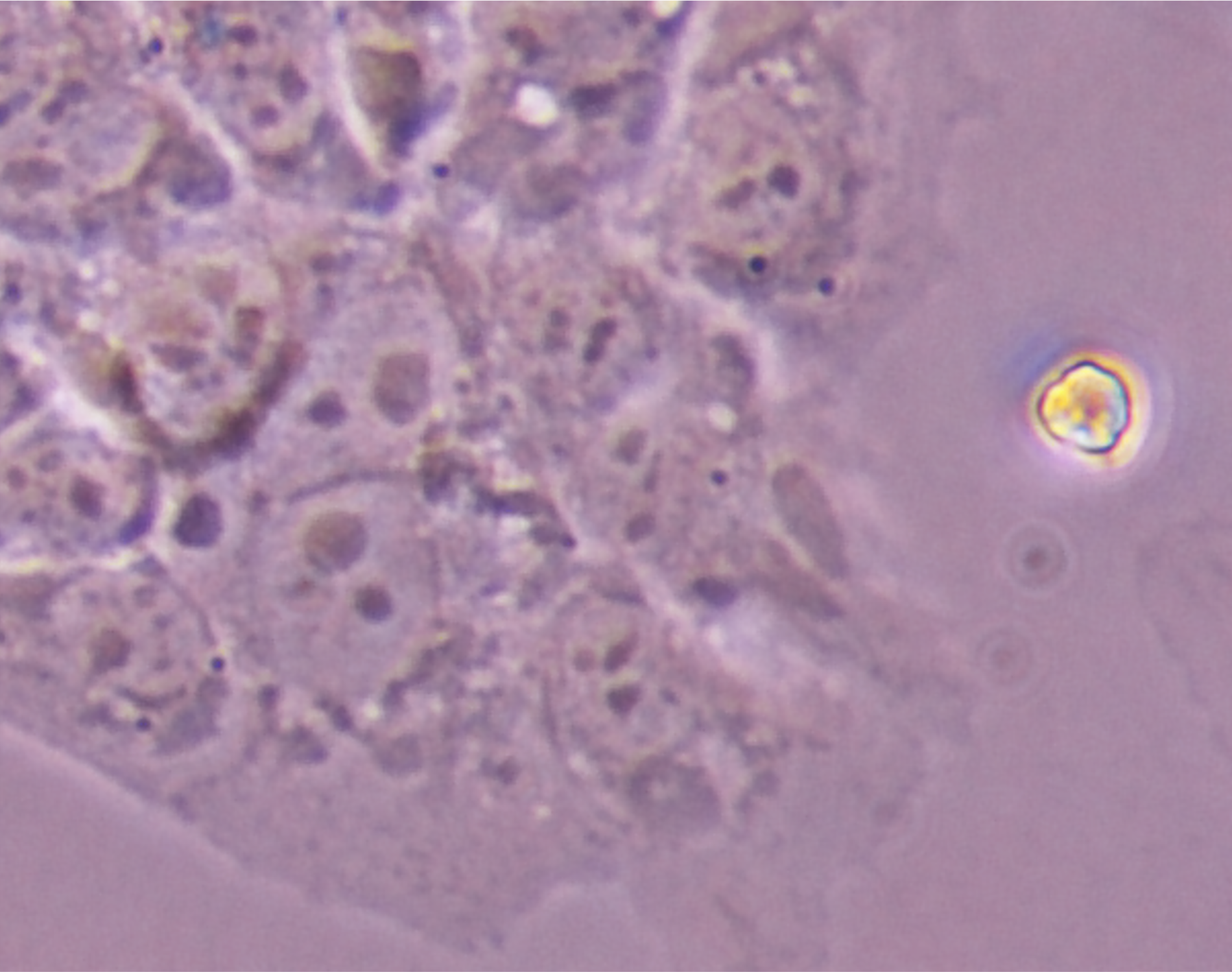

Supplement: Figure 4—source data 1. [file elife-73792-fig4-data1.zip › Figure 4-source data 1/Fig 4D/rigi kd mock.tif]

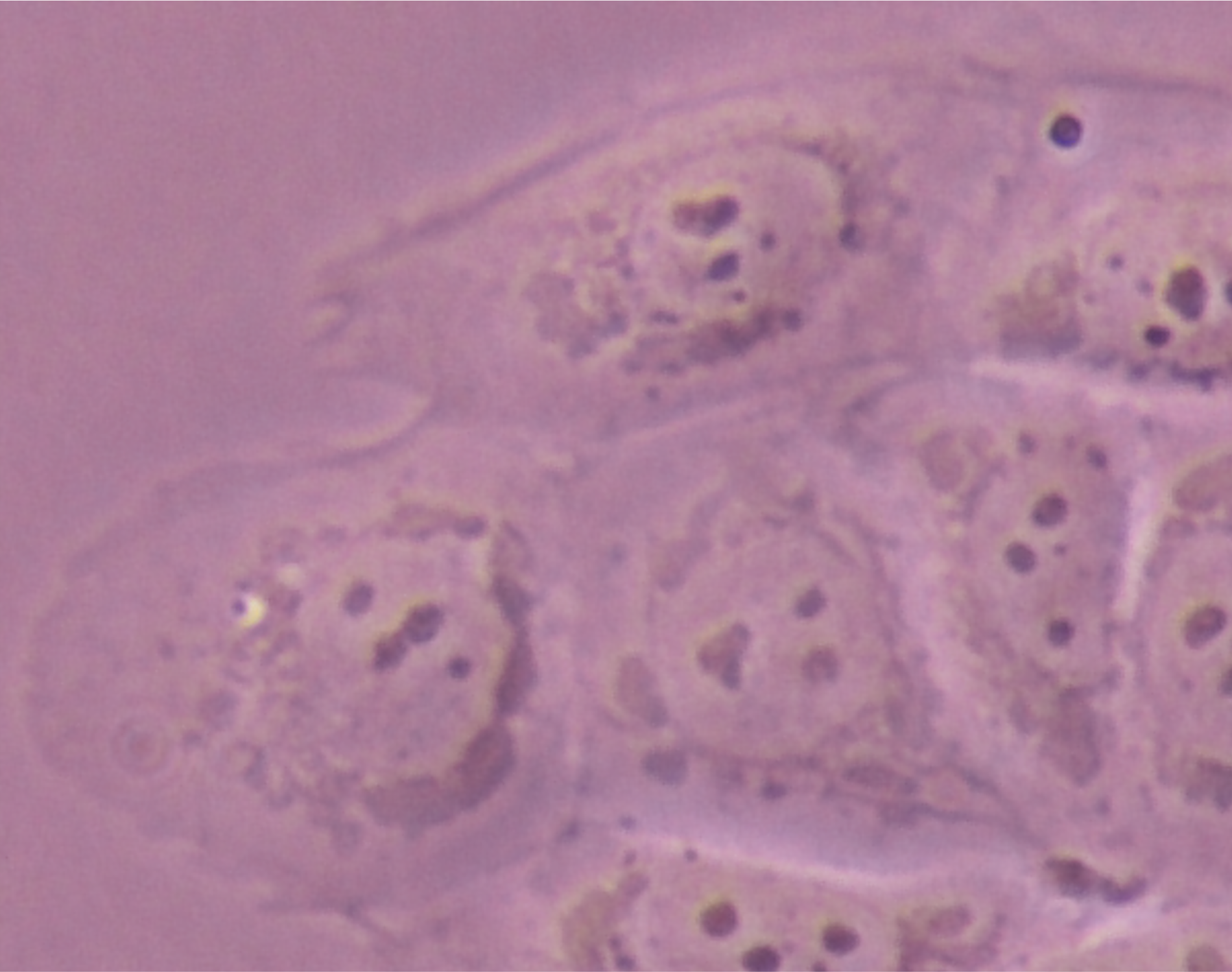

Supplement: Figure 4—source data 1. [file elife-73792-fig4-data1.zip › Figure 4-source data 1/Fig 4D/tlr7 kd mock.tif]

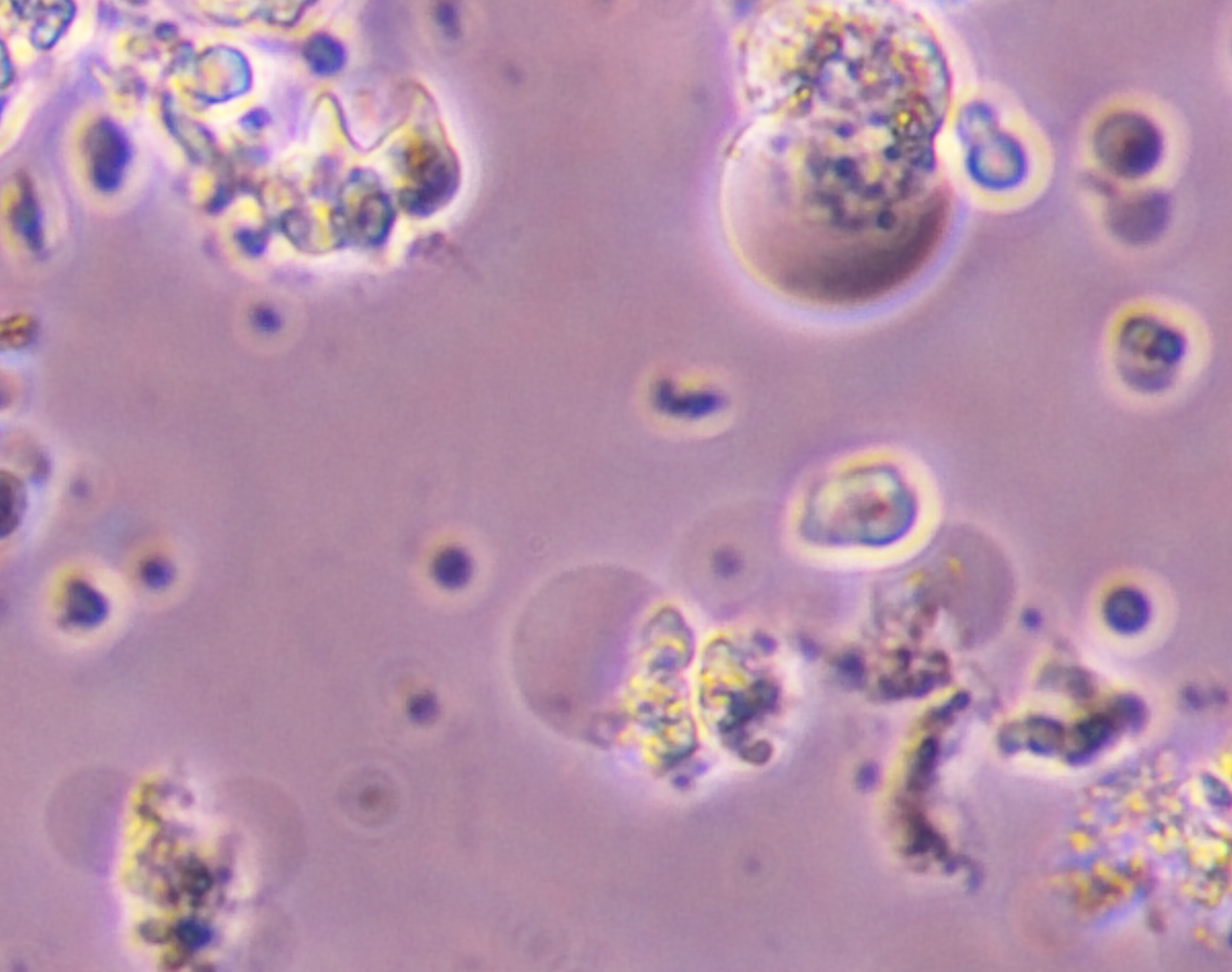

Supplement: Figure 4—source data 1. [file elife-73792-fig4-data1.zip › Figure 4-source data 1/Fig 4D/tlr7 kd zikv.tif]

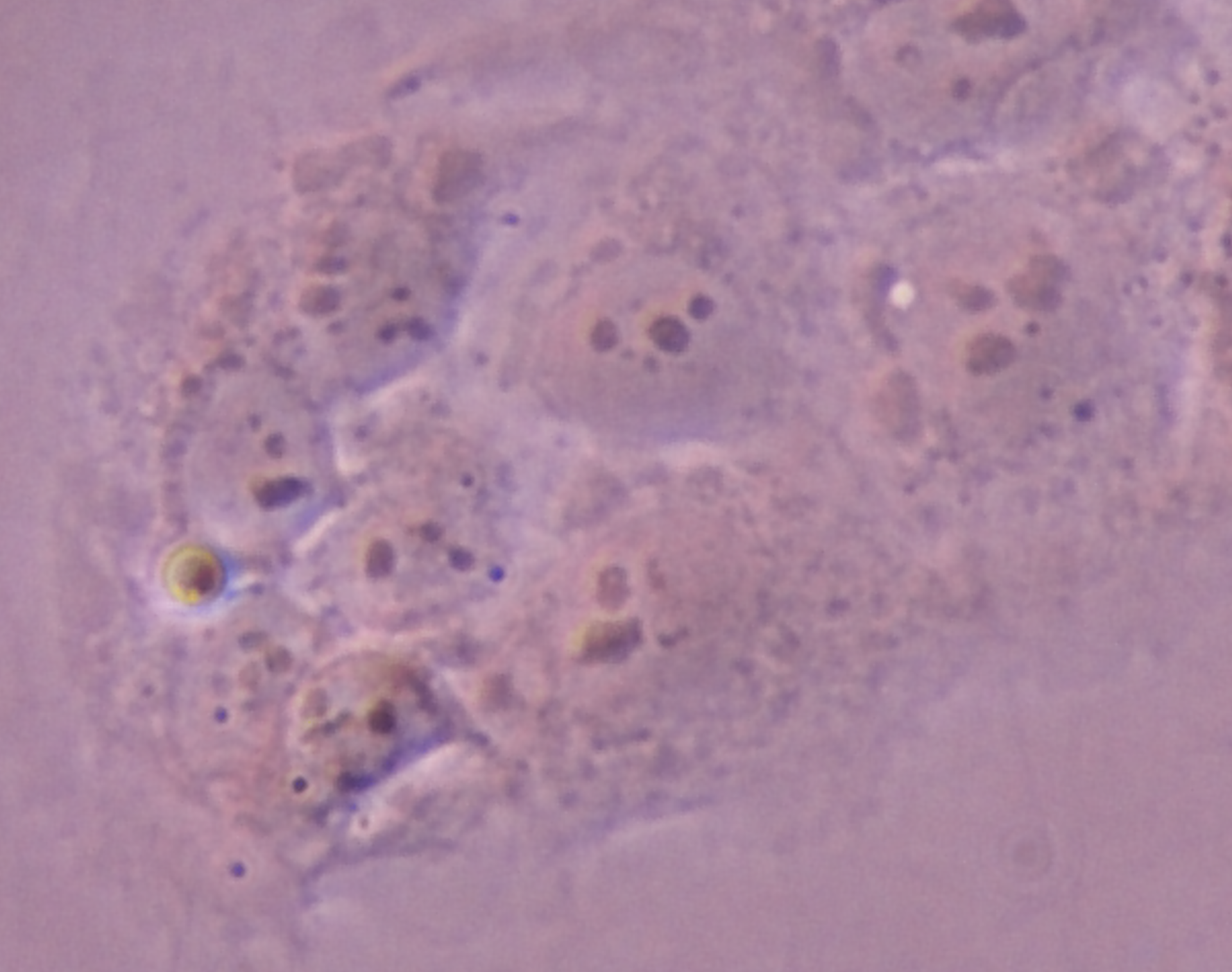

Supplement: Figure 4—source data 1. [file elife-73792-fig4-data1.zip › Figure 4-source data 1/Fig 4D/tlr8 kd mock.tif]

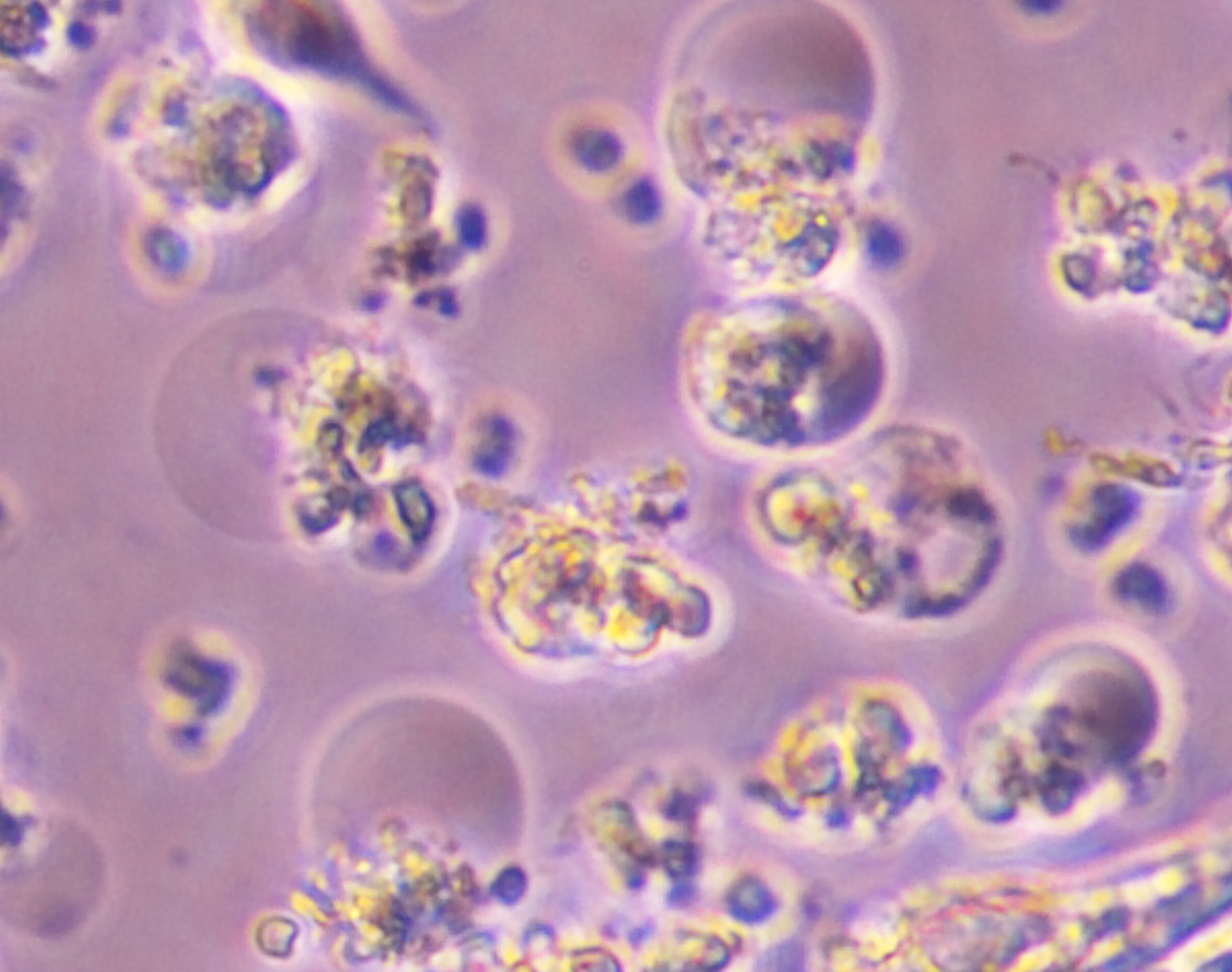

Supplement: Figure 4—source data 1. [file elife-73792-fig4-data1.zip › Figure 4-source data 1/Fig 4D/tlr8 kd zikv.tif]

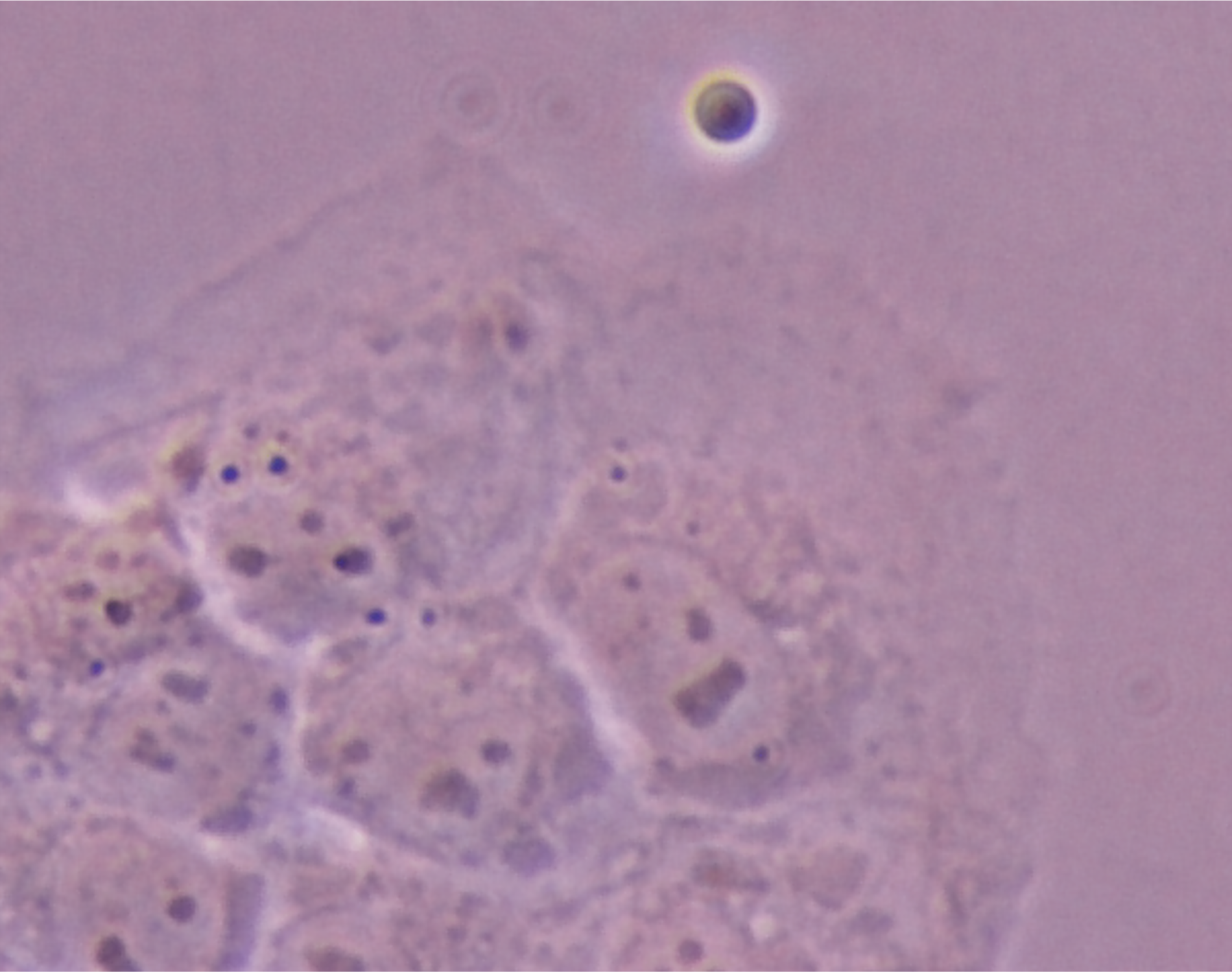

Supplement: Figure 4—source data 1. [file elife-73792-fig4-data1.zip › Figure 4-source data 1/Fig 4D/wt mock.tif]

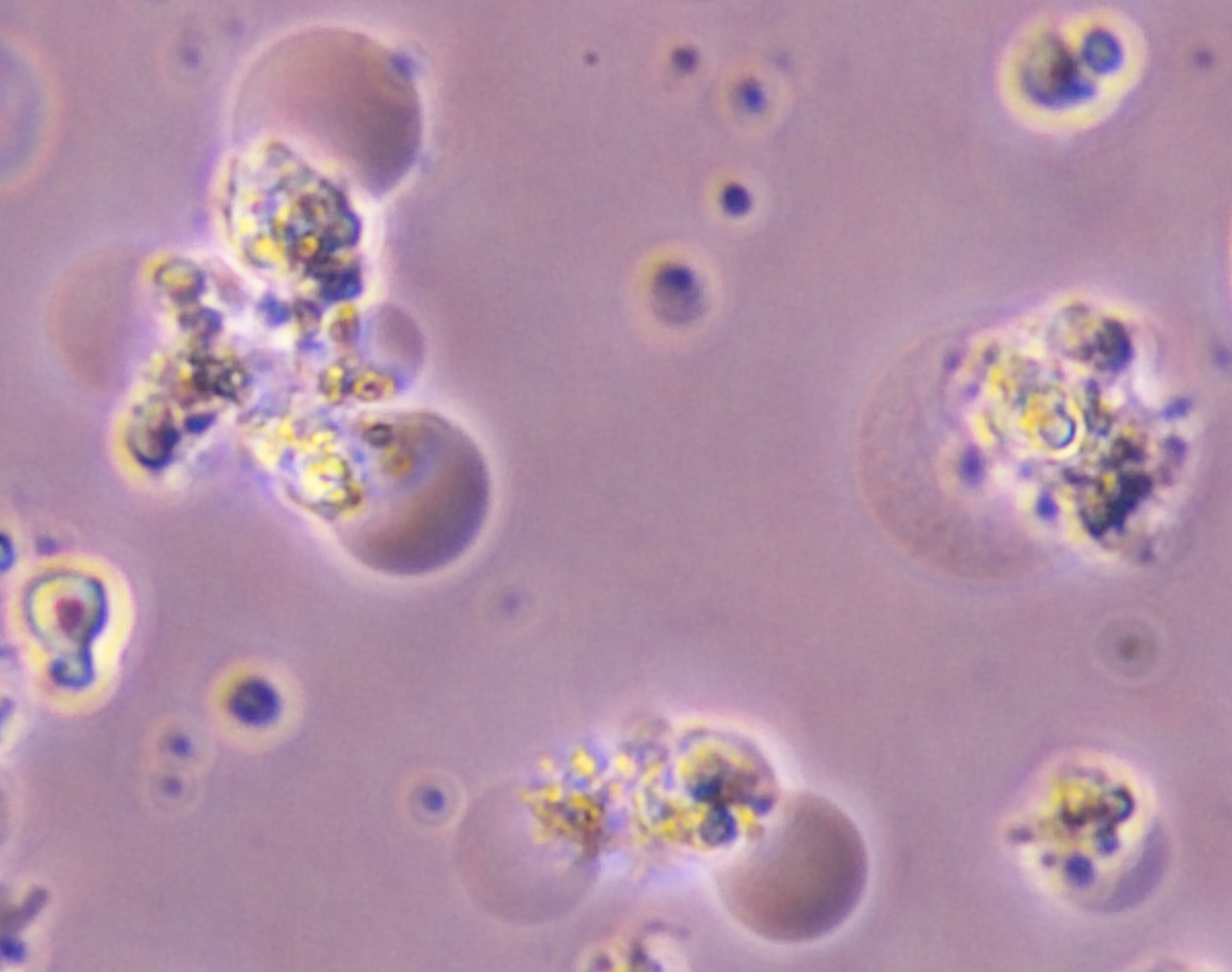

Supplement: Figure 4—source data 1. [file elife-73792-fig4-data1.zip › Figure 4-source data 1/Fig 4D/wt zikv.tif]

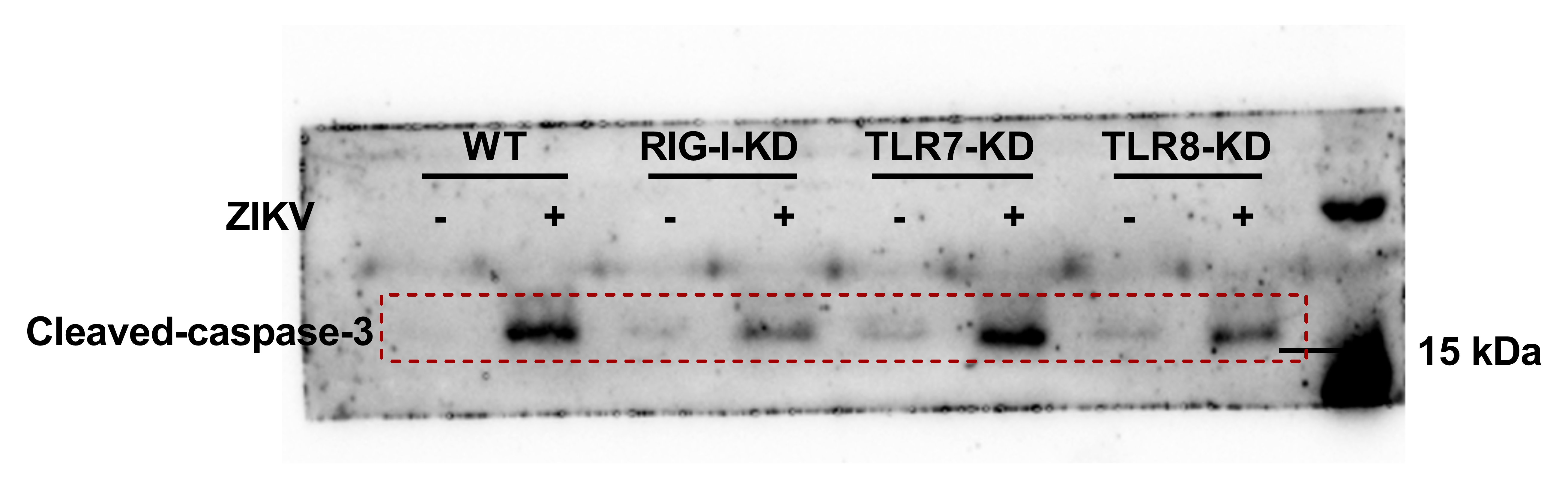

Supplement: Figure 4—source data 1. [file elife-73792-fig4-data1.zip › Figure 4-source data 1/Fig 4F/Figure 4F Cleaved-caspase-3-labeled.tif]

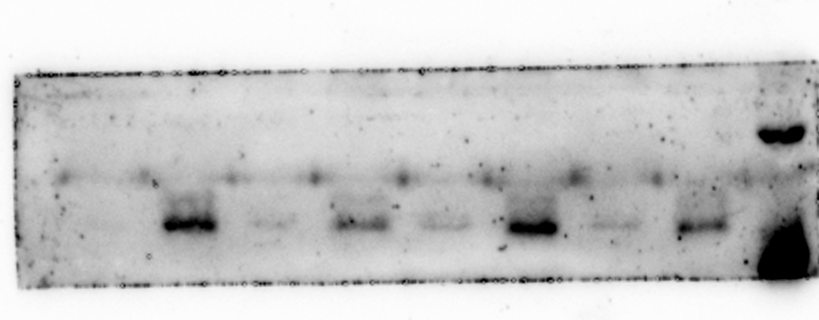

Supplement: Figure 4—source data 1. [file elife-73792-fig4-data1.zip › Figure 4-source data 1/Fig 4F/Figure 4F Cleaved-caspase-3-raw.tif]

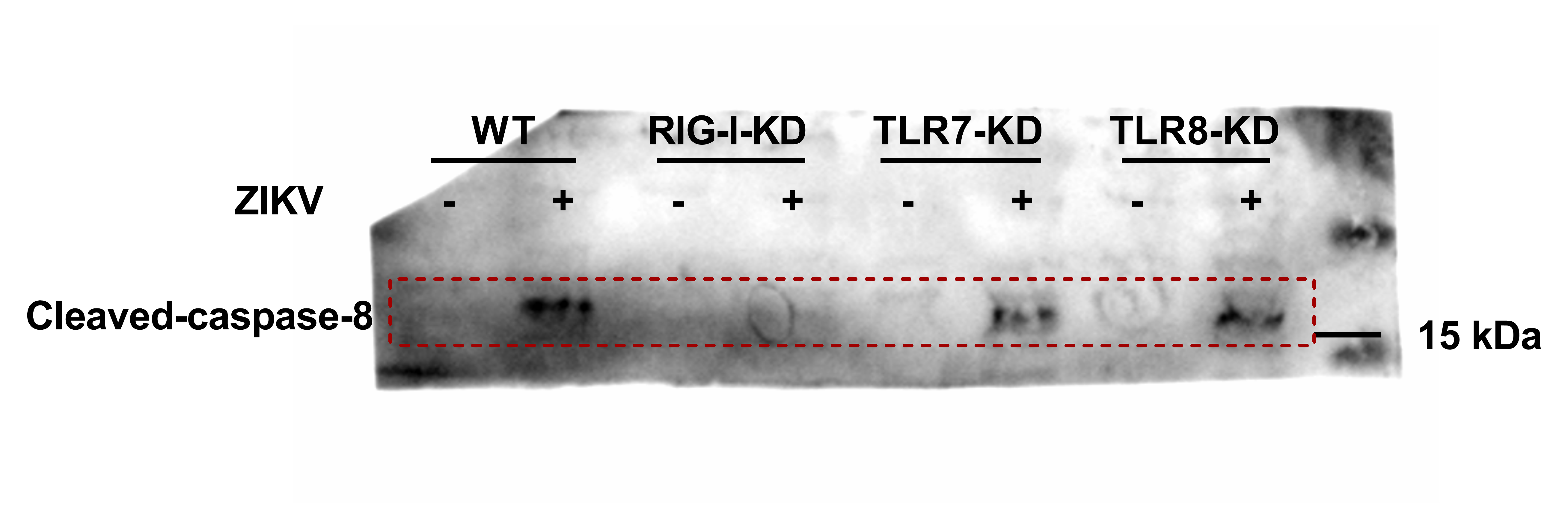

Supplement: Figure 4—source data 1. [file elife-73792-fig4-data1.zip › Figure 4-source data 1/Fig 4F/Figure 4F Cleaved-caspase-8-labeled.tif]

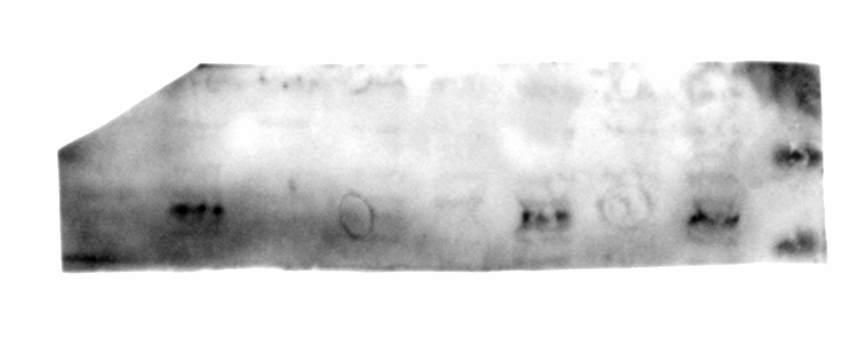

Supplement: Figure 4—source data 1. [file elife-73792-fig4-data1.zip › Figure 4-source data 1/Fig 4F/Figure 4F Cleaved-caspase-8-raw.tif.tif]

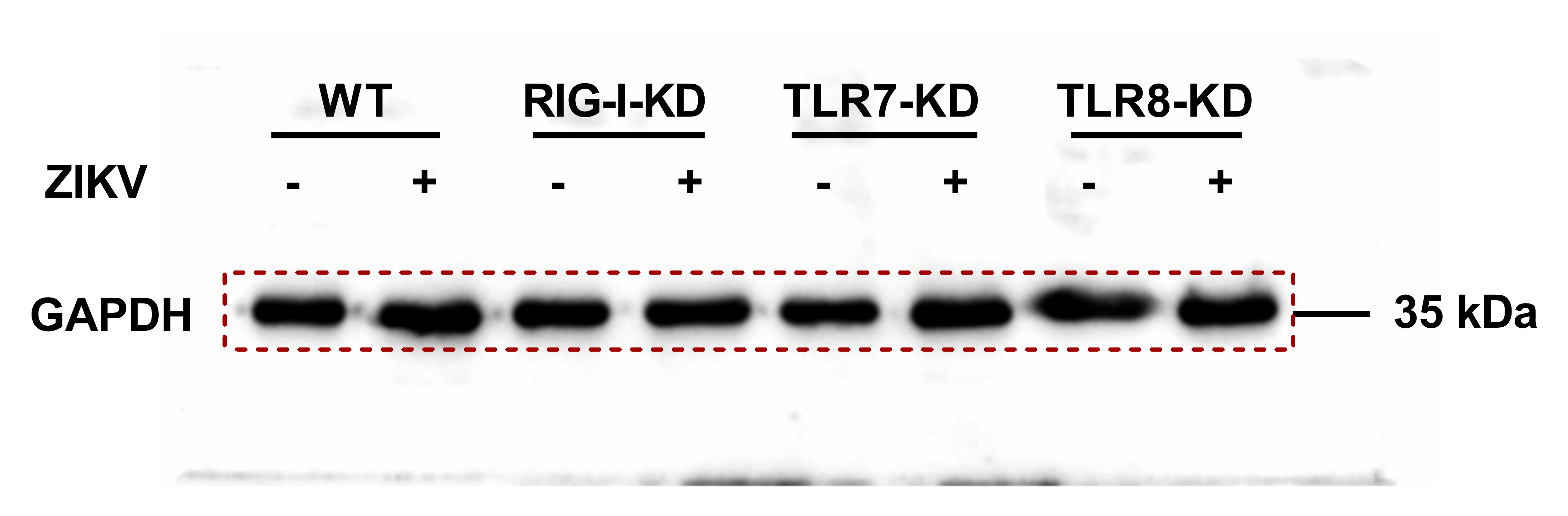

Supplement: Figure 4—source data 1. [file elife-73792-fig4-data1.zip › Figure 4-source data 1/Fig 4F/Figure 4F GAPDH-labeled.tif]

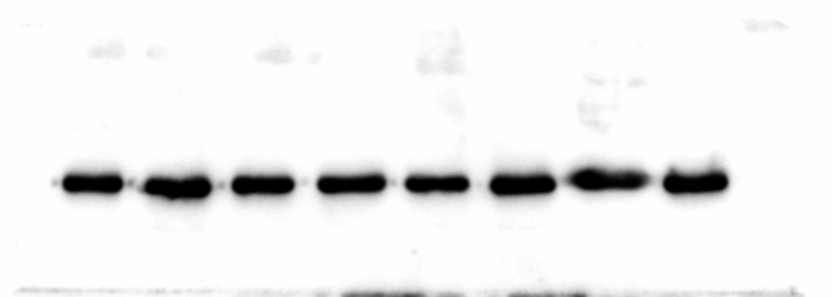

Supplement: Figure 4—source data 1. [file elife-73792-fig4-data1.zip › Figure 4-source data 1/Fig 4F/Figure 4F GAPDH-raw.tif]

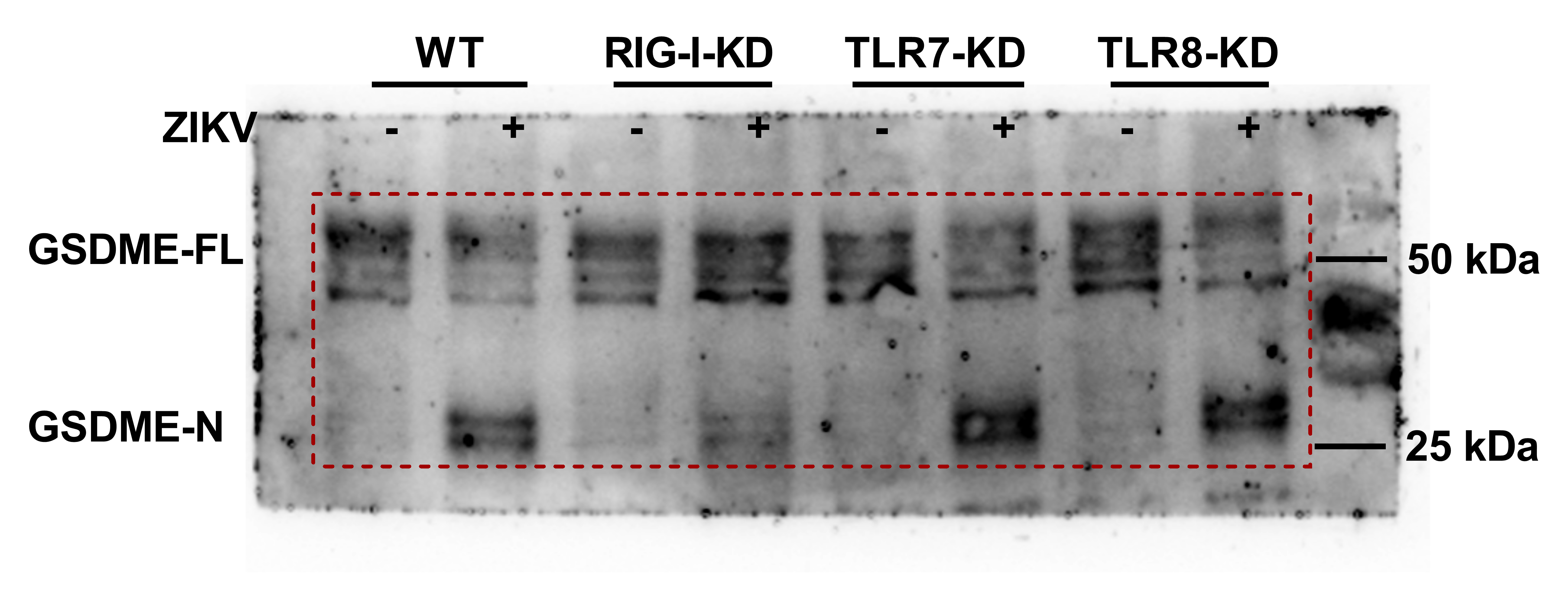

Supplement: Figure 4—source data 1. [file elife-73792-fig4-data1.zip › Figure 4-source data 1/Fig 4F/Figure 4F GSDME-labeled.tif]

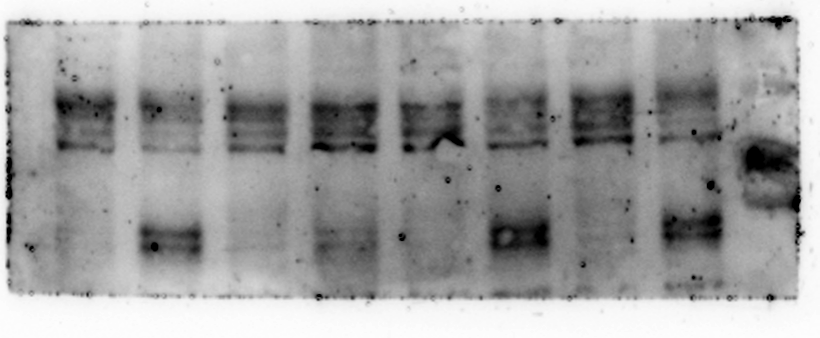

Supplement: Figure 4—source data 1. [file elife-73792-fig4-data1.zip › Figure 4-source data 1/Fig 4F/Figure 4F GSDME-raw.tif]

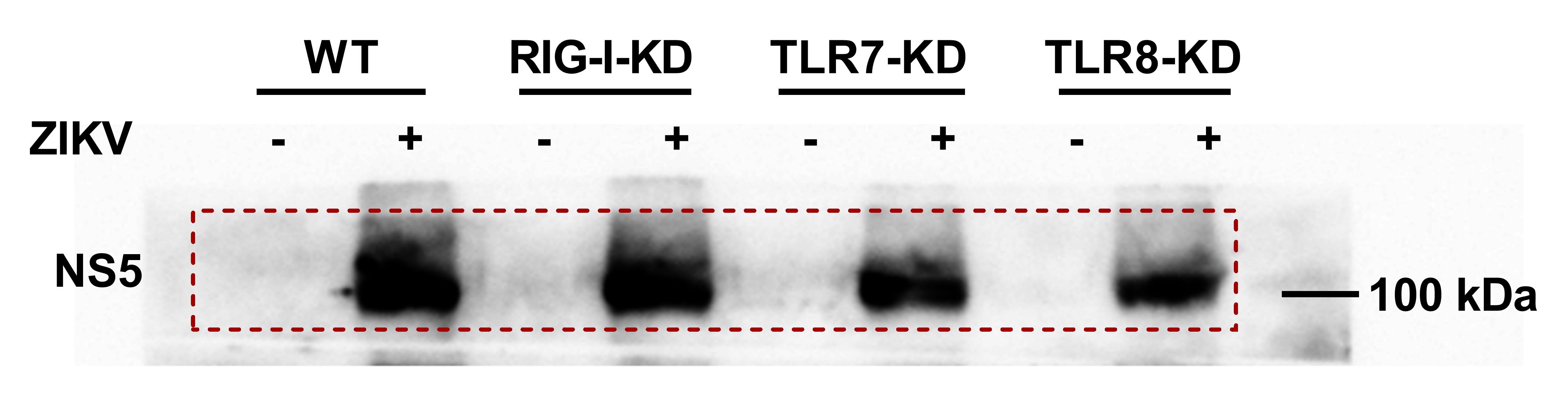

Supplement: Figure 4—source data 1. [file elife-73792-fig4-data1.zip › Figure 4-source data 1/Fig 4F/Figure 4F NS5-labeled.tif]

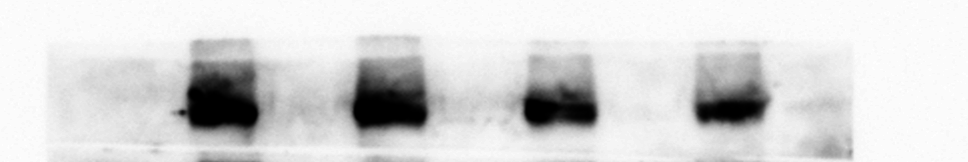

Supplement: Figure 4—source data 1. [file elife-73792-fig4-data1.zip › Figure 4-source data 1/Fig 4F/Figure 4F NS5-raw.tif]

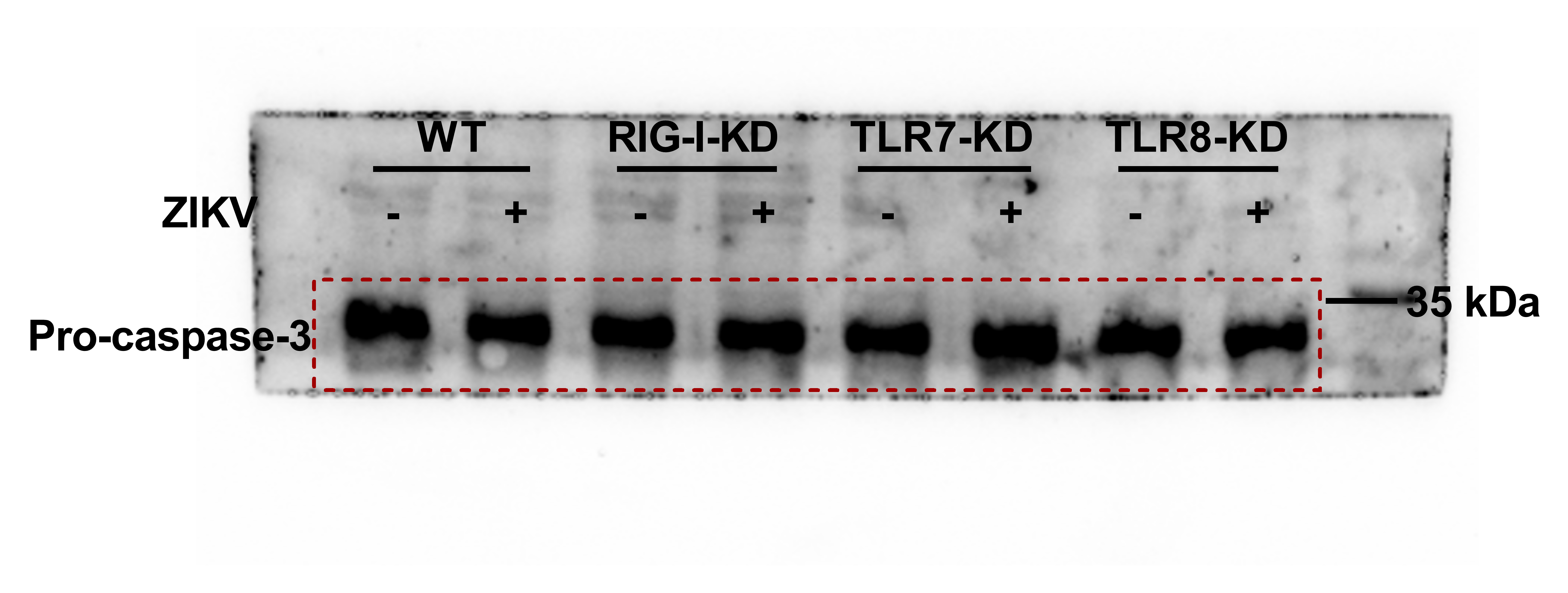

Supplement: Figure 4—source data 1. [file elife-73792-fig4-data1.zip › Figure 4-source data 1/Fig 4F/Figure 4F Pro-caspase-3-labeled.tif]

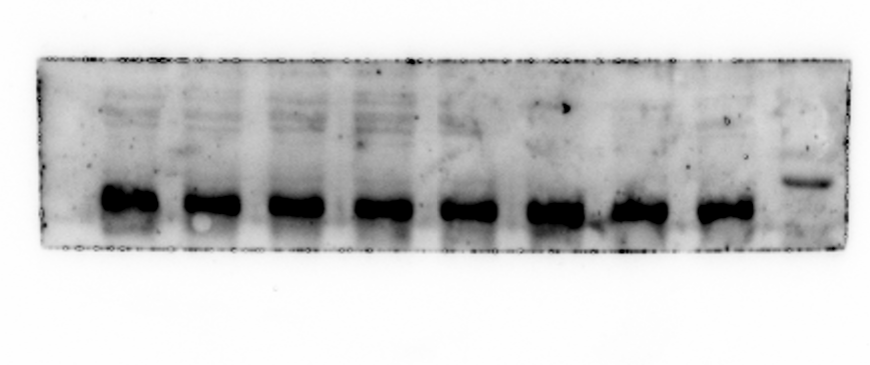

Supplement: Figure 4—source data 1. [file elife-73792-fig4-data1.zip › Figure 4-source data 1/Fig 4F/Figure 4F Pro-caspase-3-raw.tif]

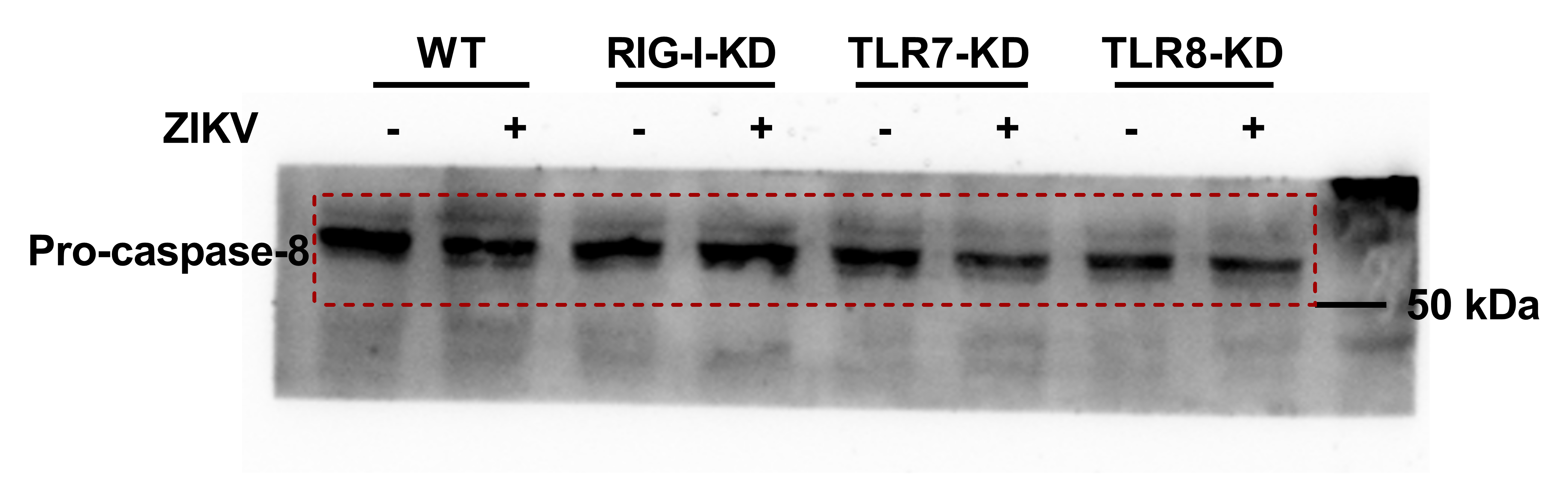

Supplement: Figure 4—source data 1. [file elife-73792-fig4-data1.zip › Figure 4-source data 1/Fig 4F/Figure 4F Pro-caspase-8-labeled.tif]

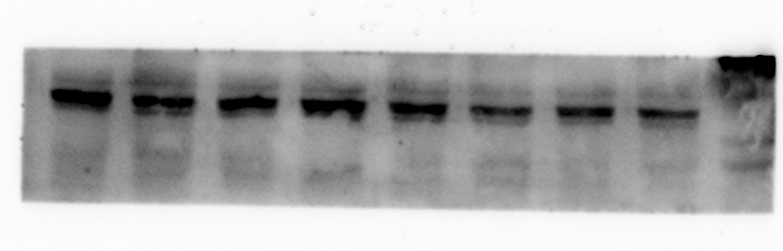

Supplement: Figure 4—source data 1. [file elife-73792-fig4-data1.zip › Figure 4-source data 1/Fig 4F/Figure 4F Pro-caspase-8-raw.tif]

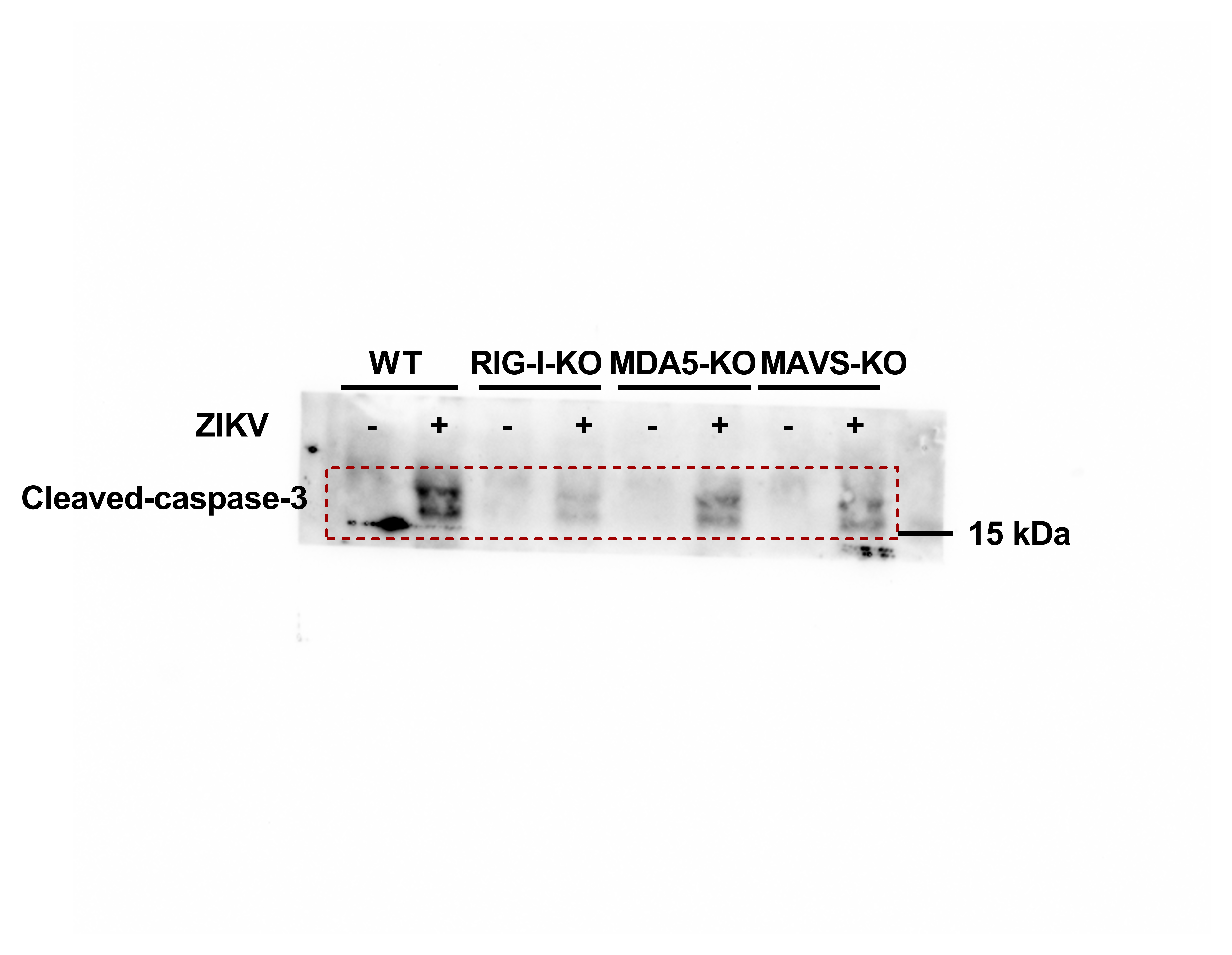

Supplement: Figure 4—source data 1. [file elife-73792-fig4-data1.zip › Figure 4-source data 1/Fig 4H/Figure 4H Cleaved-caspase-3-labeled.tif]

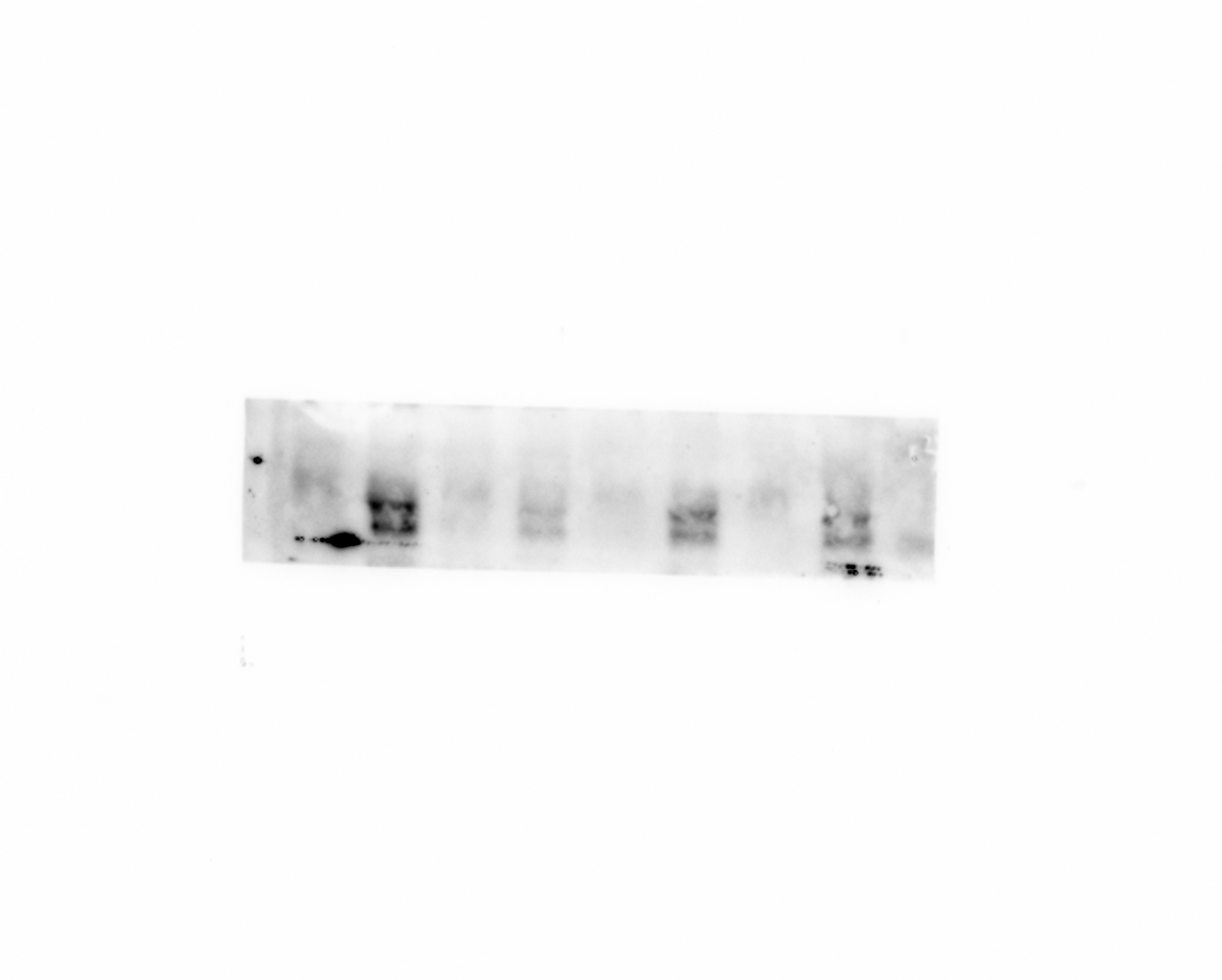

Supplement: Figure 4—source data 1. [file elife-73792-fig4-data1.zip › Figure 4-source data 1/Fig 4H/Figure 4H Cleaved-caspase-3-raw.Tif]

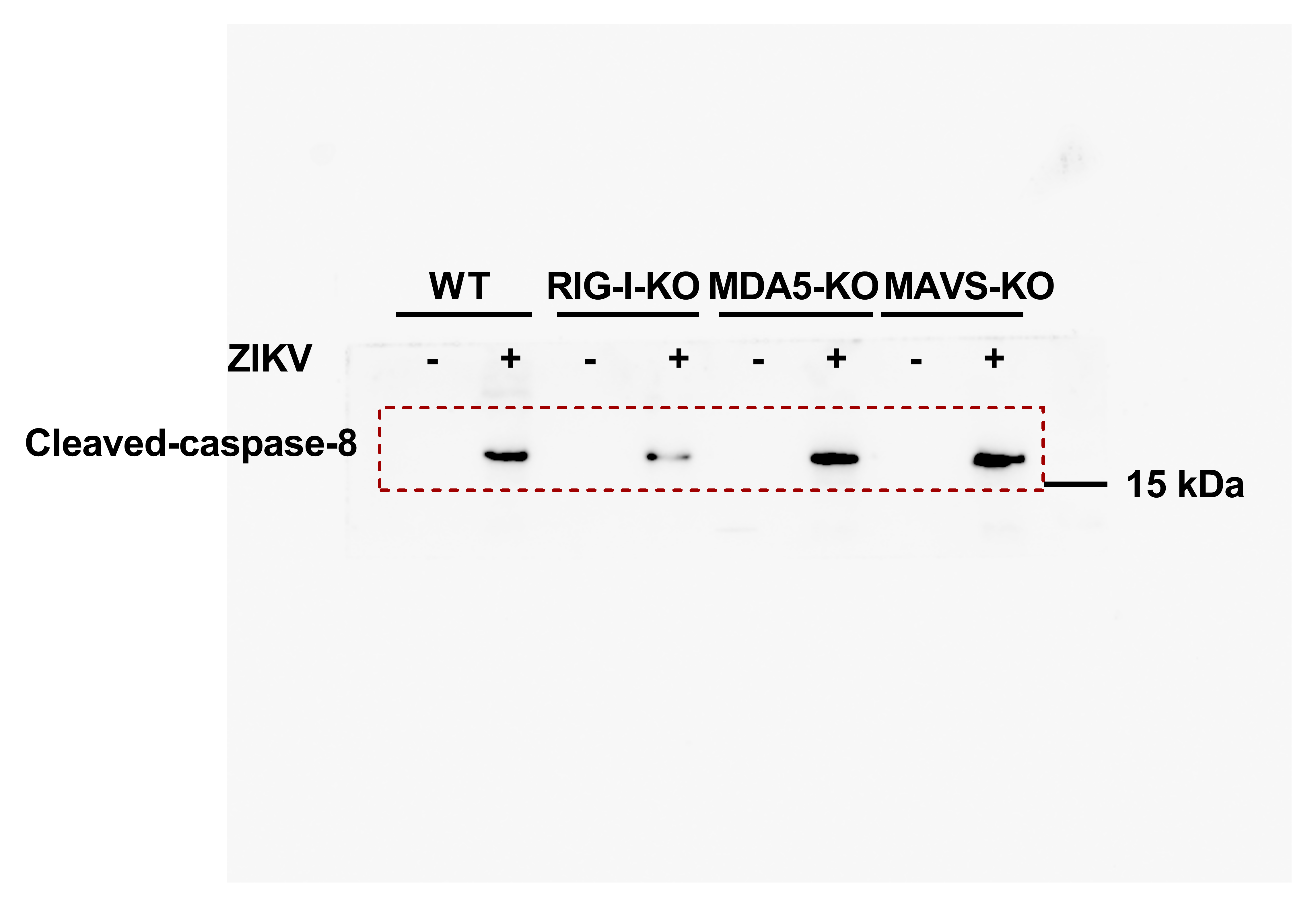

Supplement: Figure 4—source data 1. [file elife-73792-fig4-data1.zip › Figure 4-source data 1/Fig 4H/Figure 4H Cleaved-caspase-8-label.tif]

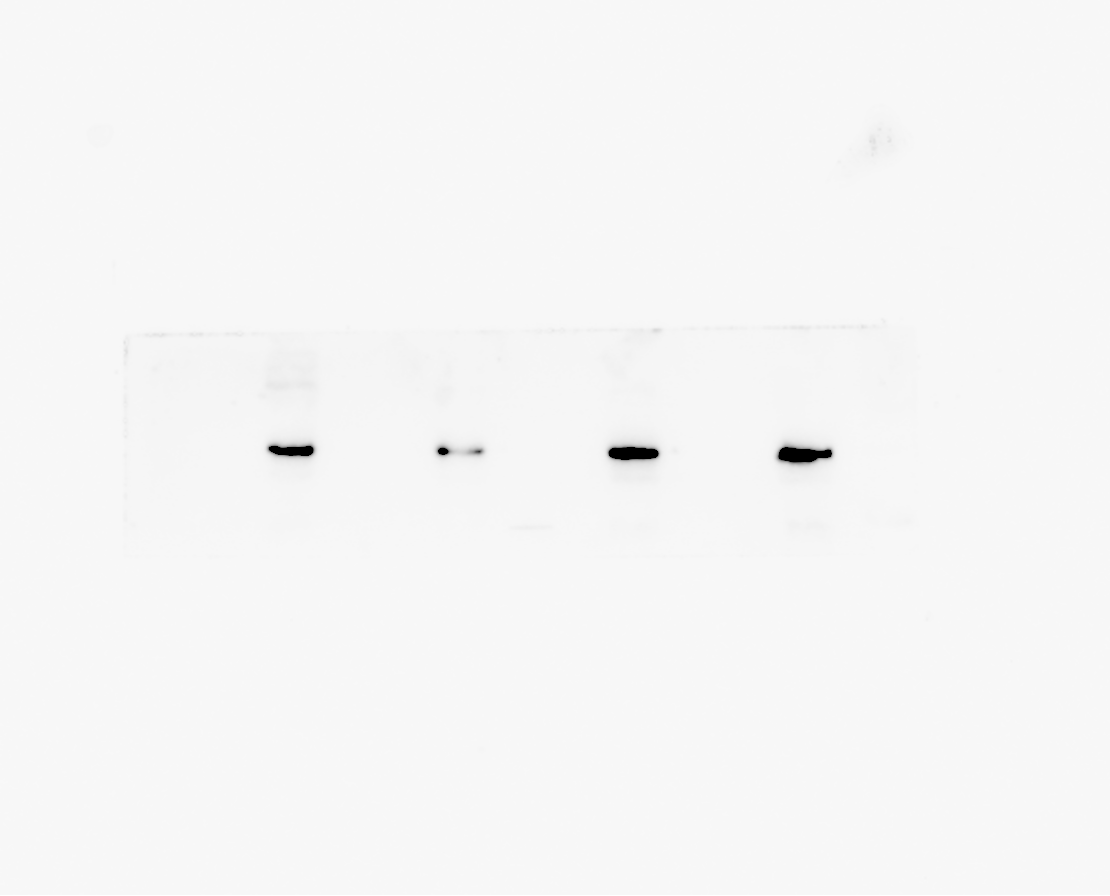

Supplement: Figure 4—source data 1. [file elife-73792-fig4-data1.zip › Figure 4-source data 1/Fig 4H/Figure 4H Cleaved-caspase-8-raw.tif.tif]

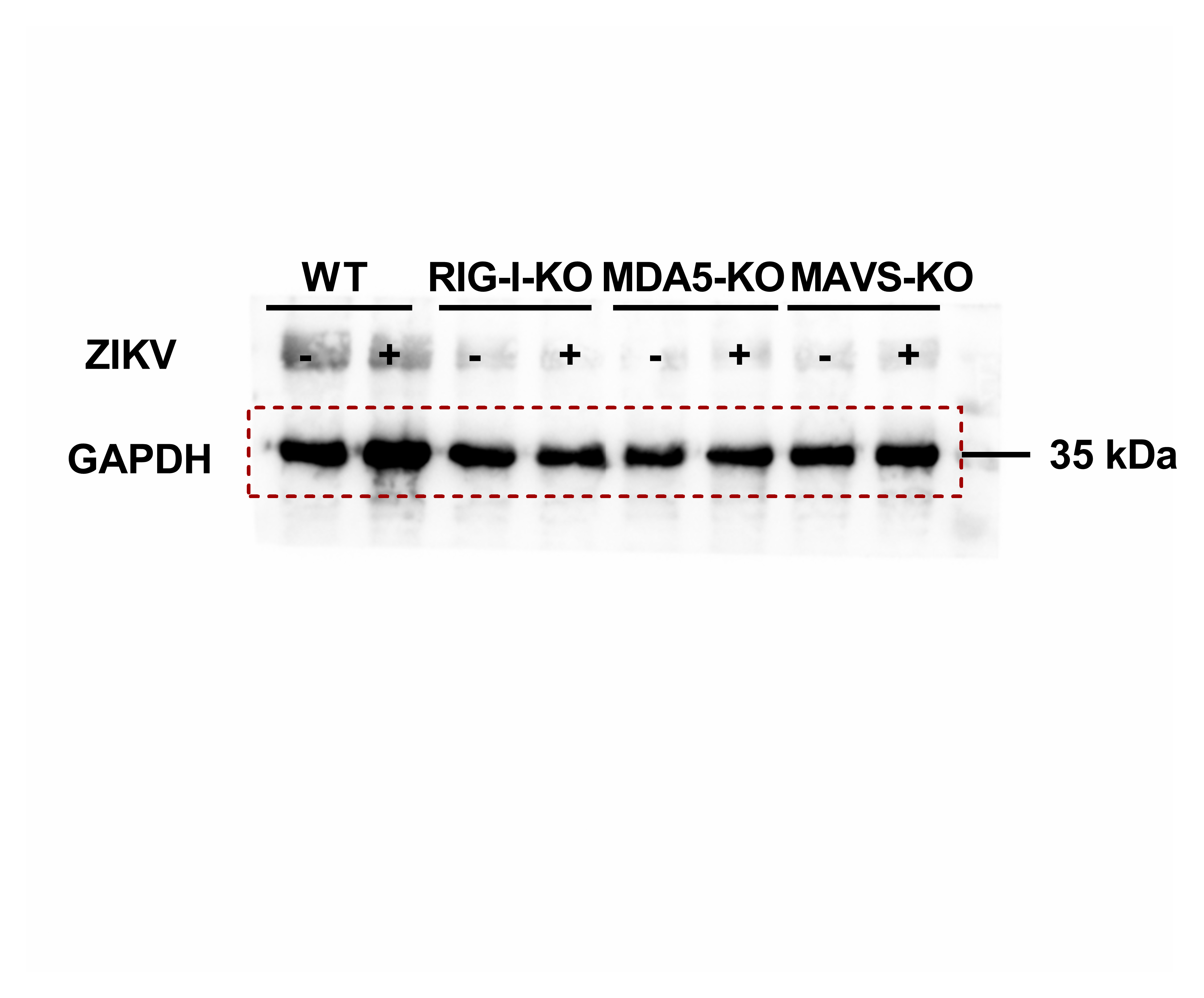

Supplement: Figure 4—source data 1. [file elife-73792-fig4-data1.zip › Figure 4-source data 1/Fig 4H/Figure 4H GAPDH-labeled.tif]

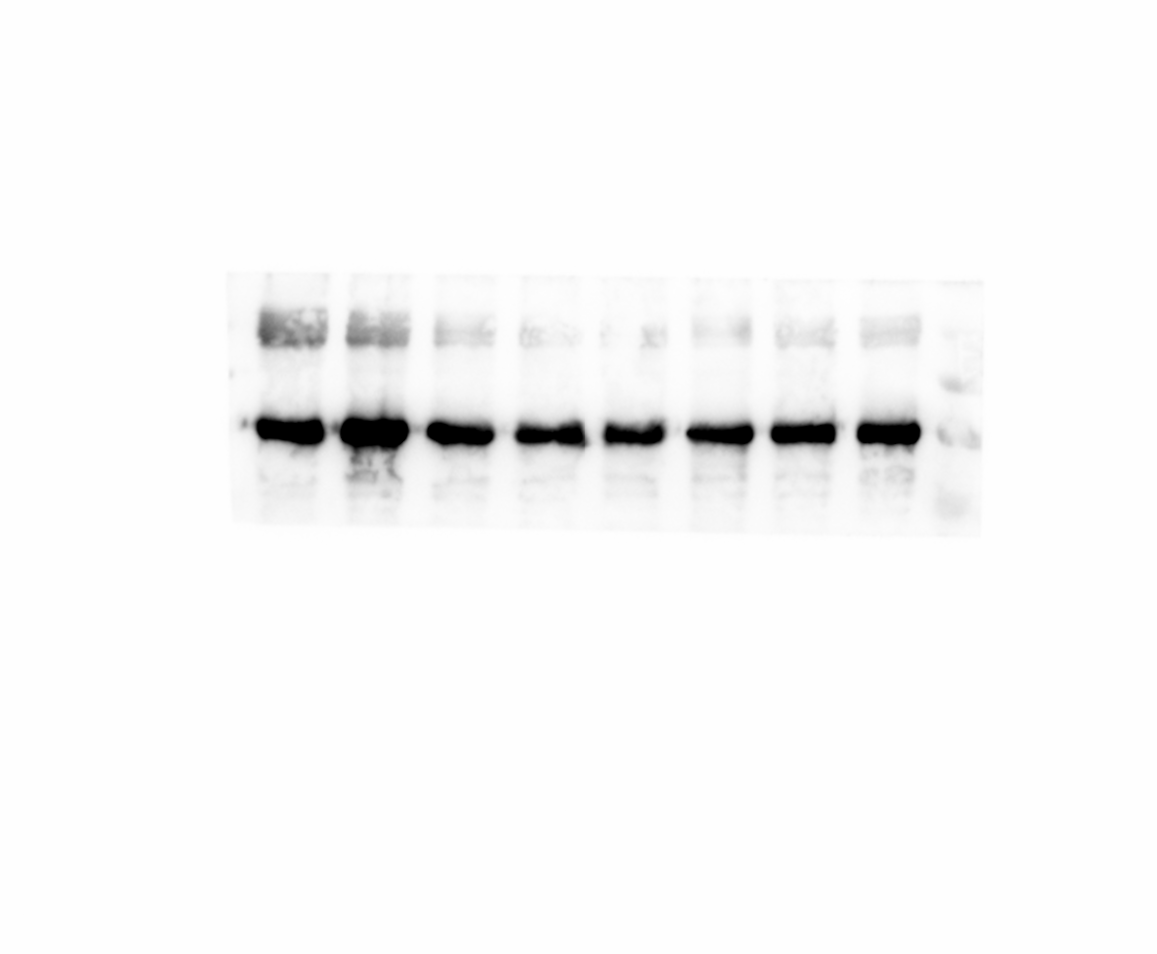

Supplement: Figure 4—source data 1. [file elife-73792-fig4-data1.zip › Figure 4-source data 1/Fig 4H/Figure 4H GAPDH-raw.tif]

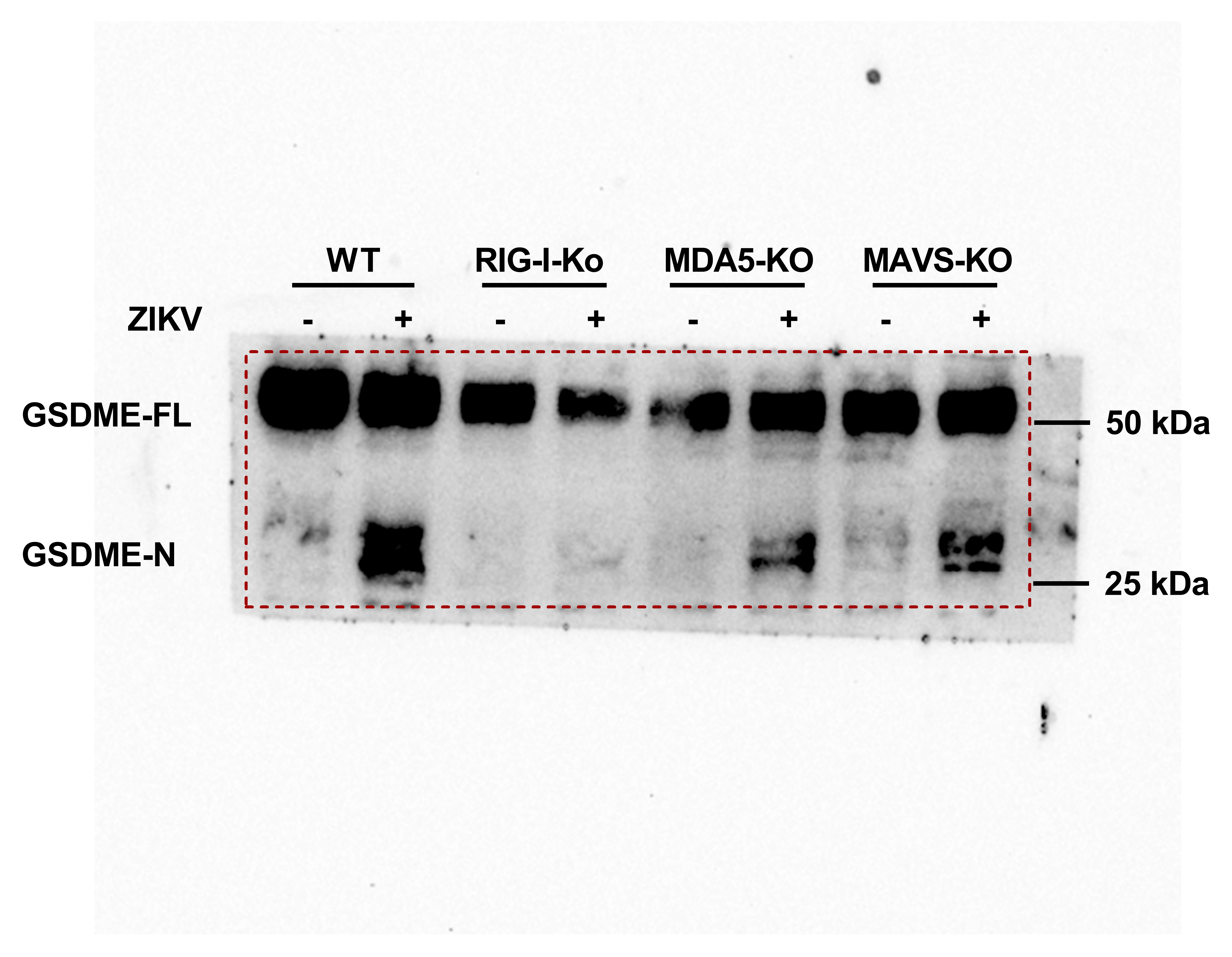

Supplement: Figure 4—source data 1. [file elife-73792-fig4-data1.zip › Figure 4-source data 1/Fig 4H/Figure 4H GSDME-labeled.tif]

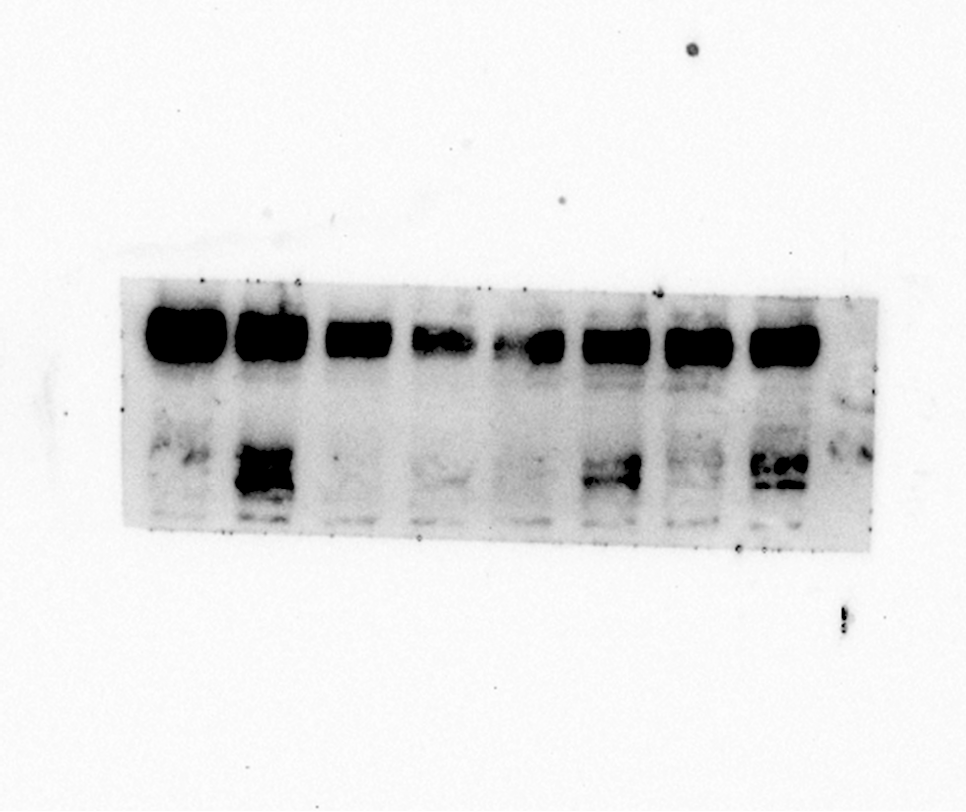

Supplement: Figure 4—source data 1. [file elife-73792-fig4-data1.zip › Figure 4-source data 1/Fig 4H/Figure 4H GSDME-raw.tif]

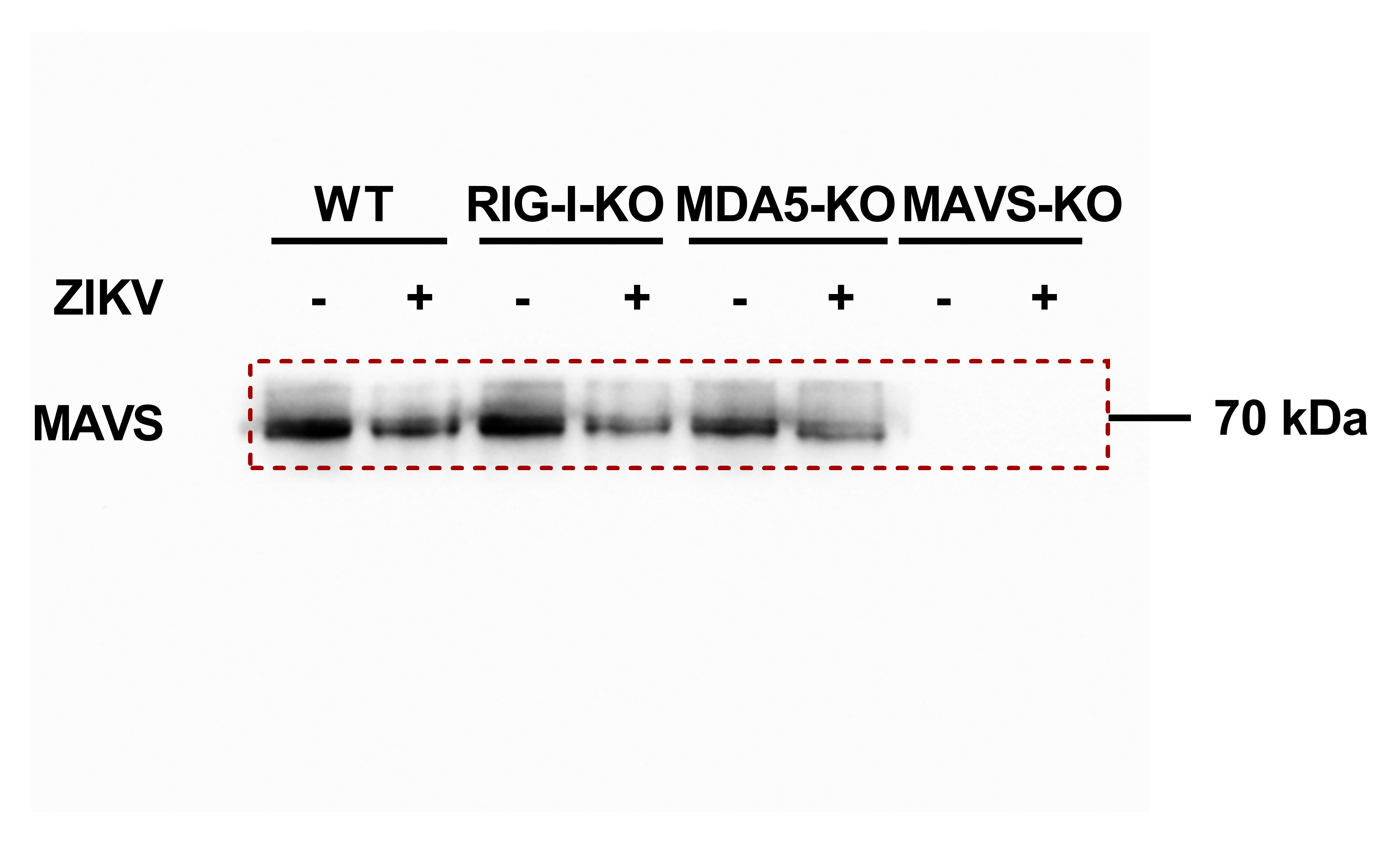

Supplement: Figure 4—source data 1. [file elife-73792-fig4-data1.zip › Figure 4-source data 1/Fig 4H/Figure 4H MAVS-labeled.tif]

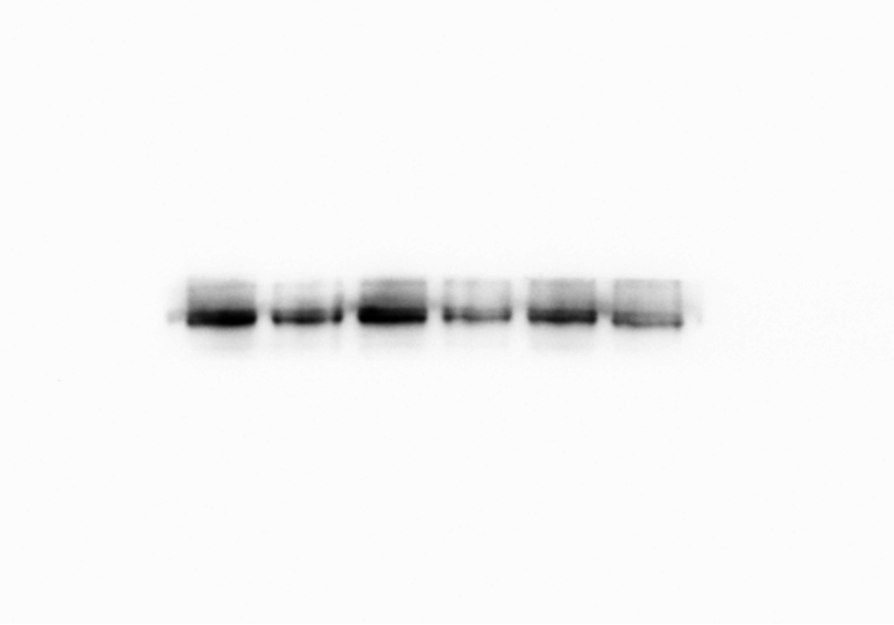

Supplement: Figure 4—source data 1. [file elife-73792-fig4-data1.zip › Figure 4-source data 1/Fig 4H/Figure 4H MAVS-raw.tif]

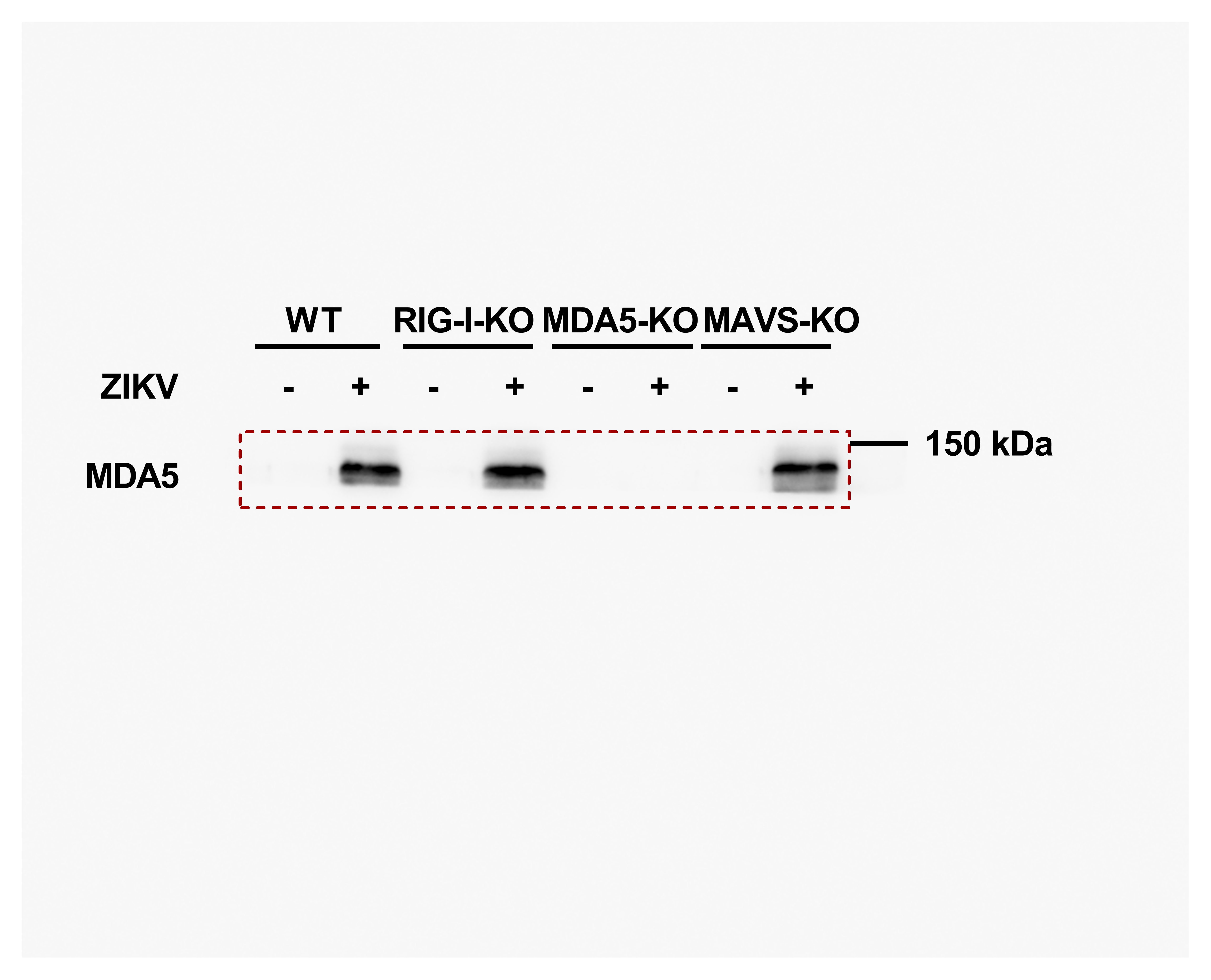

Supplement: Figure 4—source data 1. [file elife-73792-fig4-data1.zip › Figure 4-source data 1/Fig 4H/Figure 4H MDA5-labeled.tif]

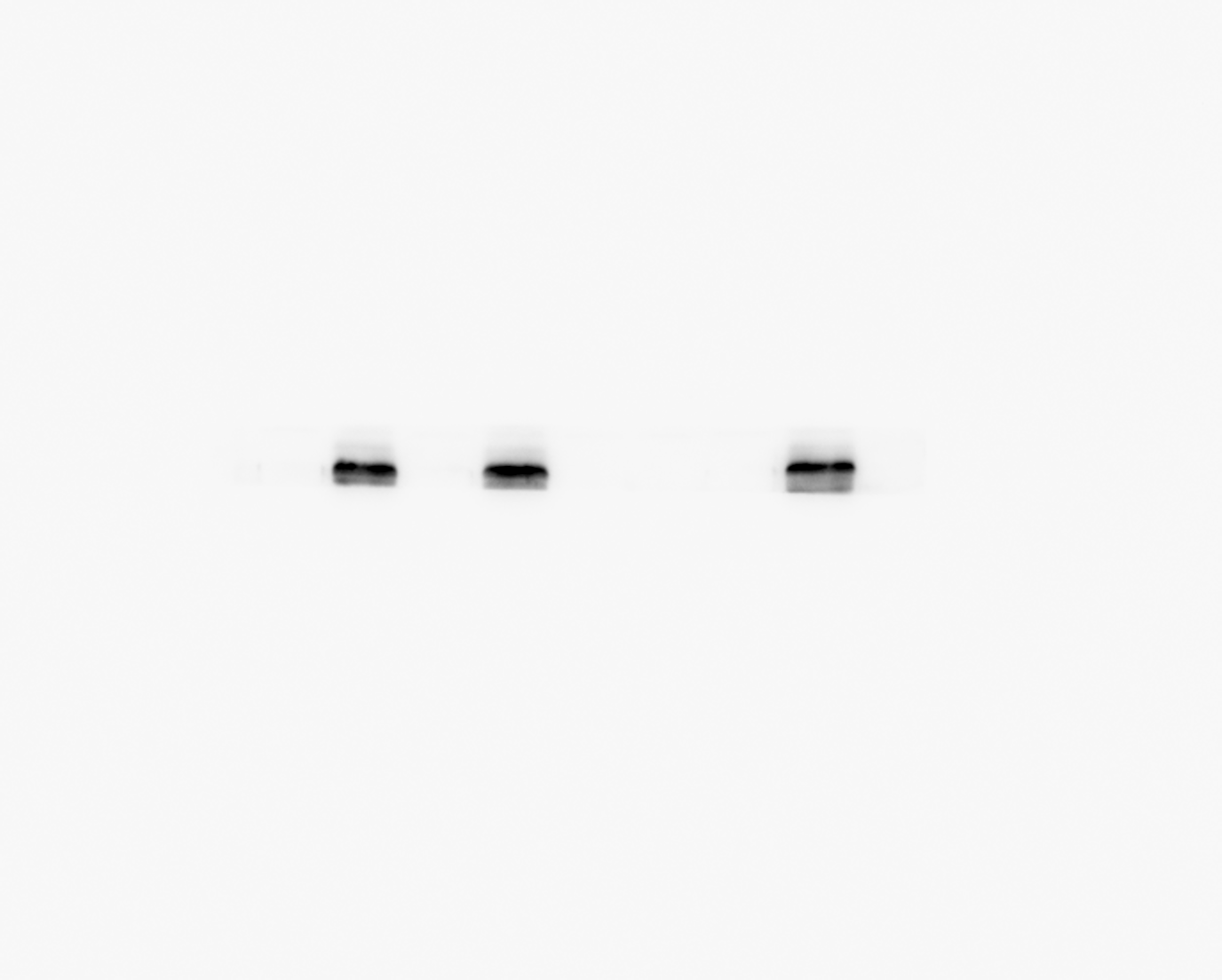

Supplement: Figure 4—source data 1. [file elife-73792-fig4-data1.zip › Figure 4-source data 1/Fig 4H/Figure 4H MDA5-raw.Tif]

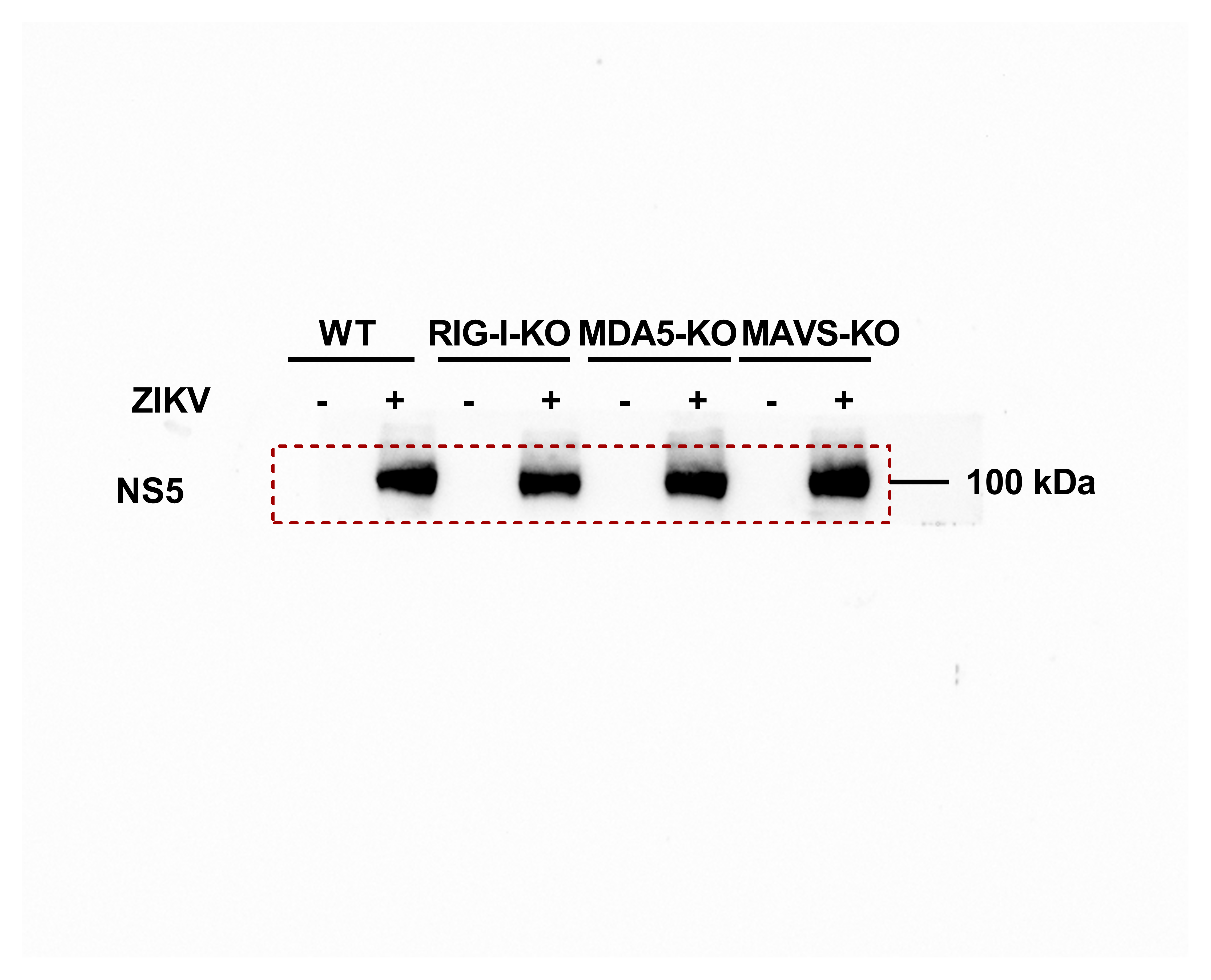

Supplement: Figure 4—source data 1. [file elife-73792-fig4-data1.zip › Figure 4-source data 1/Fig 4H/Figure 4H NS5-labeled.tif]

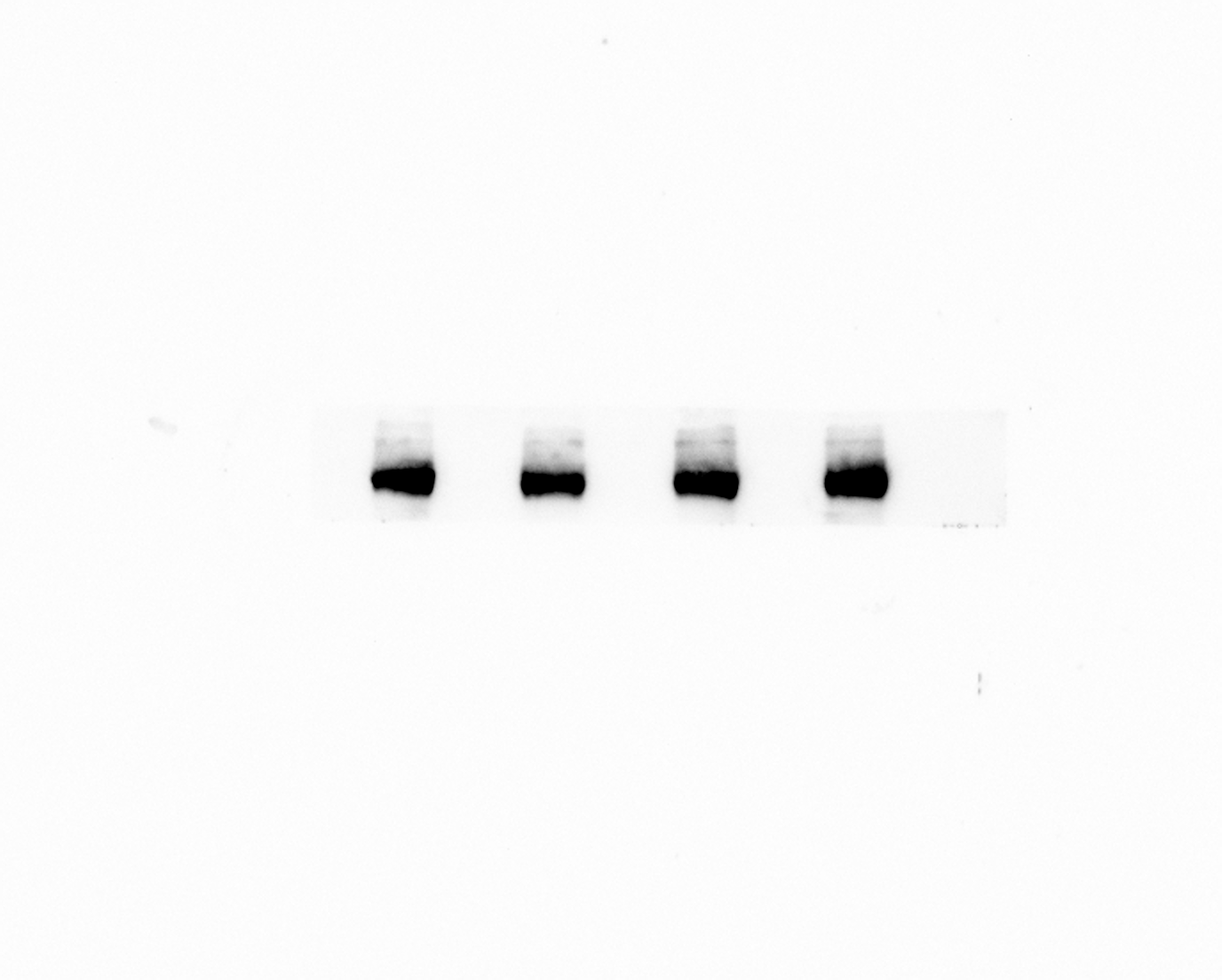

Supplement: Figure 4—source data 1. [file elife-73792-fig4-data1.zip › Figure 4-source data 1/Fig 4H/Figure 4H NS5-raw.Tif]

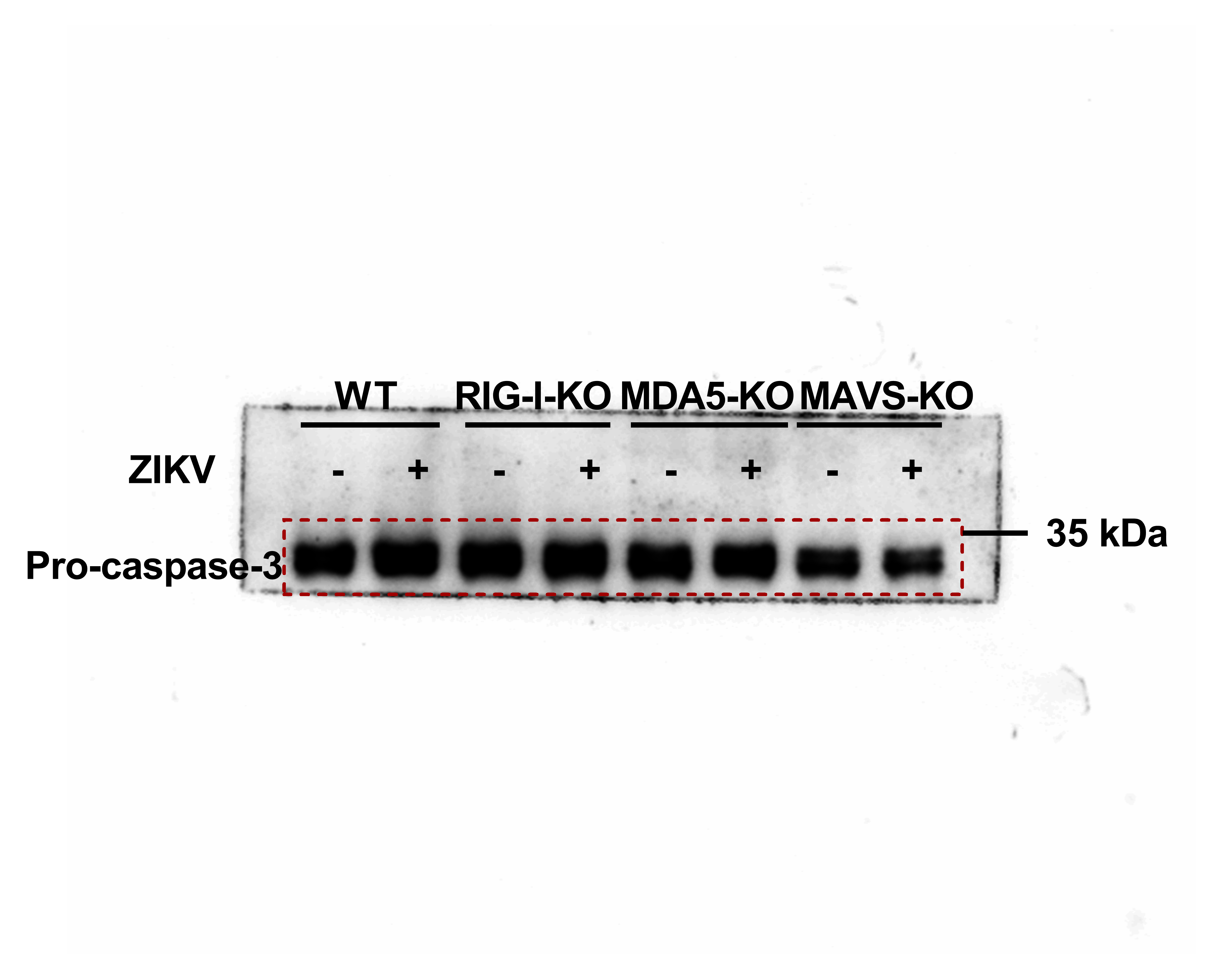

Supplement: Figure 4—source data 1. [file elife-73792-fig4-data1.zip › Figure 4-source data 1/Fig 4H/Figure 4H Pro-caspase-3-labeled.tif]

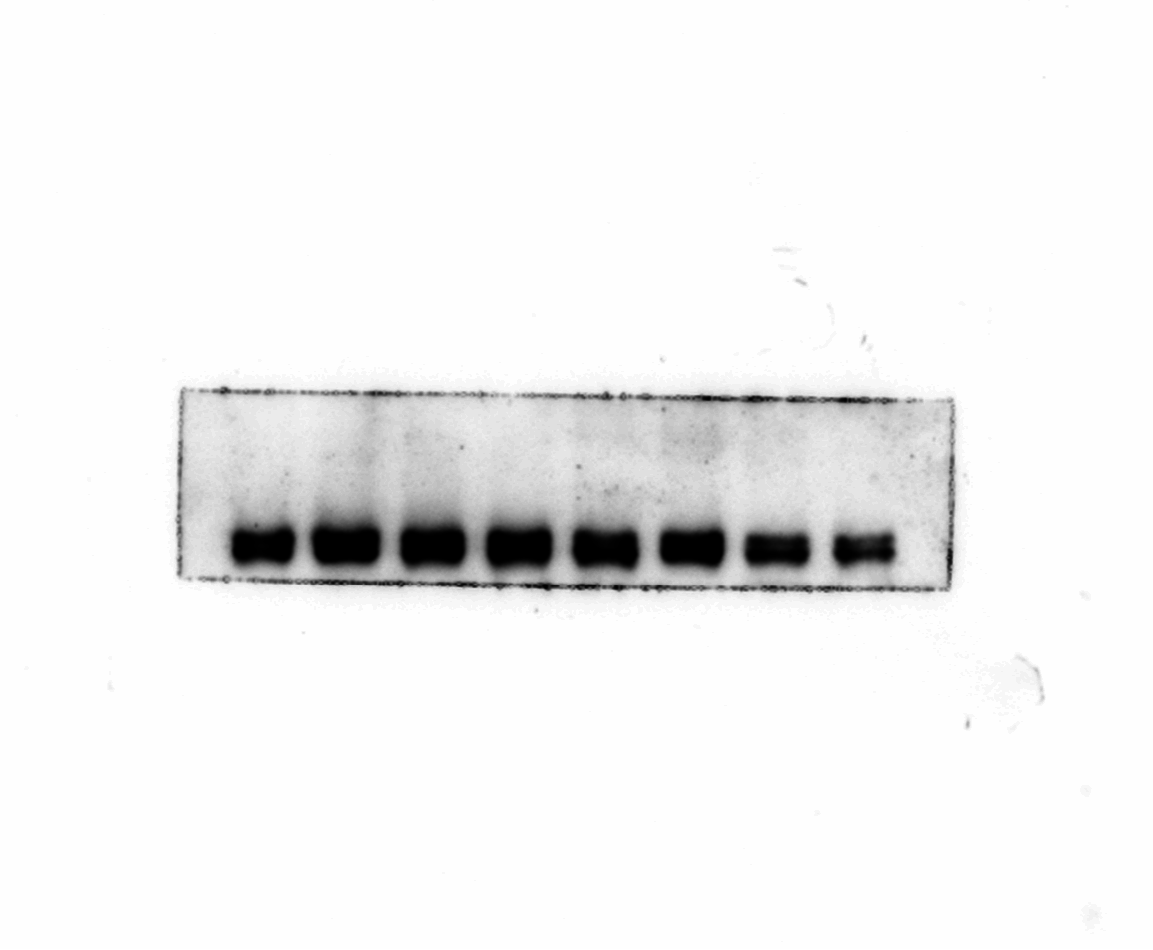

Supplement: Figure 4—source data 1. [file elife-73792-fig4-data1.zip › Figure 4-source data 1/Fig 4H/Figure 4H Pro-caspase-3-raw.tif]

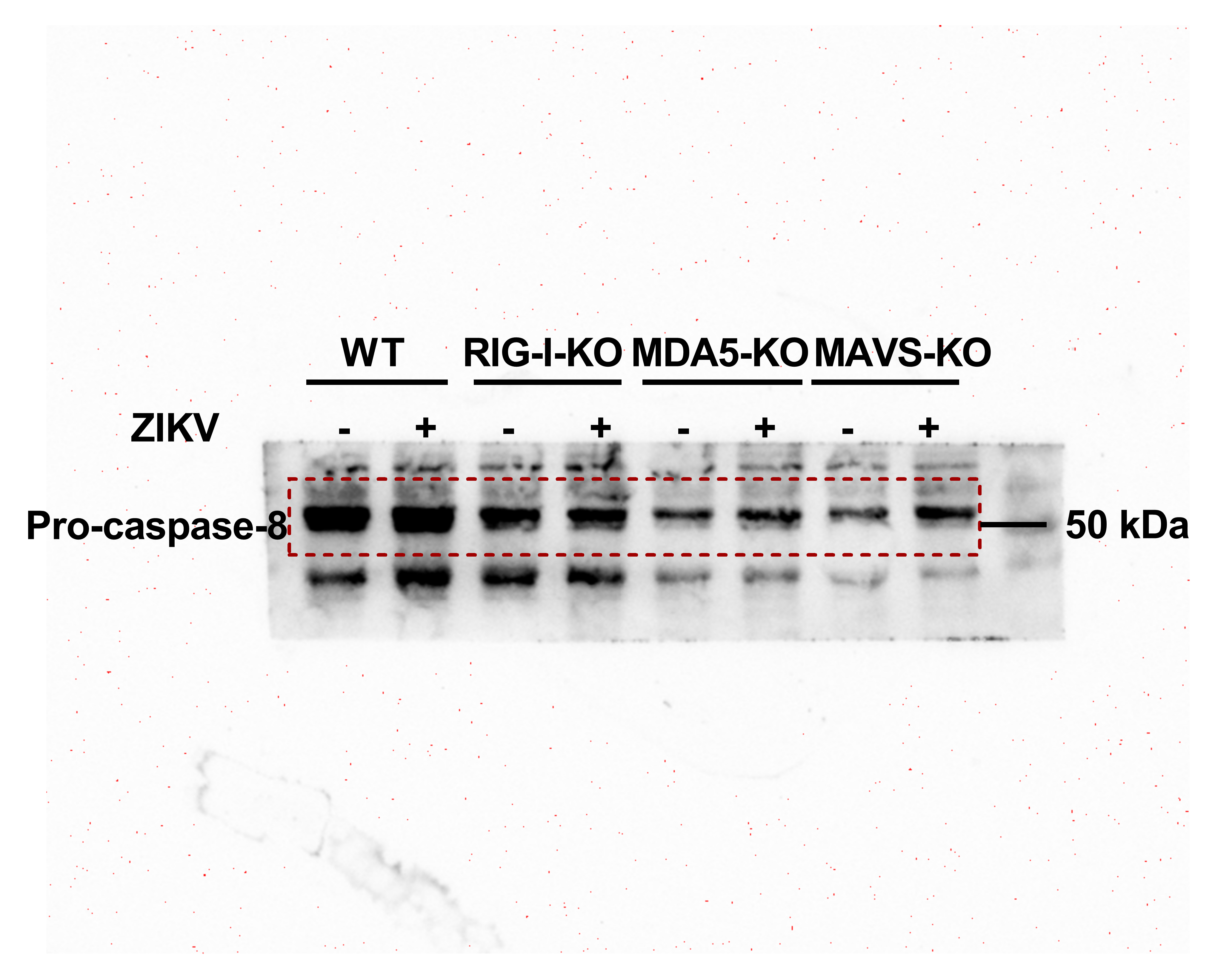

Supplement: Figure 4—source data 1. [file elife-73792-fig4-data1.zip › Figure 4-source data 1/Fig 4H/Figure 4H Pro-caspase-8-labeled.tif]

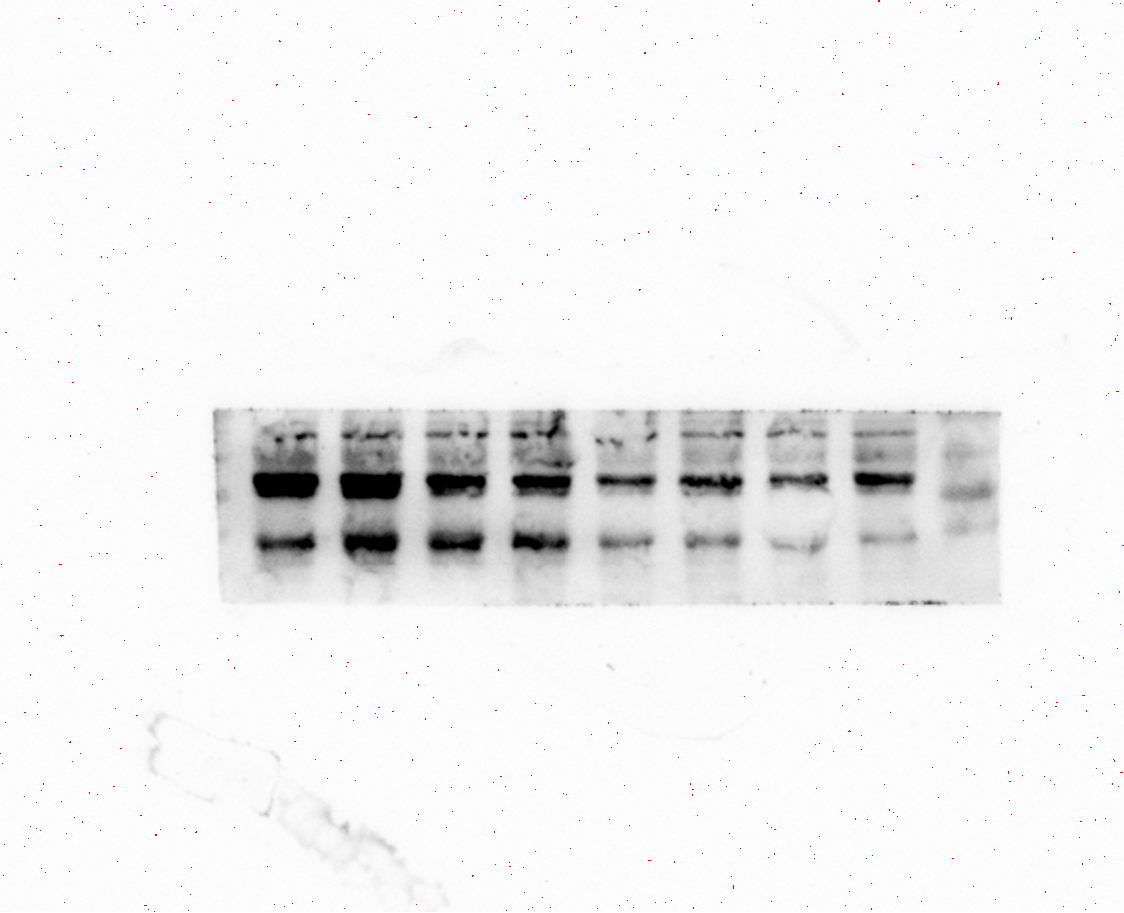

Supplement: Figure 4—source data 1. [file elife-73792-fig4-data1.zip › Figure 4-source data 1/Fig 4H/Figure 4H Pro-caspase-8-raw.tif]

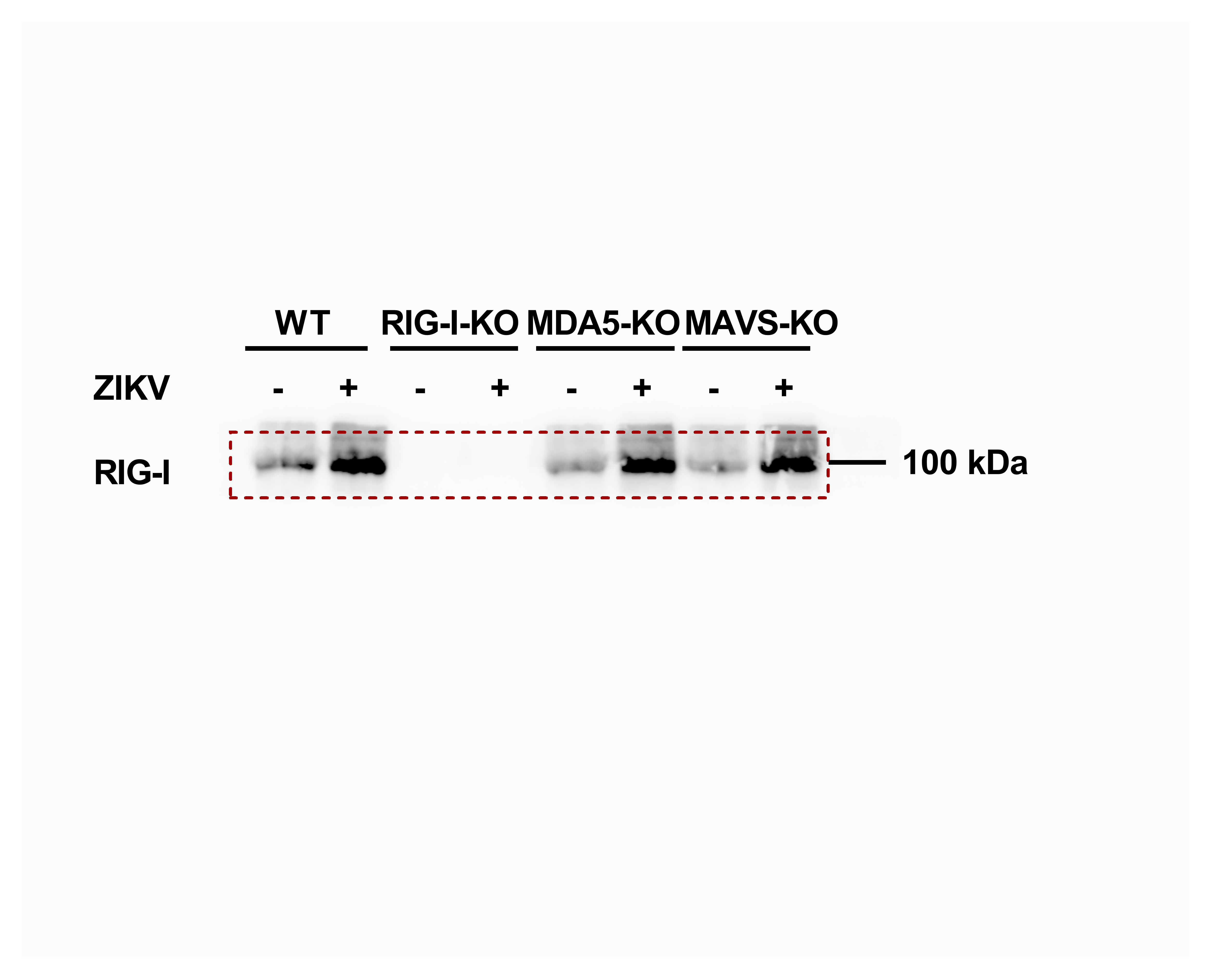

Supplement: Figure 4—source data 1. [file elife-73792-fig4-data1.zip › Figure 4-source data 1/Fig 4H/Figure 4H RIG-I-labeled.tif]

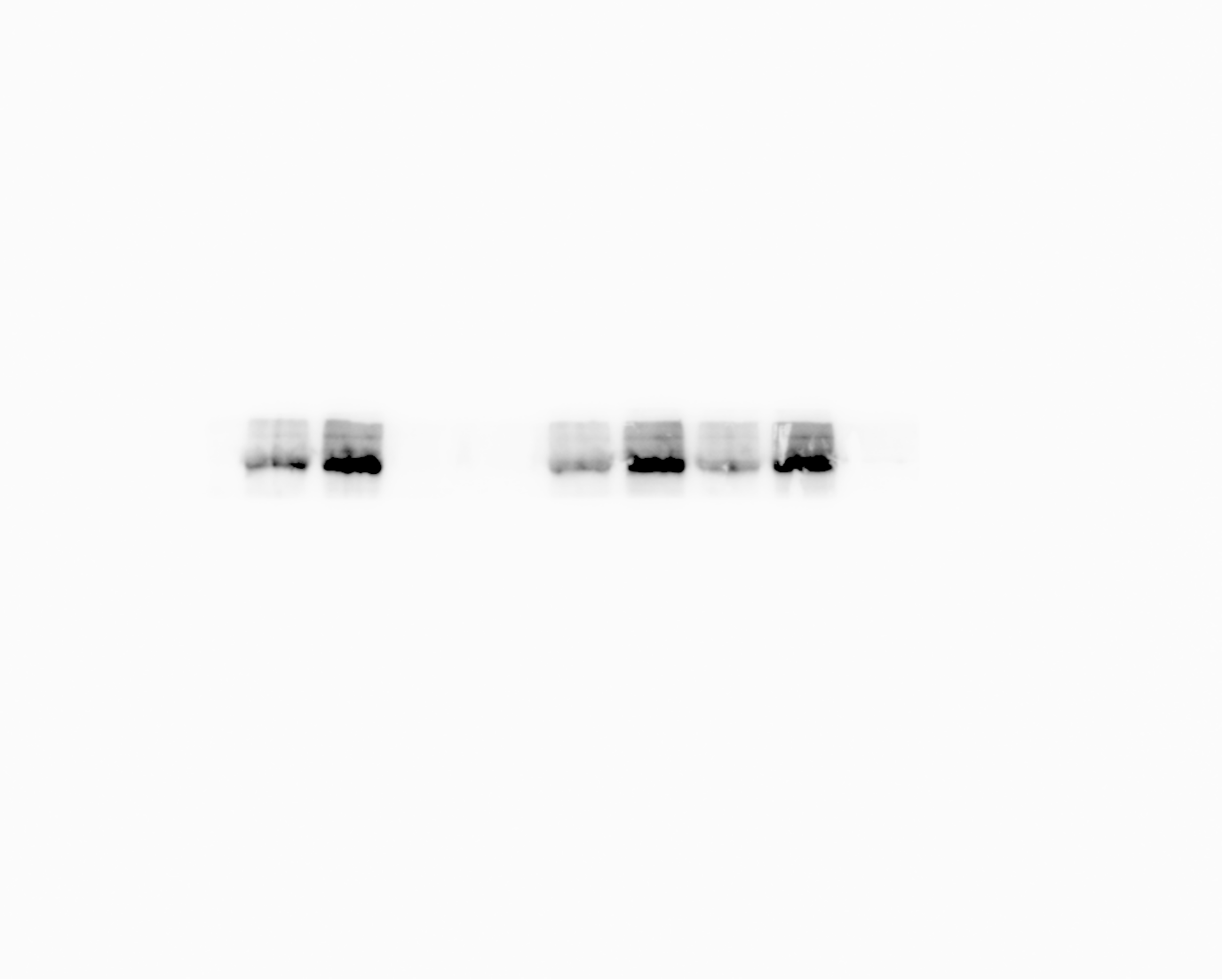

Supplement: Figure 4—source data 1. [file elife-73792-fig4-data1.zip › Figure 4-source data 1/Fig 4H/Figure 4H RIG-I-raw.Tif]

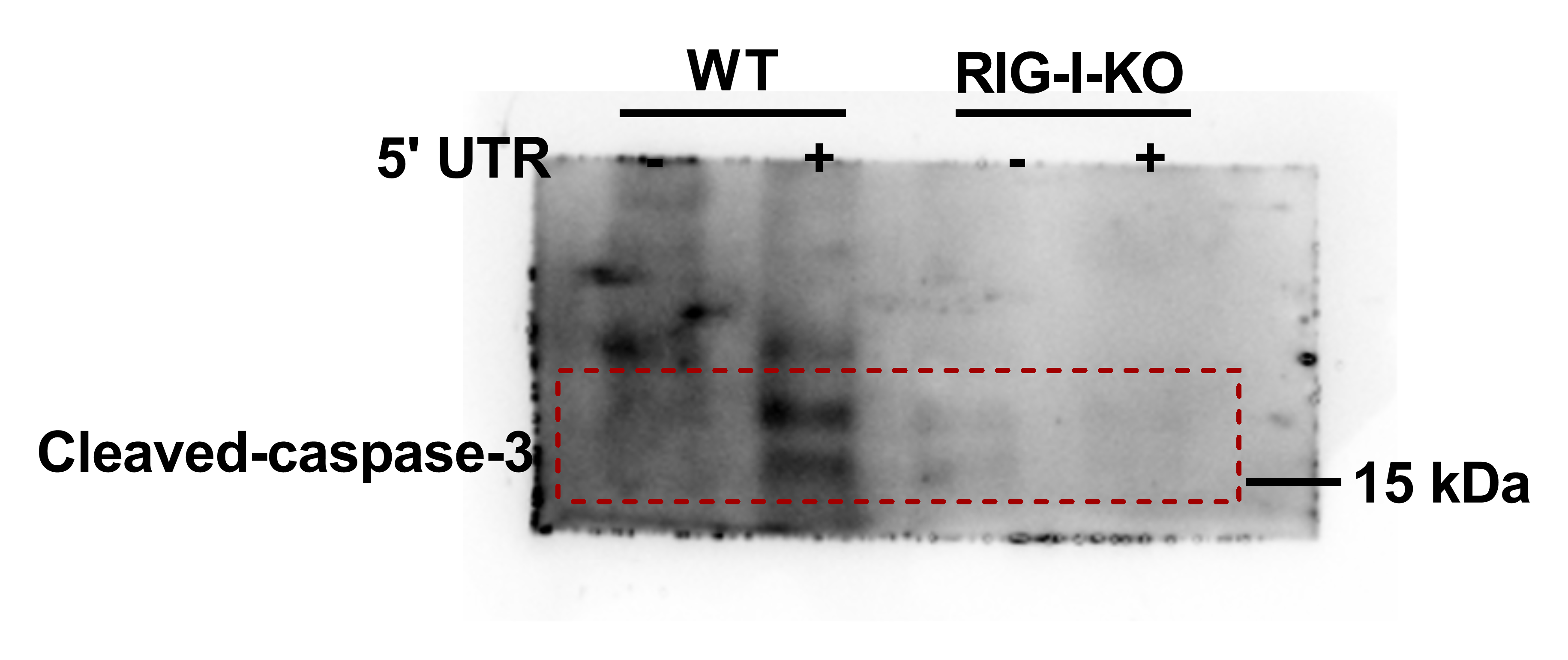

Supplement: Figure 4—source data 1. [file elife-73792-fig4-data1.zip › Figure 4-source data 1/Fig 4J/Figure 4J Cleaved-caspase-3-labeled.tif]

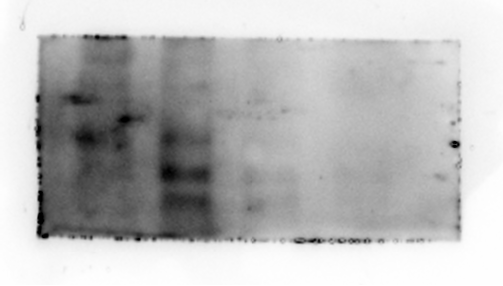

Supplement: Figure 4—source data 1. [file elife-73792-fig4-data1.zip › Figure 4-source data 1/Fig 4J/Figure 4J Cleaved-caspase-3-raw.tif]

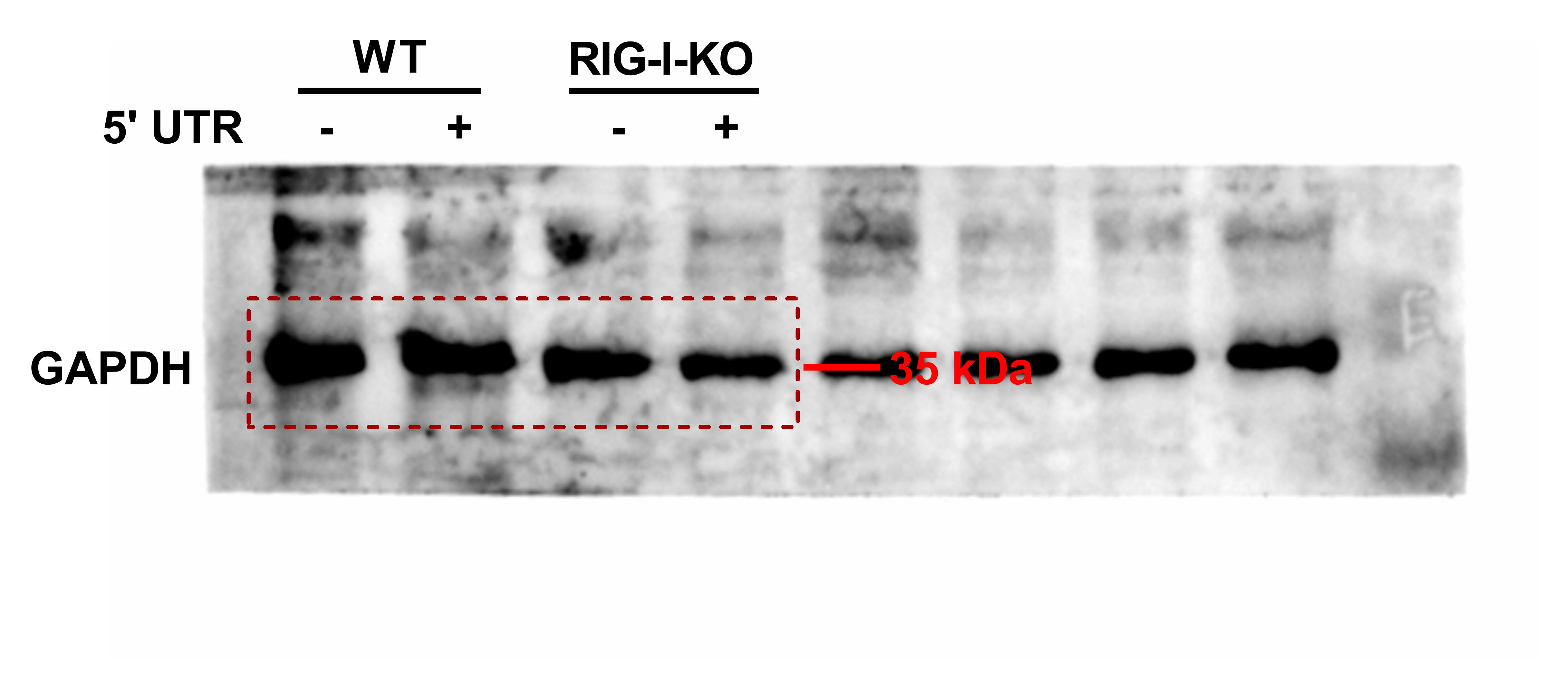

Supplement: Figure 4—source data 1. [file elife-73792-fig4-data1.zip › Figure 4-source data 1/Fig 4J/Figure 4J GAPDH-labeled.tif]

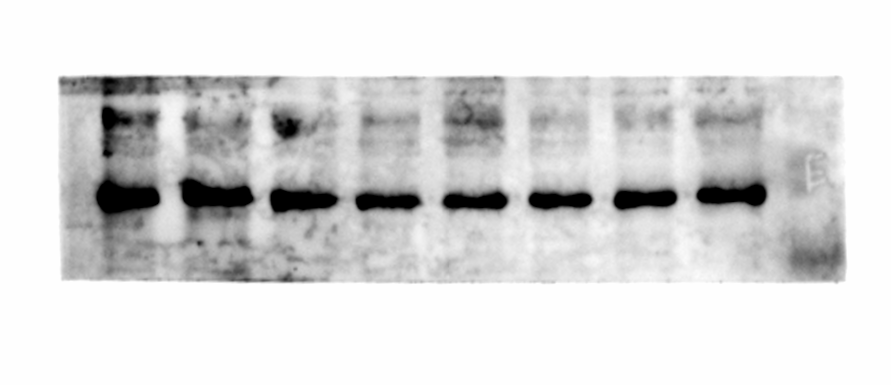

Supplement: Figure 4—source data 1. [file elife-73792-fig4-data1.zip › Figure 4-source data 1/Fig 4J/Figure 4J GAPDH-raw.tif]

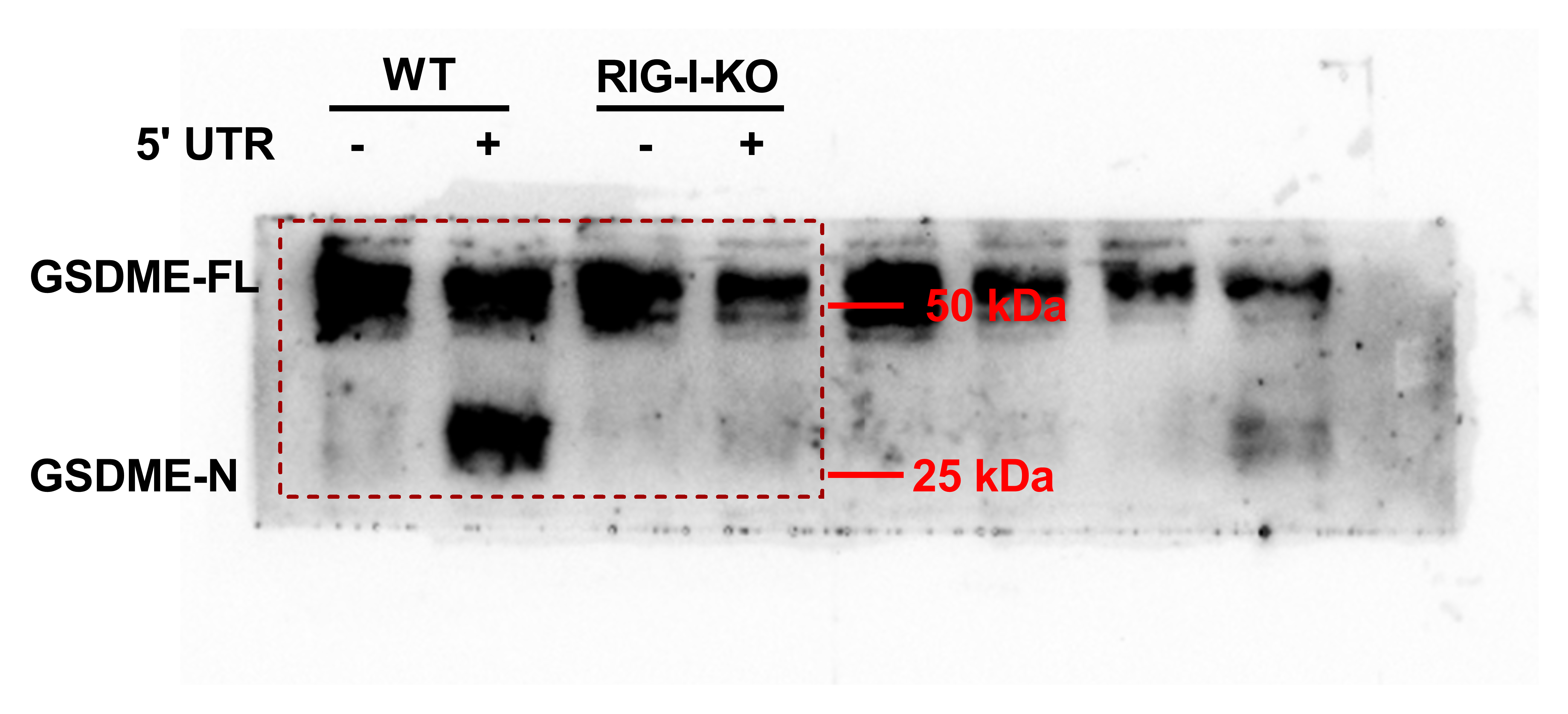

Supplement: Figure 4—source data 1. [file elife-73792-fig4-data1.zip › Figure 4-source data 1/Fig 4J/Figure 4J GSDME-labeled.tif]

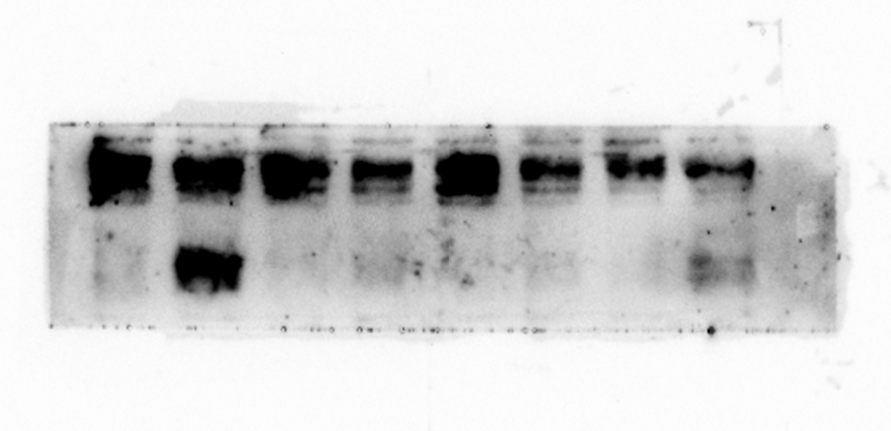

Supplement: Figure 4—source data 1. [file elife-73792-fig4-data1.zip › Figure 4-source data 1/Fig 4J/Figure 4J GSDME-raw.tif]

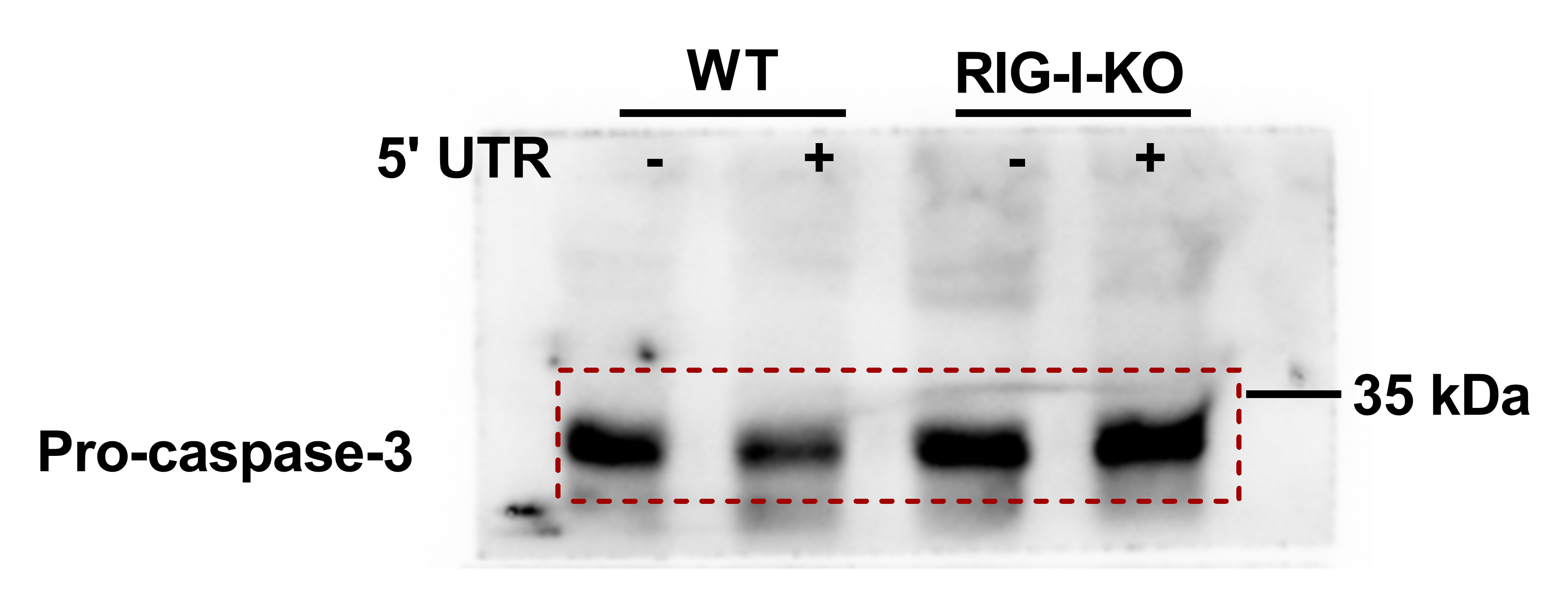

Supplement: Figure 4—source data 1. [file elife-73792-fig4-data1.zip › Figure 4-source data 1/Fig 4J/Figure 4J Pro-caspase-3-labeled.tif]

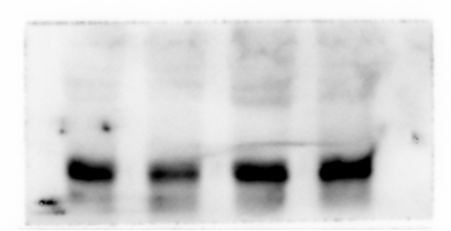

Supplement: Figure 4—source data 1. [file elife-73792-fig4-data1.zip › Figure 4-source data 1/Fig 4J/Figure 4J Pro-caspase-3-raw.tif]

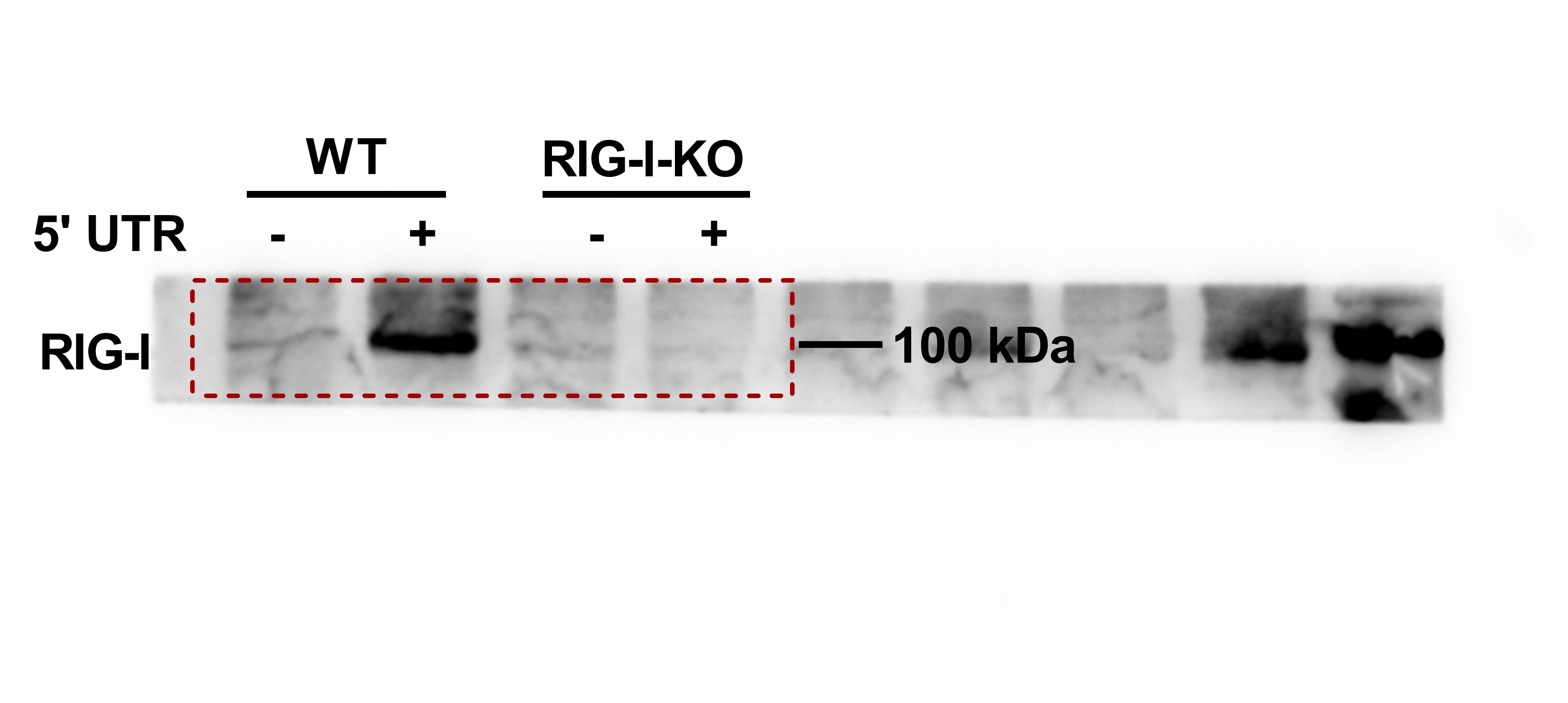

Supplement: Figure 4—source data 1. [file elife-73792-fig4-data1.zip › Figure 4-source data 1/Fig 4J/Figure 4J RIG-I-labeled.tif]

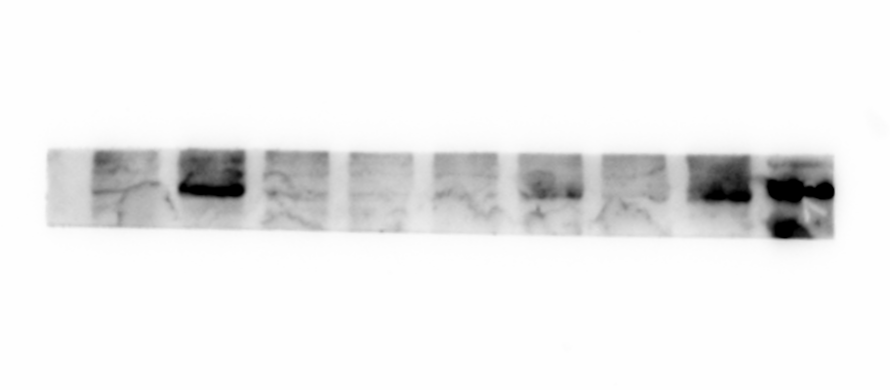

Supplement: Figure 4—source data 1. [file elife-73792-fig4-data1.zip › Figure 4-source data 1/Fig 4J/Figure 4J RIG-I-raw.tif]

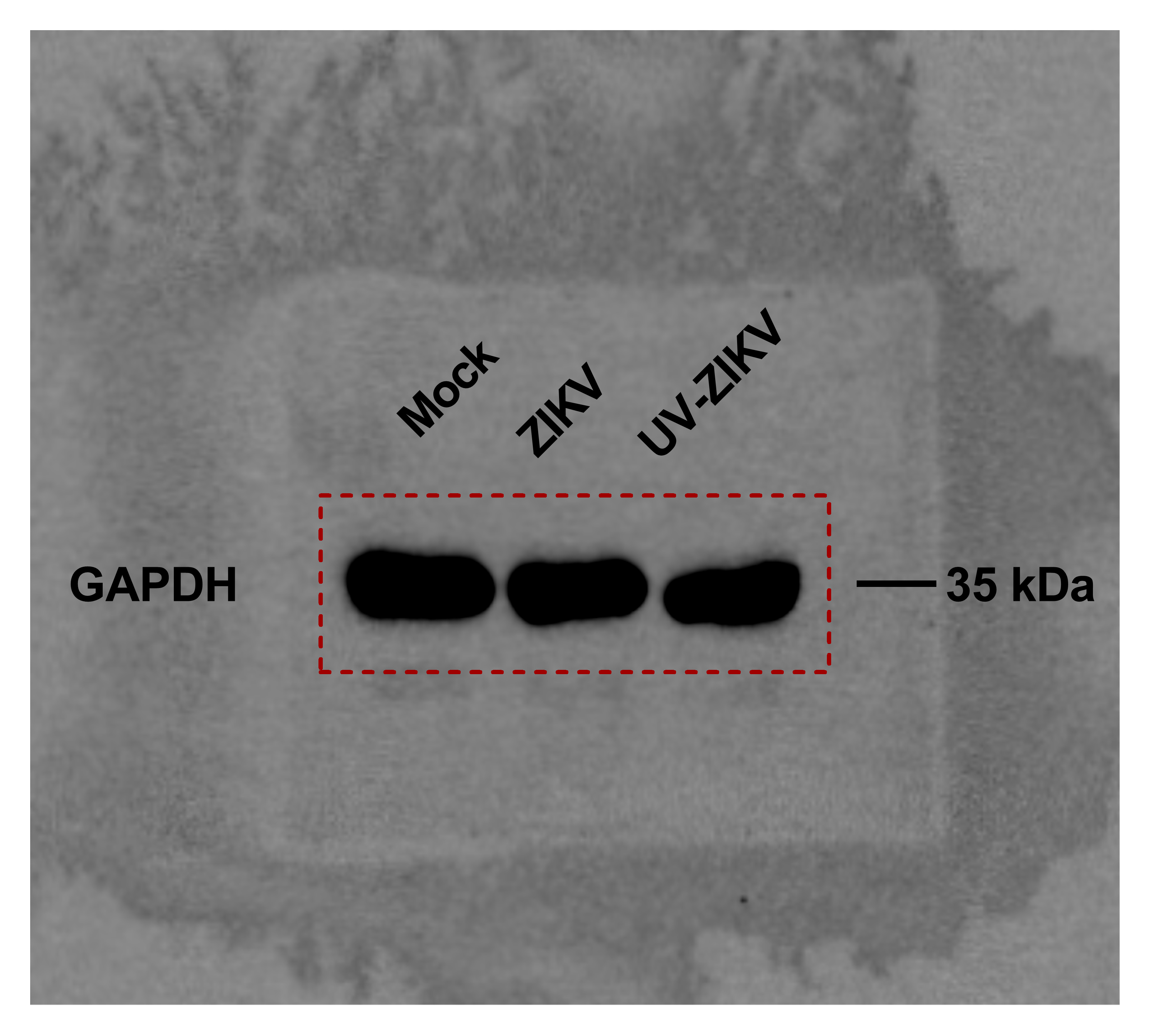

Supplement: Figure 4—figure supplement 1—source data 1. [file elife-73792-fig4-figsupp1-data1.zip › Figure 4-figure supplement 1-source data/1a/Figure 4-figure supplement 1 GAPDH-labeled.tif]

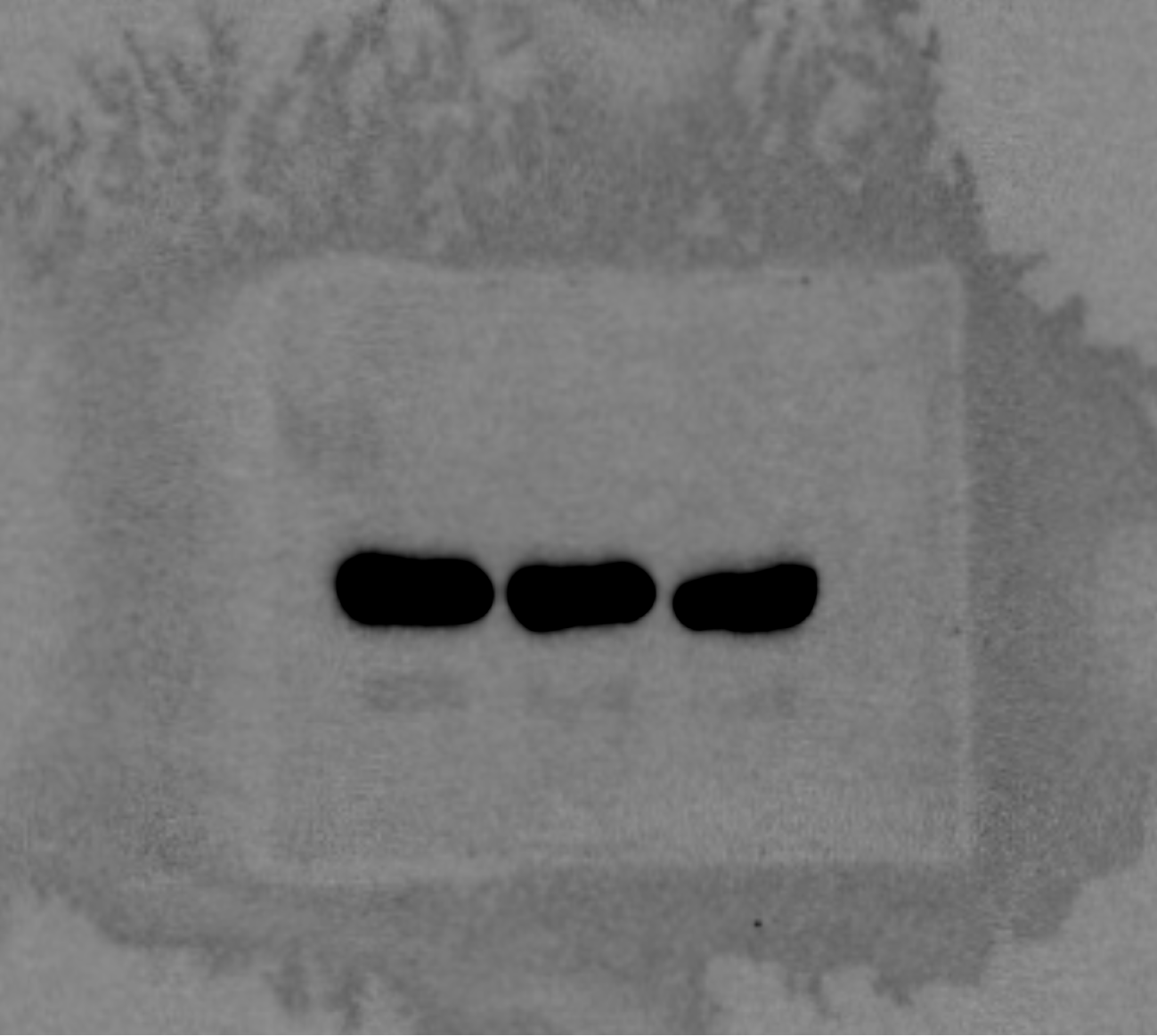

Supplement: Figure 4—figure supplement 1—source data 1. [file elife-73792-fig4-figsupp1-data1.zip › Figure 4-figure supplement 1-source data/1a/Figure 4-figure supplement 1 GAPDH-raw.tif]

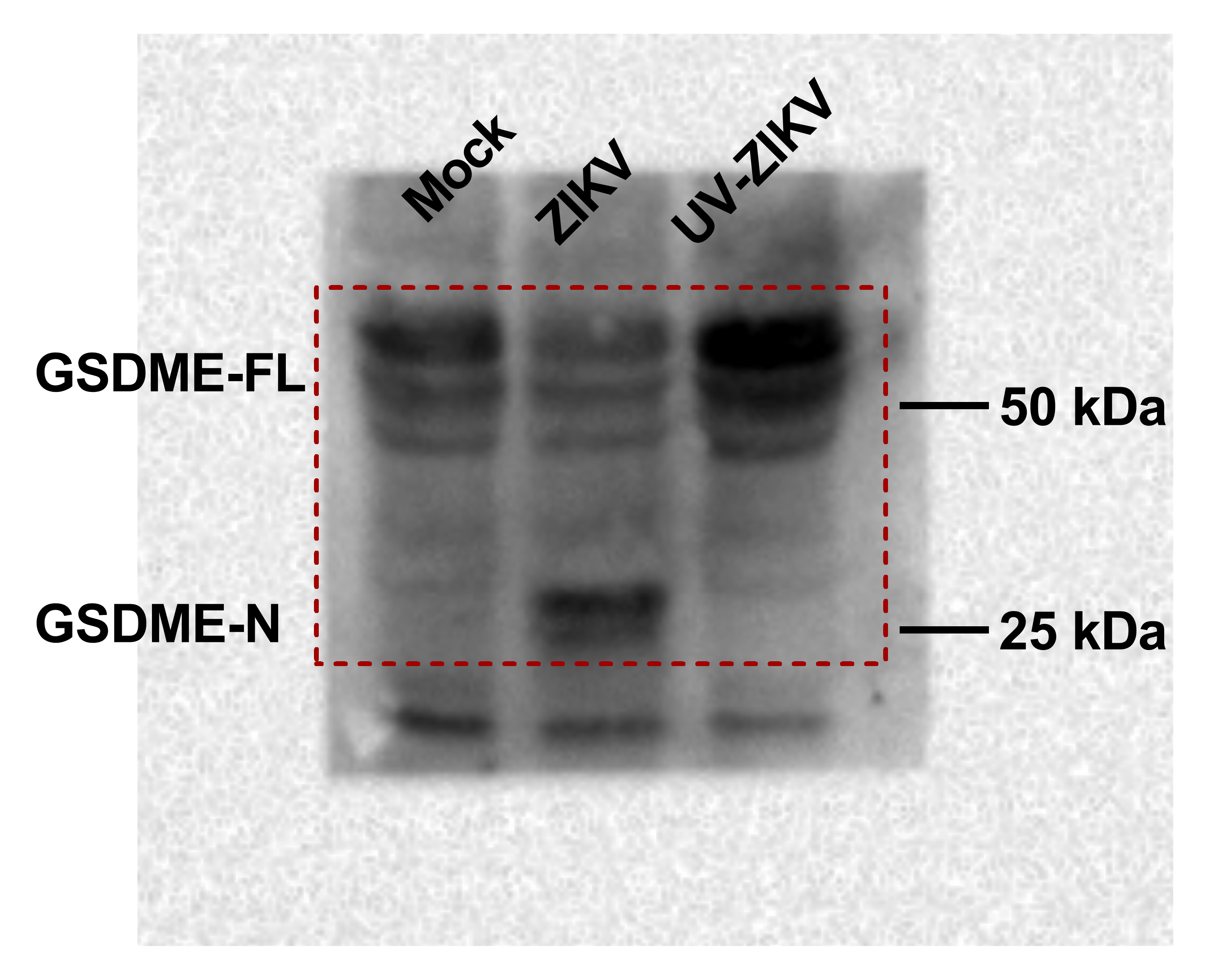

Supplement: Figure 4—figure supplement 1—source data 1. [file elife-73792-fig4-figsupp1-data1.zip › Figure 4-figure supplement 1-source data/1a/Figure 4-figure supplement 1 GSDME-labeled.tif]

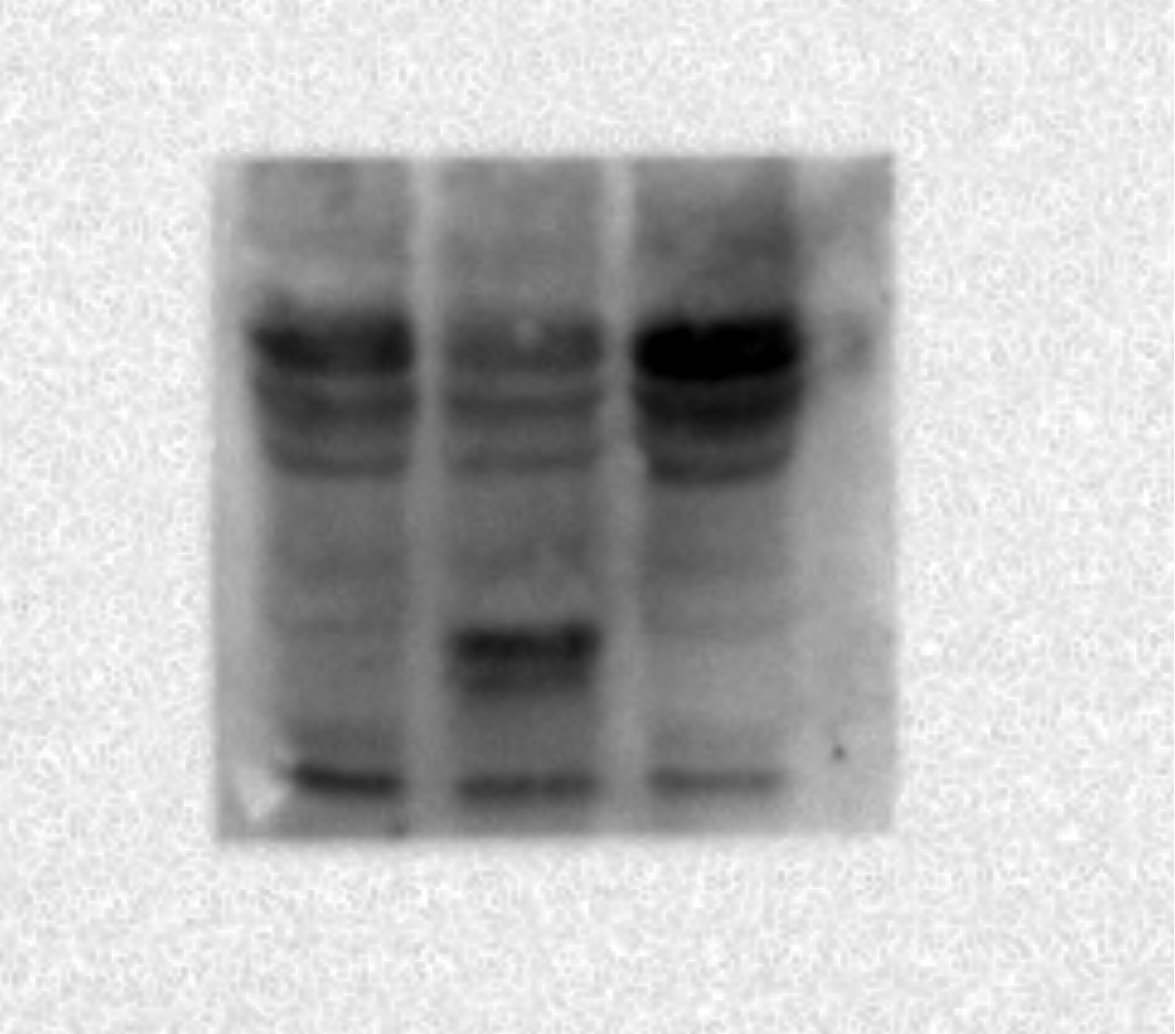

Supplement: Figure 4—figure supplement 1—source data 1. [file elife-73792-fig4-figsupp1-data1.zip › Figure 4-figure supplement 1-source data/1a/Figure 4-figure supplement 1 GSDME-raw.tif]

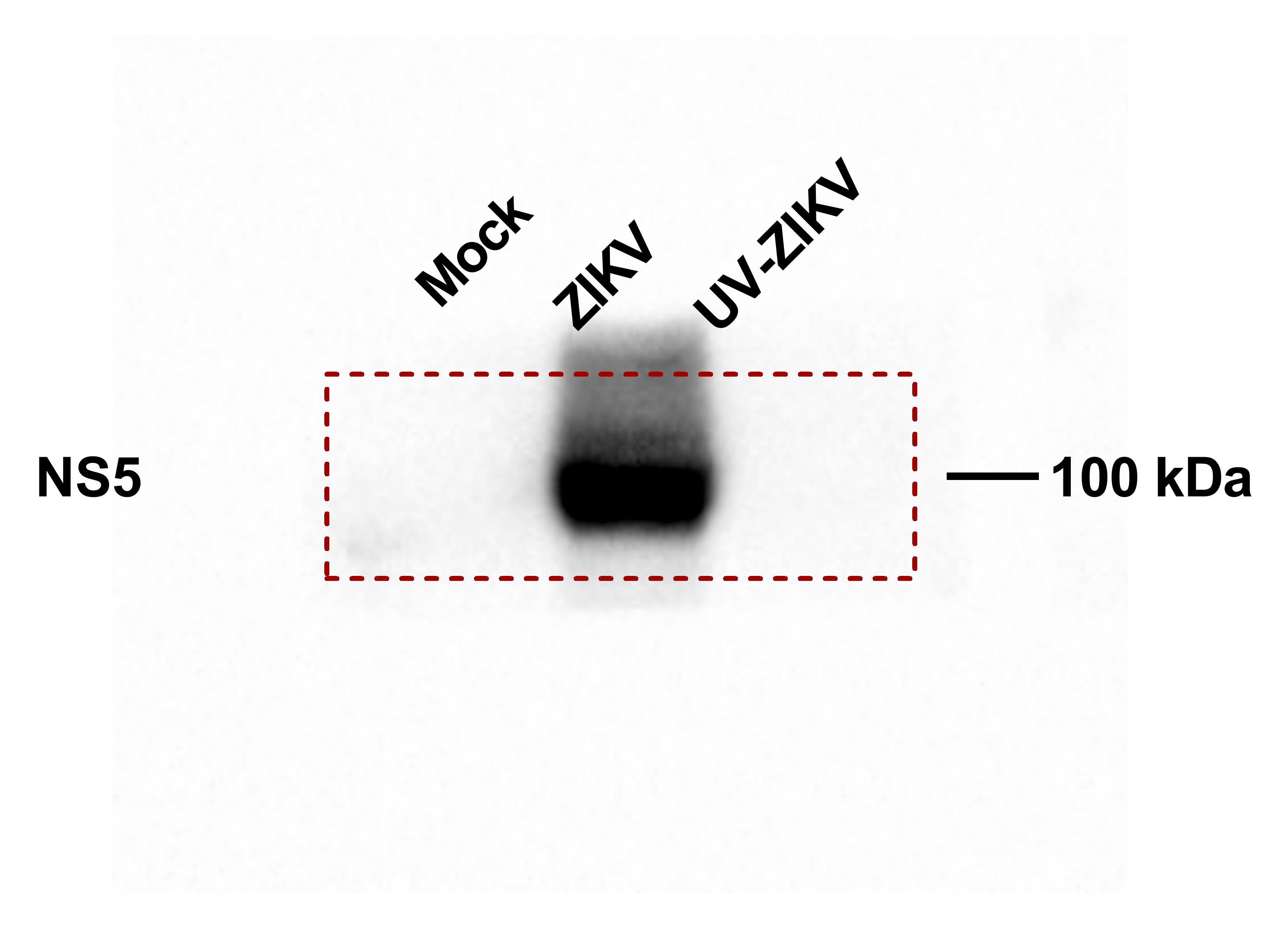

Supplement: Figure 4—figure supplement 1—source data 1. [file elife-73792-fig4-figsupp1-data1.zip › Figure 4-figure supplement 1-source data/1a/Figure 4-figure supplement 1 NS5-labeled.tif]

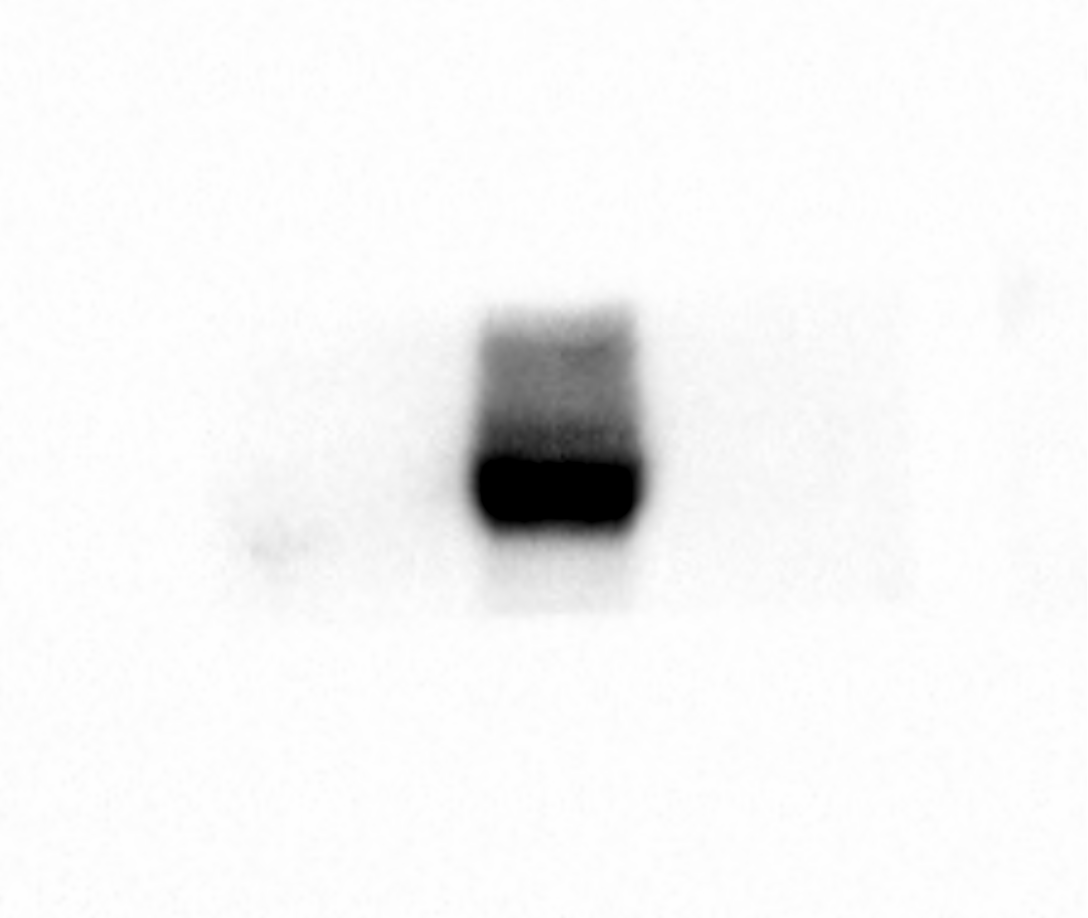

Supplement: Figure 4—figure supplement 1—source data 1. [file elife-73792-fig4-figsupp1-data1.zip › Figure 4-figure supplement 1-source data/1a/Figure 4-figure supplement 1 NS5-raw.tif]

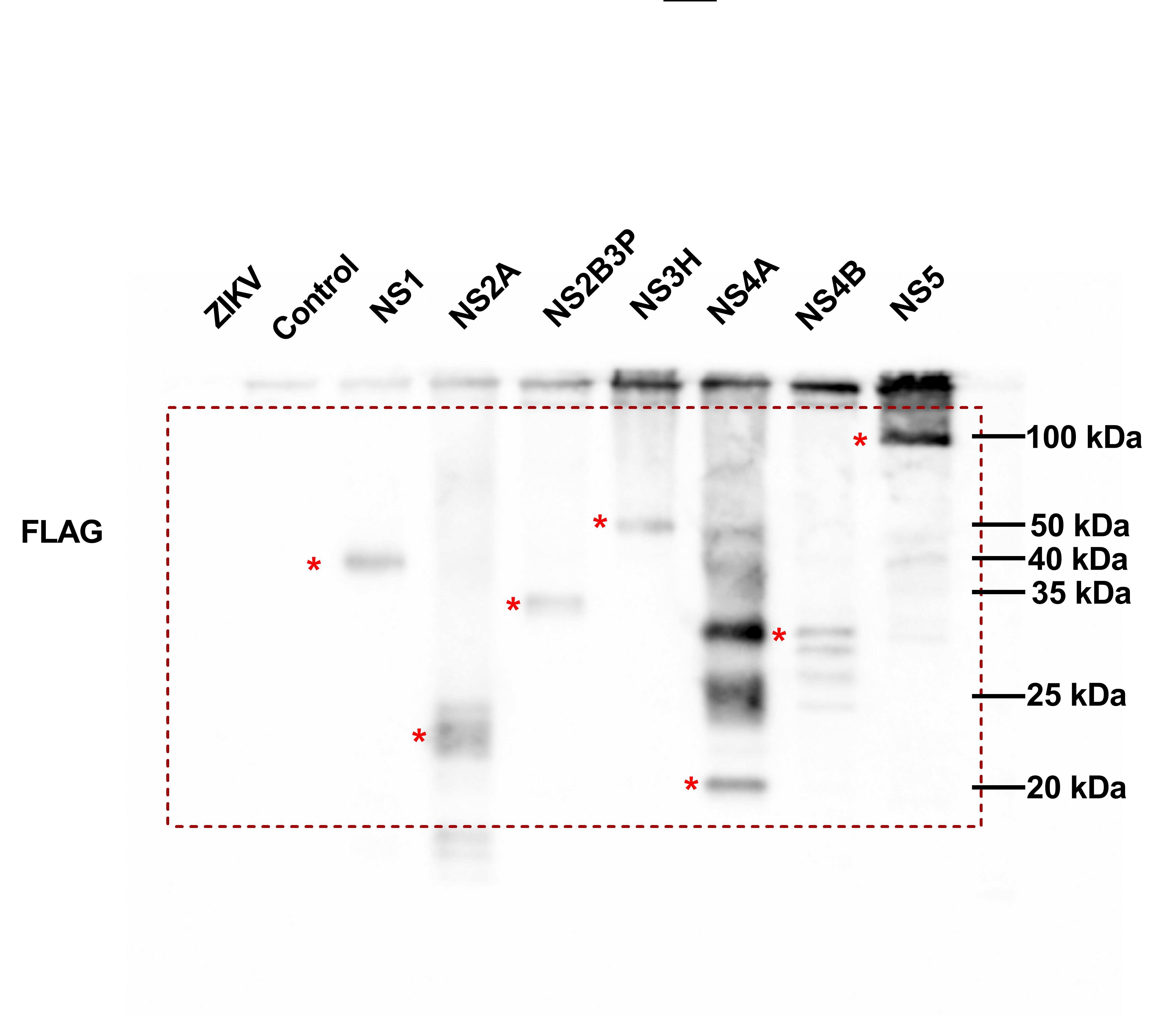

Supplement: Figure 4—figure supplement 1—source data 1. [file elife-73792-fig4-figsupp1-data1.zip › Figure 4-figure supplement 1-source data/1c/Figure 4-figure supplement 2 FLAG-labeled.tif]

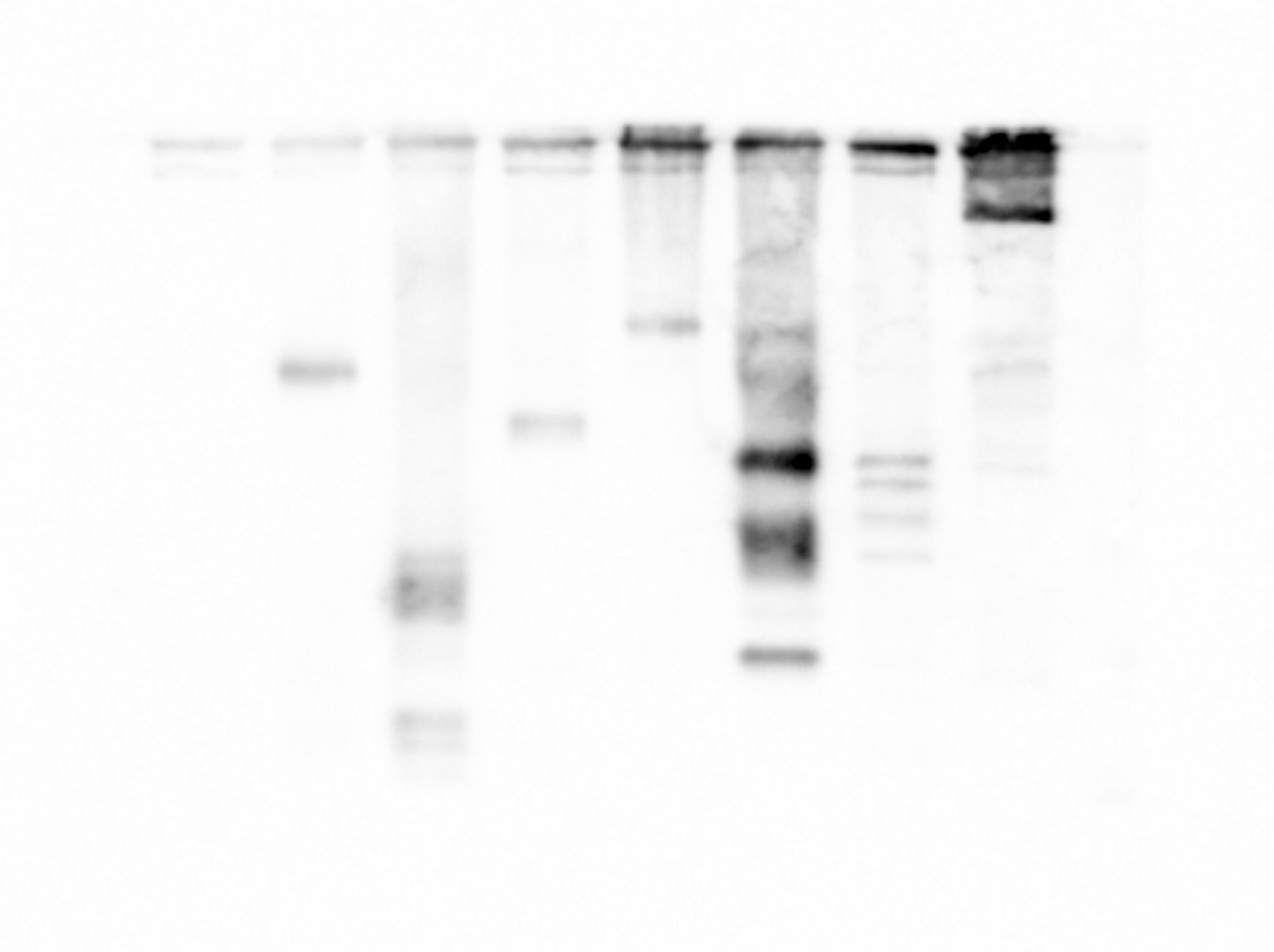

Supplement: Figure 4—figure supplement 1—source data 1. [file elife-73792-fig4-figsupp1-data1.zip › Figure 4-figure supplement 1-source data/1c/Figure 4-figure supplement 2 FLAG-raw.tif]

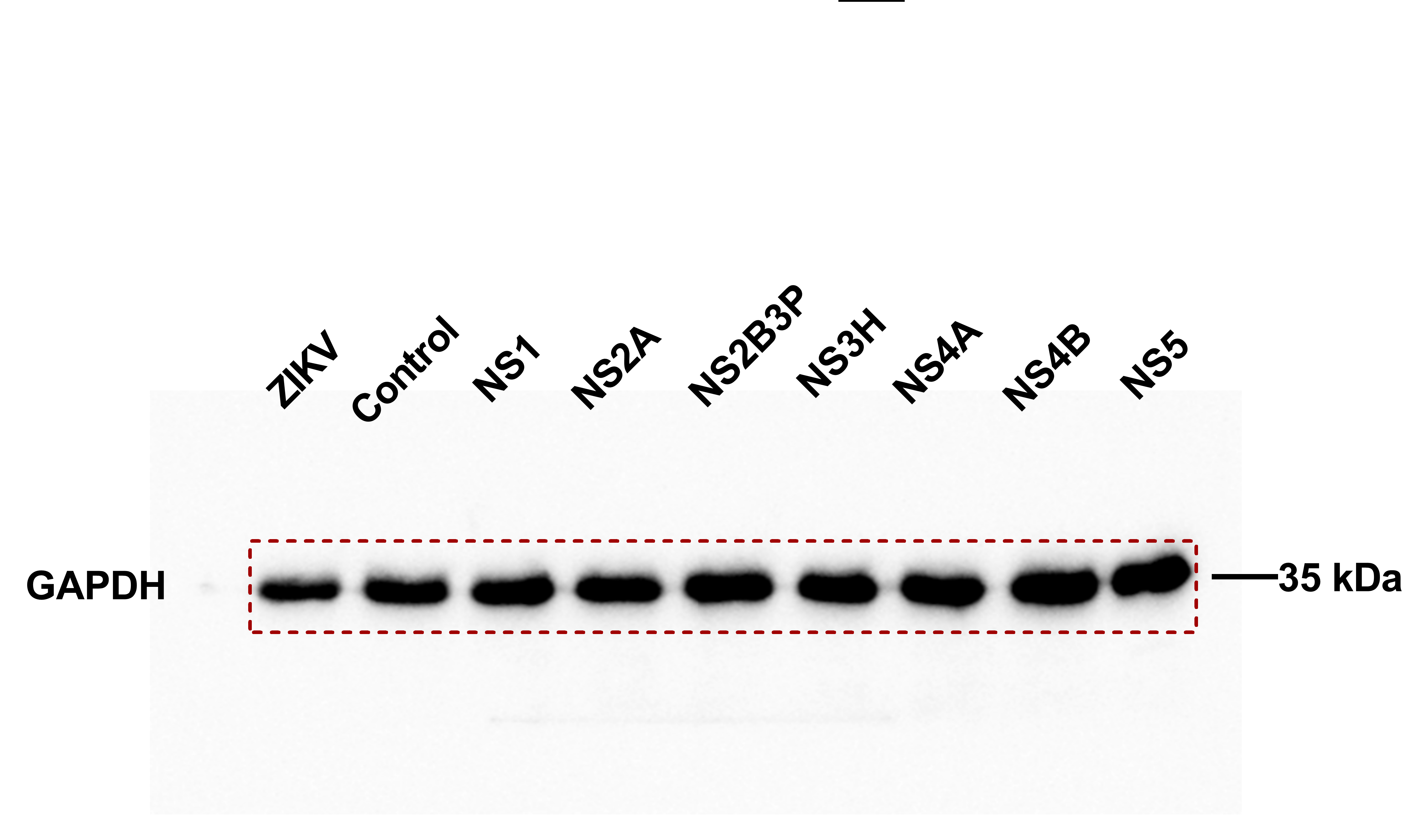

Supplement: Figure 4—figure supplement 1—source data 1. [file elife-73792-fig4-figsupp1-data1.zip › Figure 4-figure supplement 1-source data/1c/Figure 4-figure supplement 2 GAPDH-labeled.tif]

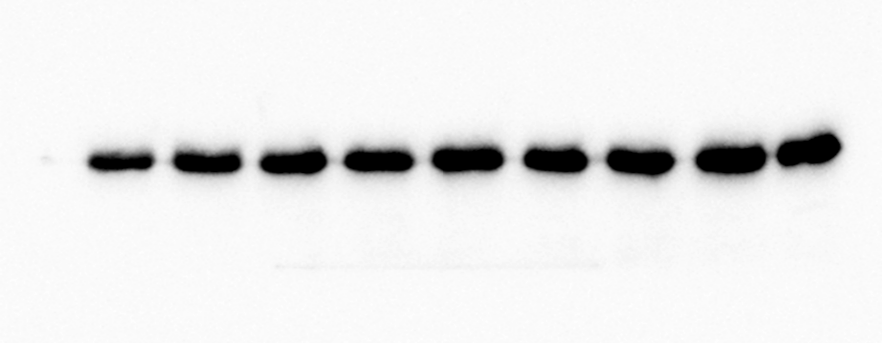

Supplement: Figure 4—figure supplement 1—source data 1. [file elife-73792-fig4-figsupp1-data1.zip › Figure 4-figure supplement 1-source data/1c/Figure 4-figure supplement 2 GAPDH-raw.tif]

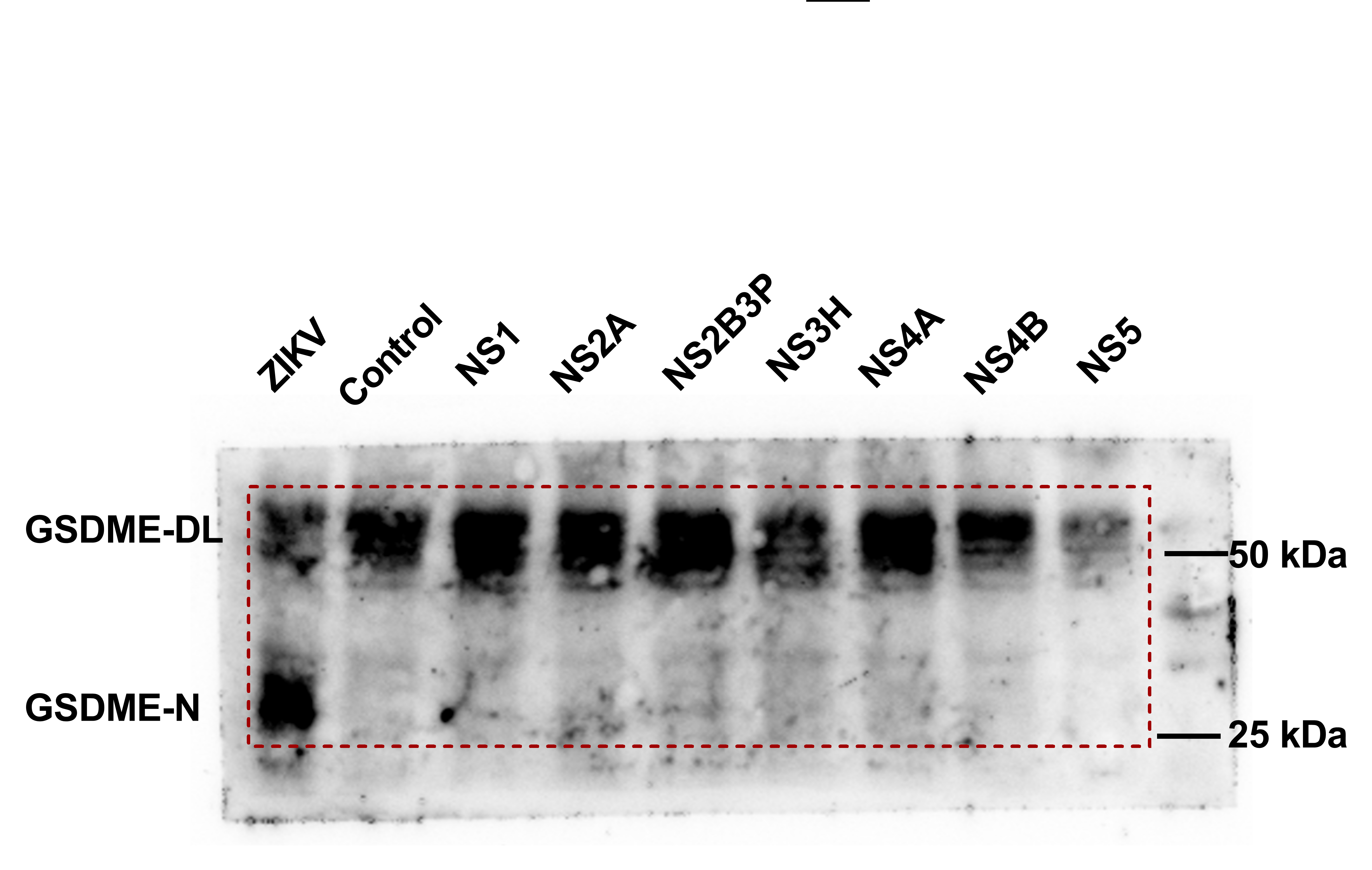

Supplement: Figure 4—figure supplement 1—source data 1. [file elife-73792-fig4-figsupp1-data1.zip › Figure 4-figure supplement 1-source data/1c/Figure 4-figure supplement 2 GSDME-labeled.tif]

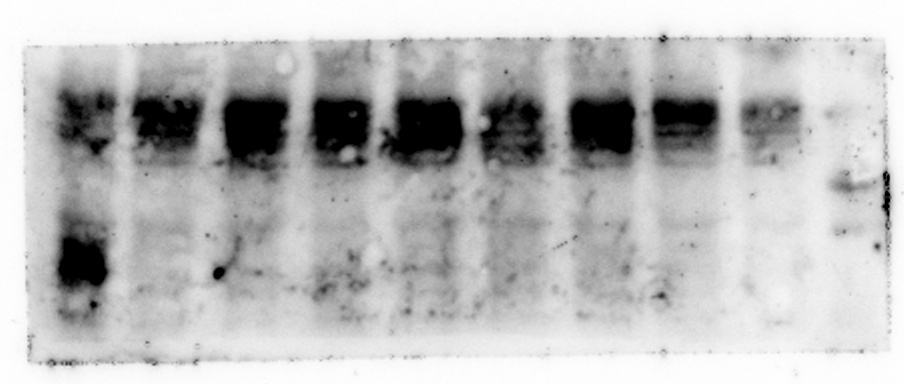

Supplement: Figure 4—figure supplement 1—source data 1. [file elife-73792-fig4-figsupp1-data1.zip › Figure 4-figure supplement 1-source data/1c/Figure 4-figure supplement 2 GSDME-raw.tif]

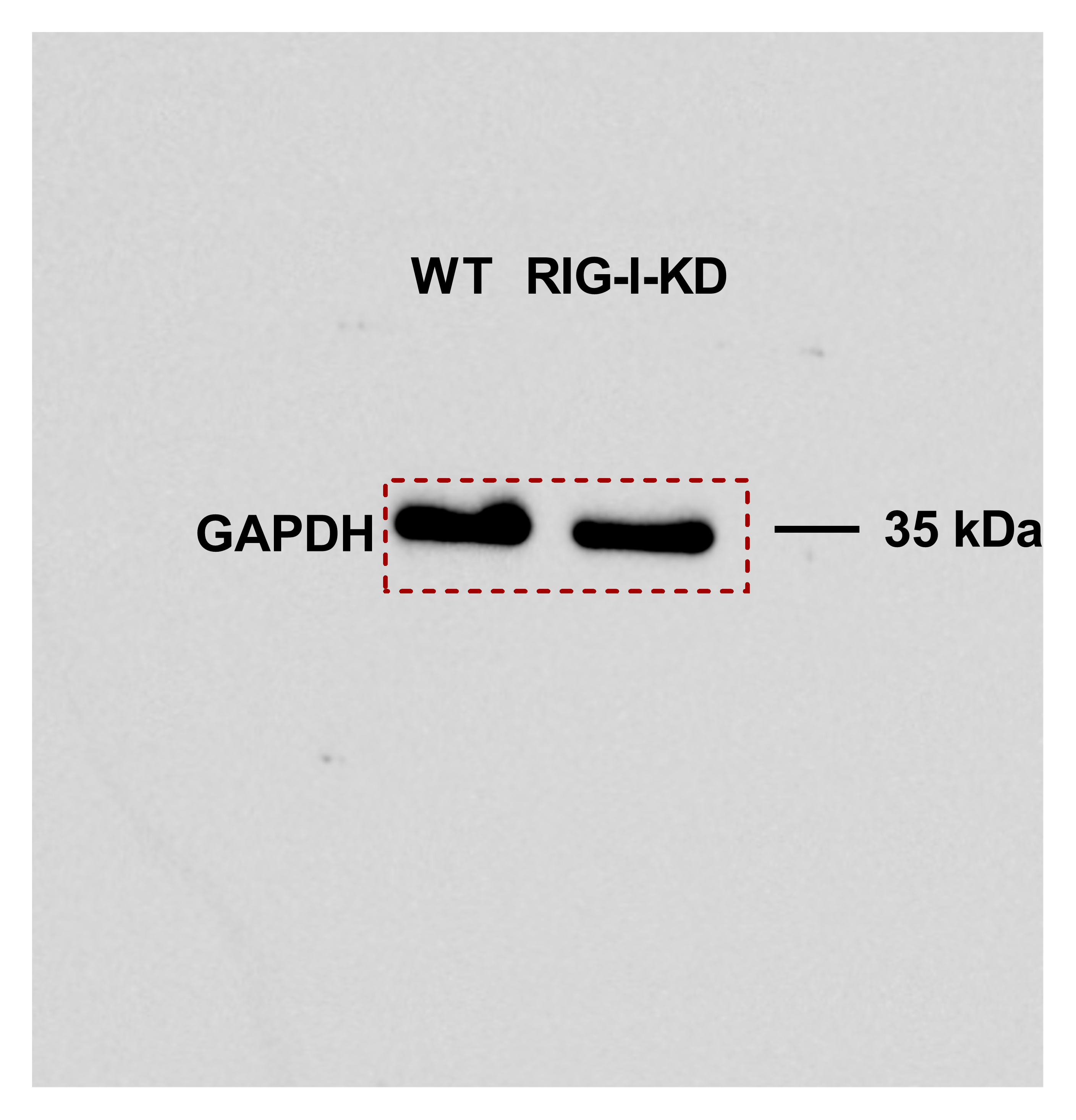

Supplement: Figure 4—figure supplement 2—source data 1. [file elife-73792-fig4-figsupp2-data1.zip › Figure 4-figure supplement 2-source data/2a/Figure 4-figure supplement 2 RIG-I-GAPDH-labeled.tif]

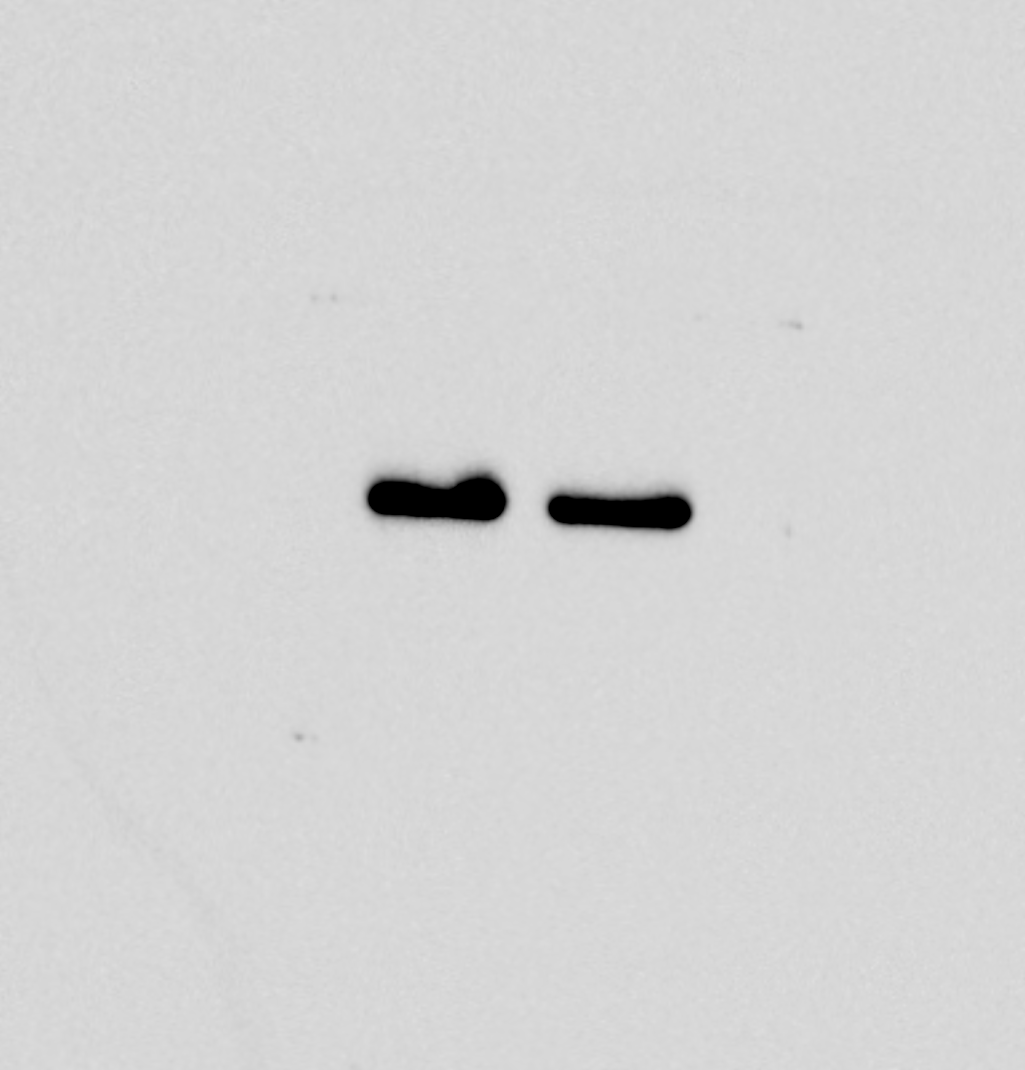

Supplement: Figure 4—figure supplement 2—source data 1. [file elife-73792-fig4-figsupp2-data1.zip › Figure 4-figure supplement 2-source data/2a/Figure 4-figure supplement 2 RIG-I-GAPDH-RAW.tif]

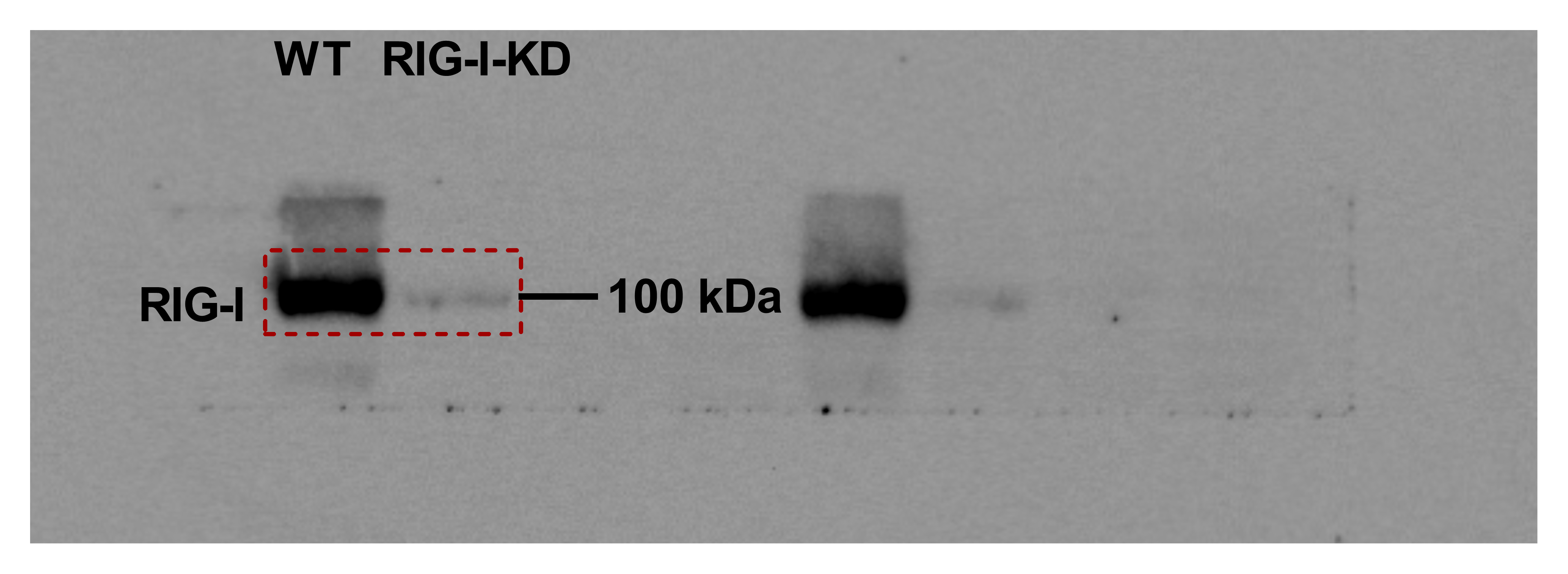

Supplement: Figure 4—figure supplement 2—source data 1. [file elife-73792-fig4-figsupp2-data1.zip › Figure 4-figure supplement 2-source data/2a/Figure 4-figure supplement 2 RIG-I-labeled.tif]

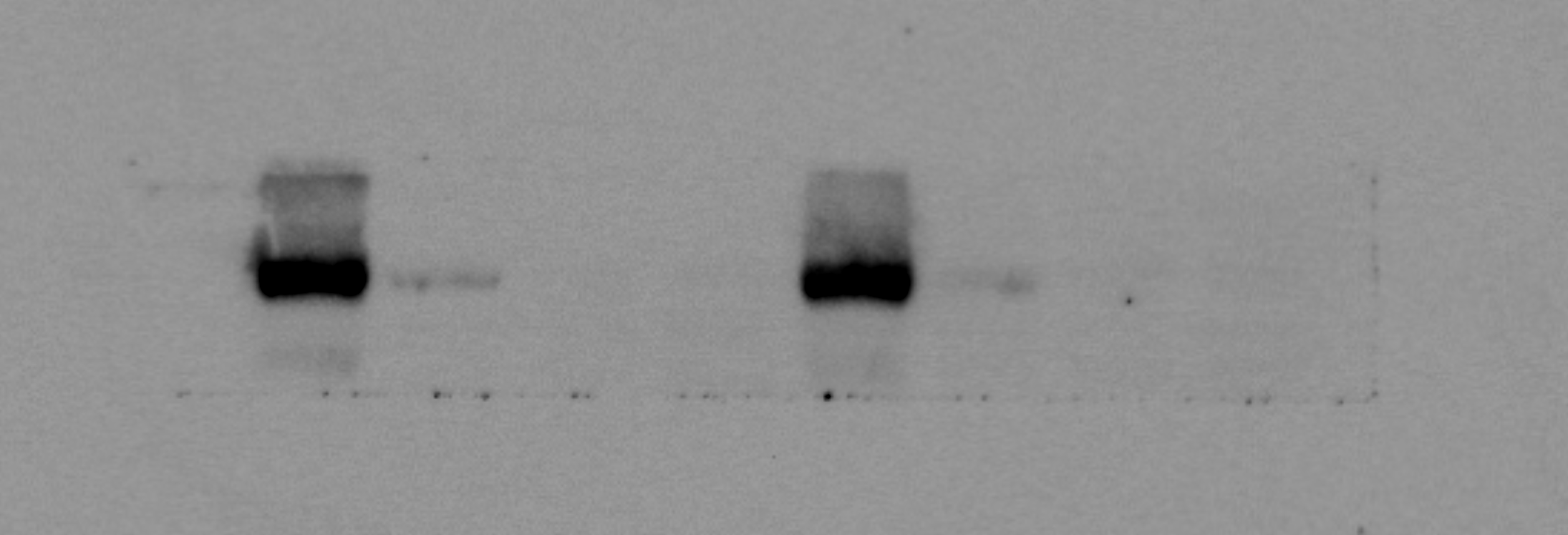

Supplement: Figure 4—figure supplement 2—source data 1. [file elife-73792-fig4-figsupp2-data1.zip › Figure 4-figure supplement 2-source data/2a/Figure 4-figure supplement 2 RIG-I-RAW.tif]

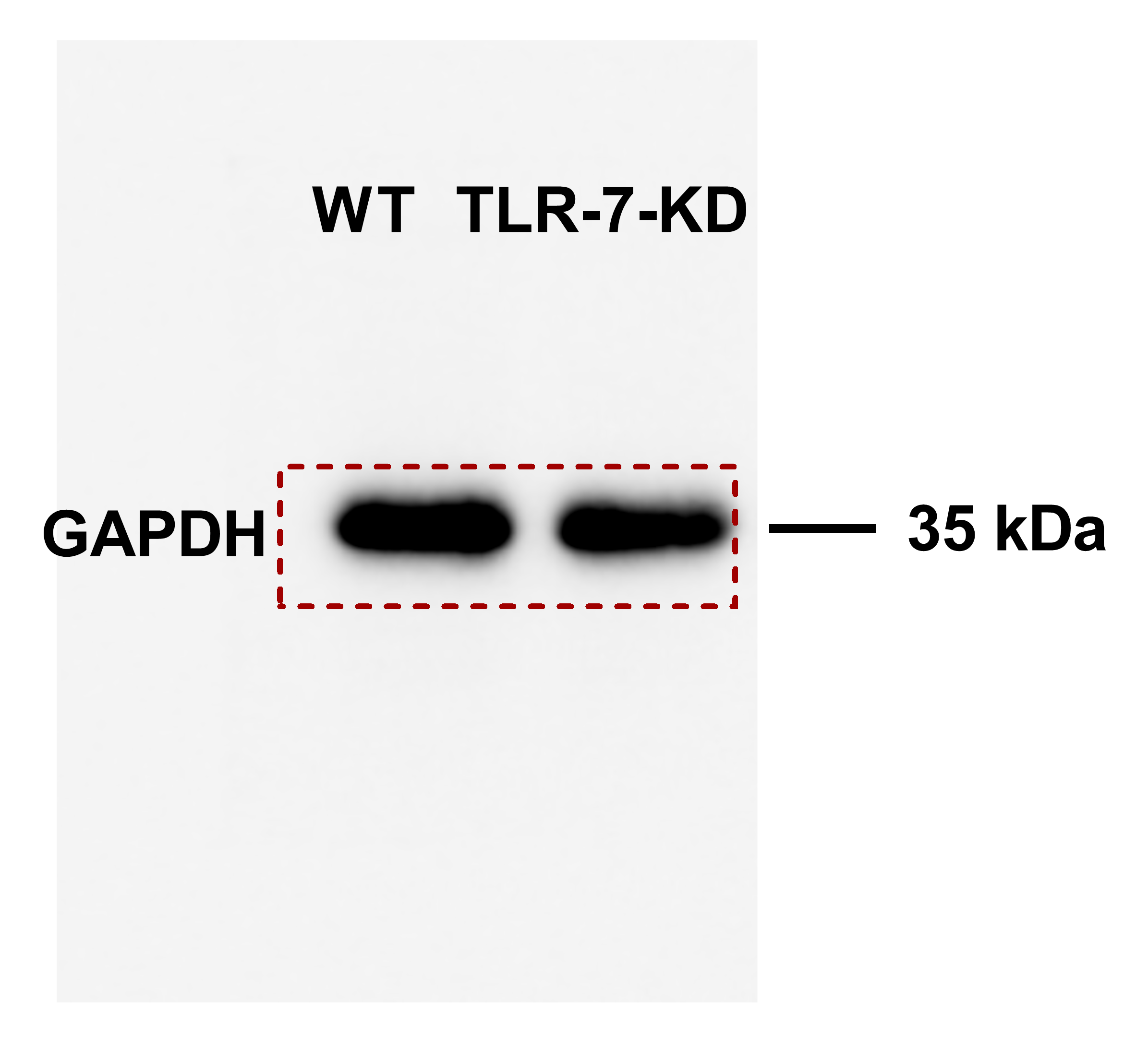

Supplement: Figure 4—figure supplement 2—source data 1. [file elife-73792-fig4-figsupp2-data1.zip › Figure 4-figure supplement 2-source data/2a/Figure 4-figure supplement 2 TLR7-GAPDH-LABELED.tif]

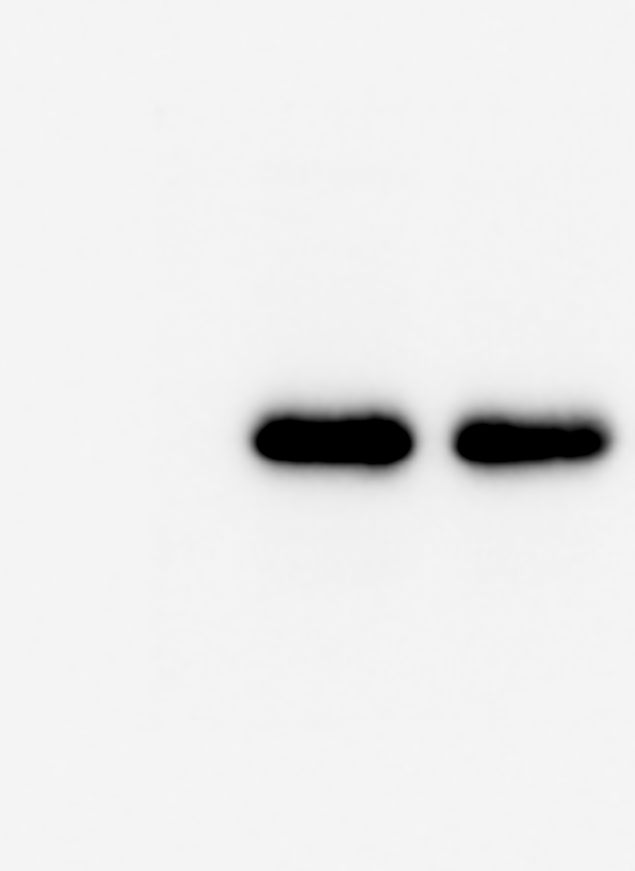

Supplement: Figure 4—figure supplement 2—source data 1. [file elife-73792-fig4-figsupp2-data1.zip › Figure 4-figure supplement 2-source data/2a/Figure 4-figure supplement 2 TLR7-GAPDH-RAW.tif]

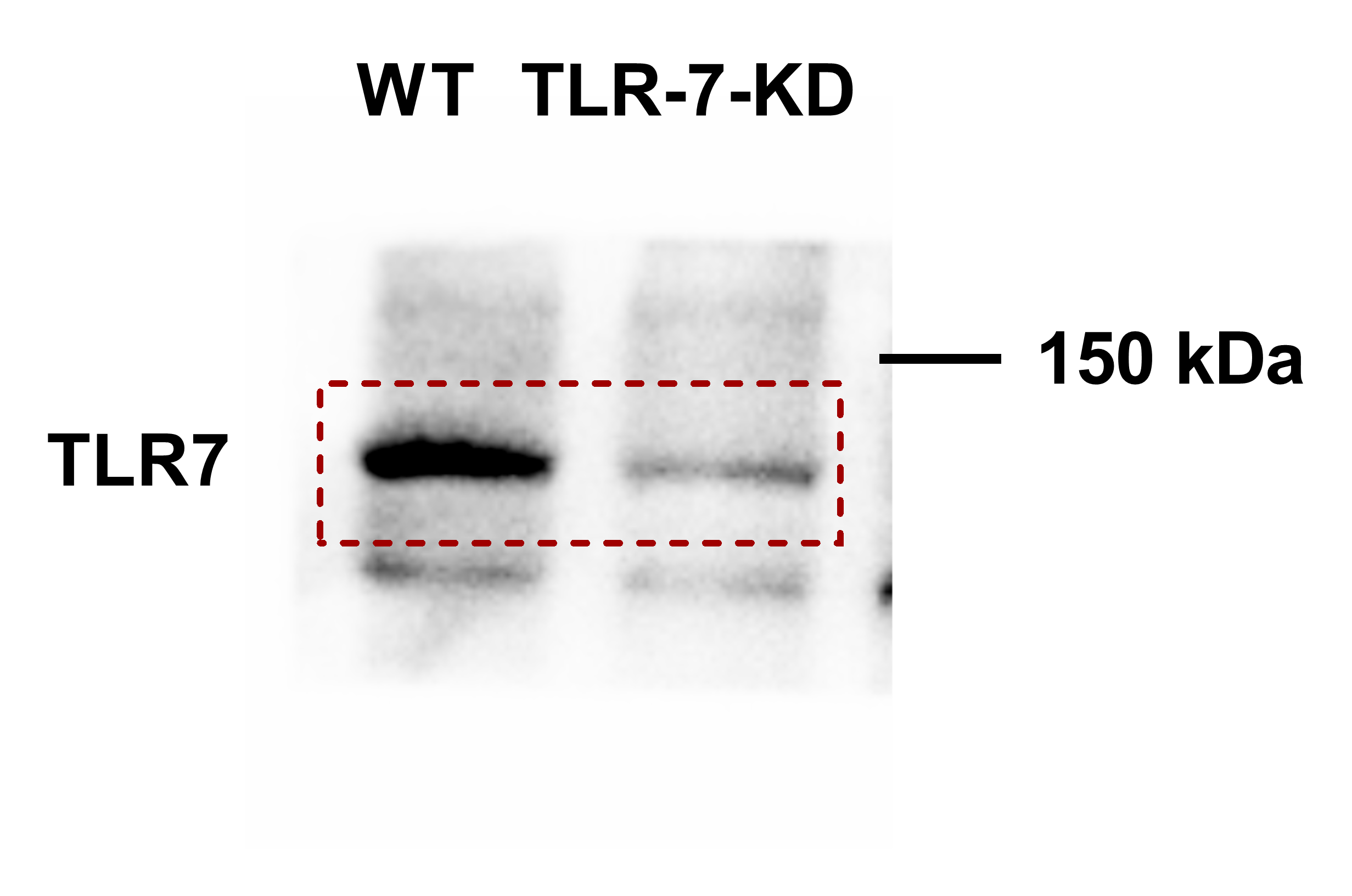

Supplement: Figure 4—figure supplement 2—source data 1. [file elife-73792-fig4-figsupp2-data1.zip › Figure 4-figure supplement 2-source data/2a/Figure 4-figure supplement 2 TLR7-LABELED.tif]
