# Supplementary figures and images for: Zika virus causes placental pyroptosis and associated adverse fetal outcomes by activating GSDME (part 4 of 4)
Source: eLife. 2022 Aug 16;11:e73792. doi: 10.7554/eLife.73792 (PMC9381041; doi:10.7554/eLife.73792)

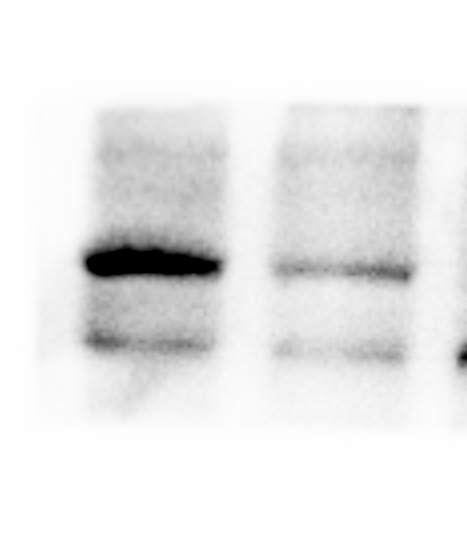

Supplement: Figure 4—figure supplement 2—source data 1. [file elife-73792-fig4-figsupp2-data1.zip › Figure 4-figure supplement 2-source data/2a/Figure 4-figure supplement 2 TLR7-RAW.tif.tif]

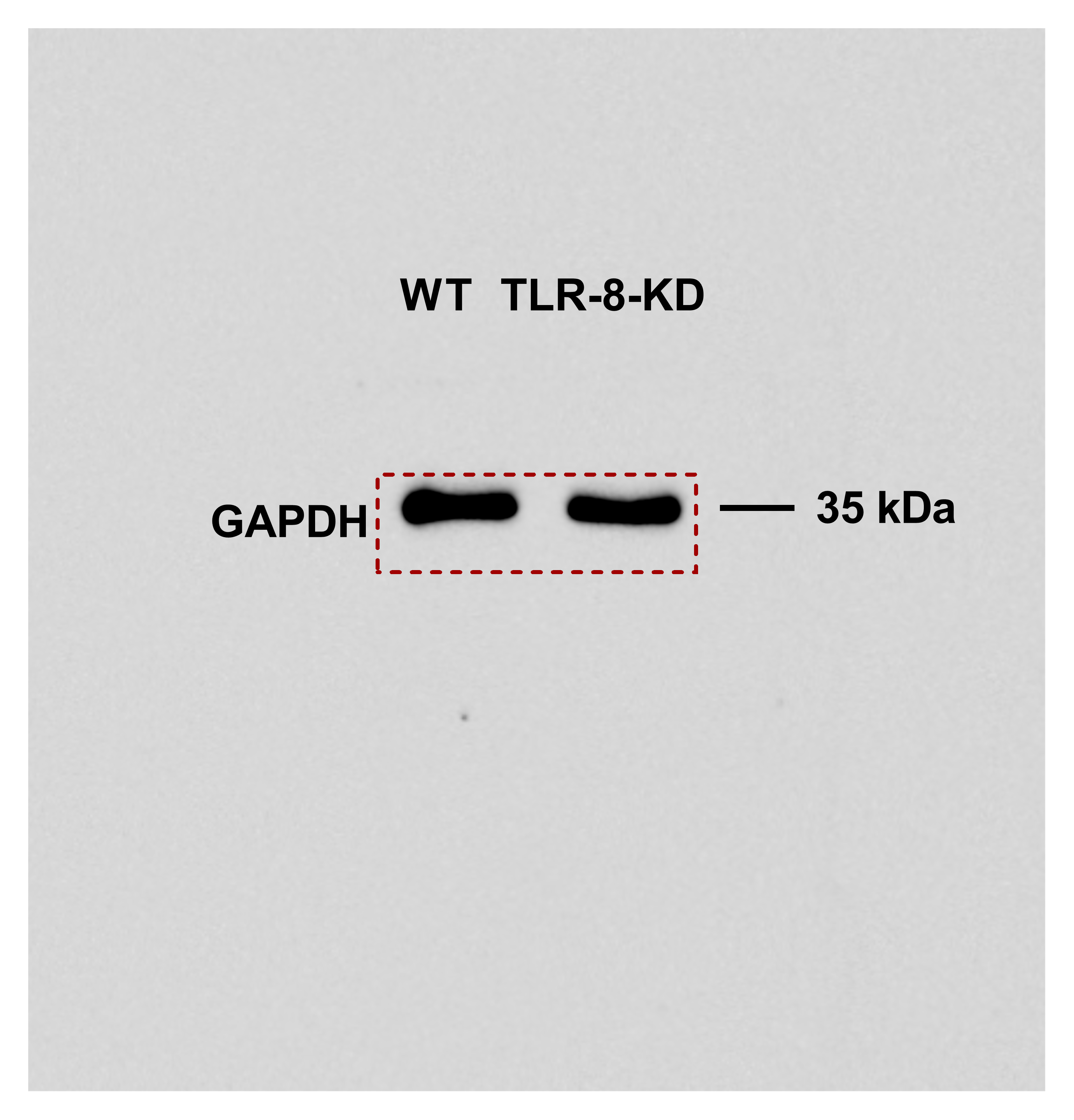

Supplement: Figure 4—figure supplement 2—source data 1. [file elife-73792-fig4-figsupp2-data1.zip › Figure 4-figure supplement 2-source data/2a/Figure 4-figure supplement 2 TLR8-GAPDH-LABELED.tif]

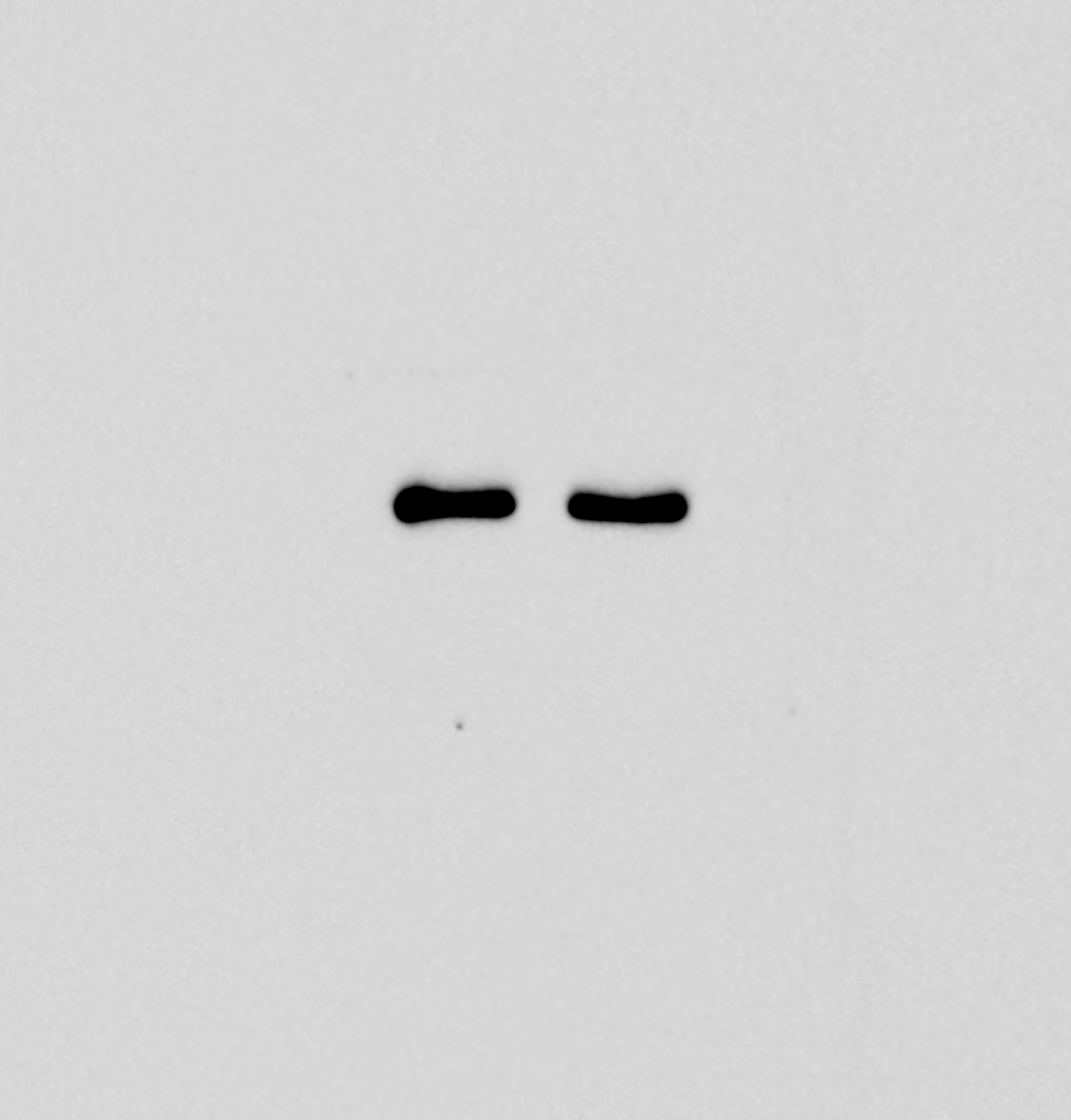

Supplement: Figure 4—figure supplement 2—source data 1. [file elife-73792-fig4-figsupp2-data1.zip › Figure 4-figure supplement 2-source data/2a/Figure 4-figure supplement 2 TLR8-GAPDH-RAW.tif.tif]

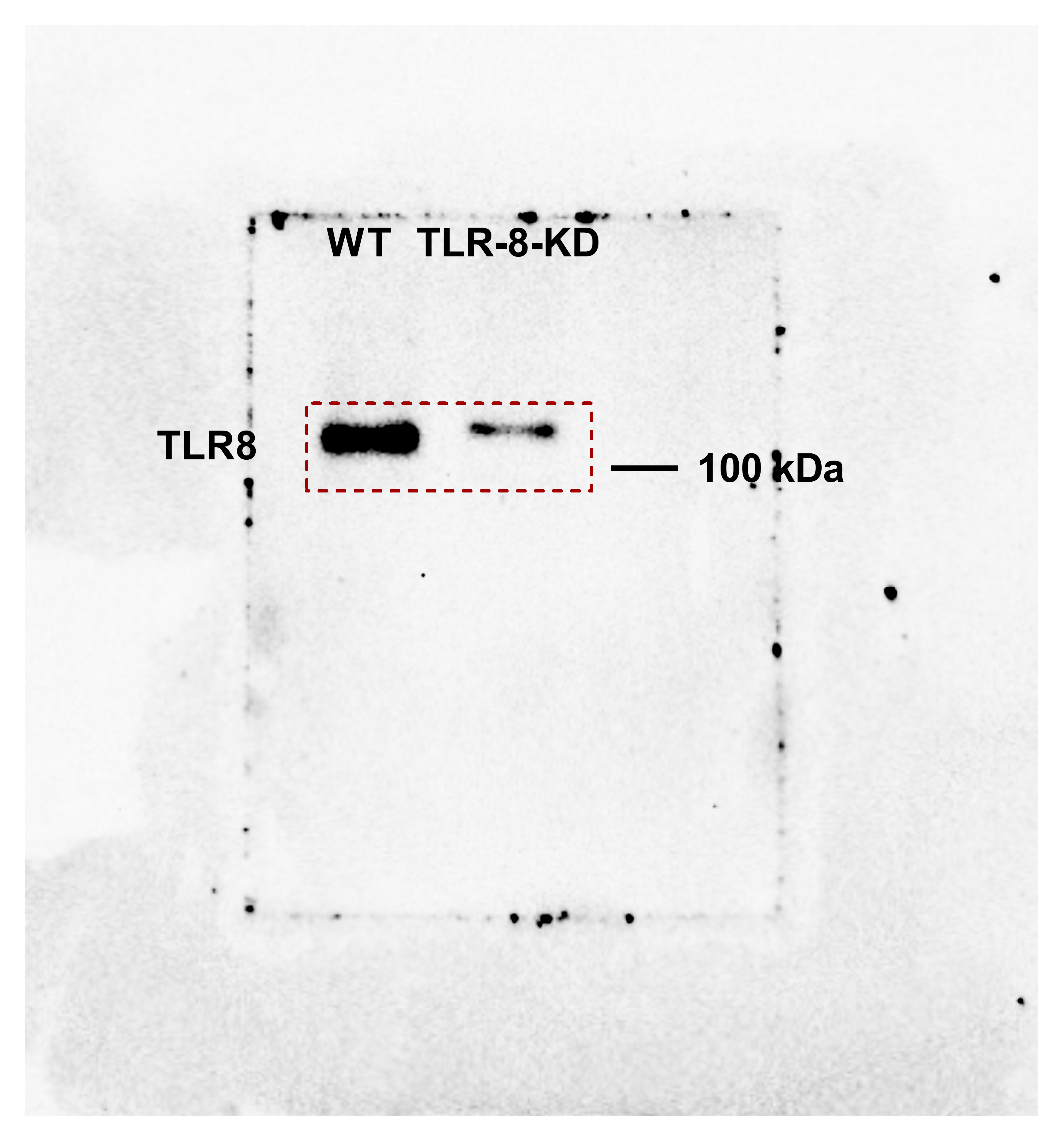

Supplement: Figure 4—figure supplement 2—source data 1. [file elife-73792-fig4-figsupp2-data1.zip › Figure 4-figure supplement 2-source data/2a/Figure 4-figure supplement 2 TLR8-LABELED.tif]

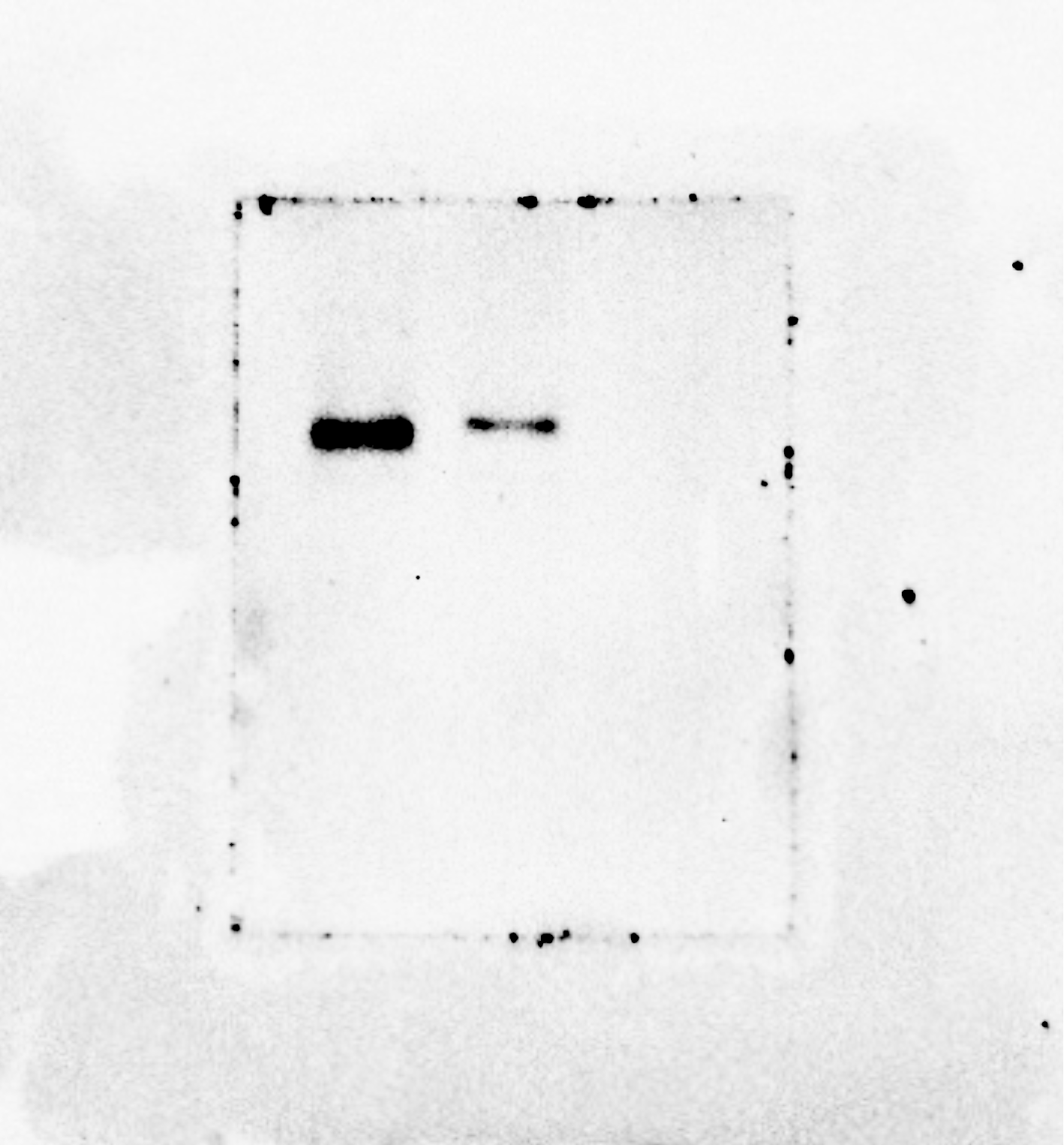

Supplement: Figure 4—figure supplement 2—source data 1. [file elife-73792-fig4-figsupp2-data1.zip › Figure 4-figure supplement 2-source data/2a/Figure 4-figure supplement 2 TLR8-RAW.tif.tif]

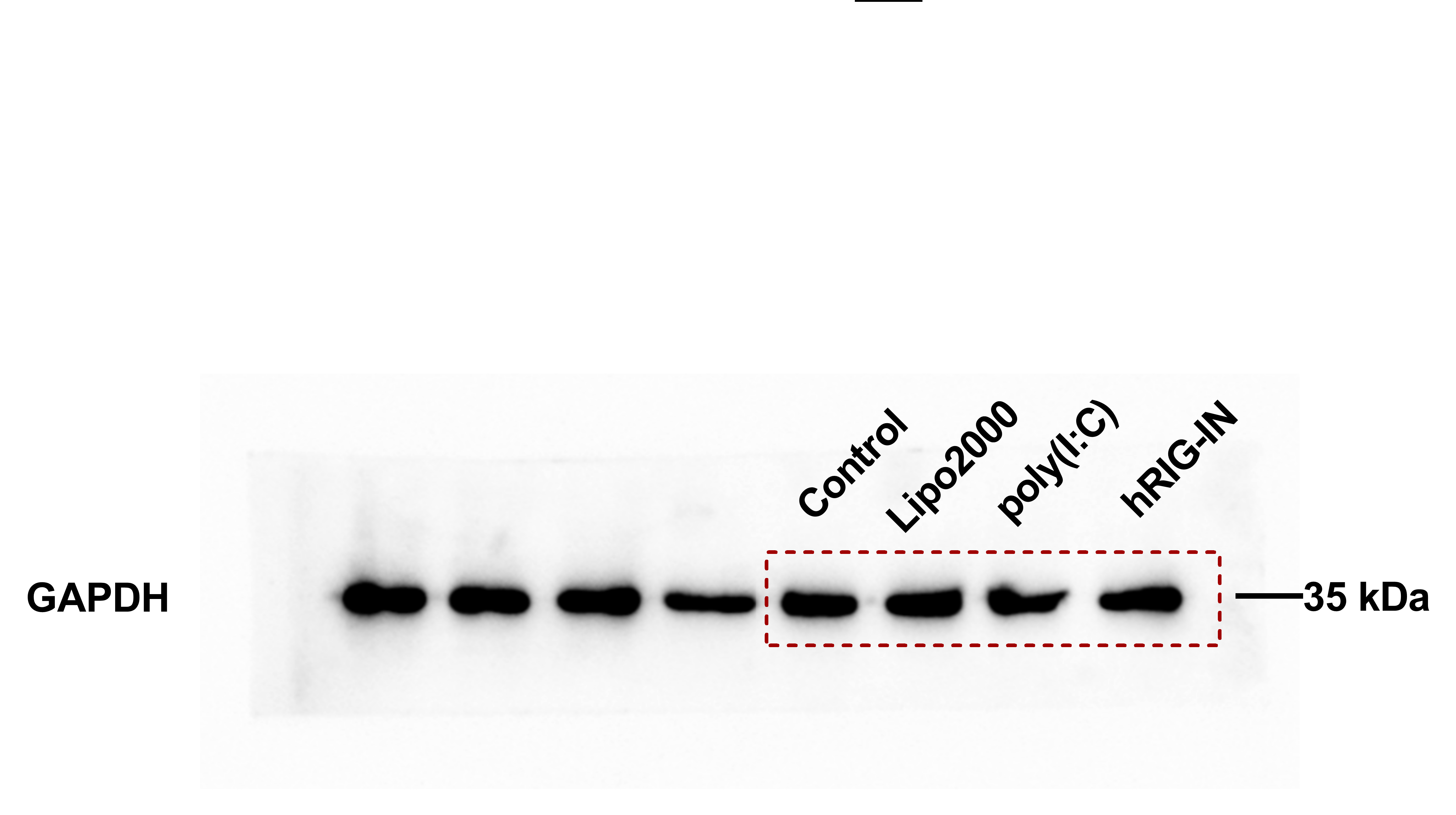

Supplement: Figure 4—figure supplement 2—source data 1. [file elife-73792-fig4-figsupp2-data1.zip › Figure 4-figure supplement 2-source data/2c/Figure 4-figure supplement 2C GAPDH-labeled.tif]

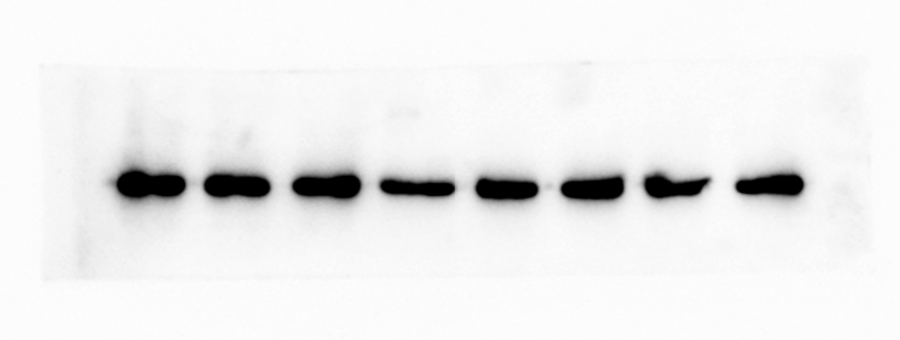

Supplement: Figure 4—figure supplement 2—source data 1. [file elife-73792-fig4-figsupp2-data1.zip › Figure 4-figure supplement 2-source data/2c/Figure 4-figure supplement 2C GAPDH-raw.tif]

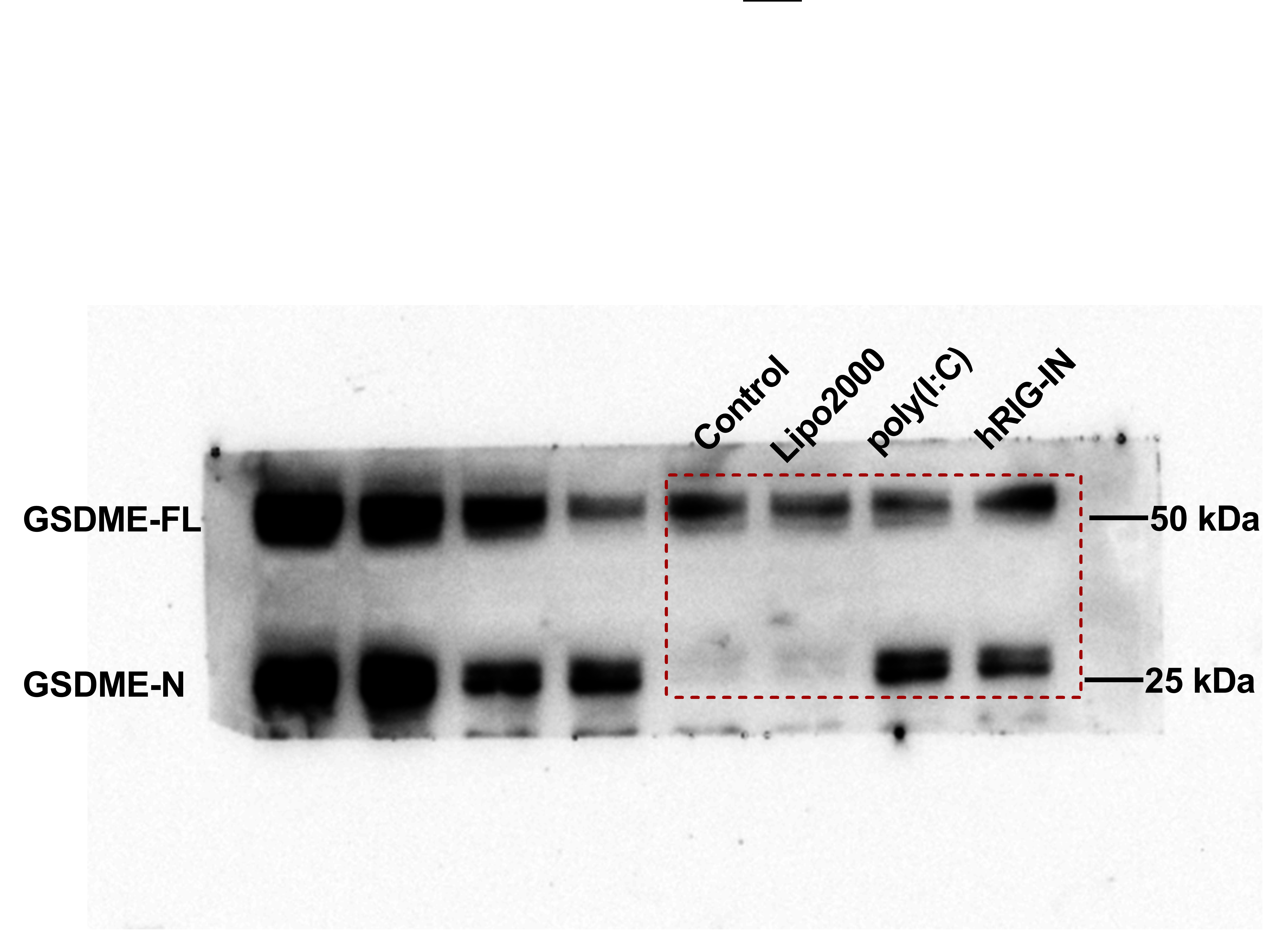

Supplement: Figure 4—figure supplement 2—source data 1. [file elife-73792-fig4-figsupp2-data1.zip › Figure 4-figure supplement 2-source data/2c/Figure 4-figure supplement 2C GSDME-labeled.tif]

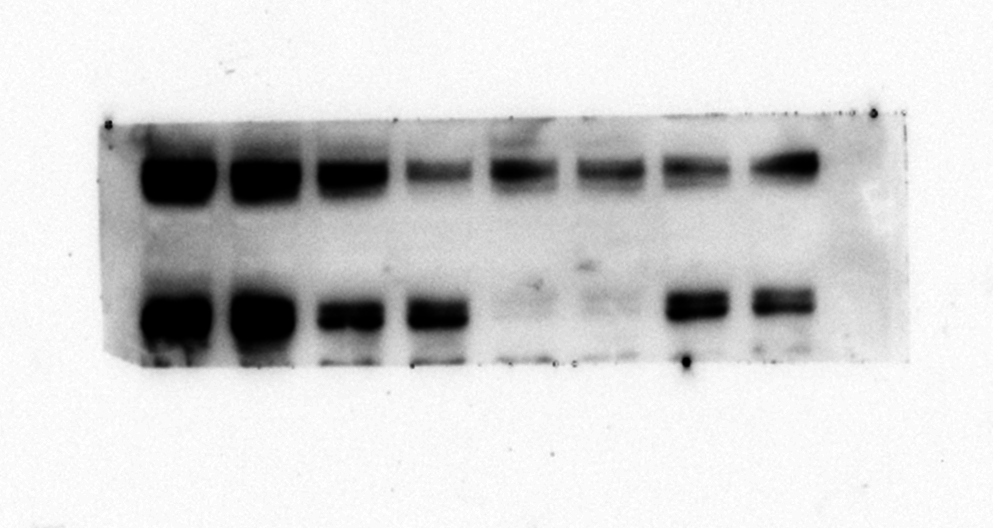

Supplement: Figure 4—figure supplement 2—source data 1. [file elife-73792-fig4-figsupp2-data1.zip › Figure 4-figure supplement 2-source data/2c/Figure 4-figure supplement 2C GSDME-raw.tif]

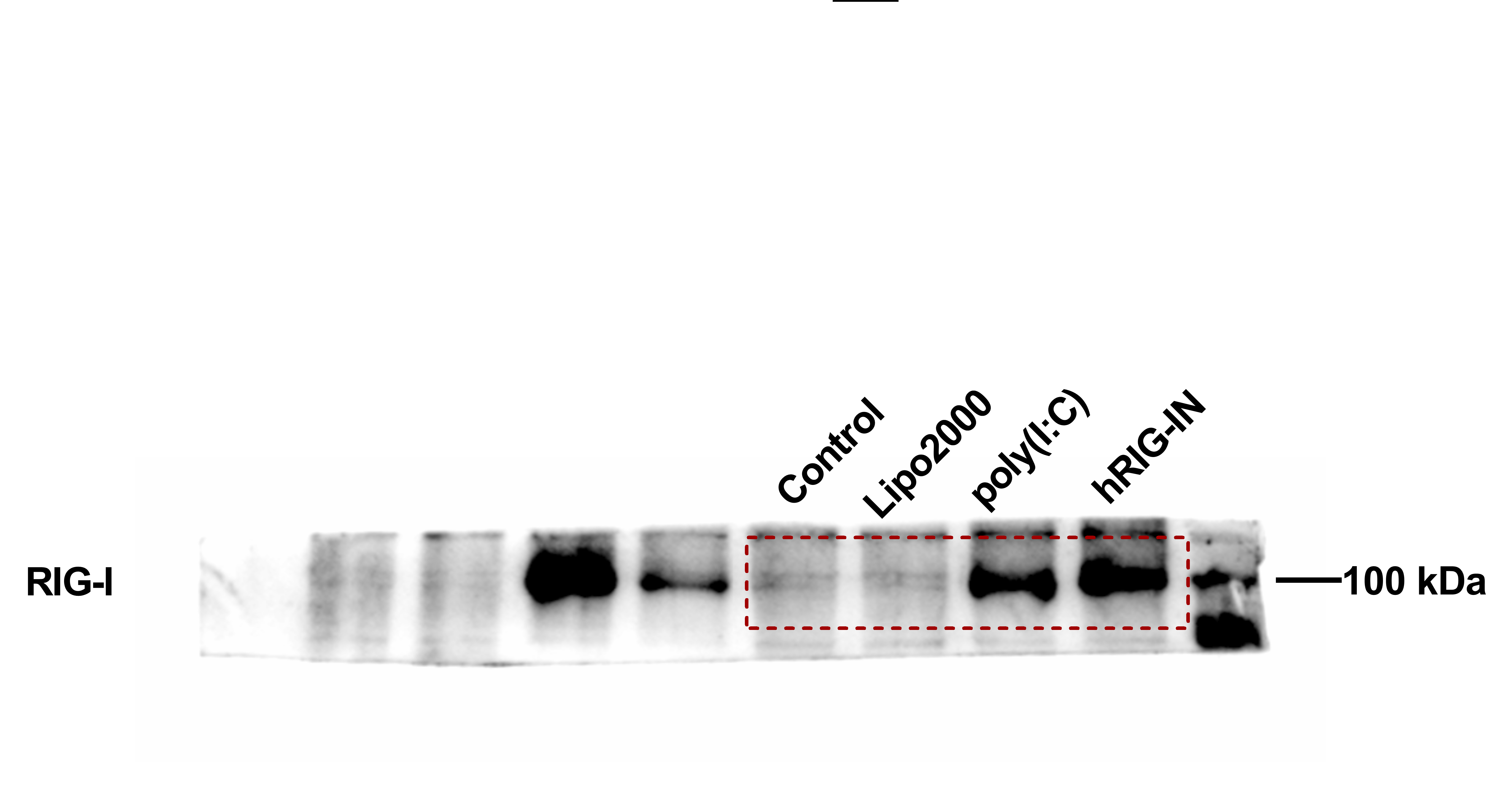

Supplement: Figure 4—figure supplement 2—source data 1. [file elife-73792-fig4-figsupp2-data1.zip › Figure 4-figure supplement 2-source data/2c/Figure 4-figure supplement 2C RIG-I-LABELED.tif]

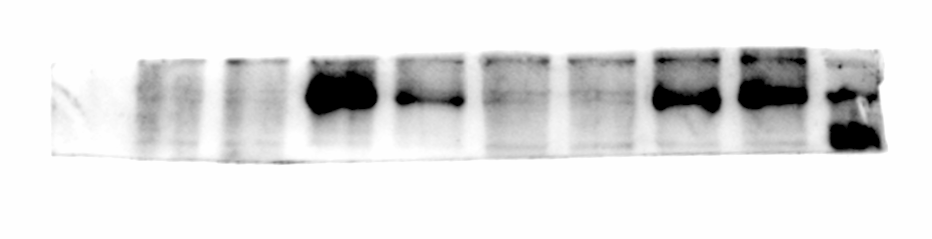

Supplement: Figure 4—figure supplement 2—source data 1. [file elife-73792-fig4-figsupp2-data1.zip › Figure 4-figure supplement 2-source data/2c/Figure 4-figure supplement 2C RIG-I-raw.tif]

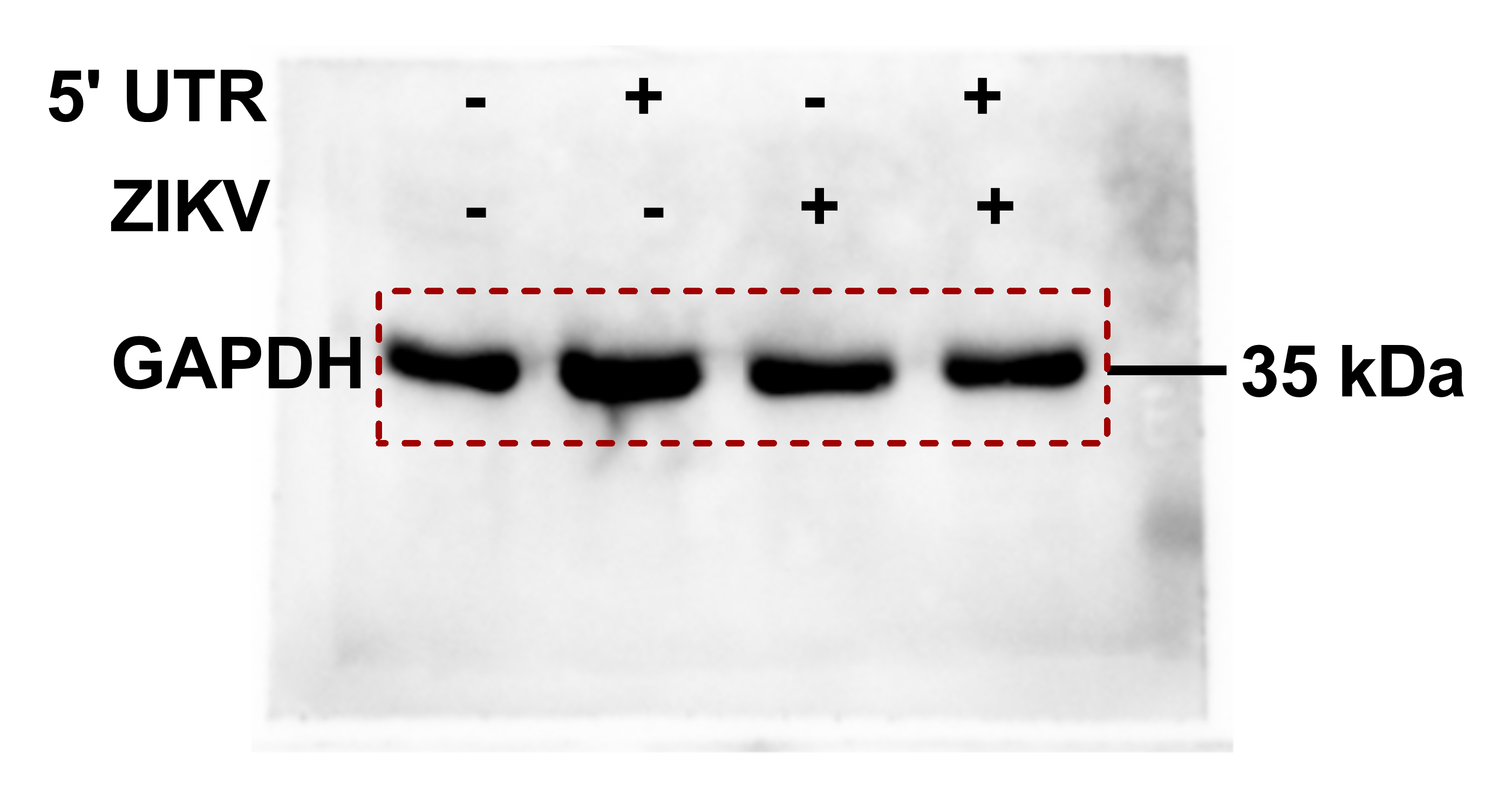

Supplement: Figure 4—figure supplement 2—source data 1. [file elife-73792-fig4-figsupp2-data1.zip › Figure 4-figure supplement 2-source data/2e/Figure 4-figure supplement 2E GAPDH-labeled.tif]

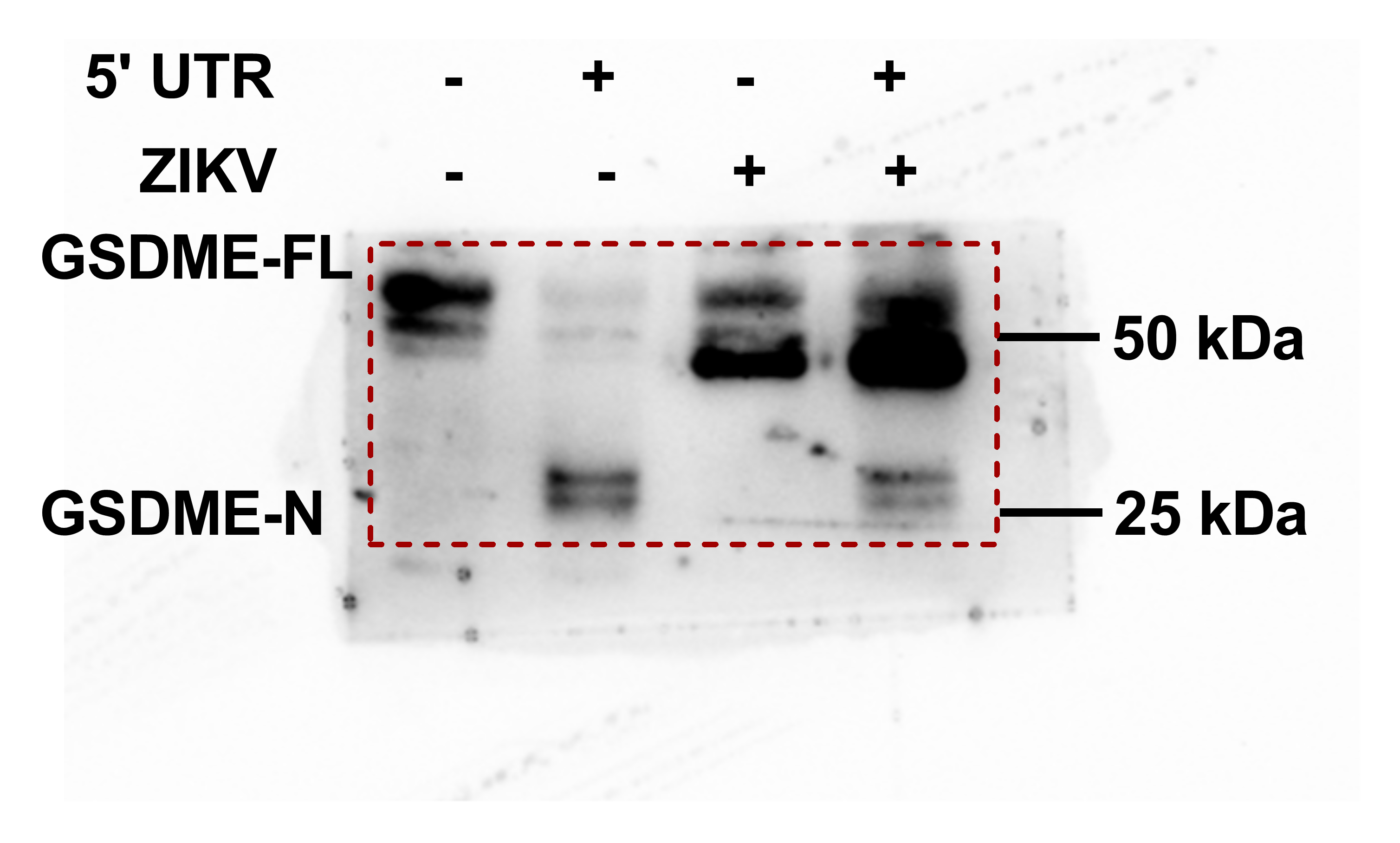

Supplement: Figure 4—figure supplement 2—source data 1. [file elife-73792-fig4-figsupp2-data1.zip › Figure 4-figure supplement 2-source data/2e/Figure 4-figure supplement 2E GSDME-LABELED.tif]

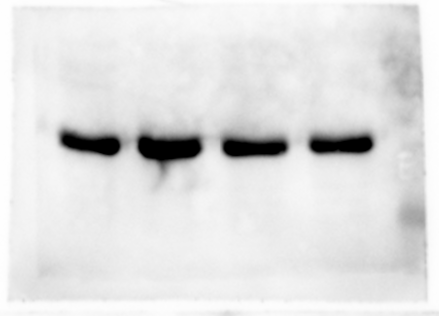

Supplement: Figure 4—figure supplement 2—source data 1. [file elife-73792-fig4-figsupp2-data1.zip › Figure 4-figure supplement 2-source data/2e/GAPDH.tif]

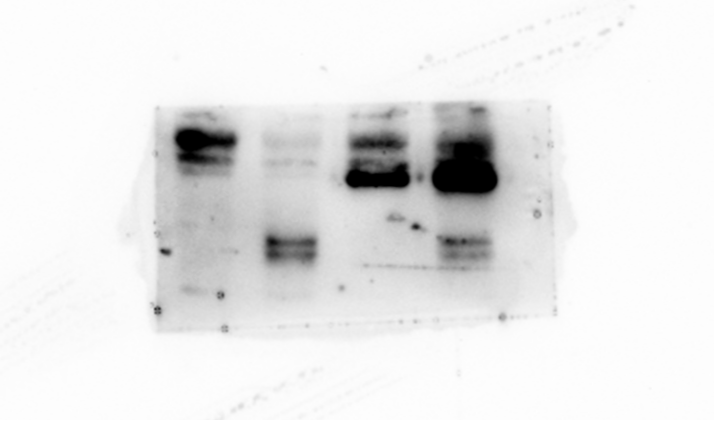

Supplement: Figure 4—figure supplement 2—source data 1. [file elife-73792-fig4-figsupp2-data1.zip › Figure 4-figure supplement 2-source data/2e/GSDME.tif]

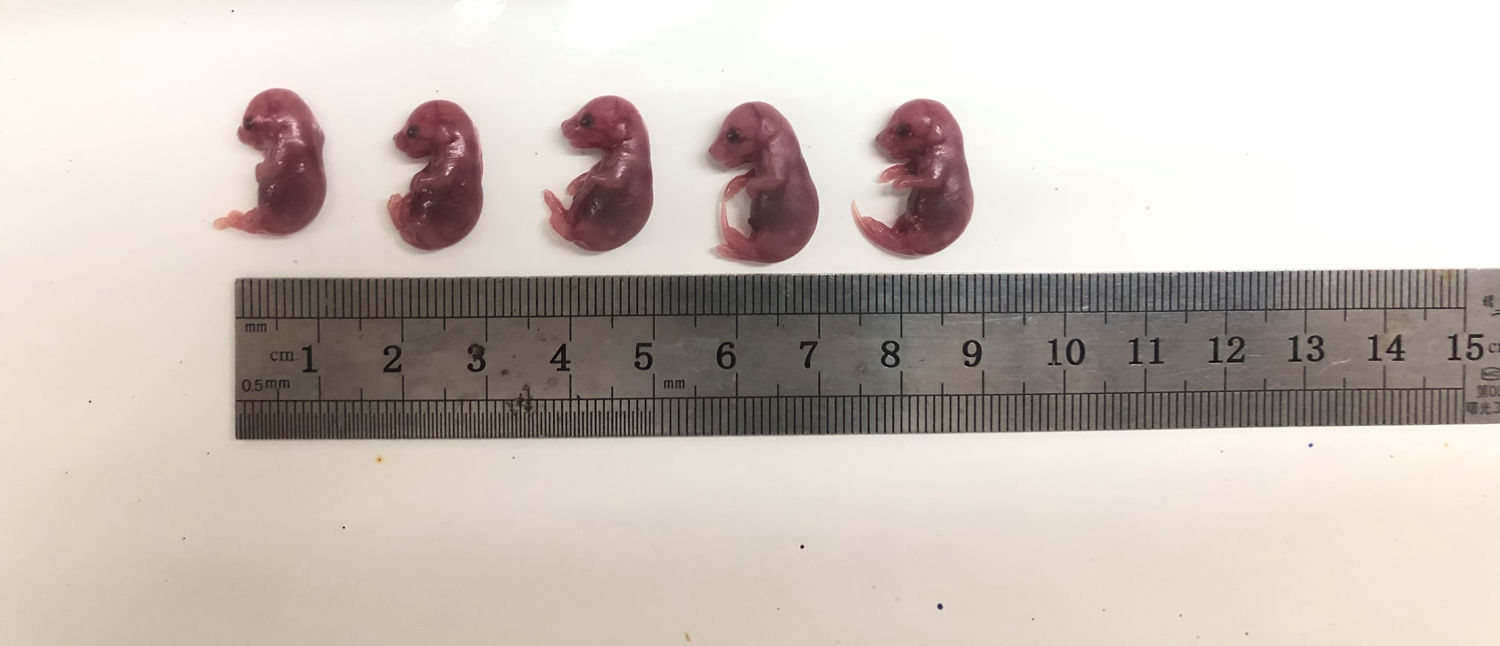

Supplement: Figure 5—source data 1. [file elife-73792-fig5-data1.zip › Figure 5-source data 1/Fig 5B/gsdme-ko mock.tif]

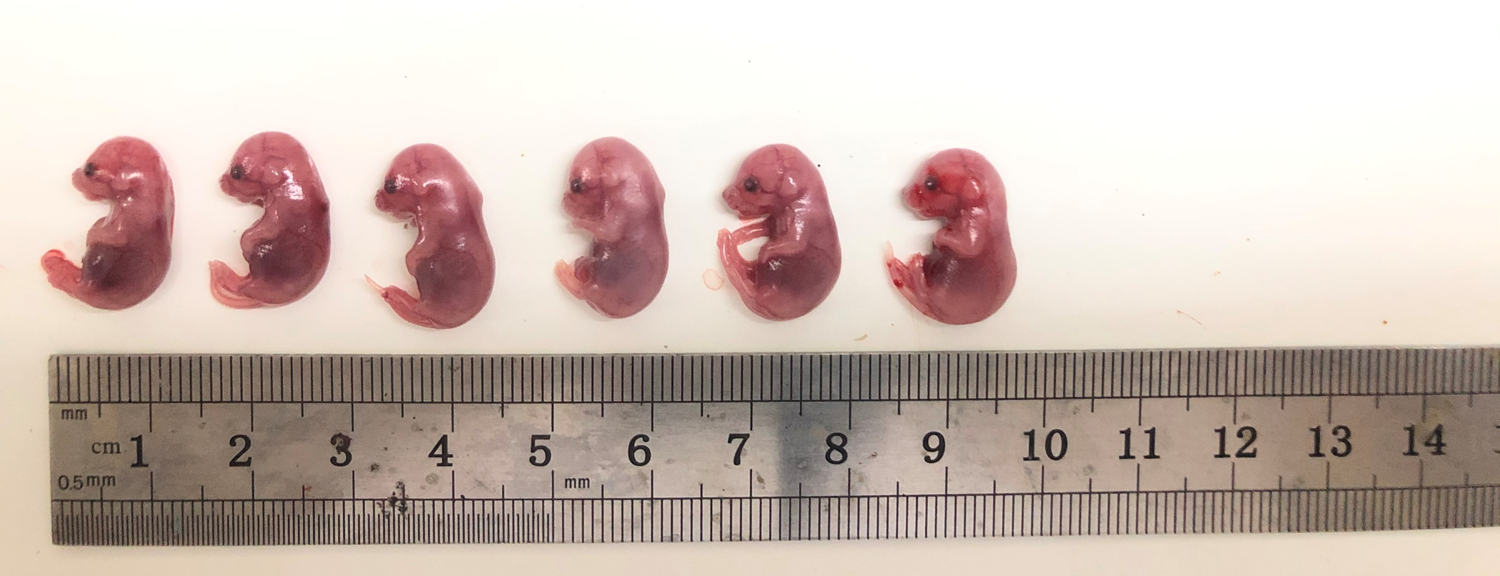

Supplement: Figure 5—source data 1. [file elife-73792-fig5-data1.zip › Figure 5-source data 1/Fig 5B/gsdme-ko zikv 1.tif]

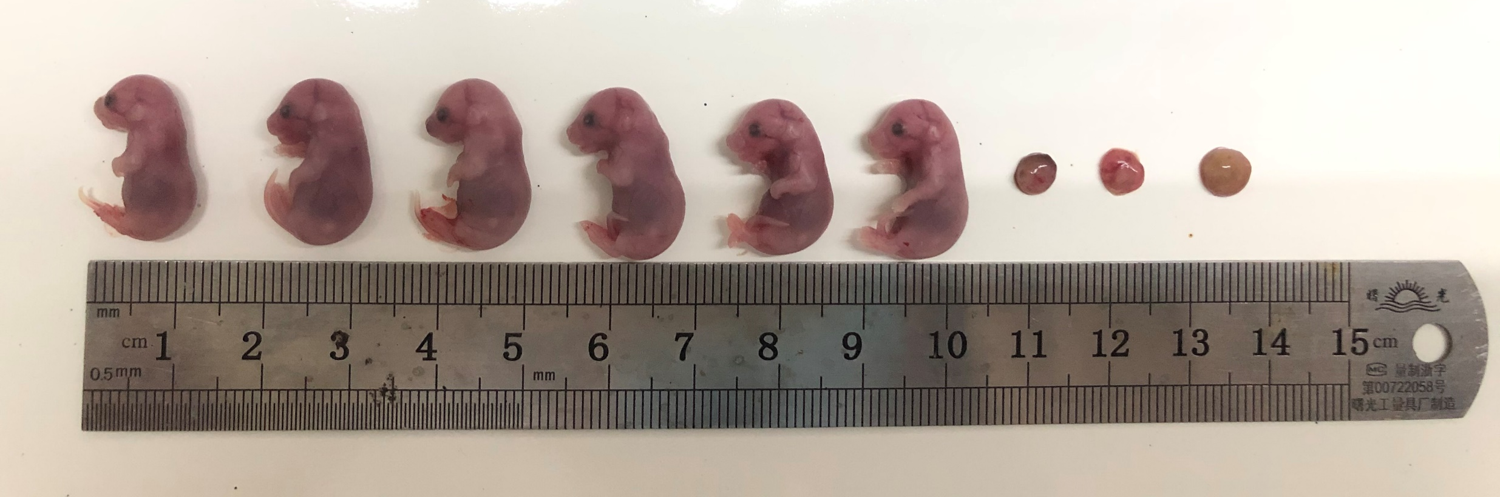

Supplement: Figure 5—source data 1. [file elife-73792-fig5-data1.zip › Figure 5-source data 1/Fig 5B/gsdme-ko zikv 2.tif]

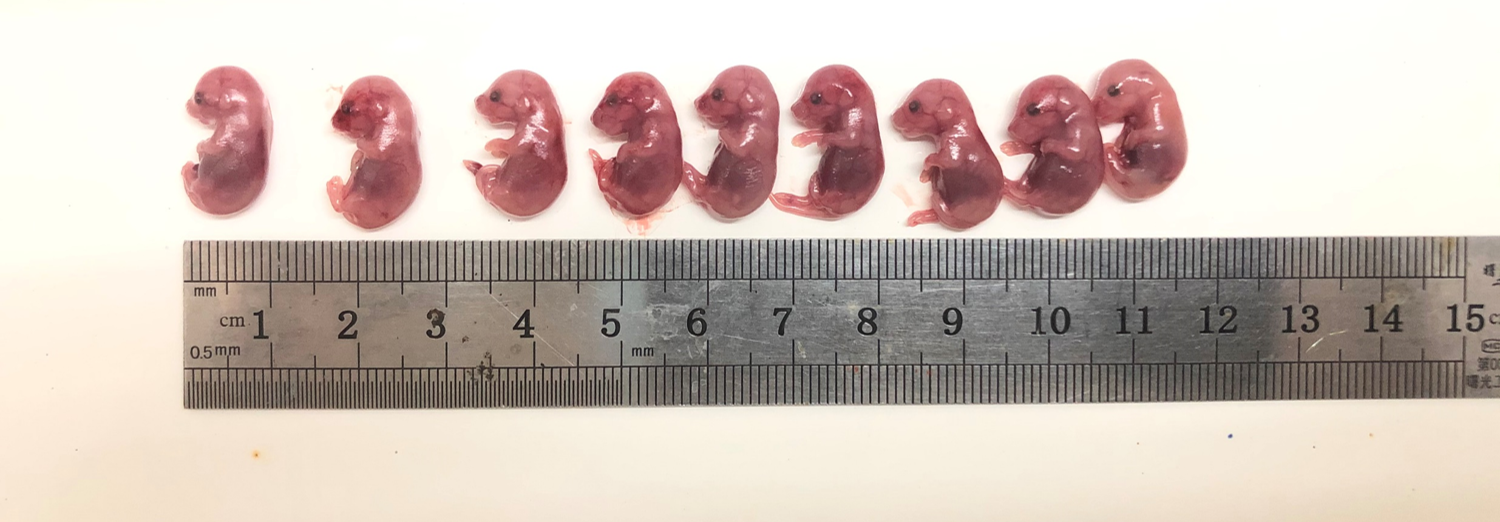

Supplement: Figure 5—source data 1. [file elife-73792-fig5-data1.zip › Figure 5-source data 1/Fig 5B/wt mock.tif]

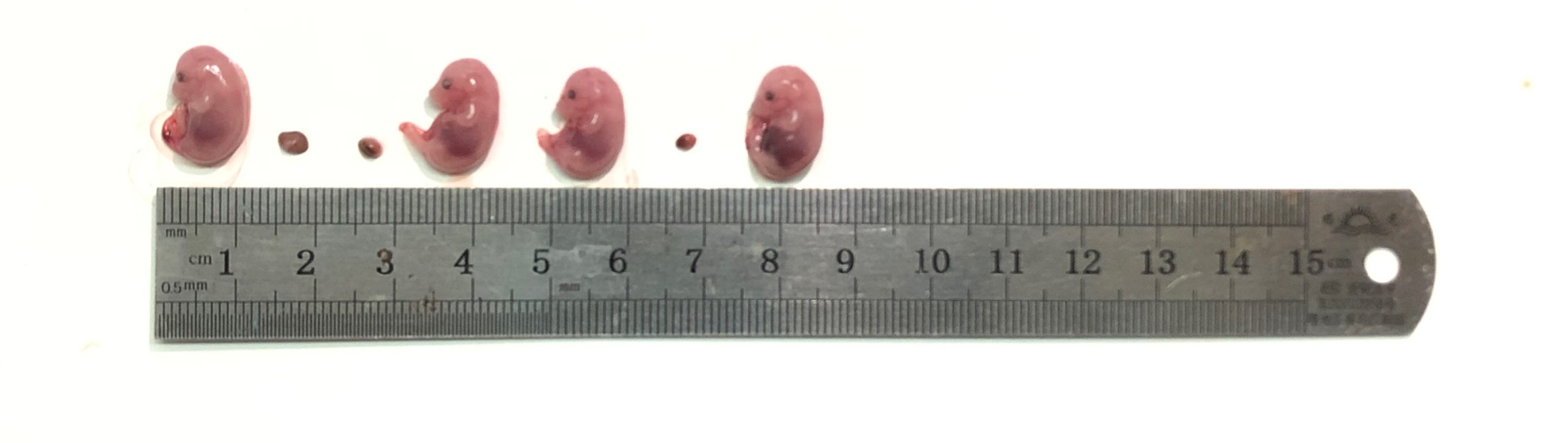

Supplement: Figure 5—source data 1. [file elife-73792-fig5-data1.zip › Figure 5-source data 1/Fig 5B/wt zikv 1.tif]

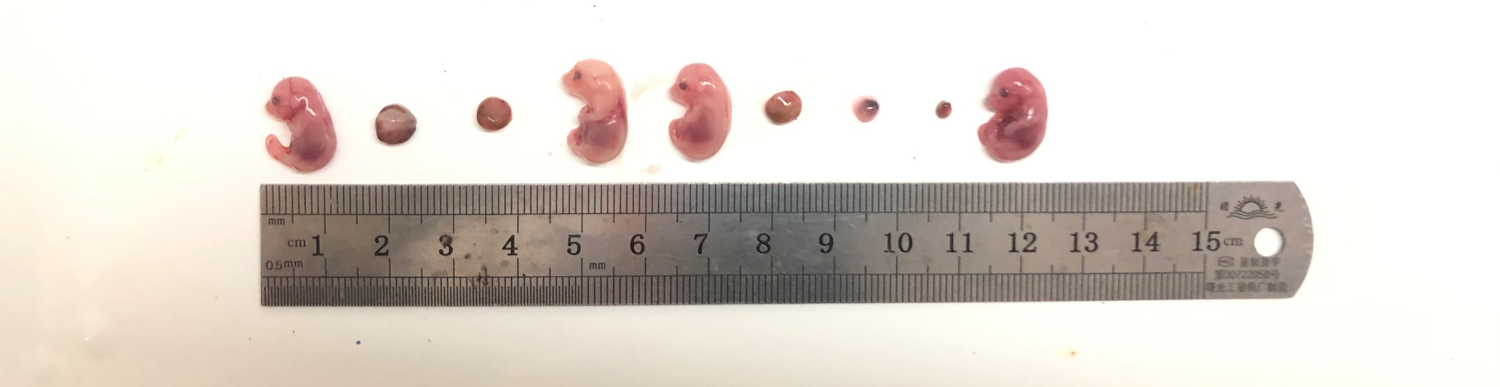

Supplement: Figure 5—source data 1. [file elife-73792-fig5-data1.zip › Figure 5-source data 1/Fig 5B/wt zikv 2.tif]

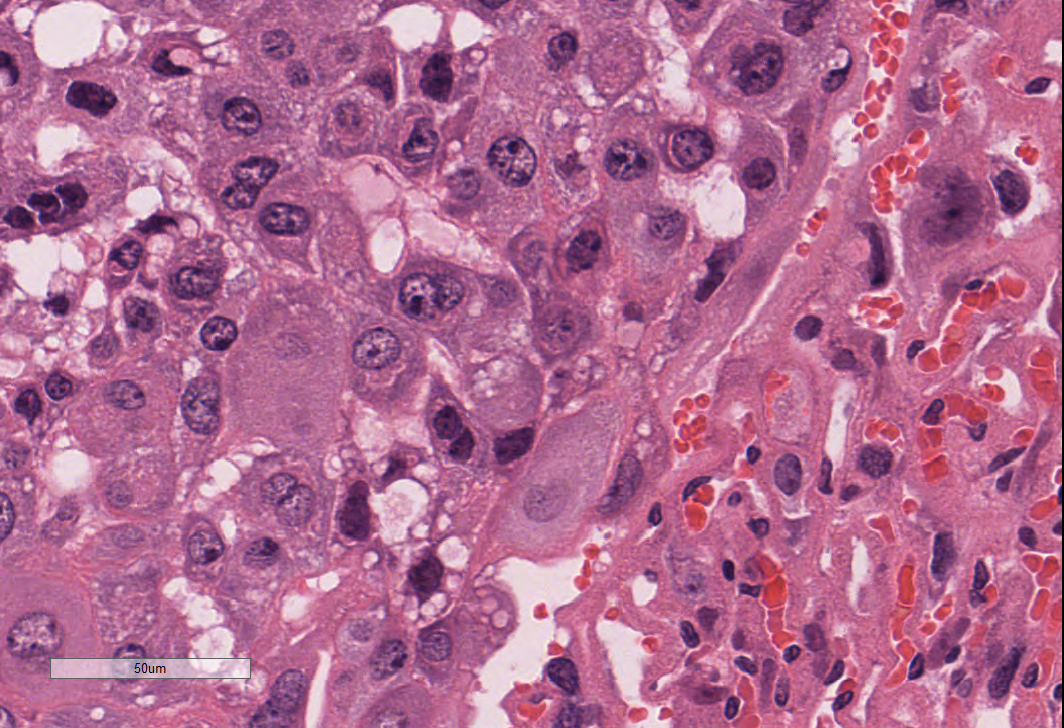

Supplement: Figure 5—source data 1. [file elife-73792-fig5-data1.zip › Figure 5-source data 1/Fig 5D/eko MOCK junctional zone.tif]

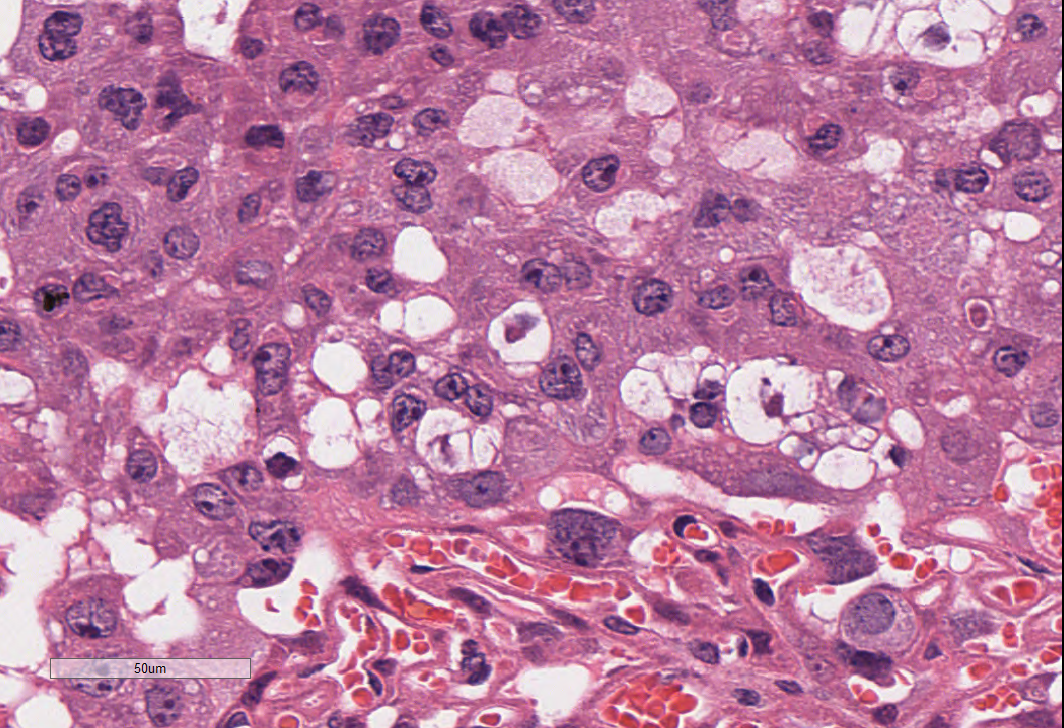

Supplement: Figure 5—source data 1. [file elife-73792-fig5-data1.zip › Figure 5-source data 1/Fig 5D/eko zikv junctional zone.tif]

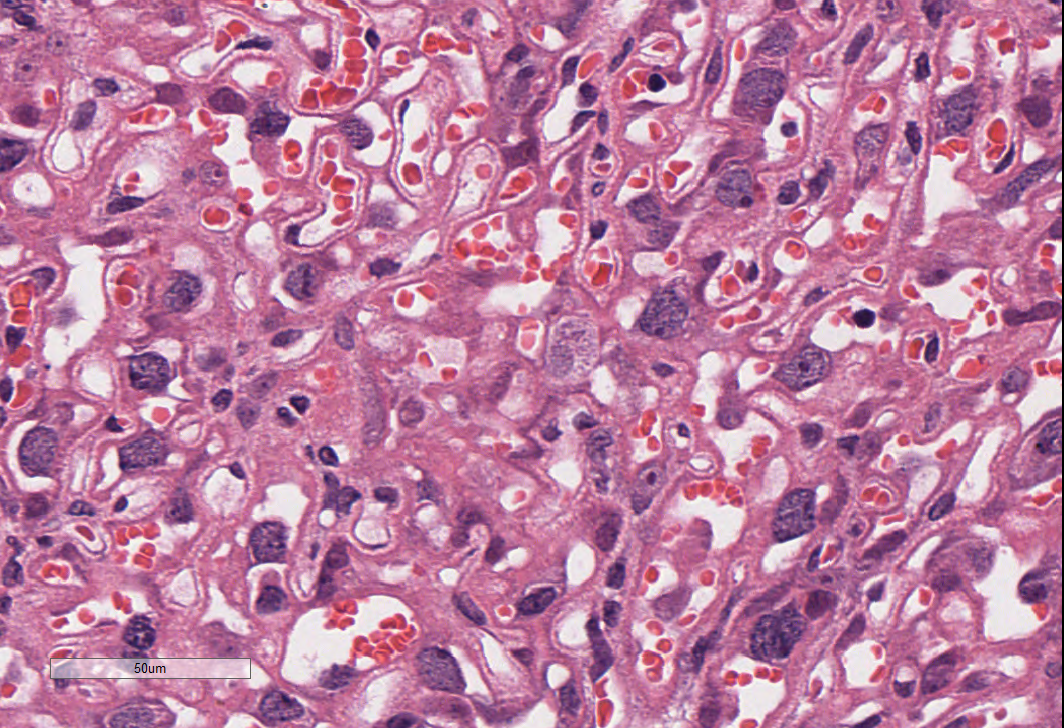

Supplement: Figure 5—source data 1. [file elife-73792-fig5-data1.zip › Figure 5-source data 1/Fig 5D/gsdme-ko mock placental labyrinth.tif]

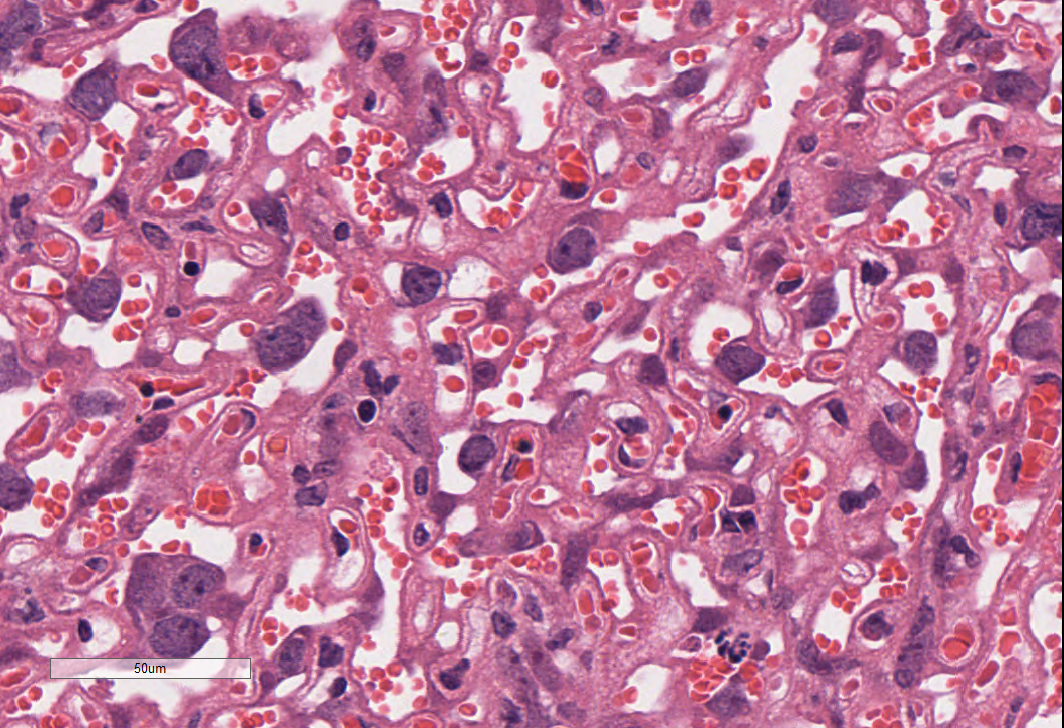

Supplement: Figure 5—source data 1. [file elife-73792-fig5-data1.zip › Figure 5-source data 1/Fig 5D/gsdme-ko zikv placental labyrinth 1.tif]

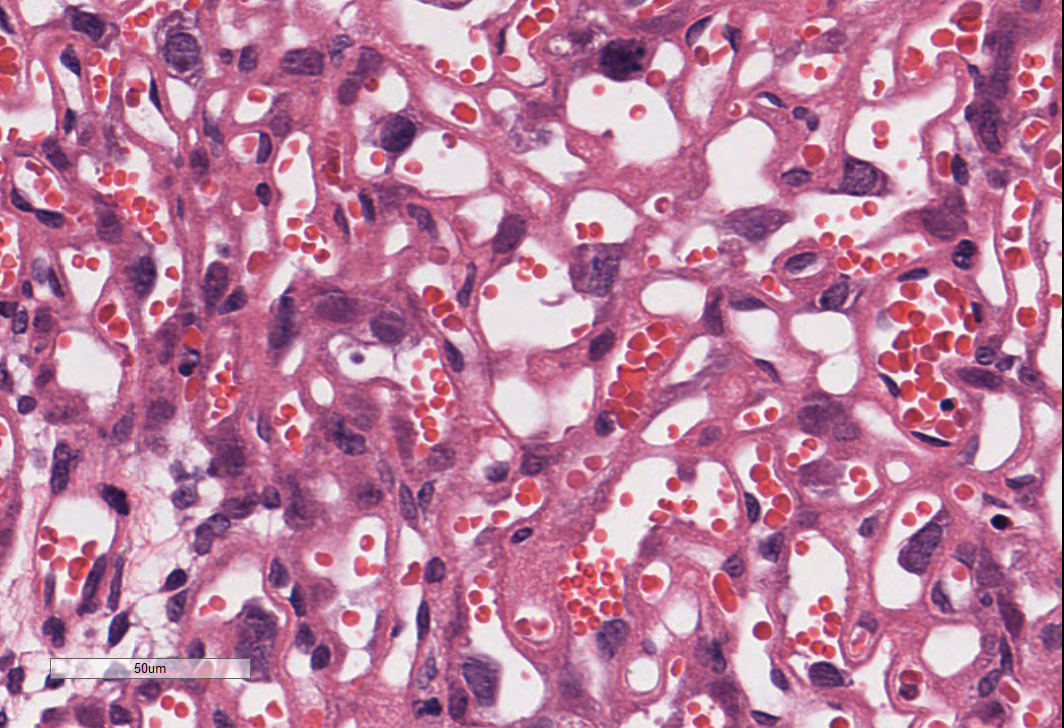

Supplement: Figure 5—source data 1. [file elife-73792-fig5-data1.zip › Figure 5-source data 1/Fig 5D/gsdme-ko zikv placental labyrinth 2.tif]

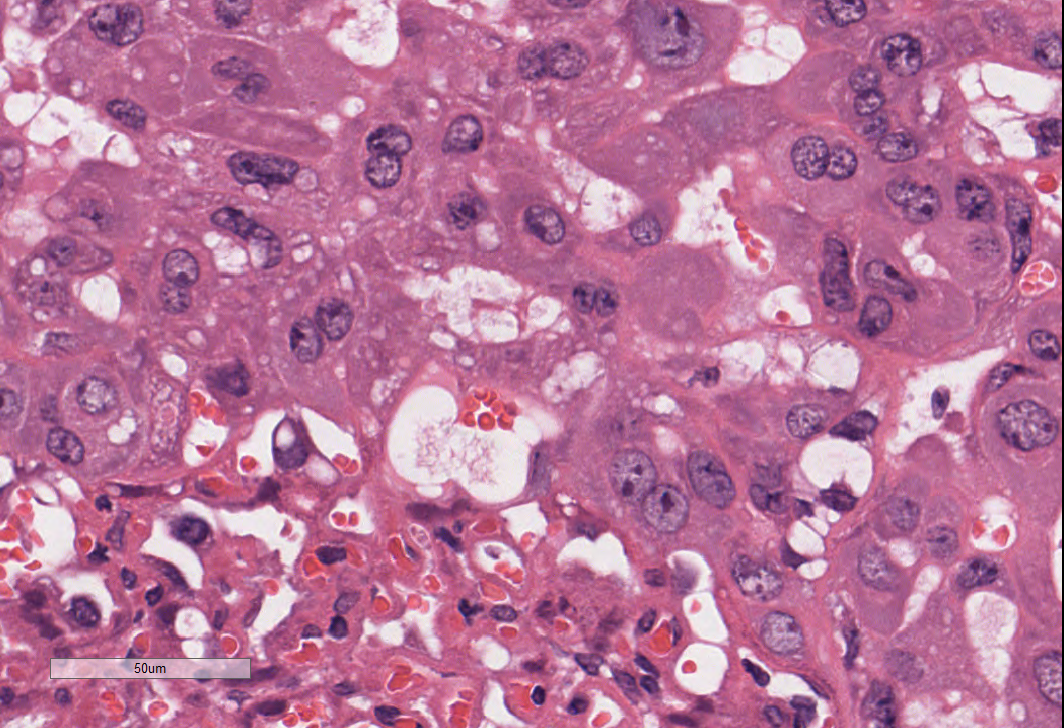

Supplement: Figure 5—source data 1. [file elife-73792-fig5-data1.zip › Figure 5-source data 1/Fig 5D/wt MOCK junctional zone.tif]

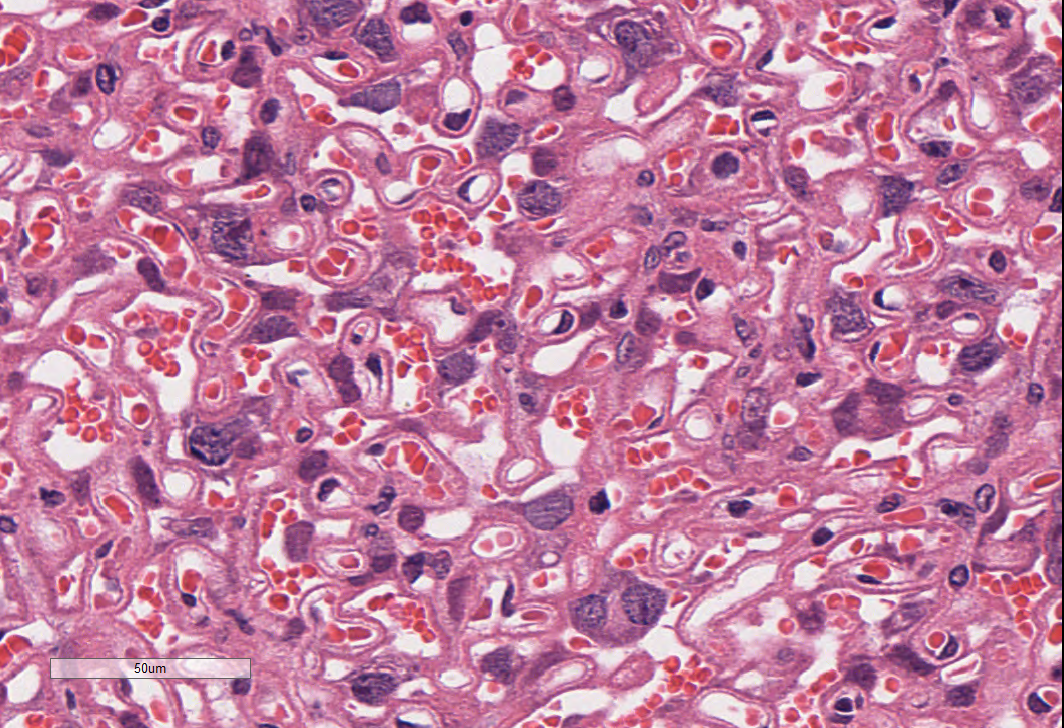

Supplement: Figure 5—source data 1. [file elife-73792-fig5-data1.zip › Figure 5-source data 1/Fig 5D/wt mock placental labryinth.tif]

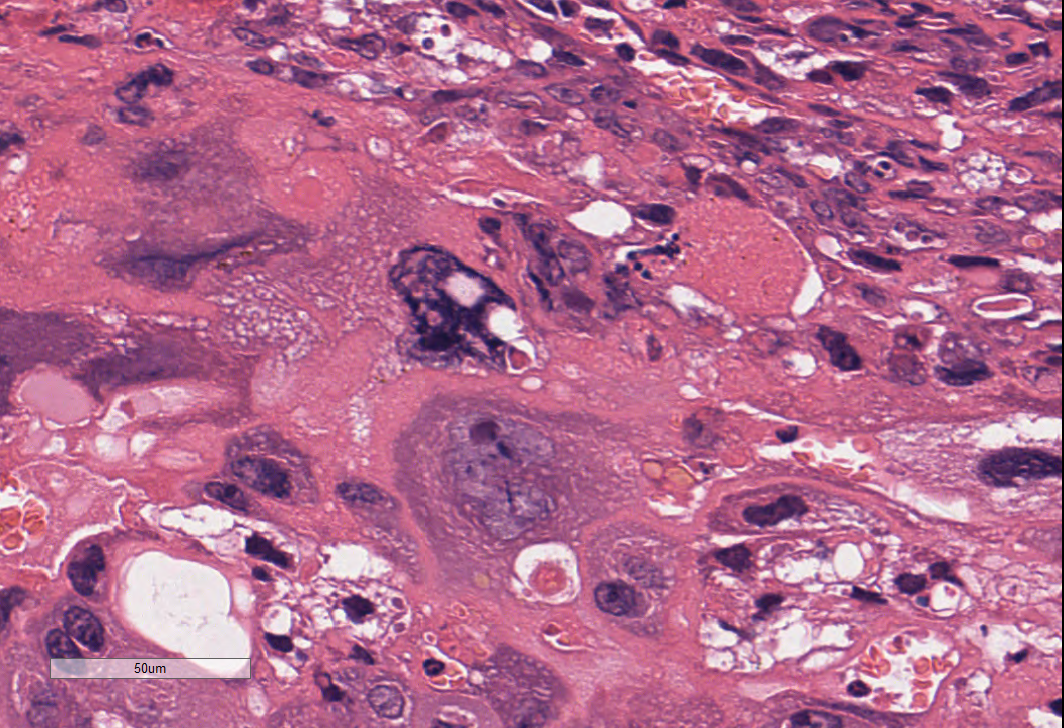

Supplement: Figure 5—source data 1. [file elife-73792-fig5-data1.zip › Figure 5-source data 1/Fig 5D/wt zikv junctional zone.tif]

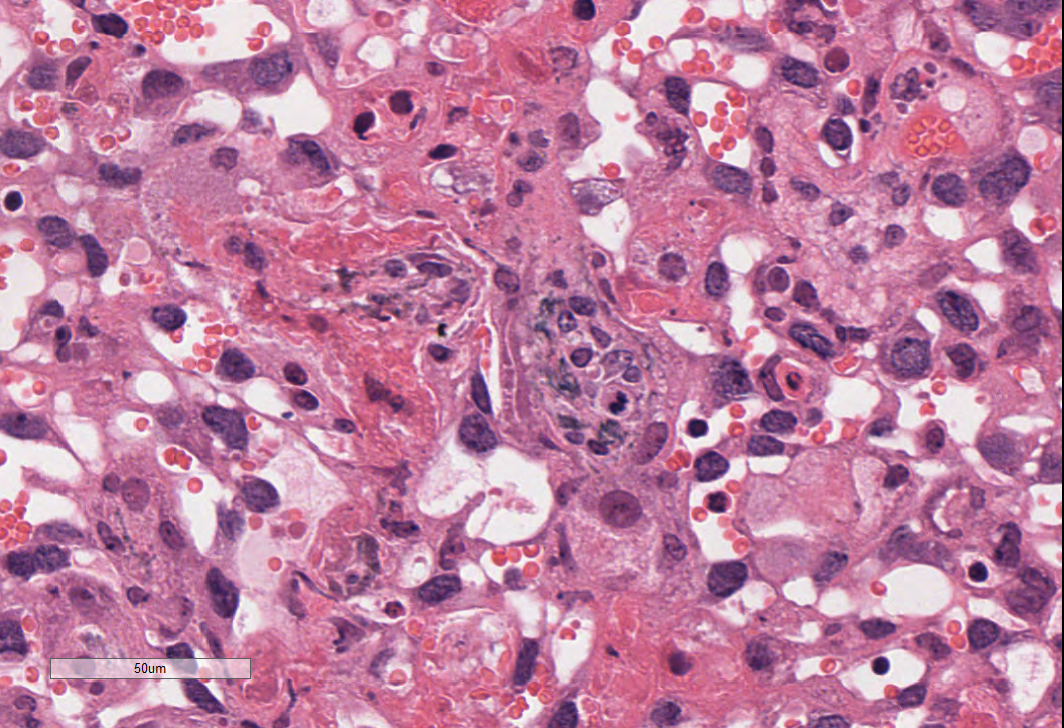

Supplement: Figure 5—source data 1. [file elife-73792-fig5-data1.zip › Figure 5-source data 1/Fig 5D/wt zikv placental labryinth 1.tif]

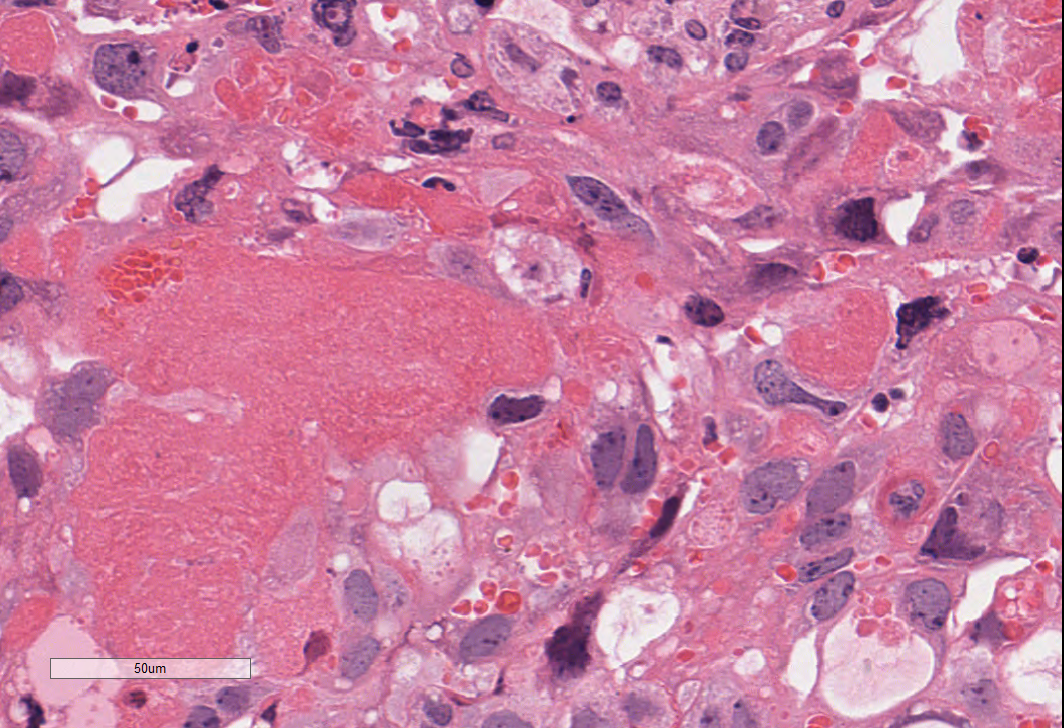

Supplement: Figure 5—source data 1. [file elife-73792-fig5-data1.zip › Figure 5-source data 1/Fig 5D/wt zikv placental labryinth 2.tif]

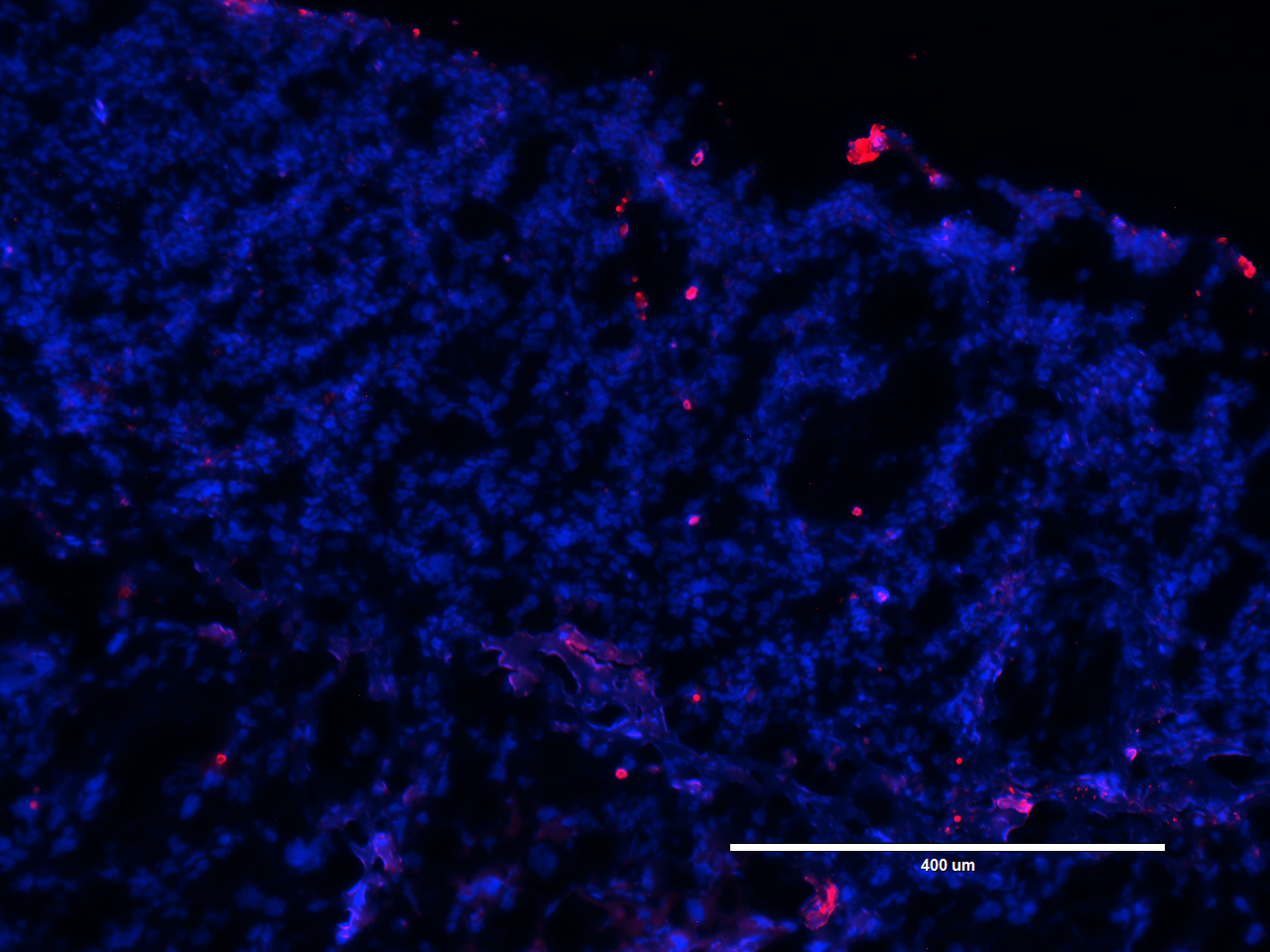

Supplement: Figure 5—source data 1. [file elife-73792-fig5-data1.zip › Figure 5-source data 1/Fig 5E/6nc.tif]

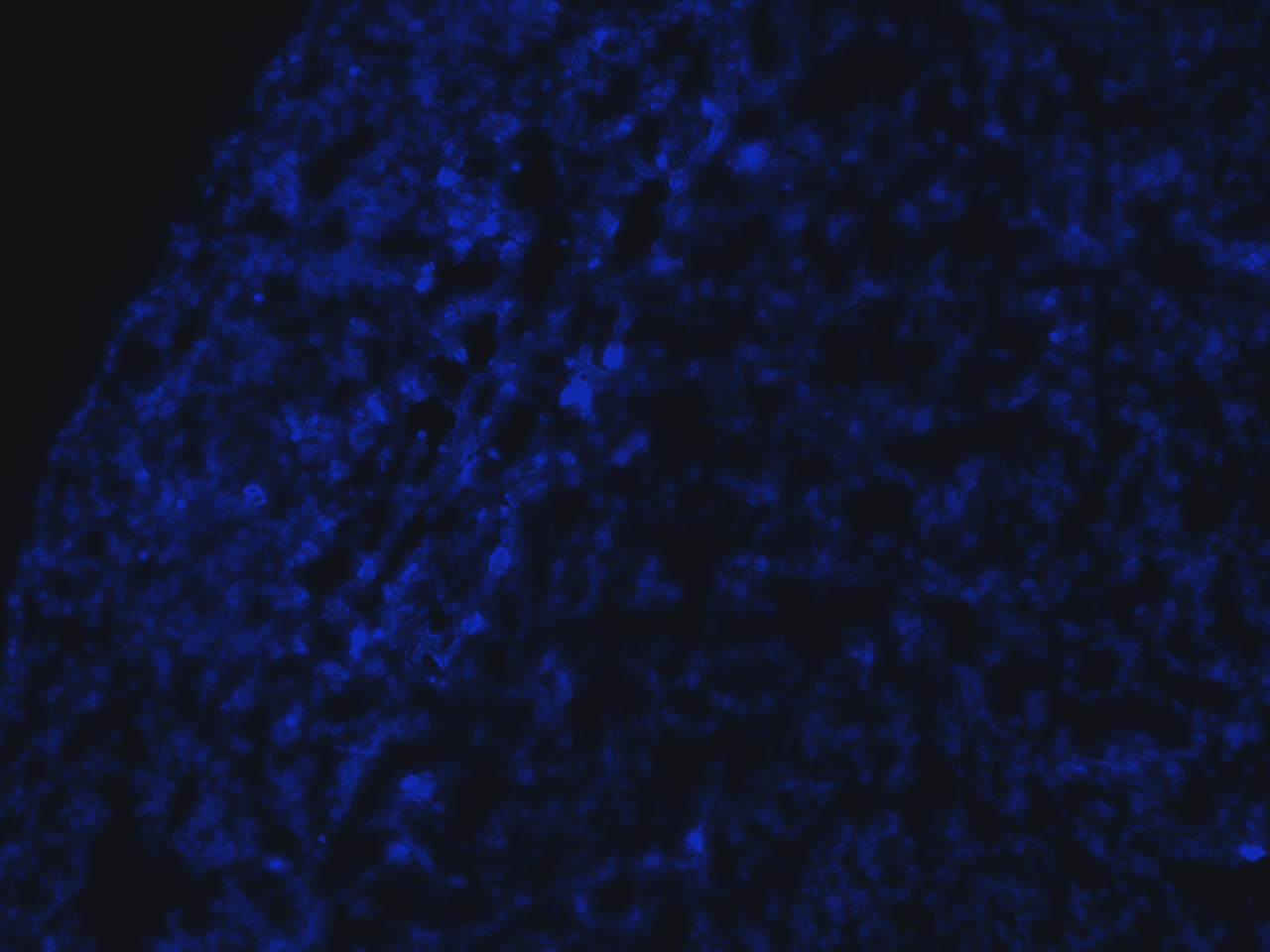

Supplement: Figure 5—source data 1. [file elife-73792-fig5-data1.zip › Figure 5-source data 1/Fig 5E/gsdme-ko mock hoechst.tif]

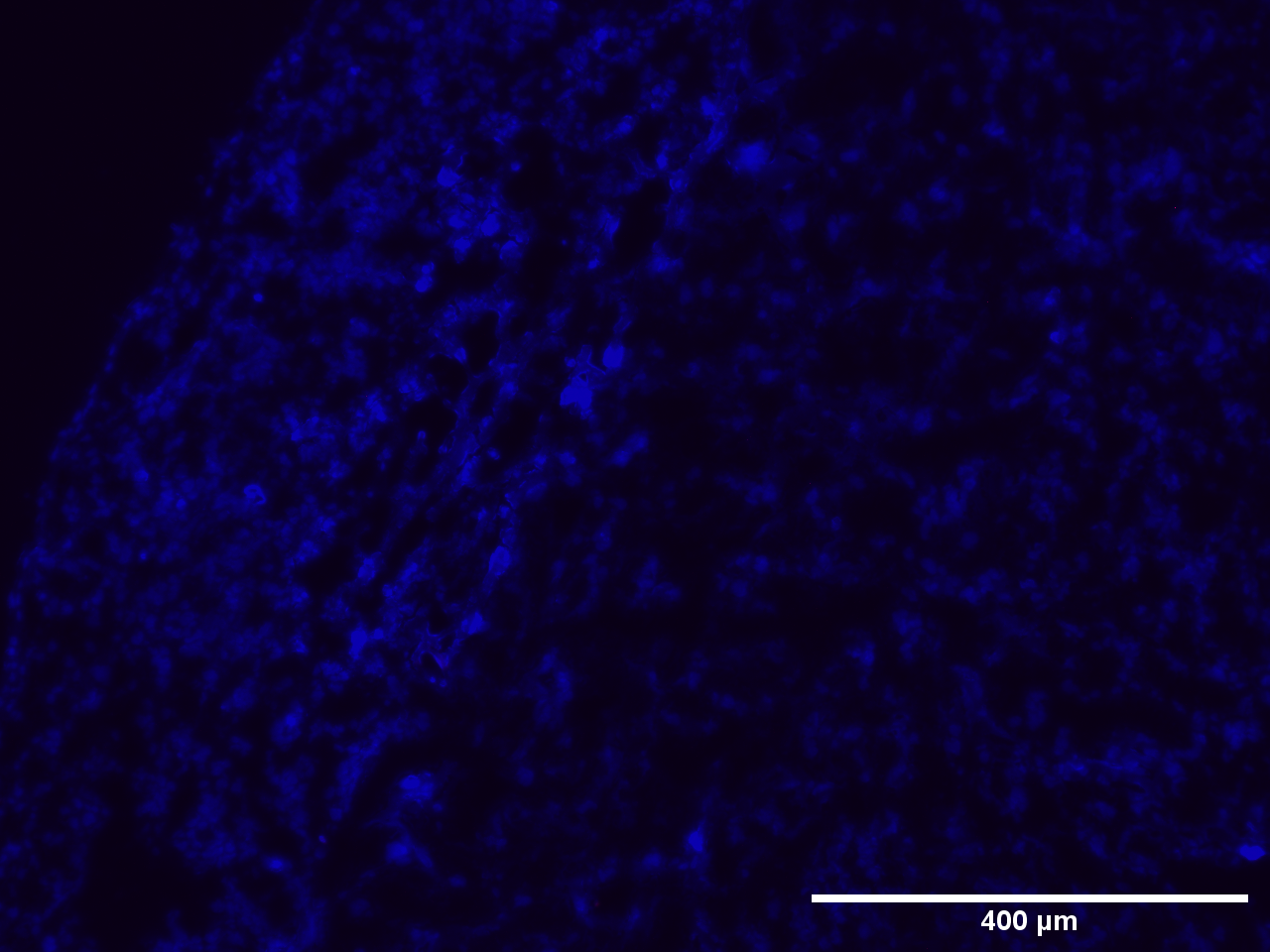

Supplement: Figure 5—source data 1. [file elife-73792-fig5-data1.zip › Figure 5-source data 1/Fig 5E/gsdme-ko mock merge.tif]

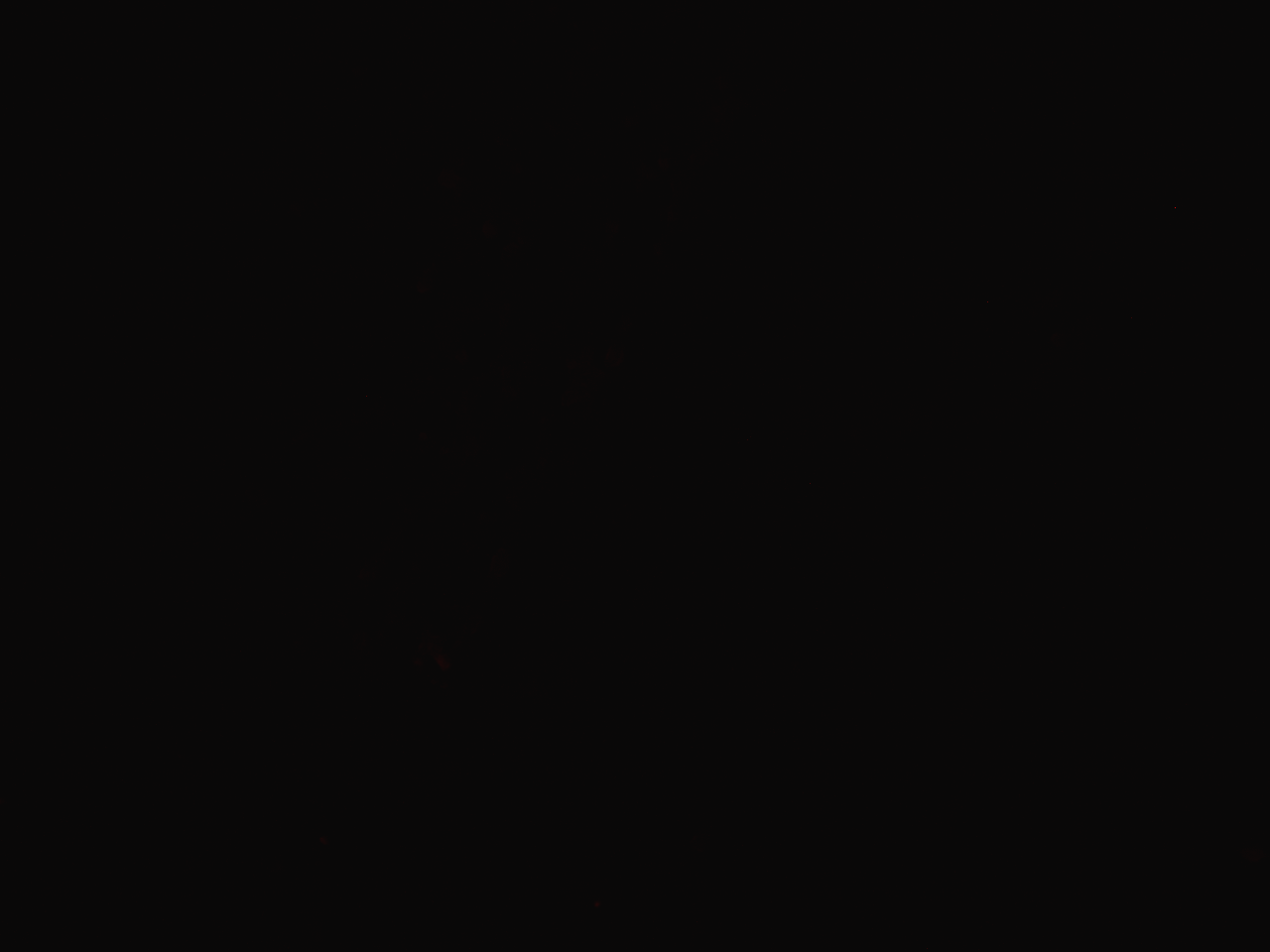

Supplement: Figure 5—source data 1. [file elife-73792-fig5-data1.zip › Figure 5-source data 1/Fig 5E/gsdme-ko mock pi.tif]

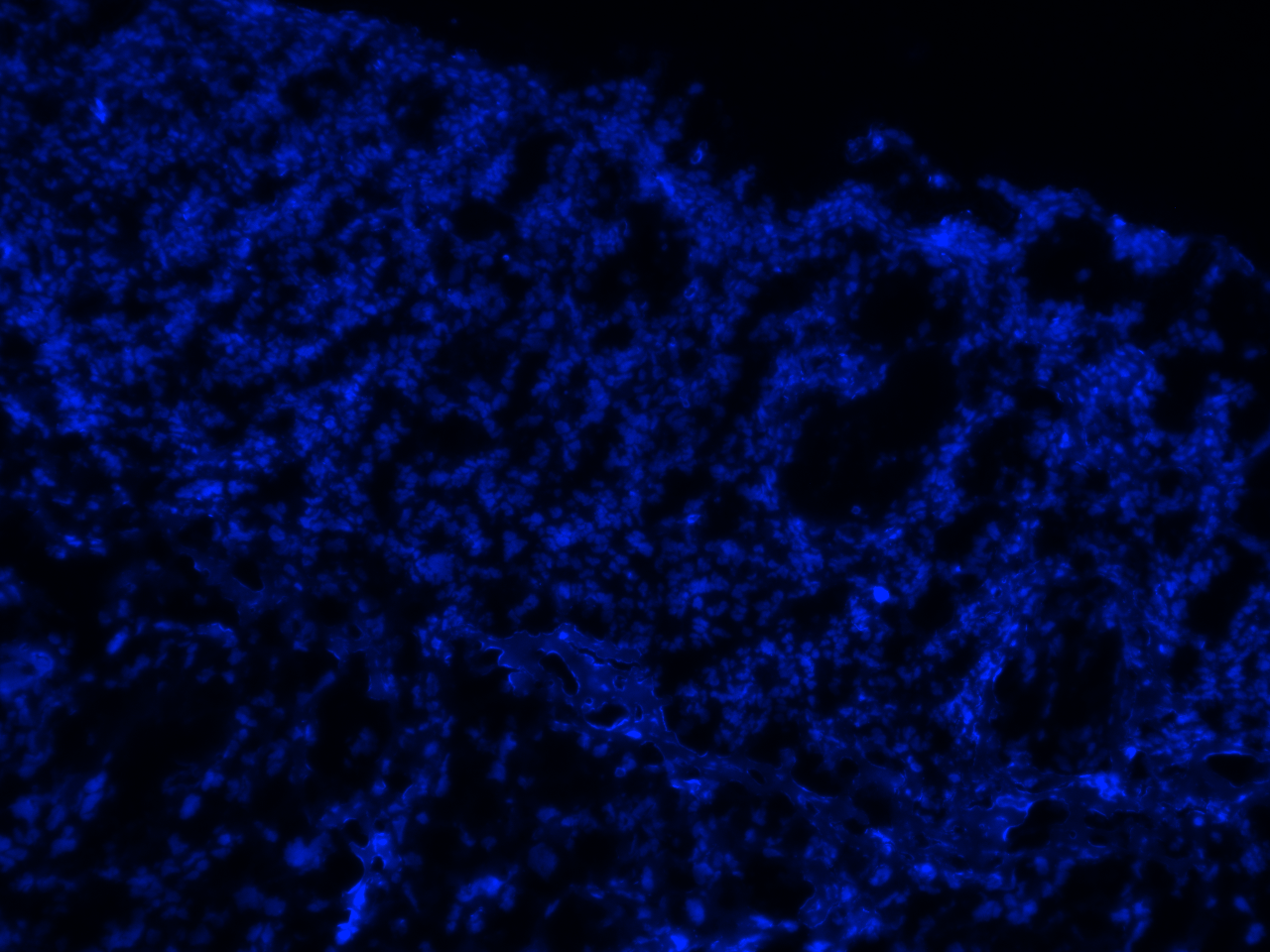

Supplement: Figure 5—source data 1. [file elife-73792-fig5-data1.zip › Figure 5-source data 1/Fig 5E/gsdme-ko zikv hoechst.tif]

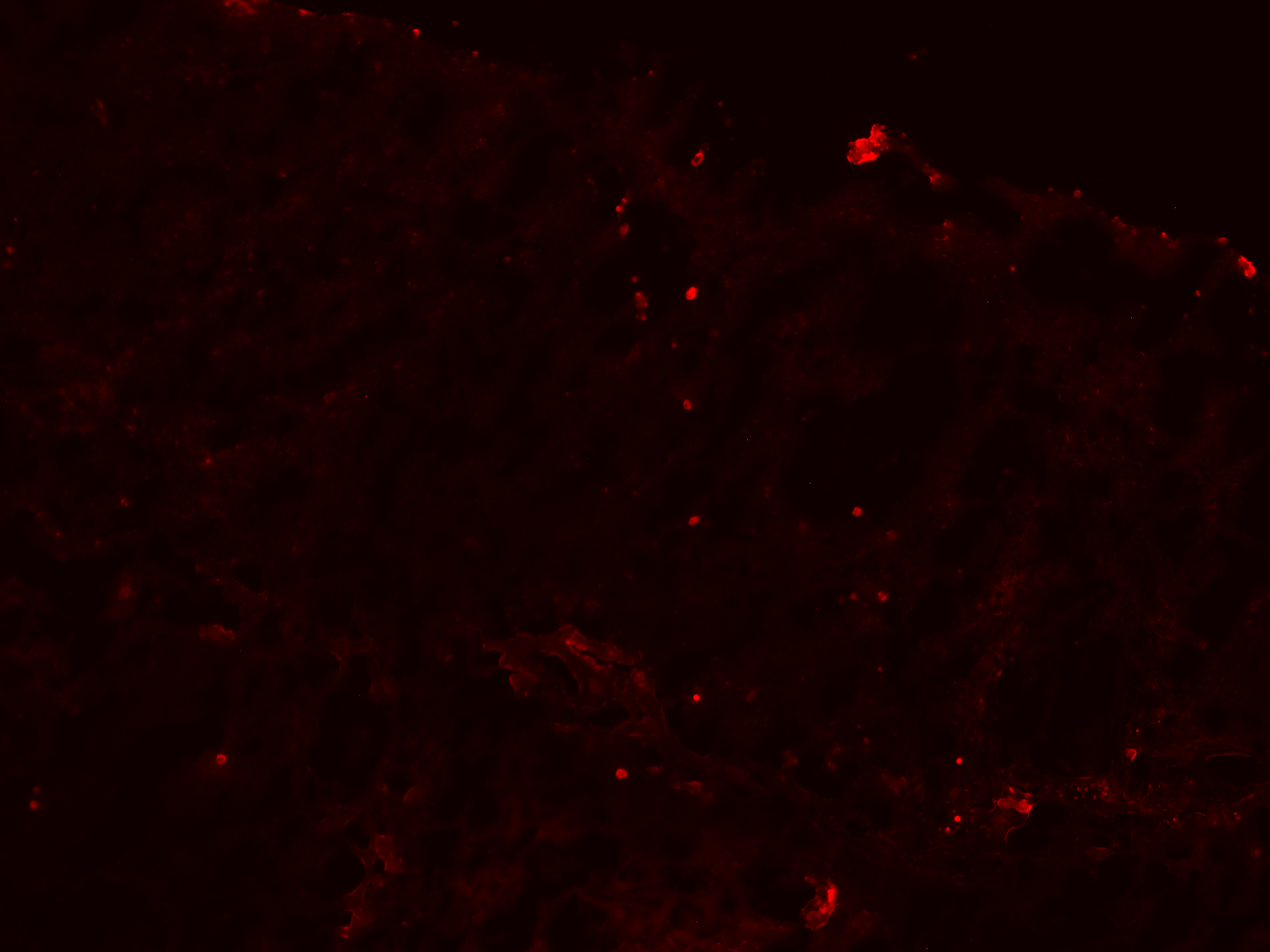

Supplement: Figure 5—source data 1. [file elife-73792-fig5-data1.zip › Figure 5-source data 1/Fig 5E/gsdme-ko zikv PI.tif]

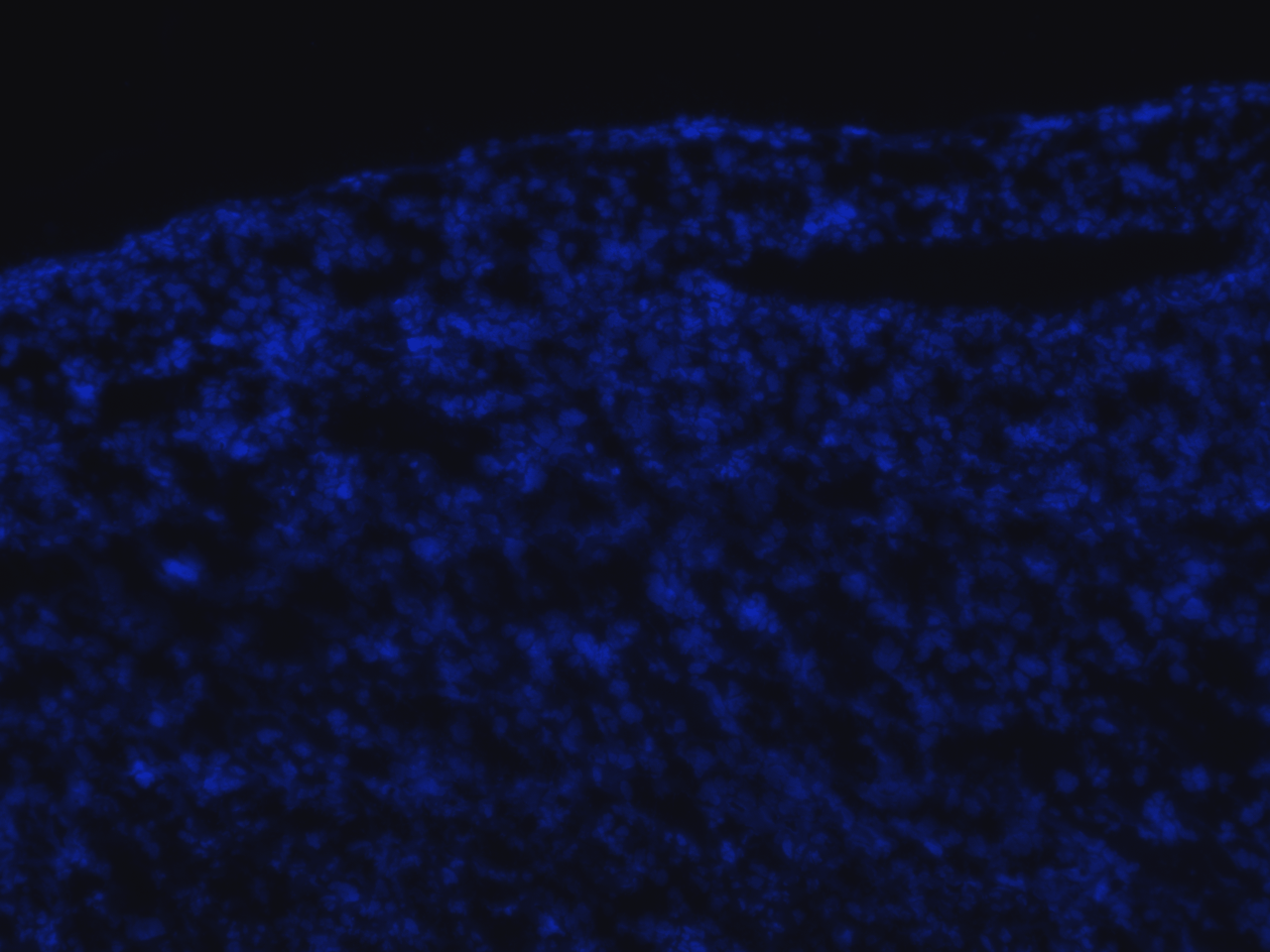

Supplement: Figure 5—source data 1. [file elife-73792-fig5-data1.zip › Figure 5-source data 1/Fig 5E/wt mock hoechst.tif]

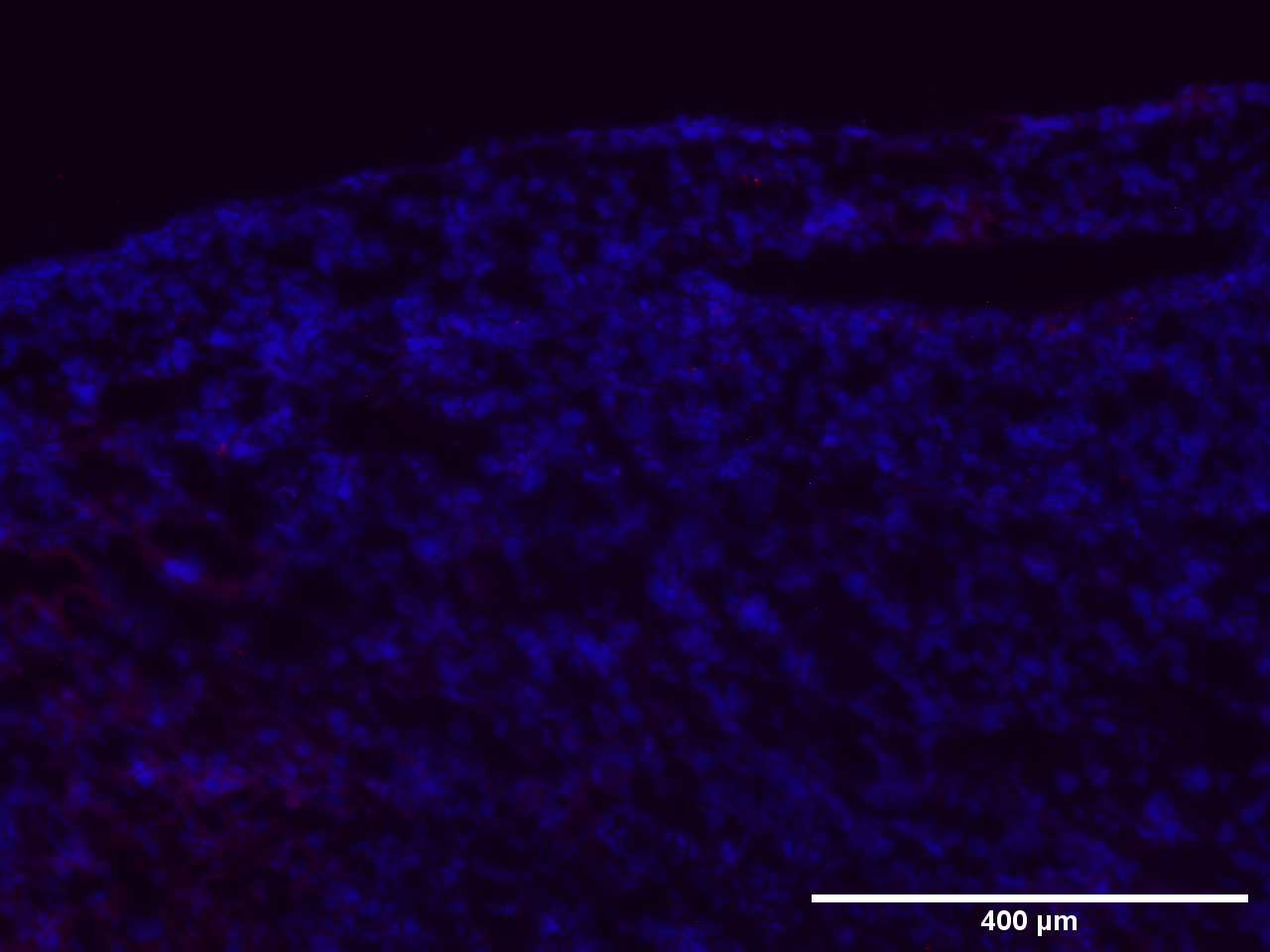

Supplement: Figure 5—source data 1. [file elife-73792-fig5-data1.zip › Figure 5-source data 1/Fig 5E/wt mock merge.tif]

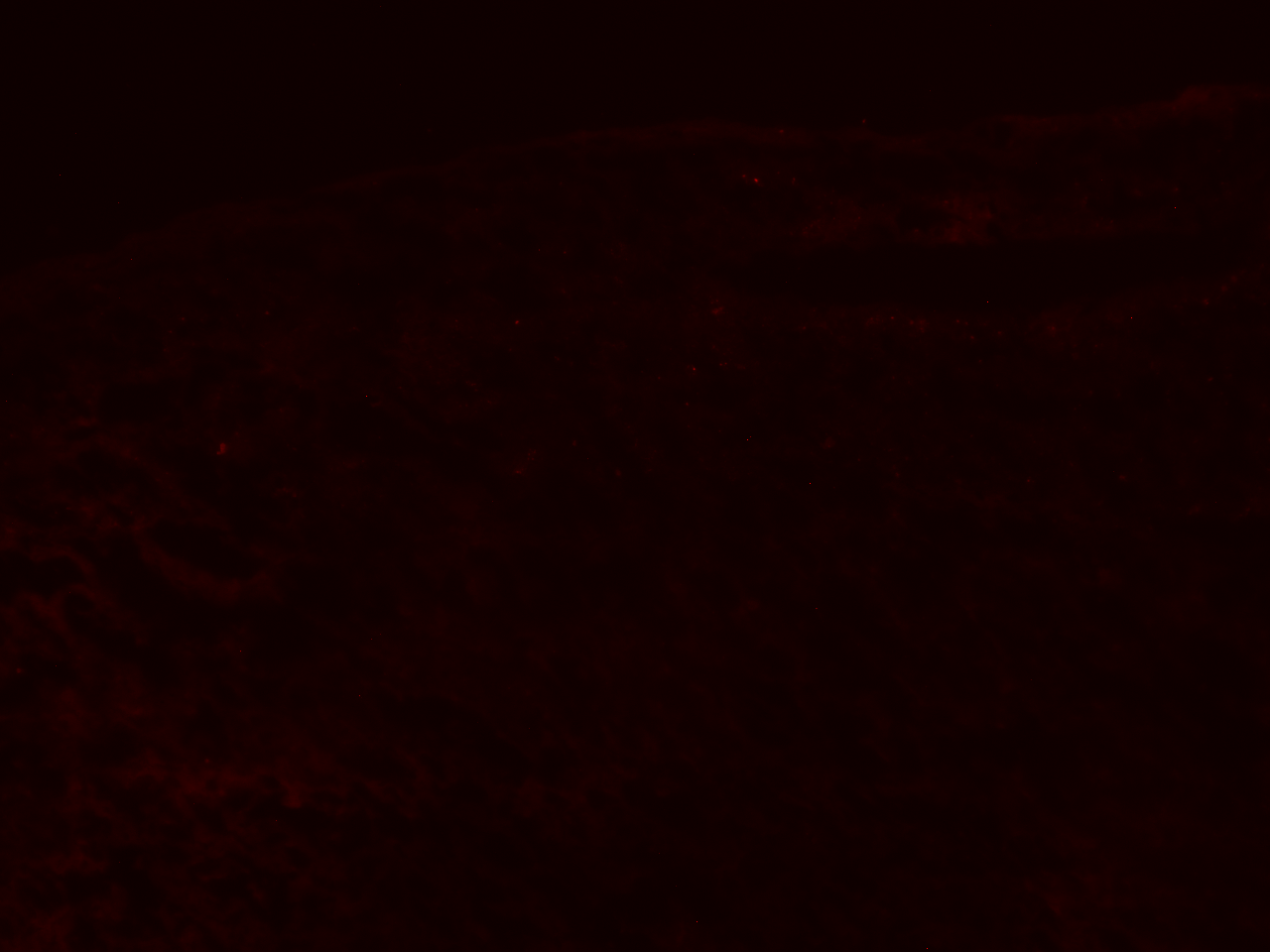

Supplement: Figure 5—source data 1. [file elife-73792-fig5-data1.zip › Figure 5-source data 1/Fig 5E/wt mock pi.tif]

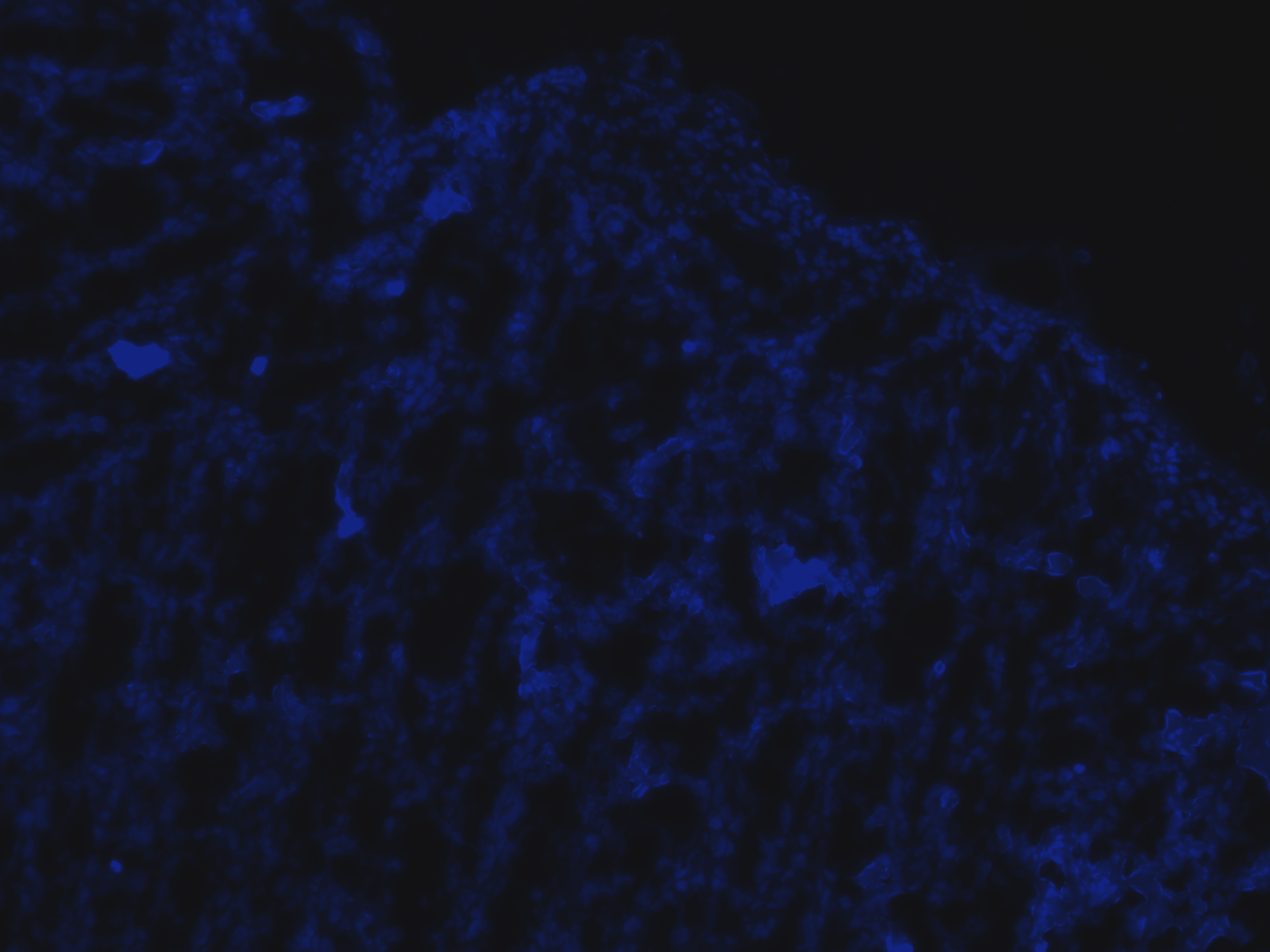

Supplement: Figure 5—source data 1. [file elife-73792-fig5-data1.zip › Figure 5-source data 1/Fig 5E/wt zikv hoechst.tif]

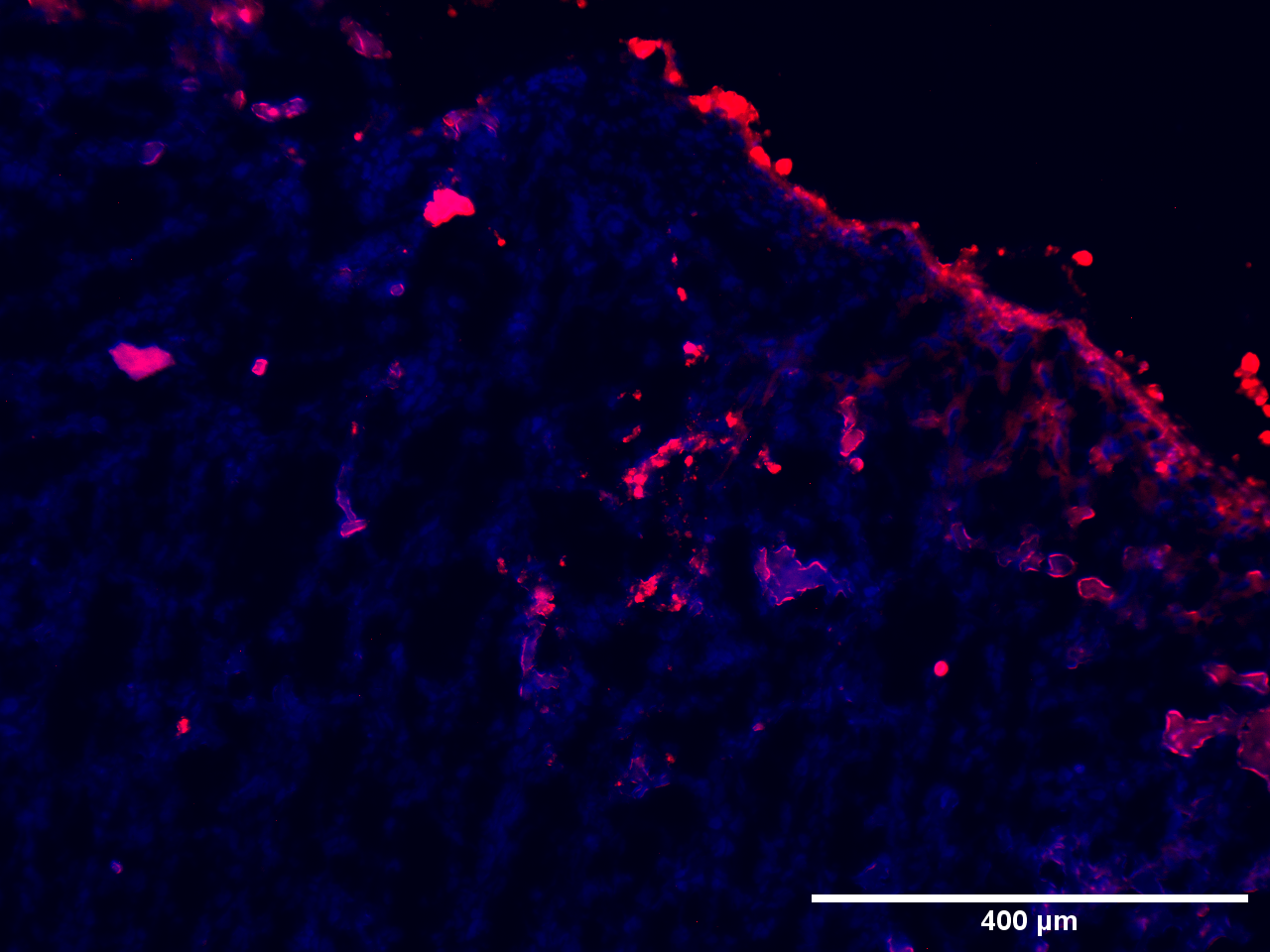

Supplement: Figure 5—source data 1. [file elife-73792-fig5-data1.zip › Figure 5-source data 1/Fig 5E/wt zikv merge.tif]

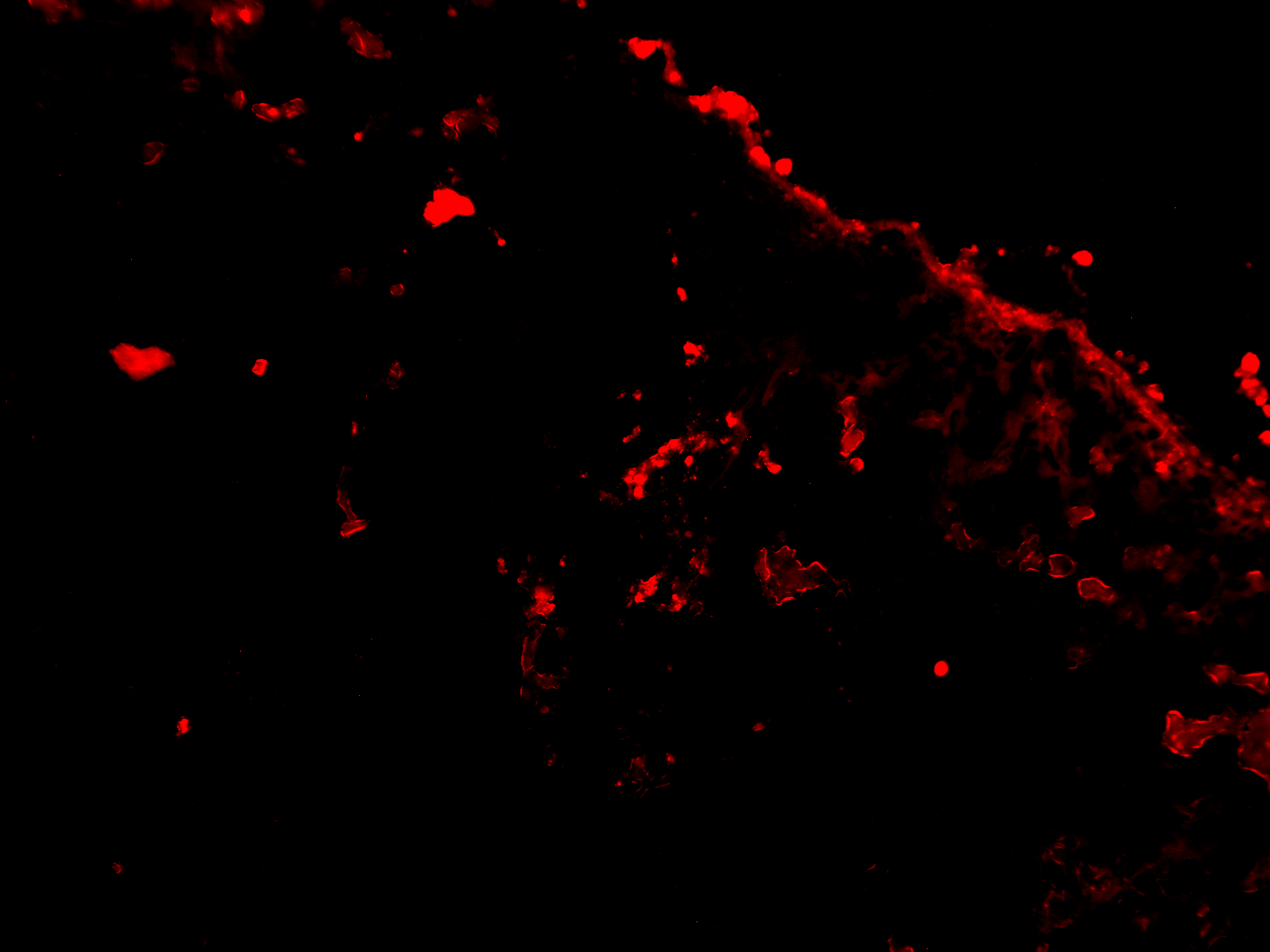

Supplement: Figure 5—source data 1. [file elife-73792-fig5-data1.zip › Figure 5-source data 1/Fig 5E/wt zikv pi.tif]

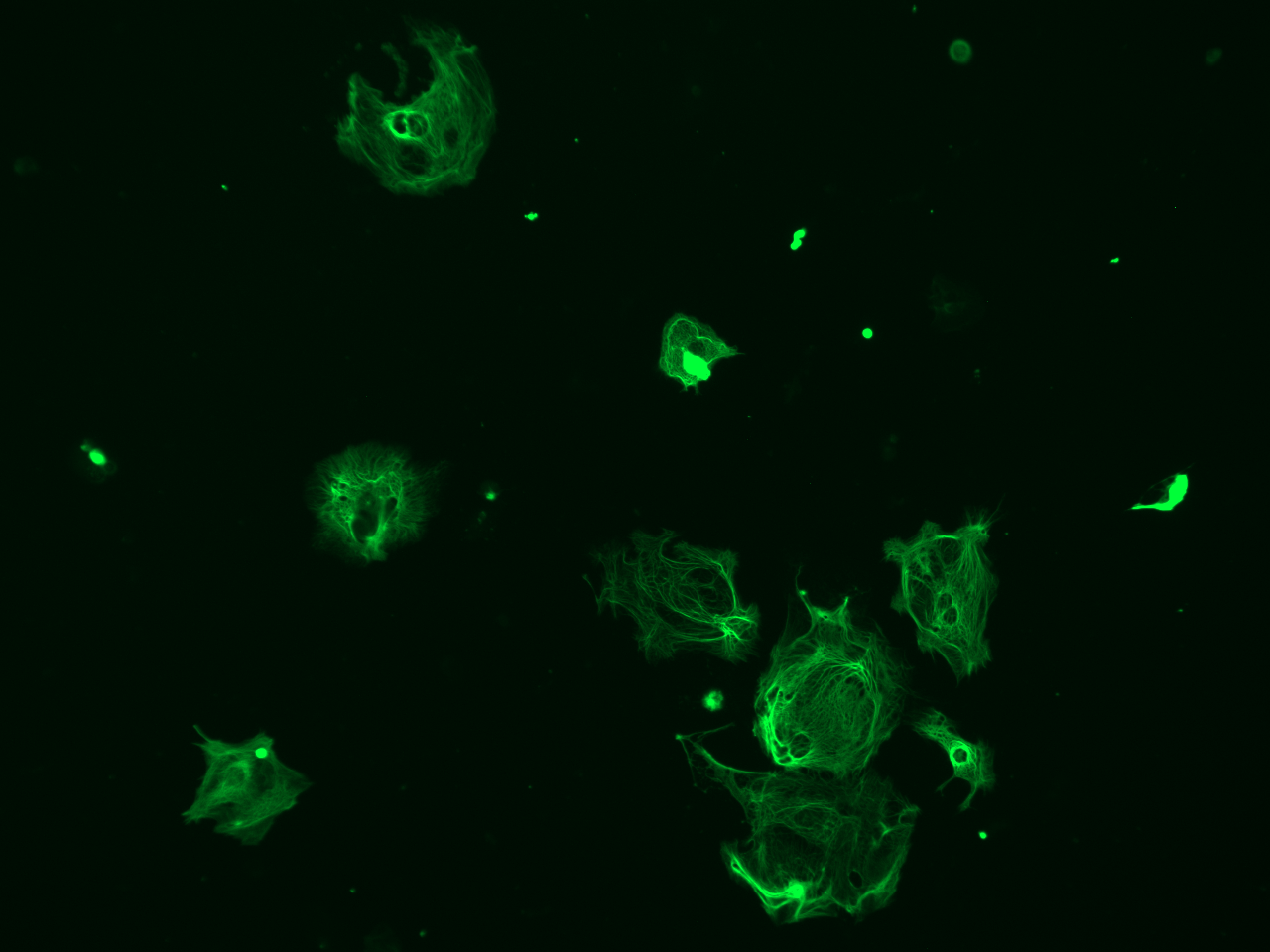

Supplement: Figure 5—figure supplement 1—source data 1. [file elife-73792-fig5-figsupp1-data1.zip › Figure 5-figure supplement 1-source data/1a/CK7.tif]

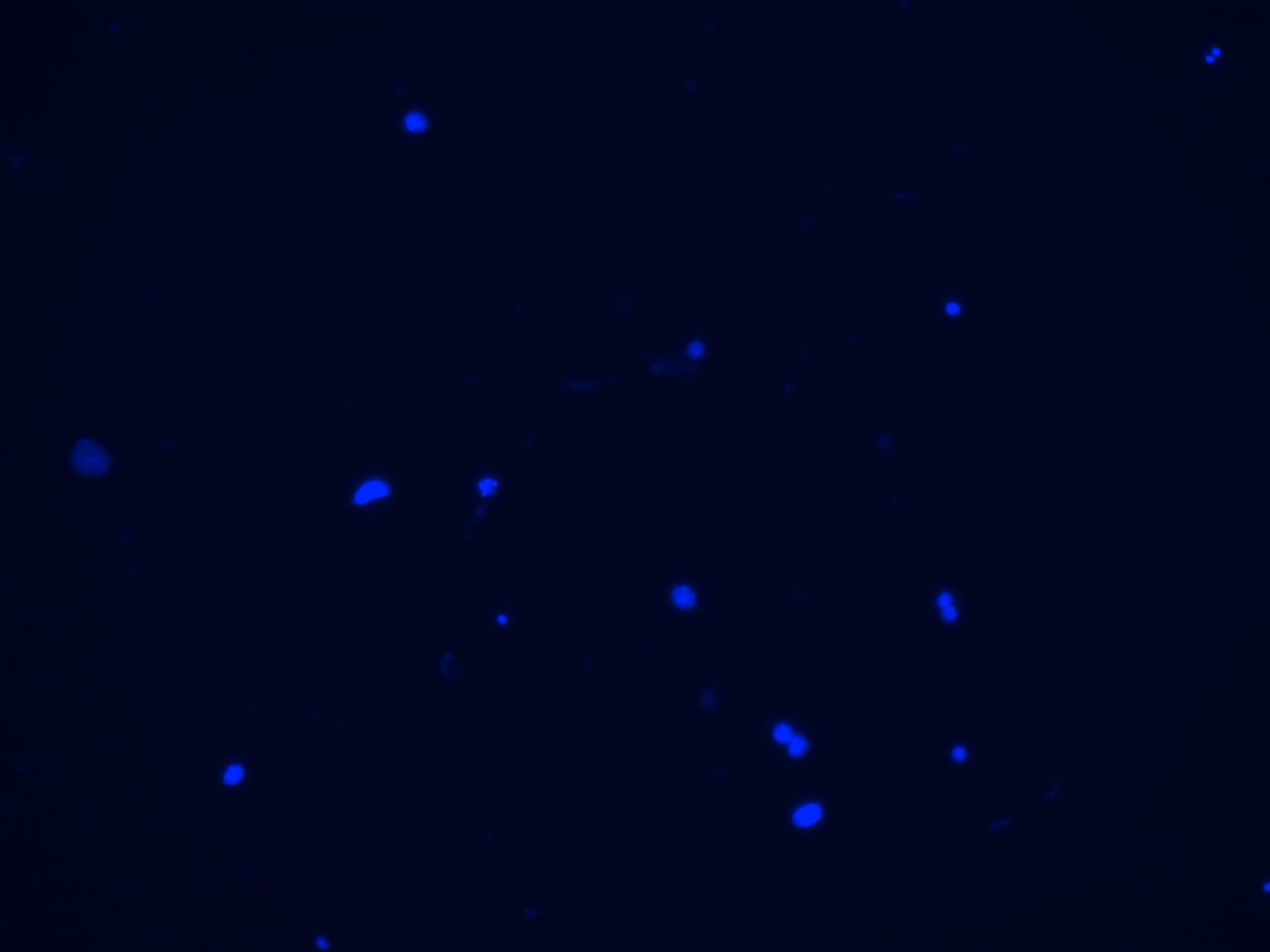

Supplement: Figure 5—figure supplement 1—source data 1. [file elife-73792-fig5-figsupp1-data1.zip › Figure 5-figure supplement 1-source data/1a/DAPI.tif]

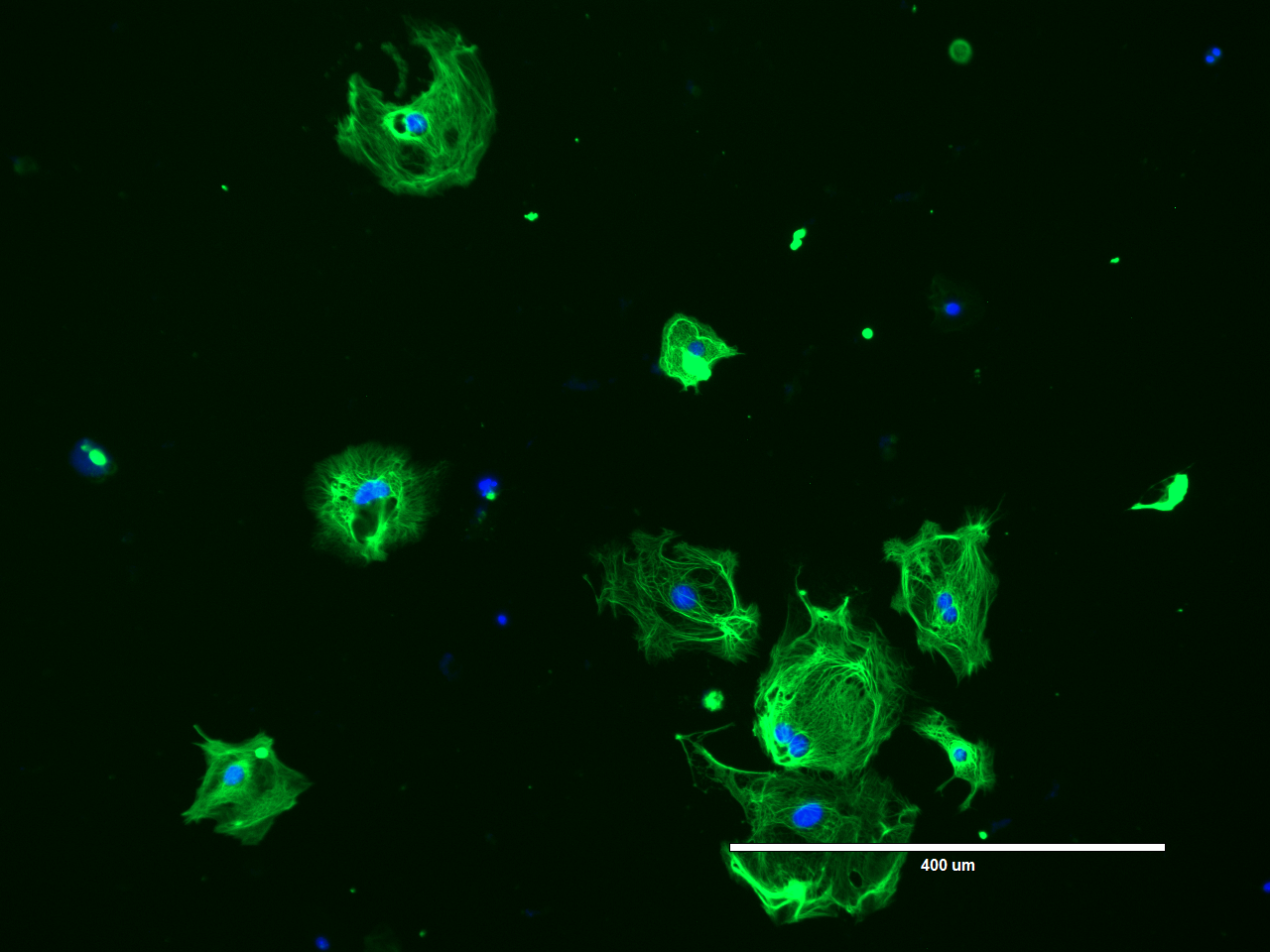

Supplement: Figure 5—figure supplement 1—source data 1. [file elife-73792-fig5-figsupp1-data1.zip › Figure 5-figure supplement 1-source data/1a/MERGE.tif]

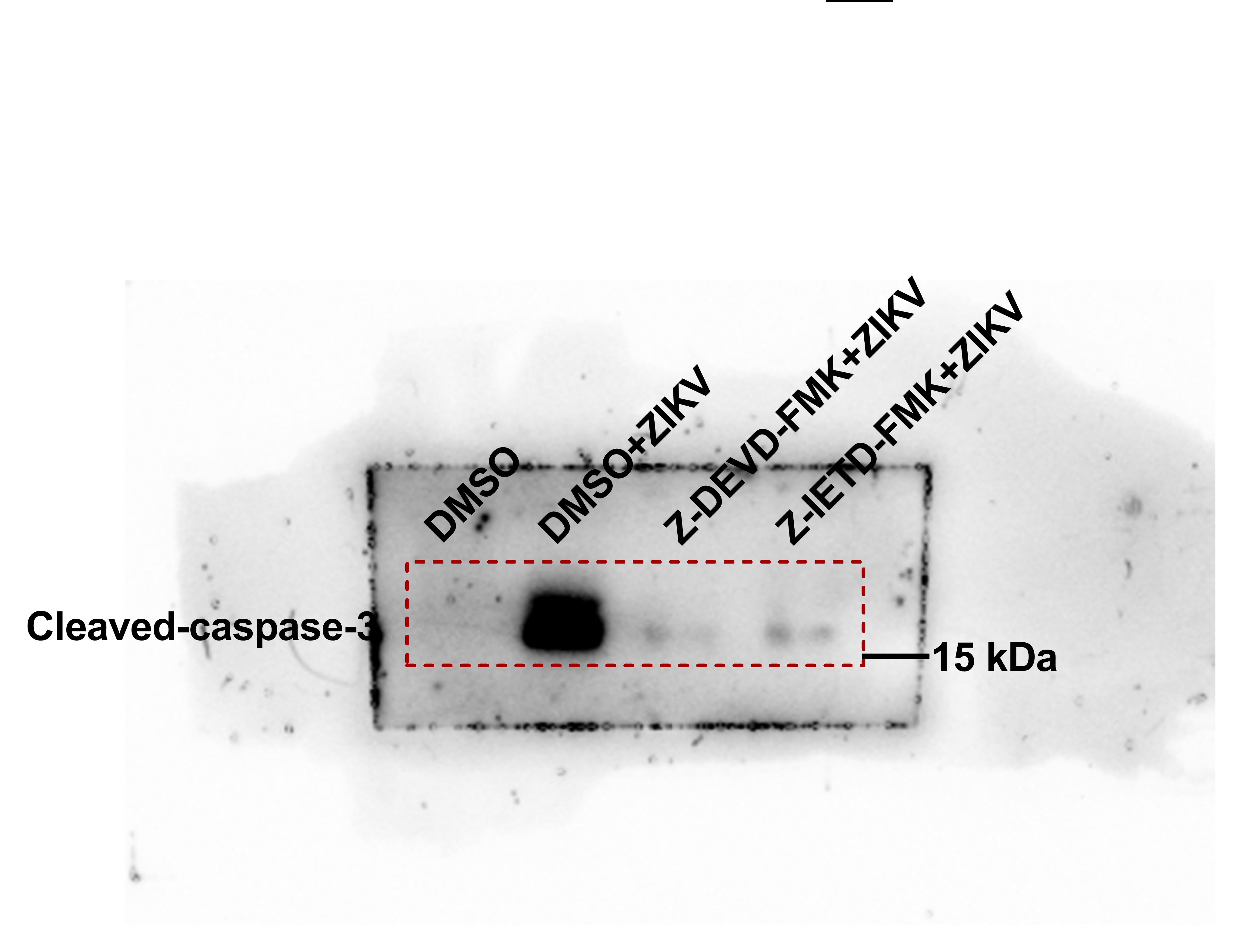

Supplement: Figure 5—figure supplement 1—source data 1. [file elife-73792-fig5-figsupp1-data1.zip › Figure 5-figure supplement 1-source data/1c/Figure 5-figure supplement 1C Cleaved-caspase-3-labeled.tif]

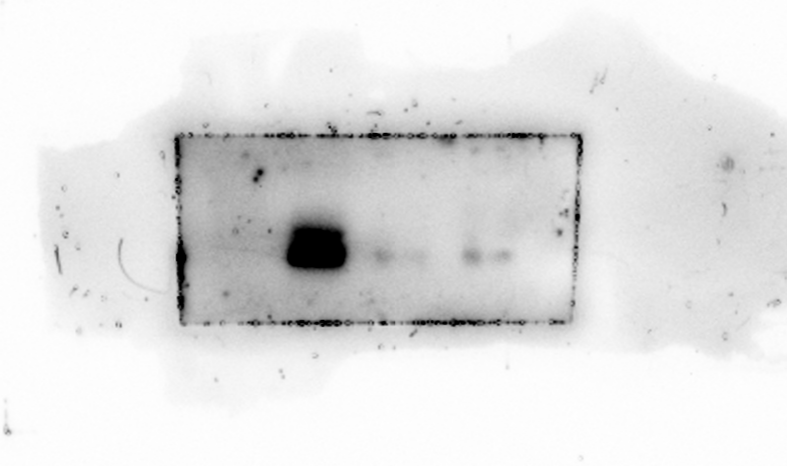

Supplement: Figure 5—figure supplement 1—source data 1. [file elife-73792-fig5-figsupp1-data1.zip › Figure 5-figure supplement 1-source data/1c/Figure 5-figure supplement 1C Cleaved-caspase-3-raw.Tif]

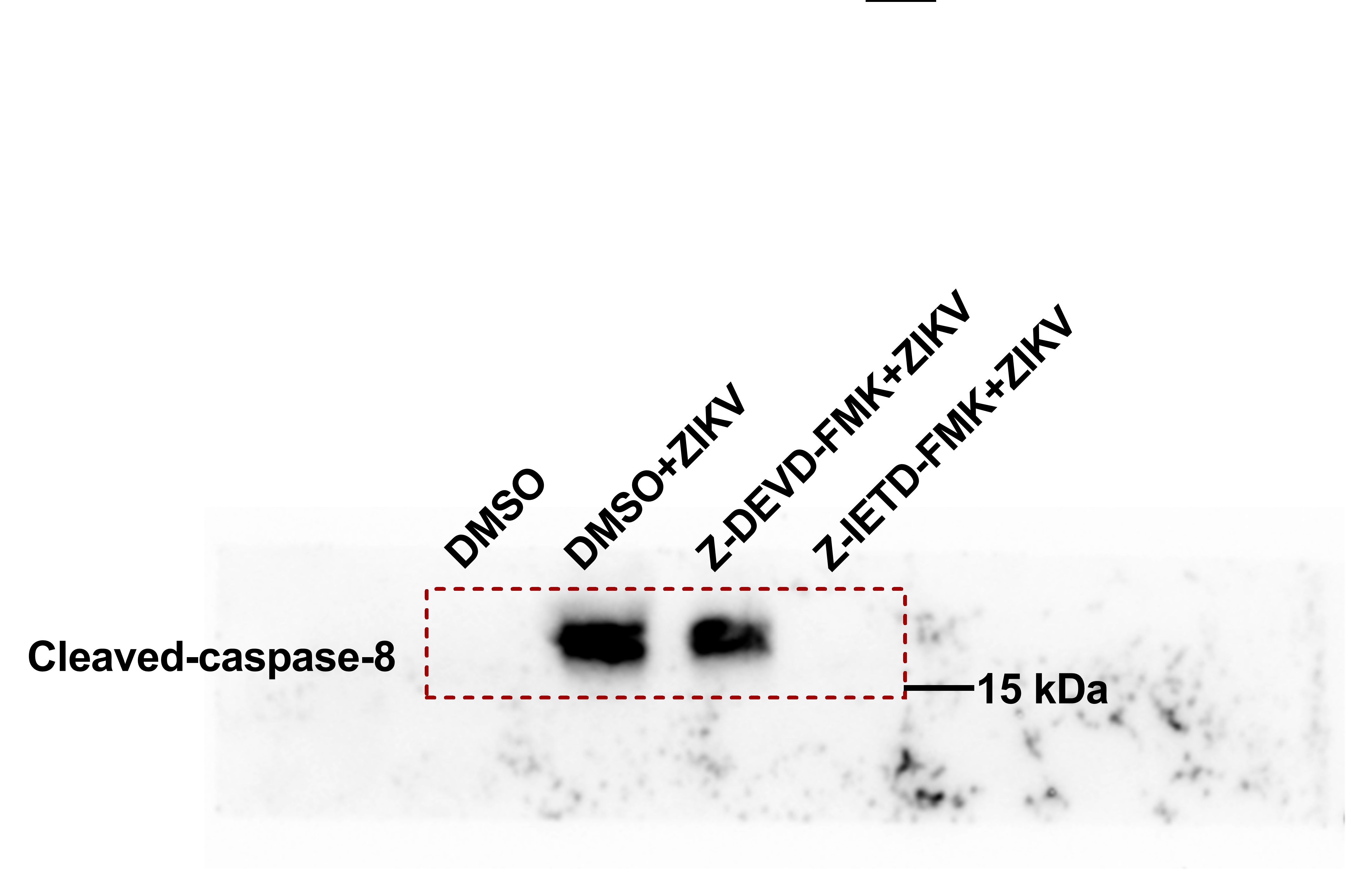

Supplement: Figure 5—figure supplement 1—source data 1. [file elife-73792-fig5-figsupp1-data1.zip › Figure 5-figure supplement 1-source data/1c/Figure 5-figure supplement 1C Cleaved-caspase-8-labeled.tif]

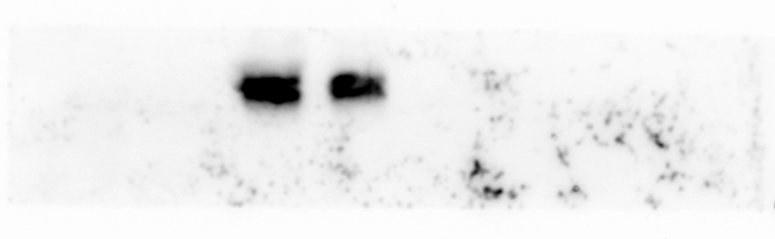

Supplement: Figure 5—figure supplement 1—source data 1. [file elife-73792-fig5-figsupp1-data1.zip › Figure 5-figure supplement 1-source data/1c/Figure 5-figure supplement 1C Cleaved-caspase-8-raw.tif]

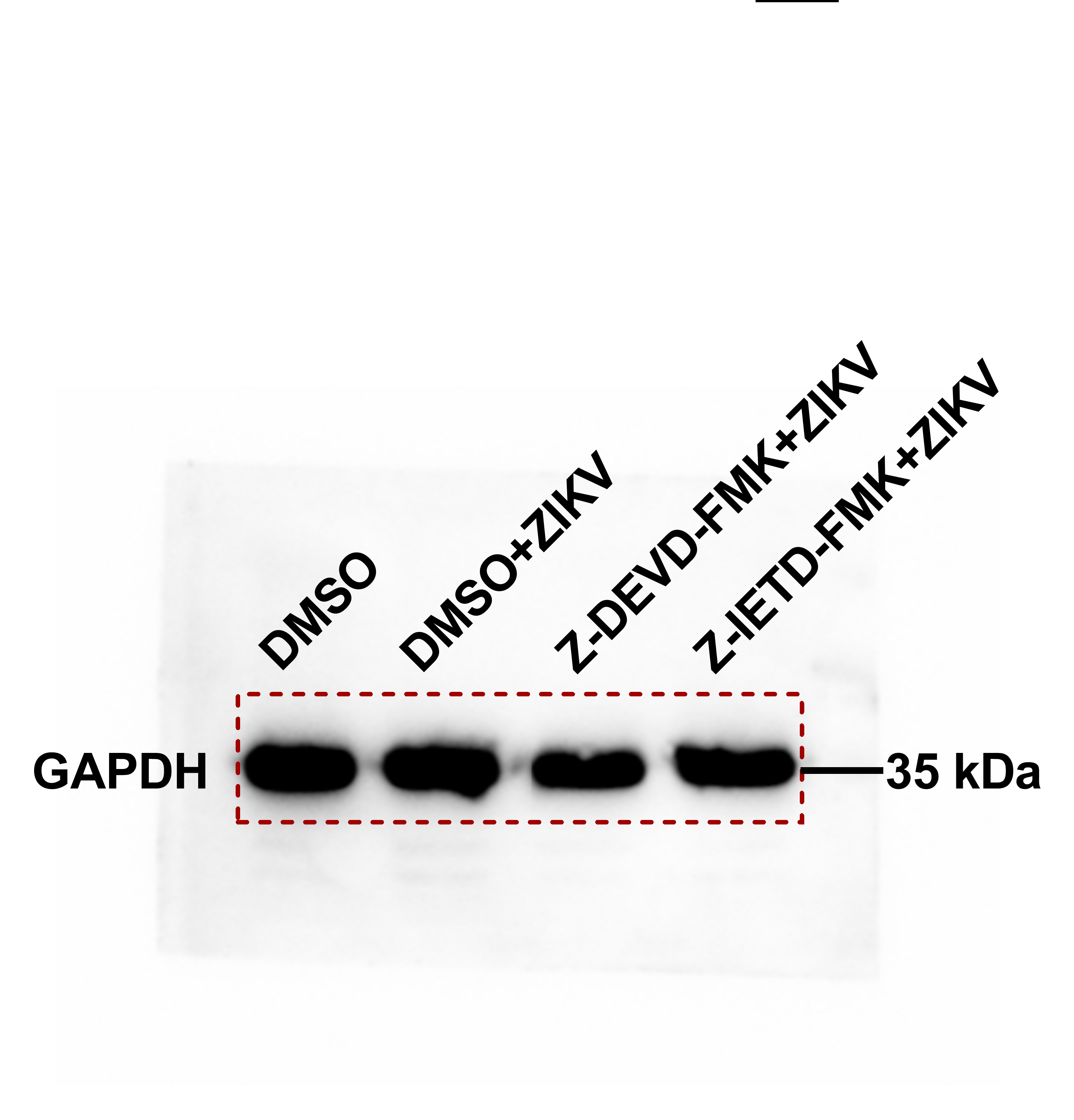

Supplement: Figure 5—figure supplement 1—source data 1. [file elife-73792-fig5-figsupp1-data1.zip › Figure 5-figure supplement 1-source data/1c/Figure 5-figure supplement 1C GAPDH-labeled.tif]

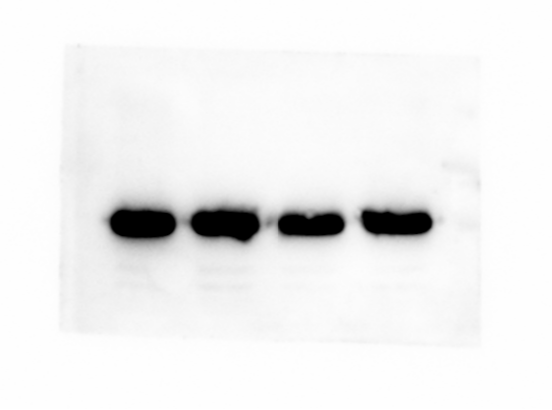

Supplement: Figure 5—figure supplement 1—source data 1. [file elife-73792-fig5-figsupp1-data1.zip › Figure 5-figure supplement 1-source data/1c/Figure 5-figure supplement 1C GAPDH-raw.tif]

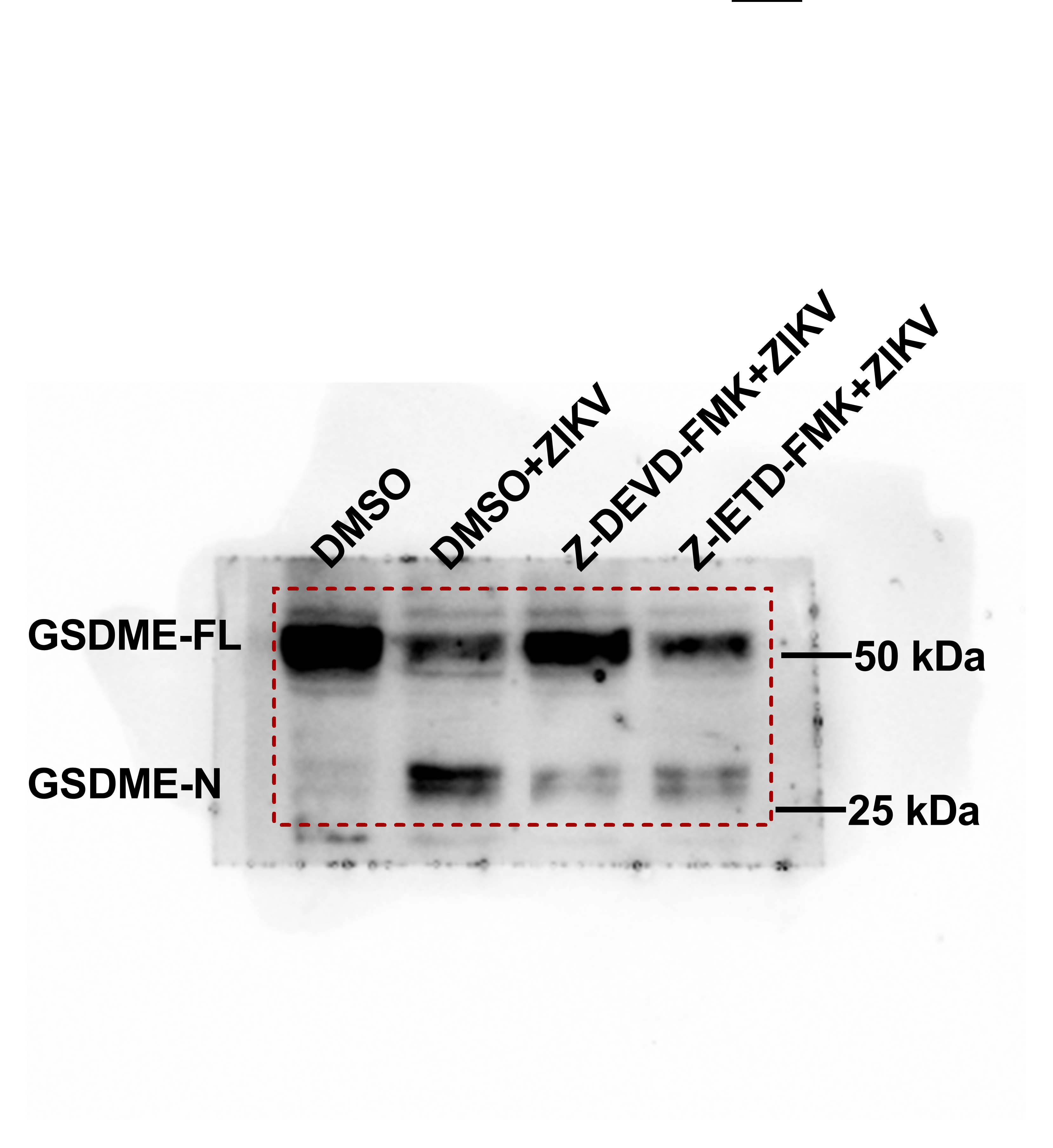

Supplement: Figure 5—figure supplement 1—source data 1. [file elife-73792-fig5-figsupp1-data1.zip › Figure 5-figure supplement 1-source data/1c/Figure 5-figure supplement 1C GSDME-LABELED.tif]

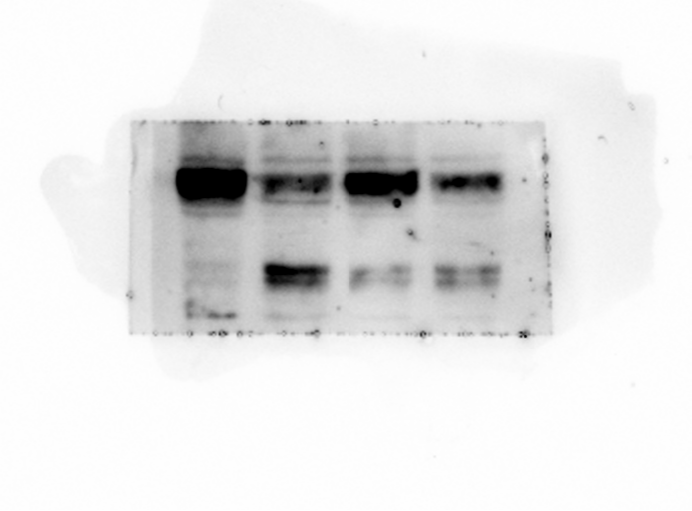

Supplement: Figure 5—figure supplement 1—source data 1. [file elife-73792-fig5-figsupp1-data1.zip › Figure 5-figure supplement 1-source data/1c/Figure 5-figure supplement 1C GSDME-raw.tif]

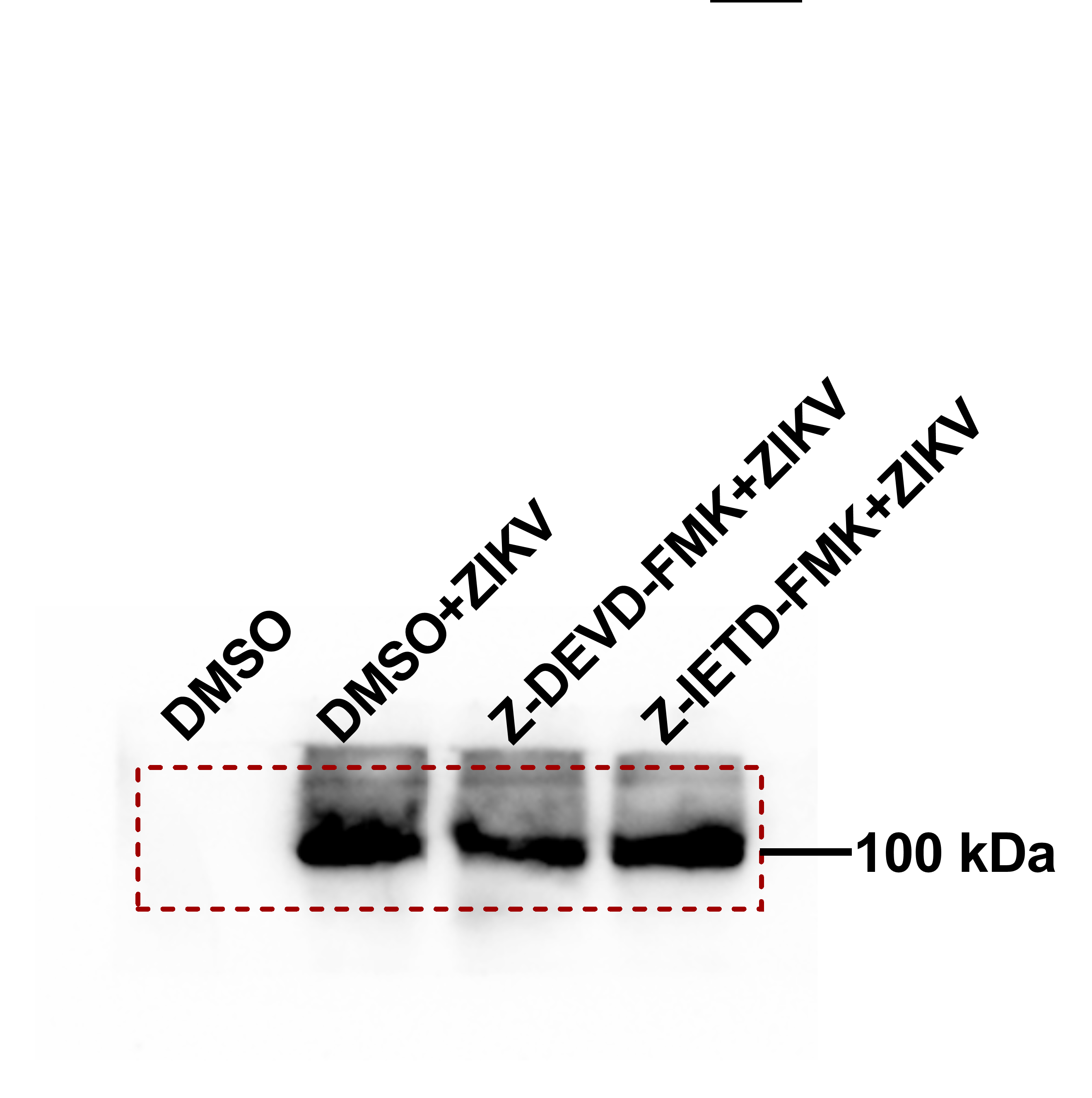

Supplement: Figure 5—figure supplement 1—source data 1. [file elife-73792-fig5-figsupp1-data1.zip › Figure 5-figure supplement 1-source data/1c/Figure 5-figure supplement 1C NS5-labeled.tif]

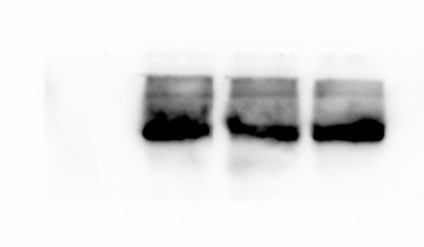

Supplement: Figure 5—figure supplement 1—source data 1. [file elife-73792-fig5-figsupp1-data1.zip › Figure 5-figure supplement 1-source data/1c/Figure 5-figure supplement 1C NS5-raw.tif]

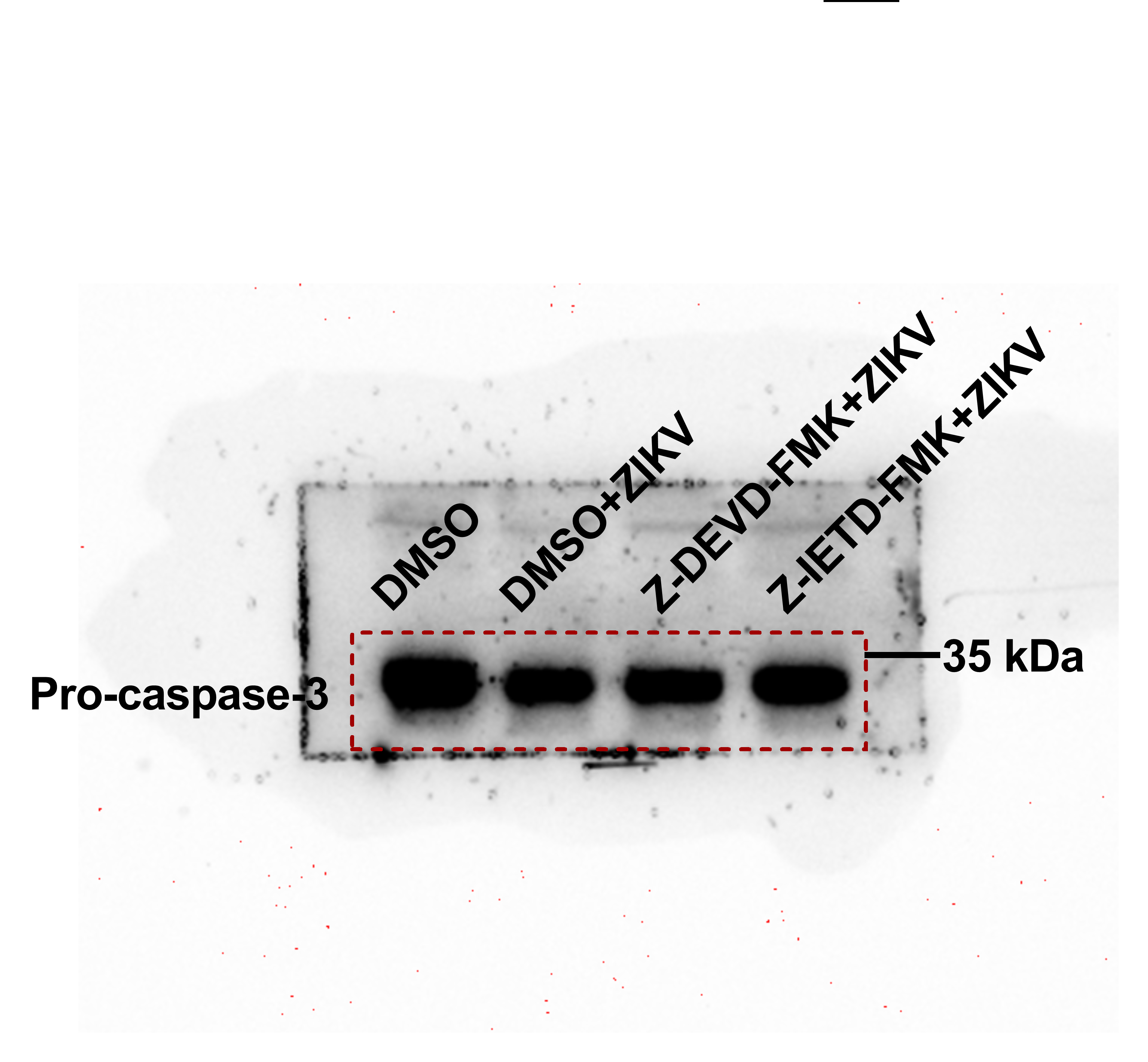

Supplement: Figure 5—figure supplement 1—source data 1. [file elife-73792-fig5-figsupp1-data1.zip › Figure 5-figure supplement 1-source data/1c/Figure 5-figure supplement 1C Pro-caspase-3-labeled.tif]

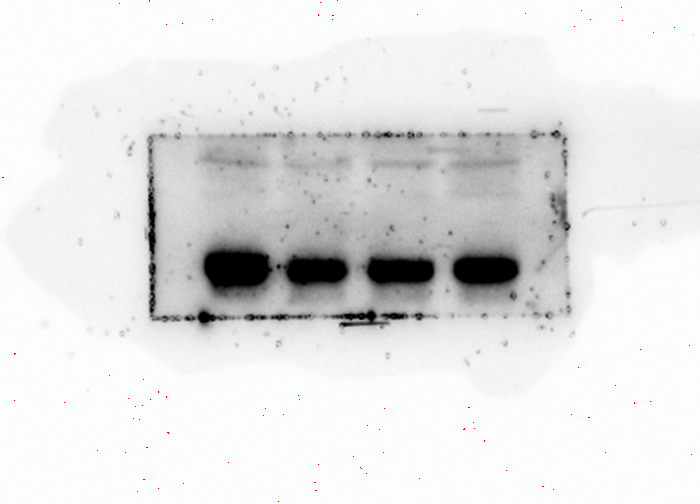

Supplement: Figure 5—figure supplement 1—source data 1. [file elife-73792-fig5-figsupp1-data1.zip › Figure 5-figure supplement 1-source data/1c/Figure 5-figure supplement 1C Pro-caspase-3-raw.tif]

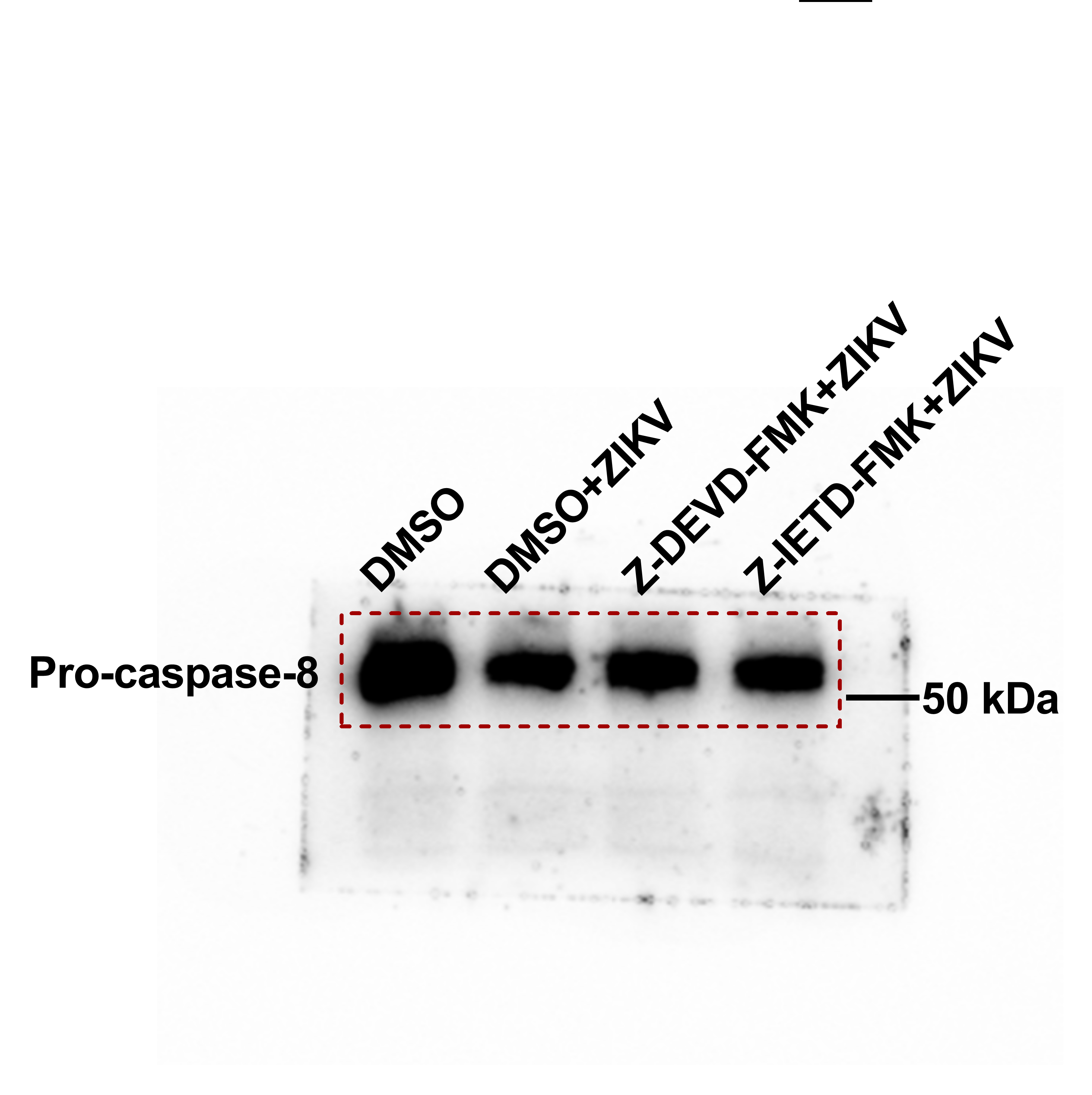

Supplement: Figure 5—figure supplement 1—source data 1. [file elife-73792-fig5-figsupp1-data1.zip › Figure 5-figure supplement 1-source data/1c/Figure 5-figure supplement 1C Pro-caspase-8-labeled.tif]

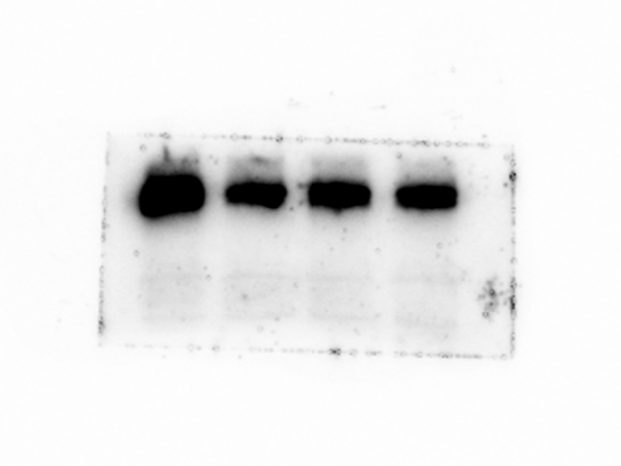

Supplement: Figure 5—figure supplement 1—source data 1. [file elife-73792-fig5-figsupp1-data1.zip › Figure 5-figure supplement 1-source data/1c/Figure 5-figure supplement 1C Pro-caspase-8-raw.tif]

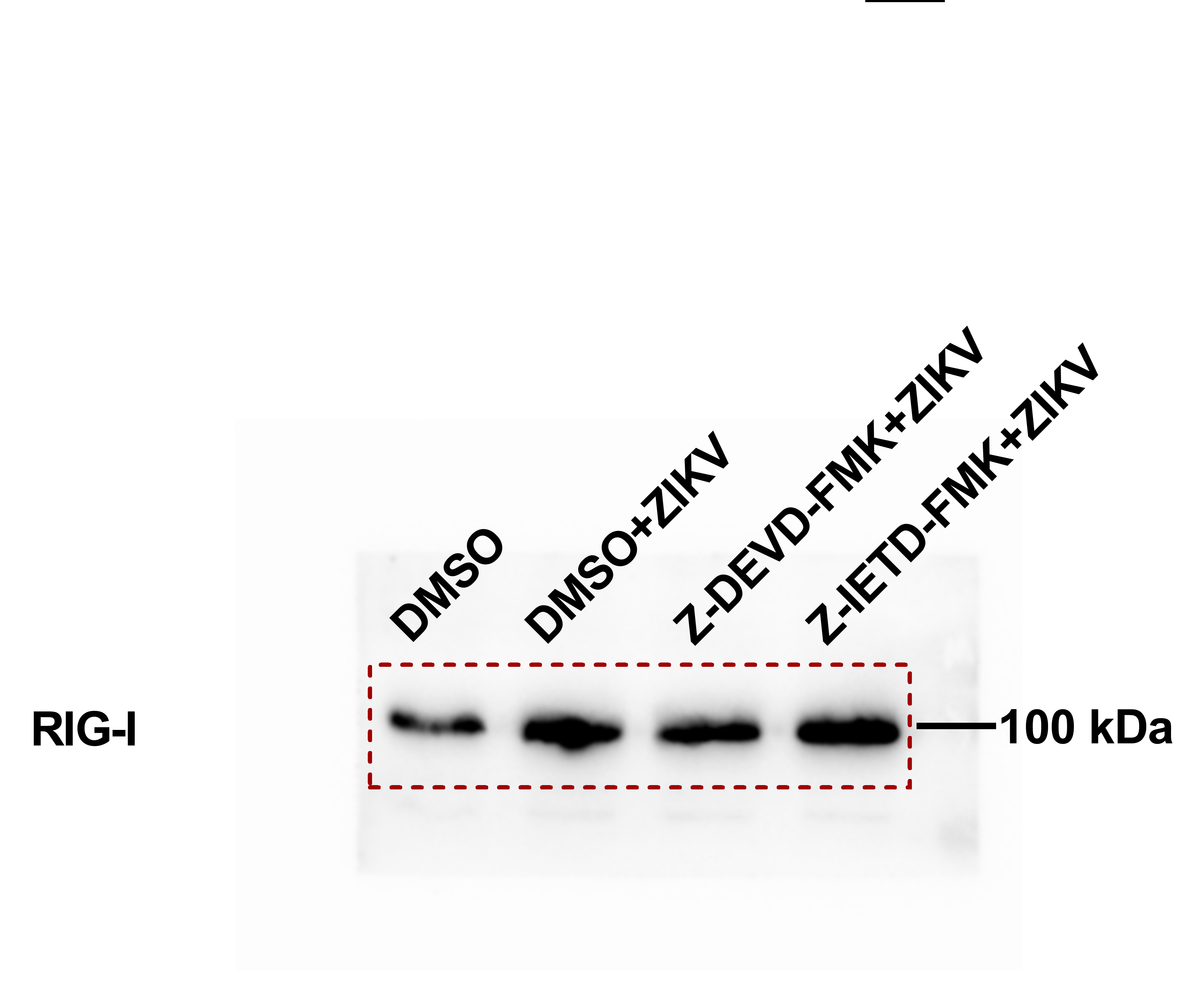

Supplement: Figure 5—figure supplement 1—source data 1. [file elife-73792-fig5-figsupp1-data1.zip › Figure 5-figure supplement 1-source data/1c/Figure 5-figure supplement 1C RIG-I-labeled.tif]

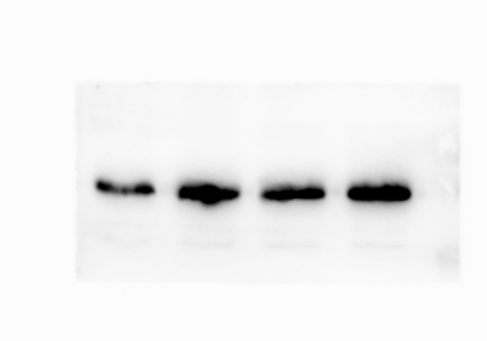

Supplement: Figure 5—figure supplement 1—source data 1. [file elife-73792-fig5-figsupp1-data1.zip › Figure 5-figure supplement 1-source data/1c/Figure 5-figure supplement 1C RIG-I-raw.tif]

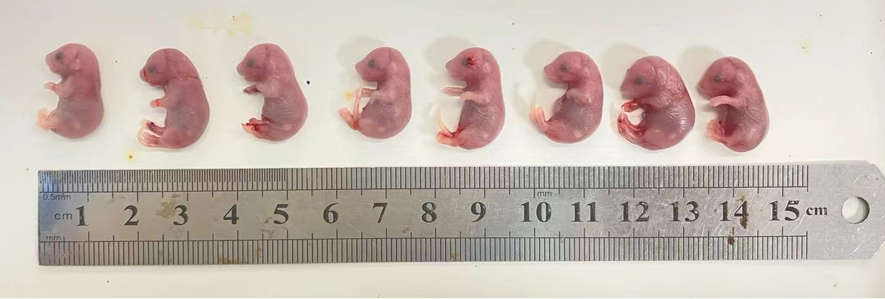

Supplement: Figure 6—source data 1. [file elife-73792-fig6-data1.zip › Figure 6-source data 1/Fig 6C/R-7050+ZIKV.tif]

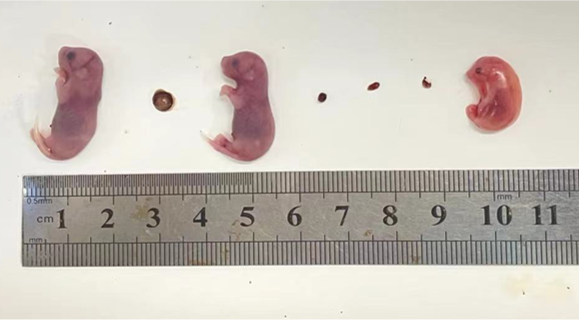

Supplement: Figure 6—source data 1. [file elife-73792-fig6-data1.zip › Figure 6-source data 1/Fig 6C/vehicle+ZIKV.tif]

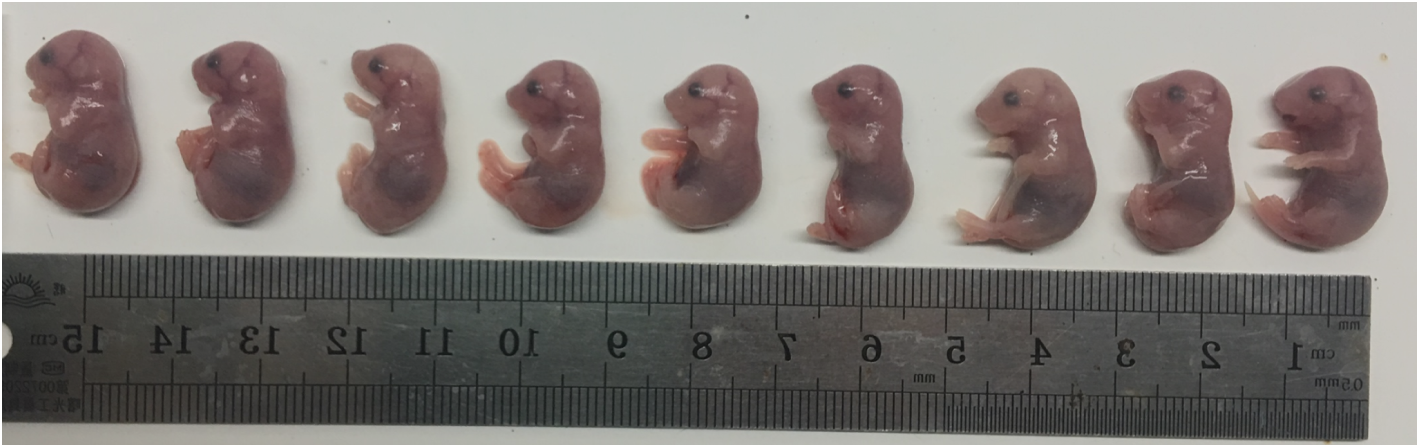

Supplement: Figure 6—source data 1. [file elife-73792-fig6-data1.zip › Figure 6-source data 1/Fig 6C/vehicle.tif]

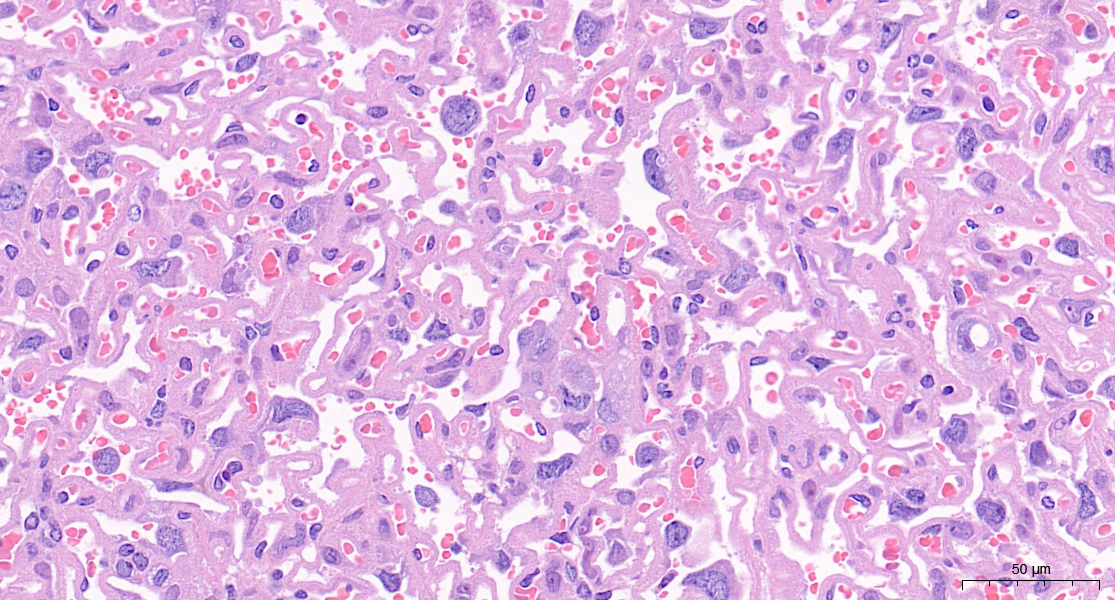

Supplement: Figure 6—source data 1. [file elife-73792-fig6-data1.zip › Figure 6-source data 1/Fig 6E/R-7050+ZIKV.tif]

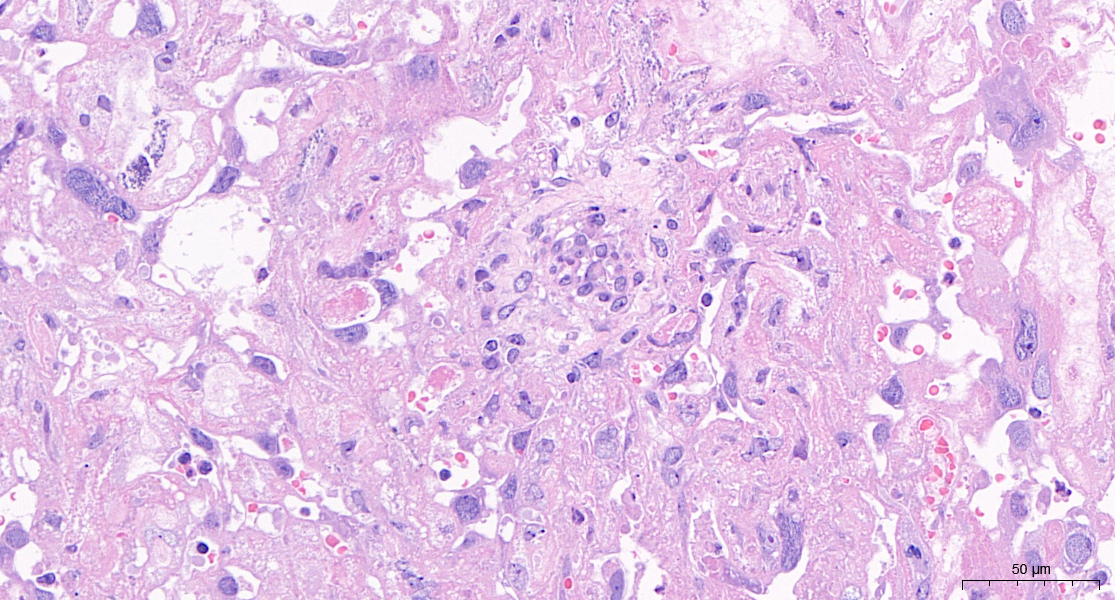

Supplement: Figure 6—source data 1. [file elife-73792-fig6-data1.zip › Figure 6-source data 1/Fig 6E/vehicle+zikv.tif]

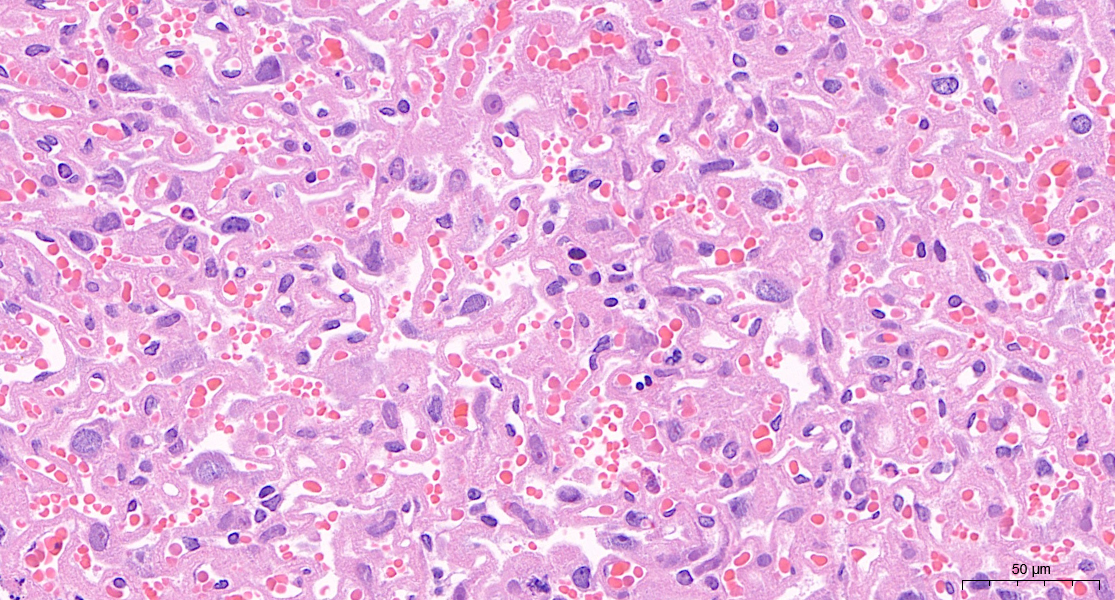

Supplement: Figure 6—source data 1. [file elife-73792-fig6-data1.zip › Figure 6-source data 1/Fig 6E/vehicle.tif]

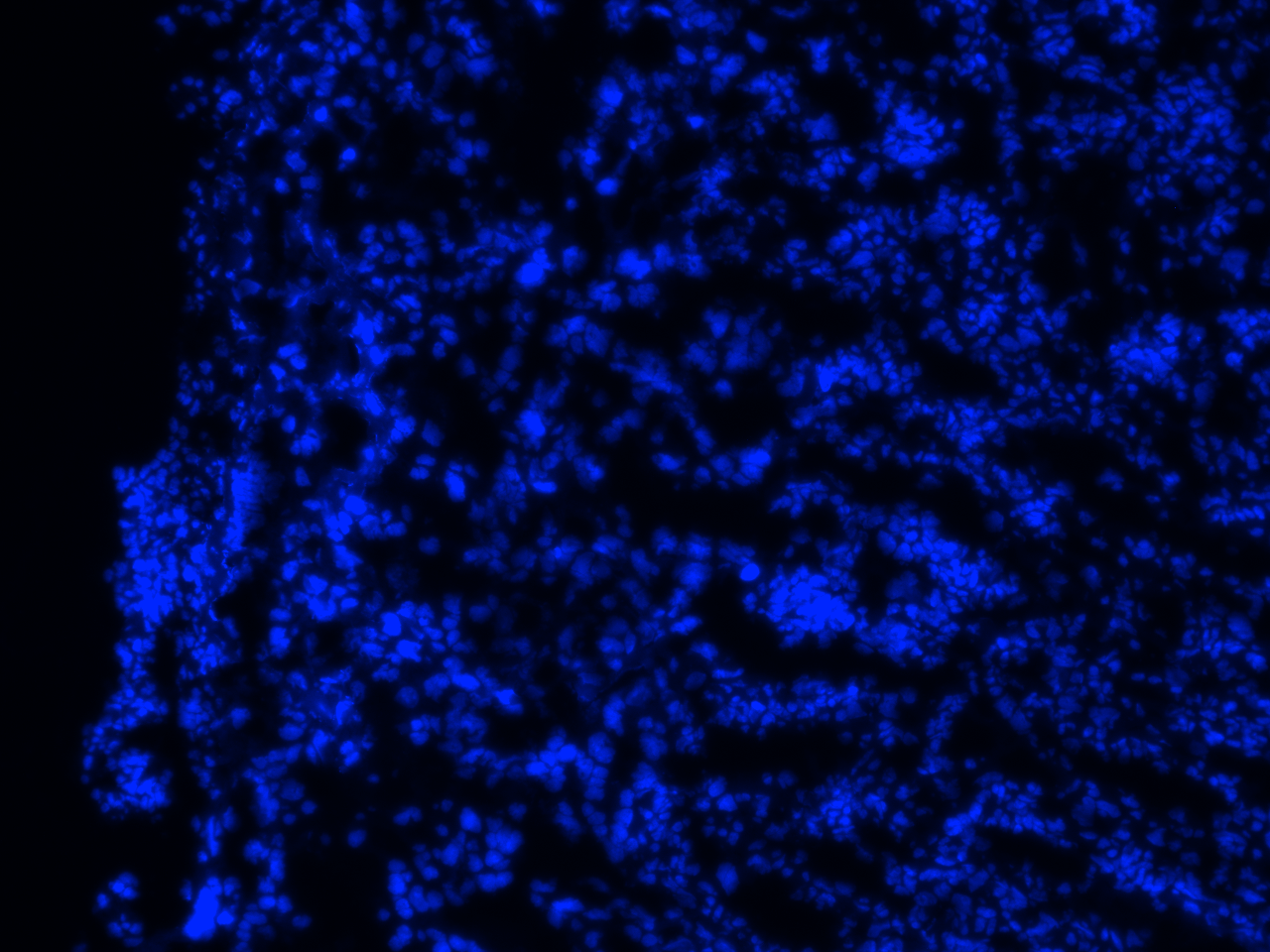

Supplement: Figure 6—source data 1. [file elife-73792-fig6-data1.zip › Figure 6-source data 1/Fig 6F/r-7050+zikv-DAPI.tif]

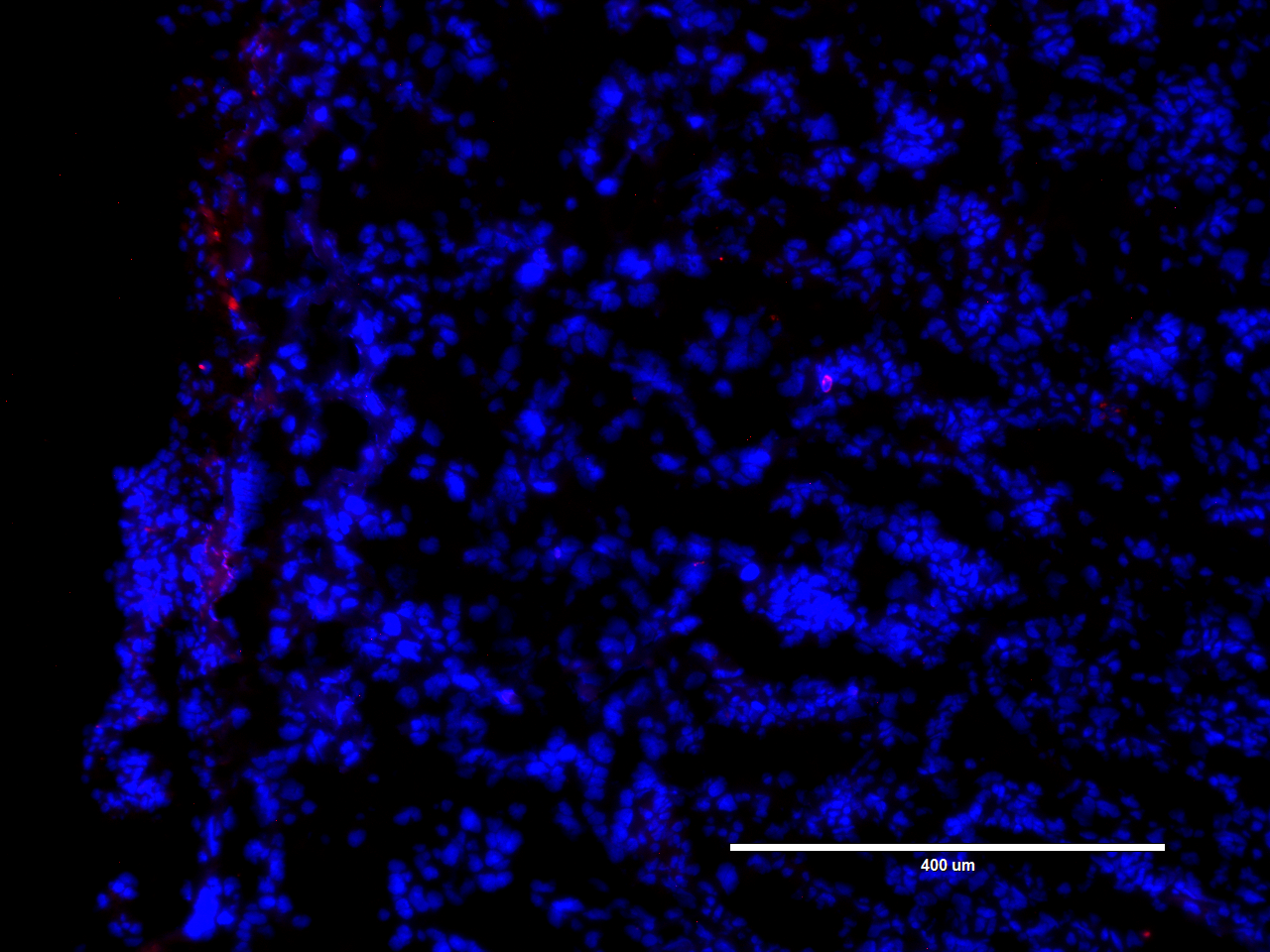

Supplement: Figure 6—source data 1. [file elife-73792-fig6-data1.zip › Figure 6-source data 1/Fig 6F/r-7050+zikv-Merge.tif]

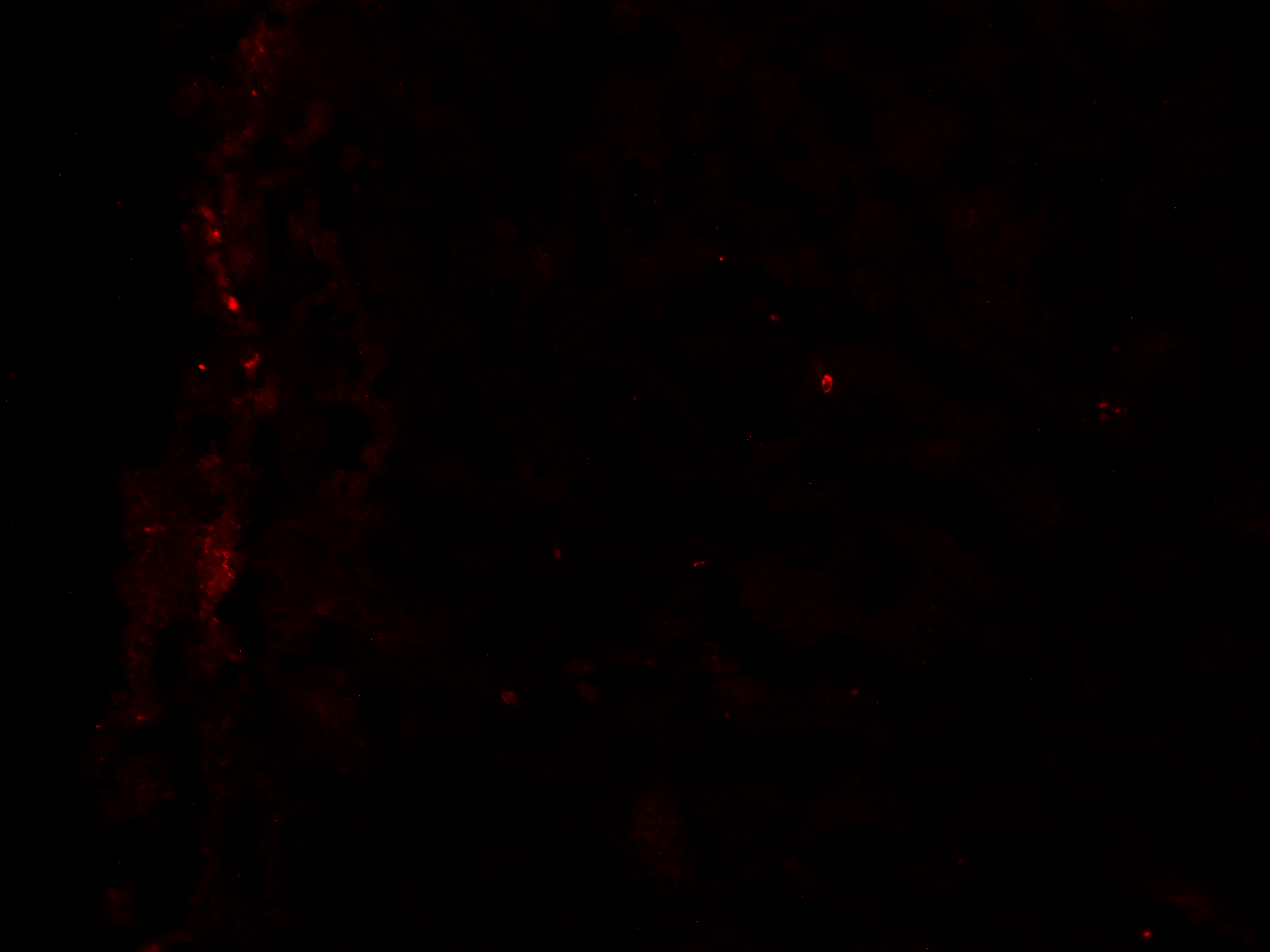

Supplement: Figure 6—source data 1. [file elife-73792-fig6-data1.zip › Figure 6-source data 1/Fig 6F/r-7050+zikv-PI.tif]

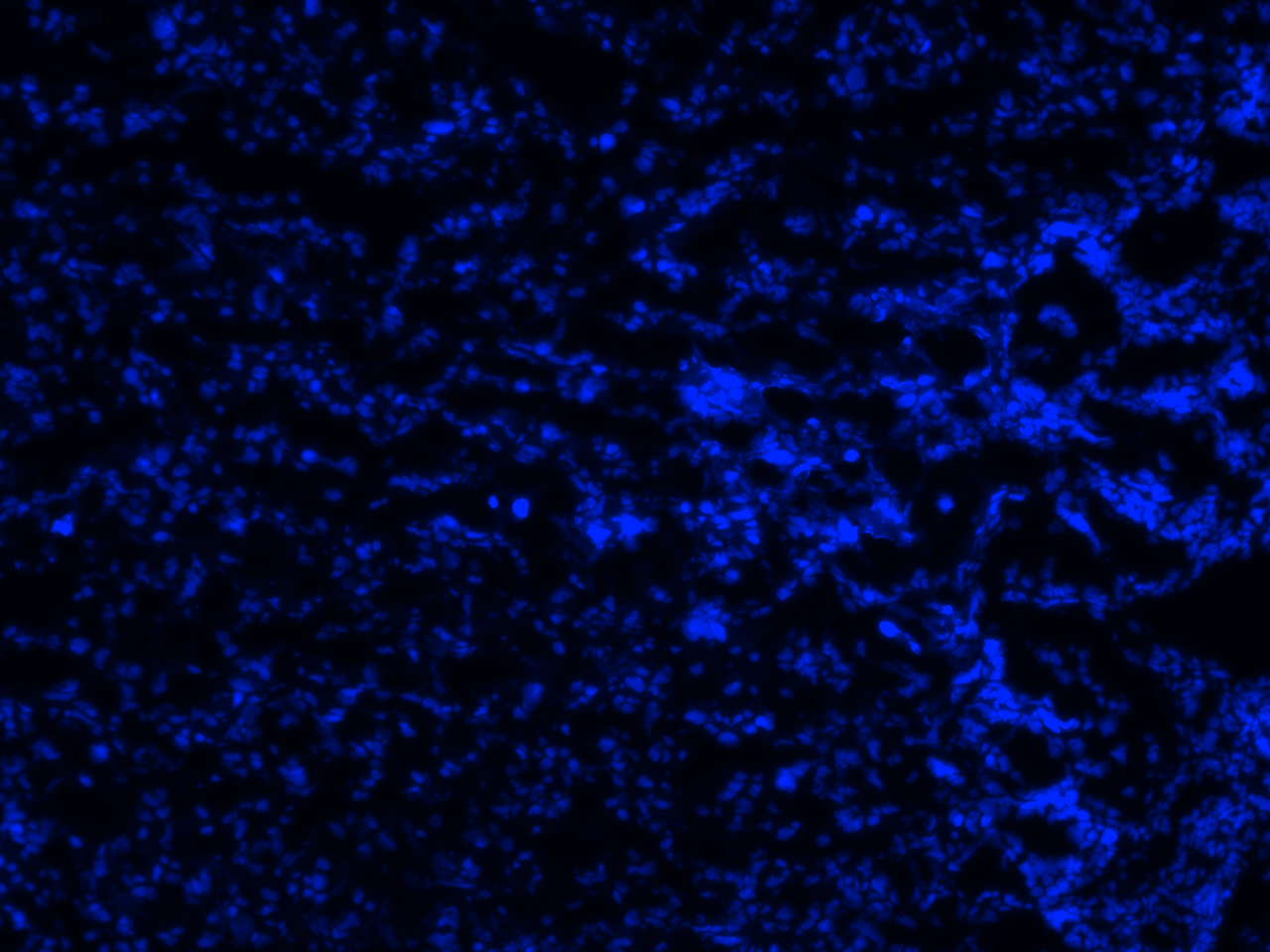

Supplement: Figure 6—source data 1. [file elife-73792-fig6-data1.zip › Figure 6-source data 1/Fig 6F/vehicle+zikv-DAPI.tif]

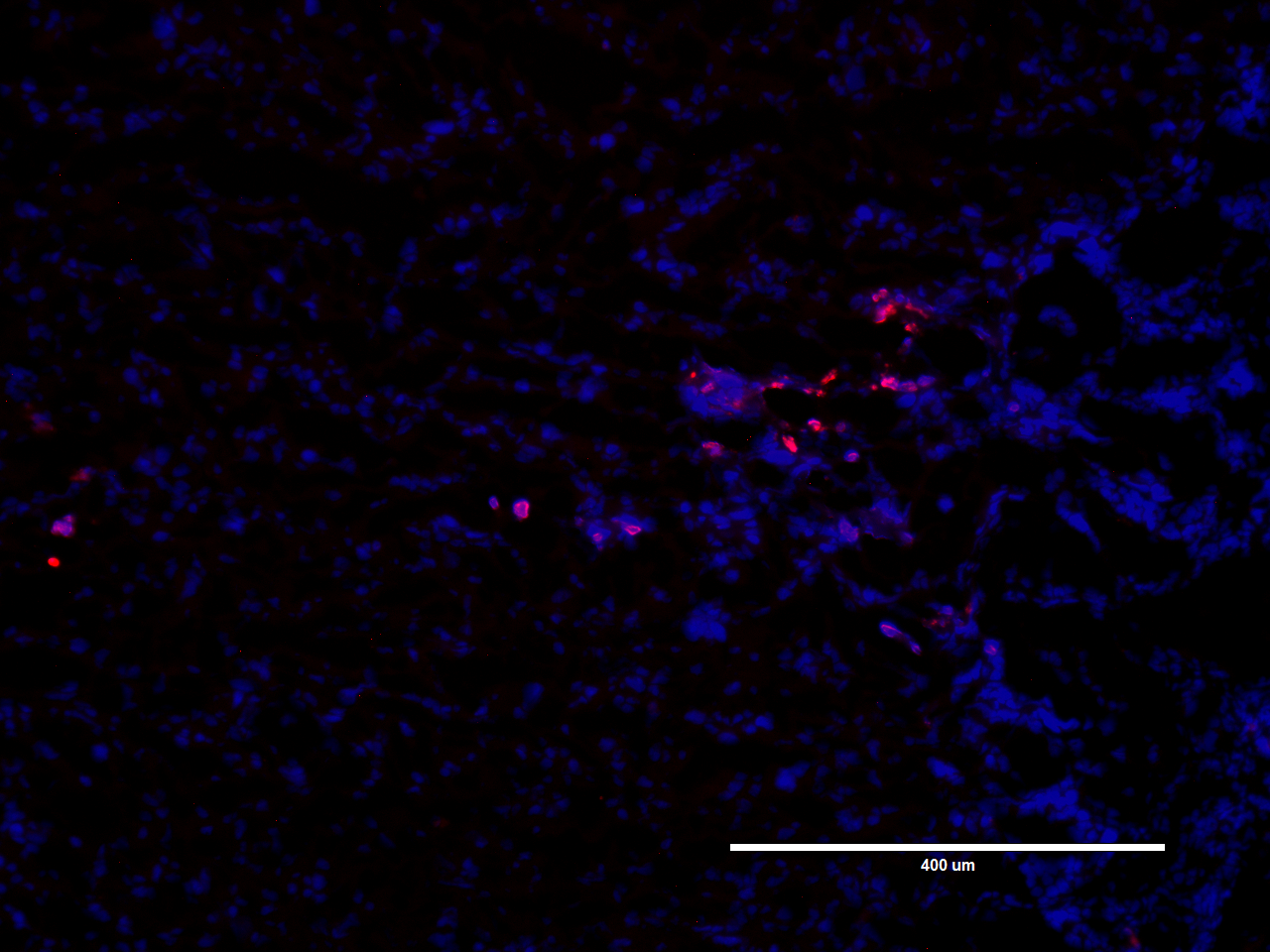

Supplement: Figure 6—source data 1. [file elife-73792-fig6-data1.zip › Figure 6-source data 1/Fig 6F/vehicle+zikv-Merge.tif]

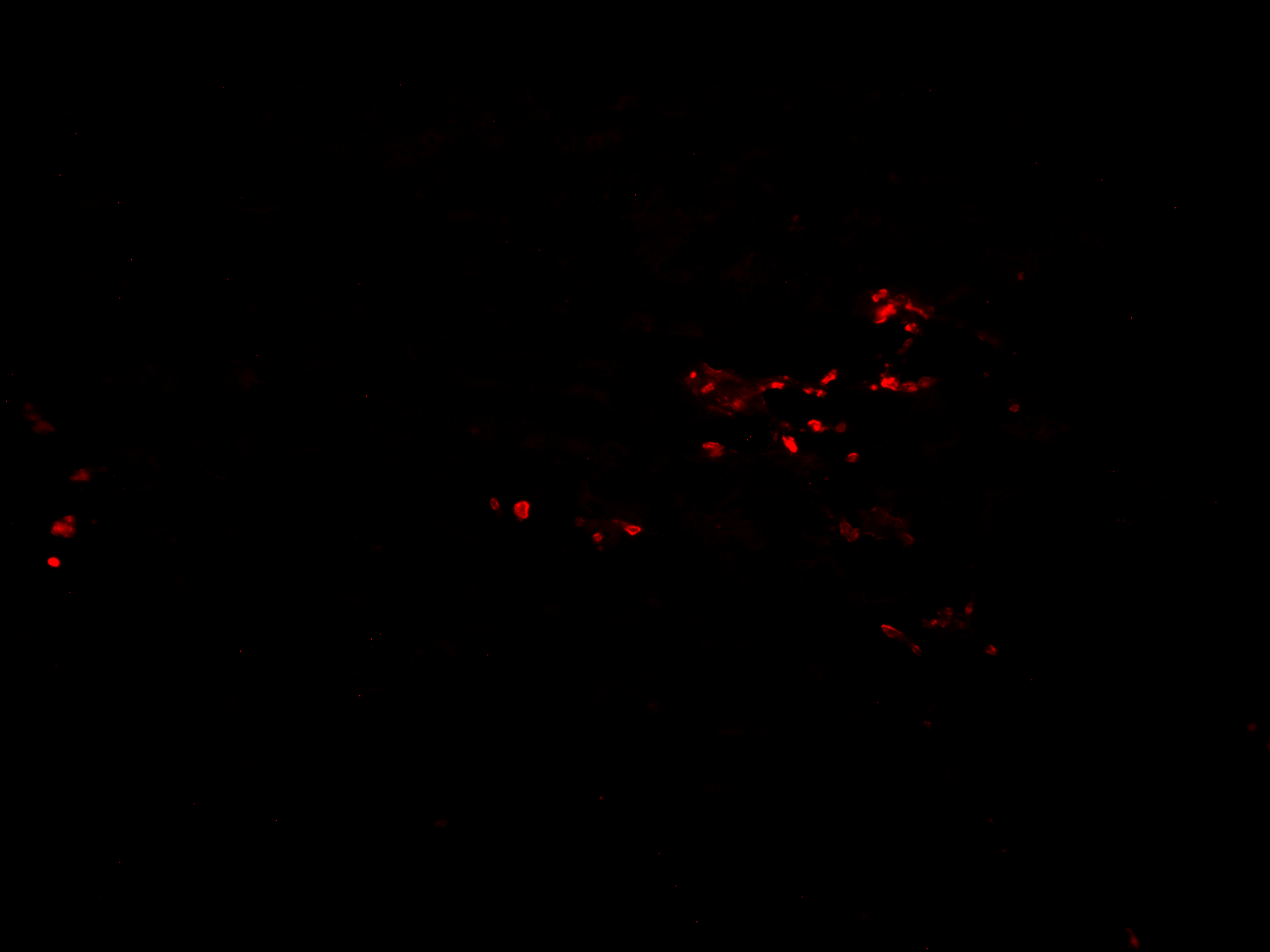

Supplement: Figure 6—source data 1. [file elife-73792-fig6-data1.zip › Figure 6-source data 1/Fig 6F/vehicle+zikv-PI.tif]

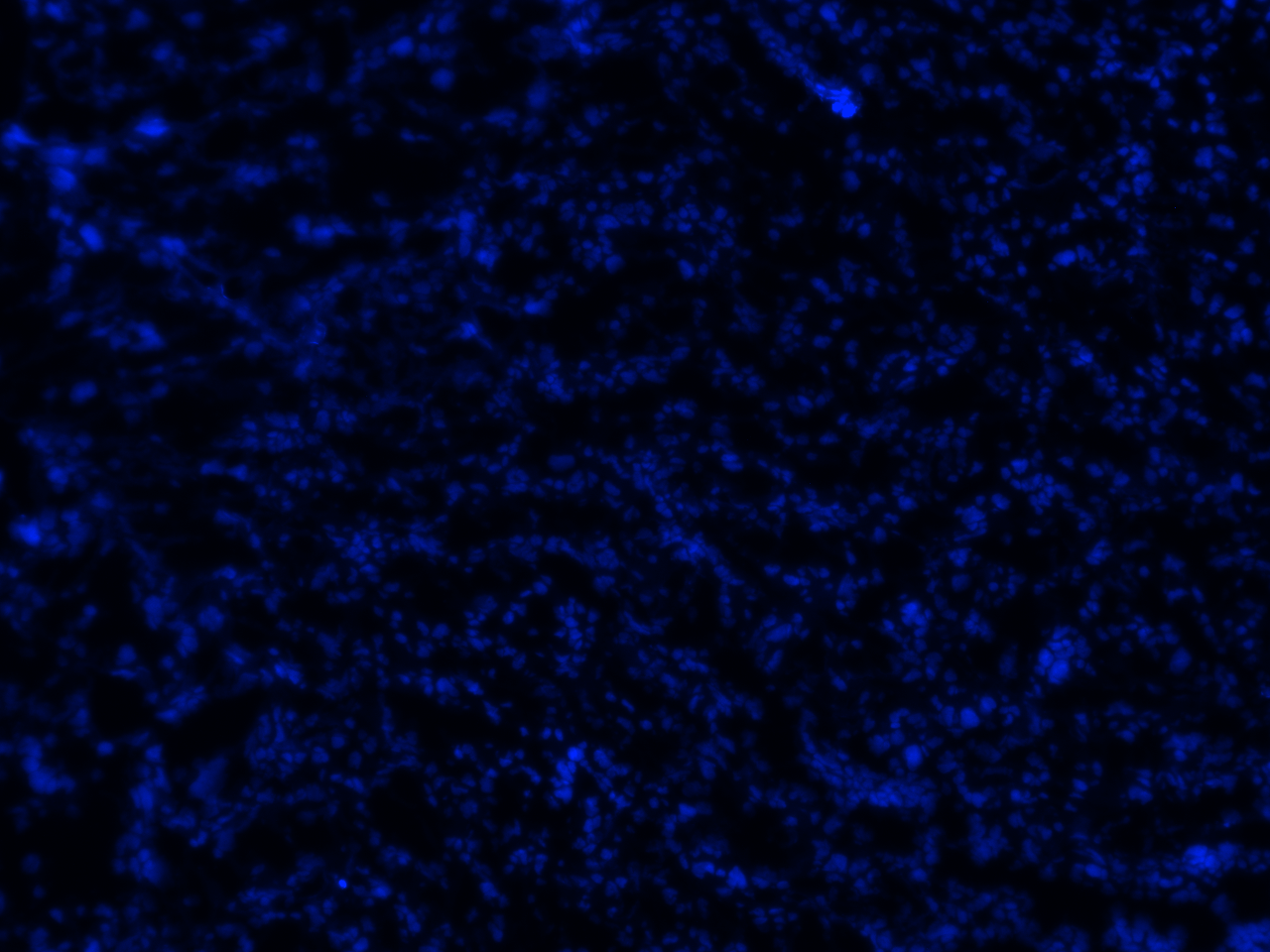

Supplement: Figure 6—source data 1. [file elife-73792-fig6-data1.zip › Figure 6-source data 1/Fig 6F/vehicle-DAPI.tif]

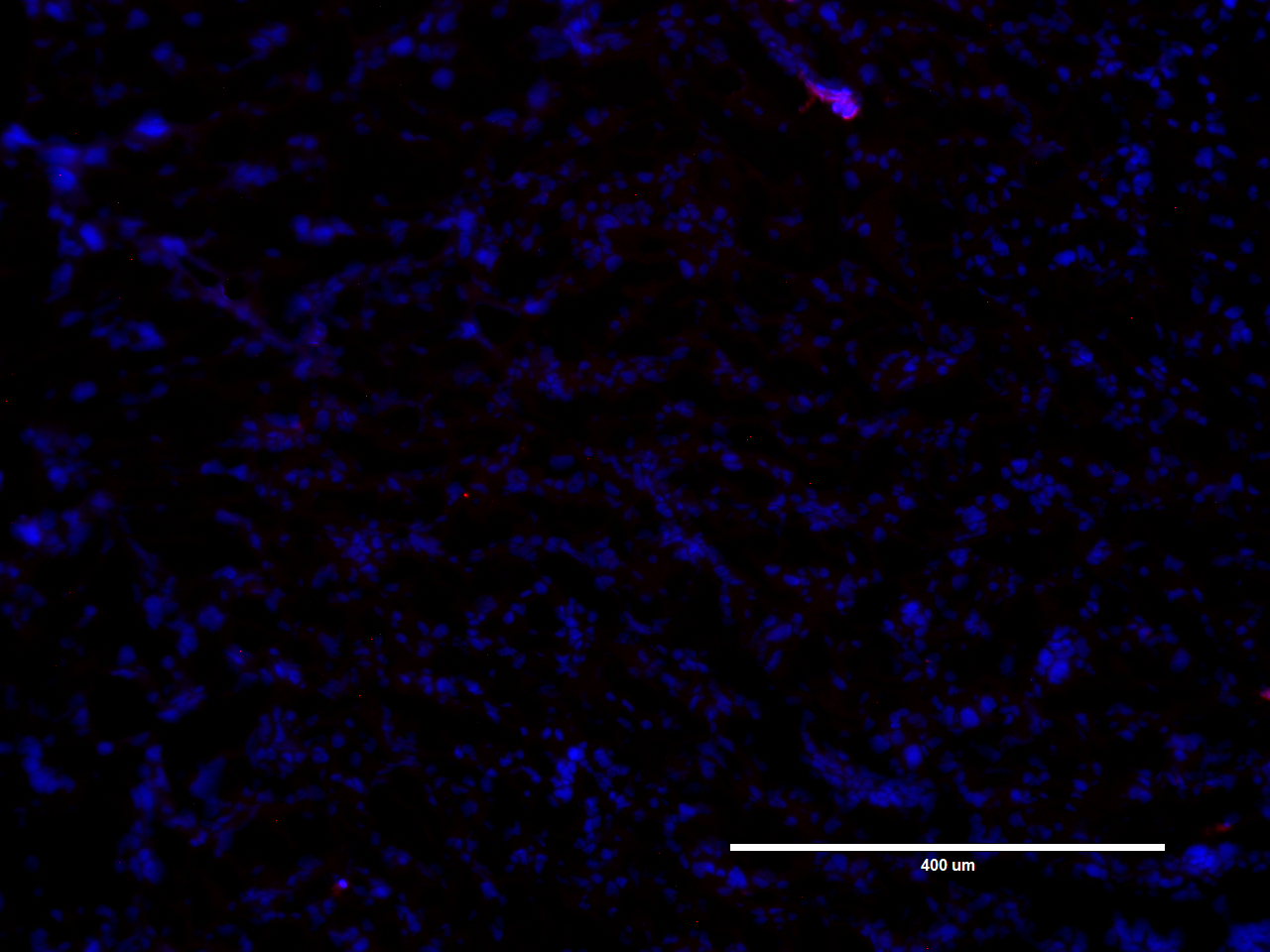

Supplement: Figure 6—source data 1. [file elife-73792-fig6-data1.zip › Figure 6-source data 1/Fig 6F/vehicle-Merge.tif]

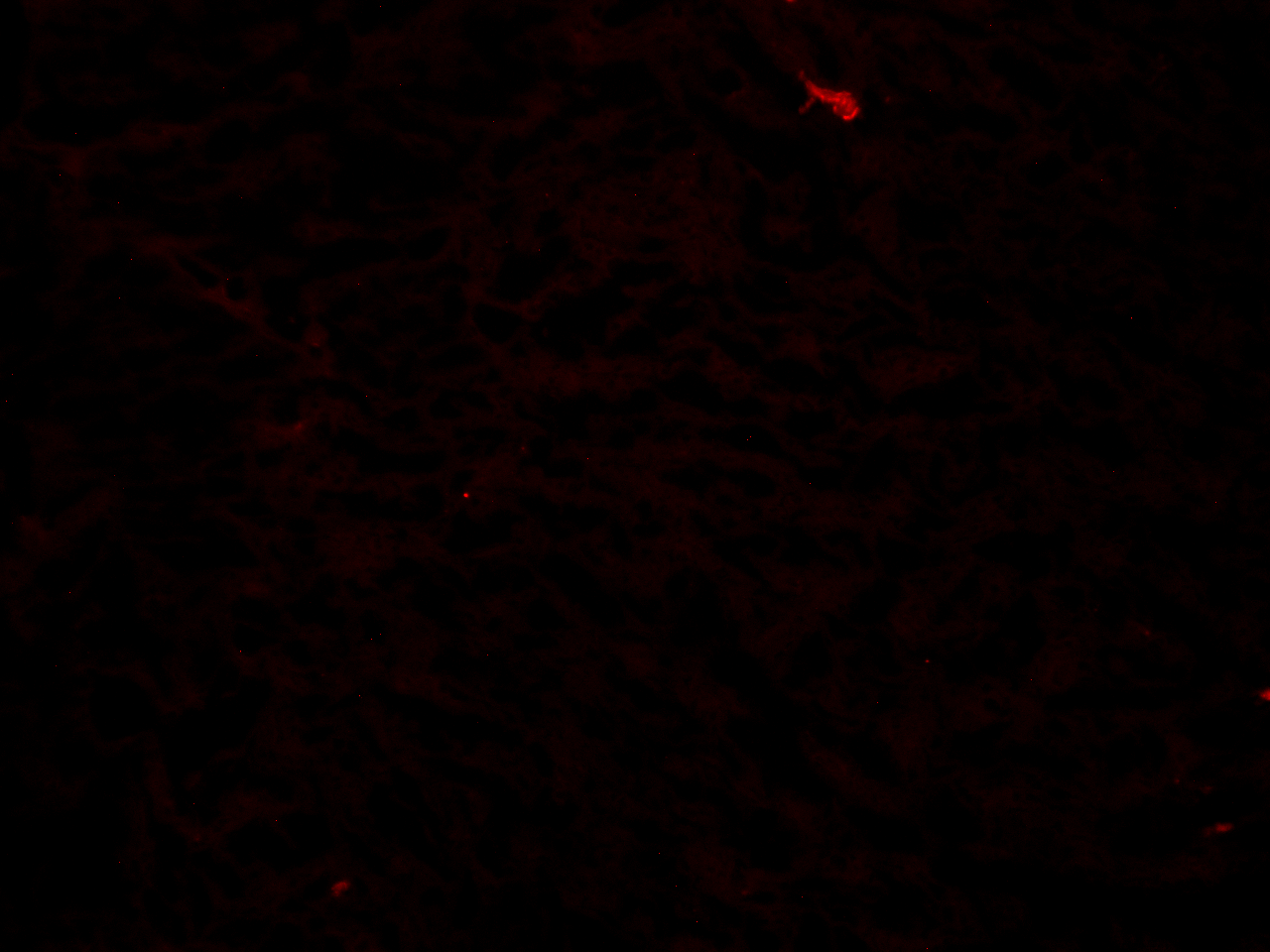

Supplement: Figure 6—source data 1. [file elife-73792-fig6-data1.zip › Figure 6-source data 1/Fig 6F/vehicle-PI.tif]
